# Cobalt-Stabilized Propargylic Oxocarbenium Ions Enable Direct and Asymmetric Nickel(II) Catalyzed Aldol-Like Reactions

**DOI:** 10.1021/acs.orglett.6c02417

**Published:** 2026-07-03

**Authors:** Xènia Tarrach, Leah O’Neill, Anna M. Costa, Pedro Romea, Fèlix Urpí, Laura Sánchez-Castillo, Esrom Berhane, Marc Fernandez-Vilana, Emilia Danczura, Cristina Puigjaner

**Affiliations:** † Department of Inorganic and Organic Chemistry, Section of Organic Chemistry, Institut de Biomedicina de la Universitat de Barcelona, 16724 Universitat de Barcelona, Carrer Martí i Franqués 1, 08028 Barcelona, Catalonia, Spain; ‡ X-ray Diffraction Unit, CCiTUB, Universitat de Barcelona, Carrer Solé i Sabarís 1−3, 08028 Barcelona, Catalonia, Spain

## Abstract

We describe a direct and asymmetric aldol-like reaction between a wide range of *N*-acyl-1,3-oxazolidine-2-thiones and cobalt-protected propargylic acetals catalyzed by a chiral nickel­(II) complex leading to *syn* β-alkoxy derivatives. This overcomes longstanding limitations associated with acetals from aliphatic aldehydes, selectively providing *syn* aldol adducts in excellent yields as single stereoisomers (dr >97:3, er up to >99:1). Furthermore, the cobalt fragment enables downstream intramolecular Pauson-Khand cyclizations, granting rapid access to densely functionalized bicyclic architectures.

## Graphical abstract

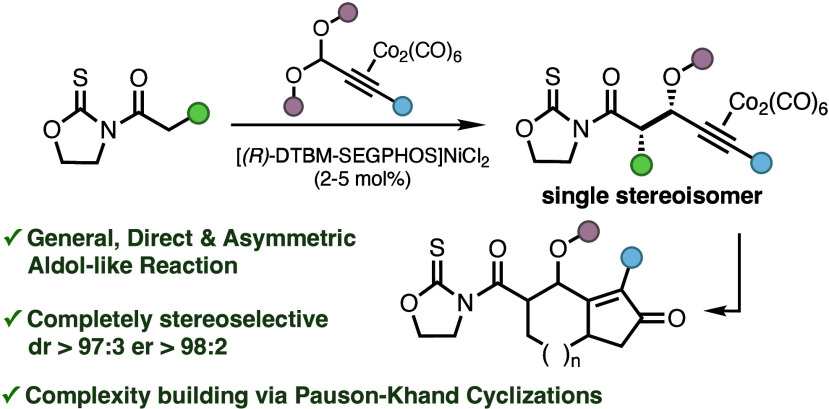

Despite the tremendous advances in asymmetric catalysis over recent decades, many reported methods still suffer from narrow substrate scopes and rely on structurally complex chiral catalysts, limiting their synthetic utility.^1^ Accordingly, the development of reliable and broad-scope methods for the stereocontrolled synthesis of carbon architectures, capable of meeting the growing demand for more efficient routes to biologically active compounds and novel chiral agrochemicals or materials, has attracted considerable interest.^2–5^

In this context, metal enolates stand out as privileged intermediates for the asymmetric construction of carbon–carbon bonds catalyzed by chiral metal complexes,^6,7^ with aldol reactions among the most prominent applications.^8,9^ Early approaches in this area relied on the stoichiometric generation of silyl enolates prior to the catalytic reaction, with clearly defined nucleophilic and electrophilic roles.^10^ By contrast, the seemingly simpler, and more convenient direct variants, in which a carbonyl adds to another in the presence of a chiral catalyst, remain a significant challenge. Against this background, pioneering studies by Shibasaki,^11^ Trost,^12^ and Evans^13^ demonstrated the feasibility of direct aldol reactions catalyzed by chiral metal complexes and laid the groundwork for further advances.^14^

Inspired by these precedents, we launched a few years ago a research project aimed at establishing a common strategy for the stereoselective formation of carbon–carbon bonds based on an S_N_1-like mechanism, an approach that hinges on electrophiles such as activated aldehydes or oxocarbenium ions.^15^ This strategy proved effective and the results met our expectations, as we were able to establish enantioselective syntheses of *protected* aldol adducts from thioimides in a single step using easy to handle chiral nickel­(II) complexes, a silyl triflate, and lutidine.^16,17^ Unfortunately, the generation of the nickel­(II) enolates was sometimes incompatible with the activation of the necessary electrophiles, such as aliphatic aldehydes or their acetal counterparts, thereby restricting the reacting species to those derived from aromatic systems (Scheme 1a). To overcome this limitation, we envisioned α,β-unsaturated aldehydes as suitable alternatives.^18^ However, controlling the preference toward the 1,2- over the 1,4-addition proved challenging, making the regioselectivity, together with the diastereo- and the enantioselectivity, a major hurdle in the overall transformation.^19,20^ We therefore reasoned that cobalt–protected propargylic aldehydes or acetals might be an appealing option because (i) the coordination of the triple bond to cobalt would suppress the 1,4-addition; (ii) the electrophile would be further activated through charge delocalization onto the cobalt complex;^21,22^ and (iii) the resulting complexes would serve as versatile platforms for building molecular complexity (Scheme 1b).

**Scheme 1:**
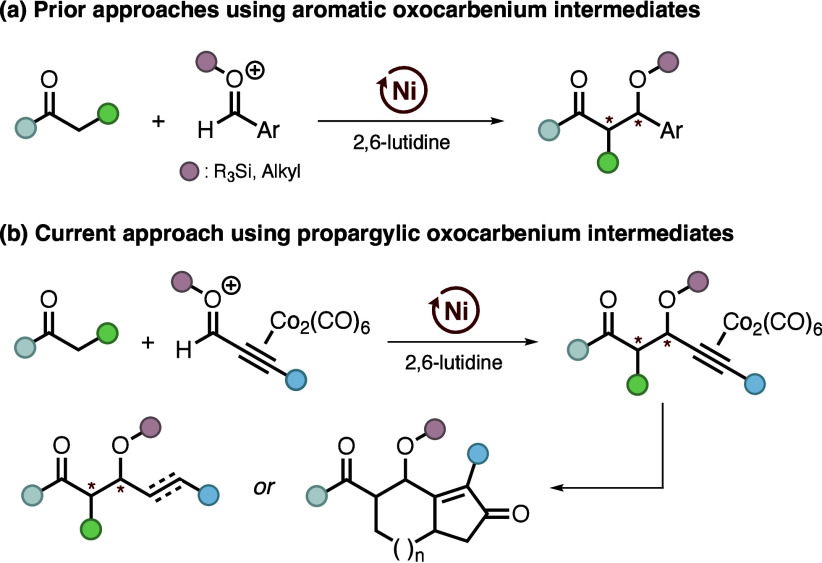
Direct, Asymmetric, and Catalytic Aldol Reactions

Herein, we report a highly stereocontrolled aldol-like addition of *N*-acyl-1,3-oxazolidine-2-thiones to dicobalt hexacarbonyl dialkyl propargylic acetals catalyzed by a chiral nickel­(II) complex to give protected *syn* aldol adducts.^23^ Subsequent reduction of the carbon–carbon triple bond provides access to formal aldol products of saturated and α,β-unsaturated acetals. Moreover, leveraging on the presence of the cobalt complex, the protected adducts undergo Pauson-Khand cyclizations to yield enantiomerically pure bicyclic architectures (Scheme 1b).^24^

With a well-established enolate platform consisting of thioimides and nickel­(II) catalysts bearing chiral diphosphine ligands,^15,25^ we began our investigations by evaluating the nature of the electrophile. While the protected phenyl propargyl aldehyde afforded complete regioselectivity toward the desired aldol adduct, it exhibited lower diastereoselectivity and yield compared to the protected analogue from the commercially available diethyl acetal **2a** (entries 1 and 2, Table 1). Consequently, cobalt-complexed propargylic acetals were identified as optimal electrophiles for this transformation. Then, following a comprehensive optimization of the experimental conditions, it was established that the TMSOTf–mediated aldol reaction of *N*-propanoyl-1,3-oxazolidine-2-thione (**1a**) and diethyl acetal **2a**, catalyzed by 2 mol % [(*R*)-DTBM-SEGPHOS]­NiCl_2_ gave quantitatively the protected *syn* aldol adduct **3a** as a single stereoisomer (dr >97:3 and er >99:1; Table 1).^25,26^ In contrast, the unprotected acetal failed to furnish the desired product (entry 3, Table 1). Likewise, low conversions were observed in the absence of the nickel catalyst (entry 4, Table 1). In turn, other thioimides, chiral phosphines, silyl triflates or solvents of varying polarity (entries 5–8, Table 1) proved less effective. Finally, the use of two equivalents of TMSOTf was essential to attain full conversion (entry 9, Table 1).

**Table 1:**
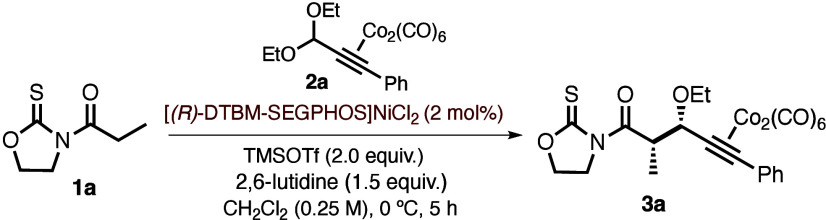
Optimization of the Reaction Conditions

Entry	Deviation from standard conditions	3a (%)^a^	dr^b^	er^c^
1	–	99	>97:3	>99:1
2	Aldehyde instead of acetal	26	78:22	nd
3	No Co protection	nr	nr	nr
4	No catalyst	8	>97:3	nd
5	[(*S*)-Tol-BINAP]NiCl_2_	68	>97:3	97:3
6	Other thioimides	37–76	>97:3	nd
7	Other solvents (DCM/Tol, EtOAc)	78–87	>97:3	nd
8	TESOTf instead of TMSOTf	52	>97:3	nd
9	TMSOTf (1.5–1.8 equiv)	81–91	>97:3	nd

a Isolated yields.

b Diastereomeric ratios were determined by ^1^H NMR analysis of the crude.

c Enantiomeric ratios were determined by chiral HPLC analysis.

With the optimized reaction conditions and the extremely promising results obtained for acetal **2a** in hand, we examined the direct TMSOTf–mediated aldol-like reaction of a variety of *N*-acyl-1,3-oxazolidine-2-thiones (**1a–g**) with a broad range of cobalt-protected dialkyl acetals (**2a–l**) catalyzed by [(*R*)-DTBM-SEGPHOS]­NiCl_2_. The results are summarized in Table 2.

**Table 2:**
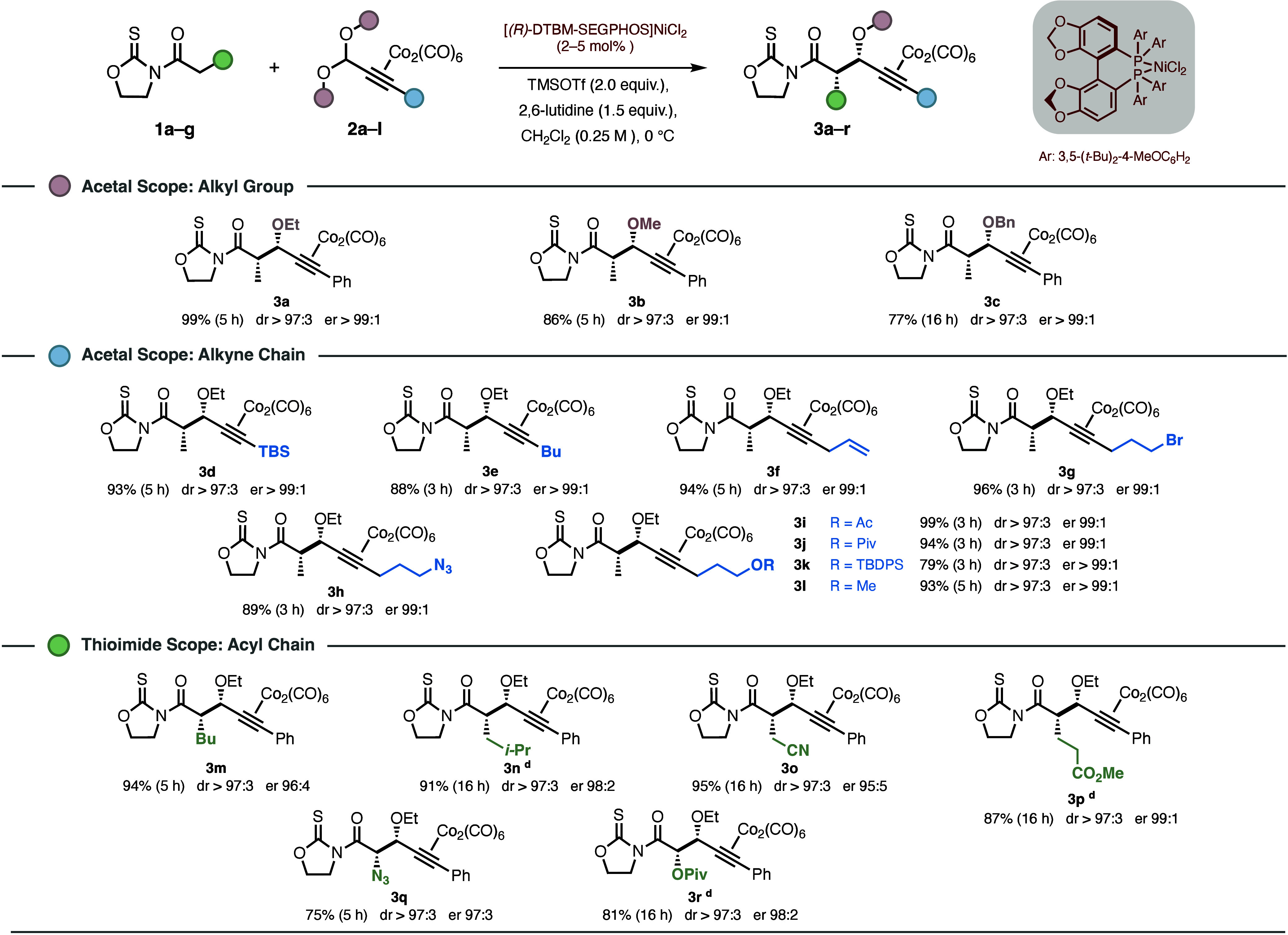
Scope of the Reaction^a^ ^,^ ^b^ ^,^ ^c^

a All reactions were performed on 0.25–0.5 mmol scale with 1.0 equiv of thioimides **1a–g** and 1.1 equiv. protected propargylic acetals **2a–l**.

b Diastereomeric ratios were determined by ^1^H NMR (400 MHz) analysis of the crude.

c Enantiomeric ratios were determined by chiral HPLC analysis.

d Reaction carried out with 5 mol % of the chiral nickel­(II) complex.

Acetals derived from protected phenylpropargylic aldehyde performed with similar efficiency. Indeed, both dimethyl and dibenzyl acetals, **2b** and **2c** respectively, provided the corresponding adducts **3b** and **3c** in high yields and with complete stereocontrol. Next, we investigated the effect of replacing the phenyl group in the diethyl acetal. While the terminal alkyne proved to be unsuitable, its silylated counterpart **2d** produced the aldol adduct **3d** as a single stereoisomer in high yield. Furthermore, propargylic acetals bearing either a simple alkyl group or more elaborate chains incorporating alkenes, halides, azides, or esters, consistently delivered the desired adducts **3e–j** in outstanding yields and with complete stereocontrol (dr >97:3, er 99:1). In contrast, free alcohols were poorly tolerated, although silyl ether or methyl derivatives **2k** and **2l** afforded the corresponding adducts **3k** and **3l** in stereochemically pure manner in high yields.

The reactivity of more elaborate thioimides **1a–g** bearing different acyl groups was also evaluated (Table 2). Longer and bulkier alkyl substituents, such as **1m** (*n*-Bu**)** and **1n** (*i*-Bu), were well tolerated, as were more functionalized alkyl chains containing nitrile (**1o**) or ester (**1p**) groups. The optimized conditions were likewise compatible with thioimides bearing heteroatoms at the α position, such as an azide (**1q**) or a protected alcohol (**1r**). Remarkably, all protected aldol adducts **3m–r** were obtained in excellent yields as single stereoisomers, although in some cases an increased catalytic loading of up to 5 mol % was needed to achieve full conversion.

To demonstrate the synthetic versatility of this new aldol-like reaction, the resultant adducts were subjected to a variety of transformations. We first targeted the decomplexation of the cobalt-protected adduct **3a** using CAN in acetone leading to thiazolidinethione **4a** (Scheme 2A). Under these conditions, a minor byproduct arising from oxidation of the heterocyclic thiocarbonyl bond (**5a** in Scheme 2A) was also detected.^27^ Alternative reagents, including Fe­(NO_3_)_3_·9H_2_O or NMO, led to the formation of the oxazolidinone **5a** too. Eventually, careful reaction monitoring under the initial conditions (CAN in acetone) minimized the formation of the oxazolidinone derivative without altering the stereochemical integrity of either the aldol adducts or the resulting oxazolidinone derivatives.^25^ Although these conditions proved acceptable, we sought to completely prevent the oxidation pathway. Since the heterocycle is ultimately converted into other carboxylic derivatives, we envisaged that its removal prior to the decomplexation step could circumvent this undesired reaction. As a proof of concept, we designed a simple three–step sequence to synthesize methyl esters **6**, requiring only a single purification and affording the corresponding carboxylic derivatives in high yield with outstanding diastereo- and enantioselectivity (dr >97:3, er >99:1) across a range of substrates (Scheme 2A). Adduct **3a** could also be deprotected and the thioimide platform displaced with (*S)*–methylbenzylamine to afford crystalline amide **7a**. Its absolute configuration was unambiguously established by X-ray analysis, thereby confirming the stereochemical assignment of adduct **3a** (Scheme 2B). Furthermore, the triple bond of ester **6a** was either completely reduced to **8a** or selectively into the *(Z)*-alkene **9a** (Scheme 2B).^28^ These and the above-mentioned transformations highlight the synthetic potential of this method to access a broad array of enantiomerically pure aldol derivatives.

**Scheme 2:**
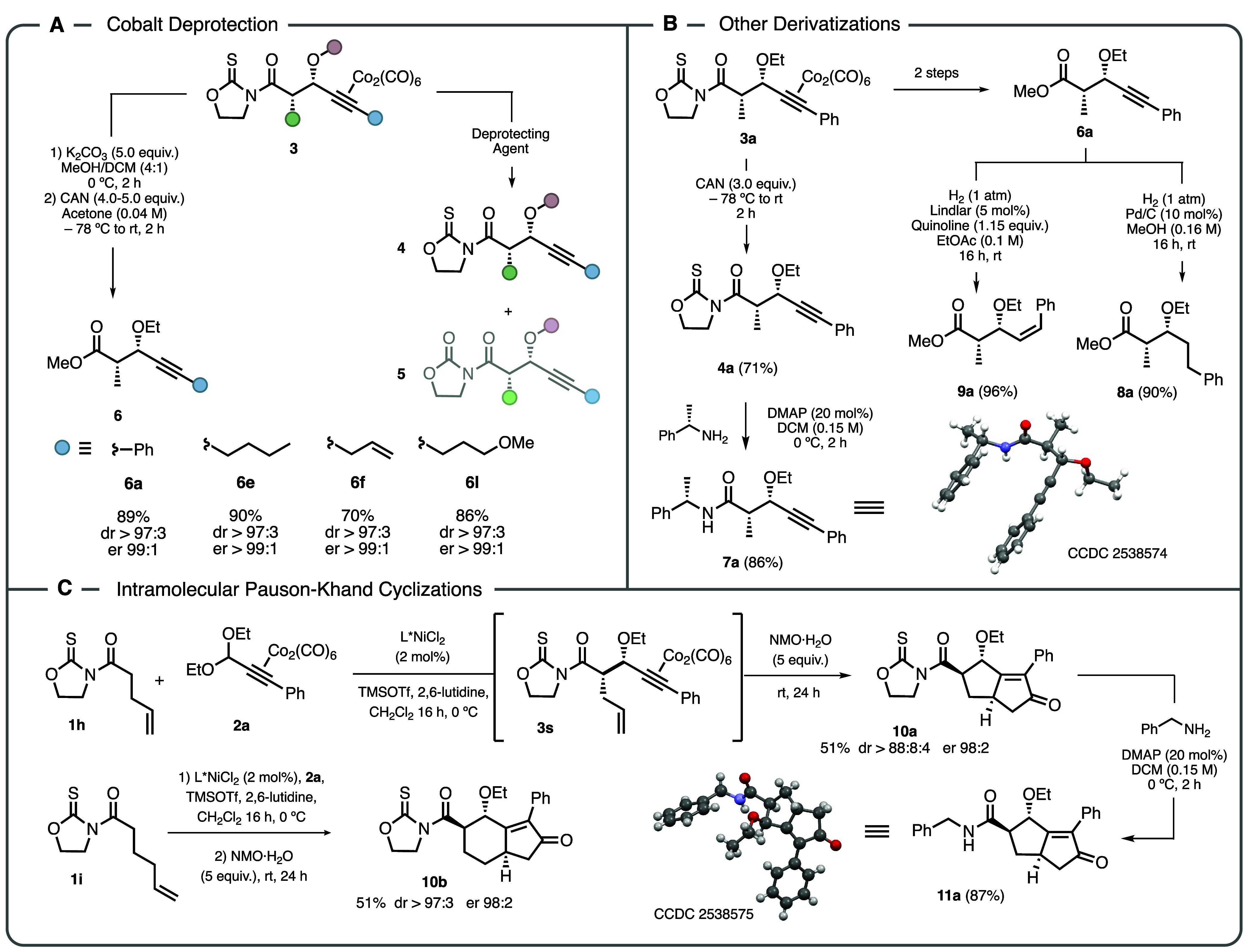
Transformations of the Aldol Adducts

Having established a direct, asymmetric, and broadly applicable aldol-like reaction, we envisioned that the cobalt complex could serve a dual purpose. Beyond its protective role, it might enable access to structurally intricate polycyclic frameworks through intramolecular Pauson–Khand cyclizations^29^ of substrates bearing a suitably positioned olefin. Such a demanding transformation would require careful control to ensure compatibility between the olefin and the cobalt-protected alkyne until the cyclization was triggered. Exploratory studies on allyl acetals as precursors to oxygenated heterocycles indicated that the intramolecular cyclization was problematic, likely owing to the tendency of the olefin and the cobalt-protected alkyne to react in previous steps of the synthetic sequence. Therefore, we turned our attention to thioimides bearing an olefin within the acyl group. Specifically, we assayed the construction of the tetrahydropentalen-2­(1*H*)-one bicyclic core starting from *N*-(4-pentenoyl)-1,3-oxazolidine-2-thione (**1h** in Scheme 2C). Under the optimized conditions (Table 1), the reaction of thioimide **1h** and diethyl acetal **2a** gave the expected aldol adduct quantitatively as a single diastereomer; however, it could not be isolated due to partial spontaneous cyclization on silica gel.^30^ Accordingly, the intramolecular Pauson-Khand cyclization was attempted directly on the crude reaction mixture. A range of conditions was thus screened. Initial trials led to the formation of numerous side products arising from oxidation of the exocyclic C = S bond, cobalt decomplexation, and/or simple degradation of the starting materials. Among the promoters tested, NMO·H_2_O emerged as the best candidate.^29^ Following this choice and after considerable experimentation, suitable conditions were identified under which a main diastereomer was observed in the crude mixture, though careful HPLC-MS analysis revealed two minor species with identical mass and fragmentation patterns in an approximate ratio of 88:8:4 based on chromatographic peak areas. Since no improvement was achieved with solvents other than dichloromethane, the same solvent used in the preceding aldol addition, a one-pot procedure was adopted. This protocol ultimately afforded bicycle **10a** in 51% isolated yield over two steps, with an enantiomeric ratio of 98:2. The absolute configuration was determined through conversion into crystalline amide **11a** and subsequent X-ray studies (Scheme 2C). Notably, this one-pot procedure seamlessly enabled the formation of four carbon–carbon bonds in a stereocon-trolled manner and with good overall yields. The optimized procedure was next applied to thioimide **1i** to access hexahydro-2*H*-inden-2-one analogue **10b** in good yield as a single stereoisomer (Scheme 2C).^31^

The high level of stereochemical control in the carbon–carbon bond forming step prompted mechanistic considerations. Based on the conversion of the nickel chloride complexes into the correspondent triflates described by Sodeoka^32^ and our prior studies on aldol-like reactions of thioimides and oxocarbenium ions,^15^ the catalytic cycle in Scheme 3 is proposed. Both the enolization step leading to chelated enolate **III**, or the formation of the oxocarbenium intermediate **IV** by reaction of TMSOTf with the dialkyl acetal,^15,33^ ultimately provide an enantiomeric ratio of up to 99:1 that likely originates from the chiral ligands of the nickel­(II) complex, which dictate the π facial selectivity of the nickel enolate through an open transition state as **V**. In contrast, the absolute diastereocontrol is attributed to the steric bulk of the cobalt-protected acetal moiety, which preferentially orients away from the thioimide scaffold. Accordingly, alkyne protection not only stabilizes the oxocarbenium cation but also enforces, through its considerable steric demand, the relative orientation of the substituents at the electrophilic carbon in the transition state, thereby accounting for the outstanding diastereoselectivities observed across the examples.

**Scheme 3:**
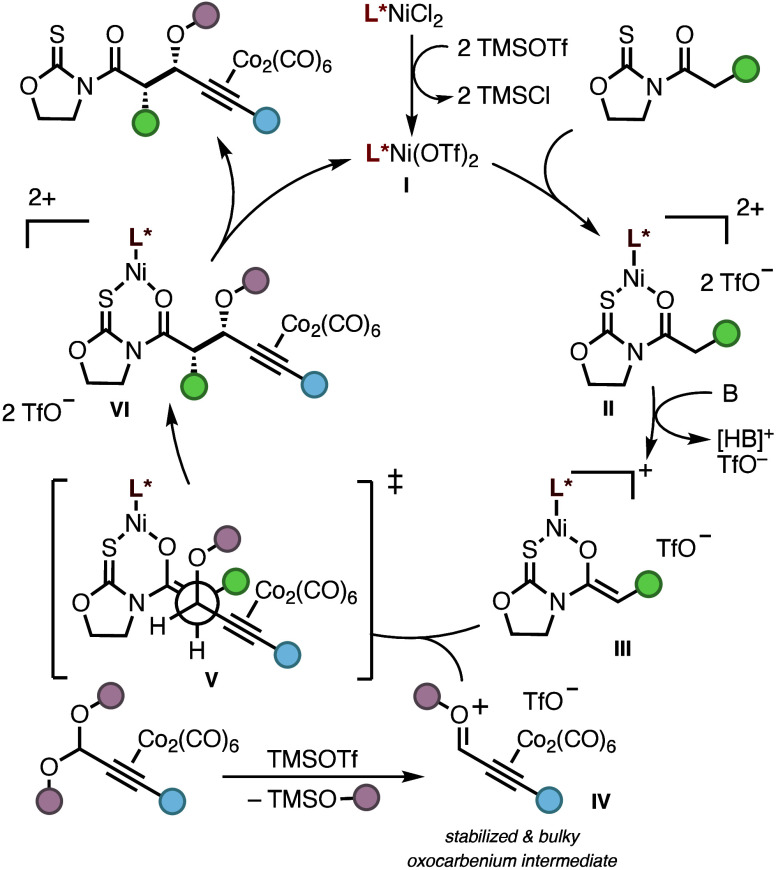
Proposed Mechanistic Cycle

In summary, we have developed a direct, asymmetric, and catalytic *syn* aldol-like reaction between a range of *N*-acyl oxazolidinethiones and cobalt-protected propargylic acetals, thereby overcoming the restrictions to aromatic electrophiles. This transformation furnishes protected *syn* aldol adducts as single stereoisomers in excellent yields with a broad substrate scope both for the groups neighboring the carbonyl and those at the distal propargylic position. In turn, these can be readily reduced to provide the formal aldol products of both saturated and α,β-unsaturated acetals. Moreover, by exploiting the reactivity of the cobalt complex, the reported methodology enables intramolecular Pauson-Khand cyclizations to install up to four new carbon–carbon bonds in a two-step sequence, affording structurally complex and densely functionalized bicyclic structures in a highly stereocontrolled manner.

## Supplementary Material



## Data Availability

The data underlying this study are available in the published article and its Supporting Information.

## Notes

Experimental procedures, characterization data of all new compounds, NMR spectra, and chromatograms (PDF)

## §

L. S.-C.: Enantia, S. L. Carrer Baldiri i Reixach 10. Barcelona, 08028, Spain

## ∥

M. F.-V.: Wanhua Chemical Europe Innovation, S. L. Carretera de l’Hospitalet 147. Edifici Atenes 1–1. Cornellà de Llobregat, 08940, Spain.

## ⊥

E. D.: Undergraduate student at Technological University Dublin, Ireland.
