# Supplementary material for: Cobalt-Stabilized Propargylic Oxocarbenium Ions Enable Direct and Asymmetric Nickel(II) Catalyzed Aldol-Like Reactions
Source: Org Lett. 2026 Jul 3;28(29):9312–7. doi: 10.1021/acs.orglett.6c02417 (PMC13411065; doi:10.1021/acs.orglett.6c02417)
Supplement: Supplementary file 1 [file ol6c02417_si_001.pdf]

Cobalt–Stabilized Propargylic Oxocarbenium Ions Enable Direct and Asymmetric Nickel(II) Catalyzed Aldol-Like Reactions

Xènia Tarrach, Leah O'Neill, Anna M. Costa,* Pedro Romea,* Fèlix Urpí,* Laura Sánchez-Castillo, Esrom Berhane, Marc Fernandez-Vilana, Emilia Danczura, and Cristina Puigjaner.

Table of Contents

1. General Experimental Methods 4

2. Synthesis of Starting Materials 6

2.1. Synthesis of heterocycles 6

2.2. Synthesis of thioimides 7

2.3. Synthesis of propargylic acetals 14

2.4. Synthesis of dicobalt hexacarbonyl-complexes of propargylic acetals 22

2.5. Synthesis of chiral catalysts 29

3. Optimization of the Asymmetric Aldol-type Reaction 30

3.1. Chiral catalyst screening 30

3.2. Heterocycle assessment 30

3.3. Evaluation of Lewis acids. 31

4. Catalytic and Asymmetric Aldol-like Reaction of Propargyl Acetals and N-acyl 1,3-oxazolidine-2-thiones. 33

5. Deprotection of Cobalted Adducts 49

6. Three-Step Methyl Ester Route 69

7. One-pot Pauson-Khand Reaction 75

7.1. Optimization of the intramolecular Pauson-Khand cyclization. 75

7.2. General Procedure H for the One-Pot Pauson-Khand Reaction. 76

8. Derivatizations 80

8.1. Reduction of the triple bond. 80

8.2. Removal of the heterocycle. 81

9. Crystal Information 84

9.1. Instrumentation 84

9.2. Sample Preparation and Crystal Measurements 84

10. References 88

11. NMR Spectra 89

12. HPLC/GC Chromatograms 377

1. General Experimental Methods

Unless otherwise noted, reactions were conducted in oven-dried (65 °C) glassware under inert atmosphere of N_2 with anhydrous solvents, all reagents and solvents were purchased from commercial suppliers and were used without further purification. Solvents such as DCM and THF were dried and purified according to standard procedures.

Melting points (Mp) were determined with a Stuart SMP10 apparatus or with a Cole-Parmer® MP-800D Series and are uncorrected.

Specific rotations ($[\alpha]_D$) were determined on a Perkin-Elmer 241 MC polarimeter equipped with a sodium lamp (λ 589 nm, D-line). Concentration (g/dL) and solvent used are indicated between brackets.

IR spectra (Attenuated Total Reflectance, ATR) were recorded in a Thermo Nicolet 6700 FT-IR spectrometer and in an Agilent Cary 630 FTIR. Only the more representative frequencies (ν) are reported in cm^{-1}.

Crude products were purified by column chromatography on Thermo Scientific silica gel 60 Å (35-70 µm) under low pressure (flash) conditions. Eluents are indicated in brackets in each case. R_f values are calculated approximately. Thin layer chromatography plates (Merck $60F_{254}$ silica gel) were visualized by exposure to UV light and/or stained with an aqueous solution of potassium permanganate or an ethanolic solution of phosphomolybdic acid. Cobalteted propargyl acetals were purified in the presence of a 5% of triethylamine. Propargyl acetals were found to be stable in silica gel.

^1H NMR and ^{13}C NMR spectra were recorded at r.t. on a Bruker Avance III HD 400 (400 MHz) with a CryoProbe™ Prodigy or a Bruker Avance Neo 500 (500 MHz) with a broadband iProb. Chemical shifts (δ) are reported in ppm and referenced to internal TMS (δ 0.00 for ^1H NMR) or $CDCl_3$ (δ 7.26 for ^1H NMR and 77.16 for ^{13}C NMR). Coupling constants (J) are quoted in Hertz (Hz) and multiplicity is reported as follows: s, singlet; d, doublet; t, triplet; q, quartet; p, quintet; m, multiplet (and their corresponding combinations). When necessary, 2D techniques (NOESY, COSY, HSQC) were also used to assist on structure elucidation.

HPLC analyses were conducted on a Shimadzu LC-20 HPLC under isocratic conditions and, unless specified, detected at 254 nm. The chiral column, mobile phase, and retention times are indicated for each case.

High resolution mass spectra (HRMS) were obtained with an Agilent 1100 spectrometer by the Unitat d’Espectrometria de Masses, Universitat de Barcelona.

2. Synthesis of Starting Materials

2.1. Synthesis of heterocycles

1,3-Oxazolidine-2-thione (A)

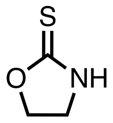

Anhydrous Et_3N (7.0 mL, 50 mmol, 1.0 equiv.) was added dropwise to a solution of 2-amino-1-etanol (3.0 mL, 50 mmol, 1.0 equiv.) in absolute methanol (50 mL). The mixture was then cooled to 0 °C, followed by the dropwise addition of CS_2 (4.5 mL, 75 mmol, 1.5 equiv.). After complete addition, the mixture was stirred at r.t. for 1 h. The resulting pale-yellow solution was cooled with an ice bath and then quenched with a slow addition of 33% (v/v) H_2O_2 until the precipitation of a white solid ceased (ca. 10 mL).

The yellow suspension was filtered and concentrated under reduced pressure using a peroxide tramp. Then, 2 M NaOH (25 mL, 0.5 mL/mmol) was added, and the solution was concentrated further to remove the leftover Et_3N. Afterwards, it was acidified with 2 M HCl until pH 1 (ca. 30 mL). The resulting bright yellow solution was extracted with DCM (4 × 30 mL), and the combined organic layers were dried over $MgSO_4$, filtered, and concentrated under reduced pressure. The solid obtained was purified by recrystallisation (Cyclohexane/DCM) to yield a white crystalline powder (3.04 g, 29 mmol, 59% yield).

White solid.
Mp 97–99 °C.
R_f 0.4 (Hex/EtOAc, 1:1).
IR (ATR) ν 3203, 2923, 1520, 1456, 1398, 1312, 1284, 1203, 1162, 1033 cm^{-1}.
^{1}H NMR (400 MHz, $CDCl_3$) δ 7.46 (br s, 1H, NH), 4.73 (t, J = 8.1 Hz, 2H, OCH_2), 3.82 (t, J = 8.1 Hz, 2H, NCH_2).
$^{13}C\{^1H\}$ NMR (101 MHz, $CDCl_3$) δ 190.1 (C), 70.4 (CH_2), 44.1 (CH_2).

All spectroscopic data are in agreement with literature.[1]

2.2. Synthesis of thioimides

General Procedure A for the acylation of heterocycles with acyl chlorides

An acyl chloride (1.2 equiv.) was added dropwise to a solution of the corresponding heterocycle (1.0 equiv.) and anhydrous Et_3N (1.3 equiv.) in DCM (0.5 M) at 0 °C. The reaction mixture was stirred at r.t.

The reaction mixture was quenched with a saturated solution of NH_4Cl (5 mL/10 mmol), rinsed with water (20 mL/10 mmol) and extracted with DCM (3 × 10 mL/ 10 mmol). The combined organic extracts were washed with 2 M NaOH (3 × 25 mL/ 10 mmol) and 2 M HCl (3 × 25 mL /10 mmol), dried with $MgSO_4$, filtered, and concentrated under reduced pressure. The resulting residue was purified by flash chromatography.

General Procedure B for the acylation of heterocycles with carboxylic acids

A carboxylic acid (1.1 equiv.) was added to a solution of the corresponding heterocycle (1.0 equiv.), EDC·HCl (1.2 equiv.) and DMAP (5 mol%) in DCM (0.3 M) at 0 °C. The reaction mixture was stirred at r.t.

Then, water (30 mL/10 mmol) was added, and the organic layer was extracted, washed with 2 M HCl (3 × 25 mL/ 10 mmol) and 2 M NaOH (3 × 25 mL/10 mmol). It was then dried with $MgSO_4$, filtered and concentrated under reduced pressure. When necessary, the resulting residue was purified by flash chromatography.

N-Propanoyl-1,3-oxazolidine-2-thione (1a)

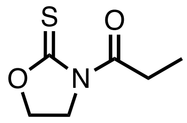

N-Propanoyl-1,3-oxazolidine-2-thione **1a** was synthesized according to General Procedure A from 1,3-oxazolidine-2-thione (**A**, 2.06 g, 20 mmol, 1.0 equiv.), anhydrous Et_3N (3.6 mL, 26 mmol, 1.3 equiv.), and propionyl chloride (2.2 mL, 24 mmol, 1.2 equiv.) in DCM (40 mL) for 16 h.

The resulting crude was purified by column chromatography (Hex/EtOAc, from 85:15 to 55:45) to afford 3.10 g (19.5 mmol, 97% yield) of the pure product.

White solid.
Mp 47–49 °C.
R_f 0.4 (Hex/EtOAc, 80:20).
IR (ATR) ν 2987, 2912, 1697, 1458, 1381, 1156, 930, 648 cm^{-1}.
^{1}H NMR ($CDCl_3$, 400 MHz) δ 4.55 (t, J = 8.6 Hz, 2H, OC$\underline{H}_2$), 4.24 (t, J = 8.6 Hz, 2H, NC$\underline{H}_2$), 3.31 (q, J = 7.3 Hz, 2H, COC$\underline{H}_2$$CH_3$), 1.20 (t, J = 7.3 Hz, $COCH_2C\underline{H}_3$).
^{13}C{^{1}H} NMR ($CDCl_3$, 101 MHz) δ 185.5 (C), 175.1 (C), 66.4 (CH_2), 47.1 (CH_2), 31.1 (CH_2), 8.5 (CH_3).

All spectroscopic data are in agreement with literature.[1]

N-Hexanoyl-1,3-oxazolidine-2-thione (1b)

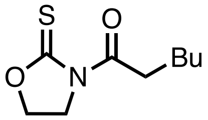

N-Hexanoyl-1,3-oxazolidine-2-thione **1b** was synthesized according to General Procedure B from 1,3-oxazolidine-2-thione (**A**, 516 mg, 5.00 mmol, 1.0 equiv.), caproic acid (0.70 mL, 5.50 mmol, 1.1 equiv.), EDC·HCl (1.2 g, 6.0 mmol, 1.2 equiv.) and DMAP (35 mg, 0.25 mmol, 5 mol%) in DCM (17 mL) for 16 h.

The crude was purified by column chromatography (Hex/EtOAc, 60:40) to afford 890 mg (4.40 mmol, 88% yield) of the pure product.

White solid with a low melting point (Mp < 27 °C).
R_f 0.4 (Hex/EtOAc, 60:40).
IR (ATR) ν 2954, 2928, 2861, 1698, 1402, 1372, 1323, 1183, 1146, 1016 cm^{-1}.
^{1}H NMR (400 MHz, $CDCl_3$) δ 4.58 – 4.49 (m, 2H, OC$\underline{H}_2$), 4.22 (t, J = 8.5 Hz, 2H, NC$\underline{H}_2$), 3.30 (t, J = 7.5 Hz, 2H, COC$\underline{H}_2$$CH_2$), 1.79 – 1.61 (m, 2H, $COCH_2C\underline{H}_2$), 1.43 – 1.23 (m, 4H, C$\underline{H}_2$$CH_2CH_3$, $CH_2C\underline{H}_2CH_3$), 0.99 – 0.81 (m, 3H, $CH_2C\underline{H}_3$).
^{13}C{^{1}H} NMR (101 MHz, $CDCl_3$) δ 185.7 (C), 174.7 (C), 66.5 (CH_2), 47.2 (CH_2), 37.3 (CH_2), 31.3 (CH_2), 24.3 (CH_2), 22.6 (CH_2), 14.1 (CH_3).

N-(4-Methylpentanoyl)-1,3-oxazolidine-2-thione (1c)

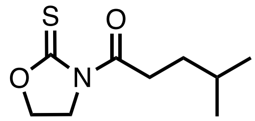

N-(4-Methylpentanoyl)-1,3-oxazolidine-2-thione **1c** was synthesized according to General Procedure B from 1,3-oxazolidine-2-thione (**A**, 1.03 g, 10 mmol, 1.0 equiv.), 4-methylpentanoic acid (1.4 mL, 11 mmol, 1.1 equiv.), EDC·HCl (2.40 mg, 12 mmol, 1.2 equiv.) and DMAP (61 mg, 0.5 mmol, 5 mol%) in a 16 h reaction in DCM (30 mL).

The crude was purified by column chromatography (Hex/EtOAc, from 100:0 to 70:30) to afford 1.72 g (8.6 mmol, 86% yield) of the pure product.

White solid with a low melting point (Mp < 27 °C).
R_f 0.3 (Hex/EtOAc, 80:20)
IR (ATR) ν 2954, 2926, 1868, 1696, 1402, 1370, 1318, 1189, 1156 cm^{-1}.
^{1}H NMR (400 MHz, $CDCl_3$) δ 4.56 – 4.47 (m, 2H, OC$\underline{H}_2$), 4.24 – 4.15 (m, 2H, NC$\underline{H}_2$), 3.31 – 3.25 (m, 2H, COC$\underline{H}_2$), 1.70 – 1.51 (m, 3H, C$\underline{H}_2$CH, CH_2C$\underline{H}$), 0.91 (d, J = 6.4 Hz, 6H, C(C$\underline{H}_3$)$_2$).
^{13}C{^{1}H} NMR (101 MHz, $CDCl_3$) δ 185.6 (C), 174.8 (C), 66.4 (CH_2), 47.2 (CH_2), 35.4 (CH_2), 33.3 (CH_2), 27.7 (CH), 22.4 (CH_3).

N-(3-Cyanopropanoyl)-1,3-oxazolidine-2-thione (1d)

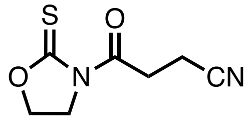

N-(3-Cyanopropanoyl)-1,3-oxazolidine-2-thione **1d** was synthesized according to General Procedure B from 1,3-oxazolidine-2-thione (**A**, 363 mg, 3.5 mmol, 1.0 equiv.), 3-cyanopropanoic acid (383 mg, 3.9 mmol, 1.1 equiv.), EDC·HCl (810 mg, 4.2 mmol, 1.2 equiv.) and DMAP (25 mg, 0.2 mmol, 5 mol%) in a 16 h reaction in DCM (12 mL).

The crude was purified by column chromatography (Hex/EtOAc, from 65:35 to 45:55) to afford 374 mg (2.0 mmol, 58% yield) of the pure product.

White solid.

Mp 122-125 °C.

R$_f$ 0.2 (Hex/EtOAc, 60:40).

IR (ATR) ν 2969, 2941, 2909, 2250, 1696, 1400, 1366, 1156 cm^{-1}.

^{1}H NMR (400 MHz, CDCl$_3$) δ 4.64 – 4.56 (m, 2H, OC<u>H</u>$_2$), 4.31 – 4.23 (m, 2H, NC<u>H</u>$_2$), 3.71 (t, J = 6.8 Hz, 2H, COC<u>H</u>$_2$), 2.73 (t, J = 6.8 Hz, 2H, C<u>H</u>$_2$CN).

^{13}C{^{1}H} NMR (101 MHz, CDCl$_3$) δ 185.4 (C), 170.8 (C), 118.7 (C), 67.0 (CH$_2$), 47.1 (CH$_2$), 34.2 (CH$_2$), 12.8 (CH$_2$).

N-(4-Methoxycarbonylbutanoyl)-1,3-oxazolidine-2-thione (1e)

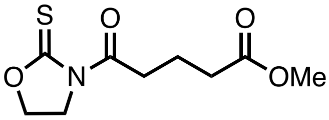

N-(4-Methoxycarbonylbutanoyl)-1,3-oxazolidine-2-thione **1e** was synthesized according to General Procedure A from 1,3-thiazolidine-2-thione (**A**, 1.03 g, 10.0 mmol, 1 equiv.), anhydrous Et$_3$N (1.4 mL, 13 mmol, 1.3 equiv.), and methyl 4-(chloroformyl)butyrate (1.4 mL, 12 mmol, 1.2 equiv.) in DCM (20 mL) for 16 h.

The crude was purified by column chromatography (Hex/EtOAc, from 100:0 to 80:20) to afford 1.776 g (7.7 mmol, 77% yield) of the pure product.

White solid

Mp 41-43 °C

R$_f$ 0.3 (Hex/EtOAc, 70:30)

IR (ATR) ν 2980, 2947, 2913, 1724, 1692, 1482, 1400, 1379, 1362, 1325, 1269 cm^{-1}.

^{1}H NMR (400 MHz, CDCl$_3$) δ 4.52 (t, J = 8.4 Hz, 2H, OC<u>H</u>$_2$), 4.20 (t, J = 8.4 Hz, 2H, NC<u>H</u>$_2$), 3.65 (s, 3H, OC<u>H</u>$_3$), 3.34 (t, J = 7.3 Hz, 2H, COC<u>H</u>$_2$), 2.40 (t, J = 7.3 Hz, 2H, COCH$_2$C<u>H</u>$_2$), 2.00 (p, J = 7.3, 2H, C<u>H</u>$_2$CO$_2$Me).

^{13}C{^{1}H} NMR (101 MHz, CDCl$_3$) δ 185.6 (C), 173.6 (C), 173.5 (C), 66.6 (CH$_2$), 51.7 (CH$_3$), 47.1 (CH$_2$), 36.5 (CH$_2$), 33.0 (CH$_2$), 19.7 (CH$_2$).

N-(α-Azidoacetyl)-1,3-oxazolidine-2-thione (1f)

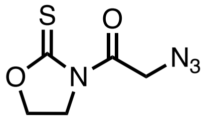

Neat 2-bromoacetic acid (13.00 g, 94 mmol, 1.0 equiv.) in 70 mL of distilled H_2O was added dropwise to an aqueous solution (60 mL) of sodium azide (12.00 g, 185 mmol, 2.0 equiv.) at 0 °C. After 15 min, the ice bath was removed, and the reaction mixture was stirred overnight at r.t. The reaction was then carefully acidified with 2 M HCl until pH 1 and the aqueous solution was extracted with Et_2O (3 × 50 mL). The combined organic extracts were dried over Na_2SO_4, filtered, and the solvent was removed under reduced pressure to afford 2-azidoacetic acid (9.40 g, 93 mmol, 99% yield) as a colorless oil which was used in the next step without further purification.

N-(α-Azidoacetyl)-1,3-oxazolidine-2-thione **1f** was synthesized according to General Procedure B from 1,3-oxazolidine-2-thione (**A**, 3.10 g, 30 mmol, 1.0 equiv.), α-azidoacetic acid (3.34 g, 33 mmol, 1.1 equiv.), EDC·HCl (7.48 g, 39 mmol, 1.2 equiv.) and DMAP (205 mg, 1.5 mmol, 5 mol%) in DCM (28 mL) for 16 h.

The crude was purified by column chromatography (Hex/EtOAc, 85:15) to afford 3.75 g (20 mmol, 67% yield) of the pure product.

White solid.
Mp 32–34 °C.
R_f 0.30 (Hex/EtOAc, 85:15)
IR (ATR) ν 2917, 2100, 1697, 1364, 1320, 1212, 1168, 1017 cm^{-1}.
^{1}H NMR ($CDCl_3$, 400 MHz) δ 4.91 (s, 2H, COC<u>H</u>$_2$N$_3$), 4.65 (t, J = 8.5 Hz, 2H, OC<u>H</u>$_2$), 4.29 (t, J = 8.5 Hz, 2H, NC<u>H</u>$_2$).
^{13}C{^{1}H} NMR (101 MHz, $CDCl_3$) δ 184.9 (C), 169.0 (C), 67.5 (CH_2), 54.5 (CH_2), 46.9 (CH_2).

All spectroscopic data are in agreement with literature.[2]

N-(α-Pivaloxyacetyl)-1,3-oxazolidine-2-thione (1g)

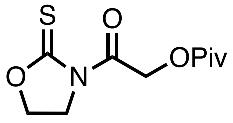

A mixture of pivaloyl chloride (2.2 mL, 18 mmol, 1.8 equiv.) and glycolic acid (761 mg, 10 mmol, 1.0 equiv.) was stirred for 48 h at r.t. Then, the volatiles were eliminated under vacuum and the product used in the next step without further purification.

N-(α-Pivaloyloxyacetyl)-1,3-oxazolidine-2-thione **1g** was synthesized according to General Procedure B from 1,3-oxazolidine-2-thione (**A**, 516 mg, 5.0 mmol, 1.0 equiv.), 2-(pivaloyloxy)acetic acid (1.04 g, 6.5 mmol, 1.3 equiv.), EDC·HCl (1.44 g, 7.5 mmol, 1.5 equiv.) and DMAP (34 mg, 0.25 mmol, 5 mol%) in DCM (17 mL) for 2 h.

The crude was purified by column chromatography (Hex/EtOAc, from 70:30 to 60:40) to afford 1.10 mg (4.5 mmol, 90% yield) of the pure product.

White solid.
Mp 56–58 ºC.
R_f 0.3 (Hex/EtOAc, 70:30).
IR (ATR) ν 2978, 2920, 2872, 1735, 1705, 1396, 1366, 1327, 1247, 1172, 1133 cm^{-1}.
^{1}H NMR ($CDCl_3$, 400 MHz) δ 5.51 (s, 2H, COC<u>H</u>$_2$OPiv), 4.63 (t, J = 8.5 Hz, 2H, OC<u>H</u>$_2$), 4.22 (t, J = 8.5 Hz, 2H, NC<u>H</u>$_2$), 1.27 (s, 9H, OCOC(C<u>H</u>$_3$)$_3$).
^{13}C{^{1}H} NMR (101 MHz, $CDCl_3$) δ 185.1 (C), 178.0 (C), 168.4 (C), 67.9 (CH_2), 64.6 (CH_2), 46.9 (CH_2), 38.9 (C), 27.2 (CH_3).

N-(4-Pentenoyl)-1,3-oxazolidine-2-thione (1h)

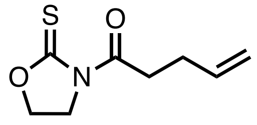

N-(4-Pentenoyl)-1,3-oxazolidine-2-thione **1h** was synthesized according to General Procedure B from 1,3-oxazolidine-2-thione (**A**, 1.0 g, 10.0 mmol, 1.0 equiv.), 4-pentenoic acid (1.1 mL, 11.0 mmol, 1.1 equiv.), EDC·HCl (2.3 g, 12.0 mmol, 1.2 equiv.) and DMAP (61 mg, 0.5 mmol, 5 mol%) in a 16 h reaction in DCM (30 mL).

The crude was purified by column chromatography (Hex/EtOAc, 80:20) to afford 1.602 g (8.6 mmol, 87% yield) of the pure product.

White solid with a low melting point (Mp < 27 °C).
R_f 0.4 (Hex/EtOAc, 80:20)
IR (ATR) ν 3066, 2978, 2950, 2911, 1698, 1642, 1467, 1444, 1377, 1361, 1327, 1262, 1193, 1157, 1018 cm^{-1}.
^{1}H NMR (400 MHz, $CDCl_3$) δ 5.88 (ddt, J = 17.0, 10.2, 6.4 Hz, 1H, C<u>H</u>=CH_2), 5.10 (dq, J = 17.0, 1.7 Hz, 1H, CH=C$\underline{H}_a H_b$), 5.03 (ddt, J = 10.2, 1.7, 1.3 Hz, 1H, CH=C$H_a \underline{H}_b$), 4.58 – 4.49 (m, 2H, OC$\underline{H}_2$), 4.27 – 4.18 (m, 2H, NC$\underline{H}_2$), 3.43 (t, J = 7.3 Hz, 2H, COC$\underline{H}_2$), 2.51 – 2.40 (m, 2H, C$\underline{H}_2$CH=CH_2).
$^{13}C\{^1H\}$ NMR (101 MHz, $CDCl_3$) δ 185.6 (C), 173.7 (C), 136.7 (CH), 115.8 (CH_2), 66.6 (CH_2), 47.1 (CH_2), 36.7 (CH_2), 28.4 (CH_2).

N-(5-Hexenoyl)-1,3-oxazolidine-2-thione (1i)

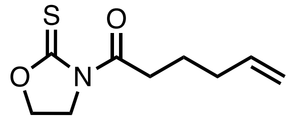

N-(5-Hexenoyl)-1,3-oxazolidine-2-thione **1i** was synthesized according to General Procedure B from 1,3-oxazolidine-2-thione (**A**, 516 mg, 5.0 mmol, 1.0 equiv.), 5-hexenoic acid (0.70 mL, 5.5 mmol, 1.1 equiv.), EDC·HCl (1.15 g, 6.0 mmol, 1.2 equiv.) and DMAP (35 mg, 0.25 mmol, 5 mol%) in DCM (16 mL) for 16 h.

The crude was purified by column chromatography (Hex/EtOAc, from 90:10 to 70:30) to afford 927 mg (4.65 mmol, 93% yield) of the pure product.

White solid.
Mp 38–41 °C.
R_f 0.4 (Hex/EtOAc, 80:20)
IR (ATR) ν 3075, 2974, 2913, 2859, 1694, 1638, 1472, 1400, 1370, 1320, 1247, 1187, 1154, 1016 cm^{-1}.
^{1}H NMR (400 MHz, $CDCl_3$) δ 5.80 (ddt, J = 17.1, 10.2, 6.7 Hz, 1H, C<u>H</u>=CH_2), 5.04 (ddt, J = 17.1, 2.1, 1.5 Hz, 1H, CH=C$\underline{H}_a H_b$), 4.99 (ddt, J = 10.2, 2.1, 1.2 Hz, 1H, CH=C$H_a \underline{H}_b$), 4.57 – 4.48 (m, 2H, OC$\underline{H}_2$), 4.26 – 4.17 (m, 2H, NC$\underline{H}_2$), 3.37 – 3.27 (m, 2H, COC$\underline{H}_2$), 2.20 – 2.09 (m, 2H, C$\underline{H}_2$CH=CH_2), 1.80 (tt, J = 8.0, 7.0 Hz, 2H, C$\underline{H}_2$$CH_2$CH=$CH_2$).

$^{13}C\{^1H\}$ NMR (101 MHz, $CDCl_3$) δ 185.6 (C), 174.4 (C), 137.9 (CH), 115.5 (CH_2), 66.5 (CH_2), 47.2 (CH_2), 36.7 (CH_2), 33.0 (CH_2), 23.7 (CH_2).

2.3. Synthesis of propargylic acetals

Phenylpropargylaldehyde dimethyl acetal (2B)

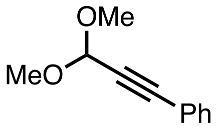

Trimethyl orthoformate (6.6 mL, 60 mmol, 4.0 equiv.), 3-phenylpropioaldehyde (1.8 mL, 15 mmol, 1.0 equiv.), alongside with Amberlyst-15® (5.7 g, 0.38 g/mmol), were dissolved in anhydrous MeOH (6.0 mL) and stirred at r.t. for 24 h.

Afterwards, the reaction mixture was filtered, washed with MeOH and concentrated in vacuo to afford 2.55 g (14.5 mmol, 97% yield) of 1,1-diethoxy-5-hydroxy-2-hexyne, which was used in the following step without further purification.

Colorless oil.
R_f 0.4 (Hex/EtOAc, 90:10).
^1H NMR (400 MHz, $CDCl_3$) δ 7.64 – 7.56 (m, 2H), 7.39 – 7.27 (m, 3H), 5.62 (s, 1H), 3.54 (s, 6H).

All spectroscopic data are in agreement with literature.[3]

Phenylpropargylaldehyde dibenzyl acetal (2C)

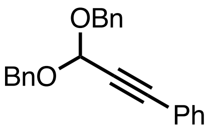

To a solution of CSA (174 mg, 0.75 mmol, 0.15 equiv.) and 4Å MS (6.0 g, 1.2 g/mmol) in DCM (6.0 mL), 3-phenylpropioaldehyde (0.6 mL, 5.0 mmol, 1.0 equiv.) and benzyl alcohol (4.0 mL, 40 mmol, 8.0 equiv.) were added and stirred at r.t. for 96 h.

After consumption of the aldehyde, the reaction mixture was washed with a saturated solution of $NaHCO_3$ (3 × 10 mL), the combined organic extracts were dried over $MgSO_4$, filtered and concentrated under reduced pressure. The resulting crude was purified via

column chromatography (Hex/DCM, from 70:30 to 30:70) to afford 1.01 g (3.1 mmol, 61% yield) of dibenzyl acetal **2C**.

White solid.
Mp 46–48 ºC.
R_f 0.5 (Hex/DCM, 1:1).
IR (ATR) ν 3088, 3060, 3032, 2909, 2878, 2373, 2227, 1653, 1444, 1359, 1333 cm^{-1}.
^{1}H NMR (400 MHz, $CDCl_3$) δ 7.50 – 7.43 (m, 2H, ArH), 7.43 – 7.26 (m, 13H, ArH), 5.69 (s, 1H, CH($OBn)_2$), 4.87 (d, J = 11.7 Hz, 2H, 2 × OCH_aH_bPh), 4.70 (d, J = 11.7 Hz, 2H, 2 × OCH_aH_bPh).
$^{13}C\{^1H\}$ NMR (101 MHz, $CDCl_3$) δ 137.6 (C), 132.1 (CH), 129.1 (C), 128.6 (CH), 128.4 (CH), 128.3 (CH), 127.9 (CH), 121.8 (C), 91.4 (CH), 86.2 (C), 84.2 (C), 67.6 (CH_2).

1,1-Diethoxy-3-(tert-butyldimethylsilyl)propyne (2D)

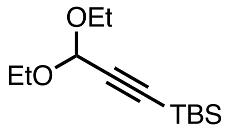

A 2.3 M solution of n-BuLi in hexanes (4.2 mL, 9.2 mmol, 1.15 equiv.) was added dropwise to a solution of propioaldehyde diethyl acetal (1.3 mL, 8.0 mmol, 1.0 equiv.) in THF (20 mL) at –78 °C and stirred for 15 min tert-Buyldimethylsilyl chloride (1.41 g, 9.6 mmol, 1.2 equiv.) was then carefully added and the mixture was stirred for 5 min at the same temperature. The resulting solution was then allowed to warm to r.t. and stirred overnight.

The mixture was then cooled with an ice-bath and quenched with water (6 mL) and extracted with Et_2O (3 × 20 mL). The combined organic extracts were washed with brine (25 mL), dried with $MgSO_4$, filtered, and concentrated under reduced pressure. The resulting crude was purified via column chromatography (Hex/Et_2O 95:5) to afford 1.42 g (5.9 mmol, 73% yield) of the title compound.

Colorless oil.
R_f 0.4 (Hex/Et_2O, 95:5, stained with $KMnO_4$).
IR (ATR) ν 2976, 2954, 2887, 2857, 1470, 1325, 1251, 1115, 1094, 1053, 1008 cm^{-1}.

^{1}H NMR (400 MHz, $CDCl_3$) δ 5.24 (s, 1H, $C\underline{H}(OEt)_2$), 3.74 (dq, J = 9.5, 6.9 Hz, 2H, 2 × $OC\underline{H}_aH_bCH_3$), 3.58 (dq, J = 9.5, 6.9 Hz, 2H, 2 × $OCH_a\underline{H}_bCH_3$), 1.22 (t, J = 6.9 Hz, 6H, $OCH_2C\underline{H}_3$), 0.94 (s, 9H, $SiC(C\underline{H}_3)_3$), 0.13 (s, 6H, 2 × $SiC\underline{H}_3$).
^{13}C{^{1}H} NMR (101 MHz, $CDCl_3$) δ 101.1 (C), 91.4 (CH), 88.7 (C), 61.0 (CH_2), 26.2 (CH_3), 16.6 (C), 15.2 (CH_3), – 4.6 (CH_3).

1,1-Diethoxy-2-heptyne (2E)

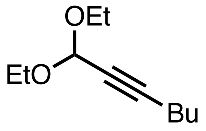

A 2.3 M solution of n-BuLi in hexanes (3.0 mL, 6.9 mmol, 1.13 equiv.) was added dropwise to a 0.1 M solution of propioaldehyde diethyl acetal (0.9 mL, 6.0 mmol, 1.0 equiv.) in THF (60 mL) at –78 °C. The reaction mixture was stirred for 30 min and 1-iodobutane (2.70 mL, 24 mmol, 4.0 equiv.) was carefully added. After 15 min, at –78 °C, the solution was allowed to warm to r.t. and left to react for two days.

The reaction mixture was cooled with an ice bath and quenched with H_2O (10 mL) and extracted with Et_2O (3 × 30 mL). The combined organic extracts were washed with water (30 mL) and brine (30 mL), dried with $MgSO_4$, filtered and concentrated in vacuo. The resulting crude was purified through flash chromatography (Hex/Et_2O, from 95:5 to 85:15) to afford 710 mg (3.65 mmol, 64% yield) of the named compound.

Colorless oil.
R_f 0.40 (Hex/Et_2O, 95:5, stained with $KMnO_4$).
IR (ATR) ν 2974, 2961, 2932, 2874, 1457, 1388, 1355, 1329, 1150, 1080, 1049 cm^{-1}.
^{1}H NMR (400 MHz, $CDCl_3$) δ 5.25 (t, J = 1.7 Hz, 1H, $C\underline{H}(OEt)_2$), 3.74 (dq, J = 9.5, 7.1 Hz, 2H, 2 × $OC\underline{H}_aH_bCH_3$), 3.57 (dq, J = 9.5, 7.1 Hz, 2H, 2 × $OCH_a\underline{H}_bCH_3$), 2.24 (td, J = 7.1, 1.7 Hz, 2H, $C{\equiv}CC\underline{H}_2$), 1.55 – 1.48 (m, 2H, $C{\equiv}CH_2C\underline{H}_2Et$) 1.46 – 1.36 (m, 2H, $CH_2C\underline{H}_2CH_3$), 1.23 (t, J = 7.1. Hz, 6H, 2 × $OCH_2C\underline{H}_3$), 0.94 – 0.85 (m, 3H, $CH_2CH_2CH_2C\underline{H}_3$).
^{13}C{^{1}H} NMR (101 MHz, $CDCl_3$) δ 91.6 (CH), 86.6 (C), 75.8 (C), 60.4 (CH_2), 30.5 (CH_2), 21.9 (CH_2), 18.4 (CH_2), 15.0 (CH_3), 13.6 (CH_3).

6,6-Diethoxy-1-hexen-4-yne (2F)

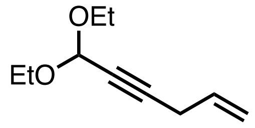

Propioaldehyde diethyl acetal (0.9 mL, 6.0 mmol, 1.0 equiv.) and allyl bromide (0.8 mL, 9.8 mmol, 1.1 equiv.) were added to a solution of K_2CO_3 (1.105 g, 8.0 mmol, 1.3 equiv.) and CuI (3 mg, 1 μmol, 0.2 mol%) in DMF (12 mL) at 30 °C and stirred overnight.

The reaction was then quenched with H_2O (10 mL) and extracted with Et_2O (3 × 20 mL). The combined organic extracts were washed with brine (25 mL), dried with $MgSO_4$ and evaporated under reduced pressure. The resulting crude was distilled in vacuo at 40 °C to afford 650 mg (3.9 mmol, 51% yield) of the title compound.

Colorless oil.
R_f 0.4 (Hex/Et_2O, 95:5, stained with $KMnO_4$).
IR (ATR) ν 3086, 2976, 2930, 2885, 2248, 1735, 1642, 1355, 1329, 1150, 1081, 1047, 1003 cm^{-1}.
^{1}H NMR (400 MHz, $CDCl_3$) δ 5.78 (ddt, J = 17.0, 10.1, 5.4 Hz, 1H, CH_2=C<u>H</u>), 5.31 (dq, J = 17.0, 1.8 Hz, 1H, C<u>H</u>$_a$H$_b$=CH), 5.27 (t, J = 1.7 Hz, 2H, C<u>H</u>(OEt)$_2$), 5.10 (dq, J = 10.1, 1.8 Hz, 1H, CH$_a$<u>H</u>$_b$=CH), 3.73 (dq, J = 9.5, 7.1 Hz, 2H, 2 × OC<u>H</u>$_a$H$_b$CH$_3$), 3.56 (dq, J = 9.5, 7.1 Hz, 2H, 2 × OCH$_a$<u>H</u>$_b$CH$_3$), 3.01 (dq, J = 5.4, 1.8 Hz, 2H, C≡CC<u>H</u>$_2$CH=CH$_2$), 1.21 (t, J = 7.1 Hz, 6H, 2 × OCH$_2$C<u>H</u>$_3$).
^{13}C{^{1}H} NMR (101 MHz, $CDCl_3$) δ 131.8 (CH), 116.6 (CH_2), 91.5 (CH), 82.9 (C), 78.1 (C), 60.8 (CH_2), 23.0 (CH_2), 15.2 (CH_3).

6-Bromo-1,1-diethoxy-2-hexyne (2G)

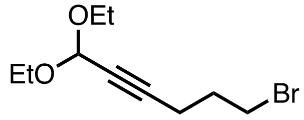

A 2.5 M solution of n-BuLi in hexanes (6.8 mL, 17.0 mmol, 1.13 equiv.) was added dropwise to a solution of propioaldehyde diethyl acetal (2.2 mL, 15.0 mmol, 1.00 equiv.) in THF (20 mL) at –78 °C. The resulting yellow solution was stirred for 30 min to then add 1,3-dibromopropane (1.9 mL, 18.15 mmol, 1.21 equiv.). After 15 min, the reaction mixture was allowed to warm to r.t. and then heated to 45 °C for 64 h.

The solution was then cooled with an ice-bath and quenched with water (10 mL) and extracted with DCM (3 × 20 mL). The combined organic extracts were washed with brine (30 mL), dried with $MgSO_4$, filtered, and concentrated under reduced pressure. The brown crude was purified by flash chromatography (Hex/EtOAc, from 100:0 to 98:2) with deactivated silica (5% Et_3N) to afford 2.81 g (11.3 mmol, 75% yield) of the product.

Colorless oil.

R_f 0.2 (Hex/Et_2O, 95:5, stained with $KMnO_4$).

IR (ATR) ν 2974, 2930, 2283, 2242, 1443, 1330, 1273, 1247, 1150, 1081, 1047 cm^{-1}.

^{1}H NMR (400 MHz, $CDCl_3$) δ 5.23 (t, J = 1.7 Hz, 1H, $C\underline{H}(OEt)_2$), 3.71 (dq, J = 9.5, 7.1 Hz, 2H, 2 × $OC\underline{H}_aH_bCH_3$), 3.55 (dq, J = 9.5, 7.1 Hz, 2H, 2 × $OCH_a\underline{H}_bCH_3$), 3.48 (t, J = 6.5 Hz, 2H, $CH_2C\underline{H}_2Br$), 2.43 (td, J = 6.8, 1.7 Hz, 2H, $C{\equiv}CC\underline{H}_2$), 2.10 – 1.99 (m, 2H, $CH_2C\underline{H}_2CH_2Br$), 1.21 (t, J = 7.1 Hz, 6H, 2 × $OCH_2C\underline{H}_3$).

$^{13}C\{^1H\}$ NMR (101 MHz, $CDCl_3$) δ 91.5 (CH), 84.2 (C), 77.0 (C), 60.8 (CH_2), 32.3 (CH_2), 31.2 (CH_2), 17.5 (CH_2), 15.2 (CH_3).

6-Azido-1,1-diethoxy-2-hexyne (2H)

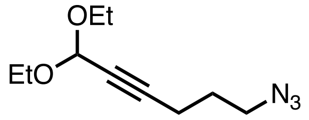

A solution of **2G** (1.74 g, 7.0 mmol, 1.0 equiv.) in DMF (3 mL) was added to a solution of NaN_3 (910 mg, 14 mmol, 2.0 equiv.) in DMF (4 mL) at 0 °C. The resulting clear solution was stirred for 15 min, and it was allowed to warm to r.t. and stirred for 16 h. The reaction mixture was extracted with Et_2O (3 × 10 mL). The combined organic extracts were washed with water (20 mL) and brine (20 mL), dried over $MgSO_4$, filtered and concentrated to afford 1.47 g of pure 6-azido-1,1-diethoxy-2-hexyne (6.9 mmol, 99% yield).

Colorless oil.

R_f 0.30 (Hex/Et_2O 90:10, stained with $KMnO_4$).

IR (ATR) ν 2976, 2932, 2881, 2240, 2093, 1444, 1329, 1254, 1146, 1081, 1047 cm^{-1}.

^{1}H NMR ($CDCl_3$, 400 MHz) δ 5.26 (t, J = 1.6 Hz, 1H, $C\underline{H}(OEt)_2$), 3.73 (dq, J = 9.5, 7.1 Hz, 2H, 2 × $OC\underline{H}_aH_bCH_3$), 3.57 (dq, J = 9.5, 7.1 Hz, 2H, 2 × $OCH_a\underline{H}_bCH_3$), 3.41 (t, J = 6.8 Hz,

2H, $CH_2C\underline{H}_2N_3$), 2.37 (td, J = 6.8, 1.6 Hz, 2H, $C{\equiv}CC\underline{H}_2$), 1.80 (p, J = 6.8 Hz, 2H, $CH_2C\underline{H}_2CH_2N_3$), 1.24 (t, J = 7.1 Hz, 6H, 2 × $OCH_2C\underline{H}_3$).

$^{13}C\{^1H\}$ NMR (101 MHz, CDCl₃) δ 91.5 (CH), 84.5 (C), 77.0 (C), 60.8 (CH_2), 50.3 (CH_2), 27.5 (CH_2), 16.1 (CH_2), 15.2 (CH_3).

6,6-Diethoxy-4-hexyn-1-yl acetate (2L)

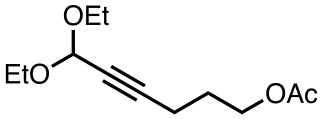

A solution of 6-bromo-1,1-diethoxy-2-hexyne (**2G**, 2.58 g, 10.4 mmol. 1.0 equiv.) in acetone (10 mL) was added via cannula to a solution of Bu_4NOAc (6.25 g, 20.7 mmol, 2.0 equiv.) in acetone (77 mL) and the resulting solution was stirred at 50 °C overnight.

The reaction mixture was then concentrated under vacuo and the resulting oil was dissolved in Et_2O (40 mL) and extracted with water (3 × 20 mL). The organic layer was then washed with a saturated solution of $NaHCO_3$ (30 mL) and brine (30 mL), dried over $MgSO_4$, filtered, and concentrated in vacuo. The resulting oil was purified through column chromatography (Hex/EtOAc 90:10) to afford 2.34 g (10.2 mmol, 99% yield) of the pure product.

Colorless oil.

R_f 0.3 (Hex/EtOAc, 90:10, stained with $KMnO_4$).

IR (ATR) ν 2974, 2932, 2883, 2246, 1739, 1366, 1329, 1232, 1148, 1046, 1003 cm^{-1}.

^1H NMR (400 MHz, CDCl₃) δ 5.21 (t, J = 1.7 Hz, 1H, $C\underline{H}(OEt)_2$), 4.11 (t, J = 6.3 Hz, 2H, $CH_2C\underline{H}_2OAc$), 3.69 (dq, J = 9.5, 7.1 Hz, 2H, 2 × $OC\underline{H}_aH_bCH_3$), 3.53 (dq, J = 9.5, 7.1 Hz, 2H, 2 × $OCH_a\underline{H}_bCH_3$), 2.32 (td, J = 7.2, 1.7 Hz, 2H, $C{\equiv}CC\underline{H}_2$), 2.01 (s, 3H, $OCOC\underline{H}_3$), 1.83 (tt, J = 7.2, 6.3 Hz, 2H, $CH_2C\underline{H}_2CH_2OAc$), 1.19 (t, J = 7.1 Hz, 6H, 2 × $OCH_2C\underline{H}_3$).

$^{13}C\{^1H\}$ NMR (101 MHz, CDCl₃) δ 171.0 (C), 91.4 (CH), 84.8 (C), 76.6 (C), 63.1 (CH_2), 60.7 (CH_2), 27.5 (CH_2), 21.0 (CH_3), 15.5 (CH_2), 15.1 (CH_3).

6,6-Diethoxy-4-hexyn-1-ol (2M)

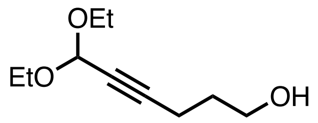

A solution of 6,6-diethoxy-4-hexyn-1-yl acetate (**2L**, 1.09 g, 4.8 mmol, 1.0 equiv.) in MeOH (10 mL) was added via cannula to a solution of NaOH (767 mg, 19.1 mmol, 4.0 equiv.) in MeOH (38 mL). The resulting clear solution was stirred at r.t. for 1 h.

Then, the reaction mixture was extracted with Et_2O (3 × 40 mL)and the combined organic extracts were washed with brine (30 mL), dried over $MgSO_4$, filtered, and concentrated in vacuo to afford 827 mg (4.4 mmol, 93% yield) of 6,6-diethoxy-4-hexyn-1-ol, which was used in the following step without further purification.

Colorless oil.
R_f 0.3 (Hex/EtOAc, 80:20, stained with $KMnO_4$).
^{1}H NMR (400 MHz, $CDCl_3$) δ 5.25 (t, J = 1.7 Hz, 1H, C<u>H</u>(OEt)$_2$), 3.78 – 3.69 (m, 4H, 2 × OC<u>H</u>$_a$H$_b$CH$_3$, CH$_2$C<u>H</u>$_2$OH), 3.57 (dq, J = 9.4, 7.1 Hz, 2H, 2 × OCH$_a$<u>H</u>$_b$CH$_3$), 2.38 (td, J = 7.1, 1.7 Hz, 2H, C≡CC<u>H</u>$_2$),1.79 (p, J = 7.1 Hz, 2H, CH$_2$C<u>H</u>$_2$CH$_2$OH), 1.23 (t, J = 7.1 Hz, 6H, 2 × OCH$_2$C<u>H</u>$_3$).

6,6-Diethoxy-4-hexynyl pivalate (2J)

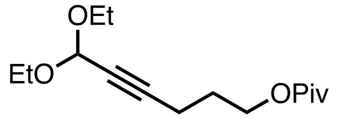

Pivaloyl chloride (490 µL, 4.0 mmol, 1.8 equiv.) and anhydrous Et_3N (780 µL, 5.6 mmol, 2.5 equiv.) were added dropwise to a solution of 6,6-diethoxy-4-hexyn-1-ol (**2M**, 448 mg, 2.2 mmol, 1.0 equiv.) in DCM (9 mL) at 0 °C. After 10 min, the reaction was allowed to warm up to r.t. and was stirred overnight.

The reaction mixture was extracted with water (10 mL) and the organic phase washed with brine (15 mL), dried over $MgSO_4$, filtered, and concentrated in vacuo. The resulting crude was purified via column chromatography (DCM) to afford 452 mg (1.7 mmol, 75% yield) of diethyl acetal **2J**.

Colorless oil.

R_f 0.3 (DCM, stained with $KMnO_4$).

IR (ATR) ν 2974, 2932, 2883, 1728, 1480, 1329, 1282, 1146, 1081, 1049, 1005 cm^{-1}.

^{1}H NMR (400 MHz, $CDCl_3$) δ 5.25 (t, J = 1.6 Hz, 1H, $C\underline{H}(OEt)_2$), 4.13 (t, J = 6.3 Hz, 2H, $C\underline{H}_2OPiv$), 3.73 (dq, J = 9.4, 7.1 Hz, 2H, 2 × $OC\underline{H}_aH_bCH_3$), 3.57 (dq, J = 9.4, 7.1 Hz, 2H, 2 × $OCH_a\underline{H}_bCH_3$), 2.35 (td, J = 7.3, 1.6 Hz, 2H, $C{\equiv}CC\underline{H}_2$), 1.87 (tt, J = 7.3, 6.3 Hz, 2H, $CH_2C\underline{H}_2CH_2OPiv$), 1.23 (t, J = 7.1 Hz, 6H, 2 × $OCH_2C\underline{H}_3$), 1.19 (s, 9H, $SiC(C\underline{H}_3)_3$).

$^{13}C\{^1H\}$ NMR (101 MHz, $CDCl_3$) δ 178.5 (C), 91.5 (CH), 84.9 (C), 76.5 (C), 63.0 (CH_2), 60.7 (CH_2), 38.9 (C), 27.6 (CH_2), 27.3 (CH_3), 15.5 (CH_2), 15.2 (CH_3).

6-tert-Butyldiphenylsilyloxy-1,1-diethoxy-2-hexyne (2K)

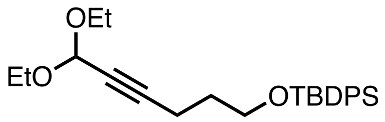

Neat TBDPSCl (1.1 mL, 4.0 mmol, 1.3 equiv.) was added dropwise to a solution of **2M** (577 mg, 3.1 mmol, 1.0 equiv.) and imidazole (274 mg, 4.0 mmol, 1.3 equiv.) in DCM (17 mL) for 10 min at 0 °C. The resulting solution was stirred for 16 h at r.t.

The reaction mixture was diluted with H_2O (20 mL) and extracted with DCM (3 × 20 mL). The combined extracts were dried with $MgSO_4$, filtered and evaporated in vacuo. The crude was purified via column chromatography (DCM) to afford 1.30 g (3.0 mmol, 97% yield) of the title compound.

Colorless oil.

R_f 0.7 (DCM, stained with $KMnO_4$).

IR (ATR) ν 3070, 3049, 2930, 2857, 2242, 1590, 1428, 1148, 1105 cm^{-1}.

^{1}H NMR (500 MHz, $CDCl_3$) δ 7.66 – 7.64 (m, 4H, Ar$\underline{H}$), 7.44 – 7.36 (m, 6H, Ar$\underline{H}$), 5.23 (t, J = 1.6 Hz, 1H, $C\underline{H}(OEt)_2$), 3.74 – 3.68 (m, 4H, 2 × $OC\underline{H}_aH_bCH_3$, $CH_2C\underline{H}_2OTBDPS$), 3.55 (dq, J = 9.6, 7.2 Hz, 2H, 2 × $OCH_a\underline{H}_bCH_3$), 2.40 (td, J = 7.4, 1.6 Hz, 2H, $C{\equiv}CC\underline{H}_2$), 1.81 – 1.75 (m, 2H, $CH_2C\underline{H}_2CH_2OTBDPS$), 1.23 (t, J = 7.2 Hz, 6H, 2 × $OCH_2C\underline{H}_3$), 1.04 (s, 9H, $SiC(C\underline{H}_3)_3$).

$^{13}C\{^1H\}$ NMR (101 MHz, $CDCl_3$) δ 135.7 (CH), 133.9 (C), 129.7 (CH), 127.8 (CH), 91.6 (CH), 86.2 (C), 76.0 (C), 62.6 (CH_2), 60.7 (CH_2), 31.4 (CH_2), 27.0 (CH_3), 19.4 (C), 15.4 (CH_2), 15.2 (CH_3).

All spectroscopic data are in agreement with literature.[4]

1,1-Diethoxy-6-methoxy-2-hexyne (2L)

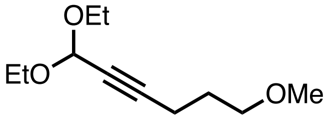

A solution of 1,1-diethoxy-5-hydroxy-2-hexyne (**2M**, 449 mg, 2.4 mmol, 1.0 equiv.) in dry THF (1.3 mL) was added via cannula to a suspension of NaH (60 % oil, 194 mg, 4.8 mmol, 2.0 equiv.) in dry THF (1.2 mL) and was stirred for 1 h at r.t. Then, methyl iodide (0.59 mL, 9.7 mmol, 4.0 equiv.) was added dropwise and the reaction mixture was stirred overnight.

The reaction was quenched with water (4 mL) and extracted with Et_2O (10 mL). The organic phase was washed with water (10 mL) and brine (10 mL), dried over $MgSO_4$, filtered, and concentrated in vacuo. The diethyl acetal **2L** was obtained quantitatively (>99% yield) and was used without further purification.

Colorless oil.
R_f 0.1 (DCM).
IR (ATR) ν 2974, 2924, 2874, 2242, 1444, 1355, 1329, 1150, 1120, 1050, 1003 cm^{-1}.
^{1}H NMR (400 MHz, $CDCl_3$) δ 5.23 (t, J = 1.7 Hz, 1H, C<u>H</u>$(OEt)_2$), 3.72 (dq, J = 9.5, 7.1 Hz, 2H, 2 × OC<u>H</u>$_a$H$_b$CH$_3$), 3.55 (dq, J = 9.5, 7.1 Hz, 2H, 2 × OCH$_a$<u>H</u>$_b$CH$_3$), 3.43 (t, J = 6.2 Hz, 2H, CH$_2$C<u>H</u>$_2$OMe), 3.31 (s, 3H), 2.32 (td, J = 7.2, 1.7 Hz, 2H, C≡CC<u>H</u>$_2$), 1.77 (tt, J = 7.2, 6.2 Hz, 2H, CH$_2$C<u>H</u>$_2$CH$_2$OMe), 1.21 (t, J = 7.1 Hz, 6H, 2 × OCH$_2$C<u>H</u>$_3$).
$^{13}C\{^1H\}$ NMR (101 MHz, $CDCl_3$) δ 91.6 (CH), 85.8 (C), 76.1 (C), 71.3 (CH_2), 60.7 (CH_2), 58.7 (CH_3), 28.5 (CH_2), 15.5 (CH_2), 15.2 (CH_3).

2.4. Synthesis of dicobalt hexacarbonyl-complexes of propargylic acetals

General Procedure C for the cobaltation of propargylic acetals

The corresponding propargyl acetal (1.0 equiv.) was added to a 0.2 M solution of $Co_2(CO)_8$ (1.05 equiv.) in DCM at r.t. and stirred for 3 h.

The reaction mixture was then concentrated under reduced pressure and the resulting crude was purified by column chromatography to yield the named compound.

Hexacarbonyl μ-[η^4-(1,1-diethoxy-3-phenylpropyne)dicobalt] (2a)

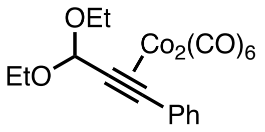

According to General Procedure C, the title compound was obtained from phenyl-propioaldehyde diethyl acetal (2.1 mL, 10 mmol, 1.0 equiv.).

The crude was purified by column chromatography (Hex/Et_3N, 95:5) to afford 4.80 g (9.8 mmol, 98% yield) of the pure product.

Dark solid with a low melting point (Mp < 27 °C).
$\mathbf{R_f}$ 0.8 (Hex/Et_3N, 95:5).
^{1}H NMR (400 MHz, $CDCl_3$) δ 7.61 (d, J = 7.9 Hz, 2H), 7.37 – 7.29 (m, 3H), 5.73 (s, 1H), 3.89 – 3.78 (m, 2H), 3.78 – 3.67 (m, 2H), 1.26 (t, J = 7.0 Hz, 6H).

Hexacarbonyl μ-[η^4-(1,1-dimethoxy-3-phenylpropyne)dicobalt] (2b)

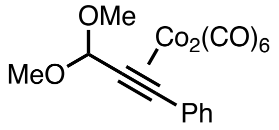

According to General Procedure C, the title compound was obtained from phenylpropargylaldehyde dimethyl acetal (**2B**, 880 mg, 5.0 mmol, 1.0 equiv.).

The crude was purified by column chromatography (Hex/Et_3N, 95:5) to afford 2.17 g (4.5 mmol, 91% yield) of the pure product.

Dark solid.
Mp 80–81 °C.
$\mathbf{R_f}$ 0.7 (Hex/Et_3N, 95:5).
^{1}H NMR (400 MHz, $CDCl_3$) δ 7.64 – 7.58 (m, 2H), 7.37 – 7.28 (m, 3H), 5.63 (s, 1H), 3.54 (s, 6H).

Hexacarbonyl μ-[η[4]-(1,1-dibenzyloxy-3-phenylpropyne)dicobalt] (2c)

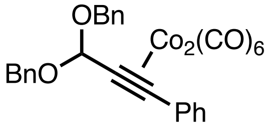

According to General Procedure C, the title compound was obtained from phenylpropargylaldehyde dibenzyl acetal (**2CFa**, 465 mg, 2.3 mmol, 1.0 equiv.).

The crude was purified by column chromatography (Hex/Et_3N, 95:5) to afford 1.11 g (2.2 mmol, 95% yield) of the pure product.
Dark oil.
R_f 0.7 (Hex/Et_3N, 95:5).
^{1}H NMR (400 MHz, $CDCl_3$) δ 7.61 – 7.52 (m, 2H), 7.40 – 7.27 (m, 13H), 5.96 (s, 1H), 4.78 – 4.71 (m, 4H).

Hexacarbonyl μ-[η[4]-(1,1-diethoxy-3-(tert-butildimethylsilane)propyne)dicobalt] (2d)

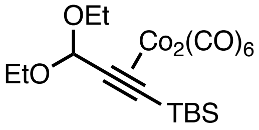

According to General Procedure C, the title compound was obtained from 1,1-diethoxy-3-(tert-butyldimethylsilyl)propyne (**2D**, 1.105 g, 4.6 mmol, 1.0 equiv.).

The crude was purified by column chromatography (Hex/Et_3N, 95:5) to afford 2.39 g (4.5 mmol, 99% yield) of the pure product.

Dark oil.
R_f 0.8 (Hex/Et_3N, 95:5).
^{1}H NMR (500 MHz, $CDCl_3$) δ 5.49 (s, 1H), 3.87 (dq, J = 9.7, 7.2 Hz, 2H), 3.64 (dq, J = 9.7, 7.2 Hz, 2H), 1.25 (t, J = 7.2. Hz, 6H), 1.01 (s, 9H), 0.25 (s, 6H).

Hexacarbonyl μ-[η⁴-(1,1-diethoxy-2-heptyne)dicobalt] (2e)

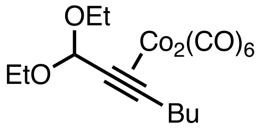

According to General Procedure C, the title compound was obtained from 1,1-diethoxy-2-heptyne (**2E**, 577 mg, 3.13 mmol, 1.0 equiv.).

The crude was purified by column chromatography (Hex/Et_3N, 95:5) to afford 1.40 g (3.0 mmol, 95% yield) of the pure product.
Dark oil.
R_f 0.9 (Hex/Et_3N, 95:5).
^{1}H NMR (400 MHz, $CDCl_3$) δ 5.48 (s, 1H), 3.80 (dq, J = 9.2, 7.0 Hz, 2H), 3.67 (dq, J = 9.2, 7.0 Hz, 2H), 2.82 (t, J = 9.0 Hz, 2H), 1.68 – 1.60 (m, 2H), 1.48 (h, J = 7.3 Hz, 2H), 1.25 (t, J = 7.0 Hz, 6H), 0.97 (t, J = 7.4 Hz, 3H).

Hexacarbonyl μ-[η⁴-(6,6-diethoxy-1-hexen-4-yne)dicobalt] (2f)

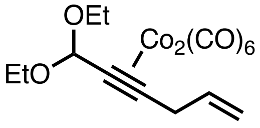

According to General Procedure C, the title compound was obtained from 6,6-diethoxy-1-hexen-4-yne (**2F**, 1.02 g, 6.0 mmol, 1.0 equiv.).

The crude was purified by column chromatography (Hex/Et_3N, 95:5) to afford 2.40 g (5.3 mmol, 88% yield) of the pure product.

Dark oil.
R_f 0.6 (Hex/Et_3N, 95:5).
^{1}H NMR (500 MHz, $CDCl_3$) δ 5.96 (ddt, J = 16.9, 9.9, 7.1 Hz, 1H), 5.49 (s, 1H), 5.24 – 5.17 (d, J = 16.9 Hz, 1H), 5.14 (d, J = 9.9 Hz, 1H), 3.81 (dq, J = 9.0, 7.0 Hz, 2H), 3.67 (dq, J = 9.0, 7.0 Hz, 2H), 3.57 (d, J = 7.1 Hz, 2H), 1.25 (t, J = 7.0 Hz, 6H).

Hexacarbonyl μ-[η[4]-(6-bromo-1,1-diethoxy-2-hexyne)dicobalt] (2g)

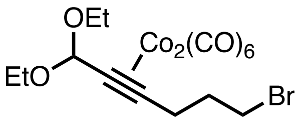

According to General Procedure C, the title compound was obtained from 6-bromo-1,1-diethoxy-2-hexyne (**2G**, 685 mg, 2.75 mmol, 1.0 equiv.).

The crude was purified by column chromatography (Hex/Et_3N, 95:5) to afford 1.46 g (2.7 mmol, 99% yield) of the pure product.

Dark oil.
R_f 0.8 (Hex/Et_3N, 95:5).
^{1}H NMR (400 MHz, $CDCl_3$) δ 5.49 (s, 1H), 3.81 (dq, J = 9.1, 7.0 Hz, 2H), 3.68 (dq, J = 9.1, 7.0 Hz, 2H), 3.57 (t, J = 6.4 Hz, 2H), 3.03 – 2.99 (m, 2H), 2.25-2.18 (m, 2H), 1.24 (t, J = 7.0. Hz, 6H).

Hexacarbonyl μ-[η[4]-(6-azido-1,1-diethoxy-2-hexyne)dicobalt] (2h)

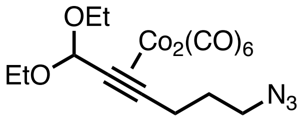

According to General Procedure C, the title compound was obtained from 5-azido-1,1-diethoxy-2-hexyne (**2H**, 1.46 g, 6.9 mmol, 1.0 equiv.).

The crude was purified by column chromatography (Hex/Et_3N, 95:5) to afford 3.27 g (6.6 mmol, 95% yield) of the pure product.

Dark oil.
R_f 0.7 (Hex/Et_3N, 95:5).
^{1}H NMR (500 MHz, $CDCl_3$) δ 5.49 (s, 1H), 3.81 (dq, J = 9.2, 7.0 Hz, 2H), 3.67 (dq, J = 9.2, 7.0 Hz, 2H), 3.47 (t, J = 6.5 Hz, 2H), 2.96 – 2.89 (m, 2H), 1.99 – 1.90 (m, 2H), 1.25 (t, J = 7.0 Hz, 6H).

Hexacarbonyl μ-[η4-(6-acetyloxy-1,1-diethoxy-2-hexyne)dicobalt] (2i)

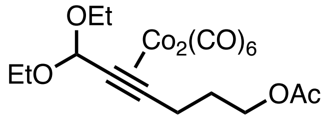

According to General Procedure C, the title compound was obtained from 6,6-diethoxy-4-hexyn-1-yl acetate (**2L**, 571 mg, 2.5 mmol, 1.0 equiv.).

The crude was purified by column chromatography (Hex/Et_3N, 95:5) to afford 1.29 g (2.5 mmol, 93% yield) of the pure product.

Dark oil.
R_f 0.5 (Hex/Et_3N, 95:5).
^{1}H NMR (400 MHz, $CDCl_3$) δ 5.49 (s, 1H), 4.21 (t, J = 6.3 Hz, 2H), 3.81 (dq, J = 9.1, 7.0 Hz, 2H), 3.67 (dq, J = 9.1, 7.0 Hz, 2H), 2.94 – 2.85 (m, 2H), 2.07 (s, 3H), 2.01 – 1.93 (m, 2H), 1.25 (t, J = 7.0 Hz, 6H).

Hexacarbonyl μ-[η4-(1,1-diethoxy-6-pivaloyloxy-2-hexyne)dicobalt] (2j)

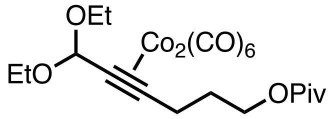

According to General Procedure C, the title compound was obtained from 6,6-diethoxy-2-hexyn-1-yl pivalate (**2J**, 452 mg, 1.67 mmol, 1.0 equiv.).

The crude was purified by column chromatography (Hex/Et_3N, 95:5) to afford 857 mg (1.54 mmol, 92% yield) of the pure product.

R_f 0.6 (Hex/Et_3N, 95:5).
^{1}H NMR (500 MHz, $CDCl_3$) δ 5.49 (s, 1H), 4.22 (t, J = 6.2 Hz, 2H), 3.81 (dq, J = 9.3, 7.0 Hz, 2H), 3.67 (dq, J = 9.3, 7.0 Hz, 2H), 2.92 – 2.88 (m, 2H), 2.03 – 1.96 (m, 2H), 1.25 (t, J = 7.0 Hz, 6H), 1.21 (s, 9H).

Hexacarbonyl μ-[η^4-(6-tert-butyldiphenylsilyloxy-1,1-diethoxy-2-hexyne)dicobalt] (2k)

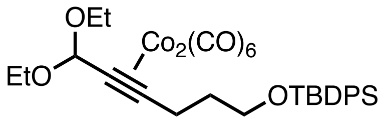

According to General Procedure C, the title compound was obtained from 6,6-diethoxy-4-hexyn-1-yl acetate (**2K**, 516 mg, 1.22 mmol, 1.0 equiv.).

The crude was purified by column chromatography (Hex/Et_3N, 95:5) to afford 835 mg (1.18 mmol, 96% yield) of the pure product.

Dark oil.
R_f 0.4 (Hex/Et_3N, 95:5).
^{1}H NMR (500 MHz, $CDCl_3$) δ 7.68 – 7.66 (m, 4H), 7.44 – 7.36 (m, 6H), 5.47 (s, 1H), 3.81 – 3.75 (m, 4H), 3.65 (dq, J = 9.2, 7.2 Hz, 2H), 2.95 – 2.92 (m, 2H), 1.91-1.86 (m, 2H), 1.23 (t, J = 7.2 Hz, 6H), 1.06 (s, 9H).

Hexacarbonyl μ-[η4-(1,1-diethoxy-6-methoxy-2-hexyne)dicobalt] (2l)

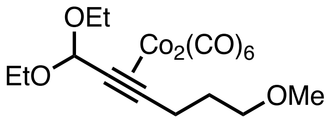

According to General Procedure C, the title compound was obtained from 1,1-diethoxy-6-methoxy-2-hexyne (**2L**, 465 mg, 2.3 mmol, 1.0 equiv.).

The crude was purified by column chromatography (Hex/Et_3N, 95:5) to afford 1.11 g (2.2 mmol, 95% yield) of the pure product.

Dark oil.
R_f 0.6 (Hex/Et_3N, 95:5).
^{1}H NMR (500 MHz, $CDCl_3$) δ 5.48 (s, 1H), 3.80 (dq, J = 9.1, 6.9 Hz, 2H), 3.67 (dq, J = 9.1, 6.9 Hz, 2H), 3.52 (t, J = 6.3 Hz, 2H), 3.37 (s, 3H), 2.95 – 2.87 (m, 2H), 1.99 – 1.87 (m, 2H), 1.25 (t, J = 6.9 Hz, 6H).

2.5. Synthesis of chiral catalysts

General Procedure D for the synthesis of chiral nickel(II) catalysts.

A solution of chiral diphosphine ligand (1.0 equiv.) and anhydrous $NiCl_2$ (1.0 equiv.) in acetonitrile (0.05 M) was heated to reflux overnight.

Afterwards, the mixture was filtered through Celite® and eluted with acetonitrile. The volatiles were removed under reduced pressure to afford the corresponding chiral Nickel compound which was used without further purification.

[(R)-DTBM-SEGPHOS]$NiCl_2$

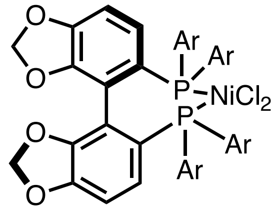

Ar = 3,5-*t*-Bu_2-4-$MeOC_6H_4$

According to General Procedure D, the title compound was obtained from $NiCl_2$ (131 mg, 1.0 mmol, 1.0 equiv.) and (R)-DTBM-SEGPHOS] (1.19 g, 1.0 mmol, 1.0 equiv.).

The resulting dark solid was used without further purification (1.290 g, 0.99 mmol, 99%).
Black solid.
Mp 249–251°C.
IR (film) 2956, 1438, 1410, 1393, 1227, 1178, 1138, 1116, 1052, 1006, 807, 639 cm^{-1}.

3. Optimization of the Asymmetric Aldol-type Reaction

3.1. Chiral catalyst screening.

A solution of an N-propanoyl-1,3-oxazinane-2-thione (**D**, 87 mg, 0.50 mmol, 1.0 equiv.) and hexacarbonyl μ-[η[4]-(1,1-diethoxy-3-phenylpropyne)dicobalt] (**2a**, 270 mg, 0.55 mmol, 1.1 equiv.), and the corresponding catalyst (2 mol%) in DCM (2 mL) was cooled to 0 °C. Then, TMSOTf (120 μL, 0.65 mmol, 1.3 equiv.) was added dropwise and stirred for 3 min. 2,6-Lutidine (90 μL, 0.75 mmol, 1.5 equiv.) was then added and the resultant mixture was stirred at 0 °C for 5 h.

The mixture was quenched with a saturated solution of NH_4Cl (2 mL) and extracted with DCM (3 × 5 mL). The combined organic extracts were dried over $MgSO_4$, filtered, and concentrated in vacuo. Finally, the crude was purified by column chromatography to yield the named compound as a single enantiomer. The syn/anti diasteromeric ratio (dr) was established in each case by ^1H NMR analysis of the crude mixture.

Table S1. Influence of the chiral catalyst.

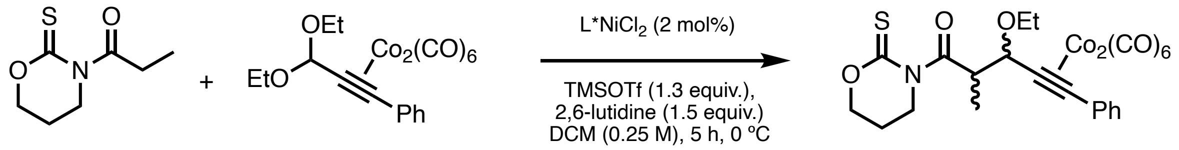

Entry	Catalyst	Conv.(%)[a]	Yield (%)[b]	d.r.[a]	e.r.[c]
1	[(R)-DTBM-SEGHPHOS]$NiCl_2$	>97	71	> 97:3	99:1
2	[(S)-Tol-BINAP]$NiCl_2$	>97	68	> 97:3	97:3

[a] Established by ^1H NMR analysis of the crude. [b] Isolated yields. [c] Determined by chiral HPLC analysis.

3.2. Heterocycle assessment.

A solution of a thioimide (0.50 mmol, 1.0 equiv.) and hexacarbonyl μ-[η[4]-(1,1-diethoxy-3-phenylpropyne)dicobalt] (**2a**, 270 mg, 0.55 mmol, 1.1 equiv.), and [(R)-DTBM-SEGPHOS]$NiCl_2$ (13.09, 0.01 mmol, 2 mol%) in DCM (2 mL) was cooled to 0 °C. Then, TMSOTf (120 μL, 0.65 mmol, 1.3 equiv.) was added dropwise and stirred for 3 min. 2,6-Lutidine (90 μL, 0.75 mmol, 1.5 equiv.) was then added and the resultant mixture was stirred at 0 °C .

The mixture was then quenched with a saturated solution of NH_4Cl (2 mL) and extracted with DCM (3 × 5 mL). The combined organic extracts were dried over $MgSO_4$, filtered, and concentrated in vacuo. Finally, the crude was purified by column chromatography to yield the named compound as a single enantiomer. The syn/anti diasteromeric ratio (dr) was established in each case by ^1H NMR analysis of the crude mixture.

Table S2. Effect of the heterocycle.

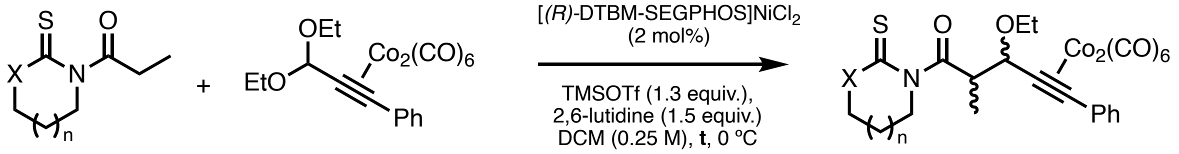

Entry	N-Acyl thioimide	Time (h)	Conv.(%)[a]	Yield (%)[b]	d.r.[a]	e.r.[c]
1	**C** (n = 1, X = O)	1	95	76	> 97:3	nd
2	**F** (n = 1, X = S)	1	69	37	> 97:3	nd
3	**E** (n = 0, X = S)	1	68	62	96:4	nd
4	**D** (n = 0, X = O)	1	75	74	> 97:3	>99:1
5	**D** (n = 0, X = O)	3	86	80	> 97:3	>99:1
6	**D** (n = 0, X = O)	16	92	92	> 97:3	>99:1

[a] Established by ^1H NMR analysis of the crude. [b] Isolated yields. [c] Determined by chiral HPLC analysis.

3.3. Evaluation of Lewis acids.

A solution of an N-propanoyl-1,3-oxazinane-2-thione (**D**, 87 mg, 0.50 mmol, 1.0 equiv.) and hexacarbonyl μ-[η^4-(1,1-diethoxy-3-phenylpropyne)dicobalt] (**2a**, 270 mg, 0.55 mmol, 1.1 equiv.), and [(R)-DTBM-SEGPHOS]$NiCl_2$ (13.09 mg, 10 μmol, 2 mol%) in DCM (2 mL) was cooled to 0 °C. Then, the corresponding Lewis acid (1.3–2.2 equiv.) was added dropwise and stirred for 3 min. 2,6-Lutidine (90 μL, 0.75 mmol, 1.5 equiv.) was then added and the resultant mixture was stirred at 0 °C for 5 h.

The mixture was quenched with a saturated solution of NH_4Cl (2 mL) and extracted with DCM (3 × 5 mL). The combined organic extracts were dried over $MgSO_4$, filtered, and

concentrated in vacuo. Finally, the crude was purified by column chromatography to yield the named compound as a single enantiomer. The syn/anti diasteromeric ratio (dr) was established in each case by ^{1}H NMR analysis of the crude mixture.

Table S3. Influence of the Lewis Acid.

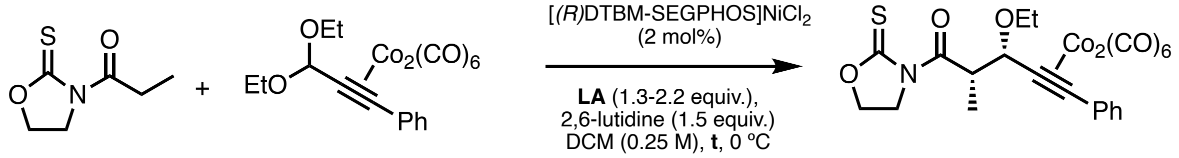

Entry	Lewis Acid	(equiv.)	Time (h)	Conv.(%)[a]	Yield (%)[b]	dr[a]	er[c]
1	TESOTf	1.3	1	52	n.d.	> 97:3	n.d.
2	TMSOTf	1.3	1	75	74	> 97:3	>99:1
3	TMSOTf	1.5	5	81	n.d.	> 97:3	nd
4	TMSOTf	1.8	5	91	n.d.	> 97:3	nd
5	TMSOTf	2.0	5	> 97	99	> 97:3	>99:1
6	TMSOTf	2.2	5	> 97	80	> 97:3	nd

[a] Established by ^{1}H NMR analysis of the crude. [b] Isolated yields. [c] Determined by chiral HPLC analysis.

4. Catalytic and Asymmetric Aldol-like Reaction of Propargyl Acetals and N-acyl 1,3-oxazolidine-2-thiones.

General Procedure E for the TMSOTf-mediated reactions catalyzed by [(R)-DTBM-SEGPHOS]NiCl$_2$.

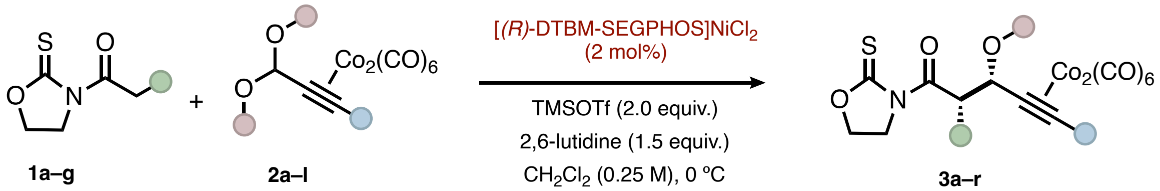

A solution of an N-acyl 1,3-oxazolidine-2-thione (0.5 mmol, 1.0 equiv.), the corresponding propargyl cobaltated acetal (0.55 mmol, 1.1 equiv.), and [(R)-DTBM-SEGPHOS]NiCl$_2$ (2-5 mol%) in DCM (2 mL) was cooled to 0 °C. Then, TMSOTf (185 µL, 1.0 mmol, 2.0 equiv.) was added dropwise and stirred for 3 min. 2,6-Lutidine (90 µL, 0.75 mmol, 1.5 equiv.) was then added and the resultant mixture was stirred at 0 °C for the desired time.

The mixture was then quenched with a saturated solution of NH_4Cl (2 mL) and extracted with DCM (3 × 5 mL). The combined organic extracts were dried over $MgSO_4$, filtered through Celite®, and concentrated in vacuo. Finally, the crude was purified by column chromatography to yield the named compound as a single enantiomer. The syn/anti diasteromeric ratio (dr) was established in each case by ^{1}H NMR analysis of the crude mixture.

N-[(2S,3S)-Hexacarbonyl{µ-[η^4-(3-ethoxy-2-methyl-5-phenyl-4-pentynoyl)]dicobalt (Co-Co)}]-1,3-oxazolidine-2-thione (3a)

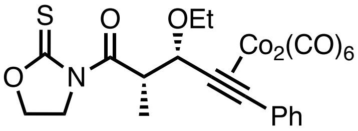

According to General Procedure E, the title compound was obtained from N-propanoyl-1,3-oxazolidine-2-thione (**1a**, 80 mg, 0.5 mmol, 1.0 equiv.) and hexacarbonyl µ-[η^4-(1,1-diethoxy-3-phenylpropyne)dicobalt] (**2a**, 270 mg, 0.55 mmol, 1.1 equiv.) at 0 °C for 5 h. The analysis of the resultant oil by ^{1}H NMR established the formation of a single diastereomer.

The crude was purified by column chromatography (Hex/EtOAc, from 1:0 to 75:25) to afford 300 mg (0.5 mmol, 99% yield) of adduct **3a**.

The reaction was also carried out on a 1.5 mmol scale following General Procedure E with 239 mg of **1a** (1.5 mmol, 1.0 equiv.), 809 mg of **2a** (1.65 mmol, 1.1 equiv.) to yield 878 mg (1.46 mmol, 97% yield) of adduct **3a**.

Dark brown oil.

R_f 0.3 (Hex/EtOAc, 80:20).

IR (ATR) ν 3076, 3053, 2974, 2930, 2876, 2089, 2048, 1999, 1777, 1692, 1603, 1486, 1439, 1385, 1367, 1324, 1254, 1188, 1158, 1085 cm^{-1}.

^{1}H NMR (500 MHz, $CDCl_3$) δ 7.48 – 7.40 (m, 2H, ArH), 7.35 – 7.28 (m, 3H, ArH), 5.10 (d, J = 8.4 Hz, 1H, CHOEt), 4.83 (dq, J = 8.4, 6.9 Hz, 1H, COCHCH3), 4.38 (td, J = 9.3, 6.3 Hz, 1H, $OCH_aH_bCH_2$), 4.17 (q, J = 9.3 Hz, 1H, $OCH_aH_bCH_2$), 4.00 – 3.90 (m, 1H, $NCH_aH_bCH_2$), 3.82 (dq, J = 8.7, 7.0 Hz, 1H, $OCH_aH_bCH_3$), 3.67 (dq, J = 8.7, 7.0 Hz, 1H, $OCH_aH_bCH_3$), 3.52 (ddd, J = 11.3, 9.3, 6.3 Hz, 1H, $NCH_aH_bCH_2$), 1.43 (d, J = 6.9 Hz, 3H, $COCHCH_3$), 1.21 (t, J = 7.0 Hz, 3H, OCH_2CH_3).

^{13}C{^{1}H} NMR (126 MHz, $CDCl_3$) δ 199.4 (CO), 184.5 (C), 175.3 (C), 138.4 (C), 129.7 (CH), 128.4 (CH), 127.3 (CH), 98.4 (C), 93.2 (C), 80.7 (CH), 68.3 (CH_2), 66.0 (CH_2), 46.9 (CH_2), 45.9 (CH), 15.3 (CH_3), 15.0 (CH_3).

N-[(2S,3S)-Hexacarbonyl{μ-[η^4-(3-methoxy-2-methyl-5-phenyl-4-pentynoyl)]dicobalt (Co-Co)}]-1,3-oxazolidine-2-thione (3b)

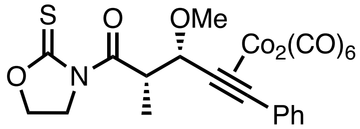

According to General Procedure E, the title compound was obtained from N-propanoyl-1,3-oxazolidine-2-thione (**1a**, 80 mg, 0.5 mmol, 1.0 equiv.) and hexacarbonyl μ-[η4-(-(1,1-dimethoxy-3-phenylpropyne)dicobalt] (**2b**, 254 mg, 0.55 mmol, 1.1 equiv.) at 0 °C for 5 h. The analysis of the resultant oil by ^{1}H NMR established the formation of a single diastereomer.

The crude was purified by column chromatography (Hex/EtOAc, from 85:15 to 70:30) to afford 255 mg (0.43 mmol, 86% yield) of adduct **3b**.

Dark brown oil.

R_f 0.2 (Hex/EtOAc, 85:15).

IR (ATR) ν 3056, 2930, 2827, 2089, 2048, 1994, 1690, 1366, 1325, 1290, 1254, 1185, 1157, 1090 cm^{-1}.

^{1}H NMR (400 MHz, CDCl$_3$) δ 7.48 – 7.42 (m, 2H, ArH), 7.35 – 7.25 (m, 3H, ArH), 5.01 (d, J = 8.1 Hz, 1H, CHOEt), 4.89 (dq, J = 8.1, 6.8 Hz, 1H, COCHCH$_3$), 4.40 (ddd, J = 9.2, 8.9, 6.5 Hz, 1H, OCH$_a$H$_b$CH$_2$), 4.22 (q, J = 8.4 Hz, 1H, OCH$_a$H$_b$CH$_2$), 4.08 – 3.93 (m, 1H, NCH$_a$H$_b$CH$_2$), 3.65 – 3.52 (m, 1H, NCH$_a$H$_b$CH$_2$), 3.58 (s, 3H, OCH$_3$), 1.43 (d, J = 6.8 Hz, 3H, COCHCH$_3$).

^{13}C{^{1}H} NMR (101 MHz, CDCl$_3$) δ 199.5 (CO), 184.7 (C), 175.4 (C), 138.3 (C), 129.8 (CH), 128.6 (CH), 127.5 (CH), 97.5 (C), 93.3 (C), 82.7 (CH), 66.1 (CH$_2$), 60.8 (CH$_3$), 47.1 (CH$_2$), 46.0 (CH), 15.2 (CH$_3$).

N-[(2S,3S)-Hexacarbonyl{μ-[η^4-(3-benzyloxy-2-methyl-5-phenyl-4-pentynoyl)]dicobalt(Co-Co)}]-1,3-oxazolidine-2-thione (3c)

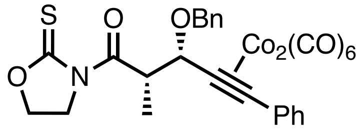

According to General Procedure E, the title compound was obtained from N-propanoyl-1,3-oxazolidine-2-thione (**1a**, 40 mg, 0.25 mmol, 1.0 equiv.) and hexacarbonyl μ-[η4-(-(1,1-dibenzyloxy-3-phenylpropyne)dicobalt] (**2c**, 167 mg, 0.275 mmol, 1.1 equiv.) at 0 °C for 16 h. The analysis of the resultant oil by ^{1}H NMR established the formation of a single diastereomer.

The crude was purified by column chromatography (Hex/EtOAc, from 90:10 to 75:25) to afford 128 mg (0.19 mmol, 77% yield) of adduct **3c**.

Dark brown oil.

R_f 0.3 (Hex/EtOAc, 80:20).

IR (ATR) ν 3064, 3030, 2920, 2853, 2089, 2048, 1992, 1687, 1366, 1323, 1189 cm^{-1}.

^{1}H NMR (400 MHz, CDCl$_3$) δ 7.51 – 7.42 (m, 2H, ArH), 7.37 – 7.27 (m, 8H, ArH), 5.35 (d, J = 7.8 Hz, 1H, CHOBn), 5.00 – 4.88 (m, 1H, COCHCH$_3$), 4.81 (d, J = 11.3 Hz, 1H, OCH$_a$H$_b$Ph), 4.61 (d, J = 11.3 Hz, 1H, OCH$_a$H$_b$Ph), 4.38 (ddd, J = 9.5, 8.8, 6.4 Hz, 1H, OCH$_a$H$_b$CH$_2$, 4.22 – 4.11 (m, 1H, OCH$_a$H$_b$CH$_2$), 3.95 (ddd, J = 11.4, 9.5, 8.5 Hz, 1H,

$NC\underline{H}_aH_bCH_2$), 3.55 (ddd, J = 11.4, 9.4, 6.4 Hz, 1H, $NCH_a\underline{H}_bCH_2$), 1.40 (d, J = 6.9 Hz, 3H, $COCHC\underline{H}_3$).

$^{13}C\{^1H\}$ NMR (101 MHz, $CDCl_3$) δ 199.5 (CO), 184.6 (C), 175.2 (C), 138.5 (C), 137.8 (C), 129.8 (CH), 128.7 (CH), 128.4 (CH), 128.2 (CH), 127.9 (CH), 127.6 (CH), 97.5 (C), 94.0 (C), 80.2 (CH), 74.2 (CH_2), 66.1 (CH_2), 47.1 (CH_2), 46.0 (CH), 15.2 (CH_3).

N-[(2S,3S)-Hexacarbonyl{μ-[η^4-(5-tert-butyldimethylsilyl-3-ethoxy-2-methyl-4-pentynoyl)]dicobalt(Co-Co)}]-1,3-oxazolidine-2-thione (**3d**)

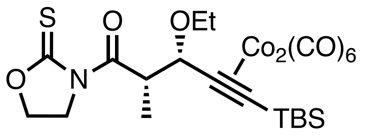

According to General Procedure E, the title compound was obtained from N-propanoyl-1,3-oxazolidine-2-thione (**1a**, 80 mg, 0.5 mmol, 1.0 equiv.) and hexacarbonyl μ-[η4-(1,1-diethoxy-3-(tert-butildimethylsilane)propyne)dicobalt] (**2d**, 298 mg, 0.55 mmol, 1.1 equiv.) at 0 °C for 16 h. The analysis of the resultant oil by ^1H NMR established the formation of a single diastereomer.

The crude was purified by column chromatography (Hex/EtOAc, from 100:0 to 80:20) to afford 299 mg (0.47 mmol, 93% yield) of adduct **3d**.

Dark brown solid.

Mp 102–104 °C.

R_f 0.3 (Hex/EtOAc, 85:15).

IR (ATR) ν 2958, 2930, 2883, 2857, 2085, 2044, 1998, 1782, 1690, 1577, 1472, 1377, 1325, 1284, 1250, 1189, 1157, 1115, 1075 cm^{-1}.

^1H NMR (400 MHz, $CDCl_3$) δ 5.03 (d, J = 6.1 Hz, 1H, $C\underline{H}OEt$), 4.75 (qd, J = 7.1, 6.1 Hz, 1H, $COC\underline{H}CH_3$), 4.58 – 4.42 (m, 2H, $OC\underline{H}_2CH_2$), 4.31 – 4.15 (m, 2H, $NC\underline{H}_2CH_2$), 3.87 – 3.72 (m, 2H, $OC\underline{H}_2CH_3$), 1.47 (d, J = 7.1 Hz, 3H, $COCHC\underline{H}_3$), 1.21 (t, J = 7.0 Hz, 3H, $OCH_2C\underline{H}_3$), 1.02 (s, 9H, $SiC(C\underline{H}_3)_3$), 0.28 (s, 3H, $SiC\underline{H}_3$), 0.28 (s, 3H, $SiC\underline{H}_3$).

$^{13}C\{^1H\}$ NMR (101 MHz, $CDCl_3$) δ 200.5 (CO), 184.5 (C), 176.3 (C), 112.3 (C), 79.4 (CH), 76.6 (C), 68.0 (CH_2), 66.1 (CH_2), 47.6 (CH_2), 47.0 (CH), 27.4 (CH_3), 18.9 (C), 15.1 (CH_3), 14.7 (CH_3), – 2.06 (CH_3), – 2.13 (CH_3).

N-[(2S,3S)-Hexacarbonyl{μ-[η^4-(3-ethoxy-2-methyl-4-nonynoyl)]dicobalt(Co-Co)}]-1,3-oxazolidine-2-thione (3e)

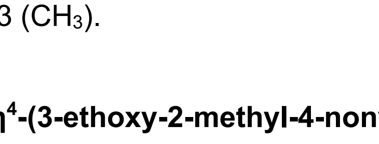

According to General Procedure E, the title compound was obtained from N-propanoyl-1,3-oxazolidine-2-thione (**1a**, 32 mg, 0.2 mmol, 1.0 equiv.) and hexacarbonyl μ-[η4-(1,1-diethoxy-2-heptyne)dicobalt] (**2e**, 104 mg, 0.22 mmol, 1.1 equiv.) at 0 °C for 3 h. The analysis of the resultant oil by ^{1}H NMR established the formation of a single diastereomer.

The crude was purified by column chromatography (Hex/EtOAc, from 85:15 to 80:20) to afford 102 mg (0.18 mmol, 88% yield) of adduct **3e**.

Dark brown oil.
R$_f$ 0.2 (Hex/EtOAc, 85:15).
IR (ATR) ν 2961, 2932, 2874, 2087, 2044, 1992, 1690, 1366, 1325, 1251, 1187, 1157, 1081, 1012, 967, 943 cm^{-1}.
^{1}H NMR (500 MHz, CDCl$_3$) δ 4.94 – 4.82 (m, 2H, COC<u>H</u>CH$_3$, C<u>H</u>OEt), 4.53 (q, J = 8.6 Hz, 1H, OC<u>H</u>$_a$H$_b$CH$_2$), 4.46 (q, J = 8.6 Hz, 1H, OCH$_a$<u>H</u>$_b$CH$_2$), 4.22 (t, J = 8.6 Hz, 2H, NC<u>H</u>$_2$CH$_2$), 3.83 (dq, J = 8.2, 7.0 Hz, 1H, OC<u>H</u>$_a$H$_b$CH$_3$), 3.68 (dq, J = 8.2, 6.9 Hz, 1H, OCH$_a$<u>H</u>$_b$CH$_3$), 2.82 – 2.65 (m, 2H, C≡CC<u>H</u>$_2$), 1.66 – 1.56 (m, 2H, C≡CCH$_2$C<u>H</u>$_2$), 1.53 – 1.39 (m, 5H, CH$_2$C<u>H</u>$_2$CH$_3$, COCHC<u>H</u>$_3$), 1.23 (t, J = 7.0 Hz, 3H, OCH$_2$C<u>H</u>$_3$), 0.97 (t, J = 7.3 Hz, 3H, CH$_2$CH$_2$C<u>H</u>$_3$).
^{13}C{^{1}H} NMR (126 MHz, CDCl$_3$) δ 200.4 (CO), 184.6 (C), 175.6 (C), 100.3 (C), 96.8 (C), 80.4 (CH), 68.0 (CH$_2$), 66.0 (CH$_2$), 47.3 (CH$_2$), 45.8 (CH), 34.0 (CH$_2$), 33.5 (CH$_2$), 22.8 (CH$_3$), 15.1 (CH$_3$), 13.9 (CH$_3$).

N-[(2S,3S)-Hexacarbonyl{μ-[η^4-(3-ethoxy-2-methyl-7-octen-4-ynoyl)]dicobalt(Co-Co)}]-1,3-oxazolidine-2-thione (3f)

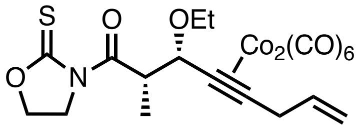

According to General Procedure E, the title compound was obtained from N-propanoyl-1,3-oxazolidine-2-thione (**1a**, 80 mg, 0.5 mmol, 1.0 equiv.) and hexacarbonyl μ-[η4-(6,6-diethoxy-1-hexen-4-yne)dicobalt] (**2f**, 250 mg, 0.55 mmol, 1.1 equiv.) at 0 °C for 5 h. The analysis of the resultant oil by ^{1}H NMR established the formation of a single diastereomer.

The crude was purified by column chromatography (Hex/EtOAc, from 95:5 to 80:20) to afford 267 mg (0.47 mmol, 94% yield) of adduct **3f**.

Dark brown oil.

R_f 0.3 (Hex/EtOAc, 80:20).

IR (ATR) ν 2978, 2930, 2874, 2089, 2044, 1994, 1690, 1366, 1323, 1187, 1157 cm^{-1}.

^{1}H NMR (400 MHz, $CDCl_3$) δ 5.95 (ddt, J = 16.9, 9.9, 7.1 Hz, 1H, CH_2=C$\underline{H}$$CH_2$), 5.25 – 5.11 (m, 2H, C$\underline{H}_2$=CH), 4.95 – 4.82 (m, 2H, C$\underline{H}$OEt, COC$\underline{H}$$CH_3$), 4.60 – 4.41 (m, 2H, OC$\underline{H}_2$$CH_2$), 4.22 (t, J = 8.5 Hz, 2H, NC$\underline{H}_2$$CH_2$), 3.84 (dq, J = 8.8, 7.0 Hz, 1H, OC$\underline{H}_a$$H_b$$CH_3$), 3.68 (dq, J = 8.8, 7.0 Hz, 1H, OC$H_a$$\underline{H}_b$$CH_3$), 3.56 – 3.40 (m, 2H, CH_2=CHC$\underline{H}_2$), 1.46 (d, J = 6.6 Hz, 3H, COCHC$\underline{H}_3$), 1.24 (t, J = 7.0 Hz, 3H, OCH_2C$\underline{H}_3$).

^{13}C{^{1}H} NMR (101 MHz, $CDCl_3$) δ 200.0 (CO), 184.7 (C), 175.7 (C), 136.0 (CH) , 117.6 (CH_2), 97.9 (C), 96.4 (C), 80.5 (CH), 68.2 (CH_2), 66.1 (CH_2), 47.4 (CH_2), 46.0 (CH), 38.3 (CH_2), 15.3 (CH_3), 15.23 (CH_3).

N-[(2S,3S)-Hexacarbonyl{μ-[η^4-(8-bromo-3-ethoxy-2-methyl-4-octynoyl)]dicobalt(Co-Co)}]-1,3-oxazolidine-2-thione (3g)

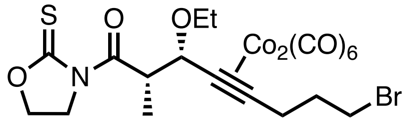

According to General Procedure E, the title compound was obtained from N-propanoyl-1,3-oxazolidine-2-thione (**1a**, 80 mg, 0.5 mmol, 1.0 equiv.) and hexacarbonyl μ-[η^4-(6-bromo-1,1-diethoxy-2-hexyne)dicobalt] (**2g**, 293 mg, 0.55 mmol, 1.1 equiv.) at 0 °C for 3 h. The analysis of the resultant oil by ^{1}H NMR established the formation of a single diastereomer.

The crude was purified by column chromatography (Hex/EtOAc, 80:20) to afford 299 mg (0.48 mmol, 96% yield) of adduct **3g**.

Dark brown oil.

R_f 0.2 (Hex/EtOAc, 85:15).

IR (ATR) ν 2976, 2933, 2915, 2878, 2088, 2045, 2001, 1772, 1690, 1600, 1366, 1324, 1288, 1250, 1187, 1158, 1083, 1011 cm^{-1}.

^{1}H NMR (500 MHz, $CDCl_3$) δ 4.87 – 4.85 (m, 2H, COC$\underline{H}$$CH_3$, C$\underline{H}$OEt), 4.58 – 4.43 (m, 2H, OC$\underline{H}_2$$CH_2$), 4.31 – 4.19 (m, 2H, NC$\underline{H}_2$$CH_2$), 3.85 (dq, J = 8.6, 7.0 Hz, 1H, OC$\underline{H}_a$$H_b$$CH_3$),

3.69 (dq, J = 8.6, 7.0 Hz, 1H, OCH$_a$H$_b$CH$_3$), 3.63 – 3.49 (m, 2H, CH$_2$CH$_2$Br), 2.91 – 2.80 (m, 2H, C≡CCH$_2$), 2.31 – 2.06 (m, 2H, CH$_2$CH$_2$Br), 1.48 – 1.45 (m, 3H, COCHCH$_3$), 1.27 – 1.24 (m, 3H, OCH$_2$CH$_3$).

^{13}C{^{1}H} NMR (126 MHz, CDCl$_3$) δ 199.9 (CO), 184.7 (C), 175.7 (C), 97.5 (C), 97.4 (C), 80.6 (CH), 68.4 (CH$_2$), 66.2 (CH$_2$), 47.5 (CH$_2$), 46.1 (CH), 34.3 (CH$_2$), 33.6 (CH$_2$), 32.4 (CH$_2$), 15.5 (CH$_3$), 15.2 (CH$_3$).

N-[(2S,3S)-Hexacarbonyl{μ-[η^4-(8-azido-3-ethoxy-2-methyl-4-octynoyl)]dicobalt(Co-Co)}]-1,3-oxazolidine-2-thione (3h)

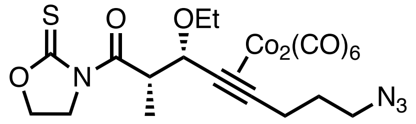

According to General Procedure E, the title compound was obtained from N-propanoyl-1,3-oxazolidine-2-thione (**1a**, 80 mg, 0.5 mmol, 1.0 equiv.) and hexacarbonyl μ-[η^4-(1,1-diethoxy-3- (trimethylsilyl)propyne)dicobalt] (**2h**, 273 mg, 0.55 mmol, 1.1 equiv.) at 0 °C for 5 h. The analysis of the resultant oil by ^{1}H NMR established the formation of a single diastereomer.

The crude was purified by column chromatography (Hex/EtOAc, from 90:10 to 60:40) to afford 270 mg (0.44 mmol, 89% yield) of adduct **3h**.

Dark brown oil.

R$_f$ 0.3 (Hex/EtOAc, 80:20).

IR (ATR) ν 2976, 2932, 2874, 2087, 2044, 1996, 1690, 1366, 1325, 1288, 1251, 1187, 1157, 1083, 1042, 1012, 967, 945 cm^{-1}.

^{1}H NMR (400 MHz, CDCl$_3$) δ 4.94 – 4.81 (m, 2H, COCHCH$_3$, CHOEt), 4.59 – 4.42 (m, 2H, OCH$_2$CH$_2$), 4.31 – 4.16 (m, 2H, NCH$_2$CH$_2$), 3.83 (dq, J = 8.7, 7.0 Hz, 1H, OCH$_a$H$_b$CH$_3$), 3.69 (dq, J = 8.7, 7.0 Hz, 1H, OCH$_a$H$_b$CH$_3$), 3.47 (t, J = 6.3 Hz, 2H, CH$_2$CH$_2$N$_3$), 2.80 (ddd, J = 9.0, 6.3, 1.8 Hz, 2H, C≡CCH$_2$), 2.04 – 1.80 (m, 2H, CH$_2$CH$_2$N$_3$), 1.51 – 1.38 (m, 3H, COCHCH$_3$), 1.24 (t, J = 7.0 Hz, 3H, OCH$_2$CH$_3$).

^{13}C{^{1}H} NMR (101 MHz, CDCl$_3$) δ 199.8 (CO), 184.7 (C), 175.7 (C), 98.1 (C), 97.3 (C), 80.5 (CH), 68.3 (CH$_2$), 66.17 (CH$_2$), 51.3 (CH$_2$), 47.4 (CH$_2$), 46.1 (CH), 31.2 (CH$_2$), 31.1 (CH$_2$), 15.4 (CH$_3$), 15.2 (CH$_3$).

N-[(2S,3S)-Hexacarbonyl{μ-[η[4]-(8-acetoxy-3-ethoxy-2-methyl-4-octynoyl)]dicobalt (Co-Co)}]-1,3-oxazolidine-2-thione (3i)

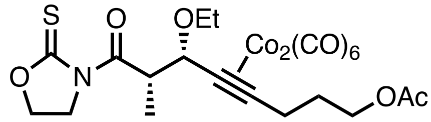

According to General Procedure E, the title compound was obtained from N-propanoyl-1,3-oxazolidine-2-thione (**1a**, 80 mg, 0.5 mmol, 1.0 equiv.) and hexacarbonyl μ-[η4-(6-azido-1,1-diethoxy-2-hexyne)dicobalt] (**2i**, 283 mg, 0.55 mmol, 1.1 equiv.) at 0 °C for 3 h. The analysis of the resultant oil by ^{1}H NMR established the formation of a single diastereomer.

The crude was purified by column chromatography (Hex/EtOAc, from 90:10 to 65:35) to afford 311 mg (0.49 mmol, 99% yield) of adduct **3i**.

Dark brown solid.
Mp 66–68 °C.
R_f 0.3 (Hex/EtOAc, 70:30).
IR (ATR) ν 2972, 2931, 2087, 2044, 1990, 1731, 1688, 1364, 1321, 1250, 1157, 1084, 1043, 944 cm^{-1}.
^{1}H NMR (400 MHz, $CDCl_3$) δ 4.97 – 4.83 (m, 2H, COC<u>H</u>CH$_3$, C<u>H</u>OEt), 4.58 – 4.44 (m, 2H, OC<u>H</u>$_2$CH$_2$), 4.29 – 4.13 (m, 4H, NC<u>H</u>$_2$CH$_2$,CH$_2$C<u>H</u>$_2$OAc), 3.83 (dq, J = 8.6, 7.0 Hz, 1H, OC<u>H</u>$_a$H$_b$CH$_3$), 3.68 (dq, J = 8.6, 7.0 Hz, 1H, OCH$_a$<u>H</u>$_b$CH$_3$), 2.75 (t, J = 8.2 Hz, 2H, C≡CC<u>H</u>$_2$), 2.07 (s, 3H, COC<u>H</u>$_3$), 2.03 – 1.86 (m, 2H, C<u>H</u>$_2$CH$_2$OAc), 1.50 – 1.39 (d, J = 6.5 Hz, 3H, COCHC<u>H</u>$_3$), 1.24 (t, J = 7.0 Hz, 3H, OCH$_2$C<u>H</u>$_3$).
^{13}C{^{1}H} NMR (101 MHz, $CDCl_3$) δ 199.9 (CO), 184.6 (C), 175.6 (C), 171.2 (C), 98.4 (C), 97.2 (C), 80.4 (CH), 68.2 (CH$_2$), 66.1 (CH$_2$), 63.6 (CH$_2$), 47.3 (CH$_2$), 45.8 (CH), 31.0 (CH$_2$), 30.4 (CH$_2$), 21.0 (CH$_3$), 15.2 (CH$_3$), 15.1 (CH$_3$).

N-[(2S,3S)-Hexacarbonyl{μ-[η[4]-(3-ethoxy-2-methyl-8-pivaloylxy-4-octynoyl)]dicobalt (Co-Co)}]-1,3-oxazolidine-2-thione (3j)

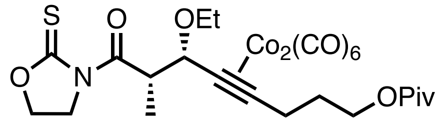

According to General Procedure E, the title compound was obtained from N-propanoyl-1,3-oxazolidine-2-thione (**1a**, 80 mg, 0.5 mmol, 1.0 equiv.) and hexacarbonyl μ-[η4-(6-pivaloyloxy-1,1-diethoxy-2-hexyne)dicobalt] (**2j**, 306 mg, 0.55 mmol, 1.1 equiv.) at 0 °C for 3 h. The analysis of the resultant oil by ^{1}H NMR established the formation of a single diastereomer.

The crude was purified by column chromatography (Hex/EtOAc, from 80:20 to 70:30) to afford 314 mg (0.47 mmol, 94% yield) of adduct **3j**.

Dark brown oil.
R_f 0.3 (Hex/EtOAc, 80:20).
IR (ATR) ν 2972, 2933, 2874, 2089, 2046, 1999, 1720, 1690, 1601, 1479, 1459, 1366, 1325, 1284, 1254, 1189, 1154, 1082cm^{-1}.
^{1}H NMR (400 MHz, CDCl$_3$) δ 4.92 – 4.84 (m, 2H, COC<u>H</u>CH$_3$, C<u>H</u>OEt), 4.59 – 4.44 (m, 2H, OC<u>H</u>$_2$CH$_2$), 4.28 – 4.17 (m, 4H, NC<u>H</u>$_2$CH$_2$, CH$_2$C<u>H</u>$_2$OPiv), 3.83 (dq, J = 8.8, 7.0 Hz, 1H, OC<u>H</u>$_a$H$_b$CH$_3$), 3.68 (dq, J = 8.8, 7.0 Hz, 1H, OCH$_a$<u>H</u>$_b$CH$_3$), 2.81 – 2.73 (m, 2H, C≡CC<u>H</u>$_2$), 2.06 – 1.86 (m, 2H, CH$_2$C<u>H</u>$_2$CH$_2$OPiv), 1.45 (d, J = 6.7 Hz, 3H, COCHC<u>H</u>$_3$), 1.23 (t, J = 7.0 Hz, 3H, OCH$_2$C<u>H</u>$_3$), 1.20 (s, 9H, C(C<u>H</u>$_3$)$_3$).
^{13}C{^{1}H} NMR (101 MHz, CDCl$_3$) δ 200.0 (CO), 184.7 (C), 178.7 (C), 175.7 (C), 98.8 (C), 97.2 (C), 80.6 (CH), 68.3 (CH$_2$), 66.2 (CH$_2$), 63.6 (CH$_2$), 47.4 (CH$_2$), 46.0 (CH), 38.9 (C), 31.3 (CH$_2$), 30.5 (CH$_2$), 27.3 (CH$_3$), 15.3 (CH$_3$), 15.2 (CH$_3$).

N-[(2S,3S)-Hexacarbonyl{μ-[η^{4}-(8-tert-butyldiphenylsilyloxy-3-ethoxy-2-methyl-4-octynoyl)]dicobalt(Co-Co)}]-1,3-oxazolidine-2-thione (3k)

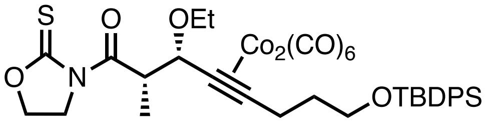

According to General Procedure E, the title compound was obtained from N-propanoyl-1,3-oxazolidine-2-thione (**1a**, 40 mg, 0.25 mmol, 1.0 equiv.) and hexacarbonyl μ-[η4-(6-tert-butyldiphenylsilyloxy-1,1-diethoxy-2-hexyne)dicobalt] (**2k**, 196 mg, 0.275 mmol, 1.1 equiv.) at 0 °C for 3 h. The analysis of the resultant oil by ^{1}H NMR established the formation of a single diastereomer.

The crude was purified by column chromatography (Hex/EtOAc, from 90:10 to 70:30) to afford 163 mg (0.20 mmol, 79% yield) of adduct **3k**.

Dark brown oil.

R$_f$ 0.3 (Hex/EtOAc, 80:20).

IR (ATR) ν 2928, 2857, 2087, 2044, 2003, 1779, 1690, 1589, 1472, 1427, 1379, 1325, 1288, 1252, 1187, 1157, 1105, 1083 cm^{-1}.

^{1}H NMR (400 MHz, CDCl$_3$) δ 7.71 – 7.62 (m, 4H, ArH), 7.47 – 7.34 (m, 6H, ArH), 4.92 – 4.79 (m, 2H, COCHCH$_3$, CHOEt), 4.40 (q, J = 8.5 Hz, 1H, OCH$_a$H$_b$CH$_2$), 4.27 (td, J = 9.2, 7.8 Hz, 1H, OCH$_a$H$_b$CH$_2$), 4.17 – 4.06 (m, 2H, NCH$_2$CH$_2$), 3.89 – 3.77 (m, 3H, OCH$_a$H$_b$CH$_3$, CH$_2$CH$_2$OTBDPS), 3.66 (dq, J = 8.7, 7.0 Hz, 1H, OCH$_a$H$_b$CH$_3$), 2.92 – 2.79 (m, 2H, C≡CCH$_2$), 1.98 – 1.78 (m, 2H, C≡CCH$_2$CH$_2$), 1.50 – 1.39 (m, 3H, COCHCH$_3$), 1.22 (t, J = 7.0 Hz, 3H, OCH$_2$CH$_3$), 1.06 (s, 9H, C(CH$_3$)$_3$).

^{13}C{^{1}H} NMR (101 MHz, CDCl$_3$) δ 200.0 (CO), 184.7 (C), 175.6 (C), 135.6 (CH), 134.0 (C), 129.8 (CH), 127.9 (CH), 100.0 (C), 97.0 (C), 80.6 (CH), 77.4 (CH$_2$), 68.2 (CH$_2$), 66.0 (CH$_2$), 63.6 (CH$_2$), 47.4 (CH$_2$), 46.0 (CH), 35.0 (CH$_2$), 30.7 (CH$_2$), 27.4 (CH$_3$), 19.4 (C), 15.4 (CH$_3$), 15.2 (CH$_3$).

N-[(2S,3S)-Hexacarbonyl{μ-[η^4-(3-ethoxy-8-methoxy-2-methyl-4-octynoyl)]dicobalt (Co-Co)}]-1,3-oxazolidine-2-thione (3l)

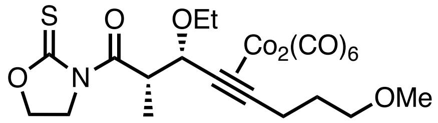

According to General Procedure E, the title compound was obtained from N-propanoyl-1,3-oxazolidine-2-thione (**1a**, 80 mg, 0.5 mmol, 1.0 equiv.) and hexacarbonyl μ-[η4-(6-methoxy-1,1-diethoxy-2-hexyne)dicobalt] (**2l**, 277 mg, 0.55 mmol, 1.1 equiv.) at 0 °C for 5 h. The analysis of the resultant oil by ^{1}H NMR established the formation of a single diastereomer.

The crude was purified by column chromatography (Hex/EtOAc, from 80:20 to 70:30) to afford 279 mg (0.47 mmol, 93% yield) of adduct **3l**.

Dark brown oil.

R$_f$ 0.3 (Hex/EtOAc, 70:30).

IR (ATR) ν 2978, 2928, 2874, 2087, 2044, 1992, 1781, 1690, 1474, 1448, 1377, 1325, 1288, 1252, 1185, 1157, 1083 cm^{-1}.

^{1}H NMR (500 MHz, CDCl$_3$) δ 4.90 – 4.78 (m, 2H, COC<u>H</u>CH$_3$, C<u>H</u>OEt), 4.60 – 4.48 (m, 1H, OC<u>H</u>$_a$H$_b$CH$_2$), 4.46 (q, J = 9.0 Hz, 1H, OCH$_a$<u>H</u>$_b$CH$_2$), 4.33 – 4.14 (m, 2H, NC<u>H</u>$_2$CH$_2$), 3.85 (dq, J = 8.7, 7.0 Hz, 1H, OC<u>H</u>$_a$H$_b$CH$_3$), 3.69 (dq, J = 8.7, 7.0 Hz, 1H, OCH$_a$<u>H</u>$_b$CH$_3$), 3.51 (t, J = 6.0 Hz, 2H, C<u>H</u>$_2$OMe), 3.35 (s, 3H, OC<u>H</u>$_3$), 2.77 – 2.66 (m, 2H, C≡CC<u>H</u>$_2$), 1.99 – 1.79 (m, 2H, CH$_2$C<u>H</u>$_2$CH$_2$OMe), 1.46 (d, J = 6.4 Hz, 3H, COCHC<u>H</u>$_3$), 1.24 (t, J = 7.0 Hz, 3H, OCH$_2$C<u>H</u>$_3$).

^{13}C{^{1}H} NMR (126 MHz, CDCl$_3$) δ 200.0 (CO), 184.8 (C), 175.7 (C), 99.4 (C), 97.3 (C), 80.6 (CH), 72.2 (CH$_2$), 68.3 (CH$_2$), 66.2 (CH$_2$), 58.7 (CH$_3$), 47.4 (CH$_2$), 46.0 (CH), 31.9 (CH$_2$), 30.8 (CH$_2$), 15.4 (CH$_3$), 15.2 (CH$_3$).

N-[(2S,3S)-Hexacarbonyl{μ-[η^4-(2-butyl-3-ethoxy-5-phenyl-4-pentynoyl)]dicobalt (Co-Co)}]-1,3-oxazolidine-2-thione (3m)

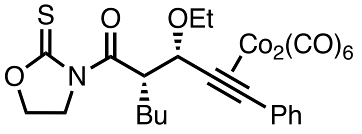

According to General Procedure E, the title compound was obtained from N-pentanoyl-1,3-oxazolidine-2-thione (**1b**, 100 mg, 0.50 mmol, 1.0 equiv.) and hexacarbonyl μ-[η^4-(1,1-diethoxy-3-phenylpropyne)dicobalt] (**2a**, 270 mg, 0.55 mmol, 1.1 equiv.) at 0 °C for 16 h. The analysis of the resultant oil by ^{1}H NMR established the formation of a single diastereomer.

The crude was purified by column chromatography (Hex/EtOAc, from 95:5 to 85:15) to afford 303 mg (0.47 mmol, 94% yield) of adduct **3m**.

Dark brown oil.

R$_f$ 0.3 (Hex/EtOAc, 85:15).

IR (ATR) ν 2958, 2930, 2872, 2089, 2050, 2018, 1696, 1370, 1321, 1187, 1156 cm^{-1}.

^{1}H NMR (400 MHz, CDCl$_3$) δ 7.52 – 7.39 (m, 2H, Ar<u>H</u>), 7.35 – 7.26 (m, 3H, Ar<u>H</u>), 5.17 (d, J = 7.8 Hz, 1H, C<u>H</u>OEt), 5.10 – 5.01 (m, 1H, COC<u>H</u>Bu), 4.41 (td, J = 9.5, 7.0 Hz, 1H, OC<u>H</u>$_a$H$_b$CH$_2$), 4.26 (td, J = 9.5, 8.0 Hz, 1H, OCH$_a$<u>H</u>$_b$CH$_2$), 4.02 (ddd, J = 11.4, 9.5, 8.0 Hz, 1H, NC<u>H</u>$_a$H$_b$CH$_2$), 3.80 (dq, J = 8.7, 7.0 Hz, 1H, OC<u>H</u>$_a$H$_b$CH$_3$), 3.69 (ddd, J = 11.4, 9.5, 7.0 Hz, 1H, NCH$_a$<u>H</u>$_b$CH$_2$), 3.57 (dq, J = 8.7, 7.0 Hz, 1H, OCH$_a$<u>H</u>$_b$CH$_3$), 2.02 (dddd, J = 13.6, 11.1, 6.7, 4.3 Hz, 1H, COCHC<u>H</u>$_a$H$_b$), 1.85 – 1.72 (m, 1H, COCHCH$_a$<u>H</u>$_b$), 1.39 – 1.21 (m,

4H, $CH_2C\underline{H}_2CH_3$, $C\underline{H}_2CH_2CH_3$), 1.19 (t, J = 7.0 Hz, 3H, $OCH_2C\underline{H}_3$), 0.81 (t, J = 7.0 Hz, 3H, $CH_2CH_2C\underline{H}_3$).

$^{13}C\{^1H\}$ NMR (101 MHz, $CDCl_3$) δ 199.6 (CO), 184.8 (C), 174.0 (C), 138.6 (C), 129.8 (CH), 128.5 (CH), 127.4 (C), 98.3 (C), 94.0 (C), 80.1 (CH), 68.2 (CH_2), 66.0 (CH_2), 50.0 (CH), 47.2 (CH_2), 28.3 (CH_2), 28.1 (CH_2), 23.2 (CH_2), 15.2 (CH_3), 13.9 (CH_3).

N-[(2S,3S)-Hexacarbonyl{μ-[η^4-(3-ethoxy-2-isobutyl-5-phenyl-4-pentynoyl)]dicobalt (Co-Co)}]-1,3-oxazolidine-2-thione (3n)

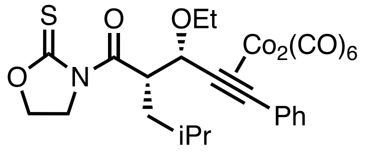

According to General Procedure E, the title compound was obtained from N-(4-methylpentanoyl)-1,3-oxazolidine-2-thione (**1c**, 101 mg, 0.50 mmol, 1.0 equiv.) and hexacarbonyl μ-[η^4-(1,1-diethoxy-3-phenylpropyne)dicobalt] (**2a**, 270 mg, 0.55 mmol, 1.1 equiv.), with a 5 mol% of [(R)-DTBM-SEGPHOS]$NiCl_2$ (32.73 mg, 0.02 mmol, 5 mol%) at 0 °C for 16 h. The analysis of the resultant oil by ^{1}H NMR established the formation of a single diastereomer.

The crude was purified by column chromatography (Hex/EtOAc, from 1:0 to 75:25) to afford 294 mg (0.45 mmol, 91% yield) of adduct **3n**.

Dark brown oil.

R_f 0.3 (Hex/EtOAc, 80:20).

IR (ATR) ν 3100, 2958, 2928, 2870, 2089, 2048, 2000, 1700, 1368, 1321 cm^{-1}.

^{1}H NMR (400 MHz, $CDCl_3$) δ 7.55 – 7.44 (m, 2H, Ar$\underline{H}$), 7.36 – 7.23 (m, 3H, Ar$\underline{H}$), 5.33 (ddd, J = 9.4, 5.3, 4.0 Hz, 1H, COC$\underline{H}$), 5.13 (d, J = 5.3 Hz, 1H, C$\underline{H}$OEt), 4.48 (td, J = 9.3, 6.7 Hz, 1H, $OC\underline{H}_aH_bCH_2$), 4.43 – 4.34 (m, 1H, $OCH_a\underline{H}_bCH_2$), 4.12 (ddd, J = 11.5, 9.3, 8.2 Hz, 1H, $NC\underline{H}_aH_bCH_2$), 3.90 (ddd, J = 11.5, 9.3, 6.7 Hz, 1H, $NCH_a\underline{H}_bCH_2$), 3.75 (dq, J = 8.6, 7.0 Hz, 1H, $OC\underline{H}_aH_bCH_3$), 3.56 (dq, J = 8.6, 7.0 Hz, 1H, $OCH_a\underline{H}_bCH_3$), 2.07 – 1.93 (m, 1H, $COCHC\underline{H}_aCH_b$), 1.59 (dqd, J = 8.6, 6.6, 5.1 Hz, 1H, $C\underline{H}(CH_3)_2$), 1.51 – 1.40 (m, 1H, $COCHCH_aC\underline{H}_b$), 1.18 (t, J = 7.0 Hz, 3H, $OCH_2C\underline{H}_3$), 0.79 (d, J = 6.6 Hz, 3H, $CH(C\underline{H}_3)_a(CH_3)_b$), 0.69 (d, J = 6.6 Hz, 3H, $CH(CH_3)_a(C\underline{H}_3)_b$).

$^{13}C\{^1H\}$ NMR (101 MHz, $CDCl_3$) δ 199.6 (CO), 185.2 (C), 174.1 (C), 138.5 (C), 129.8 (CH), 128.6 (CH), 127.5 (CH), 96.2 (C), 94.0 (C), 81.5 (CH), 68.3 (CH_2), 66.1 (CH_2), 47.6 (CH_2), 47.4 (CH), 36.7 (CH_2), 25.8 (CH), 23.7 (CH_3), 22.3 (CH_3), 15.1 (CH_3).

N-[(2S,3S)-Hexacarbonyl{μ-[η4-(2-cyanomethyl-3-ethoxy-5-phenyl-4-pentynoyl)] dicobalt(Co-Co)}]-1,3-oxazolidine-2-thione (3o)

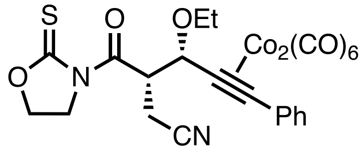

According to General Procedure E, the title compound was obtained from N-(3-cyanopropanoyl)-1,3-oxazolidine-2-thione (**1d**, 92 mg, 0.50 mmol, 1.0 equiv.) and hexacarbonyl μ-[η4-(1,1-diethoxy-3-phenylpropyne)dicobalt] (**2a**, 270 mg, 0.55 mmol, 1.1 equiv.) at 0 °C for 16 h. The analysis of the resultant oil by ^{1}H NMR established the formation of a single diastereomer.

The crude was purified by column chromatography (Hex/EtOAc, from 90:10 to 65:35) to afford 299 mg (0.48 mmol, 95% yield) of adduct **3o**.

Dark brown oil.
R_f 0.3 (Hex/EtOAc, 80:20).
IR (ATR) ν 3056, 2976, 2924, 2250, 2091, 2051, 2000, 1690, 1381, 1323 cm^{-1}.
^{1}H NMR (400 MHz, $CDCl_3$) δ 7.47 – 7.40 (m, 2H, ArH), 7.38 – 7.28 (m, 3H, ArH), 5.39 (d, J = 8.5 Hz, 1H, CHOEt), 5.10 – 5.01 (m, 1H, COCH), 4.49 (td, J = 9.4, 6.4 Hz, 1H, $OCH_aH_bCH_2$), 4.34 – 4.22 (m, 1H, $OCH_aH_bCH_2$), 4.10 (q, J = 9.4 Hz, 1H, $NCH_aH_bCH_2$), 3.90 – 3.76 (m, 1H, $OCH_aH_bCH_3$), 3.75 – 3.60 (m, 2H, $OCH_aH_bCH_3$, $NCH_aH_bCH_2$) , 3.22 (dd, J = 17.3, 6.2 Hz, 1H, CH_aH_bCN), 2.97 (dd, J = 17.3, 3.8 Hz, 1H, CH_aH_bCN), 1.22 (t, J = 7.0 Hz, 3H, OCH_2CH_3).
^{13}C{^{1}H} NMR (101 MHz, $CDCl_3$) δ 199.1 (CO), 184.7 (C), 170.5 (C), 138.0 (C), 129.6 (CH), 128.8 (CH), 127.8 (CH), 117.3 (C), 95.9 (C), 93.8 (C), 78.2 (CH), 68.7 (CH_2), 66.7 (CH_2), 47.2 (CH), 46.9 (CH_2), 17.8 (CH_2), 15.1 (CH_3).

N-[(2S,3S)-Hexacarbonyl{μ-[η[4]-(3-ethoxy-2-(3-methoxy-3-oxopropyl)5-phenyl-4-pentynoyl)]dicobalt(Co-Co)}]-1,3-oxazolidine-2-thione (3p)

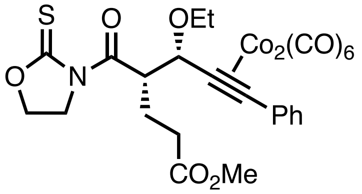

According to General Procedure E, the title compound was obtained from N-(4-methoxycarbonylbutanoyl)-1,3-oxazolidine-2-thione (**1e**, 116 mg, 0.50 mmol, 1.0 equiv.) and hexacarbonyl μ-[η[4]-(1,1-diethoxy-3-phenylpropyne)dicobalt] (**2a**, 270 mg, 0.55 mmol, 1.1 equiv.), with a 5 mol% of [(R)-DTBM-SEGPHOS]$NiCl_2$ (32.73 mg, 0.02 mmol, 5 mol%) at 0 °C for 16 h. The analysis of the resultant oil by ^{1}H NMR established the formation of a single diastereomer.

The crude was purified by column chromatography (Hex/EtOAc, from 80:20 to 65:35) to afford 294 mg (0.44 mmol, 87% yield) of adduct **3p**.

Dark brown oil.
R_f 0.3 (Hex/EtOAc, 70:30).
IR (ATR) ν 3058, 2976, 2950, 2872, 2089, 2048, 2016, 1998, 1733, 1687, 1370 cm^{-1}.
^{1}H NMR (400 MHz, $CDCl_3$) δ 7.46 – 7.37 (m, 2H, Ar<u>H</u>), 7.35 – 7.26 (m, 3H, Ar<u>H</u>), 5.20 (d, J = 8.6 Hz, 1H, C<u>H</u>OEt), 5.02 (dt, J = 8.6, 5.3 Hz, 1H, COC<u>H</u>), 4.47 – 4.36 (m, 1H, OC<u>H</u>$_a$H$_b$CH$_2$), 4.27 – 4.15 (m, 1H, OCH$_a$<u>H</u>$_b$CH$_2$), 3.97 (ddd, J = 11.3, 9.6, 8.2 Hz, 1H, NC<u>H</u>$_a$H$_b$CH$_2$), 3.81 (dq, J = 8.6, 7.0 Hz, 1H, OC<u>H</u>$_a$H$_b$CH$_3$), 3.69 – 3.53 (m, 5H, NCH$_a$<u>H</u>$_b$CH$_2$, OCH$_a$<u>H</u>$_b$CH$_2$,OC<u>H</u>$_3$), 2.63 – 2.45 (m, 2H, C<u>H</u>$_2$CO$_2$Me), 2.36 – 2.12 (m, 2H, COC<u>H</u>CH$_2$), 1.19 (t, J = 7.0 Hz, 3H, OCH$_2$C<u>H</u>$_3$).
^{13}C{^{1}H} NMR (101 MHz, $CDCl_3$) δ 199.4 (CO), 184.7 (C), 173.7 (C), 173.5 (C), 138.4 (C), 129.7 (CH), 128.5 (CH), 127.5 (CH), 98.1 (C), 94.0 (C), 79.9 (CH), 68.3 (CH_2), 66.2 (CH_2), 51.7 (CH_3), 49.0 (CH), 47.1 (CH_2), 30.9 (CH_2), 24.5 (CH_2), 15.2 (CH_3).

N-[(2S,3R)-Hexacarbonyl{μ-[η[4]-(2-azido-3-ethoxy-5-phenyl-4-pentynoyl)]dicobalt (Co-Co)}]-1,3-oxazolidine-2-thione (3q)

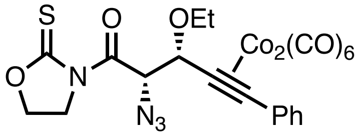

According to General Procedure E, the title compound was obtained from N-(α-azidoacetyl)-1,3- oxazolidine-2-thione (**1f**, 93 mg, 0.5 mmol, 1.0 equiv.) and hexacarbonyl μ-[η[4]-(1,1-diethoxy-3-phenylpropyne)dicobalt] (**2a**, 270 mg, 0.55 mmol, 1.1 equiv.) at 0 °C for 5 h. The analysis of the resultant oil by ^{1}H NMR established the formation of a single diastereomer.

The crude was purified by column chromatography (Hex/EtOAc, from 80:20 to 70:30) to afford 236 mg (0.37 mmol, 75% yield) of adduct **3q**.

Dark brown solid.
Mp 129–132 °C.
R$_f$ 0.3 (Hex/EtOAc, 70:30).
IR (ATR) ν 3063, 2977, 2926, 2115, 2091, 2050, 2002, 1702, 1376, 1327, 1167 cm^{-1}.
^{1}H NMR (400 MHz, CDCl$_3$) δ 7.52 – 7.43 (m, 2H, ArH), 7.39 – 7.27 (m, 3H, ArH), 6.01 (d, J = 4.7 Hz, 1H, COCHN$_3$), 5.46 (d, J = 4.7 Hz, 1H, CHOEt), 4.59 (td, J = 9.3, 6.3 Hz, 1H, OCH$_a$H$_b$CH$_2$), 4.46 (q, J = 9.0 Hz, 1H, OCH$_a$H$_b$CH$_2$), 4.30 – 4.19 (m, 1H, NCH$_a$H$_b$CH$_2$), 3.94 (ddd, J = 11.4, 9.3, 6.3 Hz, 1H, NCH$_a$H$_b$CH$_2$), 3.76 (dq, J = 8.6, 7.0 Hz, 1H, OCH$_a$H$_b$CH$_3$), 3.59 (dq, J = 8.7, 7.0 Hz, 1H, OCH$_a$H$_b$CH$_3$), 1.20 (t, J = 7.0 Hz, 3H, OCH$_2$CH$_3$).
^{13}C{^{1}H} NMR (101 MHz, CDCl$_3$) δ 199.2 (CO), 184.9 (C), 169.0 (C), 138.2 (C), 129.5 (CH), 128.8 (CH), 127.7 (CH), 93.7 (C), 93.5 (C), 80.6 (CH), 68.5 (CH$_2$), 67.0 (CH$_2$), 64.5 (CH), 47.4 (CH$_2$), 15.2 (CH$_3$).

N-[(2S,3S)-Hexacarbonyl{μ-[η⁴-(3-ethoxy-5-phenyl-2-pivaloylxy-4-pentynoyl)] dicobalt (Co-Co)}]-1,3-oxazolidine-2-thione (3r)

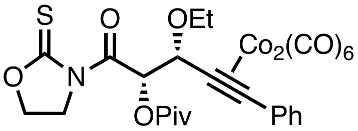

According to General Procedure E, the title compound was obtained from N-(α-pivaloyloxyacetyl)-1,3-oxazolidine-2-thione (**1g**, 61 mg, 0.25 mmol, 1.0 equiv.) and hexacarbonyl μ-[η^4-(1,1-diethoxy-3-phenylpropyne)dicobalt] (**2a**, 135 mg, 0.275 mmol, 1.1 equiv.), with a 5 mol% of [(R)-DTBM-SEGPHOS]$NiCl_2$ (16.36 mg, 12.5 μmol, 5 mol%) at 0 °C for 16 h. The analysis of the resultant oil by ^{1}H NMR established the formation of a single diastereomer.

The crude was purified by column chromatography (DCM/Hex/EtOAc, 50:48:2) to afford 139 mg (0.20 mmol, 81% yield) of adduct **3r**.

Dark brown solid.

Mp 121–124 °C.

R_f 0.3 (DCM/Hex/EtOAc, 50:48:2).

IR (ATR) ν 2974, 2924, 2894, 2868, 2091, 2050, 2033, 2013, 1731, 1711, 1476, 1381, 1364, 1329, 1169, 1131, 1090, 1068 cm^{-1}.

^{1}H NMR (400 MHz, $CDCl_3$) δ 7.78 – 7.70 (m, 2H, Ar<u>H</u>), 7.37 – 7.27 (m, 3H, Ar<u>H</u>), 7.08 (d, J = 2.3 Hz, 1H, COC<u>H</u>OPiv), 5.67 (d, J = 2.3 Hz, 1H, C<u>H</u>OEt), 4.70 – 4.54 (m, 2H, OC$\underline{H}_2$CH$_2$), 4.38 (ddd, J = 11.4, 9.8, 8.2 Hz, 1H, NC$\underline{H}_a$H$_b$CH$_2$), 4.11 (ddd, J = 11.4, 9.2, 6.8 Hz, 1H, NCH$_a\underline{H}_b$CH$_2$), 3.80 (dq, J = 8.8, 6.9 Hz, 1H, OC$\underline{H}_a$H$_b$CH$_3$), 3.53 (dq, J = 8.8, 6.9 Hz, 1H, OCH$_a\underline{H}_b$CH$_3$), 1.21 (t, J = 6.9 Hz, 3H, OCH$_2$C$\underline{H}_3$), 0.89 (s, 9H, OCOC(C$\underline{H}_3$)$_3$).

^{13}C{^{1}H} NMR (101 MHz, $CDCl_3$) δ 199.4 (CO), 185.0 (C), 178.8 (C), 168.1 (C), 137.7 (C), 130.6 (CH), 128.8 (CH), 128.0 (CH), 93.9 (C), 90.5 (C), 79.6 (CH), 75.3 (CH), 68.6 (CH_2), 67.2 (CH_2), 47.6 (CH_2), 38.7 (C), 26.6 (CH_3), 15.2 (CH_3).

5. Deprotection of Cobalted Adducts

General Procedure F for the decobaltation of the Nicholas adducts using CAN

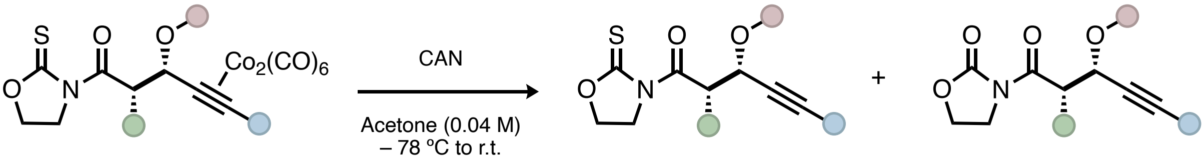

A solution of the corresponding cobalted aldol adduct (1.0 equiv.) and CAN (3.5 equiv.) in acetone (0.04 M) was stirred at – 78 °C for 30 min. Aftwerards, the cooling bath was removed and the reaction mixture was allowed to warm to r.t. for 2 h, at which time the cobalted product should still be observed on TLC (brown spot present). Then, additional CAN (0.25 – 0.5 equiv.) was added under N_2 and the solution was stirred for 2.5 min. The presence of starting material was then checked via TLC. If there was still cobalted alkyne, more CAN (0.25 – 0.5 equiv.) was added.

After consumption of the starting material, the reacting mixture was partitioned in diethyl ether (15 mL) and water (20 mL), and the aqueous layer was extracted with Et_2O (3 × 20 mL). The combined organic extracts were washed with brine (30 mL), dried with $MgSO_4$, filtered, and evaporated under reduced pressure. Finally, the crude was purified by column chromatography to yield the named compound.

***CAUTION**: A thorough monitoring of the reaction is required to prevent the undesired oxidation of the thiocarbonyl bond to its carbonyl counterpart, which was sometimes observed in the ^{1}H NMR of the crude mixture. The proportion of the two products is specified in each case.

N-[(2S,3S)-3-Ethoxy-2-methyl-5-phenyl-4-pentynoyl)]-1,3-oxazolidine-2-thione (4a)

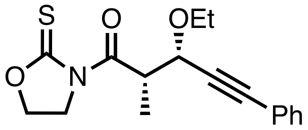

According to General Procedure F, the title compound was obtained from adduct **3a** (268 mg, 0.44 mmol, 1.0 equiv.) and CAN (844 mg, 1.54 mmol, 3.5 equiv.) in acetone (10 mL, 0.04 M) after 30 min at –78 °C and 2 h at r.t. A 95:5 mixture of **4a** and the oxidized derivative **5a** was observed in the ^{1}H NMR of the crude.

The crude was purified by column chromatography (Hex/EtOAc, from 80:20 to 60:40) to yield 100 mg (0.32 mmol, 71% yield) of pure adduct **4a**.

Colorless oil.
R$_f$ 0.4 (Hex/EtOAc, 75:25).
[α]$_D^{20}$ – 111 (c 0.8, $CHCl_3$).
IR (ATR) ν 3061, 2967, 2930, 2869, 2089, 1693, 1481, 1367, 1317, 1261, 1239, 1190, 1156, 1089, 1012 cm^{-1}.
^{1}H NMR (400 MHz, $CDCl_3$) δ 7.45 – 7.39 (m, 2H, Ar<u>H</u>), 7.34 – 7.27 (m, 3H, Ar<u>H</u>), 5.16 (dq, J = 7.5, 6.8 Hz, 1H, COC<u>H</u>CH_3), 4.58 (d, J = 7.5 Hz, 1H, C<u>H</u>OEt), 4.52 (td, J = 9.3, 6.2 Hz, 1H, OC<u>H</u>$_a$H$_b$$CH_2$), 4.36 (dt, J = 9.3, 8.8 Hz, 1H, OCH$_a$<u>H</u>$_b$$CH_2$), 4.27 – 4.12 (m, 2H, NC<u>H</u>$_2$$CH_2$), 3.85 (dq, J = 9.3, 7.0 Hz, 1H, OC<u>H</u>$_a$H$_b$$CH_3$), 3.54 (dq, J = 9.3, 7.0 Hz, 1H, OCH$_a$<u>H</u>$_b$$CH_3$), 1.40 (d, J = 6.8 Hz, 3H, COCHC<u>H</u>$_3$), 1.23 (t, J = 7.0 Hz, 3H, OCH_2C<u>H</u>$_3$).
^{13}C{^{1}H} NMR (101 MHz, $CDCl_3$) δ 185.5 (C), 175.3 (C), 132.0 (CH), 128.7 (CH), 128.4 (CH), 122.6 (C), 86.7 (C), 86.6 (C), 71.3 (CH), 66.5 (CH_2), 64.7 (CH_2), 47.6 (CH_2), 43.5 (CH), 15.2 (CH_3), 13.5 (CH_3).
HRMS (+ESI) m/z calcd. for [M – OEt]$^+$ $C_{15}H_{14}NO_2S$: 272.0740; found: 272.0738. m/z calcd. for [M + Na]$^+$ $C_{17}H_{19}NNaO_3S$: 340.0978, found: 340.0970.
Chiral HPLC (Phenomenex Lux® Cellulose-5 column, 5% iPrOH in hexanes, flow rate 1 mL/min) Rt 15.3 min (2S,3S) enantiomer, Rt 17.6 min (2R,3R) enantiomer, >99:1 e.r.

N-[(2S,3S)-3-Ethoxy-2-methyl-5-phenyl-4-pentynoyl]-1,3-oxazolidin-2-one (5a)

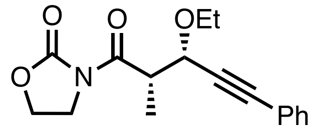

R$_f$ 0.3 (Hex/EtOAc, 75:25).
^{1}H NMR (400 MHz, $CDCl_3$) δ 7.46 – 7.39 (m, 2H, Ar<u>H</u>), 7.33 – 7.27 (m, 3H, Ar<u>H</u>), 4.49 (d, J = 7.7 Hz, 1H, C<u>H</u>OEt), 4.39 (td, J = 8.6, 7.0 Hz, 1H, OC<u>H</u>$_a$H$_b$$CH_2$), 4.34 – 4.25 (m, 2H, COC<u>H</u>CH_3, OCH$_a$<u>H</u>$_b$$CH_2$), 4.10 – 3.94 (m, 2H, NC<u>H</u>$_2$$CH_2$), 3.85 (dq, J = 9.3, 7.0 Hz, 1H, OC<u>H</u>$_a$H$_b$$CH_3$), 3.54 (dq, J = 9.3, 7.0 Hz, 1H, OCH$_a$<u>H</u>$_b$$CH_3$), 1.36 (d, J = 6.8 Hz, 3H, COCHC<u>H</u>$_3$), 1.23 (t, J = 7.0 Hz, 3H, OCH_2C<u>H</u>$_3$).
^{13}C{^{1}H} NMR (101 MHz, $CDCl_3$) δ 174.1 (C), 153.3 (C), 132.0 (CH), 128.6 (CH), 128.4 (CH), 122.7 (C), 86.7 (C), 86.5 (C), 71.1 (CH), 64.6 (CH_2), 62.0 (CH_2), 43.0 (CH), 43.0 (CH_2), 15.2 (CH_3), 13.6 (CH_3).

HRMS (+ESI) m/z calcd. for $[M + Na]^+$ $C_{17}H_{19}NNaO_4$: 324.1206, found: 324.1203. m/z calcd. for $[M – OEt]^+$ $C_{15}H_{14}NO_3$: 256.0968; found: 256.0967.

N-[(2S,3S)-3-Methoxy-2-methyl-5-phenyl-4-pentynoyl)]-1,3-oxazolidine-2-thione (4b)

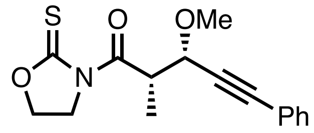

According to General Procedure F, the title compound was obtained from adduct **3b** (197 mg, 0.33 mmol, 1.0 equiv.) and CAN (625 mg, 1.14 mmol, 3.5 equiv.) in acetone (8 mL, 0.04 M) after 30 min at –78 °C and 2 h at r.t.

The crude was purified by column chromatography (Hex/EtOAc, 70:30) to yield 77 mg (0.25 mmol, 77% yield) of adduct **4b**.

Colorless oil.
R_f 0.2 (Hex/EtOAc, 75:25).
$[\alpha]_D^{25}$ – 119 (c 1.0, $CHCl_3$).
IR (ATR) ν 3055, 2984, 2930, 2876, 2824, 1696, 1489, 1362, 1318, 1187, 1154 cm^{-1}.
^{1}H NMR (400 MHz, $CDCl_3$) δ 7.47 – 7.40 (m, 2H, Ar<u>H</u>), 7.35 – 7.27 (m, 3H, Ar<u>H</u>), 5.17 (p, J = 6.8 Hz, 1H, COC<u>H</u>CH_3), 4.58 – 4.47 (m, 2H, C<u>H</u>COEt, OC<u>H</u>$_a$H$_b$CH$_2$), 4.39 (dt, J = 9.5, 8.7 Hz, 1H, OCH$_a$<u>H</u>$_b$CH$_2$), 4.28 – 4.13 (m, 2H, NC<u>H</u>$_2$CH$_2$), 3.47 (s, 3H, OC<u>H</u>$_3$), 1.40 (d, J = 6.8 Hz, 3H, COCHC<u>H</u>$_3$).
^{13}C{^{1}H} NMR (101 MHz, $CDCl_3$) δ 185.5 (C), 175.0 (C), 132.3 (CH), 128.8 (CH), 128.5 (CH), 122.5 (C), 87.2 (C), 85.9 (C), 73.0 (CH), 66.5 (CH_2), 56.8 (CH_3), 47.6 (CH_2), 43.3 (CH), 13.2 (CH_3).
HRMS (+ESI) m/z calcd. for $[M – OMe]^+$ $C_{15}H_{14}NO_2S$: 272.0740; found: 272.0736. m/z calcd. for $[M + H]^+$ $C_{16}H_{18}NO_3S$: 304.1002; found: 304.0997.
Chiral HPLC (Phenomenex Lux® Amylose-3 column, 5% iPrOH in hexanes, flow rate 1 mL/min, 271 nm) Rt 14.0 min (2S,3S) enantiomer, Rt 15.1 min (2R,3R) enantiomer, >99:1 e.r.

N-[(2S,3S)-3-Benzyloxy-2-methyl-5-phenyl-4-pentynoyl)]-1,3-oxazolidine-2-thione (4c)

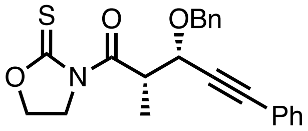

According to General Procedure F, the title compound was obtained from adduct **3c** (58 mg, 86 μmol, 1.0 equiv.) and CAN (166 mg, 0.30 mmol, 3.5 equiv.) in acetone (2 mL, 0.04 M) after 30 min at –78 °C and 45 min at r.t.

The crude was purified by column chromatography (Hex/EtOAc, 80:20 to 65:35) to yield 30 mg (78 μmol, 91% yield) of adduct **4c**.

Colorless oil.
R_f 0.2 (Hex/EtOAc, 80:20).
$[\alpha]_D^{25}$ – 161 (c 1.0, $CHCl_3$).
IR (ATR) ν 3062, 3028, 2924, 2853, 1700, 1489, 1454, 1370, 1318, 1191, 1157 cm^{-1}.
^{1}H NMR (400 MHz, $CDCl_3$) δ 7.53 – 7.41 (m, 2H, Ar<u>H</u>), 7.40 – 7.28 (m, 8H, Ar<u>H</u>), 5.25 – 5.12 (m, 1H, COC<u>H</u>CH_3), 4.84 (d, J = 12.0 Hz, 1H, OC$\underline{H}_a H_b$Ph), 4.71 (d, J = 6.5 Hz, 1H, C<u>H</u>OBn), 4.59 (d, J = 12.0 Hz, 1H, OC$H_a\underline{H}_b$Ph), 4.53 – 4.39 (m, 1H, OC$\underline{H}_a H_b CH_2$), 4.28 – 4.10 (m, 2H, OC$H_a\underline{H}_b CH_2$, NC$\underline{H}_a H_b CH_2$), 4.10 – 3.93 (m, 1H, NC$H_a\underline{H}_b CH_2$), 1.43 (d, J = 6.8 Hz, 3H, COCHC$\underline{H}_3$).
$^{13}C\{^1H\}$ NMR (101 MHz, $CDCl_3$) δ 185.4 (C), 174.8 (C), 137.8 (C), 132.0 (CH), 128.8 (CH), 128.5 (CH), 128.5 (CH), 128.3 (CH), 128.0 (CH), 122.5 (C), 87.1 (C), 86.1 (C), 70.7 (CH_2), 70.2 (CH), 66.4 (CH_2), 47.5 (CH_2), 43.5 (CH), 12.8 (CH_3).
HRMS (+ESI) m/z calcd. for $[M – OBn]^+$ $C_{15}H_{14}NO_2S$: 272.0740; found: 272.0745. m/z calcd. for $[M + H]^+$ $C_{22}H_{22}NO_3S$: 380.1315; found: 380.1321.
Chiral HPLC (Phenomenex Lux® Cellulose-5 column, 5% iPrOH in hexanes, flow rate 1 mL/min) Rt 19.6 min (2S,3S) enantiomer, Rt 24.4 min (2R,3R) enantiomer, >99:1 e.r.

N-[(2S,3S)-5-tert-Butyldimethylsilyl-3-ethoxy-2-methyl-4-pentynoyl]-1,3-oxazolidine-2-thione (4d)

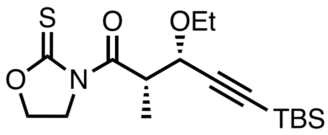

According to General Procedure F, the title compound was obtained from adduct **3d** (281 mg, 0.44 mmol, 1.0 equiv.) and CAN (961 mg, 1.75 mmol, 4.0 equiv.) in acetone (10 mL, 0.04 M) after 30 min at –78 °C and 2 h at r.t. A 92:8 mixture of **4d** and the oxidized derivative **5d** was observed in the ^{1}H NMR of the crude.

The crude was purified by column chromatography (Hex/EtOAc, 90:10 to 65:35) to yield 138 mg (0.39 mmol, 89% yield) of adduct adduct **4d**.

White solid.
Mp 42–43 °C.
R_f 0.3 (Hex/EtOAc, 80:20).
$[\alpha]_D^{20}$ – 60 (c 1.0, $CHCl_3$).
IR (ATR) ν 2952, 2928, 2885, 2857, 2169, 1699, 1470, 1374, 1351, 1318, 1295, 1249, 1211, 1189, 1164, 1136, 1118, 1094, 1008 cm^{-1}.
^{1}H NMR (500 MHz, $CDCl_3$) δ 5.03 (dq, J = 7.6, 6.9 Hz, 1H, COC<u>H</u>CH$_3$), 4.55 (td, J = 9.2, 6.3 Hz, 1H, OC<u>H</u>$_a$H$_b$CH$_2$), 4.46 (q, J = 9.0 Hz, 1H, OCH$_a$<u>H</u>$_b$CH$_2$), 4.38 (d, J = 7.6 Hz, 1H, C<u>H</u>OEt), 4.28 – 4.10 (m, 2H, NC<u>H</u>$_2$CH$_2$), 3.76 (dq, J = 9.4, 7.0 Hz, 1H, OC<u>H</u>$_a$H$_b$CH$_3$), 3.46 (dq, J = 9.4, 7.0 Hz, 1H, OCH$_a$<u>H</u>$_b$CH$_3$), 1.33 (d, J = 6.9 Hz, 3H, COCHC<u>H</u>$_3$), 1.19 (t, J = 7.0 Hz, 3H, OCH$_2$C<u>H</u>$_3$), 0.91 (s, 9H, SiC(C<u>H</u>$_3$)$_3$), 0.08 (s, 6H, Si(C<u>H</u>$_3$)$_2$).
^{13}C{^{1}H} NMR (126 MHz, $CDCl_3$) δ 185.3 (C), 175.1 (C), 103.7 (C), 89.6 (C), 70.9 (CH), 66.3 (CH_2), 64.4 (CH_2), 47.5 (CH_2), 43.5 (CH), 26.1 (CH_3), 16.6 (C), 15.1 (CH_3), 13.4 (CH_3), -4.6 (CH_3).
HRMS (+ESI) m/z calcd. for $[M – OEt]^+$ $C_{15}H_{24}NO_2SSi$: 310.1292; found: 310.1300. m/z calcd. for $[M + Na]^+$ $C_{17}H_{29}NNaO_3SSi$: 378.1530; found: 378.1553.
Chiral HPLC (Phenomenex Lux® Cellulose-5 column, 5% iPrOH in hexanes, flow rate 1 mL/min) Rt 7.4 min (2S,3S) enantiomer, Rt 9.7 min (2R,3R) enantiomer, > 99:1 e.r.

N-[(2S,3S)-5-tert-Butyldimethylsilyl-3-ethoxy-2-methyl-4-pentynoyl]-1,3-oxazolidin-2-one (5d)

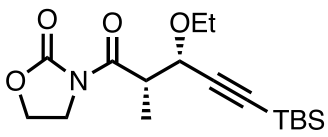

^{1}H NMR (400 MHz, $CDCl_3$) δ 4.46 – 4.31 (m, 2H), 4.29 (d, J = 7.9 Hz, 1H), 4.19 (dq, J = 7.9, 6.7 Hz, 1H), 4.10 – 3.93 (m, 2H), 3.78 (dq, J = 9.3, 7.0 Hz, 1H), 3.47 (dq, J = 9.3, 7.0 Hz, 1H), 1.30 (d, J = 6.7 Hz, 3H), 1.20 (t, J = 7.0 Hz, 3H), 0.92 (s, 9H), 0.09 (s, 6H).
HRMS (+ESI) m/z calcd. for $[M + Na]^+$ $C_{17}H_{29}NNaO_4Si$: 362.1758; found: 362.1755. m/z calcd. for $[M + H]^+$ $C_{17}H_{30}NO_4Si$: 340.1939; found: 340.1936. m/z calcd. for $[M – OEt]^+$ $C_{15}H_{24}NO_3Si$: 294.1520; found: 294.1517.

N-[(2S,3S)-3-Ethoxy-2-methyl-4-nonynoyl]-1,3-oxazolidine-2-thione (4e)

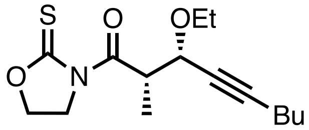

According to General Procedure F, the title compound was obtained from adduct **3e** (55 mg, 94 µmol, 1.0 equiv.) and CAN (181 mg, 330 µmol, 3.5 equiv.) in acetone (2.3 mL, 0.04 M) after 30 min at –78 °C and 1 h 20 min at r.t.

The crude was purified by column chromatography (Hex/EtOAc, 85:15 to 70:30) to yield 24 mg (79 µmol, 84% yield) of adduct **4e**.

Colorless oil.
R_f 0.4 (Hex/EtOAc, 70:30).
$[\alpha]_D^{25}$ – 29 (c 1.0, $CHCl_3$).
IR (ATR) ν 2958, 2928, 2872, 1700, 1457, 1368, 1320, 1206, 1161, 1090 cm^{-1}.
^{1}H NMR (500 MHz, $CDCl_3$) δ 5.04 – 4.93 (m, 1H, COC$\underline{H}$CH$_3$), 4.56 (td, J = 9.1, 6.3 Hz, 1H, OC$\underline{H}_a$H$_b$CH$_2$), 4.47 (q, J = 9.1 Hz, 1H, OCH$_a\underline{H}_b$CH$_2$), 4.37 (dt, J = 7.3, 2.0 Hz, 1H, C$\underline{H}$OEt), 4.28 – 4.13 (m, 2H, NC$\underline{H}_2$CH$_2$), 3.75 (dq, J = 9.4, 7.0 Hz, 1H, OC$\underline{H}_a$H$_b$CH$_3$), 3.44 (dq, J = 9.4, 7.0 Hz, 1H, OCH$_a\underline{H}_b$CH$_3$), 2.20 (td, J = 7.0, 2.0 Hz, 2H, C≡CC$\underline{H}_2$), 1.51 – 1.43 (m, 2H, C≡CCH$_2$C$\underline{H}_2$), 1.43 – 1.36 (m, 2H, CH$_2$C$\underline{H}_2$CH$_3$), 1.33 (d, J = 6.9 Hz, 3H, COCHC$\underline{H}_3$), 1.19 (t, J = 7.0 Hz, 3H, OCH$_2$C$\underline{H}_3$), 0.90 (t, J = 7.3 Hz, 3H, CH$_2$CH$_2$C$\underline{H}_3$).

$^{13}C\{^1H\}$ NMR (126 MHz, $CDCl_3$) δ 185.4 (C), 175.4 (C), 87.2 (C), 77.5 (C), 70.8 (CH), 66.4 (CH_2), 64.3 (CH_2), 47.5 (CH_2), 43.7 (CH), 30.8 (CH_2), 22.0 (CH_2), 18.5 (CH_2), 15.2 (CH_3), 13.7 (CH_3), 13.4 (CH_3).

HRMS (+ESI) m/z calcd. for $[M – OEt]^+$ $C_{13}H_{18}NO_2S$: 252.1053; found: 252.1047. m/z calcd. for $[M + Na]^+$ $C_{15}N_{23}NNaO_3S$: 320.1291; found: 320.1287.

Chiral HPLC (Phenomenex Lux® Cellulose-5 column, 5% iPrOH in hexanes, flow rate 1 mL/min) Rt 13.3 min (2S,3S) enantiomer, Rt 14.8 min (2R,3R) enantiomer, > 99:1 e.r.

N-[(2S,3S)-3-Ethoxy-2-methyl-4-oct-7-en-ynoyl]-1,3-oxazolidine-2-thione (4f)

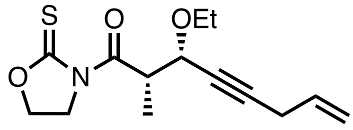

According to General Procedure F, the title compound was obtained from adduct **3f** (267 mg, 0.47 mmol, 1.00 equiv.) and CAN (1.03 g, 1.88 mmol, 4.0 equiv.) in acetone (12 mL, 0.04 M) after 30 min at –78 °C and 2 h and 2.5 min at r.t.

The crude was purified by column chromatography (Hex/EtOAc, 80:20 to 60:40) to yield 112 mg (0.40 mmol, 84% yield) of adduct **4f**.

Colorless oil.

R_f 0.3 (Hex/EtOAc, 70:30).

$[\alpha]_D^{25}$ – 58 (c 1.0, $CHCl_3$).

IR (ATR) v 2974, 2917, 2876, 1696, 1642, 1472, 1456, 1366, 1318, 1262, 1191, 1157, 1088, 1012 cm^{-1}.

^1H NMR (400 MHz, $CDCl_3$) δ 5.79 (ddt, J = 17.0, 10.1, 5.1 Hz, 1H, $CH_2=C\underline{H}CH_2$), 5.29 (dq, J = 17.0, 1.8 Hz, 1H, $C\underline{H_a}H_b=CH$), 5.09 (dq, J = 10.1, 1.8 Hz, 1H, $CH_a\underline{H_b}=CH$), 5.07 – 4.94 (m, 1H, $COC\underline{H}CH_3$), 4.54 (td, J = 9.1, 6.4 Hz, 1H, $OC\underline{H_a}H_bCH_2$), 4.45 (q, J = 9.1 Hz, 1H, $OCH_a\underline{H_b}CH_2$), 4.39 (dt, J = 7.4, 2.0 Hz, 1H, $C\underline{H}OEt$), 4.26 – 4.11 (m, 2H, $NC\underline{H_2}CH_2$), 3.76 (dq, J = 9.2, 7.0 Hz, 1H, $OC\underline{H_a}H_bCH_3$), 3.45 (dq, J = 9.2, 7.0 Hz, 1H, $OCH_a\underline{H_b}CH_3$), 2.98 (dq, J = 5.1, 2.0 Hz, 2H, $C\equiv CC\underline{H_2}CH=CH_2$), 1.33 (d, J = 6.9 Hz, 3H, $COCHC\underline{H_3}$), 1.18 (t, J = 7.0 Hz, 3H, $OCH_2C\underline{H_3}$).

$^{13}C\{^1H\}$ NMR (101 MHz, $CDCl_3$) δ 185.4 (C), 175.4 (C), 132.2 (CH), 115.9 (CH_2), 83.5 (C), 80.2 (C), 70.8 (CH), 66.6 (CH_2), 64.4 (CH_2), 47.5 (CH_2), 43.6 (CH), 23.1 (CH_2), 15.1 (CH_3), 13.4 (CH_3).

HRMS (+ESI) m/z calcd. for [M + Na]+ $C_{14}H_{19}NNaO_3S$: 304.0978; found: 304.0976.

Chiral HPLC (Phenomenex Lux® Cellulose-5 column, 5% iPrOH in hexanes, flow rate 1 mL/min) Rt 15.7 min (2S,3S) enantiomer, Rt 17.0 min (2R,3R) enantiomer, >99:1 e.r.

N-[(2S,3S)-8-Bromo-3-ethoxy-2-methyl-4-octynoyl]-1,3-oxazolidine-2-thione (4g)

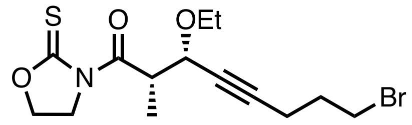

According to General Procedure F, the title compound was obtained from adduct **3g** (202 mg, 0.31 mmol, 1.00 equiv.) and CAN (640 mg, 1.16 mmol, 3.75 equiv.) in acetone (8 mL, 0.04 M) after 30 min at –78 °C and 2 h and 5 min at r.t.

The crude was purified by column chromatography (Hex/EtOAc, 80:20 to 60:40) to yield 106 mg (0.29 mmol, 94% yield) of adduct **4g**.

Colorless oil.
R$_f$ 0.3 (Hex/EtOAc, 70:30).
[α]$_D^{20}$ – 56 (c 0.5, $CHCl_3$).
IR (ATR) ν 2973, 2930, 2872, 1696, 1475, 1367, 1318, 1261, 1191, 1158, 1088 cm^{-1}.
^{1}H NMR (400 MHz, $CDCl_3$) δ 5.03 (dq, J = 7.6, 6.8 Hz, 1H, COC<u>H</u>CH_3), 4.61 – 4.44 (m, 2H, OC<u>H</u>$_2CH_2$), 4.35 (dt, J = 7.6, 1.9 Hz, 1H, C<u>H</u>OEt), 4.22 (t, J = 8.5 Hz, 2H, NC<u>H</u>$_2CH_2$), 3.75 (dq, J = 9.3, 7.0 Hz, 1H, OC<u>H</u>$_aH_bCH_3$), 3.51 (t, J = 6.7 Hz, 2H, CH_2C<u>H</u>$_2$Br), 3.44 (dq, J = 9.3, 7.0 Hz, 1H, OCH_a<u>H</u>$_bCH_3$), 2.41 (td, J = 6.7, 1.9 Hz, 2H, C≡CC<u>H</u>$_2$), 2.01 (p, J = 6.7 Hz, 2H, C≡CCH_2C<u>H</u>$_2$), 1.33 (d, J = 6.8 Hz, 3H, COCHC<u>H</u>$_3$), 1.19 (t, J = 7.0 Hz, 3H, OCH_2C<u>H</u>$_3$).
^{13}C{^{1}H} NMR (101 MHz, $CDCl_3$) δ 185.4 (C), 175.3 (C), 84.8 (C), 79.0 (C), 70.7 (CH), 66.4 (CH_2), 64.5 (CH_2), 47.5 (CH_2), 43.5 (CH), 32.6 (CH_2), 31.3 (CH_2) , 17.5 (CH_2), 15.1 (CH_3), 13.64 (CH_3).
HRMS (+ESI) m/z calcd. for $[M + Na]^+$ $C_{14}H_{20}BrNNaO_3S$: 384.0239, found: 384.0238.
Chiral HPLC (Phenomenex Lux® Cellulose-5 column, 5% iPrOH in hexanes, flow rate 1 mL/min) Rt 17.6 min (2S,3S) enantiomer, Rt 19.8 min (2R,3R) enantiomer, >99:1 e.r.

N-[(2S,3S)-8-Azido-3-ethoxy-2-methyl-4-octynoyl]-1,3-oxazolidine-2-thione (4h)

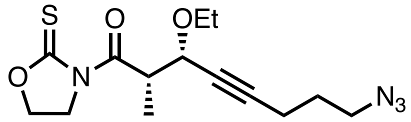

According to General Procedure F, the title compound was obtained from adduct **3h** (239 mg, 0.39 mmol, 1.0 equiv.) and CAN (751 mg, 1.37 mmol, 3.5 equiv.) in acetone (7.6 mL, 0.04 M) after 30 min at –78 °C and 2 h at r.t. An 86:14 mixture of **4h** and the oxidized derivative **5h** was observed in the ^{1}H NMR of the crude.

The crude was purified by column chromatography (Hex/EtOAc, 80:20 to 55:45) to yield 107 mg (0.33 mmol, 85% yield) of adduct **4h**.

Colorless oil.
R_f 0.20 (Hex/EtOAc, 80:20).
$[\alpha]_D^{25}$ – 66 (c 1.0, $CHCl_3$).
IR (ATR) ν 2974, 2928, 2874, 2095, 1697, 1472, 1452, 1368, 1320, 1191, 1157 cm^{-1}.
^{1}H NMR (400 MHz, $CDCl_3$) δ 5.06 – 4.94 (m, 1H, COC$\underline{H}$CH_3), 4.60 – 4.43 (m, 2H, OC$\underline{H}_2$CH_2), 4.35 (dt, J = 7.2, 1.9 Hz, 1H, C$\underline{H}$OEt), 4.28 – 4.12 (m, 2H, NC$\underline{H}_2$CH_2), 3.74 (dq, J = 9.3, 7.0 Hz, 1H, OC$\underline{H}_a$$H_bCH_3$), 3.42 (dq, J = 9.3, 7.0 Hz, 1H, OC$H_a$$\underline{H}_bCH_3$), 3.39 (t, J = 6.5 Hz, 2H, CH_2C$\underline{H}_2$$N_3$), 2.32 (td, J = 6.8, 1.9 Hz, 2H, C≡CC$\underline{H}_2$), 1.75 (p, J = 6.8 Hz, 2H, C≡CCH_2C$\underline{H}_2$), 1.31 (d, J = 6.8 Hz, 3H, COCHC$\underline{H}_3$), 1.18 (t, J = 7.0 Hz, 3H, OCH_2C$\underline{H}_3$).
^{13}C{^{1}H} NMR (101 MHz, $CDCl_3$) δ 185.4 (C), 175.2 (C), 85.1 (C), 78.9 (C), 70.7 (CH), 66.4 (CH_2), 64.5 (CH_2), 50.2 (CH_2), 47.5 (CH_2), 43.4 (CH), 27.9 (CH_2), 16.1 (CH_2), 15.1 (CH_3), 13.5 (CH_3).
HRMS (+ESI) m/z calcd. for $[M – OEt]^+$ $C_{12}H_{15}N_4O_2S$: 279.0910; found: 279.0911. m/z calcd. for $[M + H]^+$ $C_{14}H_{21}N_4O_3S$: 325.1329; found: 325.1329.
Chiral HPLC (Phenomenex Lux® Cellulose-5 column, 5% iPrOH in hexanes, flow rate 1 mL/min) Rt 22.7 min (2S,3S) enantiomer, Rt 25.5 min (2R,3R) enantiomer, 99:1 e.r.

N-[(2S,3S)-8-Azido-3-ethoxy-2-methyl-4-octynoyl]-1,3-oxazolidin-2-one (5h)

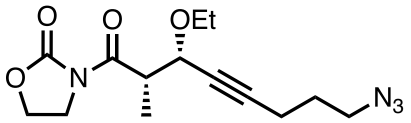

^{1}H NMR (400 MHz, CDCl$_3$) δ 4.44 – 4.37 (m, 2H), 4.27 (dt, J = 7.5, 1.9 Hz, 1H), 4.20 – 4.11 (m, 1H), 4.06 – 3.99 (m, 2H), 3.75 (dq, J = 9.3, 7.1 Hz, 1H), 3.49 – 3.36 (m, 3H), 2.33 (td, J = 6.8, 1.9 Hz, 2H), 1.76 (p, J = 6.7 Hz, 2H), 1.28 (d, J = 6.8 Hz, 3H), 1.19 (t, J = 7.1 Hz, 3H).
HRMS (+ESI) m/z calcd. for $[M + Na]^+$ $C_{14}H_{20}N_4NaO_4$: 331.1377; found: 331.1375. m/z calcd. for $[M – OEt – N_2]^+$ $C_{12}H_{15}N_2O_3$: 235.1077; found: 235.1076.

N-[(2S,3S)-8-Acetyloxy-3-ethoxy-2-methyl-4-octynoyl]-1,3-oxazolidine-2-thione (4i)

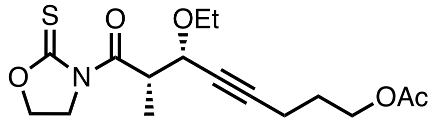

According to General Procedure F, the title compound was obtained from adduct **3i** (310 mg, 0.49 mmol, 1.00 equiv.) and CAN (1.097 mg, 2.00 mmol, 4.00 equiv.) in acetone (12 mL, 0.04 M) after 30 min at –78 °C and 2 h at r.t.

The crude was purified by column chromatography (DCM/Hex/EtOAc, 50:40:10) to yield 155 mg (0.45 mmol, 92% yield) of adduct **4i**.

Colorless oil.
R$_f$ 0.3 (DCM/Hex/EtOAc, 50:40:10).
$[\alpha]_D^{25}$ – 61 (c 1.0, CHCl$_3$).
IR (ATR) ν 2973, 2928, 2874, 1735, 1698, 1472, 1456, 1366, 1318, 1239, 1191, 1157, 1088, 1040 cm^{-1}.
^{1}H NMR (400 MHz, CDCl$_3$) δ 5.07 – 4.96 (m, 1H, COC$\underline{H}$CH$_3$), 4.60 – 4.46 (m, 2H, OC$\underline{H}_2$CH$_2$), 4.36 (dt, J = 7.4, 1.9 Hz, 1H, C$\underline{H}$OEt), 4.26 – 4.18 (m, 2H, NC$\underline{H}_2$CH$_2$), 4.11 (td, J = 6.4, 1.1 Hz, 2H, CH$_2$C$\underline{H}_2$OAc), 3.75 (dq, J = 9.4, 7.0 Hz, 1H, OC$\underline{H}_a$H$_b$CH$_3$), 3.43 (dq, J = 9.4, 7.0 Hz, 1H, OCH$_a\underline{H}_b$CH$_3$), 2.31 (td, J = 7.0, 1.9 Hz, 2H, C≡CC$\underline{H}_2$), 2.04 (s, 3H, COC$\underline{H}_3$), 1.91 – 1.77 (m, 2H, C≡CCH$_2$C$\underline{H}_2$), 1.32 (d, J = 6.9 Hz, 3H, COCHC$\underline{H}_3$), 1.19 (t, J = 7.0 Hz, 3H).
^{13}C{^{1}H} NMR (101 MHz, CDCl$_3$) δ 185.3 (C), 175.3 (C), 171.2 (C), 85.5 (C), 78.5 (C), 70.6 (CH), 66.4 (CH$_2$), 64.4 (CH$_2$), 63.1 (CH$_2$), 47.5 (CH$_2$), 43.5 (CH), 27.7 (CH$_2$), 21.1 (CH$_3$), 15.6 (CH$_2$), 15.1 (CH$_3$), 13.6 (CH$_3$).
HRMS (+ESI) m/z calcd. for $[M – OEt]^+$ $C_{15}H_{24}NO_2S$: 296.0951; found: 296.0950 .m/z calcd. for $[M + Na]^+$ $C_{16}H_{23}O_5NNaS$: 364.1189; found: 364.1189.

Chiral HPLC (Phenomenex Lux® Cellulose-5 column, 5% iPrOH in hexanes, flow rate 1 mL/min, 271 nm) Rt 51.4 min (2S,3S) enantiomer, Rt 54.8 min (2R,3R) enantiomer, 99:1 e.r.

N-[(2S,3S)-3-Ethoxy-2-methyl-8-pivaloyloxy-4-octynoyl]-1,3-oxazolidine-2-thione (4j)

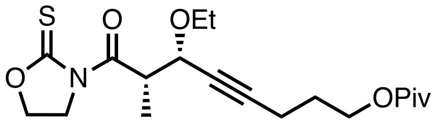

According to General Procedure F, the title compound was obtained from adduct **3j** (314 mg, 0.47 mmol, 1.00 equiv.) and CAN (1.03 g, 1.33 mmol, 4.0 equiv.) in acetone (12 mL, 0.04 M) after 30 min at –78 °C and 2 h and 5 min at r.t. An 87:13 mixture of **4j** and the oxidized derivative **5j** was observed in the ^{1}H NMR of the crude.

The crude was purified by column chromatography (Hex/EtOAc, 80:20 to 55:45) to yield 150 mg (0.39 mmol, 83% yield) of adduct **4j**.
Colorless oil
R_f 0.3 (Hex/EtOAc, 75:25).
$[\alpha]_D^{20}$ – 52 (c 1.0, $CHCl_3$).
IR (ATR) ν 2973, 2933, 2872, 1720, 1698, 1479, 1457, 1366, 1319, 1284, 1261, 1193, 1151, 1090 cm^{-1}.
^{1}H NMR (500 MHz, $CDCl_3$) δ 5.05 – 4.96 (m, 1H, COC<u>H</u>CH_3), 4.59 – 4.47 (m, 2H, OC<u>H</u>$_2$$CH_2$), 4.36 (dt, J = 7.4, 1.9 Hz, 1H, C<u>H</u>OEt), 4.28 – 4.16 (m, 2H, NC<u>H</u>$_2$$CH_2$), 4.09 (t, J = 6.3 Hz, 2H, CH_2C<u>H</u>$_2$OPiv), 3.75 (dq, J = 9.3, 7.1 Hz, 1H, OC<u>H</u>$_a$H$_b$$CH_3$), 3.43 (dq, J = 9.3, 7.1 Hz, 1H, OCH$_a$<u>H</u>$_b$$CH_3$), 2.30 (td, J = 7.0, 1.9 Hz, 2H, C≡CC<u>H</u>$_2$), 1.86 – 1.78 (m, 2H, C≡CCH_2C<u>H</u>$_2$), 1.32 (d, J = 6.8 Hz, 3H, COCHC<u>H</u>$_3$), 1.18 (t, J = 7.1 Hz, 3H, OCH_2C<u>H</u>$_3$), 1.18 (s, 9H, C(C<u>H</u>$_3$)$_3$).
^{13}C{^{1}H} NMR (126 MHz, $CDCl_3$) δ 185.4 (C), 178.6 (C), 175.3 (C), 85.5 (C), 78.5 (C), 70.6 (CH), 66.4 (CH_2), 64.4 (CH_2), 63.0 (CH_2), 47.5 (CH_2), 43.5 (CH), 38.9 (C), 27.8 (CH_2), 27.3 (CH_3), 15.6 (CH_2), 15.1 (CH_3), 13.6 (CH_3).
HRMS (+ESI) m/z calcd. for [M – OEt]$^+$ $C_{17}H_{24}NO_4S$: 338.1421; found: 338.1427. m/z calcd. for [M + Na]$^+$ $C_{19}H_{29}NNaO_5S$: 406.1659, found: 406.1665.
Chiral HPLC (Phenomenex Lux® Cellulose-5 column, 5% iPrOH in hexanes, flow rate 1 mL/min) Rt 22.9 min (2S,3S) enantiomer, Rt 24.4 min (2R,3R) enantiomer, 99:1 e.r.

N-[(2S,3S)-3-Ethoxy-2-methyl-8-pivaloyloxy-4-octynoyl]-1,3-oxazolidin-2-one (5j)

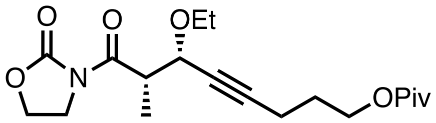

^{1}H NMR (400 MHz, CDCl$_3$) δ 4.49 – 4.37 (m, 2H), 4.26 (dt, J = 7.7, 1.9 Hz, 1H), 4.19 – 3.96 (m, 5H), 3.75 (dq, J = 9.2, 7.1 Hz, 1H), 3.44 (dq, J = 9.2, 7.1 Hz, 1H), 2.30 (td, J = 7.0, 1.9 Hz, 2H), 1.87 – 1.79 (m, 2H), 1.28 (d, J = 6.8 Hz, 3H), 1.19 (t, J = 7.0 Hz, 3 H), 1.19 (s, 9H).
HRMS (+ESI) m/z calcd. for $[M + Na]^+$ $C_{19}H_{29}NNaO_6$: 390.1887, found: 390.1885. m/z calcd. for $[M – OEt]^+$ $C_{17}H_{24}NO_5$: 322.1649; found: 322.1648.

N-[(2S,3S)-8-(tert-Butyldiphenylsilyl)oxy-3-ethoxy-2-methyl-4-octynoyl]-1,3-oxazolidine-2-thione (4k)

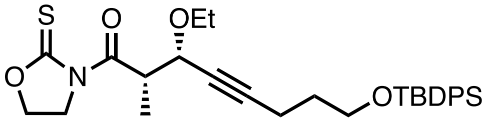

According to General Procedure F, the title compound was obtained from adduct **3k** (265 mg, 0.32 mmol, 1.00 equiv.) and CAN (679 mg, 1.24 mmol, 3.85 equiv.) in acetone (8 mL, 0.04 M) after 30 min at –78 °C and 2 h at r.t.

The crude was purified by column chromatography (Hex/EtOAc, 90:10 to 70:30) to yield 159 mg (0.30 mmol, 92% yield) of adduct **4k**.

Colorless oil.
R$_f$ 0.3 (Hex/EtOAc, 80:20).
$[\alpha]_D^{20}$ – 34 (c 1.0, CHCl$_3$).
IR (ATR) ν 2956, 2928, 2857, 1699, 1589, 1472, 1428, 1369, 1319, 1261, 1189, 1157, 1105, 1010 cm^{-1}.
^{1}H NMR (500 MHz, CDCl$_3$) δ 7.69 – 7.62 (m, 4H, Ar$\underline{H}$), 7.46 – 7.34 (m, 6H, Ar$\underline{H}$), 5.02 – 4.91 (m, 1H, COC$\underline{H}$CH$_3$), 4.50 (td, J = 9.2, 6.2 Hz, 1H, OC$\underline{H}_a$H$_b$CH$_2$), 4.41 (q, J = 9.2 Hz, 1H, OCH$_a\underline{H}_b$CH$_2$), 4.38 (dt, J = 7.3, 2.0 Hz, 1H, C$\underline{H}$OEt), 4.24 – 4.07 (m, 2H, NC$\underline{H}_2$CH$_2$), 3.72 (m, 3H, OC$\underline{H}_a$H$_b$CH$_3$, CH$_2$C$\underline{H}_2$OTBDPS), 3.40 (dq, J = 9.4, 7.0 Hz, 1H, OCH$_a\underline{H}_b$CH$_3$), 2.36 (td, J = 7.2, 2.0 Hz, 2H, C≡CC$\underline{H}_2$), 1.79 – 1.71 (m, 2H, C≡CCH$_2$C$\underline{H}_2$), 1.32 (d, J = 6.8 Hz, 3H, COCHC$\underline{H}_3$), 1.16 (t, J = 7.0 Hz, 3H, OCH$_2$C$\underline{H}_3$), 1.05 (s, 9H, SiC(C$\underline{H}_3$)).

$^{13}C\{^1H\}$ NMR (126 MHz, CDCl$_3$) δ 185.4 (C), 175.3 (C), 135.6 (CH), 133.9 (C), 129.7 (CH), 127.8 (CH), 86.8 (C), 77.6 (C), 70.7 (CH), 66.4 (CH_2), 64.3 (CH_2), 62.6 (CH_2), 47.2 (CH_2), 43.6 (CH), 31.8 (CH_2), 27.0 (CH_3), 19.4 (C), 15.5 (CH_2), 15.1 (CH_3), 13.3 (CH_3).
HRMS (+ESI) m/z calcd. for $[M – OEt]^+$ $C_{15}H_{24}NO_2SSi$: 492.2023; found: 492.2033. m/z calcd. for $[M + Na]^+$ $C_{30}H_{39}NNaO_4SSi$: 560.2261, found: 560.2272.
Chiral HPLC (Phenomenex Lux® Cellulose-5 column, 5% iPrOH in hexanes, flow rate 1 mL/min) Rt 9.6 min (2S,3S) enantiomer, Rt 10.8 min (2R,3R) enantiomer, > 99:1 e.r.

N-[(2S,3S)-3-Ethoxy-8-methoxy-2-methyl-4-octynoyl]-1,3-oxazolidine-2-thione (4l)

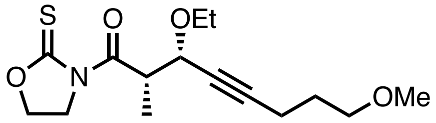

According to General Procedure F, the title compound was obtained from adduct **3l** (279 mg, 0.47 mmol, 1.00 equiv.) and CAN (1.02 g, 1.86 mmol, 4.0 equiv.) in acetone (12 mL, 0.04 M) after 30 min at –78 °C and 2 h and 2.5 min at r.t. A 95:5 mixture of **4l** and the oxidized derivative **5l** was observed in the ^{1}H NMR of the crude.

The crude was purified by column chromatography (Hex/EtOAc, 80:20 to 60:40) to yield 133 mg (0.42 mmol, 91% yield) of adduct **4l**.

Colorless oil.
R_f 0.3 (Hex/EtOAc, 70:30).
$[\alpha]_D^{20}$ – 60 (c 1.0, CHCl$_3$).
IR (ATR) ν 2961, 2924, 2872, 1698, 1456, 1366, 1318, 1260, 1189, 1157, 1112, 1088, 1074, 1012 cm^{-1}.
^{1}H NMR (400 MHz, CDCl$_3$) δ 5.04 – 4.92 (m, 1H, $COC\underline{H}CH_3$), 4.60 – 4.51 (m, 1H, $OC\underline{H}_aH_bCH_2$), 4.48 (q, J = 8.9 Hz, 1 H, $OCH_a\underline{H}_bCH_2$) 4.36 (dt, J = 7.3, 1.9 Hz, 1H, $C\underline{H}OEt$), 4.29 – 4.12 (m, 2H, $NC\underline{H}_2CH_2$), 3.75 (dq, J = 9.3, 7.0 Hz, 1H, $OC\underline{H}_aH_bCH_3$), 3.50 – 3.37 (m, 3H, $OCH_a\underline{H}_bCH_3,CH_2C\underline{H}_2OMe$), 3.32 (s, 3H, $OC\underline{H}_3$), 2.28 (td, J = 7.0, 2.0 Hz, 2H, $C{\equiv}CC\underline{H}_2$), 1.79 – 1.68 (m, 2H, $C{\equiv}CCH_2C\underline{H}_2$), 1.32 (d, J = 6.8 Hz, 3H, $COCHC\underline{H}_3$), 1.18 (t, J = 7.0 Hz, 3H, $OCH_2C\underline{H}_3$).
$^{13}C\{^1H\}$ NMR (101 MHz, CDCl$_3$) δ 185.4 (C), 175.4 (C), 86.4 (C), 77.9 (C), 71.2 (CH_2), 70.7 (CH), 66.4 (CH_2), 64.3 (CH_2), 58.7 (CH_3), 47.5 (CH_2), 43.6 (CH), 28.8 (CH_2), 15.6 (CH_2), 15.1 (CH_3), 13.4 (CH_3).

HRMS (+ESI) m/z calcd. for $[M - OEt]^+$ $C_{13}H_{18}NO_3S$: 268.1002; found: 268.1005. m/z calcd. for $[M + Na]^+$ $C_{15}H_{23}NNaO_4S$: 336.1240, found: 336.1247.
Chiral HPLC (Phenomenex Lux® Cellulose-5 column, 5% iPrOH in hexanes, flow rate 1 mL/min) Rt 23.2 min (2S,3S) enantiomer, Rt 24.9 min (2R,3R) enantiomer, >99:1 e.r.

N-[(2S,3S)-3-Ethoxy-8-methoxy-2-methyl-4-octynoyl]-1,3-oxazolidin-2-one (5l)

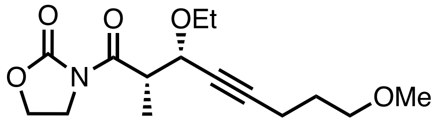

^{1}H NMR (400 MHz, $CDCl_3$) δ 4.43 – 4.37 (m, 2H), 4.27 (dt, J = 7.5, 2.0 Hz, 1H), 4.19 – 4.11 (m, 1H), 4.06 – 4.00 (m, 2H), 3.76 (dq, J = 9.3, 7.0 Hz, 1H), 3.50 – 3.38 (m, 3H), 3.33 (s, 3H), 2.29 (td, J = 7.0, 2.0 Hz, 2H), 1.79 – 1.69 (m, 2H), 1.29 (d, J = 6.8 Hz, 3H), 1.19 (t, J = 7.0 Hz, 3H).
HRMS (+ESI) m/z calcd. for $[M + Na]^+$ $C_{15}H_{23}NNaO_5$: 320.1468, found: 320.1469. m/z calcd. for $[M - OEt]^+$ $C_{13}H_{18}NO_4$: 252.1230; found: 252.1231.

N-[(2S,3S)-2-Butyl-3-ethoxy-5-phenyl-4-pentynoyl)]-1,3-oxazolidine-2-thione (4m)

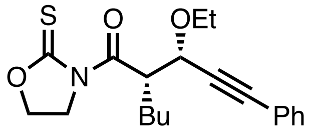

According to General Procedure F, the title compound was obtained from adduct **3m** (303 mg, 0.47 mmol, 1.0 equiv.) and CAN (1031 mg, 1.88 mmol, 4.0 equiv.) in acetone (12 mL, 0.04 M) after 30 min at –78 °C and 2 h 10 min at r.t. A 78:22 mixture of **4m** and the oxidized derivative **5m** was observed in the ^{1}H NMR of the crude.

The crude was purified by column chromatography (DCM/Hex/EtOAc, 50:48:2 to 50:44:6) to yield 104 mg (0.29 mmol, 61% yield) of adduct **4m**.

Colorless oil.
R_f 0.2 (DCM/Hex, 60:40).
$[\alpha]_D^{20}$ – 103 (c 1.0, $CHCl_3$).
IR (ATR) ν 2984, 2954, 2928, 2861, 1696, 1368, 1318, 1239, 1204, 1187, 1154 cm^{-1}.
^{1}H NMR (400 MHz, $CDCl_3$) δ 7.48 – 7.37 (m, 2H, ArH), 7.35 – 7.27 (m, 3H, ArH), 5.46 (td, J = 7.7, 6.1 Hz, 1H, COCHCH$_2$), 4.51 (d, J = 7.7 Hz, 1H, CHOEt), 4.50 – 4.44 (m, 1H,

$OC\underline{H}_aH_bCH$), 4.33 – 4.22 (m, 1H, $OCH_a\underline{H}_bCH_2$), 4.22 – 4.12 (m, 2H, $CH_2C\underline{H}_2N$), 3.83 (dq, J = 9.3, 7.0 Hz, 1H, $OCH_a\underline{H}_bCH_3$), 3.54 (dq, J = 9.3, 7.0 Hz, 1H, $OC\underline{H}_aH_bCH_3$), 2.01 – 1.85 (m, 2H, $COCHC\underline{H}_2$), 1.45 – 1.26 (m, 4H, $C\underline{H}_2C\underline{H}_2CH_3$), 1.23 (t, J = 7.0 Hz, 3H, $OCH_2C\underline{H}_3$), 0.90 (t, J = 7.1 Hz, 3H, $CH_2C\underline{H}_3$).

$^{13}C\{^1H\}$ NMR (101 MHz, $CDCl_3$) δ 185.6 (C), 174.8 (C), 132.0 (CH), 128.7 (CH), 128.5 (CH), 122.5 (C), 86.8 (C), 86.4 (C), 71.1 (CH), 66.2 (CH_2), 64.6 (CH_2), 47.6 (CH_2), 47.5 (CH), 29.3 (CH_2), 28.9 (CH_2), 23.0 (CH_2), 15.2 (CH_3), 14.1 (CH_3).

HRMS (+ESI) m/z calcd. for $[M + Na]^+$ $C_{20}H_{25}NNaO_3S$: 382.1447; found: 382.1461.

Chiral HPLC (Phenomenex Lux® Cellulose-5 column, 5% iPrOH in hexanes, flow rate 1 mL/min) Rt 8.5 min (2S,3S) enantiomer, Rt 9.5 min (2R,3R) enantiomer, 96:4 e.r.

N-[(2S,3S)-2-Butyl-3-ethoxy-5-phenyl-4-pentynoyl)]-1,3-oxazolidin-2-one (5m)

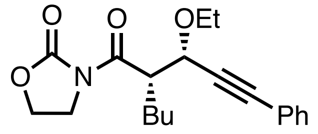

^1H NMR (400 MHz, $CDCl_3$) δ 7.45 – 7.39 (m, 2H), 7.33 – 7.27 (m, 3H), 4.50 – 4.43 (m, 2H), 4.36 (td, J = 8.6, 7.0 Hz, 1H), 4.26 – 4.17 (m, 1H), 4.05 – 3.99 (m, 2H), 3.84 (dq, J = 9.3, 7.0 Hz, 1H), 3.53 (dq, J = 9.3, 7.0 Hz, 1H), 1.94 – 1.80 (m, 2H), 1.38 – 1.26 (m, 4H), 1.23 (t, J = 7.0 Hz, 3H), 0.89 (t, J = 7.0 Hz, 3H).

HRMS (+ESI) m/z calcd. for $[M + Na]^+$ $C_{20}H_{25}NNaO_4$: 366.1676; found: 366.1676. m/z calcd. for $[M – OEt]^+$ $C_{18}H_{20}NO_3$: 298.1438; found: 298.1438.

N-[(2S,3S)-3-Ethoxy-2-isobutyl-5-phenyl-4-pentynoyl)]-1,3-oxazolidine-2-thione (4n)

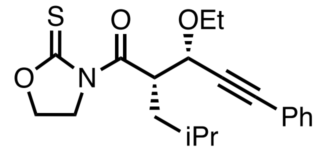

According to General Procedure F, the title compound was obtained from adduct **3n** (155 mg, 0.24 mmol, 1.0 equiv.) and CAN (461 mg, 0.84 mmol, 3.75 equiv.) in acetone (6 mL, 0.04 M) after 30 min at –78 °C and 2 h at r.t.

The crude was purified by column chromatography (Hex/EtOAc, 100:0 to 80:20) to yield 64 mg (0.18 mmol, 74% yield) of adduct **4n**.

Colorless oil.

R_f 0.3 (Hex/EtOAc, 80:20).

$[\alpha]_D^{25}$ – 100 (c 1.0, $CHCl_3$).

IR (ATR) ν 2956, 2928, 2868, 1696, 1489, 1366, 1320, 1189, 1154 cm^{-1}.

^1H NMR (400 MHz, $CDCl_3$) δ 7.48 – 7.39 (m, 2H, ArH), 7.34 – 7.27 (m, 3H, ArH), 5.57 (ddd, J = 9.8, 7.2, 3.9 Hz, 1H, COCH), 4.54 – 4.41 (m, 2H, CHOEt, $OCH_aH_bCH_2$), 4.32 – 4.11 (m, 3H, $OCH_aH_bCH_2$, NCH_2CH_2), 3.81 (dq, J = 9.3, 7.0 Hz, 1H, $OCH_aH_bCH_3$), 3.54 (dq, J = 9.3, 7.0 Hz, 1H, $OCH_aH_bCH_3$), 1.95 (ddd, J = 13.4, 9.8, 5.5 Hz, 1H, $COCHCH_aH_b$), 1.74 (ddd, J = 13.4, 8.2, 3.9 Hz, 1H, $COCHCH_aH_b$), 1.69 – 1.55 (m, 1H, $CH(CH_3)_2$), 1.22 (t, J = 7.0 Hz, 3H, OCH_2CH_3), 0.96 (d, J = 7.4 Hz, 3H, $CH(CH_3)_a(CH_3)_b$), 0.94 (d, J = 6.5 Hz, 3H, $CH(CH_3)_a(CH_3)_b$).

$^{13}C\{^1H\}$ NMR (101 MHz, $CDCl_3$) δ 185.6 (C), 174.8 (C), 132.0 (CH), 128.7 (CH), 128.5 (CH), 122.5 (C), 87.0 (C), 86.0 (C), 71.5 (CH), 66.2 (CH_2), 64.5 (CH_2), 47.7 (CH_2), 45.5 (CH), 38.0 (CH_2), 26.6 (CH), 23.4 (CH_3), 22.8 (CH_3), 15.2 (CH_3).

HRMS (+ESI) m/z calcd. for $[M - OEt]^+$ $C_{18}H_{20}NO_2S$: 314.1215; found: 314.1207. m/z calcd. for $[M + H]^+$ $C_{20}H_{26}NO_3S$: 360.1628; found: 360.1628.

Chiral HPLC (Phenomenex Lux® Amylose-1 column, 10% iPrOH in hexanes, flow rate 1 mL/min) Rt 7.5 min (2S,3S) enantiomer, Rt 8.5 min (2R,3R) enantiomer, 98:2 e.r.

N-[(2S,3S)-Hexacarbonyl{μ-[η^4-(2-cyanomethyl-3-ethoxy-5-phenyl-4-pentynoyl)] dicobalt(Co-Co)}]-1,3-oxazolidine-2-thione (4o)

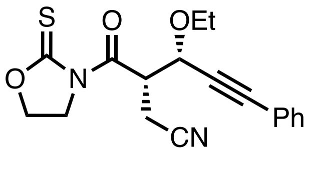

According to General Procedure F, the title compound was obtained from adduct **3o** (266 mg, 0.42 mmol, 1.0 equiv.) and CAN (870 mg, 1.59 mmol, 3.75 equiv.) in acetone (10 mL, 0.04 M) after 30 min at –78 °C and 2 h at r.t.

The crude was purified by column chromatography (Hex/EtOAc, 90:10 to 60:40) to yield 108 mg (0.32 mmol, 75% yield) of adduct **4o**.

White solid.

Mp 98–100 °C.

R$_f$ 0.3 (Hex/EtOAc, 70:30).

[α]$_D^{25}$ – 60 (c 1.0, $CHCl_3$).

IR (ATR) ν 2973, 2922, 2881, 2242, 1687, 1385, 1362, 1325, 1299 cm^{-1}.

^{1}H NMR (400 MHz, $CDCl_3$) δ 7.49 – 7.40 (m, 2H, Ar<u>H</u>), 7.38 – 7.28 (m, 3H, Ar<u>H</u>), 5.48 (ddd, J = 8.9, 5.8, 4.2 Hz, 1H, COC<u>H</u>), 4.88 (d, J = 5.8 Hz, 1H, C<u>H</u>OEt), 4.60 (ddd, J = 9.5, 8.9, 6.2 Hz, 1H, OC<u>H</u>$_a$CH$_b$CH$_2$), 4.45 (ddd, J = 9.2, 8.9, 8.7 Hz, 1H, OCH$_a$C<u>H</u>$_b$CH$_2$), 4.32 (ddd, J = 11.4, 9.5, 8.7 Hz, 1H, NC<u>H</u>$_a$CH$_b$CH$_2$), 4.19 (ddd, J = 11.4, 9.2, 6.2 Hz, 1H, NCH$_a$C<u>H</u>$_b$CH$_2$), 3.85 (dq, J = 9.3, 7.0 Hz, 1H, OC<u>H</u>$_a$CH$_b$CH$_3$), 3.50 (dq, J = 9.3, 7.0 Hz, 1H, OCH$_a$C<u>H</u>$_b$CH$_3$), 3.10 (dd, J = 16.9, 8.9 Hz, 1H, C<u>H</u>$_a$CH$_b$CN), 2.96 (dd, J = 16.9, 4.2 Hz, 1H, CH$_a$C<u>H</u>$_b$CN), 1.21 (t, J = 7.0 Hz, 3H, OCH$_2$C<u>H</u>$_3$).

^{13}C{^{1}H} NMR (101 MHz, $CDCl_3$) δ 185.3 (C), 170.5 (C), 132.0 (CH), 129.1 (CH), 128.5 (CH), 121.7 (C), 118.1 (C), 88.0 (C), 84.4 (C), 69.0 (CH), 66.9 (CH_2), 65.1 (CH_2), 47.6 (CH_2), 45.7 (CH), 15.9 (CH_2), 15.0 (CH_3).

HRMS (+ESI) m/z calcd. for [M – OEt]$^+$ $C_{16}H_{13}N_2O_2S$: 297.0698; found: 297.0688. m/z calcd. for [M + H]$^+$ $C_{18}H_{19}N_2O_3S$: 344.1111; found: 344.1102.

Chiral HPLC (Phenomenex Lux® Cellulose-5 column, 40% iPrOH in hexanes, flow rate 1 mL/min) Rt 11.8 min (2S,3S) enantiomer, Rt 14.8 min (2R,3R) enantiomer, 95:5 e.r.

N-[(2S,3S)-3-Ethoxy-2-(3-methoxy-3-oxopropyl)5-phenyl-4-pentynoyl)]-1,3-oxazolidine-2-thione (4p)

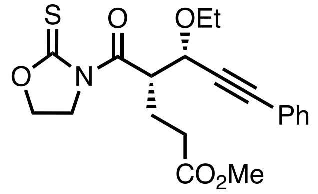

According to General Procedure F, the title compound was obtained from adduct **3p** (270 mg, 0.40 mmol, 1.0 equiv.) and CAN (767 mg, 1.40 mmol, 3.5 equiv.) in acetone (10 mL, 0.04 M) after 30 min at –78 °C and 2 h at r.t.

The crude was purified by column chromatography (Hex/EtOAc, 80:20 to 60:40) to yield 128 mg (0.33 mmol, 82% yield) of adduct **4p**.

Colorless oil.

R$_f$ 0.2 (Hex/EtOAc, 70:30).

[α]$_D^{25}$ – 59 (c 1.0, $CHCl_3$).

IR (ATR) ν 2974, 2950, 2870, 1733, 1690, 1385, 1489, 1440, 1368, 1321 cm^{-1}.

^{1}H NMR (400 MHz, $CDCl_3$) δ 7.47 – 7.36 (m, 2H, ArH), 7.34 – 7.27 (m, 3H, ArH), 5.49 – 5.40 (m, 1H, COCH), 4.56 (d, J = 7.7 Hz, 1H, CHOEt), 4.50 (td, J = 8.5, 6.2 Hz, 1H, OCH$_a$CH$_b$CH$_2$), 4.33 – 4.11 (m, 3H, OCH$_a$CH$_b$CH$_2$, NCH$_2$CH$_2$), 3.84 (dq, J = 9.3, 7.0 Hz, 1H, OCH$_a$CH$_b$CH$_3$), 3.66 (s, 3H, OCH$_3$), 3.52 (dq, J = 9.3, 7.0 Hz, 1H, OCH$_a$CH$_b$CH$_3$), 2.58 – 2.39 (m, 2H, CH$_2$CO$_2$Me), 2.36 – 2.15 (m, 2H, COCHCH$_2$), 1.22 (t, J = 7.0 Hz, 3H, OCH$_2$CH$_3$).

^{13}C{^{1}H} NMR (101 MHz, $CDCl_3$) δ 185.6 (C), 174.1 (C), 173.6 (C), 132.0 (CH), 128.8 (CH), 128.5 (CH), 122.3 (C), 87.0 (C), 86.0 (C), 70.7 (CH), 66.4 (CH$_2$), 64.6 (CH$_2$), 51.8 (CH$_3$), 47.6 (CH$_2$), 47.0 (CH), 31.6 (CH$_2$), 24.0 (CH$_2$), 15.1 (CH$_3$).

HRMS (+ESI) m/z calcd. for [M – OEt]$^+$ $C_{18}H_{18}NO_4S$: 344.0957; found: 344.0950. m/z calcd. for [M + H]$^+$ $C_{20}H_{24}NO_5S$: 390.1370; found: 390.1364.

Chiral HPLC (Phenomenex Lux® Cellulose-5 column, 30% iPrOH in hexanes, flow rate 1 mL/min) Rt 10.7 min (2S,3S) enantiomer, Rt 12.0 min (2R,3R) enantiomer, 99:1 e.r.

N-[(2S,3R)-2-Azido-3-ethoxy-5-phenyl-4-pentynoyl)]-1,3-oxazolidine-2-thione (4q)

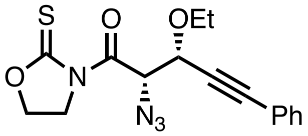

According to General Procedure F, the title compound was obtained from adduct **3q** (169 mg, 0.29 mmol, 1.0 equiv.) and CAN (514 mg, 0.90 mmol, 3.5 equiv.) in acetone (7 mL, 0.04 M) after 30 min at –78 °C and 2 h at r.t.

The crude was purified by column chromatography (Hex/EtOAc, 90:10 to 60:40) to yield 72 mg (0.21 mmol, 78% yield) of adduct **4q**.

Pale yellow solid.

Mp 110–112 °C.

R$_f$ 0.3 (Hex/EtOAc, 70:30).

[α]$_D^{20}$ – 122 (c 0.8, $CHCl_3$).

IR (ATR) ν 2979, 2964, 2921, 2875, 2089, 1699, 1493, 1475, 1362, 1324, 1286, 1244, 1167, 1091, 1018 cm^{-1}.

^{1}H NMR (400 MHz, CDCl$_3$) δ 7.50 – 7.40 (m, 2H, ArH), 7.36 – 7.29 (m, 3H, ArH), 6.45 (d, J = 6.8 Hz, 1H, COCHN$_3$), 4.92 (d, J = 6.8 Hz, 1H, CHOEt), 4.58 (td, J = 9.2, 6.4 Hz, 1H, OCH$_a$H$_b$CH$_2$), 4.43 (td, J = 9.2, 8.5 Hz, 1H, OCH$_a$H$_b$CH$_2$), 4.33 – 4.11 (m, 2H, NCH$_2$), 3.93 (dq, J = 9.2, 7.0 Hz, 1H, OCH$_a$H$_b$CH$_3$), 3.61 (dq, J = 9.2, 7.0 Hz, 1H, OCH$_a$H$_b$CH$_3$), 1.28 (t, J = 7.0 Hz, 3H, OCH$_2$CH$_3$).
^{13}C{^{1}H} NMR (101 MHz, CDCl$_3$) δ 185.2 (C), 168.5 (C), 132.1 (CH), 129.4 (CH), 128.5 (CH), 121.9 (C), 88.4 (C), 83.7 (C), 70.9 (CH), 66.9 (CH$_2$), 65.3 (CH$_2$), 62.8 (CH), 47.4 (CH$_2$), 15.2 (CH$_3$).
HRMS (+ESI) m/z calcd. for [M – OEt]$^+$ $C_{14}H_{11}N_4O_2S$: 299.0597; found: 299.0586. m/z calcd. for [M + Na]$^+$ $C_{16}H_{16}N_4NaO_3S$: 367.0835, found: 367.0829.
Chiral HPLC (Phenomenex Lux® Amylose-3 column, 5% iPrOH in hexanes, flow rate 1 mL/min) Rt 17.9 min (2R,3S) enantiomer, Rt 28.3 min (2S,3R) enantiomer, 97:3 e.r.

N-[(2S,3R)-3-Ethoxy-5-phenyl-2-pivaloyloxy-4-pentynoyl)]-1,3-oxazolidine-2-thione (4r)

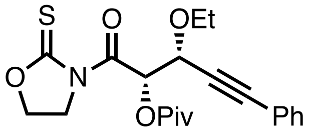

According to General Procedure F, the title compound was obtained from adduct **3r** (134 mg, 0.20 mmol, 1.0 equiv.) and CAN (481 mg, 0.88 mmol, 4.5 equiv.) in acetone (4.8 mL, 0.04 M) after 30 min at –78 °C and 3 h at r.t.

The crude was purified by column chromatography (DCM/Hex/EtOAc, 50:48:2 to 50:44:6) to yield 79 mg (0.20 mmol, 99% yield) of adduct **4r**.

Light yellow oil.
R$_f$ 0.4 (DCM/Hex/EtOAc, 50:48:2).
$[\alpha]_D^{25}$ – 41 (c 1.0, CHCl$_3$).
IR (ATR) ν 2969, 2926, 2872, 2855, 1718, 1478, 1370, 1327, 1286, 1213, 1146 cm^{-1}.
^{1}H NMR (400 MHz, CDCl$_3$) δ 7.48 – 7.39 (m, 2H, ArH), 7.36 – 7.30 (m, 3H, ArH), 7.29 (d, J = 5.1 Hz, 1H, COCHOPiv), 5.23 (d, J = 5.1 Hz, 1H, CHOEt), 4.58 (ddd, J = 9.5, 8.8, 6.2 Hz, 1H, OCH$_a$H$_b$CH$_2$), 4.43 (q, J = 8.8 Hz, 1H, OCH$_a$H$_b$CH$_2$), 4.30 (ddd, J = 11.2, 9.5, 8.8 Hz, 1H, NCH$_a$H$_b$CH$_2$), 4.15 (ddd, J = 11.2, 9.3, 6.2 Hz, 1H, NCH$_a$H$_b$CH$_2$), 3.89 (dq, J = 9.5, 7.0 Hz, 1H, OCH$_a$H$_b$CH$_3$), 3.71 (dq, J = 9.5, 7.0 Hz, 1H, OCH$_a$H$_b$CH$_3$), 1.30 (s, 9H, COC(CH$_3$)$_3$), 1.21 (t, J = 7.0 Hz, 3H, OCH$_2$CH$_3$).

$^{13}C\{^{1}H\}$ NMR (101 MHz, $CDCl_3$) δ 185.1 (C), 177.9 (C), 168.0 (C), 132.0 (CH), 129.0 (CH), 128.5 (CH), 122.1 (C), 88.2 (C), 82.9 (C), 73.8 (CH), 68.6 (CH), 67.1 (CH_2), 64.6 (CH_2), 47.4 (CH_2), 38.8 (C), 27.1 (CH_3), 15.2 (CH_3).

HRMS (+ESI) m/z calcd. for $[M – OEt]^+$ $C_{19}H_{20}NO_4S$: 358.1108; found: 358.1116. m/z calcd. for $[M + H]^+$ $C_{21}H_{26}NO_5S$: 404.1526; found: 404.1535.

Chiral HPLC (Phenomenex Lux® Cellulose-1 column, 5% iPrOH in hexanes, flow rate 1 mL/min) Rt 17.5 min (2S,3R) enantiomer, Rt 20.9 min (2R,3S) enantiomer, 98:2 e.r.

6. Three-Step Methyl Ester Route

General Procedure G for the synthesis of methyl esters

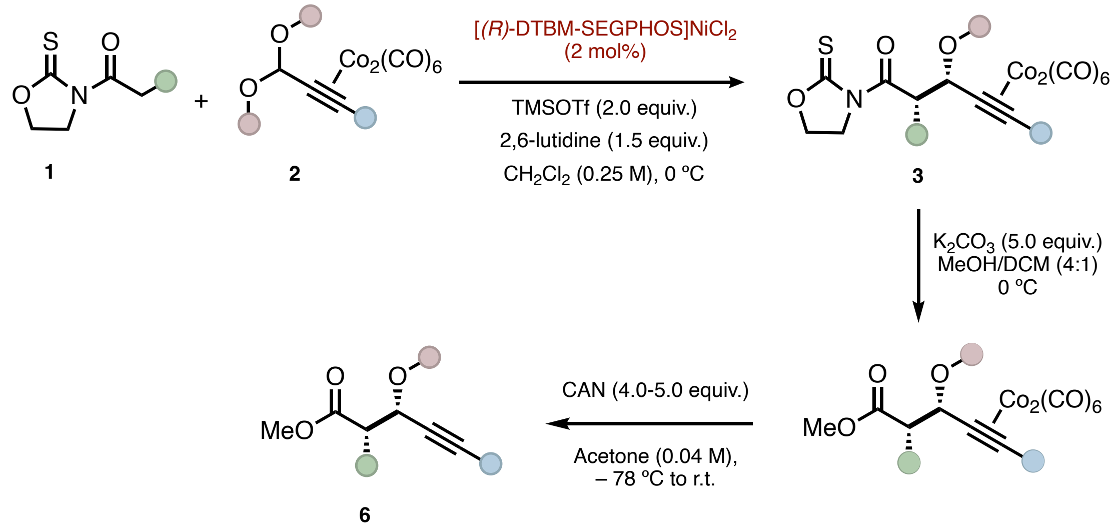

Step 1: Aldol-type reaction.

A solution of a thioimide (0.5 mmol, 1.0 equiv.), the corresponding propargylic cobalted acetal (0.55 mmol, 1.1 equiv.) and [(R)-DTBM-SEGPHOS]NiCl₂ (2-5 mol%) in DCM (2 mL, 0.25 M) was cooled to 0 °C. Then, neat TMSOTf (185 µL, 1.0 mmol, 2.0 equiv.) was added dropwise and stirred for 3 min. 2,6-lutidine (90 µL, 0.75 mmol, 1.5 equiv.) was then added and the resultant mixture was stirred at 0 °C.

The mixture was then quenched with a saturated solution of NH_4Cl (2 mL) and extracted with DCM (3 × 5 mL). The combined organic extracts were dried over $MgSO_4$, filtered through Celite®, and concentrated in vacuo. The resulting crude was used in the next step without further purification.

Step 2: Methyl ester formation.

A solution of the unpurified aldol adduct in DCM (2.5 mL per mmol of the aldol adduct) was added to a solution of K_2CO_3 (5.0 equiv.) in MeOH (9 mL per mmol of K_2CO_3) at 0 °C. The solution was stirred for 2 h.

The reaction was then quenched with a saturated solution of NH_4Cl (6 mL/mmol) and partitioned in DCM (5 mL)and water (5 mL). The organic phase was extracted with DCM (3 × 15 mL/0.5 mmol) and the combined organic extracts were washed with 2M NaOH (2 × 15 mL/0.5 mmol), dried over $MgSO_4$ and concentrated in vacuo. The resulting crude was used in the next step without further purification.

Step 3: Decomplexation.
The unpurified methyl ester was added to a stirring solution of CAN (3.0 to 5.0 equiv.) in acetone (12.5 mL/0.5 mmol) and was stirred at –78 °C for 30 min. Afterwards, the reaction mixture was allowed to warm to r.t. for at least an additional hour. Reaction completion was monitored by TLC.

After consumption of the starting material, the solution was partitioned in diethyl ether (20 mL) and brine (15 mL), and the aqueous layer was extracted thrice with Et_2O (3 × 15 mL/0.5 mmol). The combined organic extracts were dried with $MgSO_4$, filtered, and evaporated under reduced pressure. Finally, the crude was purified by column chromatography to yield the named compound as a single enantiomer.

Methyl (2S,3S)-3-ethoxy-2-methyl-5-phenyl-4-pentynoate (6a)

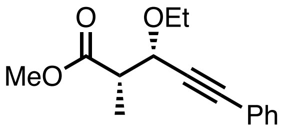

According to General Procedure G, the title compound was obtained from N-propanoyl-1,3-oxazolidine-2-thione (**1a**, 80 mg, 0.5 mmol, 1.0 equiv.) and hexacarbonyl μ-[η^4-(1,1-diethoxy-3-phenylpropyne)dicobalt] (**2a**, 270 mg, 0.55 mmol, 1.1 equiv.) at 0 °C for 5 h, followed by subsequent treatment with K_2CO_3 (346 mg, 2.50 mmol, 5.0 equiv.) in MeOH at 0 °C for 2 h and posterior decomplexation by CAN (1.371 g, 2.50 mmol, 5.0 equiv.) for a total of 2 h and 30 min. The analysis of the resultant oil by ^{1}H NMR established the formation of a single diastereomer.

The crude was purified by column chromatography (Hex/DCM, 70:30 to 30:70) to yield 110 mg (0.45 mmol, 89% yield) of methyl ester **6a**.

Colorless oil.
R$_f$ 0.2 (Hex/DCM, 70:30).
$[\alpha]_D^{25}$ – 67 (c 1.0, $CHCl_3$).
IR (ATR) ν 2976, 2948, 2876, 1735, 1489, 1456, 1435, 1333, 1262, 1198, 1169, 1092, 1072, 1029 cm^{-1}.
^{1}H NMR (400 MHz, $CDCl_3$) δ 7.48 – 7.38 (m, 2H, Ar$\underline{H}$), 7.34 – 7.26 (m, 3H, Ar$\underline{H}$), 4.58 (d, J = 5.9 Hz, 1H, C$\underline{H}$OEt), 3.85 (dq, J = 9.4, 6.9 Hz, 1H, OC$\underline{H}_a$H$_b$CH$_3$), 3.72 (s, 3H, OC$\underline{H}_3$),

3.52 (dq, J = 9.4, 6.9 Hz, 1H, $OCH_a\underline{H}_bCH_3$), 2.85 (qd, J = 7.0, 5.9 Hz, 1H, $COC\underline{H}CH_3$), 1.36 (d, J = 7.0 Hz, 3H, $COCHC\underline{H}_3$), 1.22 (t, J = 7.0 Hz, 3H, $OCH_2C\underline{H}_3$).

$^{13}C\{^1H\}$ NMR (101 MHz, $CDCl_3$) δ 173.8 (C), 131.9 (CH), 128.5 (CH), 128.4 (CH), 122.7 (C), 86.8 (C), 86.4 (C), 70.7 (CH), 65.0 (CH_2), 52.0 (CH_3), 45.5 (CH), 15.1 (CH_3), 12.4 (CH_3)

HRMS (+ESI) m/z calcd. for $[M – Na]^+$ $C_{15}H_{18}NaO_3$: 269.1148; found: 269.1150.

Chiral HPLC (Phenomenex Lux® Amylose-3 column, 1% iPrOH in hexanes, flow rate 1 mL/min) Rt 6.8 min (2S,3S) enantiomer, Rt 8.4 min (2R,3R) enantiomer, 99:1 e.r.

Methyl (2S,3S)-3-ethoxy-2-methyl-4-nonynoate (6e)

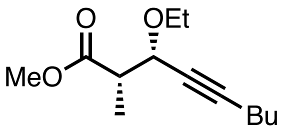

According to General Procedure G, the title compound was obtained from N-propanoyl-1,3-oxazolidine-2-thione (**1a**, 80 mg, 0.5 mmol, 1.0 equiv.) and hexacarbonyl μ-[η4-(1,1-diethoxy-2-heptyne)dicobalt] (**2e**, 259 mg, 0.55 mmol, 1.1 equiv.) at 0 °C for 3 h, followed by subsequent treatment with K_2CO_3 (346 mg, 2.50 mmol, 5.0 equiv.) in MeOH at 0 °C for 2 h and posterior decomplexation by CAN (1.01 g, 2.00 mmol, 4.0 equiv.) for a total of 2 h and 30 min. The analysis of the resultant oil by ^1H NMR established the formation of a single diastereomer.

The crude was purified by column chromatography (Hex/Et_2O, from 1:0 to 91:9) to yield 101 mg (0.45 mmol, 90% yield) of methyl ester **6e**.

Colorless oil.

R_f 0.3 (Hex/Et_2O, 95:5).

$[\alpha]_D^{20}$ – 39 (c 1.0, $CHCl_3$).

IR (ATR) ν 2976, 2952, 2872, 1737, 1642, 1457, 1435, 1333, 1198, 1169, 1090 cm^{-1}.

^1H NMR (400 MHz, $CDCl_3$) δ 4.31 (dt, J = 6.2, 2.0 Hz, 1H, $C\underline{H}OEt$), 3.75 (dq, J = 9.4, 7.0 Hz, 1H, $OC\underline{H}_aH_bCH_3$), 3.69 (s, 3H, $OC\underline{H}_3$), 3.42 (dq, J = 9.4, 7.0 Hz, 1H, $OCH_a\underline{H}_bCH_3$), 2.71 (qd, J = 7.1, 6.2 Hz, 1H, $COC\underline{H}CH_3$), 2.20 (td, J = 6.9, 2.0 Hz, 2H, $C≡CC\underline{H}_2$), 1.51 – 1.42 (m, 2H, $C≡CCH_2C\underline{H}_2$), 1.42 – 1.33 (m, 2H, $CH_2C\underline{H}_2CH_3$), 1.27 (d, J = 7.1 Hz, 3H, $COCHC\underline{H}_3$), 1.18 (t, J = 7.0 Hz, 3H, $OCH_2C\underline{H}_3$), 0.90 (t, J = 7.2 Hz, 3H, $CH_2CH_2C\underline{H}_3$).

$^{13}C\{^1H\}$ NMR (101 MHz, $CDCl_3$) δ 174.1 (C), 87.1 (C), 77.6 (C), 70.4 (CH), 64.5 (CH_2), 51.8 (CH_3), 45.7 (CH), 30.8 (CH_2), 22.0 (CH_2), 18.5 (CH_2), 15.0 (CH_3), 13.7 (CH_3), 12.4 (CH_3).

HRMS (+ESI) m/z calcd. for $[M – Na]^+$ $C_{13}H_{22}NaO_3$: 249.1461; found: 249.1472.

Chiral GC (GC Column CP-ChiraSil-DEX CB, Initial Temp. 100 °C, equilibration time 1 min, 2 °C/min until 160 °C, hold time 0.5 min, 10 °C/min until 180 °C, hold time 5 min), Rt 15.0 min (2S,2R) enantiomer, 15.3 min (2R,3R) enantiomer, > 99:1 e.r.

Methyl (2S,3S)-3-ethoxy-2-methyl-7-octen-4-ynoate (6f)

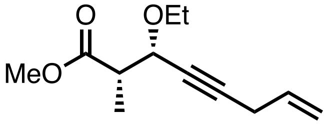

According to General Procedure G, the title compound was obtained from N-propanoyl-1,3-oxazolidine-2-thione (**1a**, 80 mg, 0.5 mmol, 1.0 equiv.) and hexacarbonyl μ-[η4-(6,6-diethoxy-1-hexen-4-yne)dicobalt] (**2f**, 250 mg, 0.55 mmol, 1.1 equiv.) at 0 °C for 5 h, followed by subsequent treatment with K_2CO_3 (346 mg, 2.50 mmol, 5.0 equiv.) in MeOH at 0 °C for 2 h and posterior decomplexation by CAN (1.01 g, 2.00 mmol, 4.0 equiv.) for a total of 2 h and 30 min. The analysis of the resultant oil by ^1H NMR established the formation of a single diastereomer.

The crude was purified by column chromatography (Hex/Et_2O, from 100:0 to 80:20) to yield 69 mg (0.35 mmol, 70% yield) of methyl ester **6f**.

Light yellow oil.

R_f 0.3 (Hex/Et_2O, 95:5).

$[\alpha]_D^{20}$ – 31 (c 1.0, $CHCl_3$).

IR (ATR) ν 2956, 2932, 2872, 1739, 1457, 1433, 1198, 1090, 1075, 755 cm^{-1}.

^1H NMR (400 MHz, $CDCl_3$) δ 5.80 (ddt, J = 17.0, 10.0, 5.2 Hz, 1H, CH_2=C<u>H</u>), 5.31 (dq, J = 17.0, 1.7 Hz, 1H, C$\underline{H}_a$$H_b$=CH), 5.11 (dq, J = 10.0, 1.7 Hz, 1H, C$H_a$$\underline{H}_b$=CH), 4.36 (dt, J = 6.1, 1.9 Hz, 1H, C<u>H</u>OEt), 3.77 (dq, J = 9.4, 7.0 Hz, 1H, OC$\underline{H}_a$$H_b$$CH_3$), 3.69 (s, 3H, OC$\underline{H}_3$), 3.44 (dq, J = 9.4, 7.0 Hz, 1H, OC$H_a$$\underline{H}_b$$CH_3$), 3.00 (dq, J = 5.2, 1.9 Hz, 2H, C$\underline{H}_2$CH=CH_2), 2.74 (qd, J = 7.1, 6.1 Hz, 1H, COC<u>H</u>CH_3), 1.29 (d, J = 7.1 Hz, 3H, COCHC$\underline{H}_3$), 1.18 (t, J = 7.0 Hz, 3H, OCH_2C$\underline{H}_3$).

$^{13}C\{^1H\}$ NMR (101 MHz, $CDCl_3$) δ 174.0 (C), 132.4 (CH), 116.2 (CH_2), 83.4 (C), 80.2 (C), 70.4 (CH), 64.7 (CH_2), 51.9 (CH_3), 45.6 (CH), 23.1 (CH_2), 15.0 (CH_3), 12.4 (CH_3).

HRMS (+ESI) m/z calcd. for $[M – Na]^+$ $C_{12}H_{18}NaO_3$: 233.1148; found: 233.1155.
Chiral GC (GC Column CP-ChiraSil-DEX CB, Initial Temp. 80 °C, equilibration time 2 min, 5 °C/min until 180 °C, hold time 5 min), Rt 12.6 min (2S,2R) enantiomer, 12.8 min (2R,3R) enantiomer, > 99:1 e.r.

Methyl (2S,3S)-3-ethoxy-8-methoxy-2-methyl-4-octynoate (6l)

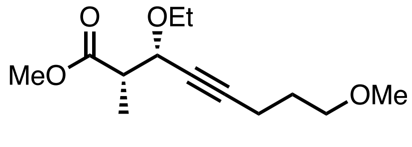

According to General Procedure G, the title compound was obtained from N-propanoyl-1,3-oxazolidine-2-thione (**1a**, 80 mg, 0.5 mmol, 1.0 equiv.) and hexacarbonyl μ-[η4-(6-methoxy-1,1-diethoxy-2-hexyne)dicobalt] (**2l**, 277 mg, 0.55 mmol, 1.1 equiv.) at 0 °C for 5 h, followed by subsequent treatment with K_2CO_3 (346 mg, 2.50 mmol, 5.0 equiv.) in MeOH at 0 °C for 2 h and posterior decomplexation by CAN (1.01 g, 2.00 mmol, 4.0 equiv.) for a total of 2 h. The analysis of the resultant oil by ^1H NMR established the formation of a single diastereomer.

The crude was purified by column chromatography (Hex/EtOAc, from 100:0 to 80:20) to give 104 mg (0.43 mmol, 86% yield) of methyl ester **6l**.

Colorless oil.
R_f 0.3 (Hex/EtOAc, 90:10).
$[\alpha]_D^{20}$ – 47 (c 1.0, $CHCl_3$).
IR (ATR) ν 2976, 2930, 2872, 2227, 1735, 1457, 1435, 1198, 1170, 1118, 1090 cm^{-1}.
^1H NMR (400 MHz, $CDCl_3$) δ 4.30 (dt, J = 6.1, 2.0 Hz, 1H, C<u>H</u>OEt), 3.74 (dq, J = 9.4, 7.0 Hz, 1H, $OC\underline{H}_aH_bCH_3$), 3.68 (s, 3H, $OCOC\underline{H}_3$), 3.43 (t, J = 6.3 Hz, 2H, $CH_2C\underline{H}_2OMe$), 3.41 (dq, J = 9.4, 7.0 Hz, 1H, $OCH_a\underline{H}_bCH_3$), 3.32 (s, 3H, $CH_2OC\underline{H}_3$), 2.70 (qd, J = 7.1, 6.1 Hz, 1H, $COC\underline{H}CH_3$), 2.29 (td, J = 7.1, 2.0 Hz, 2H, $C{\equiv}CC\underline{H}_2$), 1.74 (tt, J = 7.1, 6.3 Hz, 2H, $C{\equiv}CCH_2C\underline{H}_2$), 1.26 (d, J = 7.1 Hz, 3H, $COCHC\underline{H}_3$), 1.17 (t, J = 7.0 Hz, 3H, $OCH_2C\underline{H}_3$).
$^{13}C\{^1H\}$ NMR (101 MHz, $CDCl_3$) δ 174.0 (C), 86.3 (C), 78.0 (C), 71.2 (CH_2), 70.4 (CH), 64.6 (CH_2), 58.8 (CH_3), 51.9 (CH_3), 45. 7 (CH), 28.8 (CH_2), 15.6 (CH_2), 15.0 (CH_3), 12.4 (CH_3).
HRMS (+ESI) m/z calcd. for $[M – OEt]^+$ $C_{11}H_{17}O_3$: 197.1172; found: 197.1174. m/z calcd. for $[M + H]^+$ $C_{13}H_{23}O_4$: 243.1591; found: 243.1593.

Chiral GC (GC Column CP-ChiraSil-DEX CB, Initial Temp. 100 ºC, equilibration time 1 min, 2 ºC/min until 160 ºC, hold time 0.5 min, 10 ºC/min until 180 ºC, hold time 5 min), Rt 21.0 min (2S,2R) enantiomer, 21.2 min (2R,3R) enantiomer, > 99:1 e.r.

7. One-pot Pauson-Khand Reaction

7.1. Optimization of the intramolecular Pauson-Khand cyclization.

A solution of a **1h** (0.5 mmol, 1.0 equiv.), **2a** (0.55 mmol, 1.1 equiv.) and [(R)-DTBM-SEGPHOS]$NiCl_2$ (2 mol%) in DCM (2 mL) was cooled to 0 °C. Then, neat TMSOTf (185 μL, 1.0 mmol, 2.0 equiv.) was added dropwise and stirred for 3 min. 2,6-lutidine (90 μL, 0.75 mmol, 1.5 equiv.) was then added and the resultant mixture was stirred at 0 °C for 16 h.

The mixture was then quenched with a saturated solution of NH_4Cl (2 mL) and extracted with DCM (3 × 5 mL). The combined organic extracts were dried over $MgSO_4$, filtered through Celite®, and concentrated in vacuo.

The corresponding promoter (3.5–5.0 equiv.) was added to a purged solution of the crude oil in the adequate solvent (0.05 M). The reaction mixture was stirred for 24 h at r.t.

Afterwards, the solution was filtered through a 1:1 mixture of SiO_2/Celite® and eluted with DCM, and the volatiles were evaporated under reduced pressure.

Table S4. Influence of the promoter.

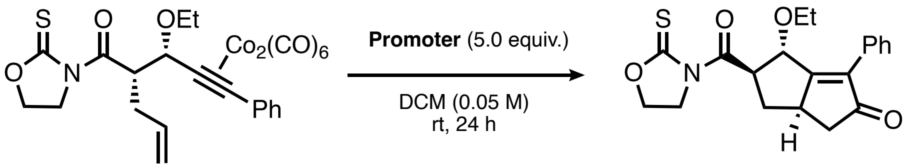

Entry	Promoter	Yield (%)[a]
1	NMO·H_2O	62
2	NMO·H_2O (3.5 equiv.)	43
3	NMO	< 3
4	TMANO	32
5	TMANO·2 H_2O	< 3
6	Me_2S	< 3

[a] ^1H NMR yields with respect to methyl 4-nitrobenzoate as internal standard.

Table S5. Solvent screening.

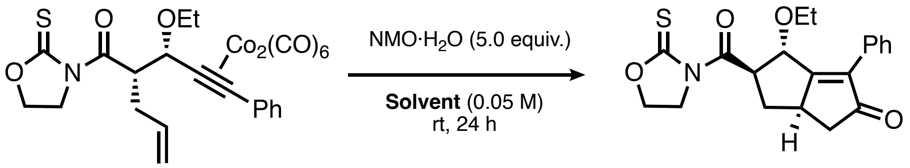

Entry	Solvent	Yield (%)[a]
1	DCM	62
2[b]	DCM	52(51)
3	ACN	< 3
4	THF	46
5	Tol	< 3
6	DCE	56

[a] ^{1}H NMR yields with respect to methyl 4-nitrobenzoate as internal standard. Isolated yields are indicated between parentheses. [b] One-pot procedure.

The formation of side-products due to the deprotection of the triple bond and/or oxidation of the thiocarbonyl bond could be observed via ^{1}H NMR analysis of the crude mixture in all two-pot procedures. Carrying out the reaction in one pot reduced significantly the amount of undesired side-products.

7.2. General Procedure H for the One-Pot Pauson-Khand Reaction.

A solution of a thioimide (0.5 mmol, 1.0 equiv.), the corresponding propargylic cobalted acetal (0.55 mmol, 1.1 equiv.) and [(R)-DTBM-SEGPHOS]$NiCl_2$ (2 mol%) in DCM (2 mL) was cooled to 0 °C. Then, neat TMSOTf (185 µL, 1.0 mmol, 2.0 equiv.) was added dropwise and stirred for 3 min. 2,6-lutidine (90 µL, 0.75 mmol, 1.5 equiv.) was then added and the resultant mixture was stirred at 0 °C for 16 h. Afterwards, NMO·H_2O (338 mg, 2.5 mmol, 5.0 equiv., from Fluorochem) was added to the reaction mixture, which was sealed again and stirred at r.t. for 24 h.

The reaction mixture was then filtered through a 1:1 mixture of SiO_2/Celite® and eluted with DCM. The volatiles were evaporated under reduced pressure and the resulting crude purified via column chromatography. The diasteromeric ratio (dr) was established in each case by HPLC-MS analysis of the crude mixture.

Bicycle 10a

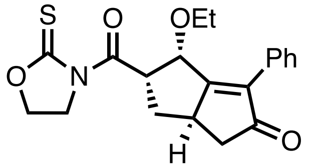

According to General Procedure H, the title compound was obtained from N-(4-pentenoyl)-1,3-oxazolidine-2-thione (**1h**, 46 mg, 0.25 mmol, 1.0 equiv.) and hexacarbonyl μ-[η^4-(1,1-diethoxy-3-phenylpropyne)dicobalt] (**F**, 135 mg, 0.28 mmol, 1.1 equiv.) at 0 °C for 16 h, followed by the addition of NMO·H_2O (167 mg, 1.25 mmol, 5.0 equiv.). The reaction mixture was stirred for an additional 24 h at r.t.

The residue (d.r. 88:8:4) was purified by column chromatography (Hex/EtOAc, from 70:30 to 30:70) to afford 47 mg of pure **10a** (0.13 mmol, 51% yield).

Off-white solid.

Mp 38–40 °C.

R_f 0.3 (Hex/EtOAc, 60:40).

$[\alpha]_D^{20}$ + 19 (c 1.0, $CHCl_3$).

IR (ATR) ν 2969, 2922, 2855, 1700, 1377, 1359, 1316, 1191, 1156, 1115, 1074 cm^{-1}.

^{1}H NMR (400 MHz, $CDCl_3$) δ 7.48 – 7.31 (m, 5H, ArH), 5.49 – 5.40 (m, 1H, COCH), 5.34 (d, J = 7.5 Hz, 1H, CHOEt), 4.63 – 4.46 (m, 2H, OCH_2CH_2N), 4.33 (ddd, J = 11.4, 9.6, 7.9 Hz, 1H, $OCH_2CH_aH_bN$), 4.21 (ddd, J = 11.4, 9.3, 7.2 Hz, 1H, $OCH_2CH_aH_bN$), 3.56 – 3.45 (m, 1H, CH_2CHCH_2CO), 3.37 (dq, J = 9.1, 7.0 Hz, 1H, $OCH_aH_bCH_3$), 3.26 (dq, J = 9.1, 7.0 Hz, 1H, $OCH_aH_bCH_3$), 2.96 – 2.84 (m, 2H, $COCHCH_aH_b$,CH_aH_bCO), 2.29 (dd, J = 18.4, 2.9 Hz, 1H, CH_aH_bCO), 1.46 – 1.31 (m, 1H, $COCHCH_aH_b$), 1.00 (t, J = 7.0 Hz, 3H, OCH_2CH_3).

$^{13}C\{^1H\}$ NMR (101 MHz, $CDCl_3$) δ 208.5 (C), 185.7 (C), 177.3 (C), 172.1 (C), 137.9 (C), 131.0 (C), 128.6 (CH), 128.5 (2 × CH), 75.2 (CH), 66.5 (CH_2), 66.0 (CH_2), 49.5 (CH), 47.6 (CH_2), 42.8 (CH_2), 40.7 (CH), 31.6 (CH_2), 15.4 (CH_3).

HRMS (+ESI) m/z calcd. for $[M + Na]^+$ $C_{20}H_{21}NNaO_4S$: 394.1084; found: 394.1100. m/z calcd. for $[M – OEt]^+$ $C_{18}H_{16}NO_3S$: 326.0845; found: 326.0858.

Chiral HPLC (Phenomenex Lux® Cellulose-5 column, 40% iPrOH in hexanes, flow rate 1 mL/min) Rt 15.3 min (4R,5R,6S) enantiomer, Rt 17.6 min (4S,5S,6R) enantiomer, 98:2 e.r.

Bicycle 10b

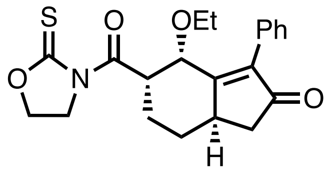

According to General Procedure H, the title compound was obtained from N-(5-hexenoyl)-1,3-oxazolidine-2-thione (**1i**, 100 mg, 0.50 mmol, 1.0 equiv.) and hexacarbonyl μ-[η^4-(1,1-diethoxy-3-phenylpropyne)dicobalt] (**F**, 270 mg, 0.55 mmol, 1.1 equiv.) at 0 °C for 16 h, followed by the addition of NMO·H_2O (228 mg, 2.50 mmol, 5.0 equiv.). The reaction mixture was stirred for an additional 24 h at r.t.The analysis of the crude by HPLC-MS established the formation of a sole diastereomer.

The residue was purified by column chromatography (Hex/EtOAc, from 60:40 to 35:65) to afford 47 mg of pure **10b** (0.13 mmol, 51% yield).

Light brown solid.
Mp 52–54 °C.
R_f 0.3 (Hex/EtOAc, 60:40).
$[\alpha]_D^{25}$ + 68 (c 1.0, $CHCl_3$).
IR (ATR) ν 2920, 2851, 1733, 1700, 1653, 1444, 1370, 1239, 1206, 1170, 1156 cm^{-1}.
^{1}H NMR (400 MHz, $CDCl_3$) δ 7.45 – 7.27 (m, 5H, Ar<u>H</u>), 5.03 (d, J = 2.8 Hz, 1H, C<u>H</u>OEt), 4.82 – 4.73 (m, 1H, COC<u>H</u>CH_2), 4.52 (ddd, J = 9.5, 8.9, 5.6 Hz, 1H, OC<u>H_a</u>H_bCH_2), 4.41 (q, J = 9.1 Hz, 1H, OCH_a<u>H_b</u>CH_2), 4.24 (dt, J = 11.3, 9.5 Hz, 1H, NC<u>H_a</u>H_bCH_2), 4.06 (ddd, J = 11.3, 9.1, 5.6 Hz, 1H, NCH_a<u>H_b</u>CH_2), 3.39 (dq, J = 9.5, 7.0 Hz, 1H, OC<u>H_a</u>H_bCH_3), 3.13 (dq, J = 9.5, 7.0 Hz, 1H, OCH_a<u>H_b</u>CH_3), 3.10 – 3.01 (m, 1H, CH_2C<u>H</u>CH_2CO), 2.77 (dd, J = 19.1, 6.7 Hz, 1H, C<u>H_a</u>H_bCO), 2.47 – 2.25 (m, 2H, COCHC<u>H_a</u>H_bCH_2, $COCHCH_2C$<u>H_a</u>H_b), 2.17 (dd, J = 19.1, 2.2 Hz, 1H, C<u>H_a</u><u>H_b</u>CO), 1.85 (dq, J = 15.2, 4.2 Hz, 1H, $COCHCH_a$<u>H_b</u>CH_2), 1.37 – 1.26 (m, 1H, $COCHCH_2CH_a$<u>H_b</u>), 1.00 (t, J = 7.0 Hz, 3H, OCH_2C<u>H_3</u>).
$^{13}C\{^1H\}$ NMR (101 MHz, $CDCl_3$) δ 206.9 (C), 185.2 (C), 172.8 (C), 171.1 (C), 141.6 (C), 130.5 (C), 129.3 (CH), 128.5 (CH), 128.3 (CH), 71.3 (CH), 66.4 (CH_2), 64.4 (CH_2), 47.8 (CH_2), 47.6 (CH), 41.5 (CH_2), 35.6 (CH), 33.1 (CH_2), 22.3 (CH_2), 15.1 (CH_3).
HRMS (+ESI) m/z calcd. for $[M + Na]^+$ $C_{21}H_{23}NNaO_4S$: 408.1240; found: 408.1257. m/z calcd. for $[M – OEt]^+$ $C_{19}H_{18}NO_3S$: 340.1002; found: 340.1015.

Chiral HPLC (Phenomenex Lux® Cellulose-5 column, 40% iPrOH in hexanes, flow rate 1 mL/min) Rt 17.6 min (4R,5R,6S) enantiomer, Rt 18.9 min (4S,5S,6R) enantiomer, 98:2 e.r.

8. Derivatizations

8.1. Reduction of the triple bond.

Methyl (2S,3R)-3-ethoxy-2-methyl-5-phenylpentanoate (8a)

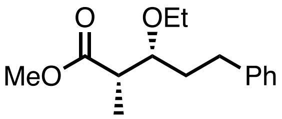

A 0.15 M solution of **6a** (99 mg, 0.4 mmol, 1.0 equiv.) and 10% Pd/C (43 mg, 40 μmol, 10 mol%) in MeOH (2.5 mL) was bubbled with H_2 for 10 min. The round-bottom flask was equipped with a H_2 balloon and the solution was stirred overnight at r.t..

Afterwards, N_2 was bubbled through the solution for 10 min and it was then filtered through a pad of Celite® to give **8a** as a pure product (90 mg, 90% yield).

Colorless oil.
$[\alpha]_D^{25}$ + 14.6 (c 1.0, $CHCl_3$).
IR (ATR) ν 3025, 2948, 2878, 1733, 1497, 1456, 1435, 1372, 1247, 1198, 1165 cm^{-1}.
^{1}H NMR (400 MHz, $CDCl_3$) δ 7.32 – 7.23 (m, 2H, Ar$\underline{H}$), 7.22 – 7.13 (m, 3H, Ar$\underline{H}$), 3.68 (s, 3H, OC$\underline{H}_3$), 3.61 – 3.45 (m, 3H, C$\underline{H}$OEt, OC$\underline{H}_a$H$_b$CH$_3$, OCH$_a\underline{H}_b$CH$_3$), 2.79 (ddd, J = 13.6, 10.1, 5.7 Hz, 1H, C$\underline{H}_a$H$_b$CH$_2$Ph), 2.71 – 2.55 (m, 2H, CH$_a\underline{H}_b$CH$_2$Ph, COC$\underline{H}$CH$_3$), 1.90 – 1.70 (m, 2H, C$\underline{H}_a$H$_b$Ph, CH$_a\underline{H}_b$Ph), 1.24 – 1.13 (m, 6H, COC$\underline{H}_3$, OCH$_2$C$\underline{H}_3$).
^{13}C{^{1}H} NMR (101 MHz, $CDCl_3$) δ 175.6 (C), 142.2 (C), 128.5 (CH × 2), 125.9 (CH), 80.1 (CH), 65.9 (CH_2), 51.7 (CH), 43.7 (CH), 34.6 (CH_2), 32.0 (CH_2), 15.7 (CH_3), 12.4 (CH_3).
HRMS (+ESI) m/z calcd. for $[M – OEt]^+$ $C_{13}H_{17}O_2$: 205.1223; found: 205.1225. m/z calcd. for $[M + H]^+$ $C_{15}H_{23}O_3$: 251.1642; found: 251.1643.

Methyl (2S,3R,Z)-3-ethoxy-2-methyl-5-phenyl-4-pentenoate (9a)

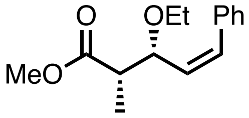

A 0.1 M solution of **6a** (99 mg, 0.4 mmol, 1.0 equiv.), 5% Lindlar catalyst (43 mg, 20 μmol, 5 mol%) and quinoline (55 μL, 0.46 mmol, 1.15 equiv.) in EtOAc (4 mL) was bubbled with H_2 for 10 min. The round-bottom flask was equipped with a H_2 balloon and the solution was stirred overnight at r.t..

Afterwards, N_2 was bubbled through the solution for 10 min, and it was then filtered through a pad of Celite®. The residue was purified via column chromatography (Hex/Et_2O from 100:0 to 90:10) to give 95 mg (96% yield) of pure **9a**.

Colorless oil.
R_f 0.3 (Hex/Et_2O, 90:10)
$[\alpha]_D^{25}$ – 54.0 (c 1.0, $CHCl_3$).
IR (ATR) ν 2058, 2974, 2948, 2876, 1735, 1493, 1457, 1435, 1195, 1165, 1094 cm^{-1}.
^{1}H NMR (400 MHz, $CDCl_3$) δ 7.42 – 7.21 (m, 5H, ArH), 6.69 (d, J = 11.9 Hz, 1H, CH=CHPh), 5.62 (dd, J = 11.9, 9.7 Hz, 1H, CH=CHPh), 4.58 – 4.49 (m, 1H, CHOEt), 3.64 (s, 3H, OCH_3), 3.47 (dq, J = 9.4, 7.0 Hz, 1H, $OCH_aH_bCH_3$), 3.19 (dq, J = 9.4, 7.0 Hz, 1H, $OCH_aH_bCH_3$), 2.78 – 2.66 (m, 1H, $COCHCH_3$), 1.25 (d, J = 7.0 Hz, 3H, $COCHCH_3$), 1.06 (t, J = 7.0 Hz, 3H, OCH_2CH_3).
^{13}C{^{1}H} NMR (101 MHz, $CDCl_3$) δ 174.7 (CO), 136.7 (C), 133.2 (CH), 131.0 (CH), 128.9 (CH), 128.4 (CH), 127.3 (CH), 75.3 (CH), 63.9 (CH_2), 51.7 (CH_3), 45.0 (CH), 15.2 (CH_3), 12.5 (CH_3).
HRMS (+ESI) m/z calcd. for $[M – OEt]^+$ $C_{13}H_{15}O_2$: 203.1067; found: 203.1064. m/z calcd. for $[M + Na]^+$ $C_{15}H_{20}O_3Na$: 271.1305; found: 271.1301.

8.2. Removal of the heterocycle.

(2S,3S)-3-Ethoxy-2-methyl-5-phenyl-N-((S)-1-phenylethyl)-4-pentynamide (7a)

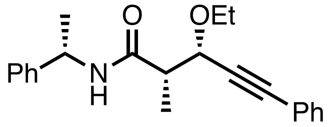

(S)-(−)-α-Methylbenzylamine (36 μL, 0.28 mmol, 1.5 equiv.) was added to a solution of **4a** (59 mg, 0.19 mmol, 1.0 equiv.) and DMAP (4.54 mg, 0.04 mmol, 20 mol%) in DCM (1.5 mL) and stirred for 2 h at 0 °C.

The solution was concentrated under reduced pressure and the resulting crude purified by column chromatography (DCM/EtOAc, from 100:0 to 90:10) to afford 55 mg (0.16 mmol, 86% yield) of amide **7a**, recovering also 20 mg of 1,3-oxazolidine-2-thione (**1**, 0.13 mmol, 67% yield).

White solid.

Mp 120–123 °C.

R$_f$ 0.4 (Hex/EtOAc, 70:30).

[α]$_D$^{20} – 153 (c 1.0, $CHCl_3$).

IR (ATR) ν 3278, 2055, 2982, 2964, 2930, 2866, 2111, 2093, 1634, 1548, 1488, 1441, 1372, 1264, 1085, 1055, 1011 cm^{-1}.

1H NMR (400 MHz, CDCl$_3$) δ 7.36 – 7.22 (m, 7H, Ar<u>H</u>), 7.20 – 7.11 (m, 3H, Ar<u>H</u>), 6.88 (d, J = 7.8 Hz, 1H, N<u>H</u>), 5.14 (p, J = 7.1 Hz, 1H, C<u>H</u>NH), 4.44 (d, J = 4.8 Hz, 1H, C<u>H</u>OEt), 3.92 (dq, J = 9.3, 7.0 Hz, 1H, OC<u>H</u>$_a$H$_b$CH$_3$), 3.55 (dq, J = 9.3, 7.0 Hz, 1H, OCH$_a$<u>H</u>$_b$CH$_3$), 2.75 (qd, J = 7.2, 4.8 Hz, 1H, COC<u>H</u>), 1.48 (d, J = 7.1 Hz, 3H, C<u>H</u>$_3$CHNH), 1.31 (d, J = 7.2 Hz, 3H, COCHC<u>H</u>$_3$), 1.24 (t, J = 7.0 Hz, 3H, OCH$_2$C<u>H</u>$_3$).

^{13}C{^{1}H} NMR (101 MHz, CDCl$_3$) δ 171.9 (C), 143.7 (C), 132.0 (CH), 128.6 (CH), 128.6 (CH), 128.3 (CH), 127.0 (CH), 126.2 (CH), 122.4 (C), 87.3 (C), 85.8 (C), 71.9 (CH), 65.0 (CH_2), 48.7 (CH), 45.7 (CH), 22.4 (CH_3), 15.2 (CH_3), 13.4 (CH_3).

HRMS (+ESI) m/z calcd. for $[M + Na]^+$ $C_{22}H_{25}NNaO_2$: 358.1778; found: 358.1793. m/z calcd. for $[M + H]^+$ $C_{22}H_{26}NO_2$: 336.1958; found: 336.1971.

Bicycle 11a

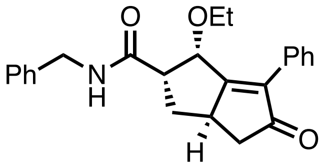

Benzylamine (12 µL, 109 µmol, 1.5 equiv.) was added to a solution of **10a** (27.0 mg, 73 µmol, 1.0 equiv.) and DMAP (2.1 mg, 15 µmol, 20 mol%) in DCM (0.5 mL) at 0 °C and stirred for 16 h at r.t.

The reaction mixture was partitioned in DCM (10 mL) and water (10 mL). The organic layer was separated and the aqueous layer extracted with DCM (2 × 10 mL). The combined organic extracts were washed with 0.5 M NaOH (10 mL) and 0.5 M HCl (10 mL), dried over $MgSO_4$, filtered and concentrated under reduced pressure. The resulting crude was purified by column chromatography (Hex/EtOAc, from 60:40 to 30:70) to afford 24 mg (63 µmol, 87% yield) of pure amide **11a**.

White solid.

Mp 190–192 °C.

R$_f$ 0.2 (Hex/EtOAc, 1:1).

$[\alpha]_D^{25}$ + 181 (c 0.8, $CHCl_3$).

IR (ATR) ν 3245, 3084, 2954, 2920, 2885, 2853, 2343, 1696, 1664, 1638, 1560, 1497, 1454, 1377, 1355, 1306, 1239, 1113, 1090, 1068, 1034 cm^{-1}.

^1H NMR (400 MHz, $CDCl_3$) δ 7.48 – 7.27 (m, 10H, Ar$\underline{H}$), 6.85 (s broad, 1H, $PhCH_2N\underline{H}$), 4.80 (d, J = 4.7 Hz, 1H, $C\underline{H}OEt$), 4.56 – 4.41 (m, 2H, $PhC\underline{H}_2NH$), 3.66 – 3.49 (m, 2H, $OC\underline{H}_2CH_3$), 3.42 (dtd, J = 10.6, 7.1, 3.4 Hz, 1H, $CH_2C\underline{H}CH_2CO$), 3.02 – 2.91 (m, 2H, $COC\underline{H}$, $C\underline{H}_aH_bCO$), 2.62 (ddd, J = 13.3, 10.6, 7.7 Hz, 1H, $COCHC\underline{H}_aH_b$), 2.30 (dd, J = 18.1, 3.4 Hz, 1H, $CH_a\underline{H}_bCO$), 1.80 (ddd, J = 13.3, 10.1, 7.1 Hz, 1H, $COCHCH_a\underline{H}_b$), 1.13 (t, J = 7.0 Hz, 3H, $OCH_2C\underline{H}_3$).

$^{13}C\{^1H\}$ NMR (101 MHz, $CDCl_3$) δ 208.3 (C), 177.3 (C), 171.1 (C), 138.4 (C), 138.0 (C), 130.7 (C), 128.8 (CH), 128.8 (CH), 128.7 (CH), 128.6 (CH), 127.9 (CH), 127.6 (CH), 76.4 (CH), 65.9 (CH_2), 50.3 (CH), 44.7 (CH_2), 43.8 (CH_2), 38.7 (CH), 32.1 (CH_2), 15.4 (CH_3).

HRMS (+ESI) m/z calcd. for $[M + Na]^+$ $C_{24}H_{25}NNaO_3$: 398.1727; found: 398.1739. m/z calcd. for $[M + H]^+$ $C_{24}H_{26}NO_3$: 376.1907; found: 376.1919. m/z calcd. for $[M - OEt]^+$ $C_{22}H_{20}NO_2$: 330.1489; found: 330.1498.

9. Crystal Information

9.1. Instrumentation

Both crystals were analyzed in the same way. The X-ray intensity data were measured at 100 K on a D8 Venture system equipped with a multilayer monochromator and a Mo microfocus (λ = 0.71073 Å). The frames were integrated with the Bruker SAINT software package using a narrow-frame algorithm.

Eventually, the structures were solved and refined using the Bruker SHELXTL Software Package.

9.2. Sample Preparation and Crystal Measurements

From (2S,3S)-3-ethoxy-2-methyl-5-phenyl-N-[(S)-1-phenylethyl]-4-pentynamide **(7a)**

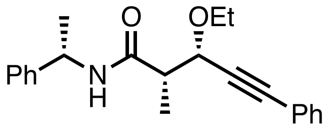

CCDC 2538574

Single crystals of **7a** suitable for X-ray diffraction analysis were obtained by slow evaporation of a DCM/hexanes (1:2) solution at room temperature over several days.

Table 1. Sample and crystal data for 7a

Chemical formula	$C_{22}H_{25}NO_2$	
Formula weight	335.43 g/mol	
Temperature	100(2) K	
Wavelength	0.71073 Å	
Crystal system	monoclinic	
Space group	P 1 21 1	
Unit cell dimensions	a = 4.8169(3) Å	α = 90°
	b = 21.3881(16) Å	β = 103.480(2)°
	c = 9.2877(7) Å	γ = 90°
Volume	930.50(11) $Å^3$	
Z	2	
Density (calculated)	1.197 g/cm3	
Absorption coefficient	0.076 mm^{-1}	
F(000)	360	

Table 2. Data collection and structure refinement for 7a

Theta range for data collection	1.90 to 26.43°	
Index ranges	-5<=h<=5, -26<=k<=26, -11<=l<=11	
Reflections collected	16217	
Independent reflections	3668 [R(int)=0.0729]	
Refinement method	Full-matrix least-squares on F^2	
Refinement program	SHELXL-2019/1 (Sheldrick, 2019)	
Function minimized	Σ w(F_o^2 - F_c^2)^2	
Data / restraints / parameters	3668 / 1 / 302	
Goodness-of-fit on F^2	1.042	
Final R indices	3075 data; I>2σ(I)	R1 = 0.0514, wR2 = 0.1046
	all data	R1 = 0.0665, wR2 = 0.1111
Weighting scheme	w=1/[$\sigma^2(F_o^2)+(0.0420P)^2+0.2680P$] where P=($F_o^2+2F_c^2$)/3	
Absolute structure parameter	1.6(9)	
Largest diff. peak and hole	0.345 and -0.210 eÅ^{-3}	
R.M.S. deviation from mean	0.043 eÅ^{-3}	

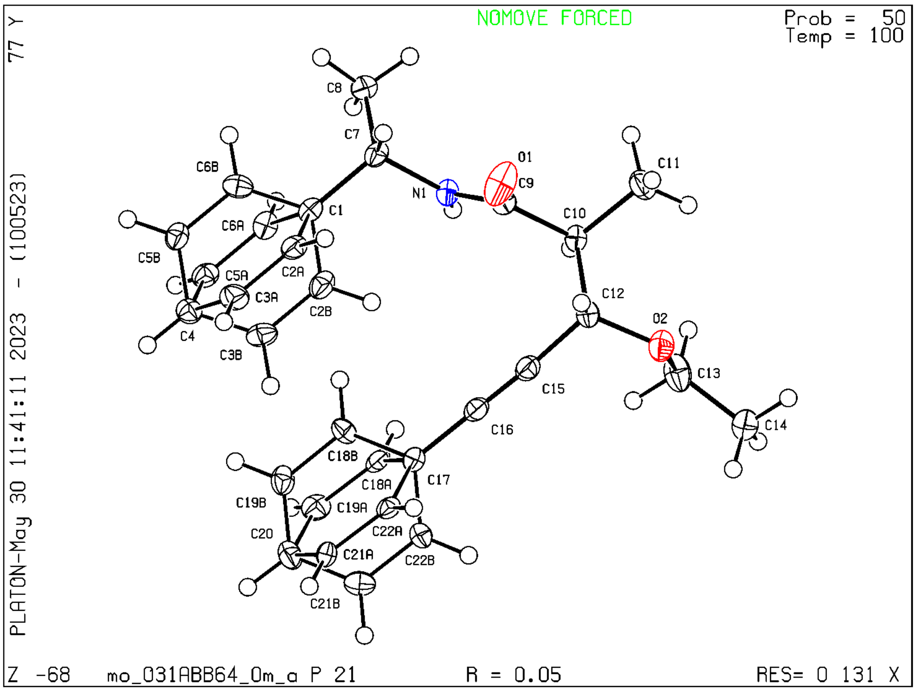

ORTEP diagram of 7a Showing Ellipsoid Contours of 50% Probability

From bicycle **11a**

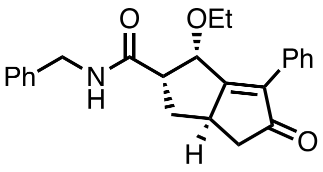

CCDC 2538575

Single crystals of **11a** suitable for X-ray diffraction analysis were obtained by slow evaporation of a DCM solution at room temperature over several days.

Table 3. Sample and crystal data for 11a

Chemical formula	$C_{48}H_{49}N_2O_6$	
Formula weight	749.89 g/mol	
Temperature	100(2) K	
Wavelength	0.71073 Å	
Crystal system	monoclinic	
Space group	P 1 21 1	
Unit cell dimensions	a = 8.8421(11) Å	α = 90°
	b = 24.650(16) Å	β = 94.185(4)°
	c = 8.9490(10) Å	γ = 90°
Volume	1945.3(4) $Å^3$	
Z	2	
Density (calculated)	1.280 g/cm^3	
Absorption coefficient	0.084 mm^{-1}	
F(000)	798	

Table 4. Data collection and structure refinement for 11a

Theta range for data collection	2.28 to 26.60°	
Index ranges	-11<=h<=11, -30<=k<=30, -11<=l<=10	
Reflections collected	49285	
Independent reflections	8008 [R(int)=0.1040]	
Refinement method	Full-matrix least-squares on F^2	
Refinement program	SHELXL-2019/1 (Sheldrick, 2019)	
Function minimized	Σ w(F_o^2 - F_c^2)^2	
Data / restraints / parameters	8008 / 1 / 494	
Goodness-of-fit on F^2	1.078	
Final R indices	7374 data; I>2σ(I)	R1 = 0.1223, wR2 = 0.3386
	all data	R1 = 0.1281, wR2 = 0.3433
Weighting scheme	w=1/[σ^2(F_o^2)+(0.1920P)^2+11.2058P] where P=(F_o^2+2F_c^2)/3	
Absolute structure parameter	–0.8 (5)	
Largest diff. peak and hole	1.384 and -0.516 eÅ^{-3}	
R.M.S. deviation from mean	0.180 eÅ^{-3}	

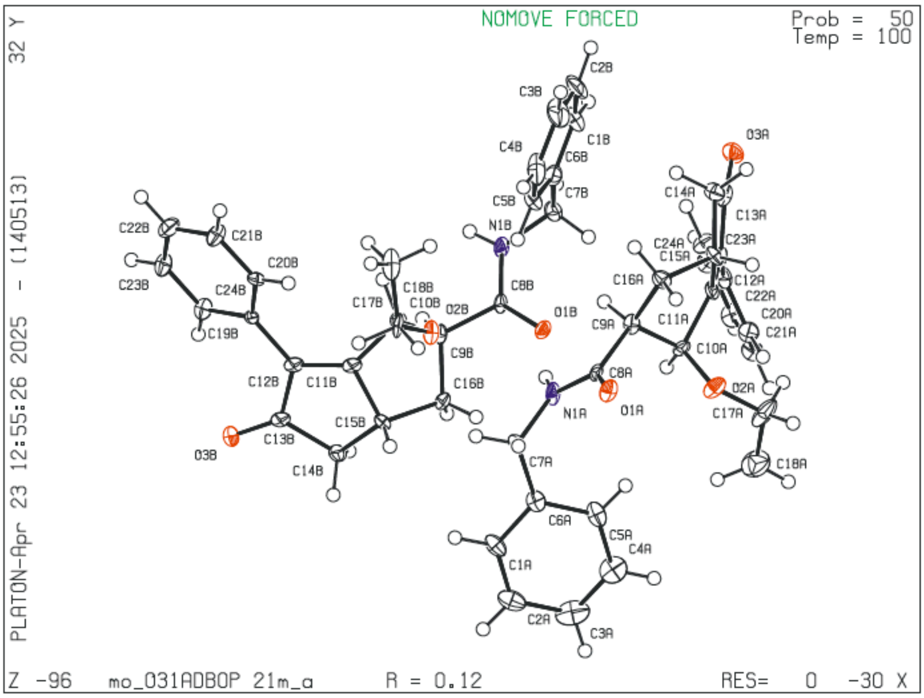

ORTEP diagram of 11a Showing Ellipsoid Contours of 50% Probability

10. References

(1) Mellado-Hidalgo, M.; Romero-Cavagnaro, E. A.; Nageswaran, S.; Puddu, S.; Kennington, S. C. D.; Costa, A. M.; Romea, P.; Urpí, F.; Aullón, G.; Font-Bardia, M. Protected Syn-Aldol Compounds from Direct, Catalytic, and Enantioselective Reactions of N-Acyl-1,3-Oxazinane-2-Thiones with Aromatic Acetals. Org. Lett. **2023**, 25, 659–664.

(2) Teloxa, S. F.; Kennington, S. C. D.; Camats, M.; Romea, P.; Urpí, F.; Aullón, G.; Font-Bardia, M. Direct, Enantioselective, and Nickel(II) Catalyzed Reactions of N-Azidoacetyl Thioimides with Trimethyl Orthoformate: A New Combined Methodology for the Rapid Synthesis of Lacosamide and Derivatives. Chem. Eur. J. **2020**, 26, 11540–11548.

(3) Liu, X.; Liu, R.; Dai, J.; Cheng, X.; Li, G. Application of Hantzsch Ester and Meyer Nitrile in Radical Alkynylation Reactions. Org. Lett. **2018**, 20, 6906–6909.

(4) Paley, R. S.; Hejna, B. G.; Quevedo, R. E.; Wong, A. R.; Dow, N. W.; Murphy, J. S.; Choi, S.; Yu, M.; Rablen, P. R.; Pike, R. D. N-Oxazolidinoyl Diene Iron(0) Tricarbonyl Complexes: Preparation by Diastereoselective Complexation and Synthetic Applications. Organometallics **2022**, 41, 2188–2219.

11. NMR Spectra

^{1}H NMR (400 MHz, $CDCl_3$)

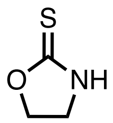

A

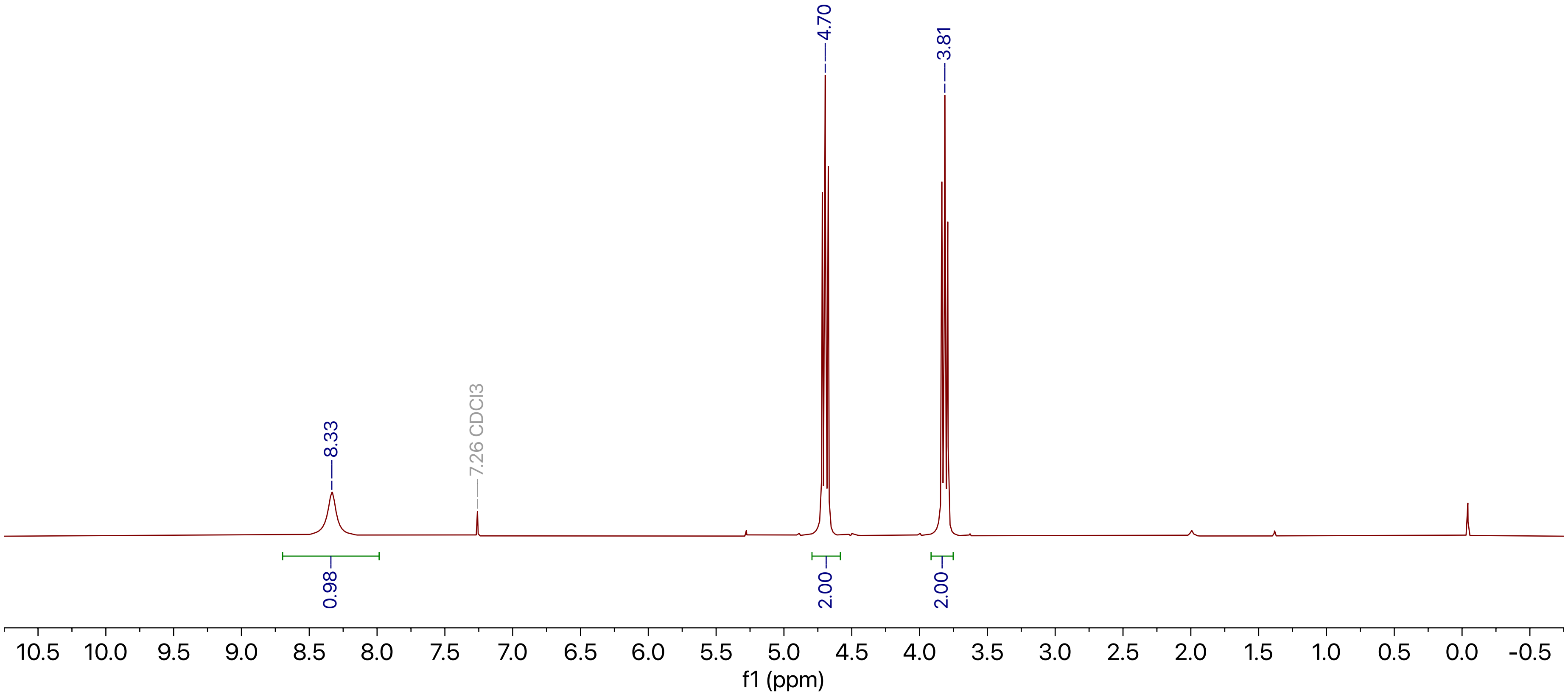

$^{13}C\{^{1}H\}$ NMR (101 MHz, $CDCl_3$)

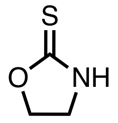

A

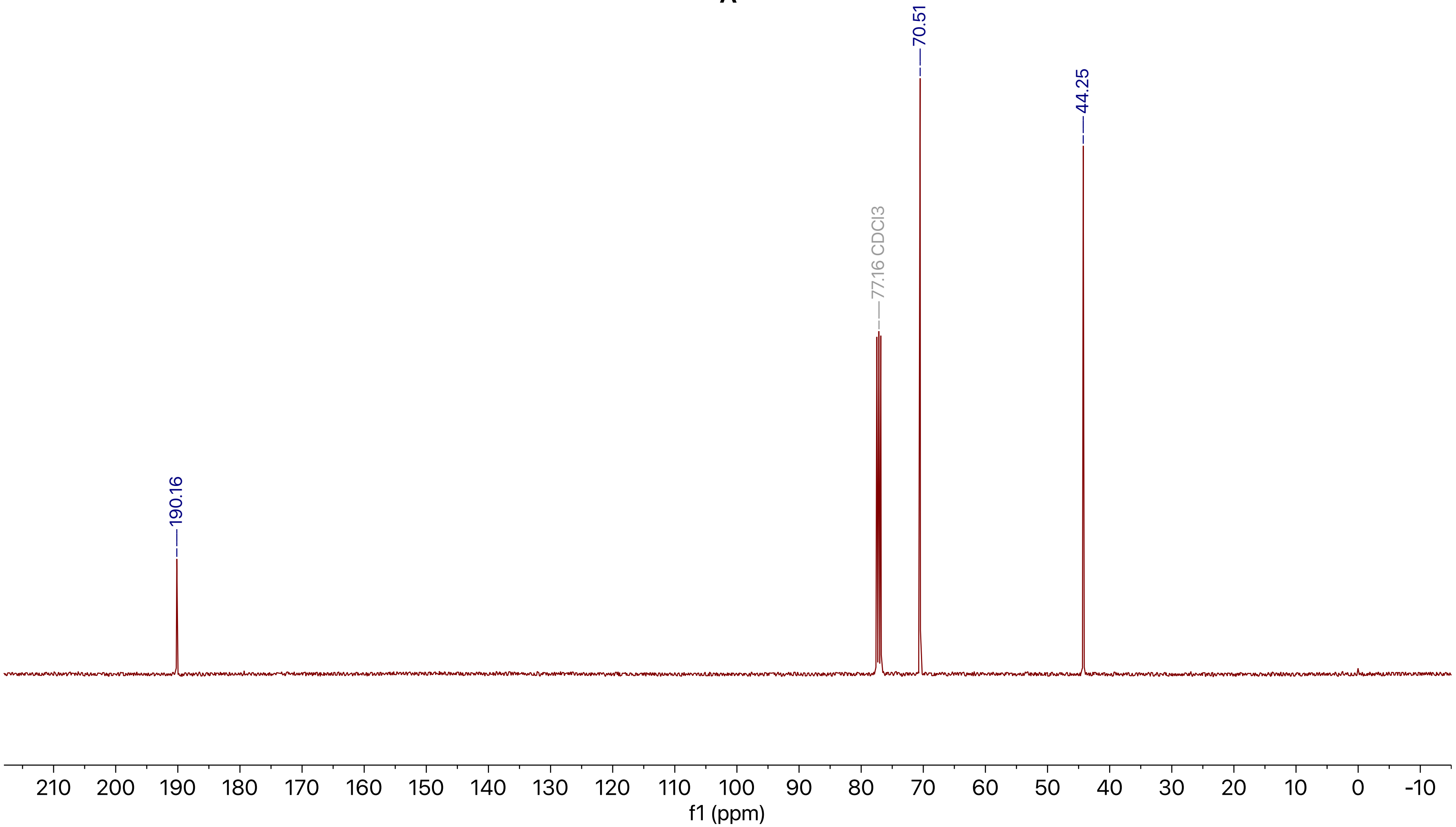

2D ^1H–^1H COSY (400 MHz, $CDCl_3$)

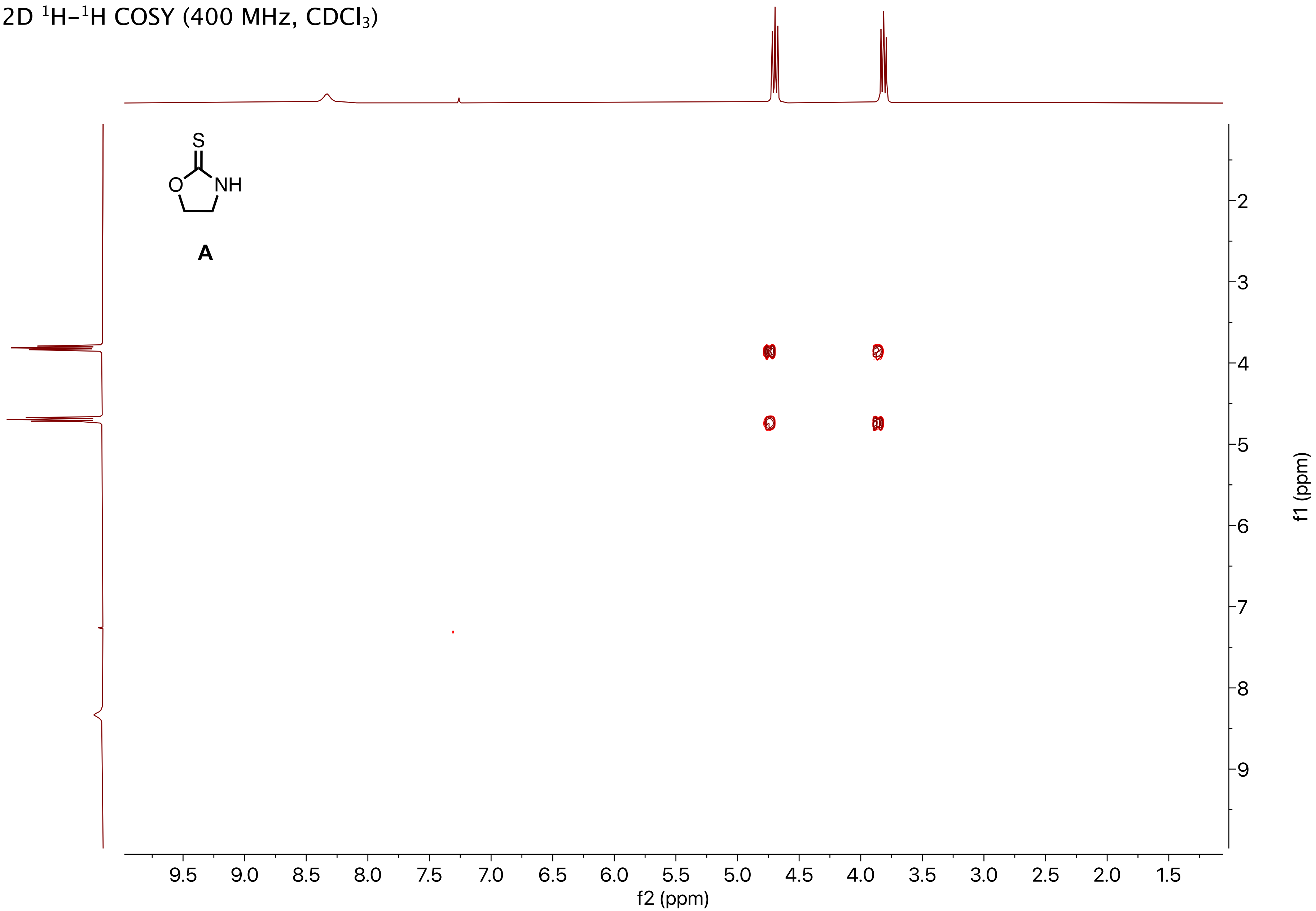

2D ^{1}H–^{13}C HSQC (400 MHz, $CDCl_3$)

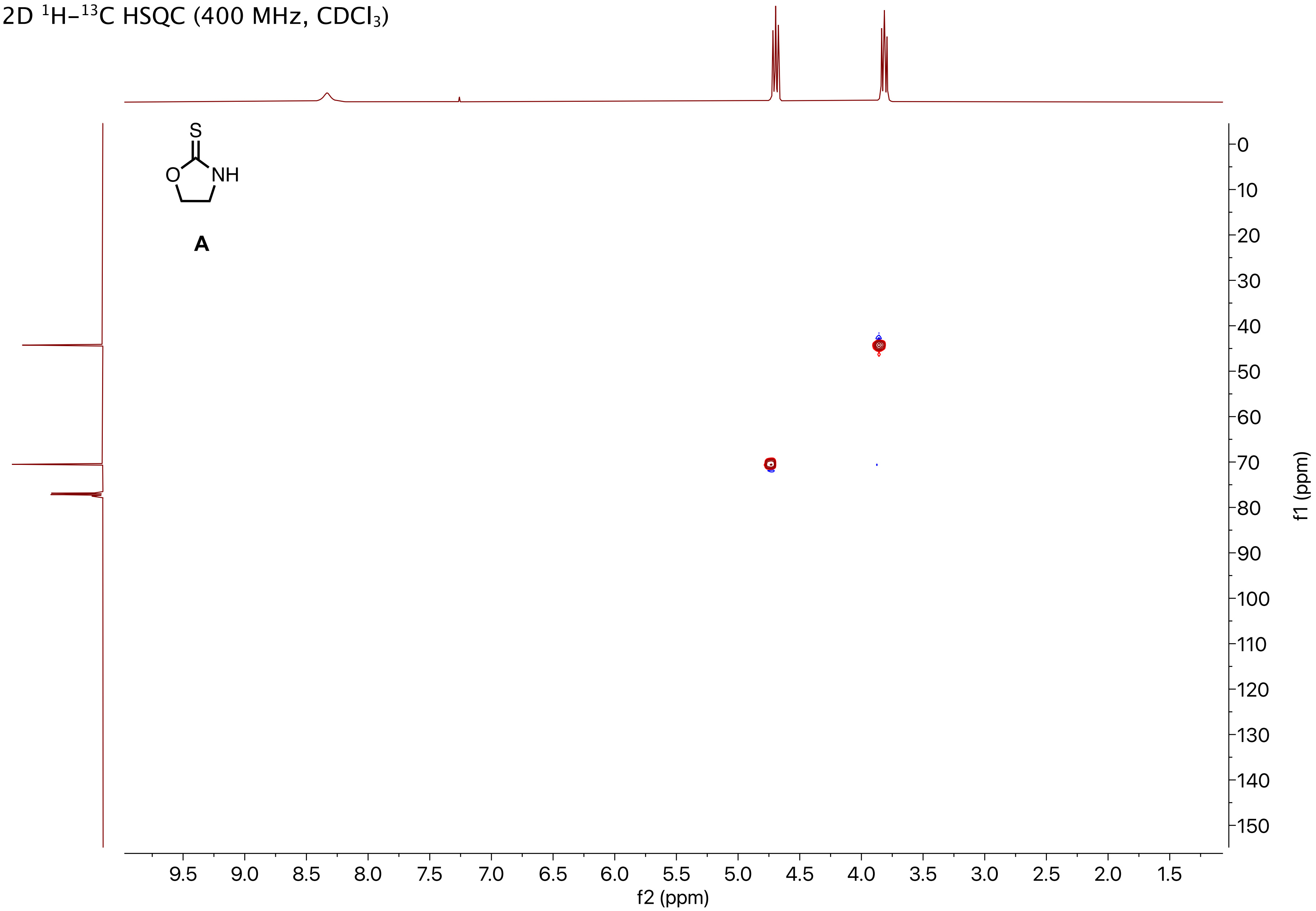

^{1}H NMR (400 MHz, $CDCl_3$)

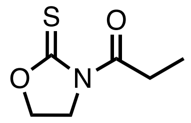

1a

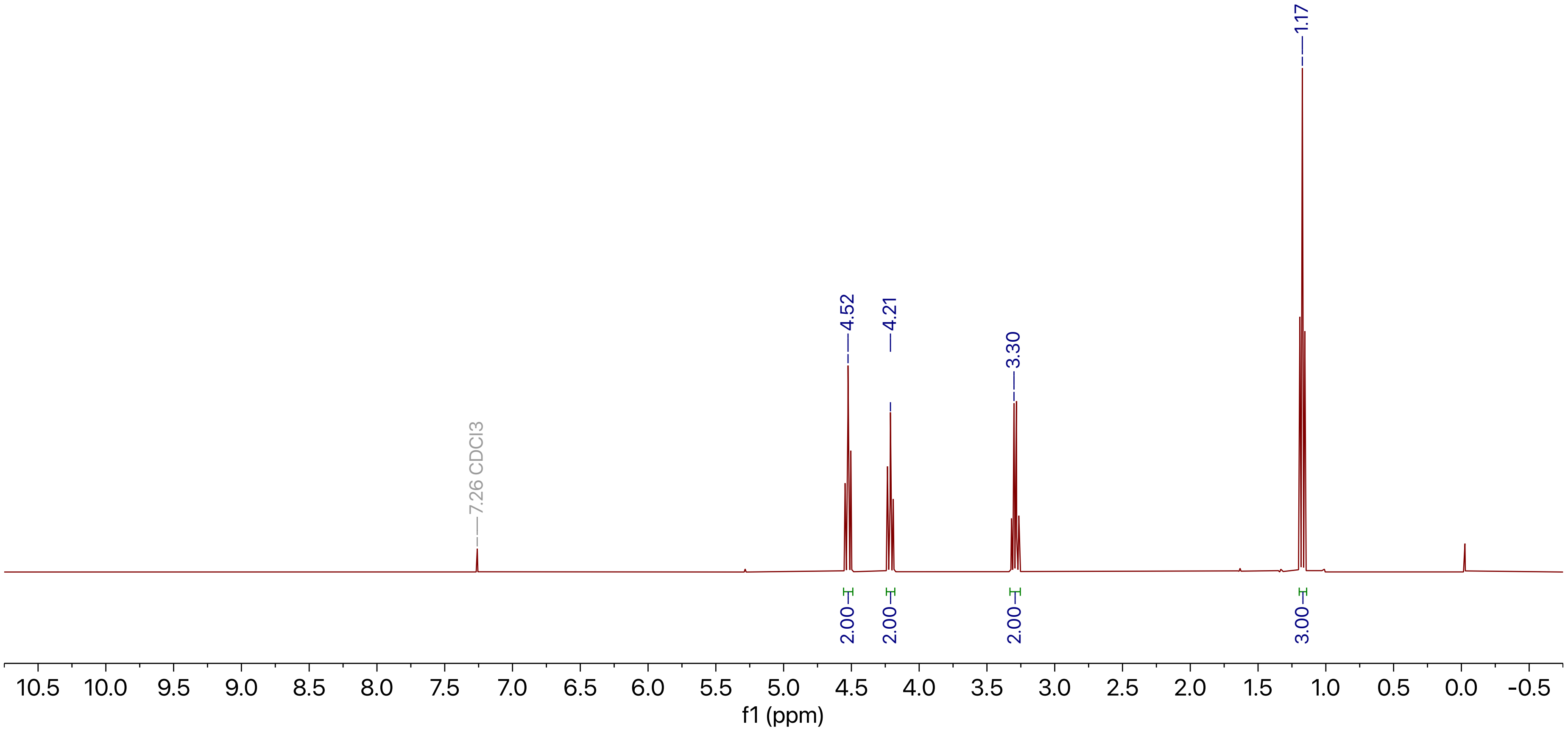

$^{13}C\{^{1}H\}$ NMR (101 MHz, $CDCl_3$)

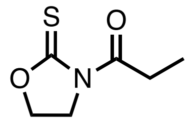

1a

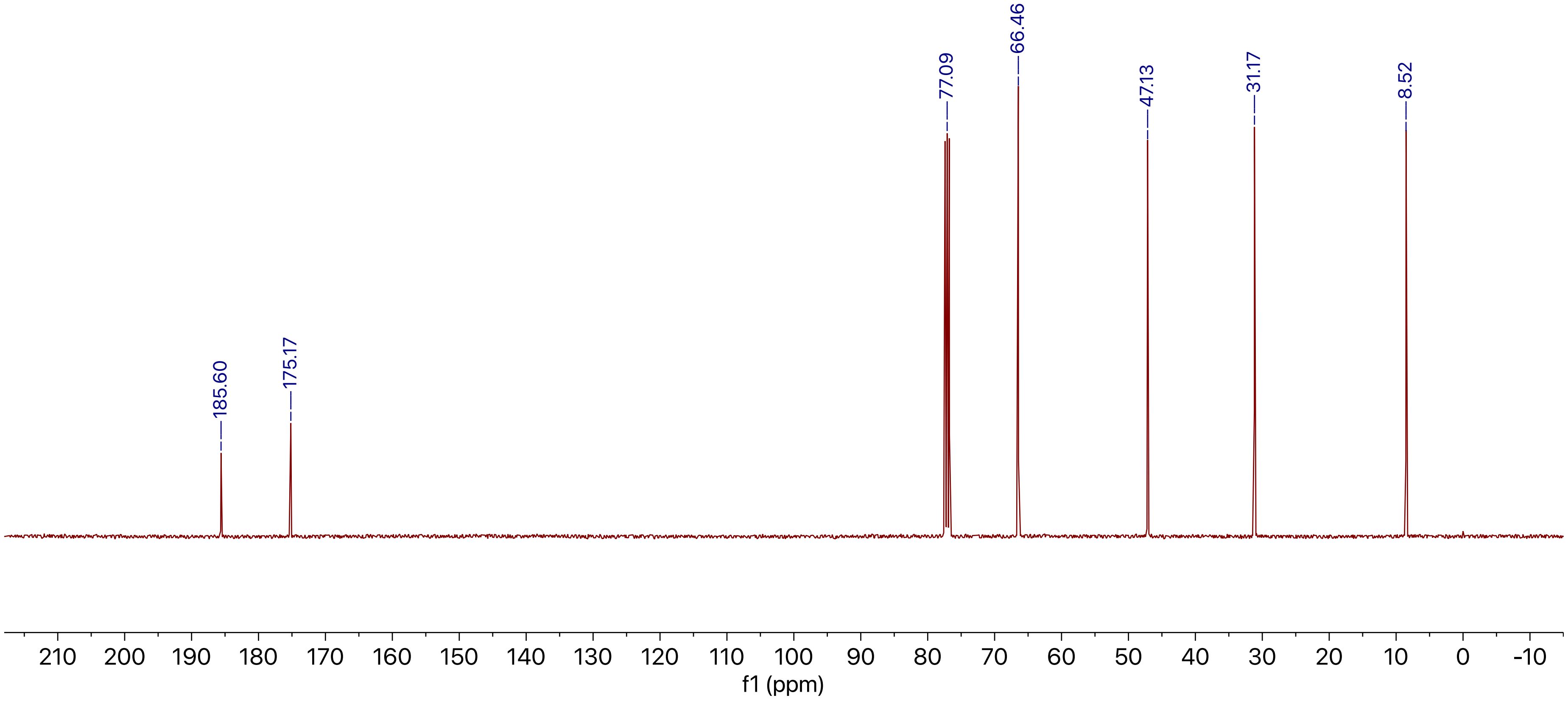

2D ^1H–^1H COSY (400 MHz, $CDCl_3$)

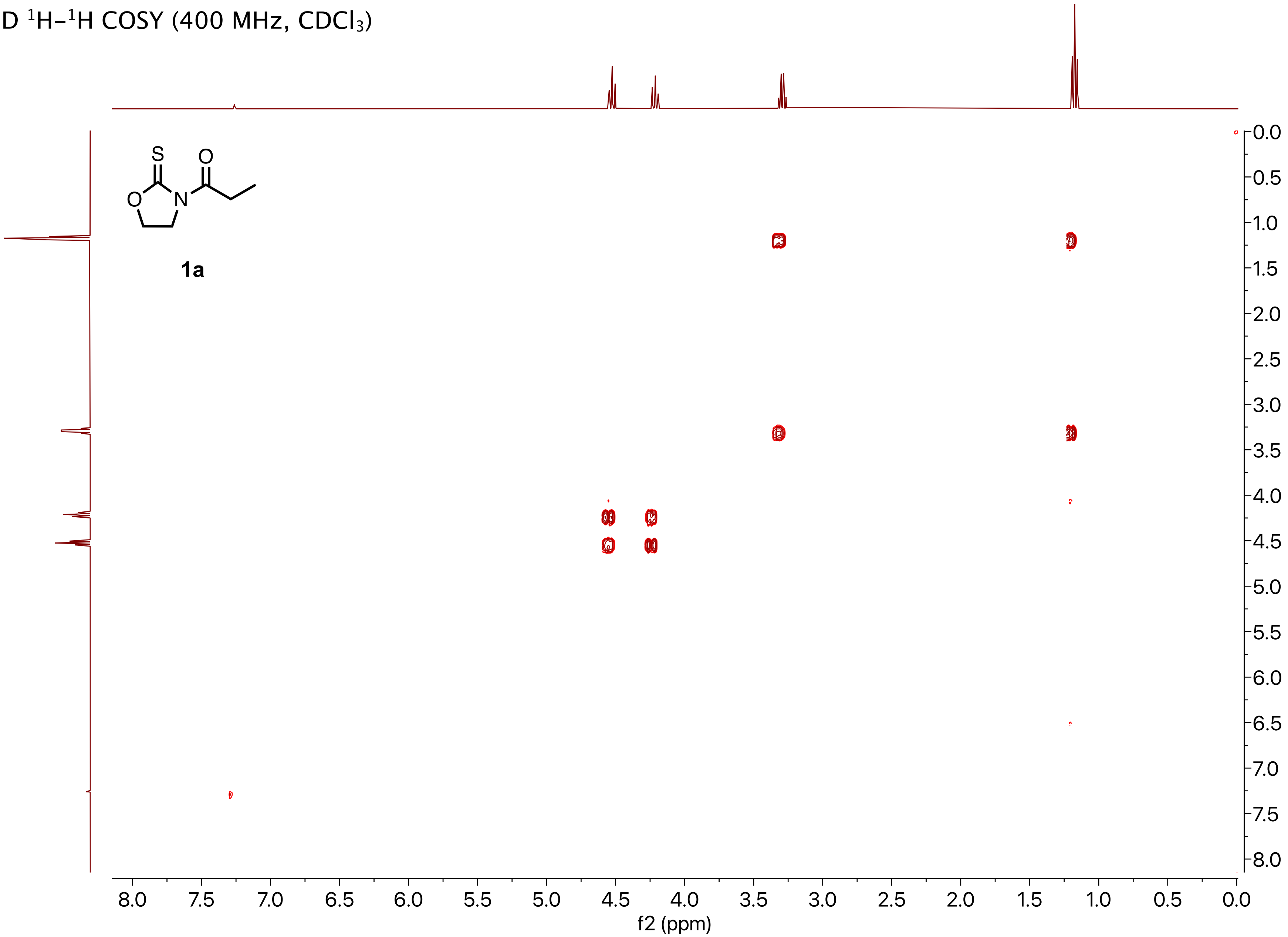

1a

2D $^{1}H-^{13}C$ HSQC (400 MHz, $CDCl_3$)

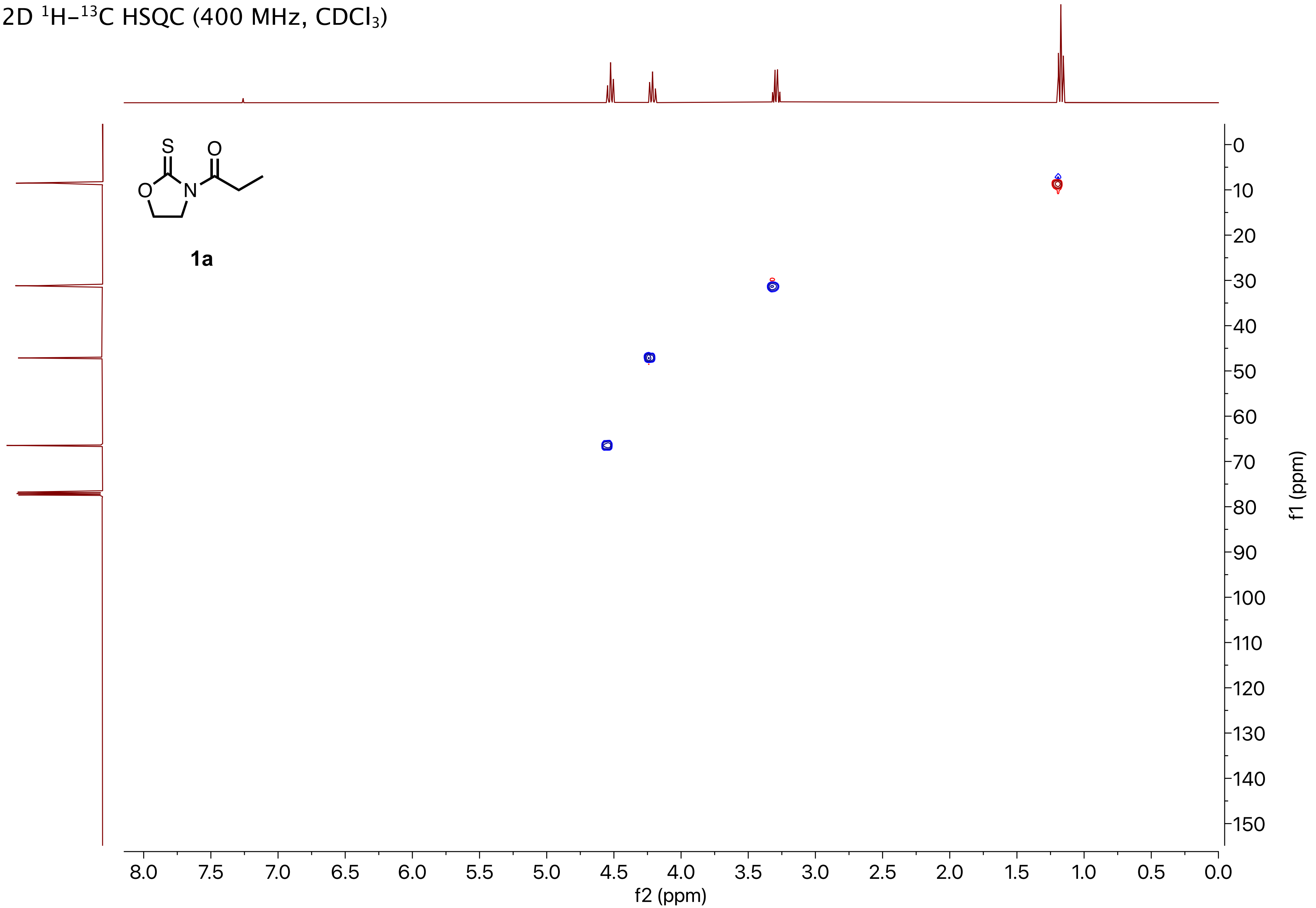

^{1}H NMR (400 MHz, $CDCl_3$)

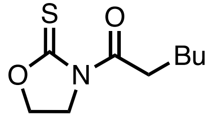

1b

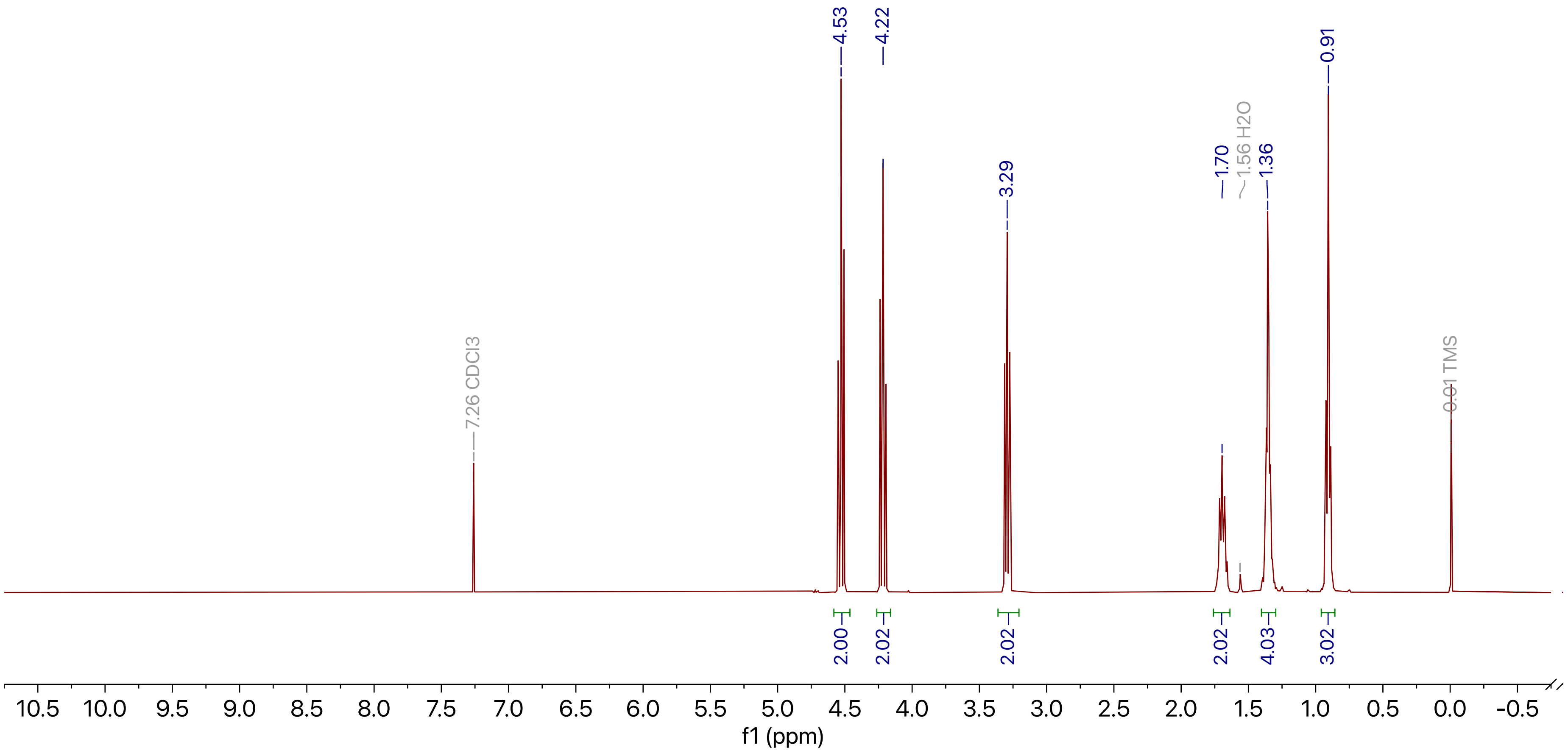

$^{13}C\{^{1}H\}$ NMR (101 MHz, $CDCl_3$)

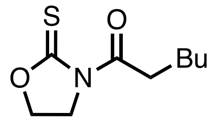

1b

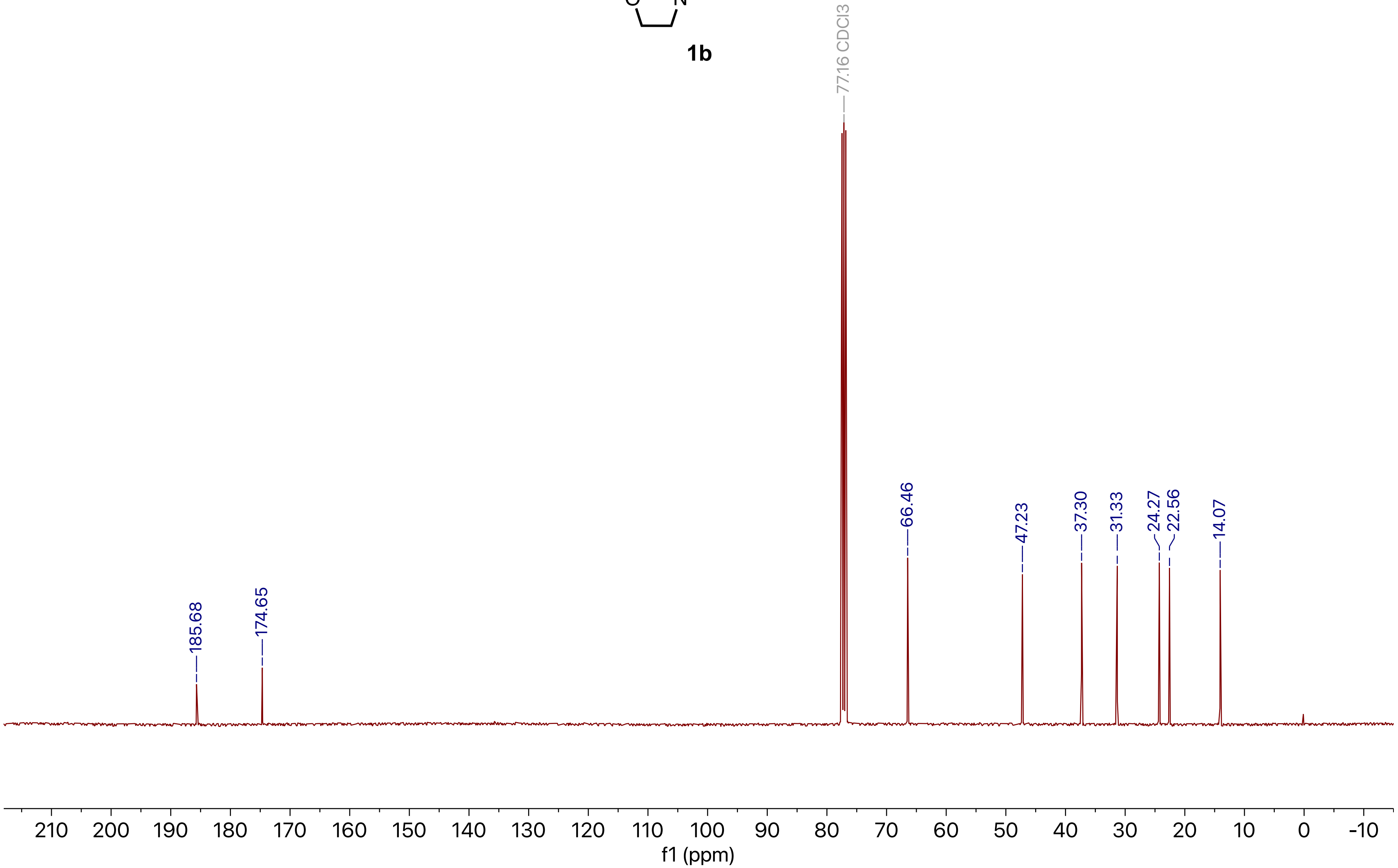

2D ^1H–^1H COSY (400 MHz, $CDCl_3$)

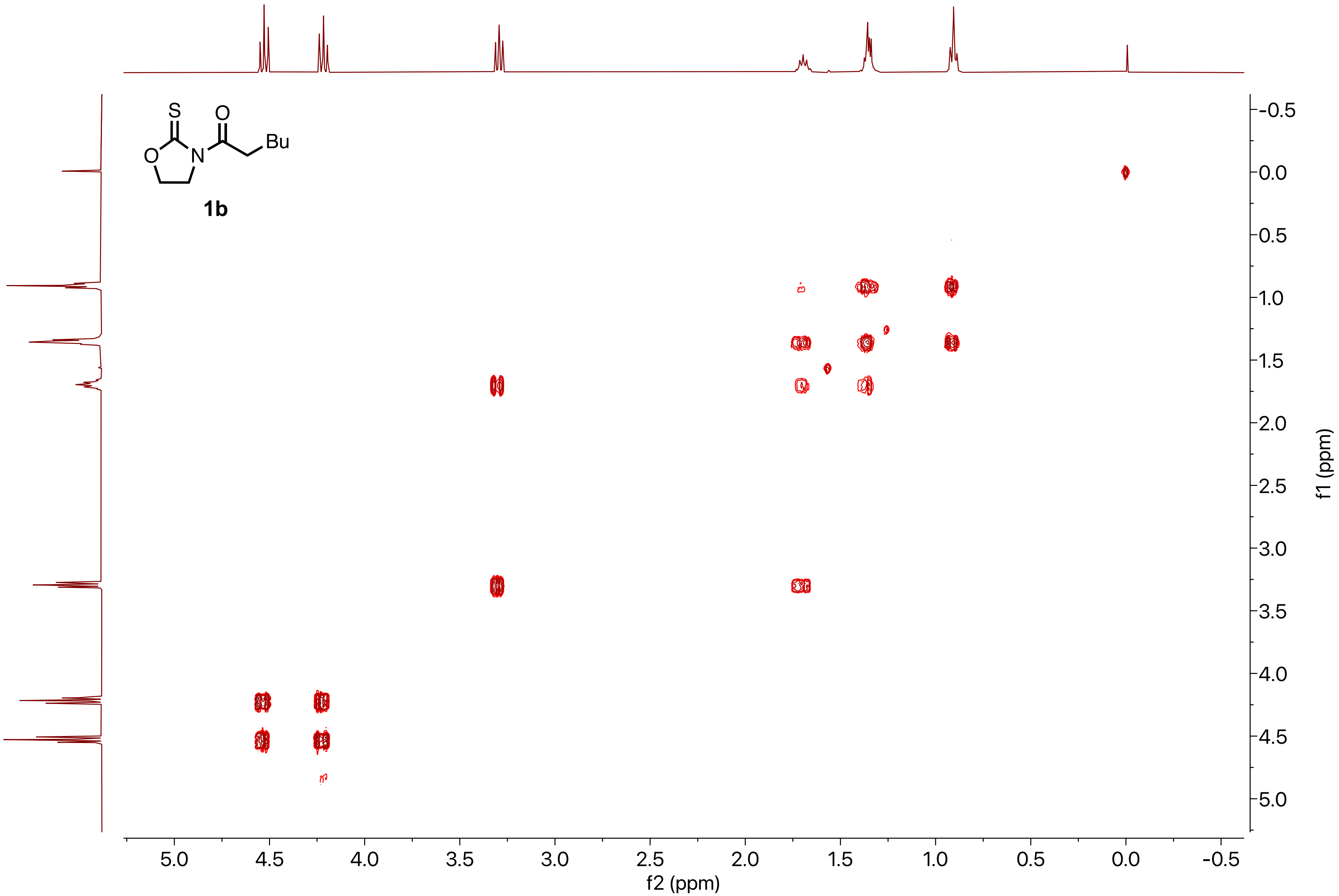

2D ^{1}H–^{13}C HSQC (400 MHz, $CDCl_3$)

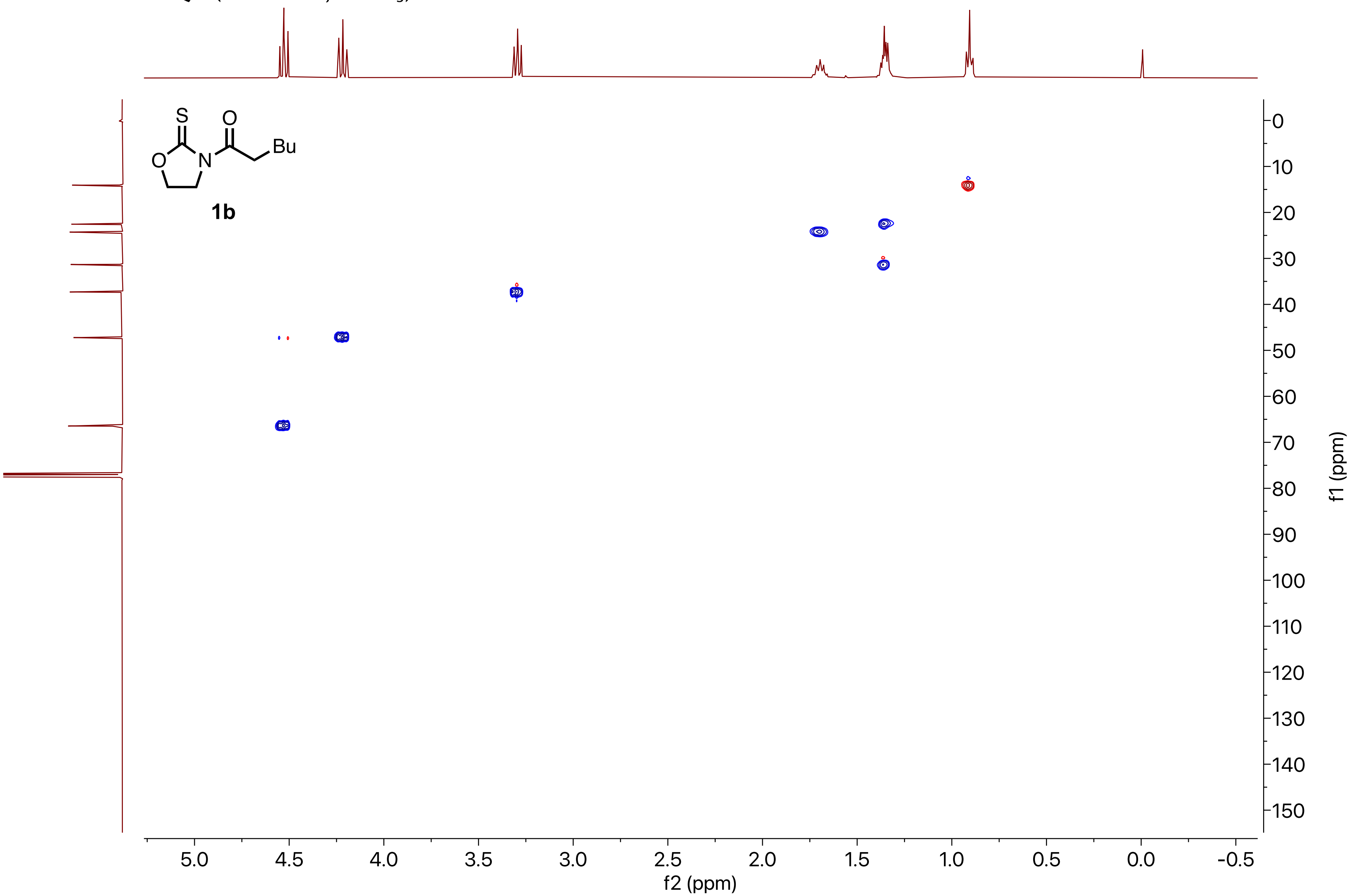

^{1}H NMR (400 MHz, $CDCl_3$)

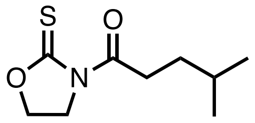

1c

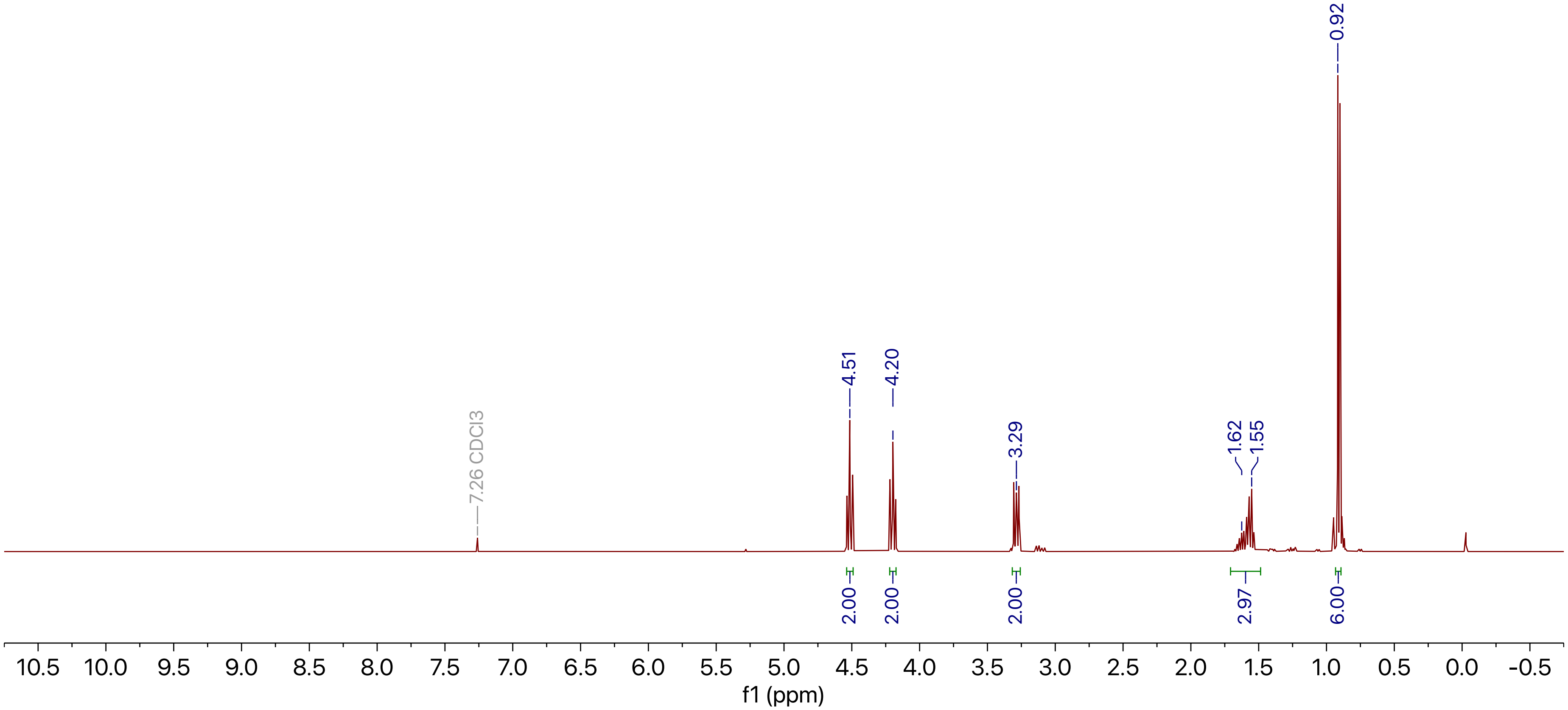

$^{13}C\{^{1}H\}$ NMR (101 MHz, $CDCl_3$)

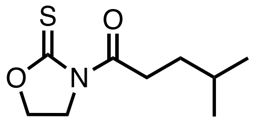

1c

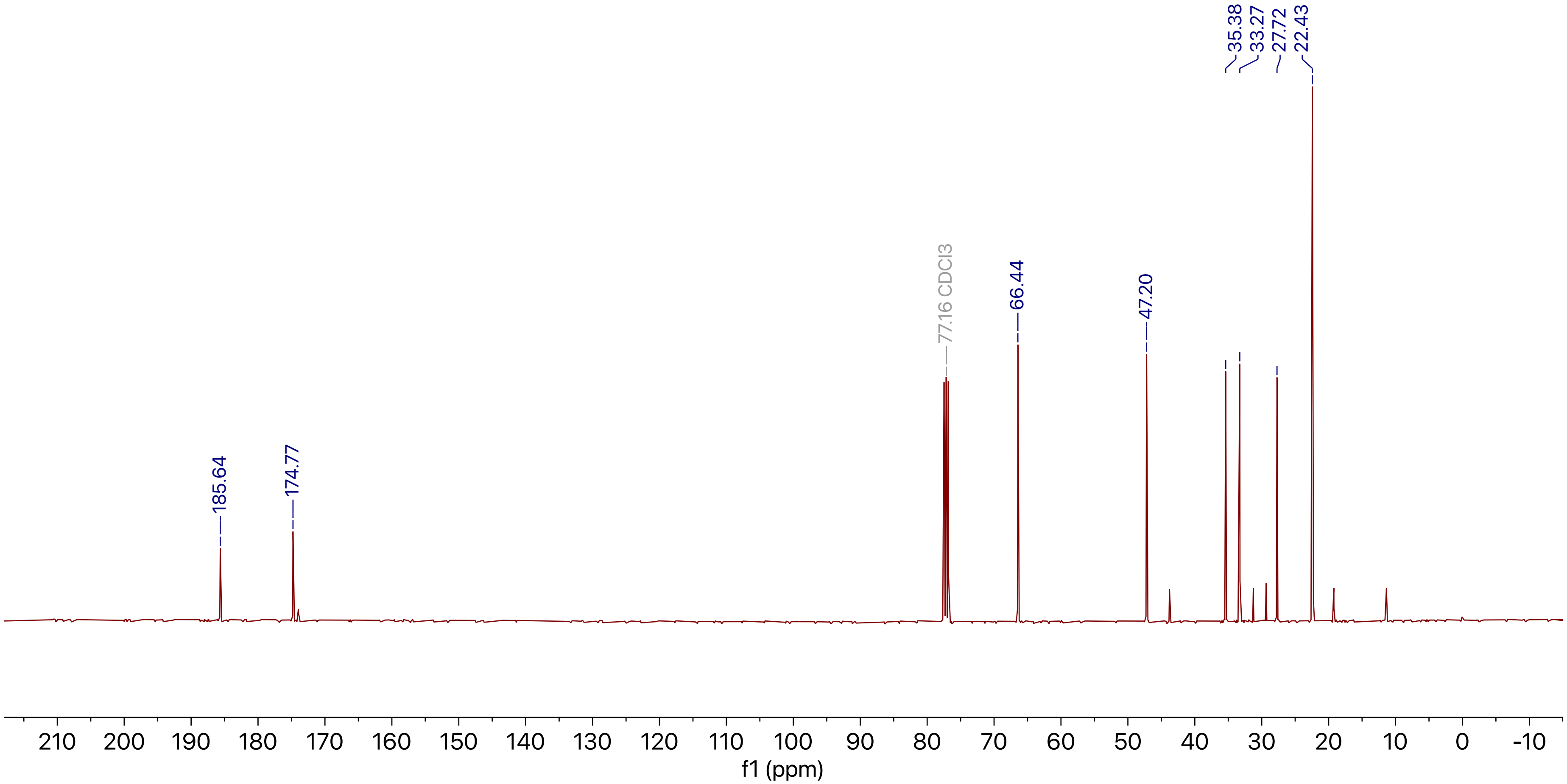

2D ^1H–^1H COSY (400 MHz, $CDCl_3$)

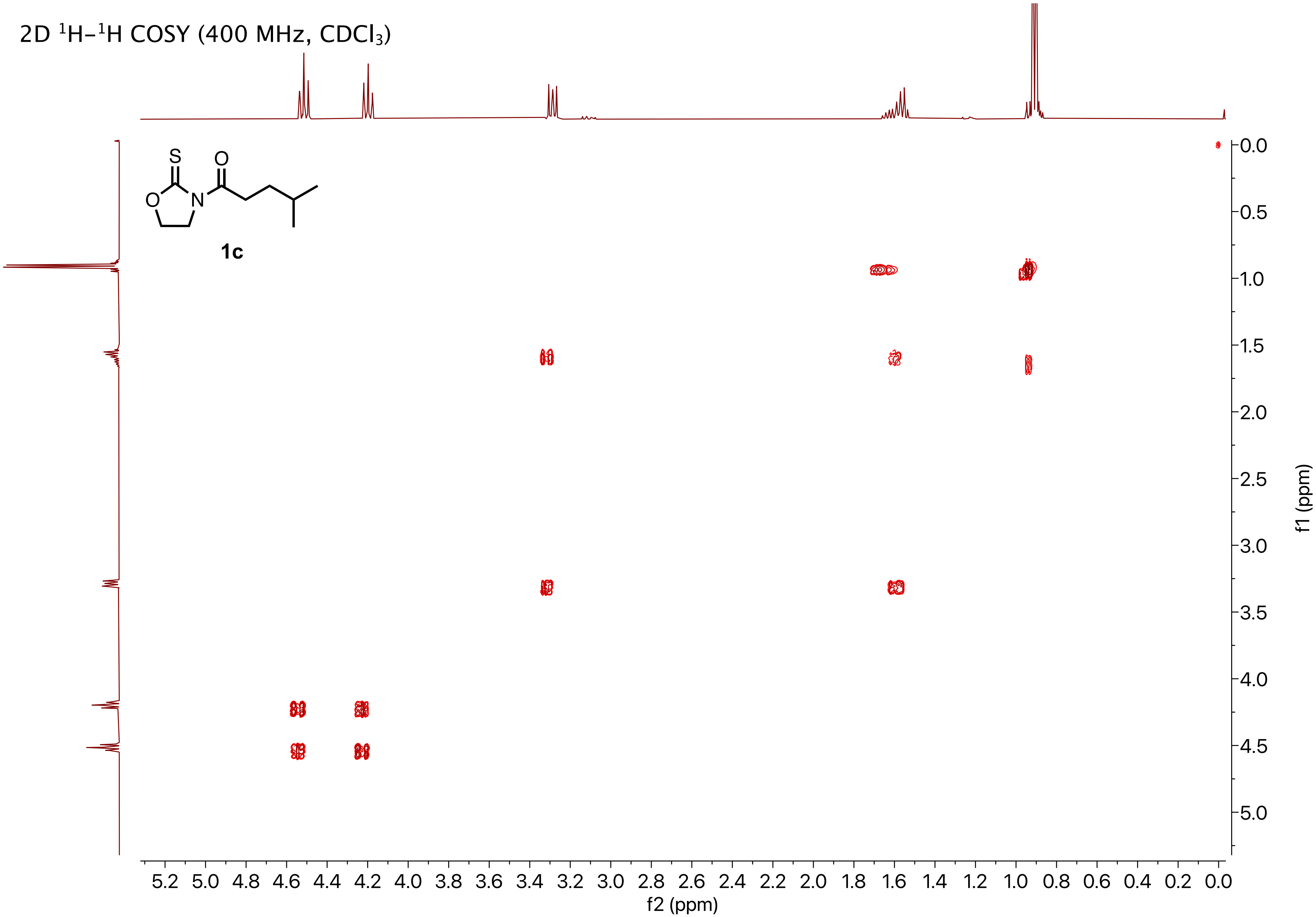

2D ^{1}H–^{13}C HSQC (400 MHz, $CDCl_3$)

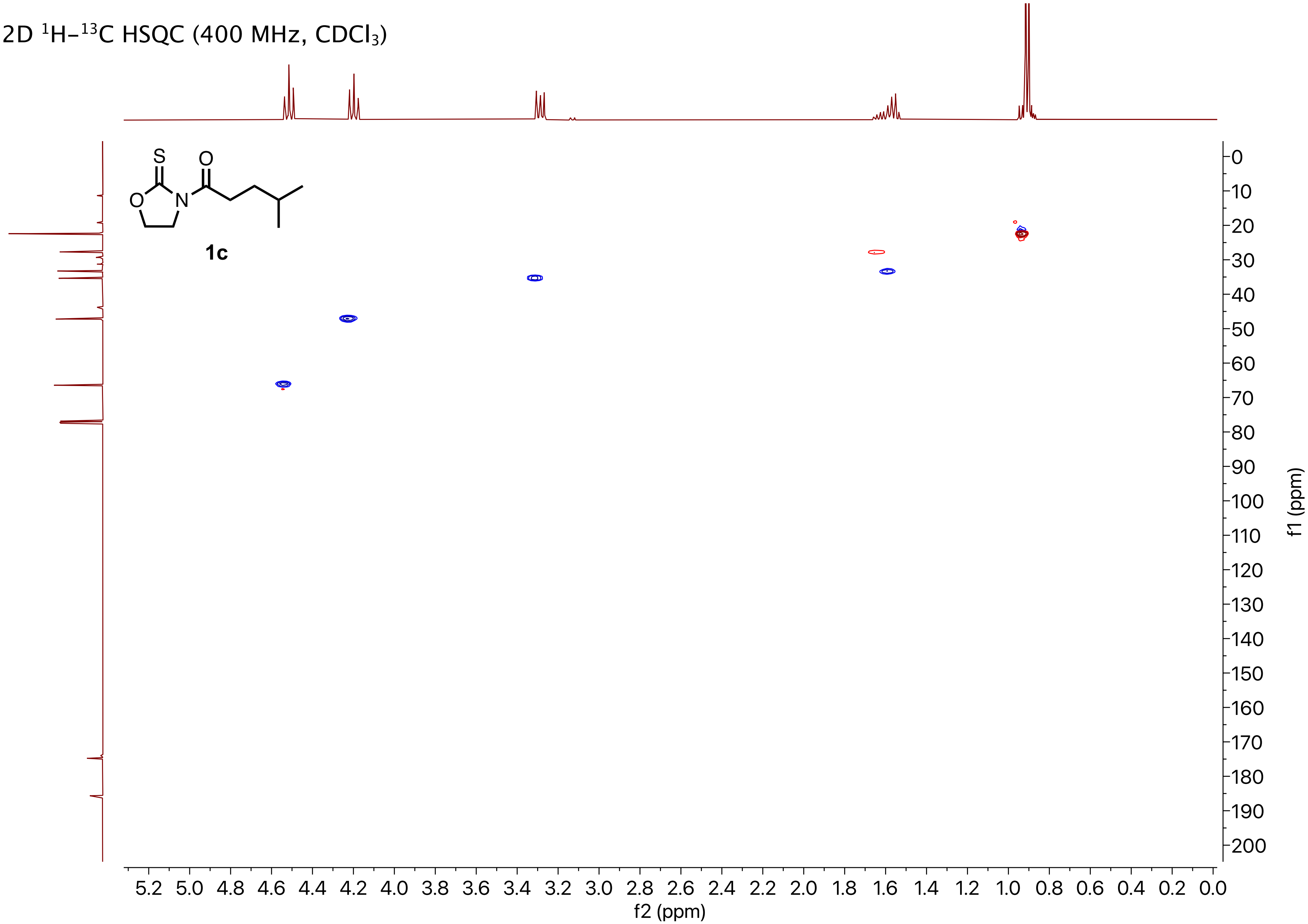

^{1}H NMR (400 MHz, $CDCl_3$)

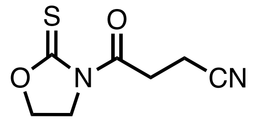

1d

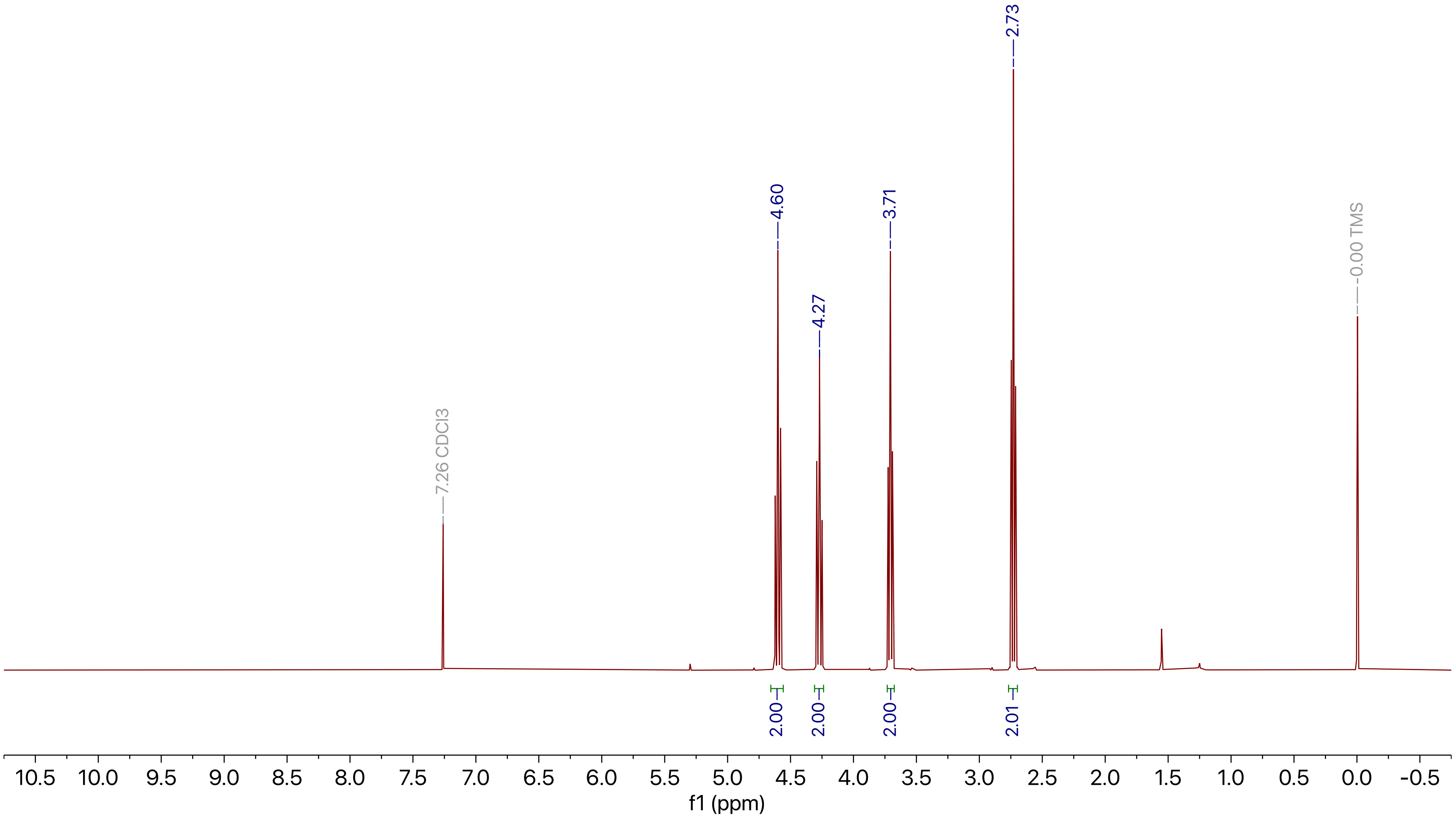

$^{13}C\{^{1}H\}$ NMR (101 MHz, $CDCl_3$)

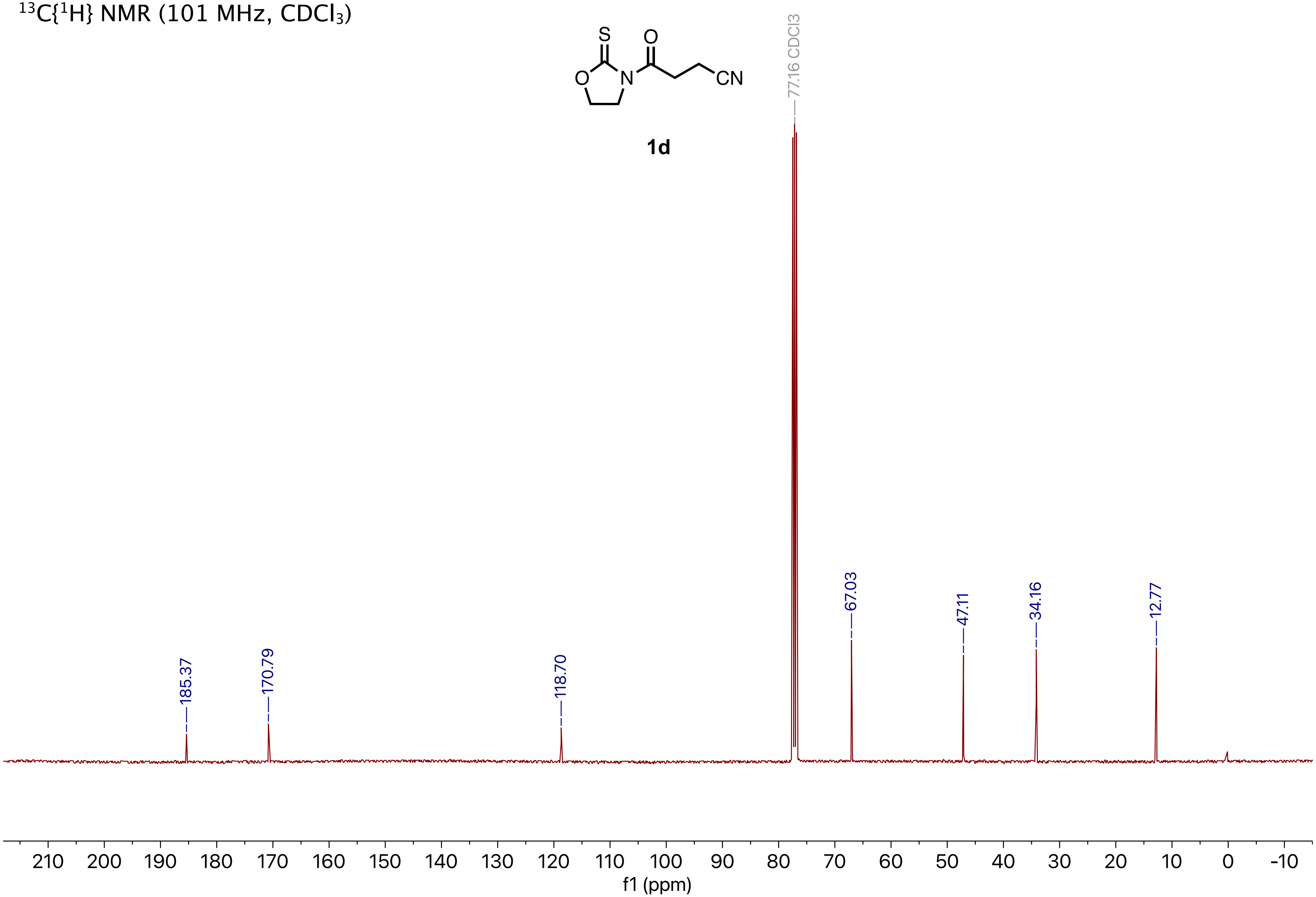

1d

2D ^{1}H–^{1}H COSY (400 MHz, $CDCl_3$)

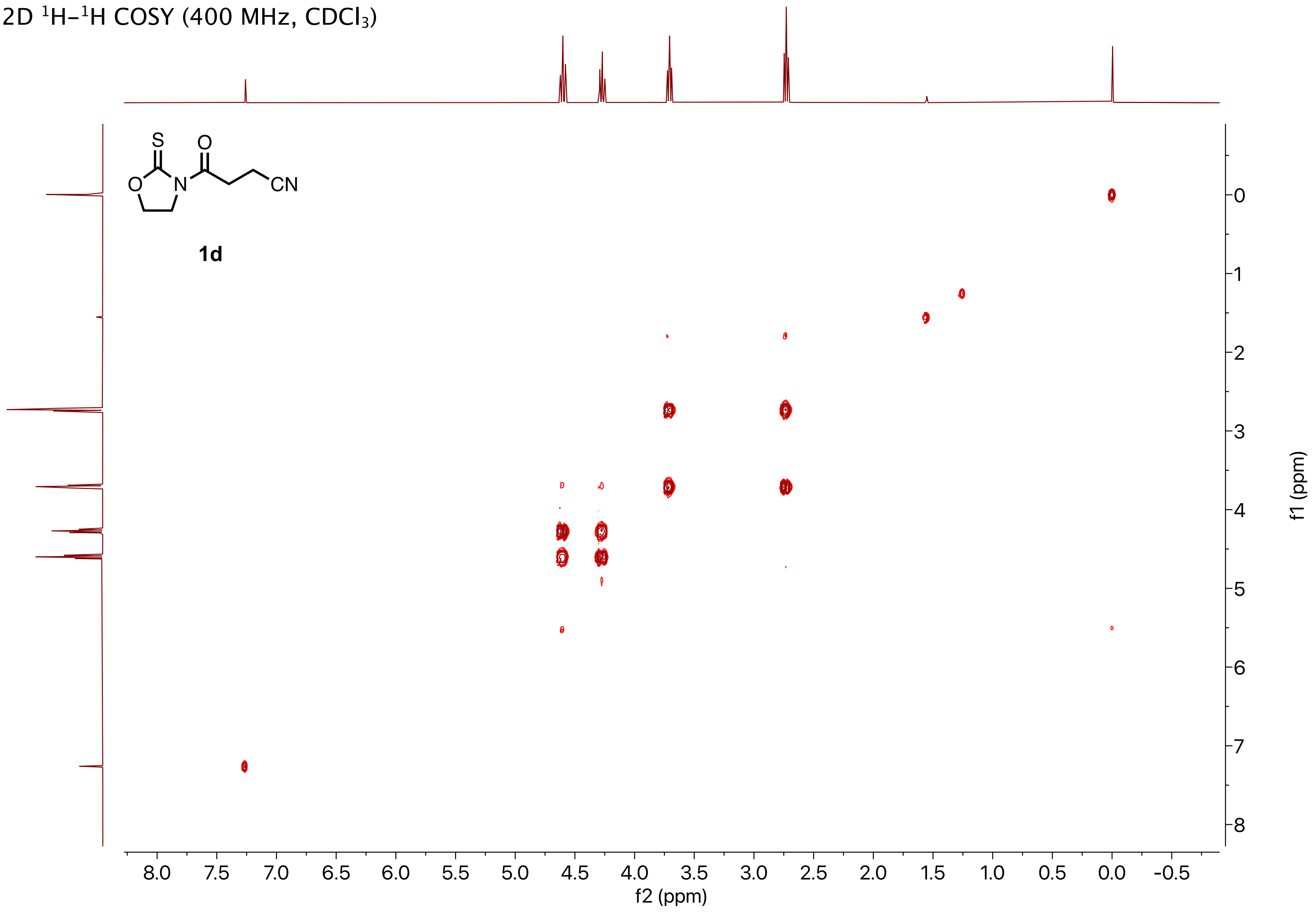

2D ^{1}H–^{13}C HSQC (400 MHz, $CDCl_3$)

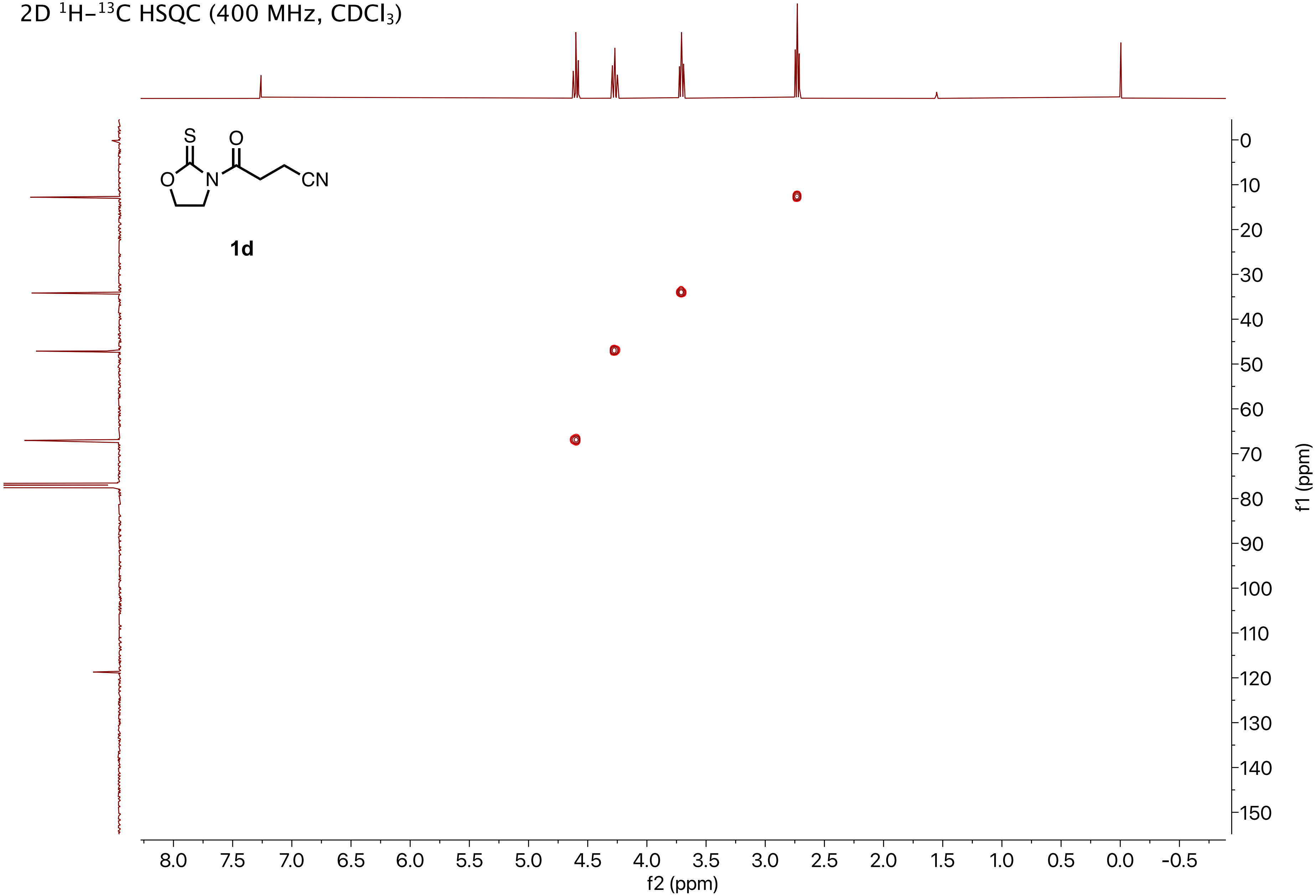

1d

^{1}H NMR (400 MHz, $CDCl_3$)

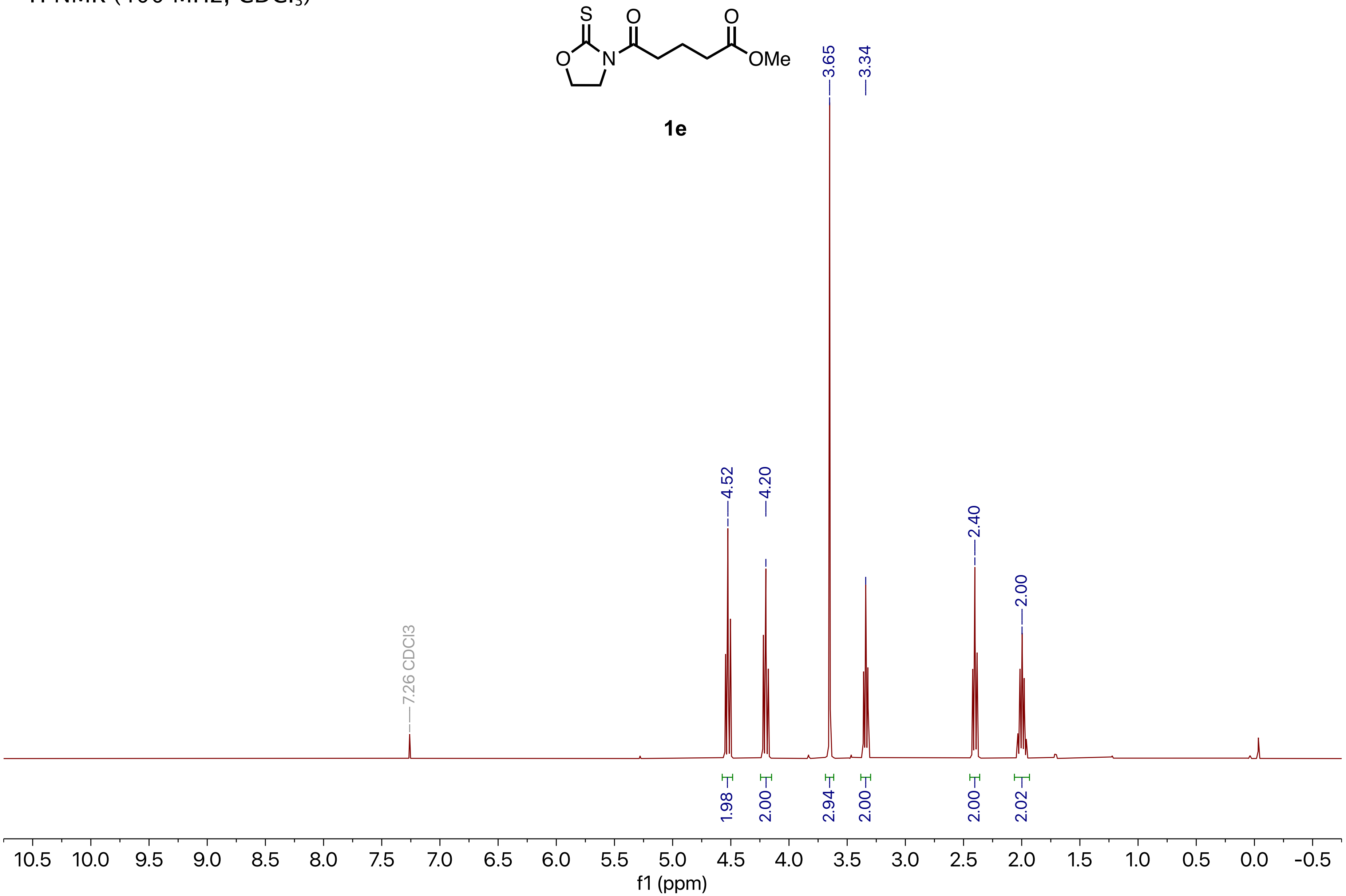

$^{13}C\{^{1}H\}$ NMR (101 MHz, $CDCl_3$)

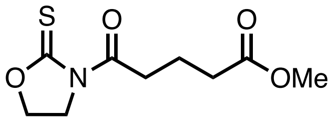

1e

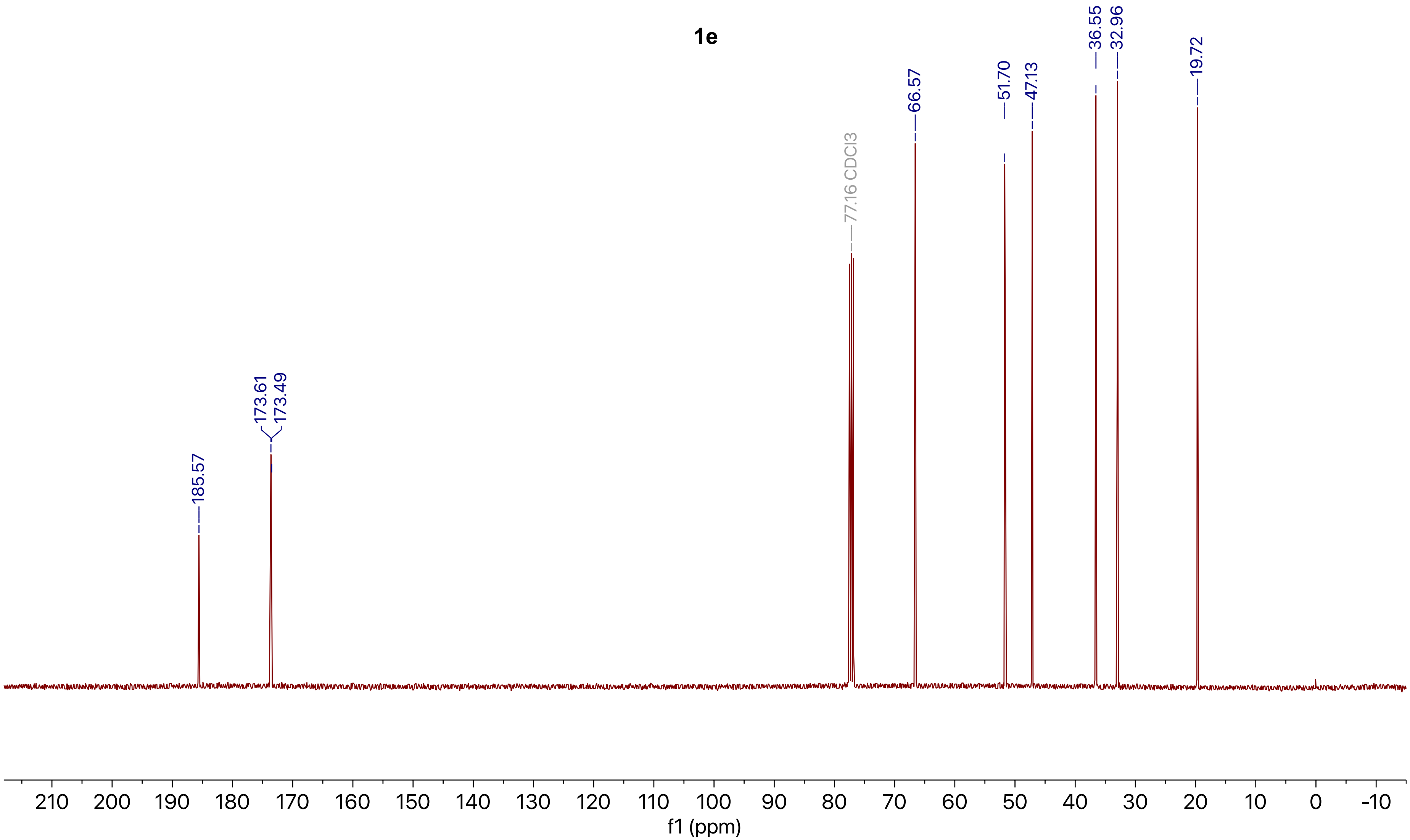

2D ^{1}H–^{1}H COSY (400 MHz, $CDCl_3$)

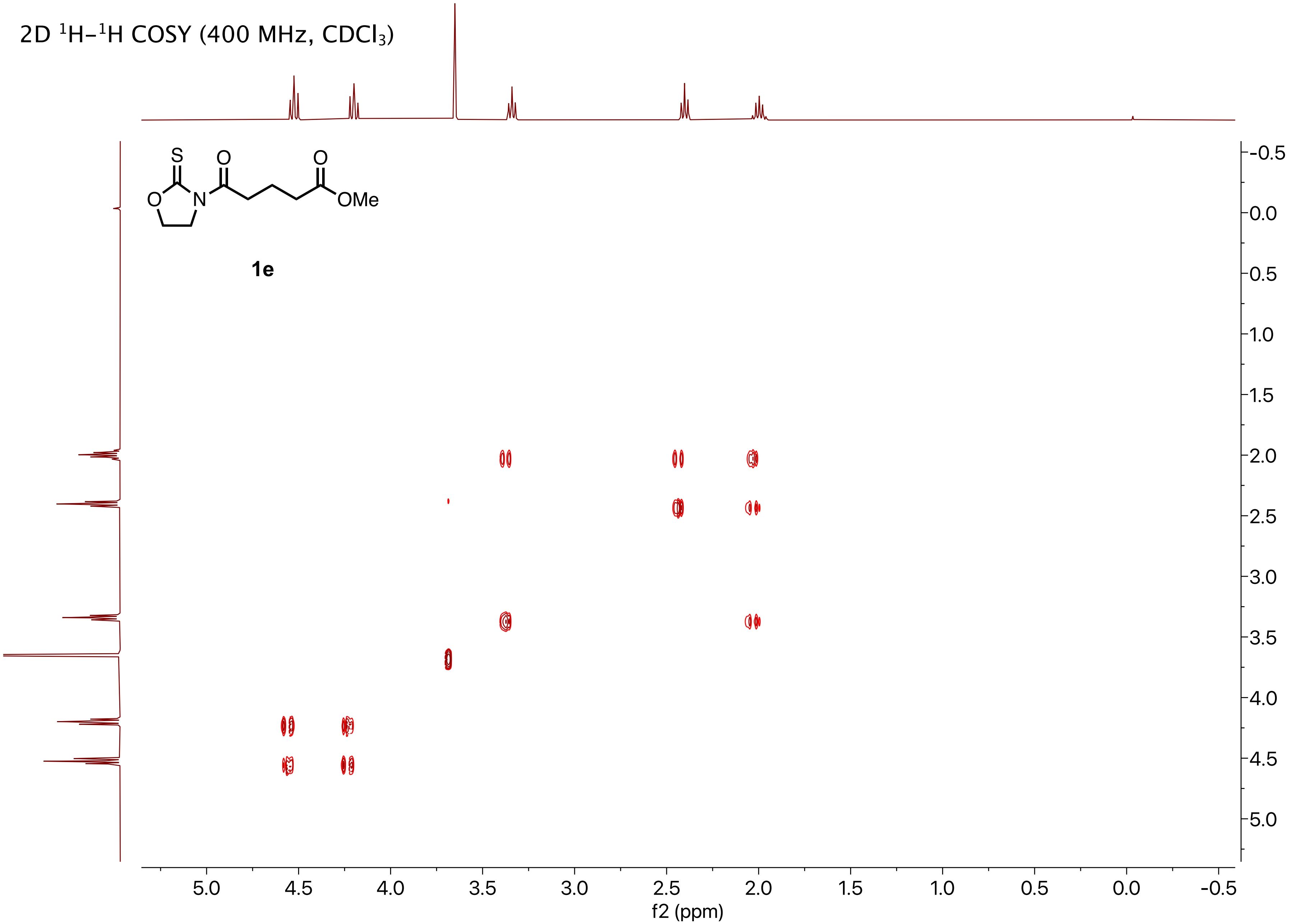

2D ^{1}H–^{13}C HSQC (400 MHz, $CDCl_3$)

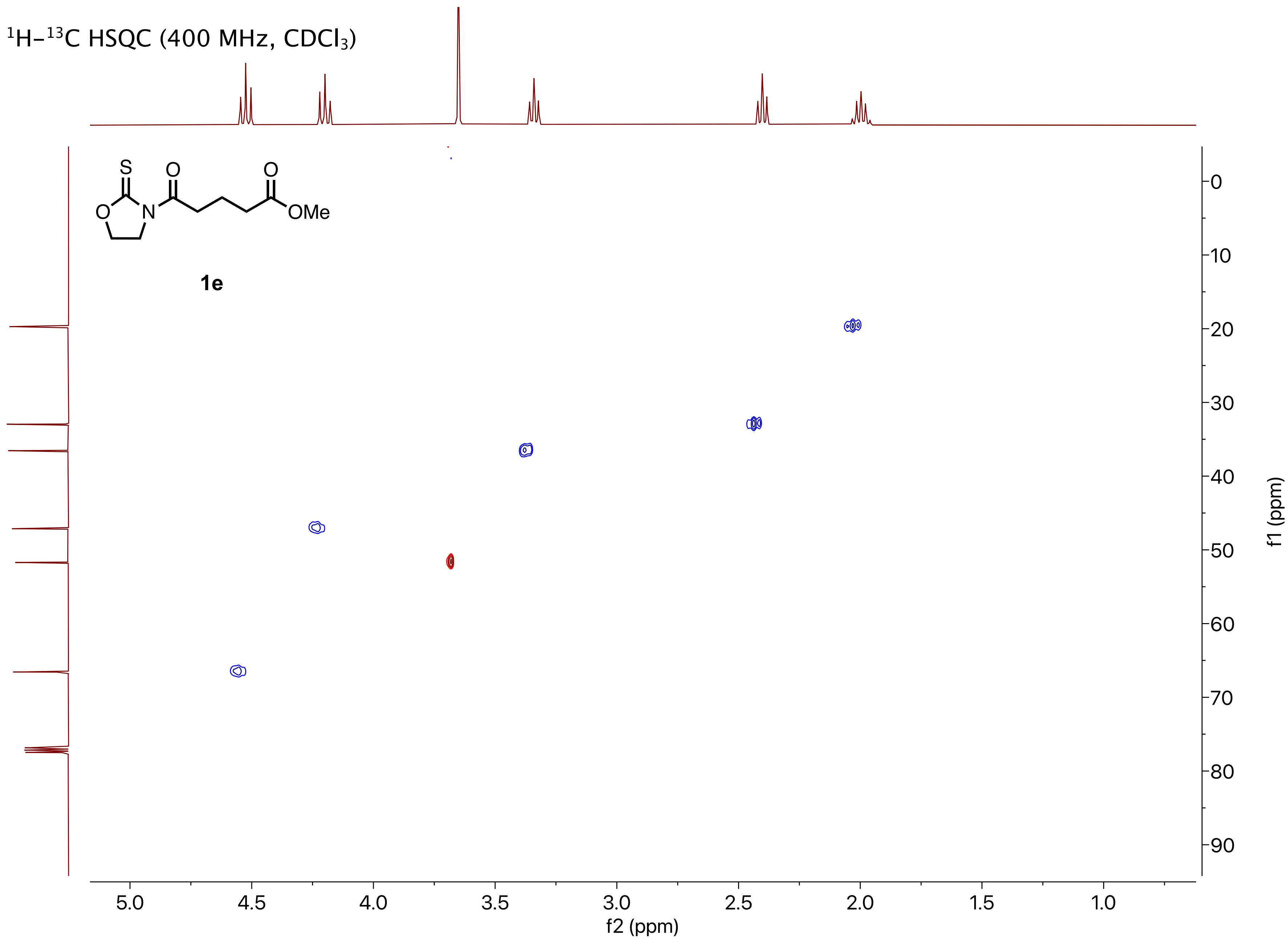

^{1}H NMR (400 MHz, $CDCl_3$)

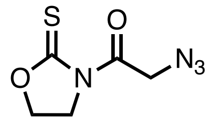

1f

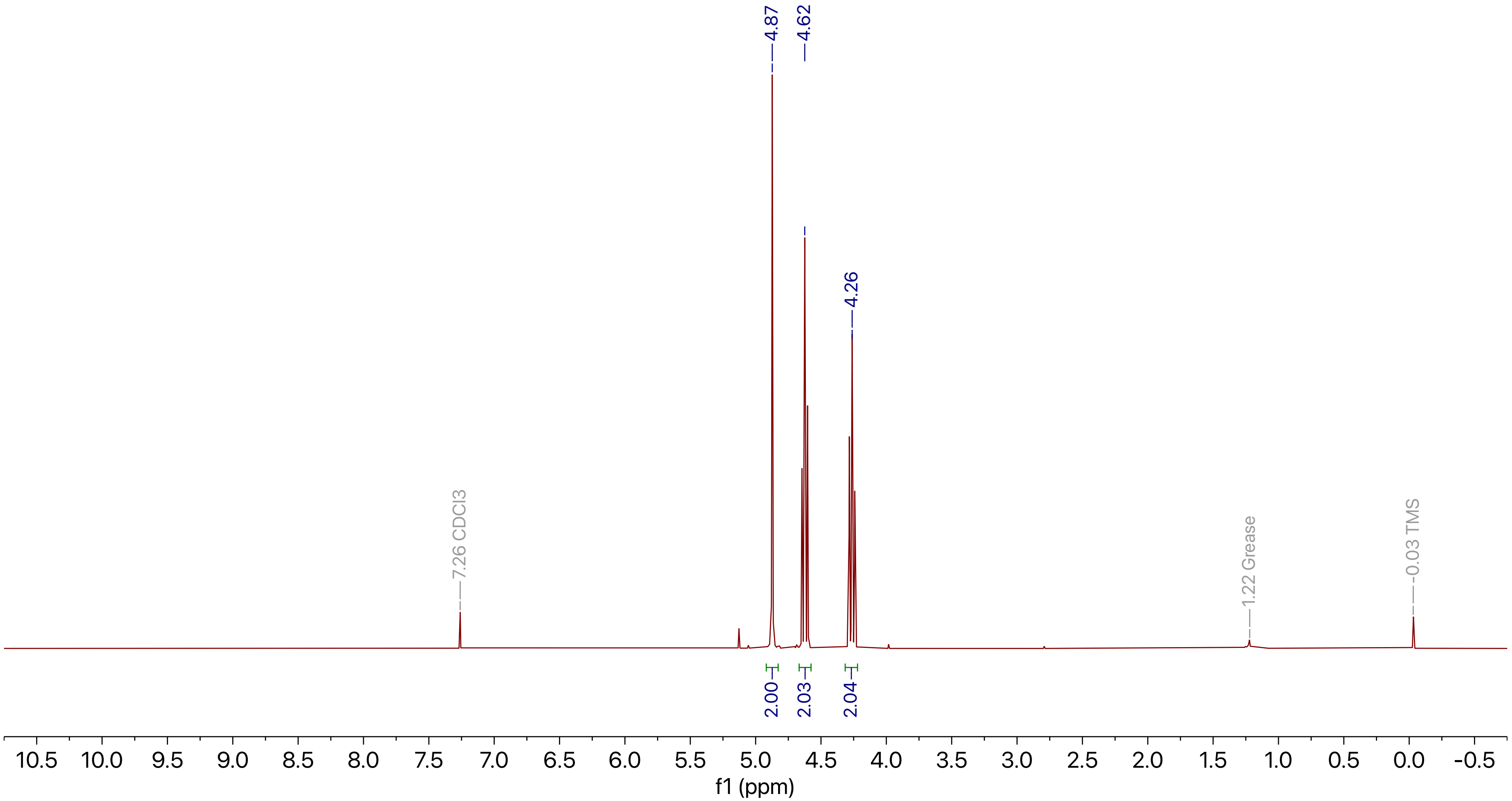

$^{13}C\{^{1}H\}$ NMR (101 MHz, $CDCl_3$)

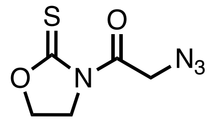

1f

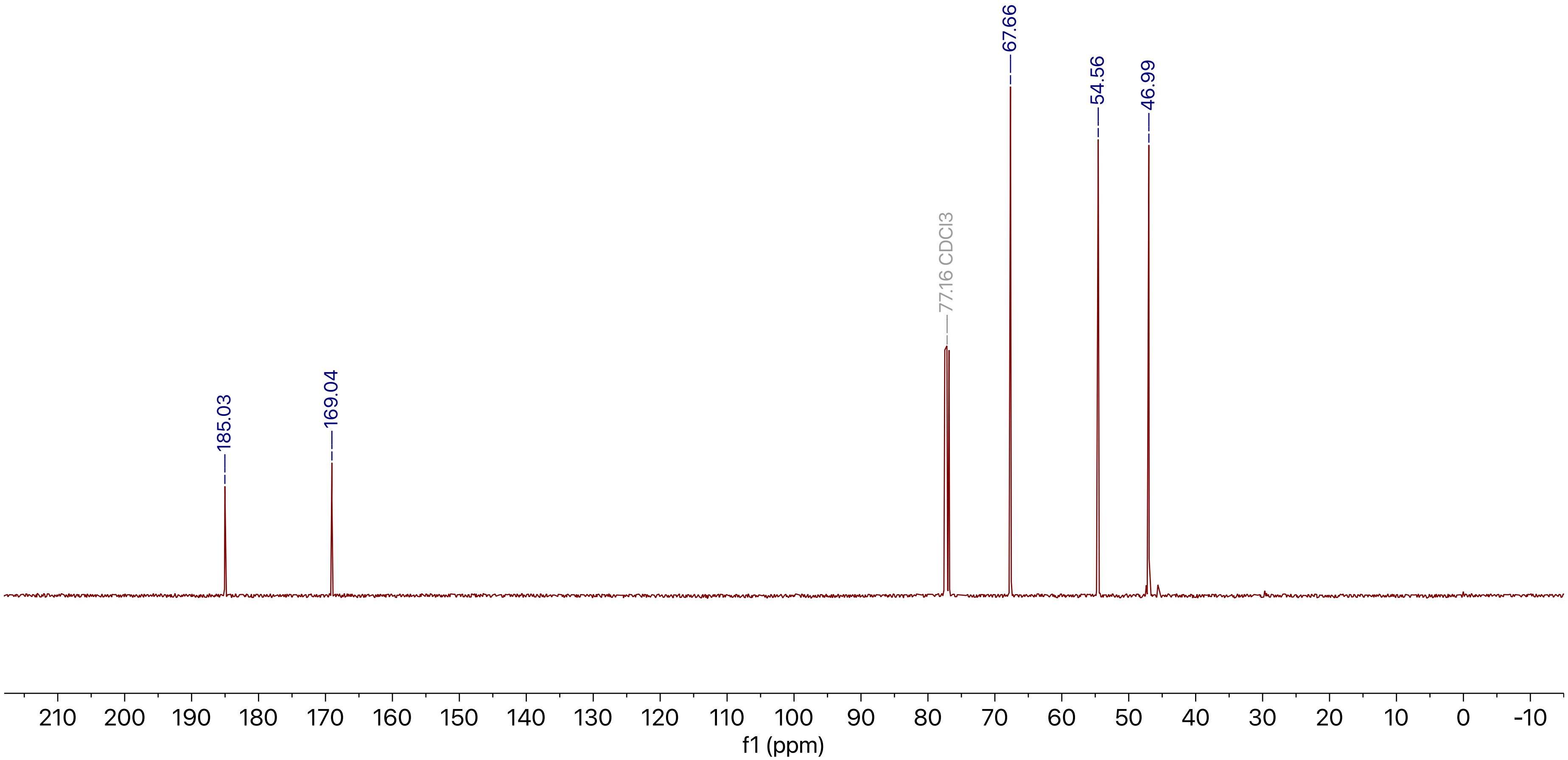

2D ^{1}H–^{1}H COSY (400 MHz, $CDCl_3$)

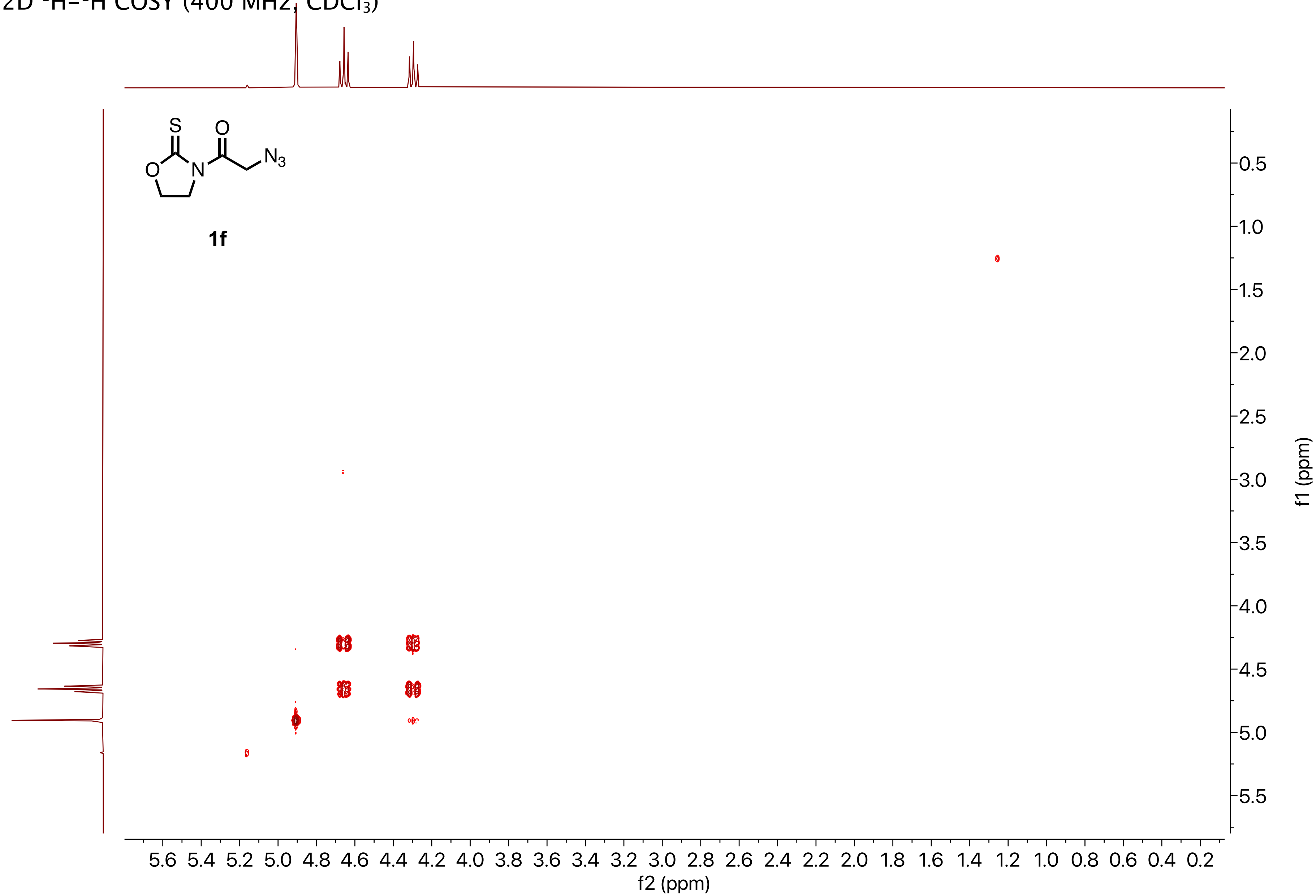

2D ^{1}H–^{13}C HSQC (400 MHz, $CDCl_3$)

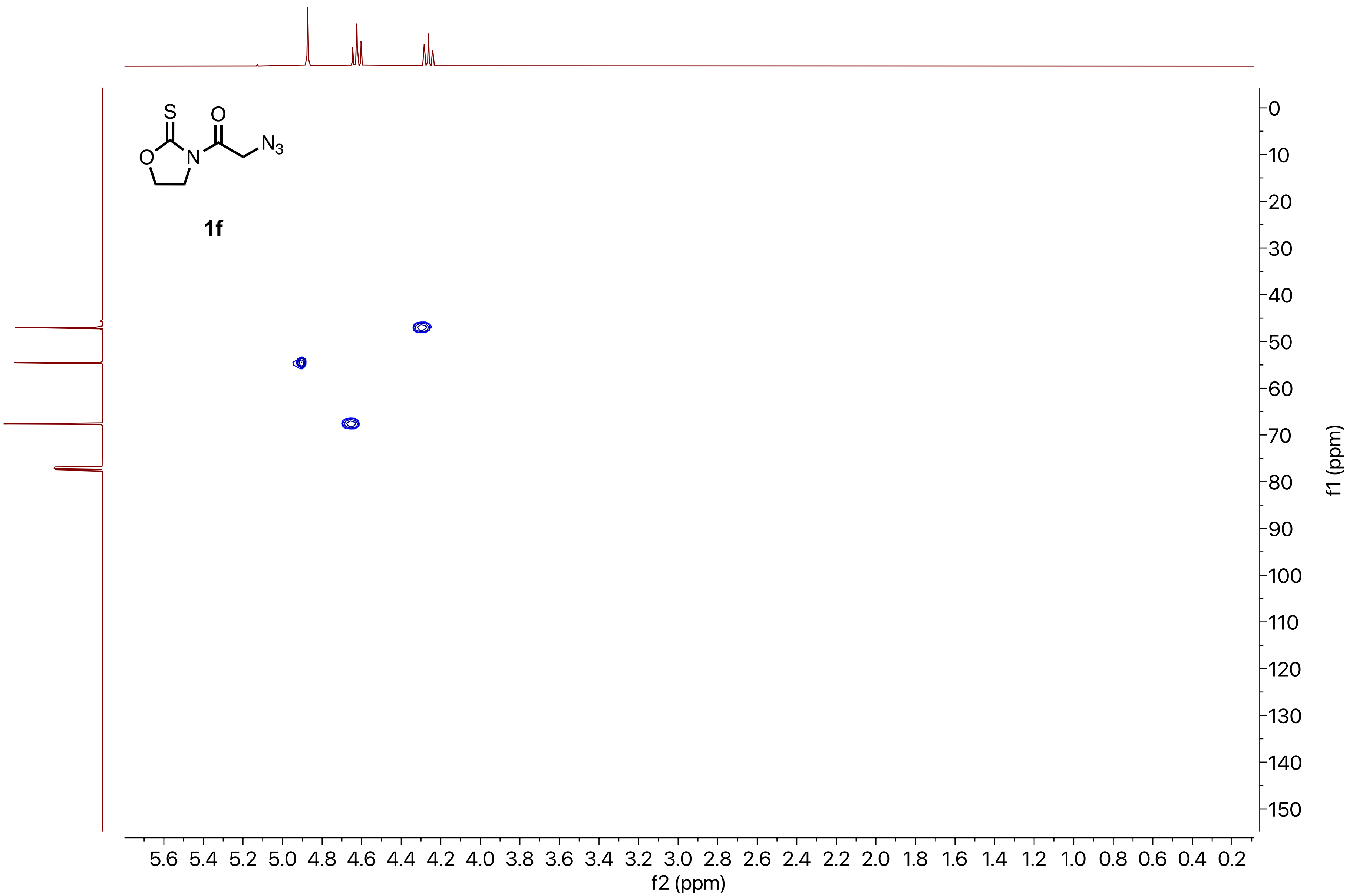

^{1}H NMR (400 MHz, $CDCl_3$)

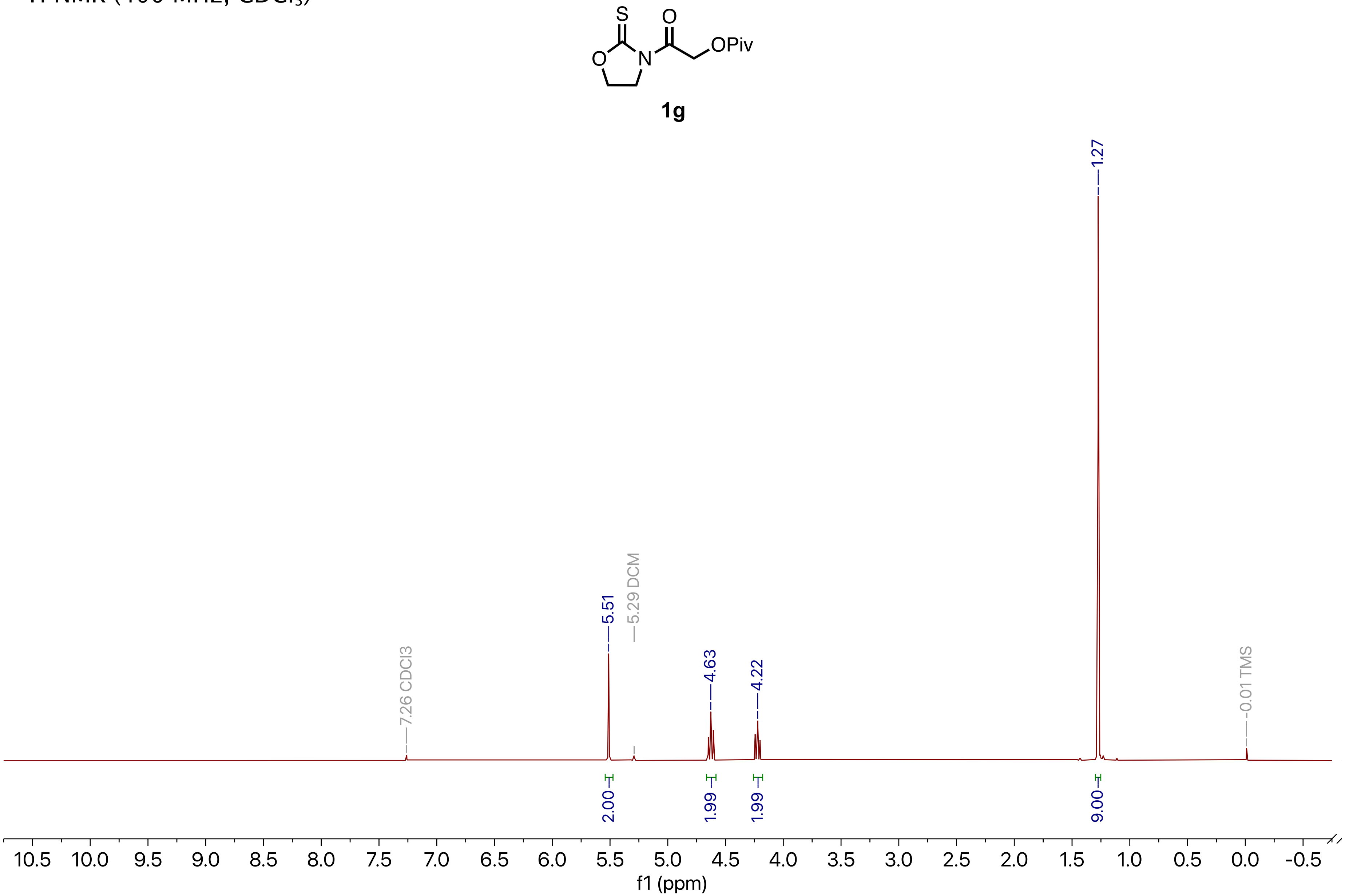

$^{13}C\{^{1}H\}$ NMR (101 MHz, $CDCl_3$)

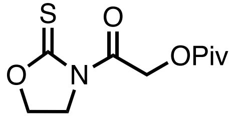

1g

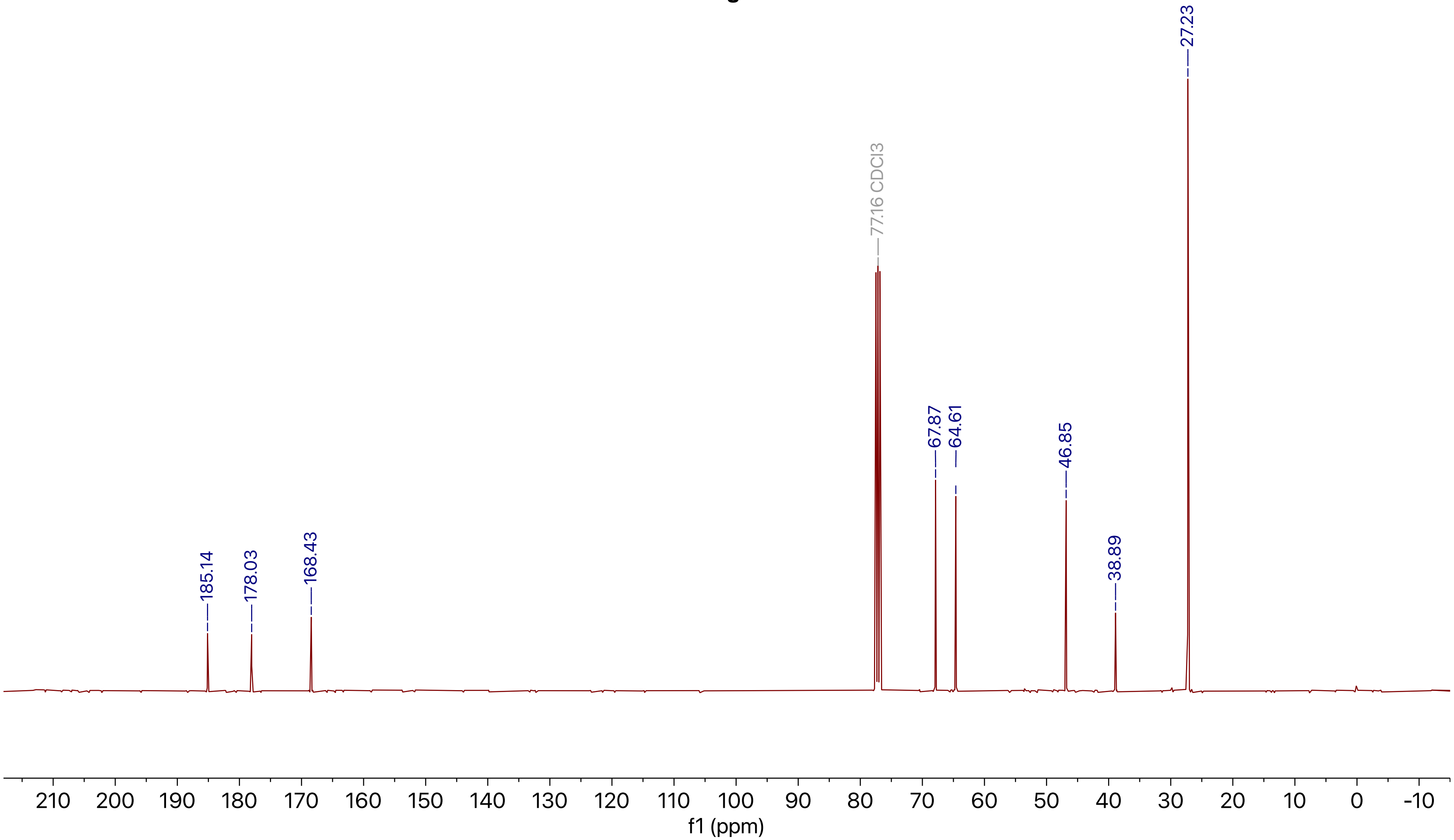

2D ^{1}H–^{1}H COSY (400 MHz, $CDCl_3$)

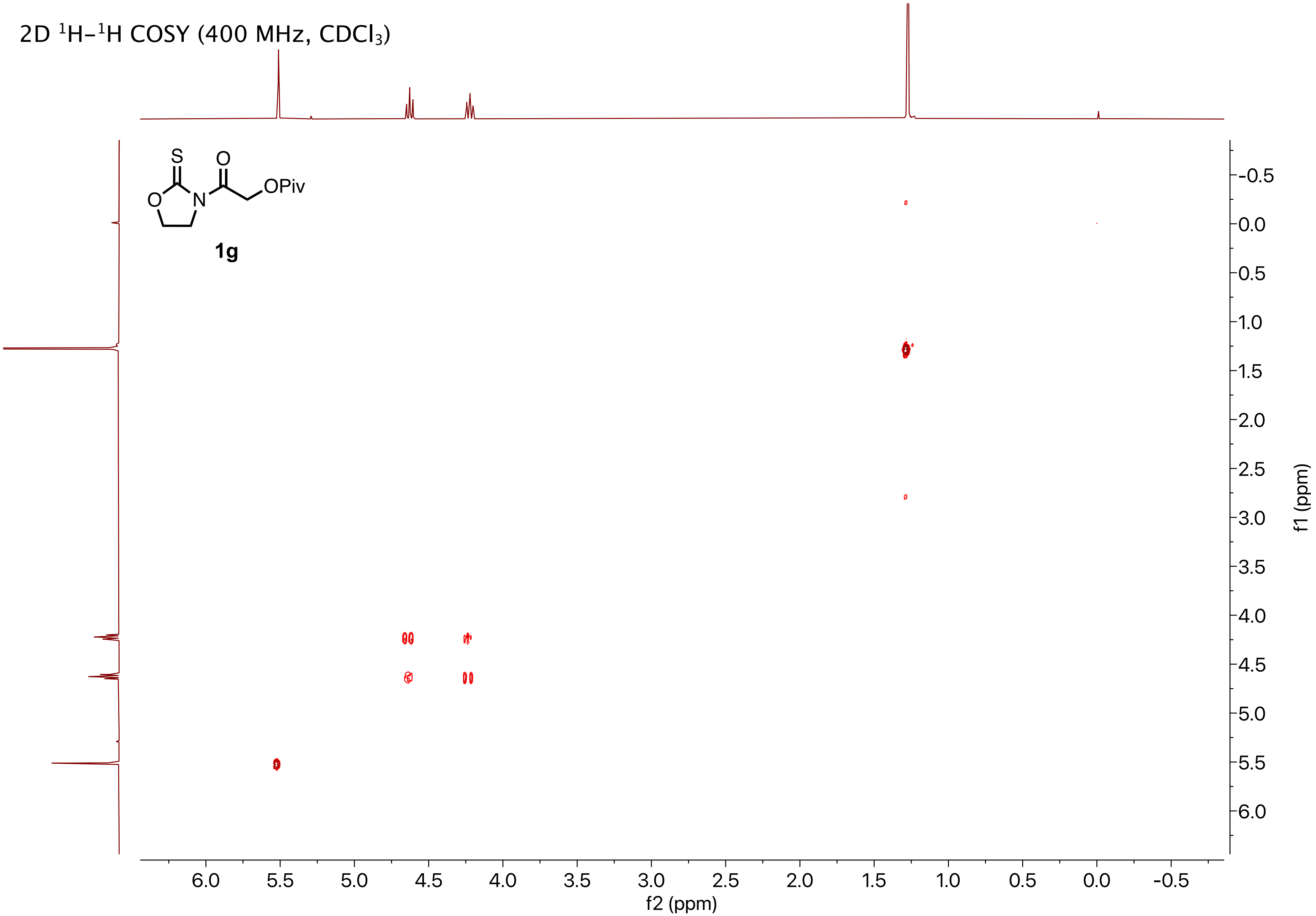

2D ^{1}H–^{13}C HSQC (400 MHz, $CDCl_3$)

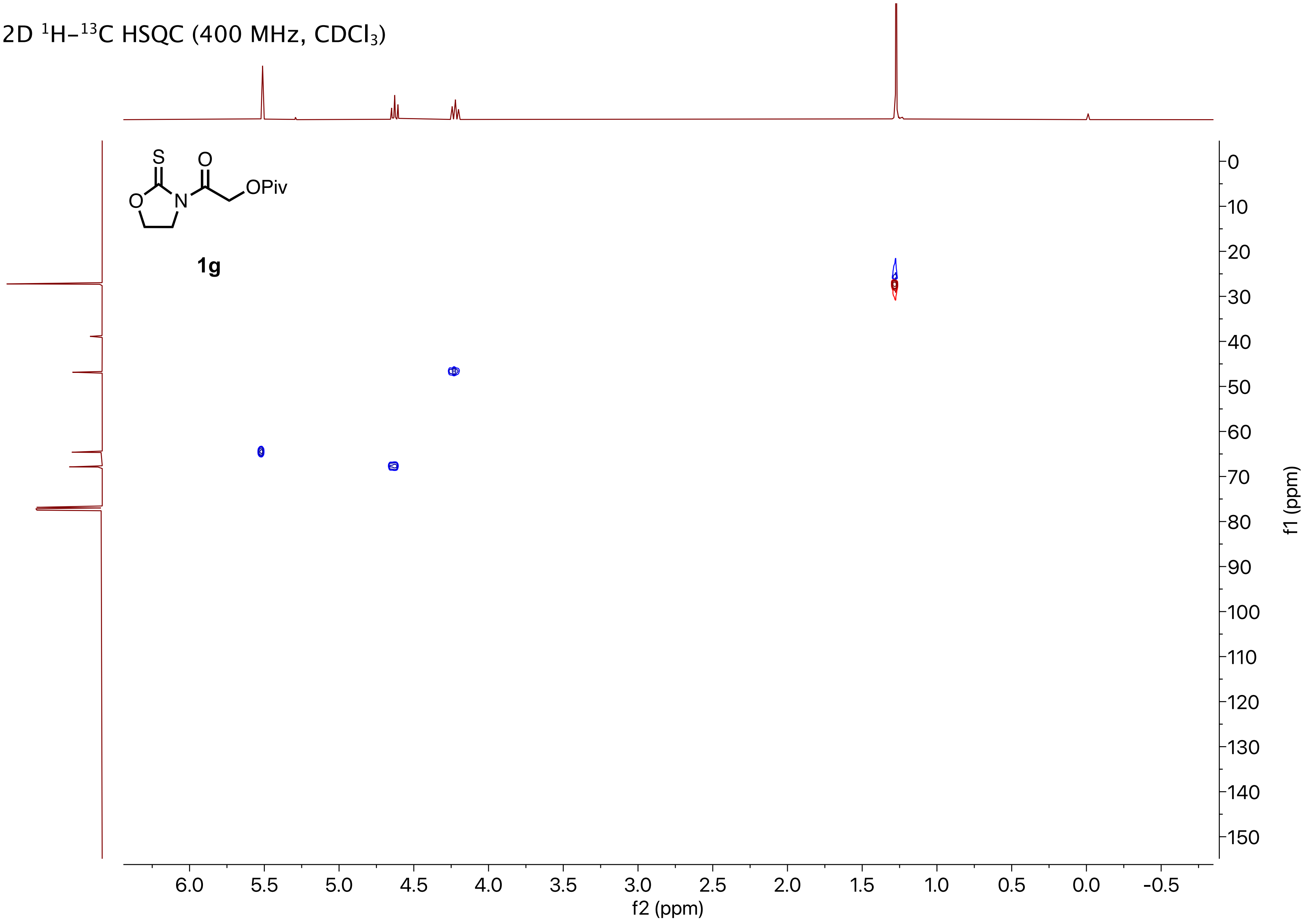

^{1}H NMR (400 MHz, $CDCl_3$)

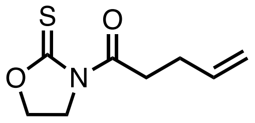

1h

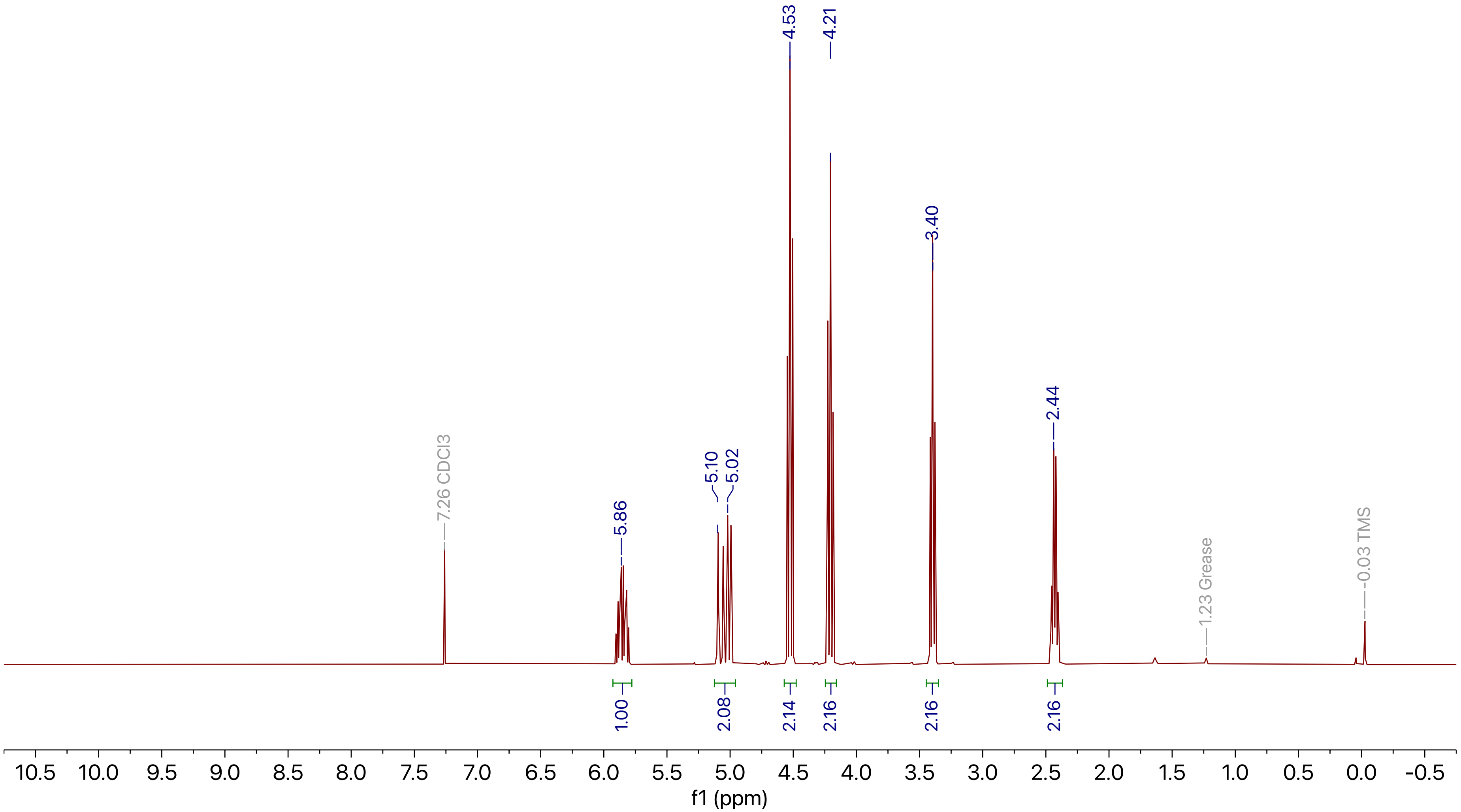

$^{13}C\{^{1}H\}$ NMR (101 MHz, $CDCl_3$)

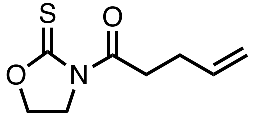

1h

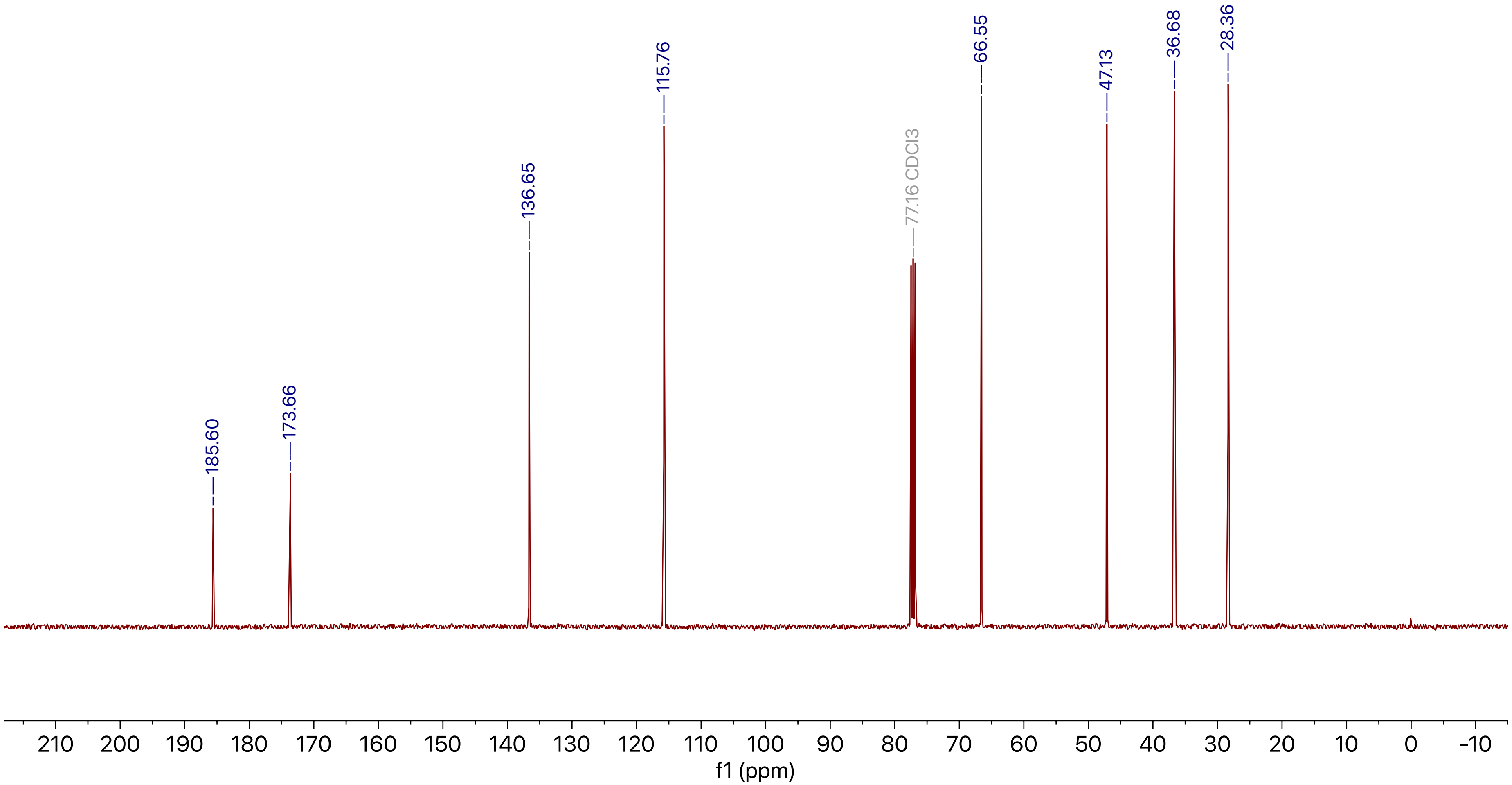

2D ^{1}H–^{1}H COSY (400 MHz, $CDCl_3$)

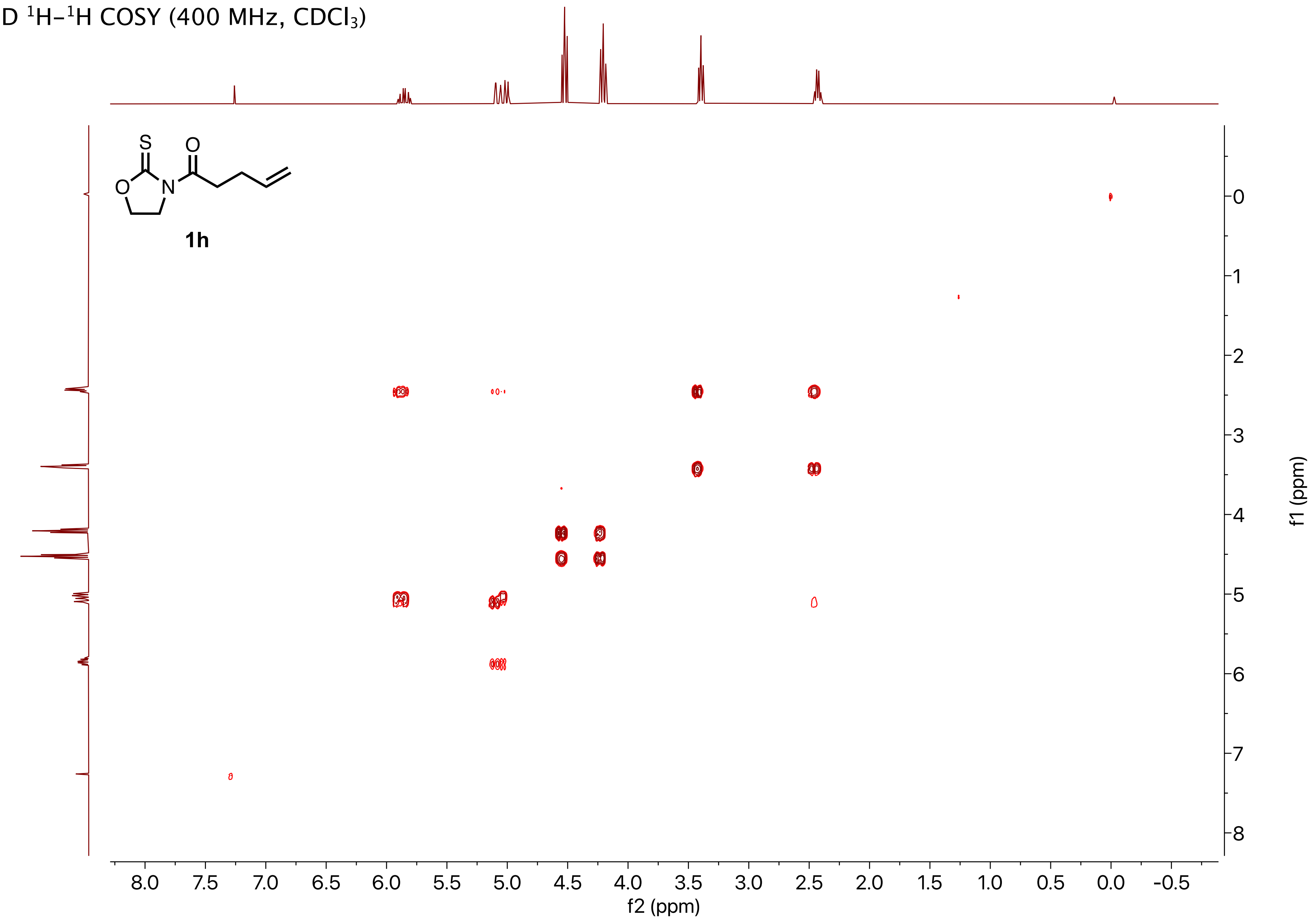

2D $^{1}H-^{13}C$ HSQC (400 MHz, $CDCl_3$)

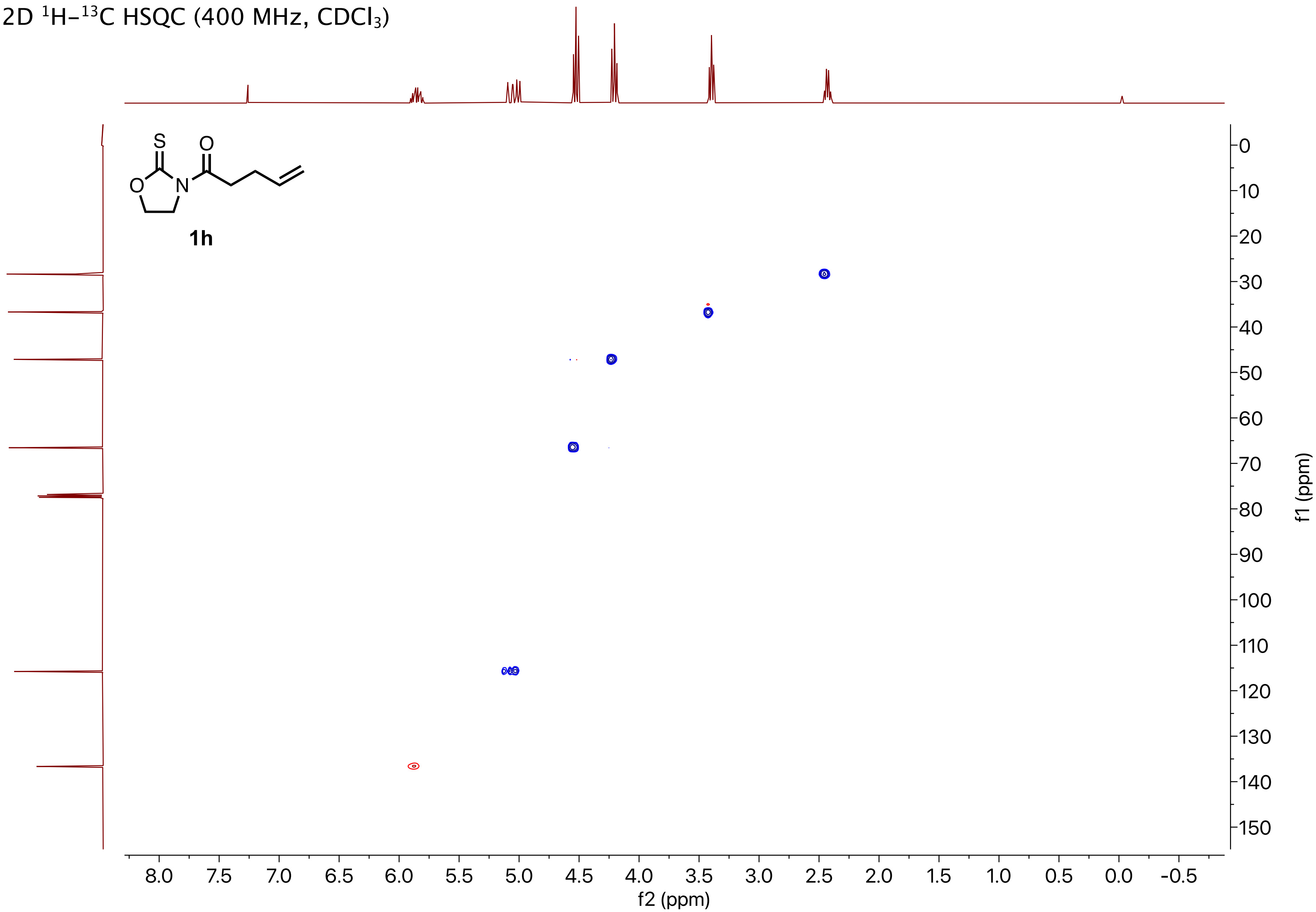

^{1}H NMR (400 MHz, $CDCl_3$)

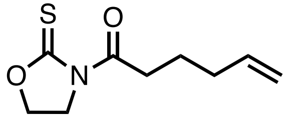

1i

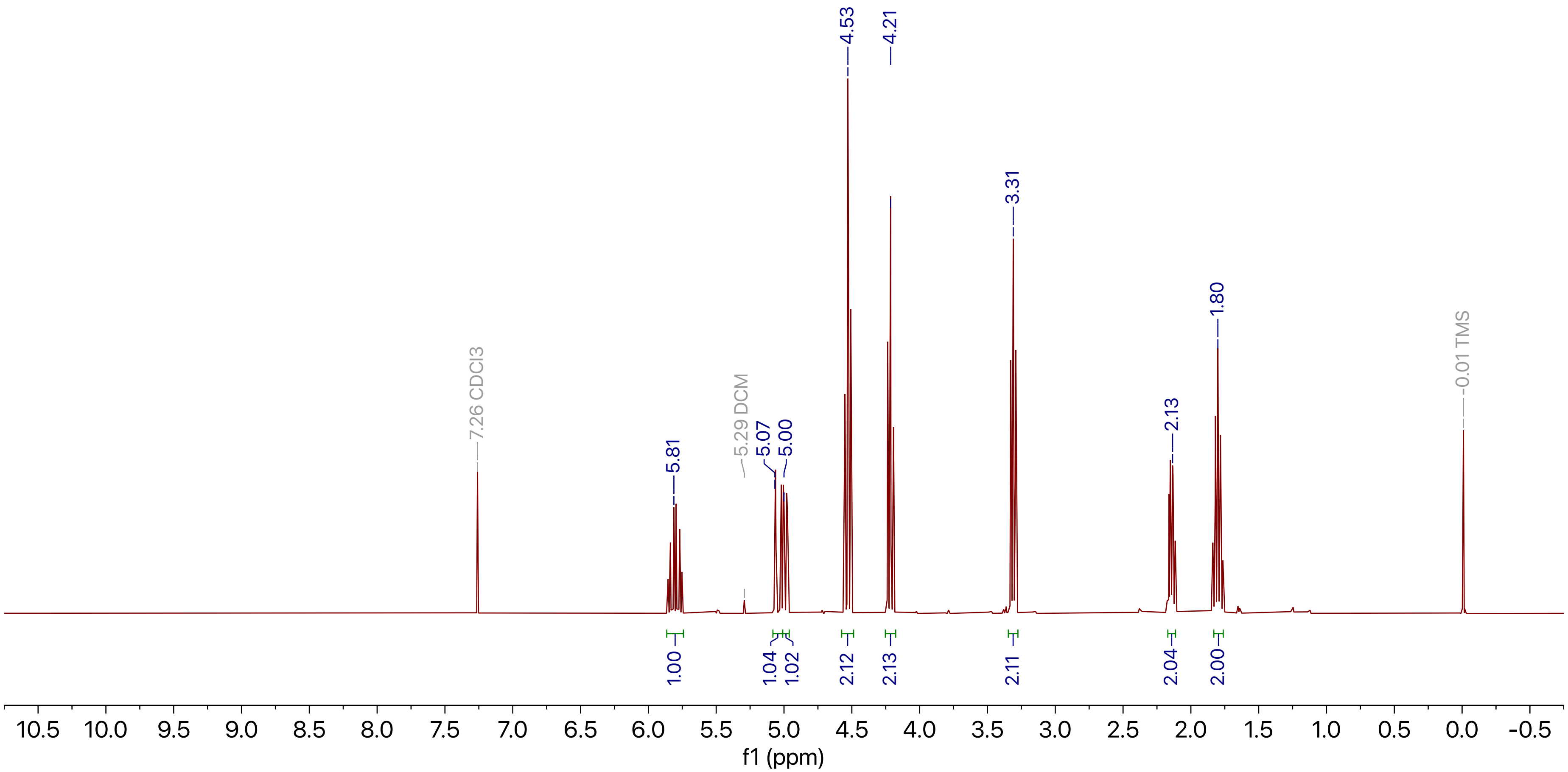

$^{13}C\{^{1}H\}$ NMR (101 MHz, $CDCl_3$)

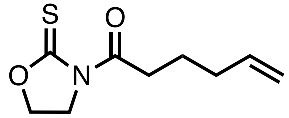

1i

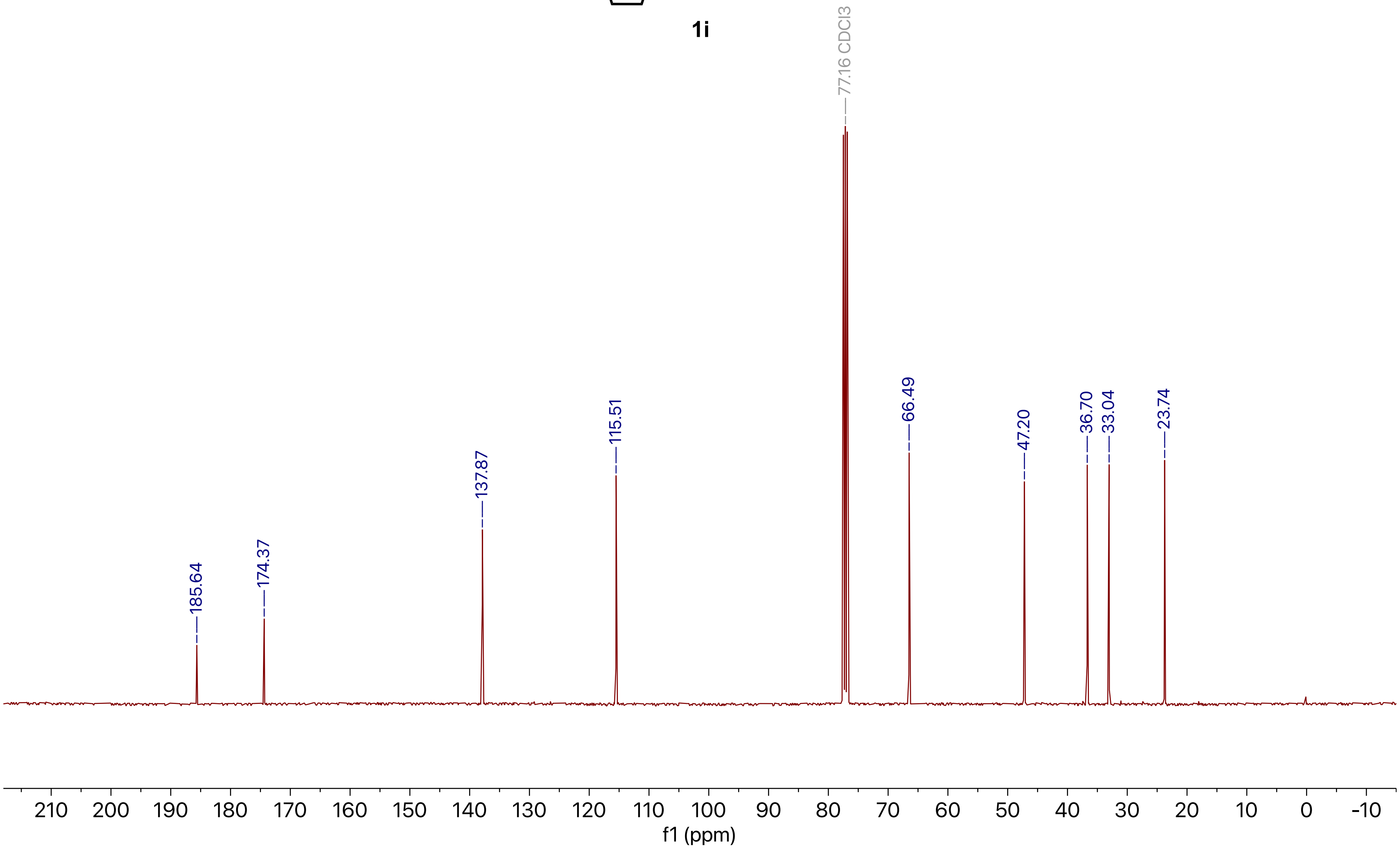

2D ^{1}H–^{1}H COSY (400 MHz, $CDCl_3$)

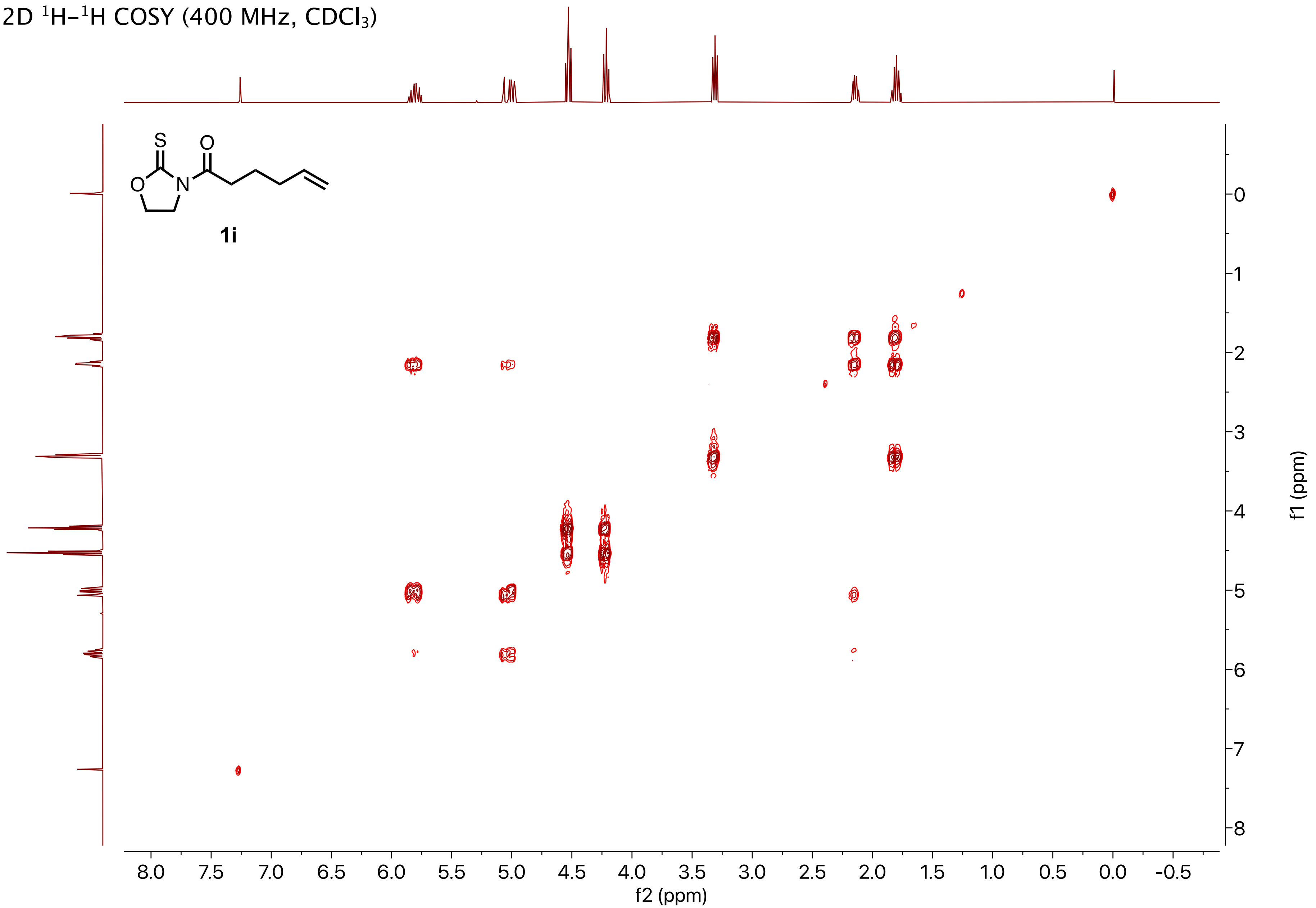

2D ^{1}H–^{13}C HSQC (400 MHz, $CDCl_3$)

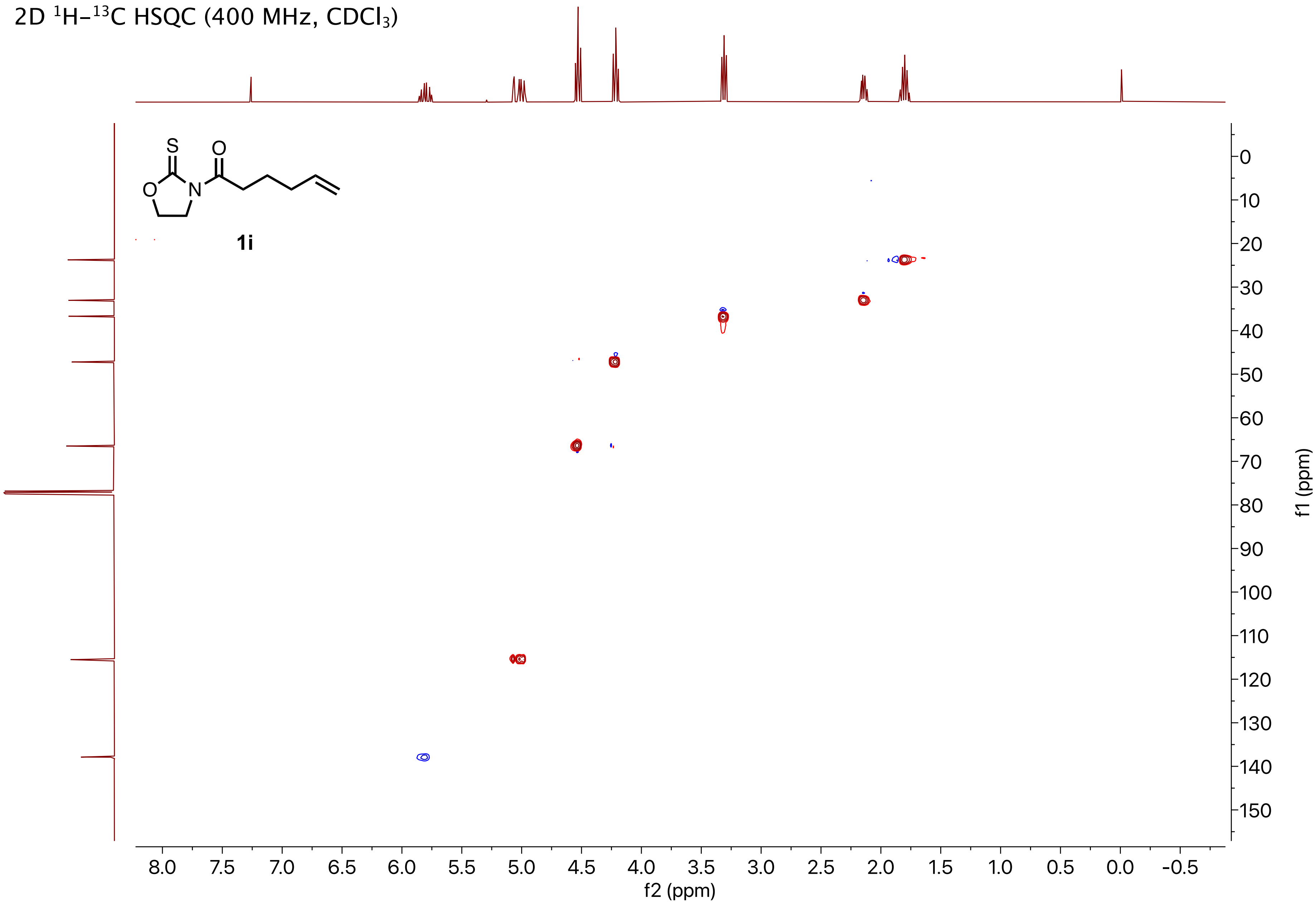

1i

^{1}H NMR (400 MHz, $CDCl_3$)

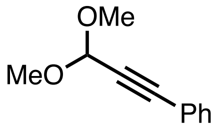

2B

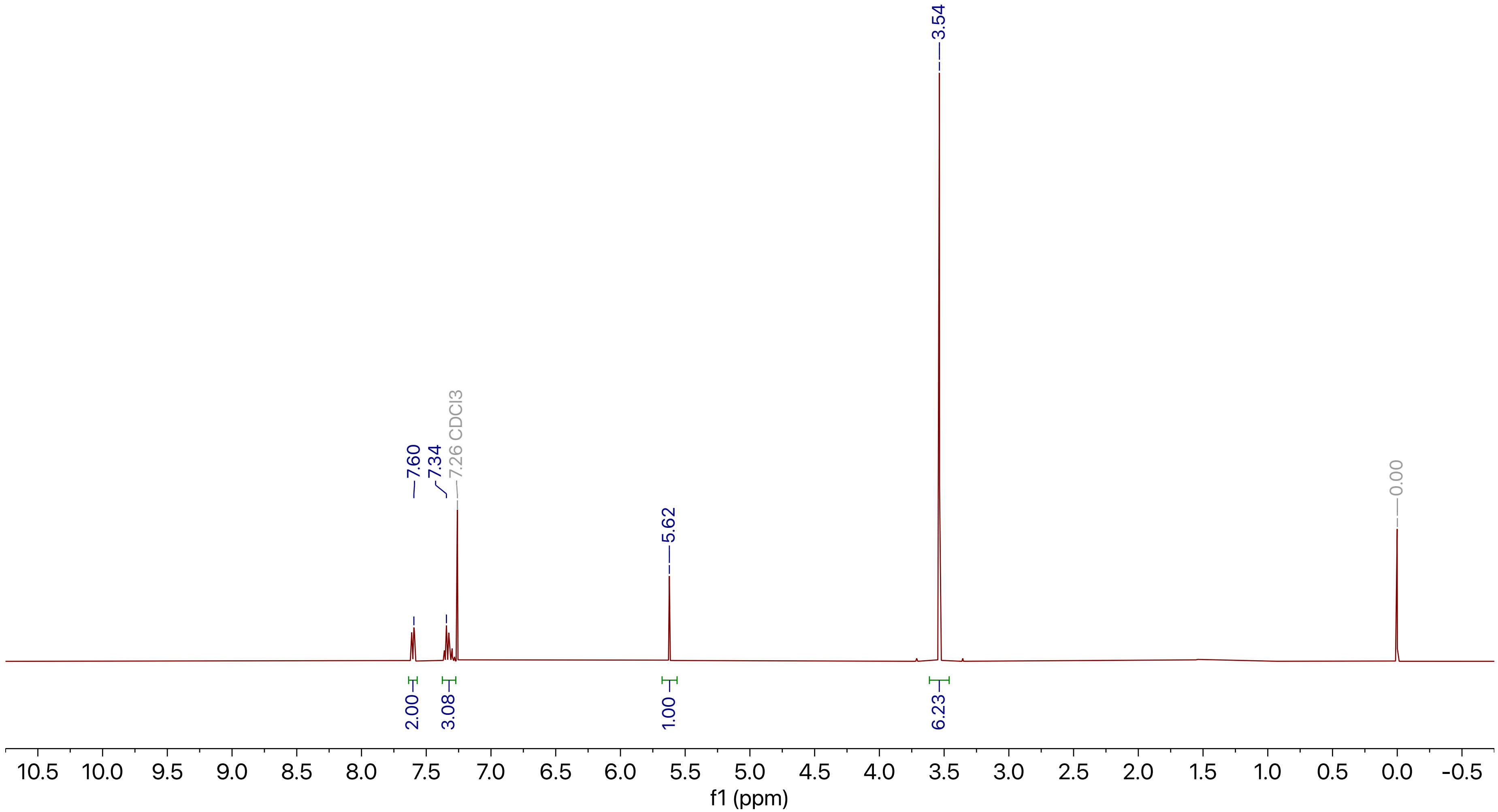

^{1}H NMR (400 MHz, $CDCl_3$)

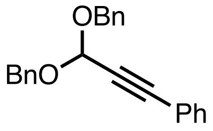

XT379

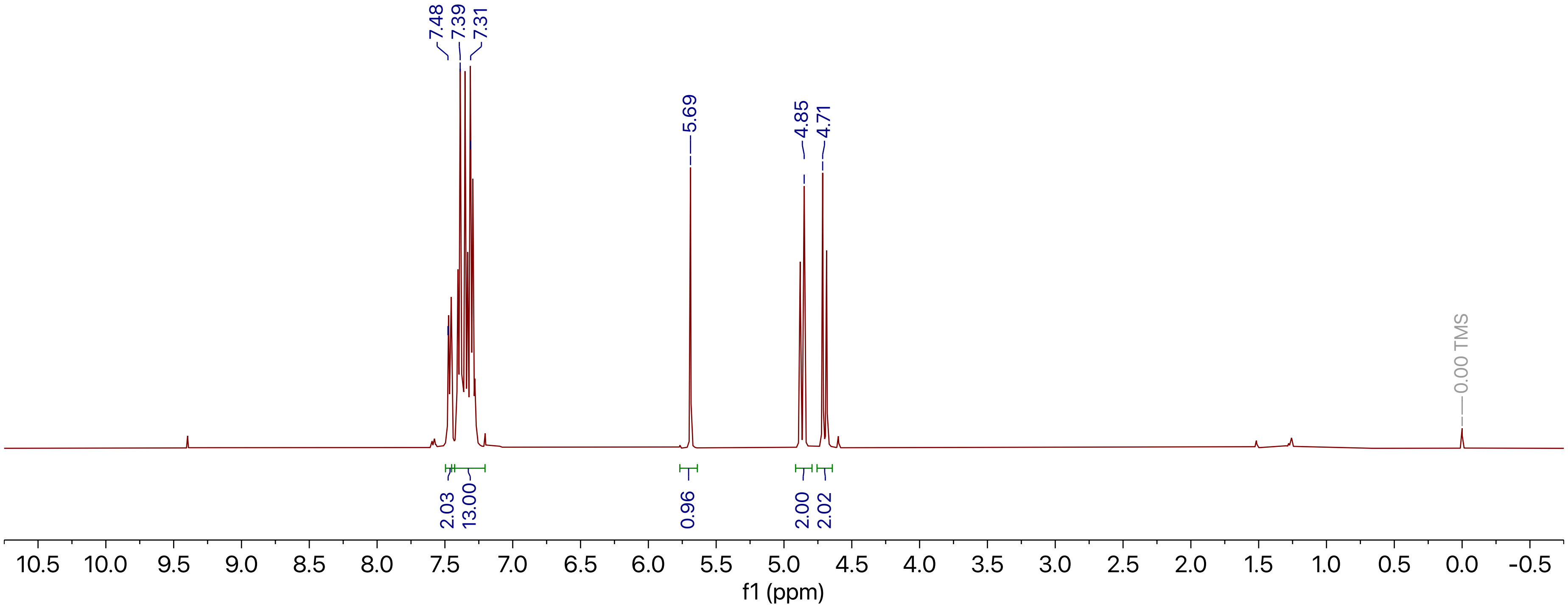

$^{13}C\{^{1}H\}$ NMR (101 MHz, $CDCl_3$)

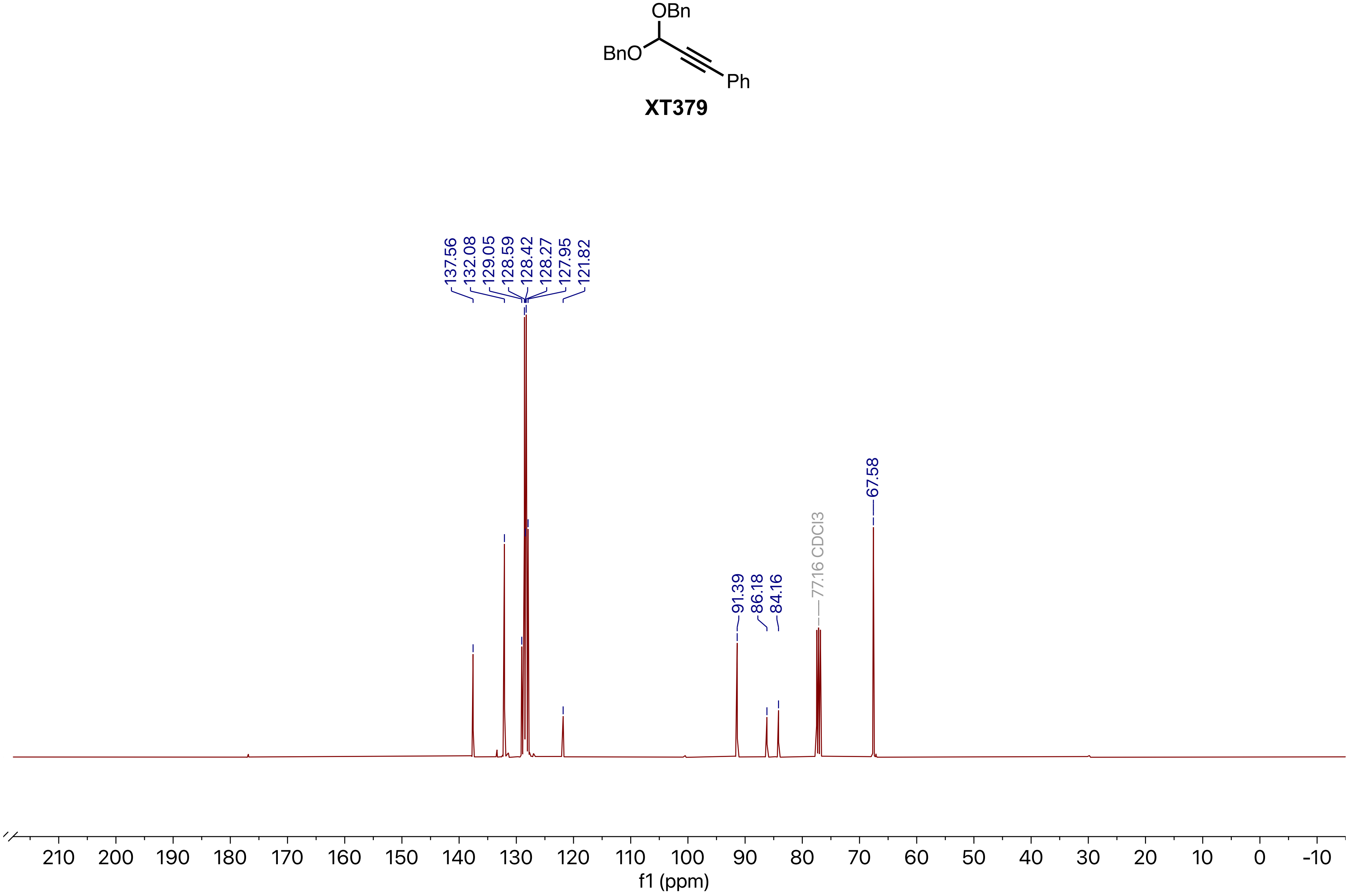

2D ^{1}H–^{1}H COSY (400 MHz, $CDCl_3$)

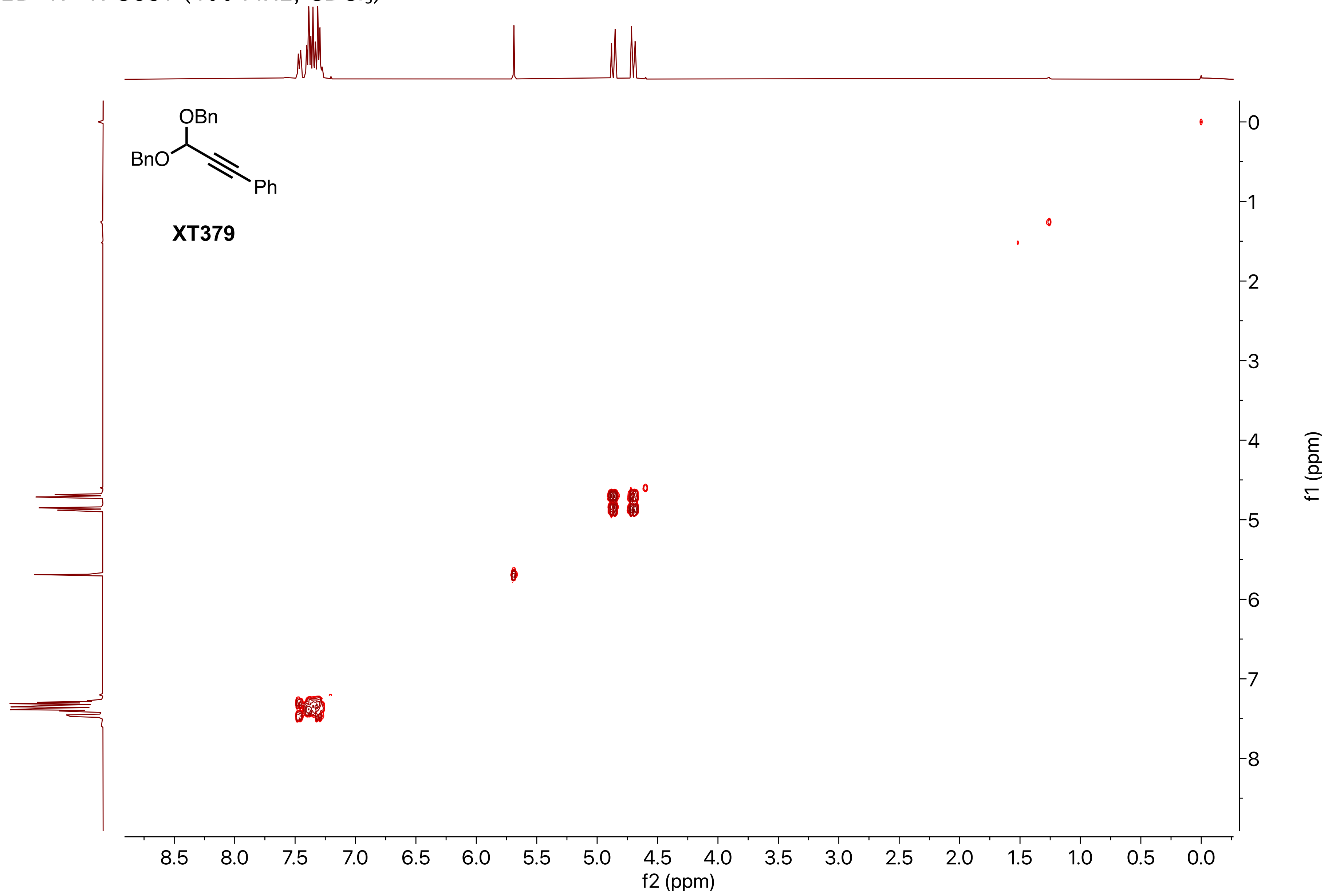

2D ^{1}H–^{13}C HSQC (400 MHz, $CDCl_3$)

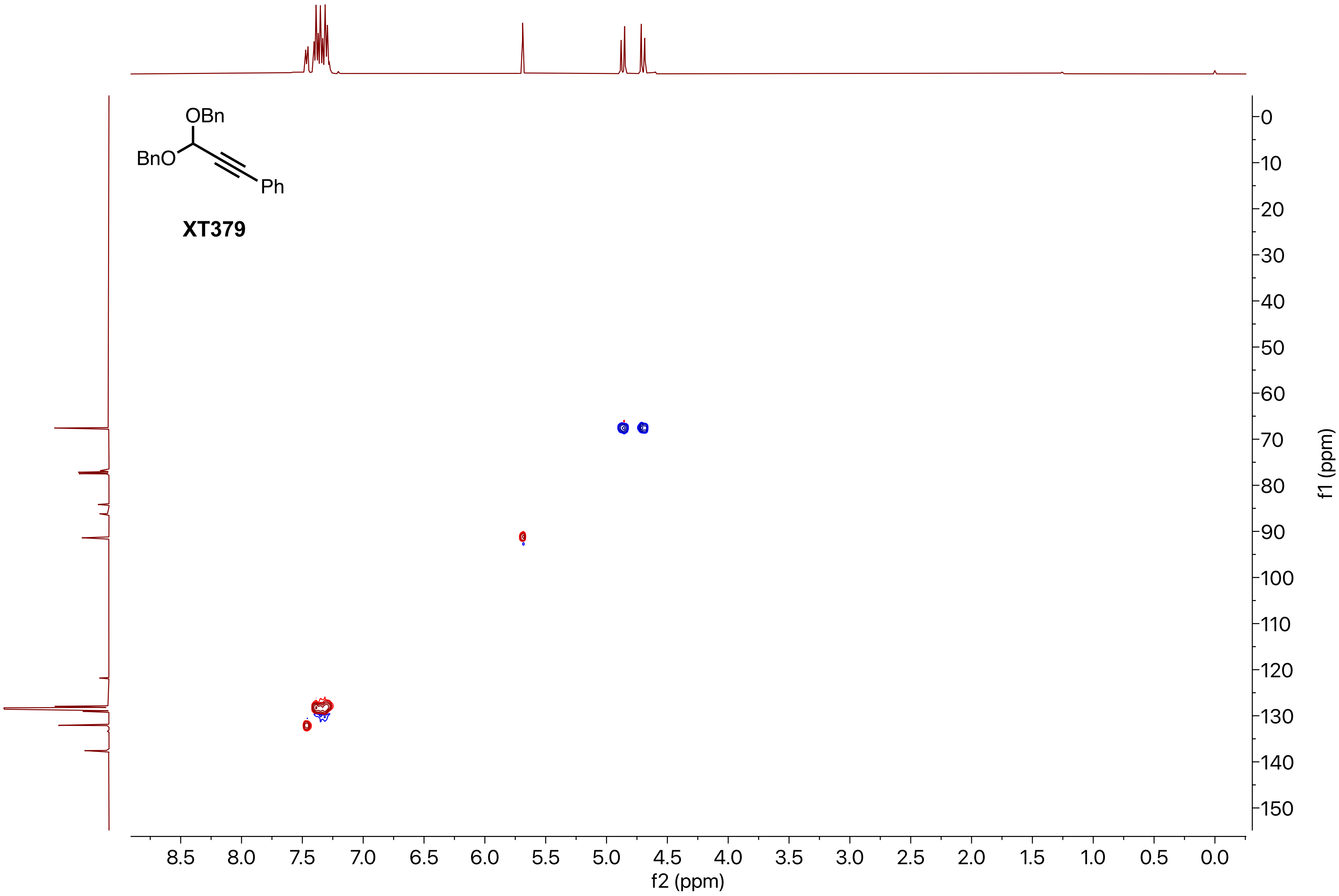

^{1}H NMR (400 MHz, $CDCl_3$)

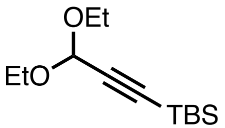

2D

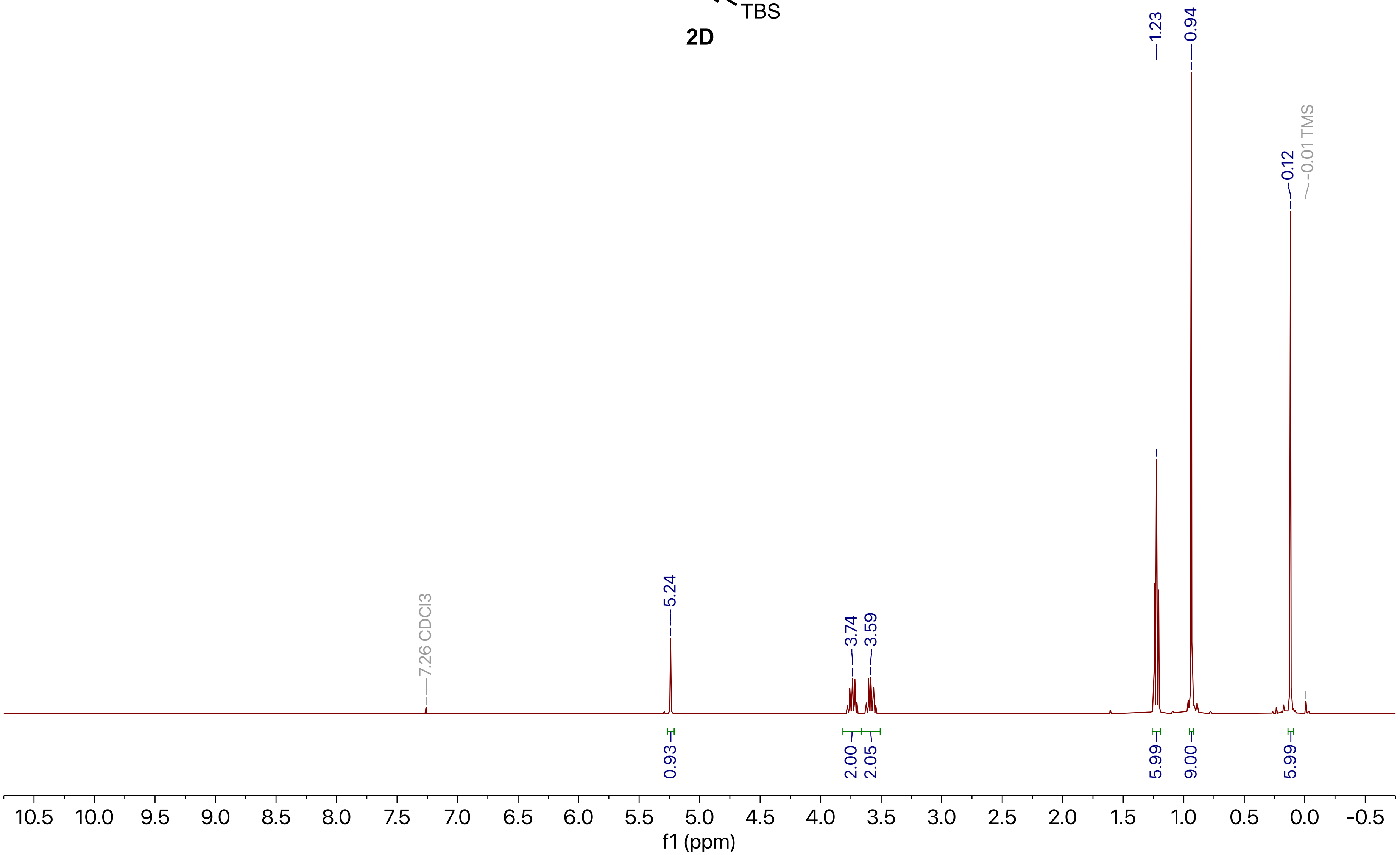

$^{13}C\{^{1}H\}$ NMR (101 MHz, $CDCl_3$)

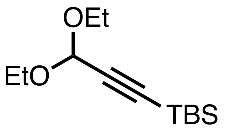

2D

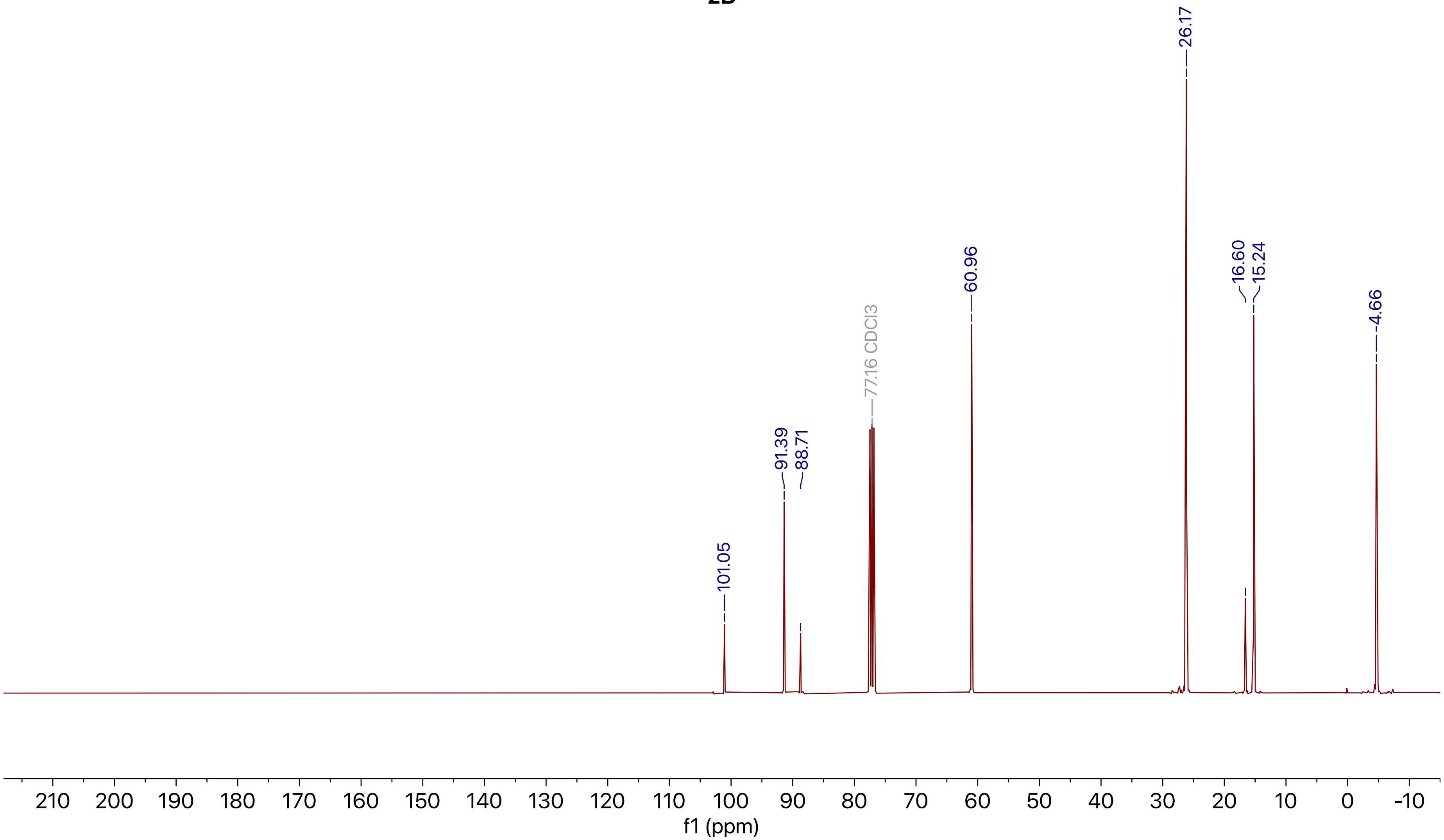

2D ^{1}H–^{1}H COSY (400 MHz, $CDCl_3$)

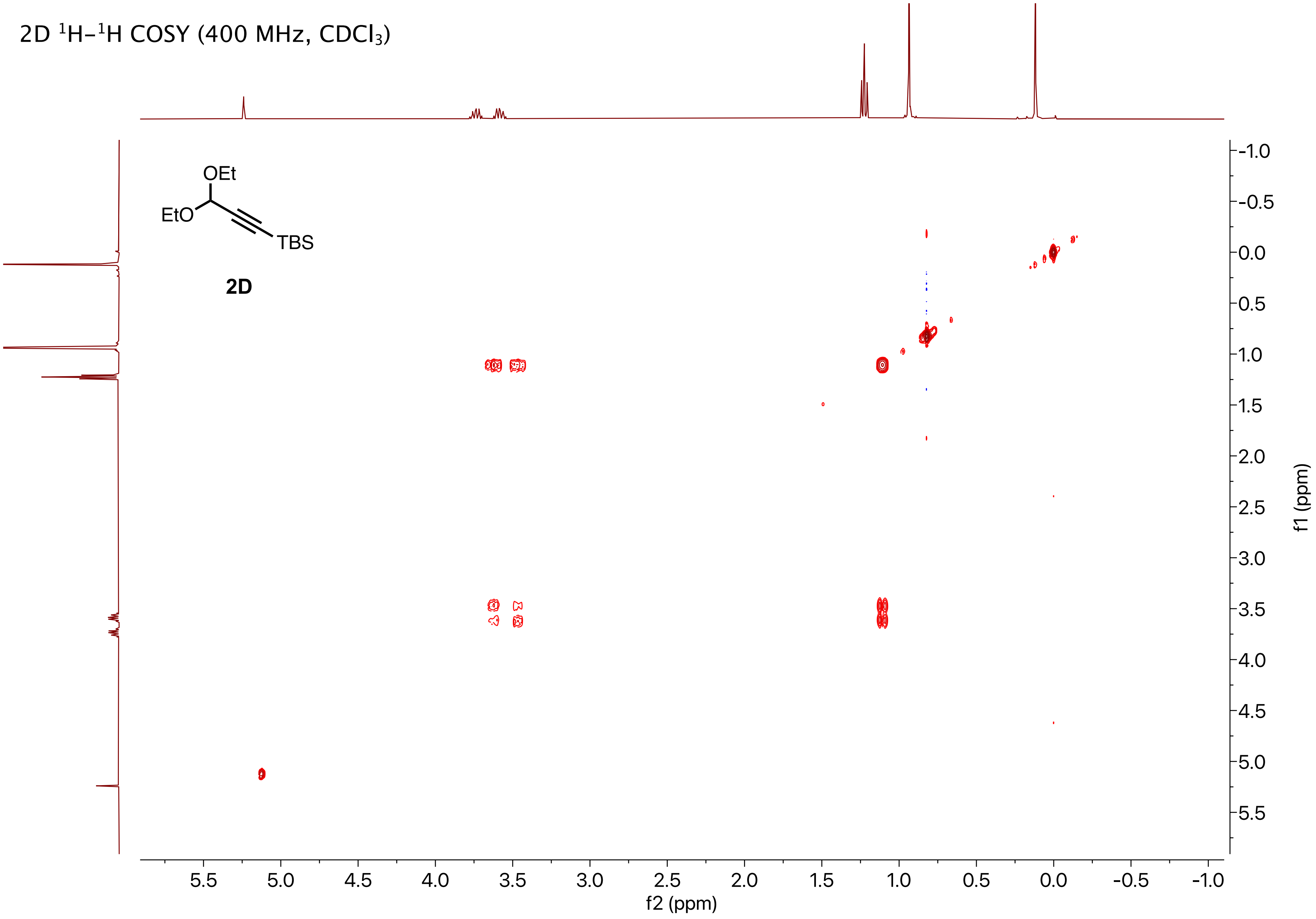

2D ^{1}H–^{13}C HSQC (400 MHz, $CDCl_3$)

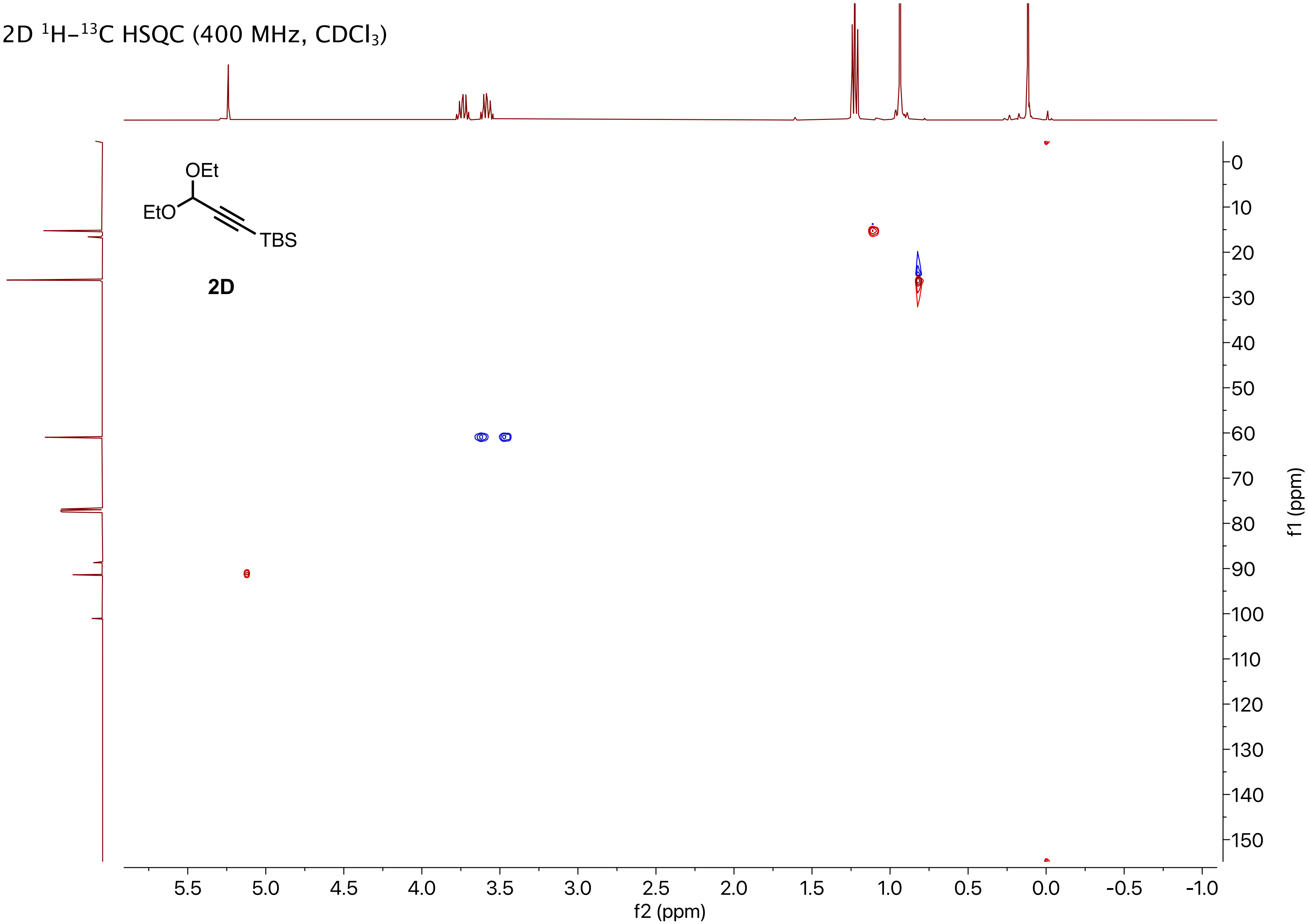

^{1}H NMR (400 MHz, $CDCl_3$)

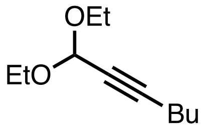

2E

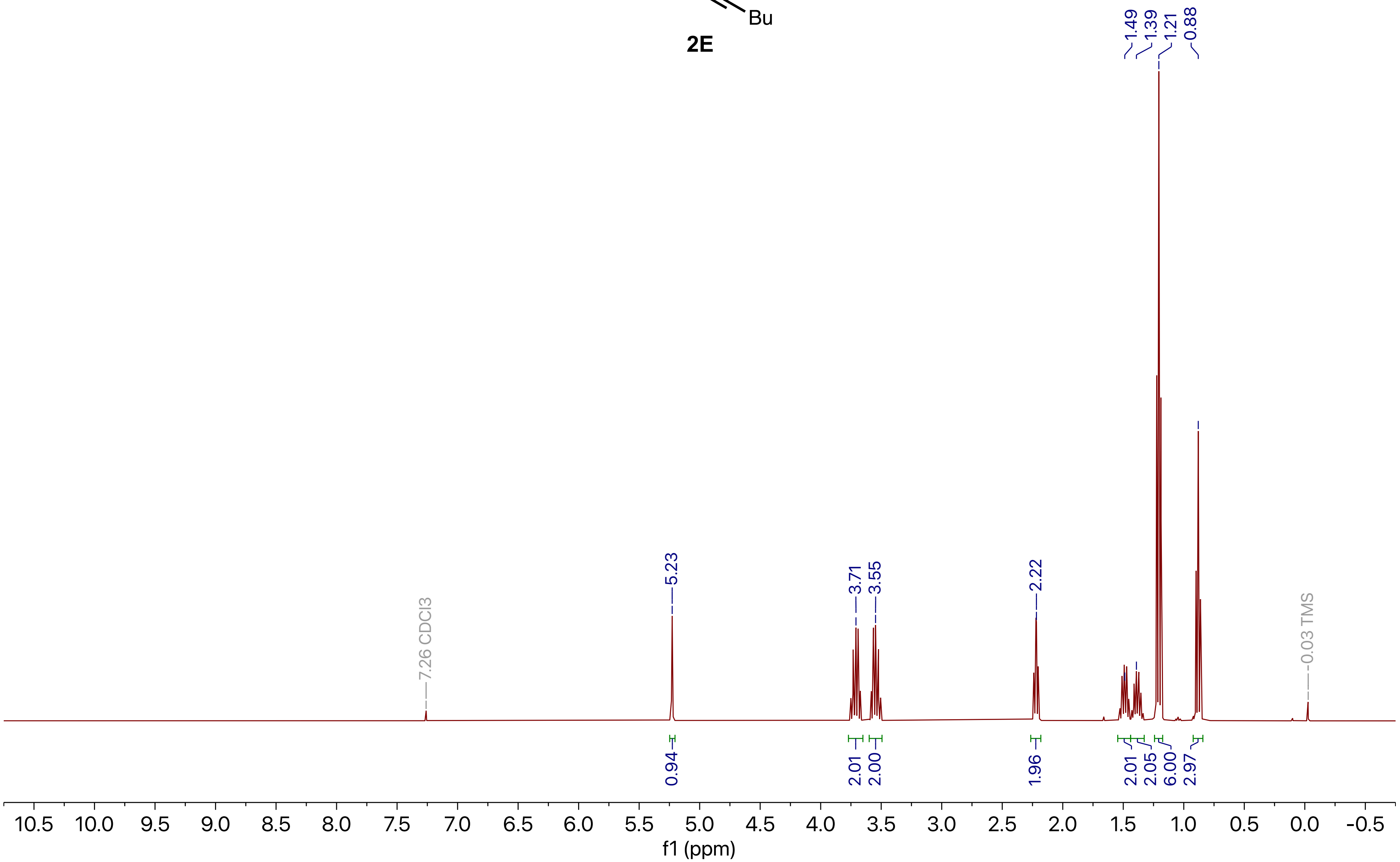

$^{13}C\{^{1}H\}$ NMR (101 MHz, $CDCl_3$)

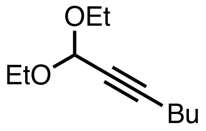

2E

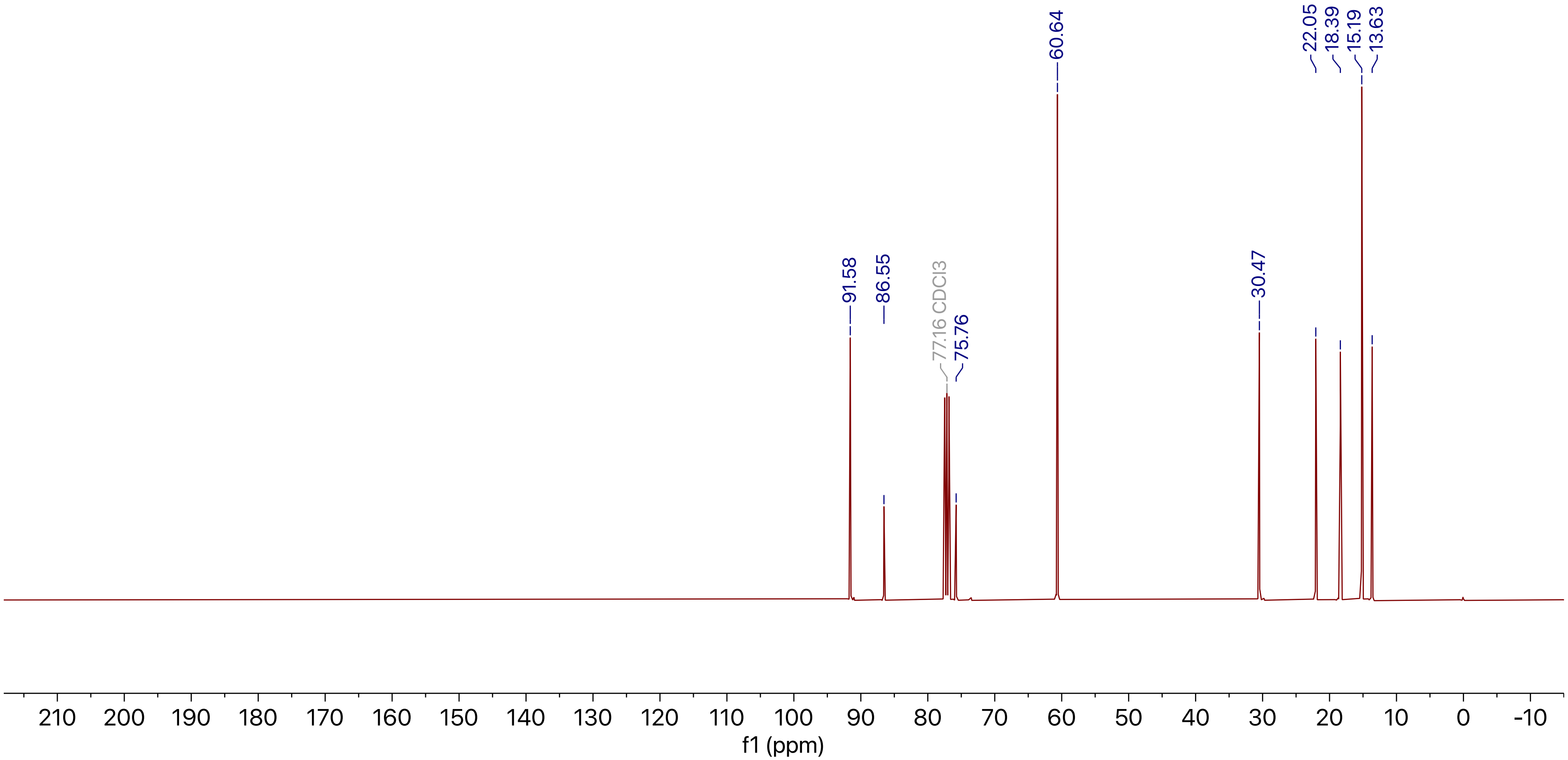

2D ^{1}H–^{1}H COSY (400 MHz, $CDCl_3$)

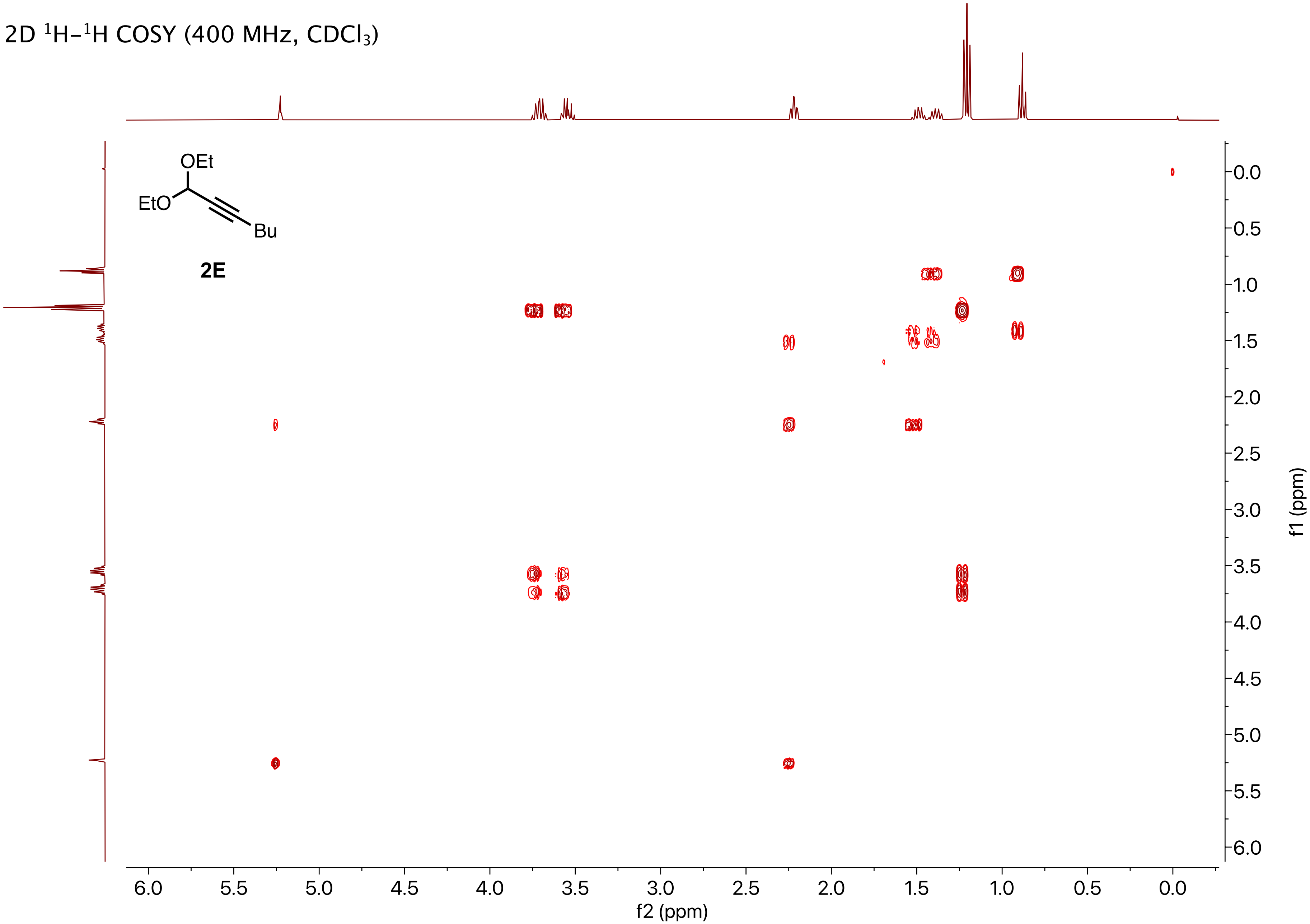

2D ^{1}H–^{13}C HSQC (400 MHz, $CDCl_3$)

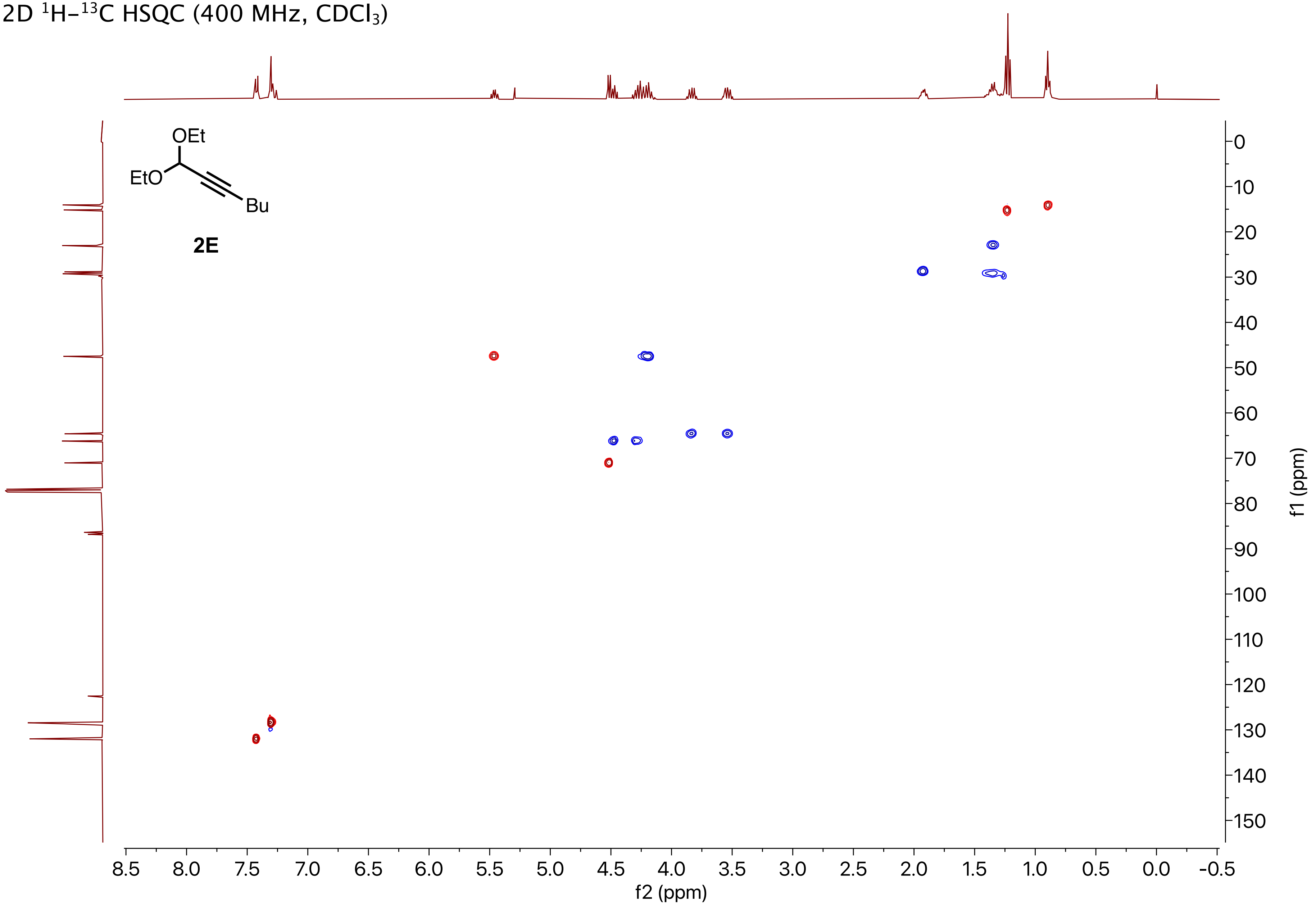

^{1}H NMR (400 MHz, $CDCl_3$)

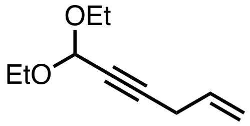

2F

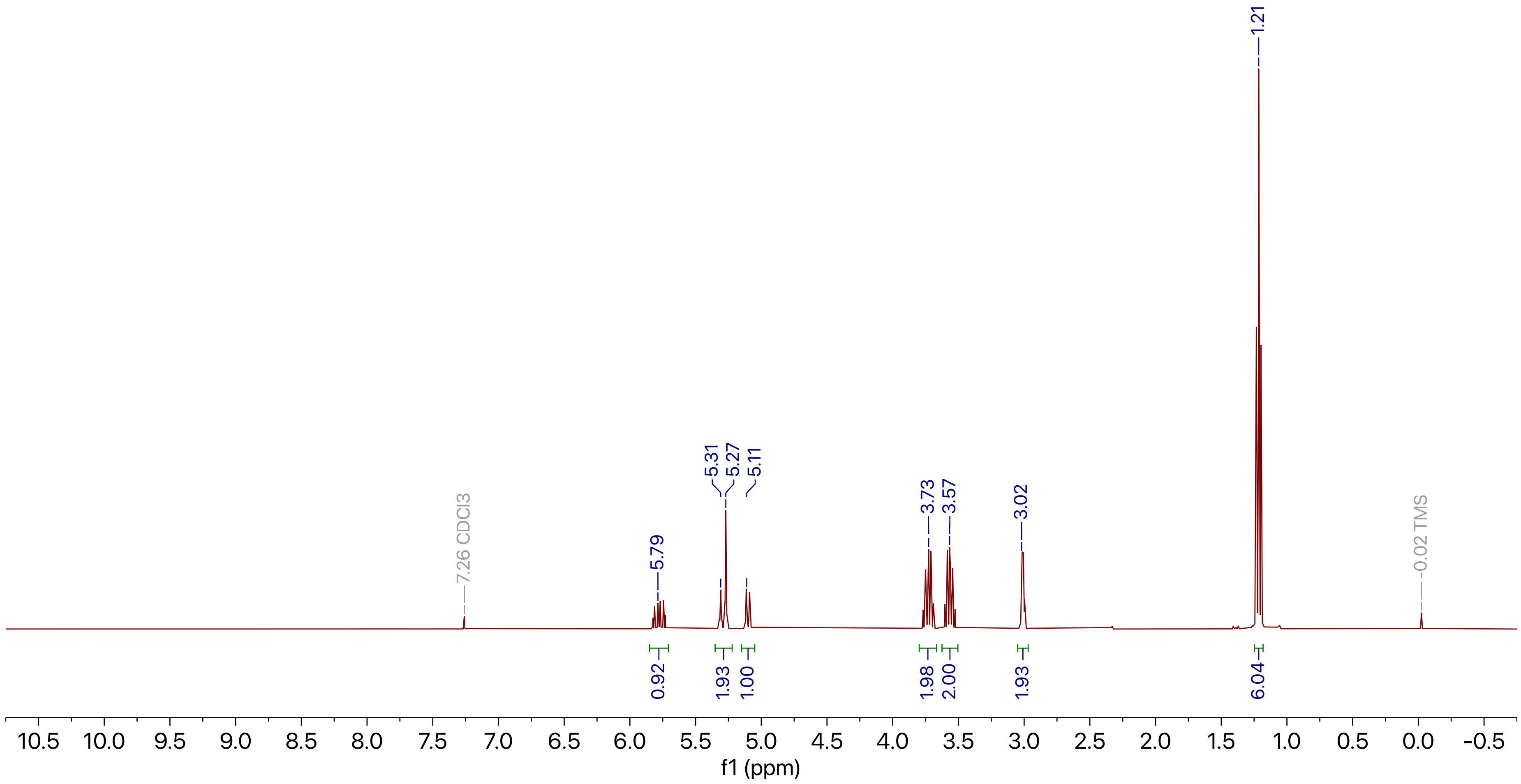

$^{13}C\{^{1}H\}$ NMR (101 MHz, $CDCl_3$)

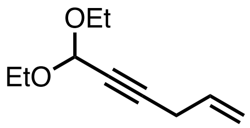

2F

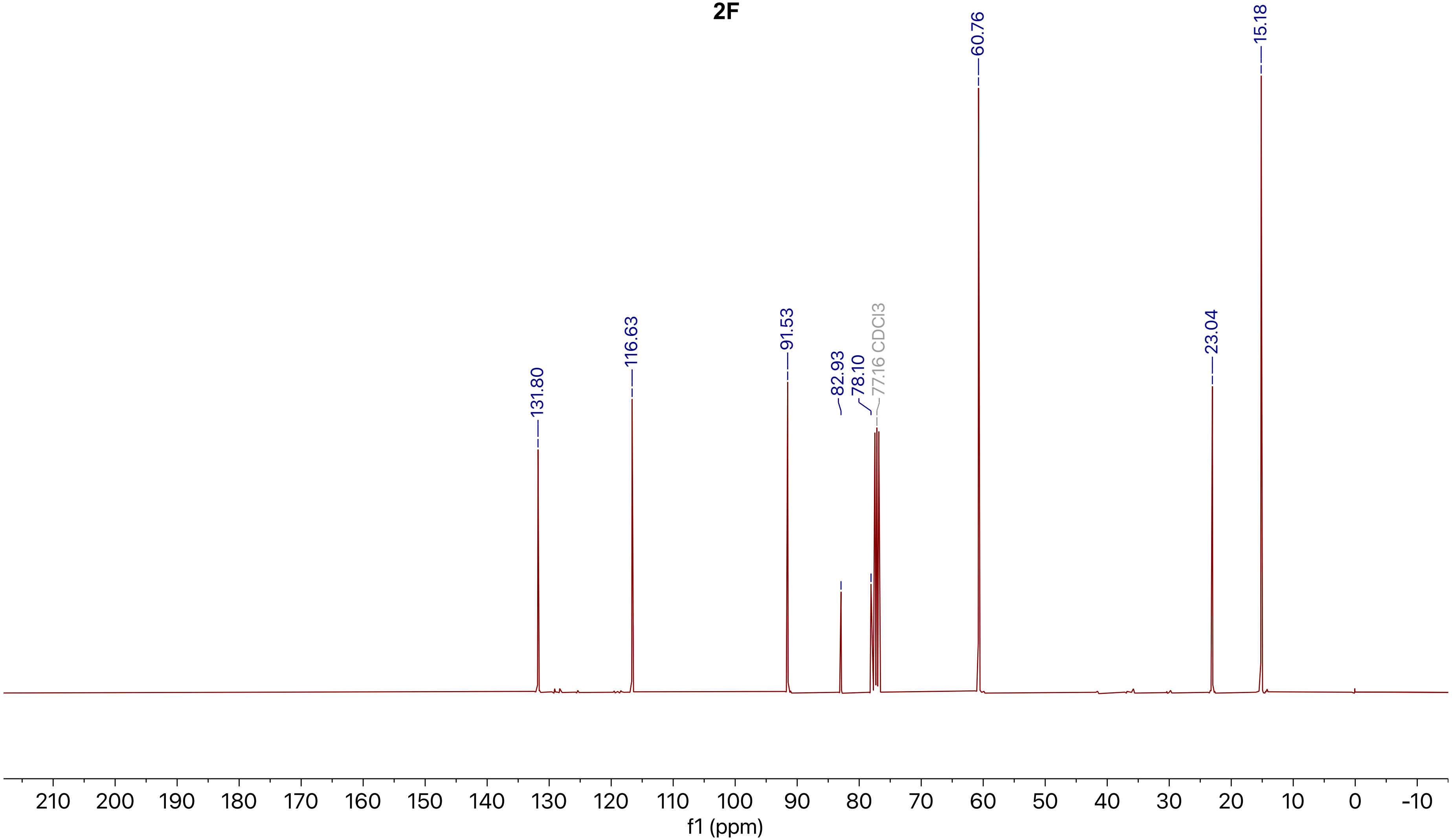

2D ^{1}H–^{1}H COSY (400 MHz, $CDCl_3$)

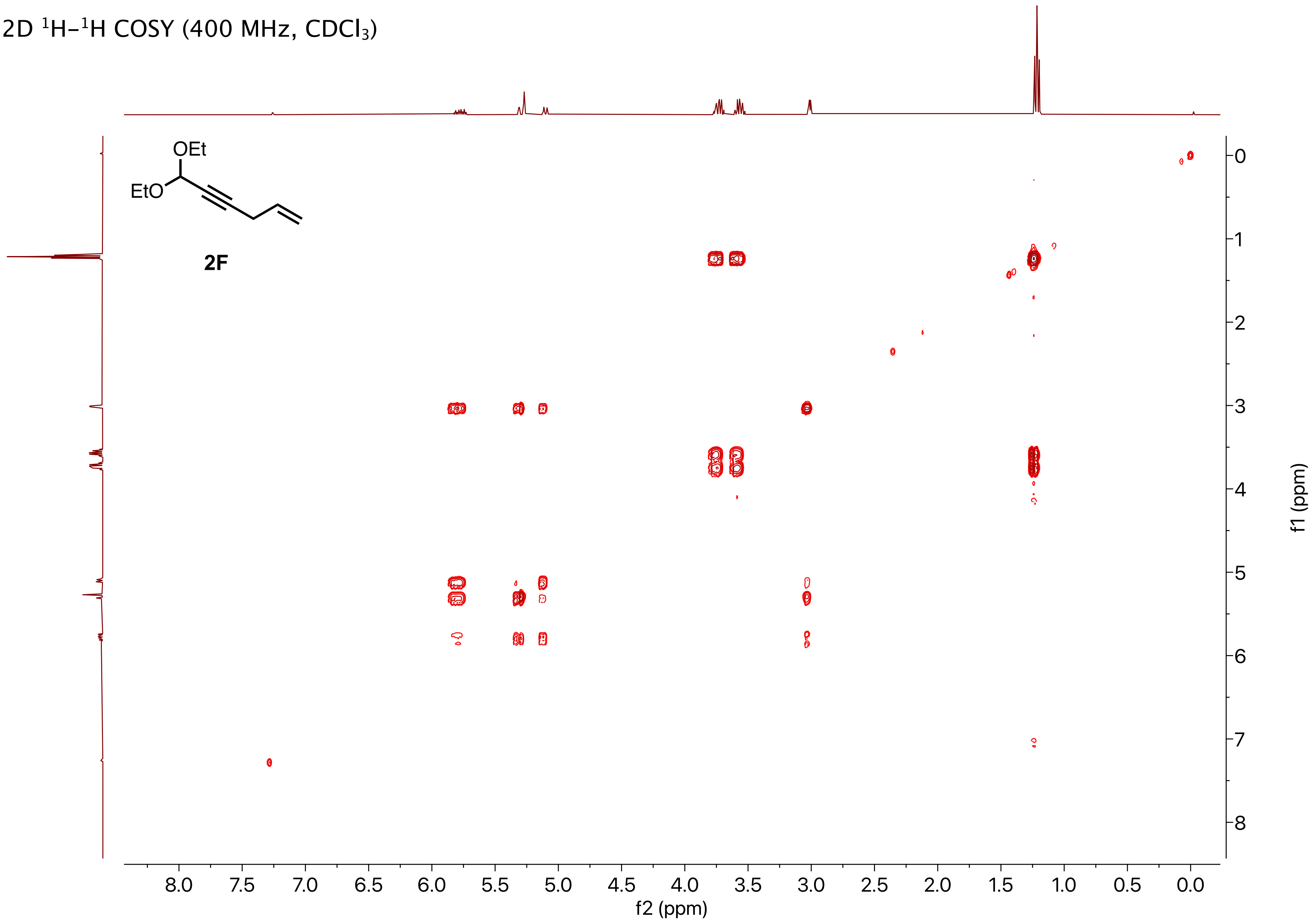

2D ^{1}H–^{13}C HSQC (400 MHz, $CDCl_3$)

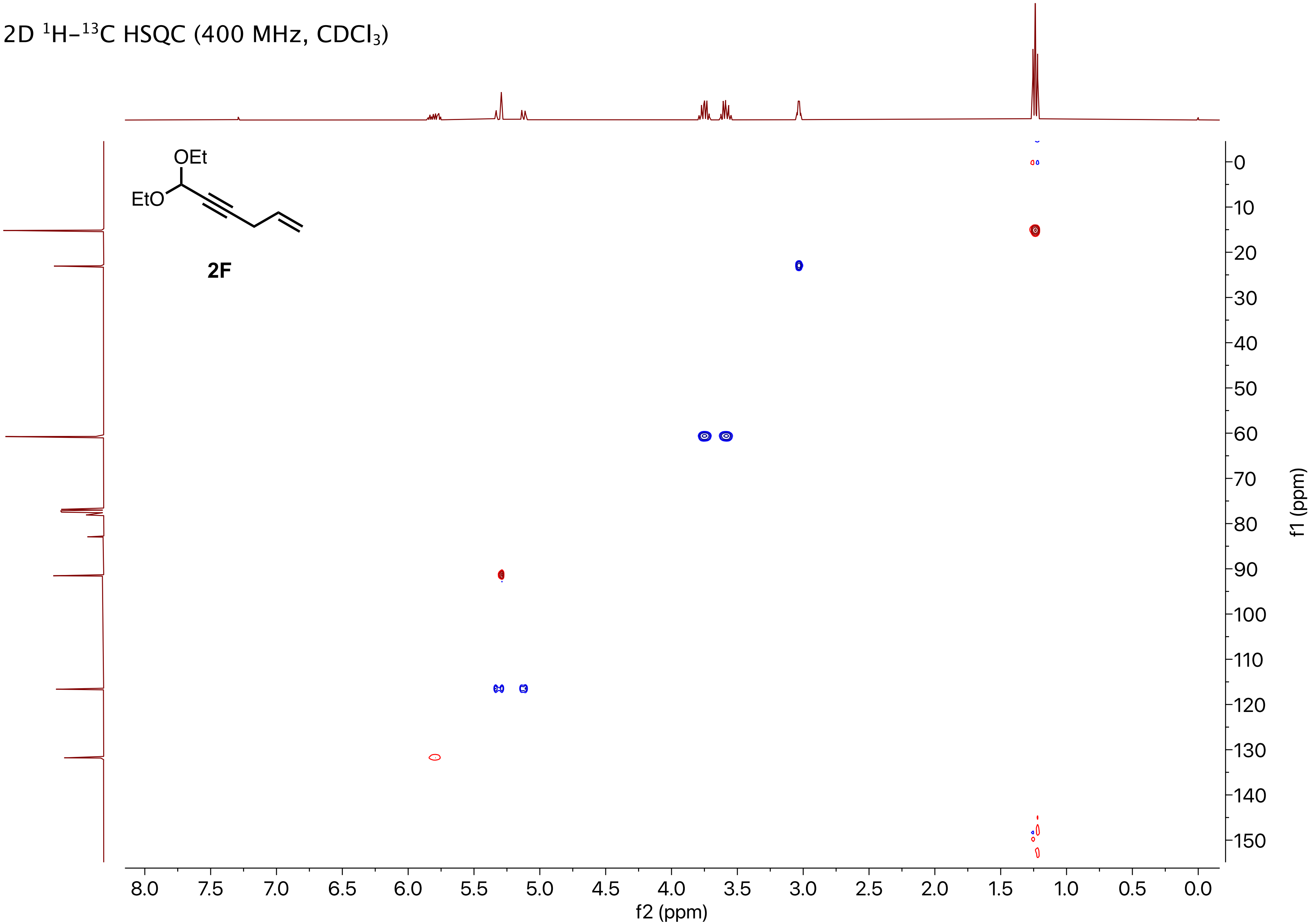

2F

^{1}H NMR (400 MHz, $CDCl_3$)

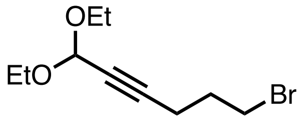

2G

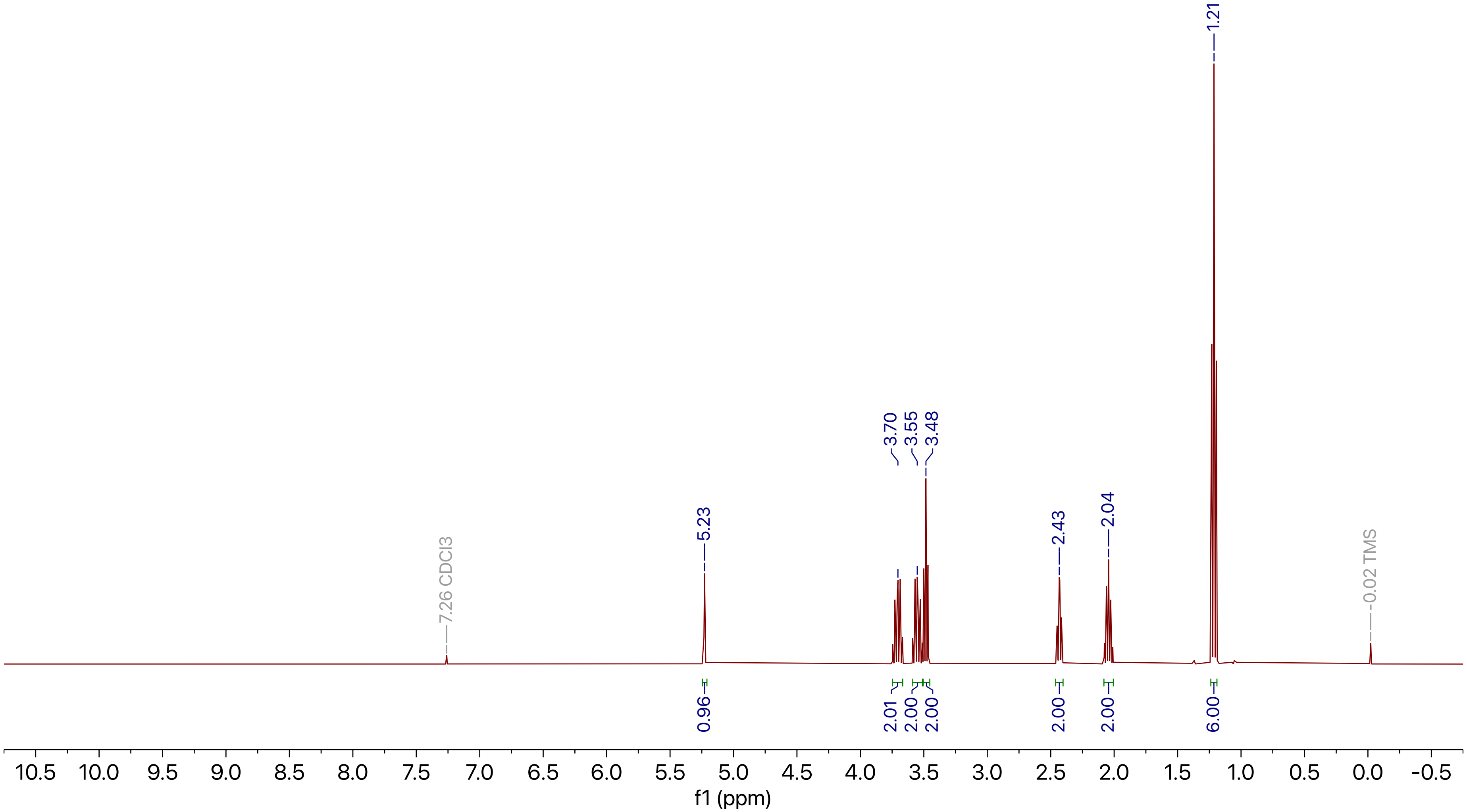

$^{13}C\{^{1}H\}$ NMR (101 MHz, $CDCl_3$)

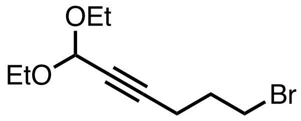

2G

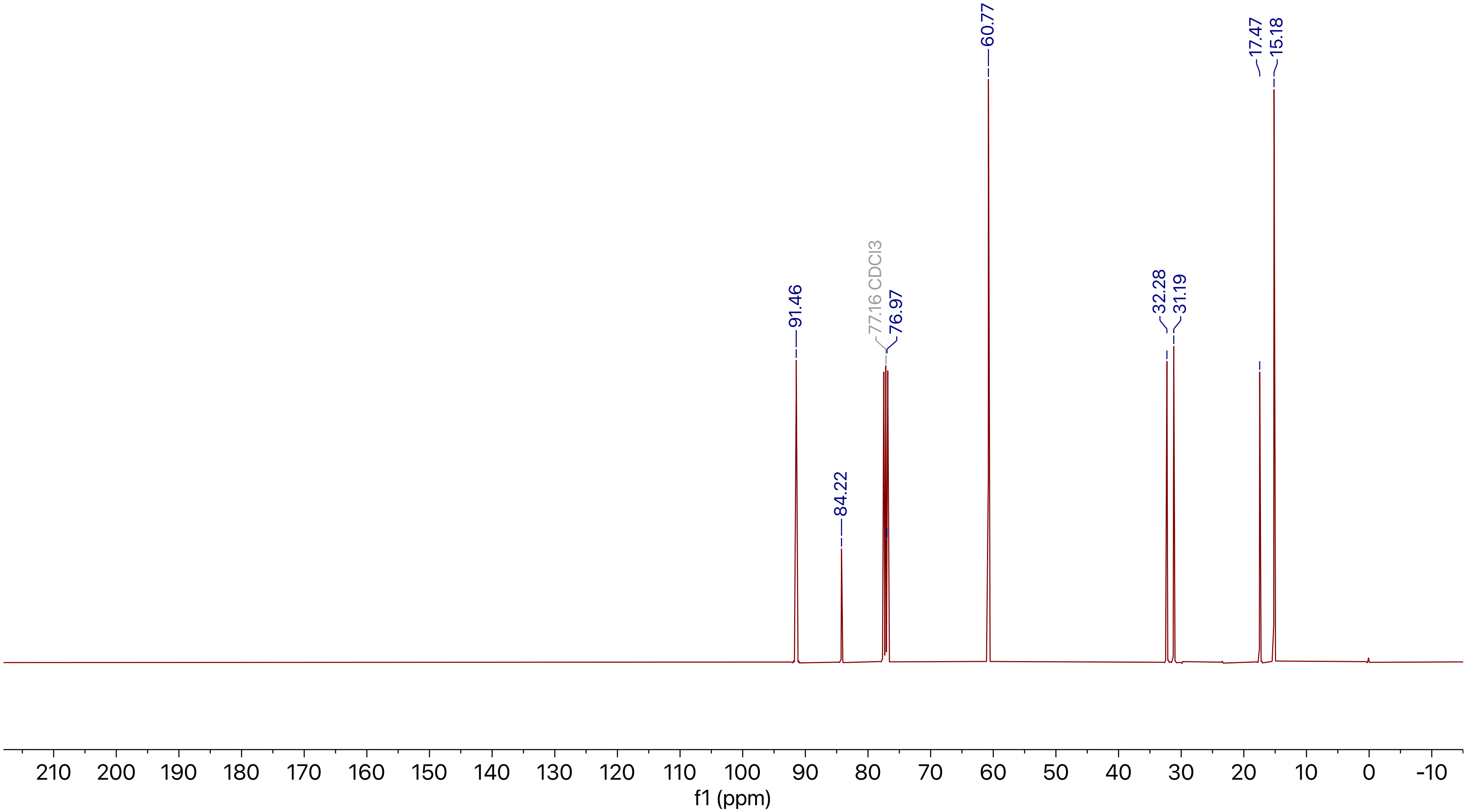

2D ^1H–^1H COSY (400 MHz, $CDCl_3$)

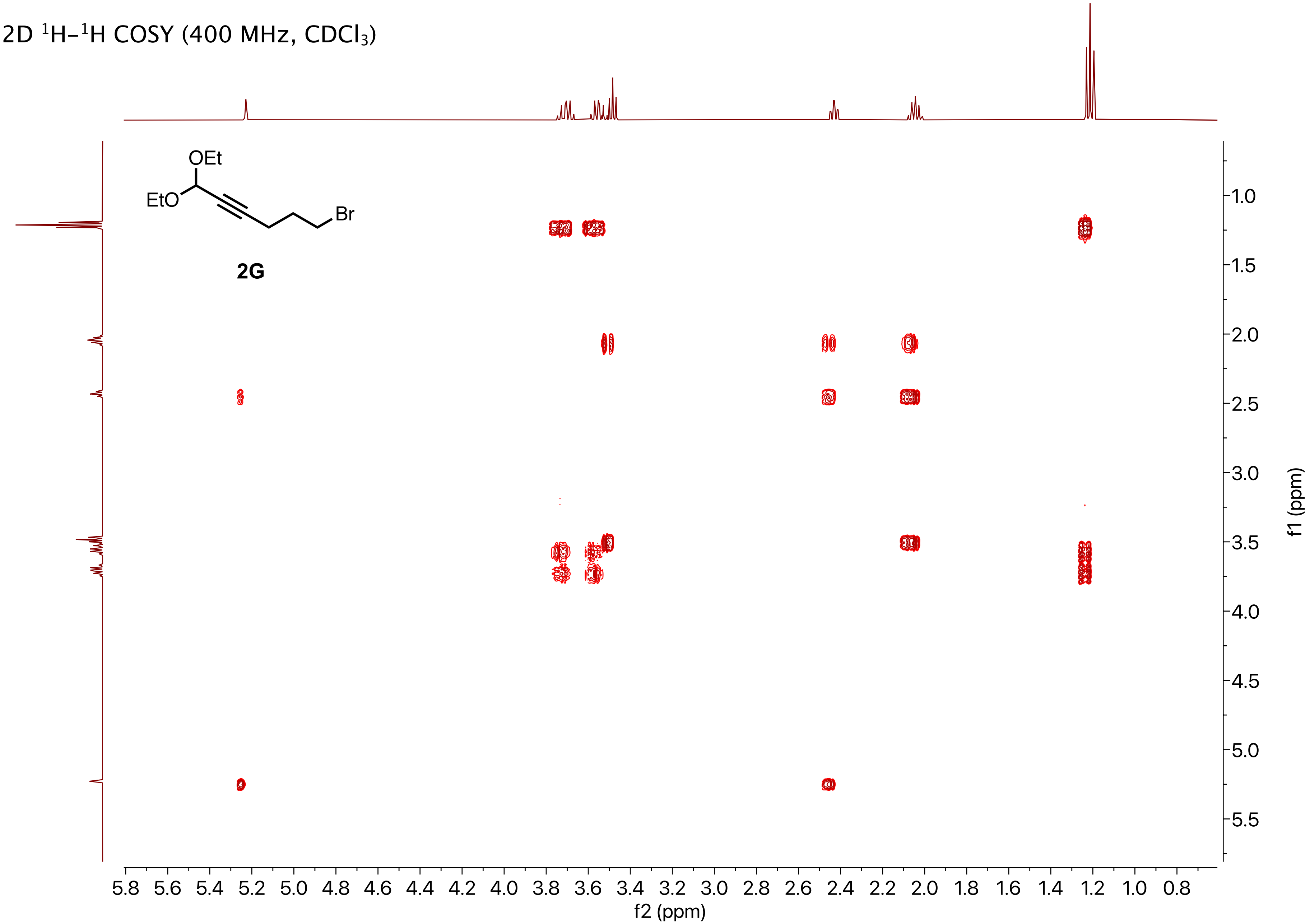

2D ^{1}H–^{13}C HSQC (400 MHz, $CDCl_3$)

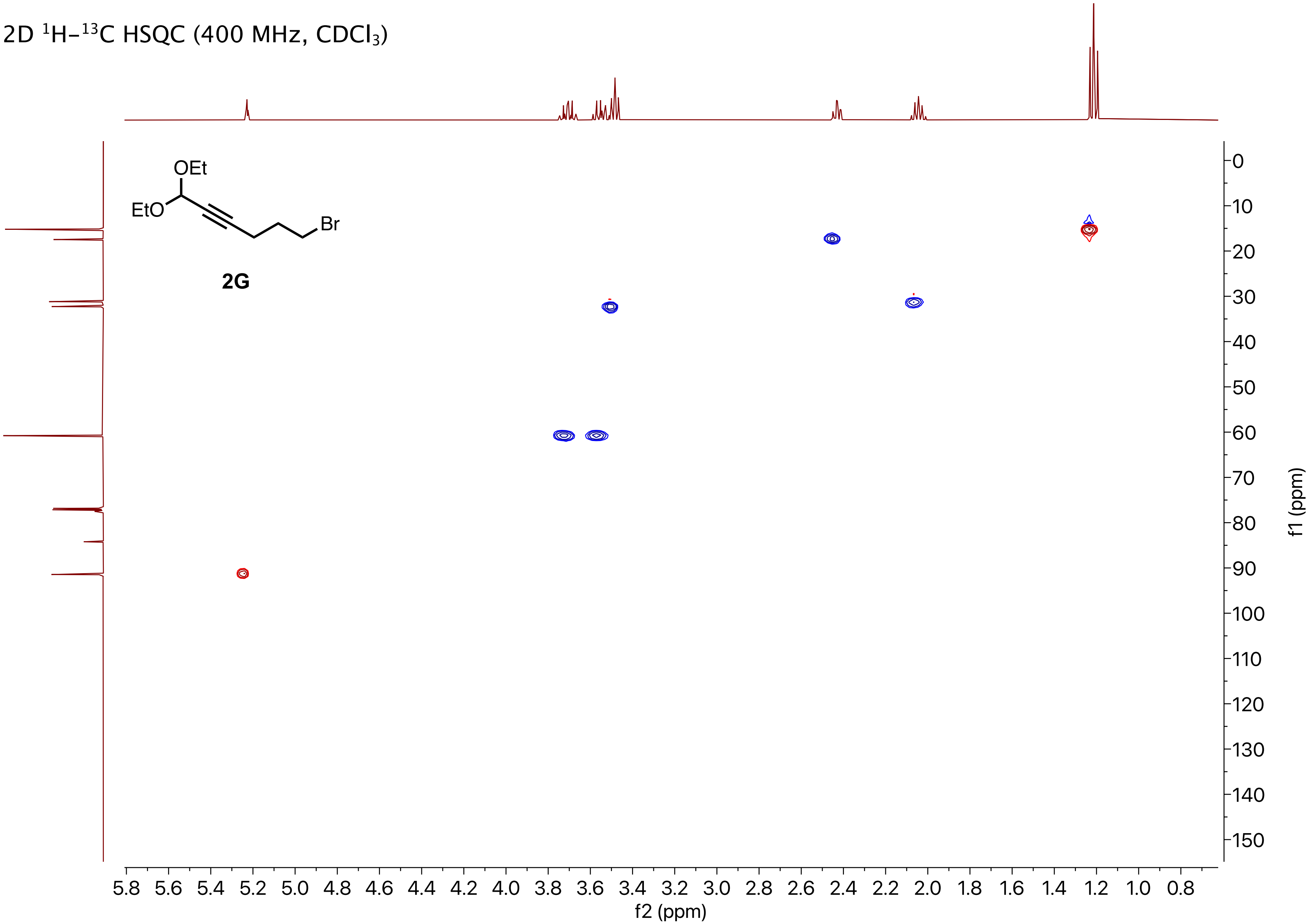

^{1}H NMR (400 MHz, $CDCl_3$)

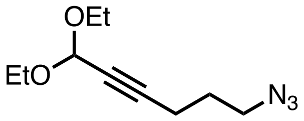

2H

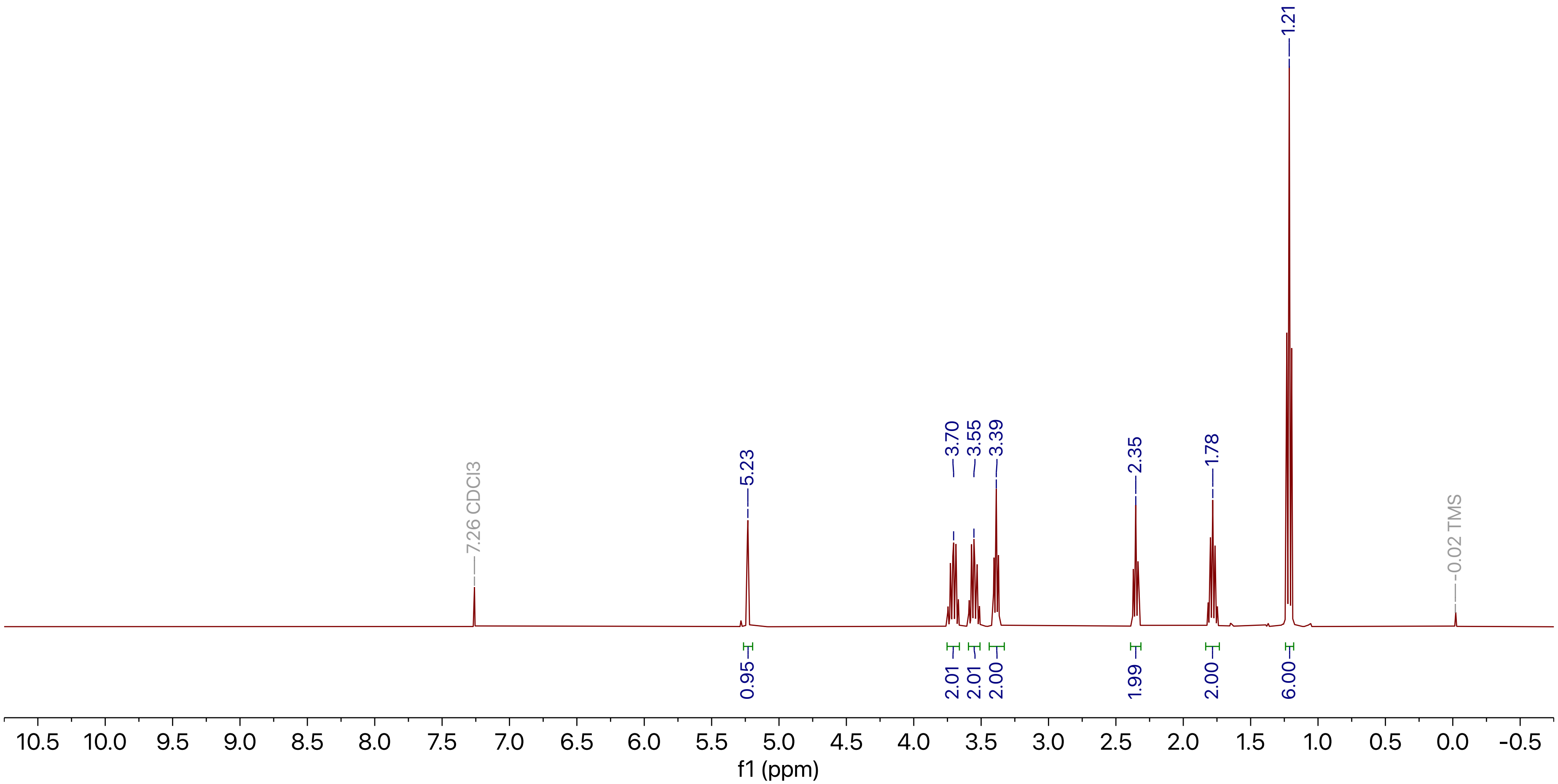

$^{13}C\{^1H\}$ NMR (101 MHz, $CDCl_3$)

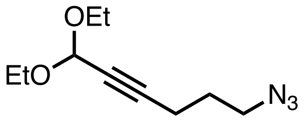

2H

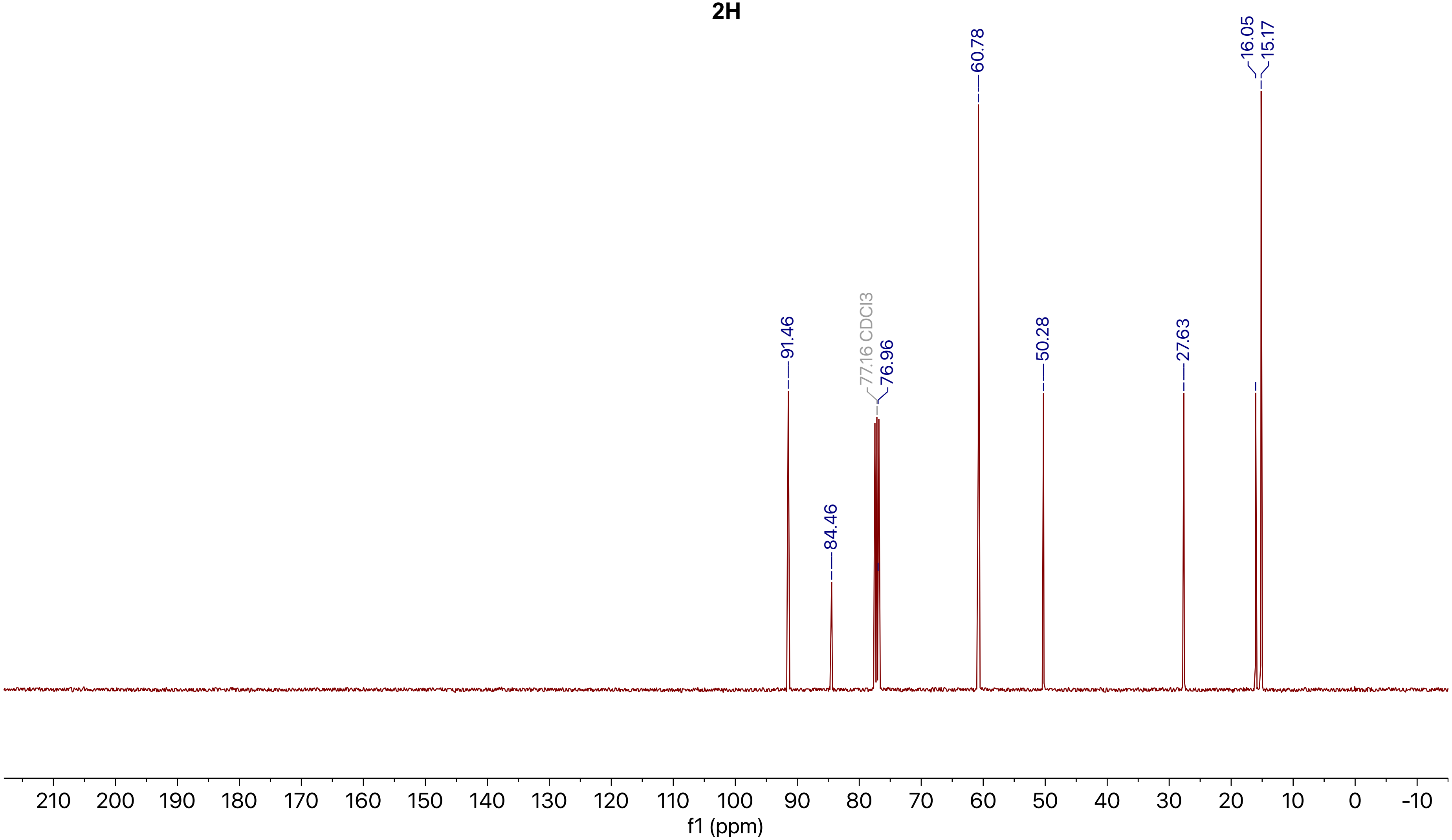

2D ^1H–^1H COSY (400 MHz, $CDCl_3$)

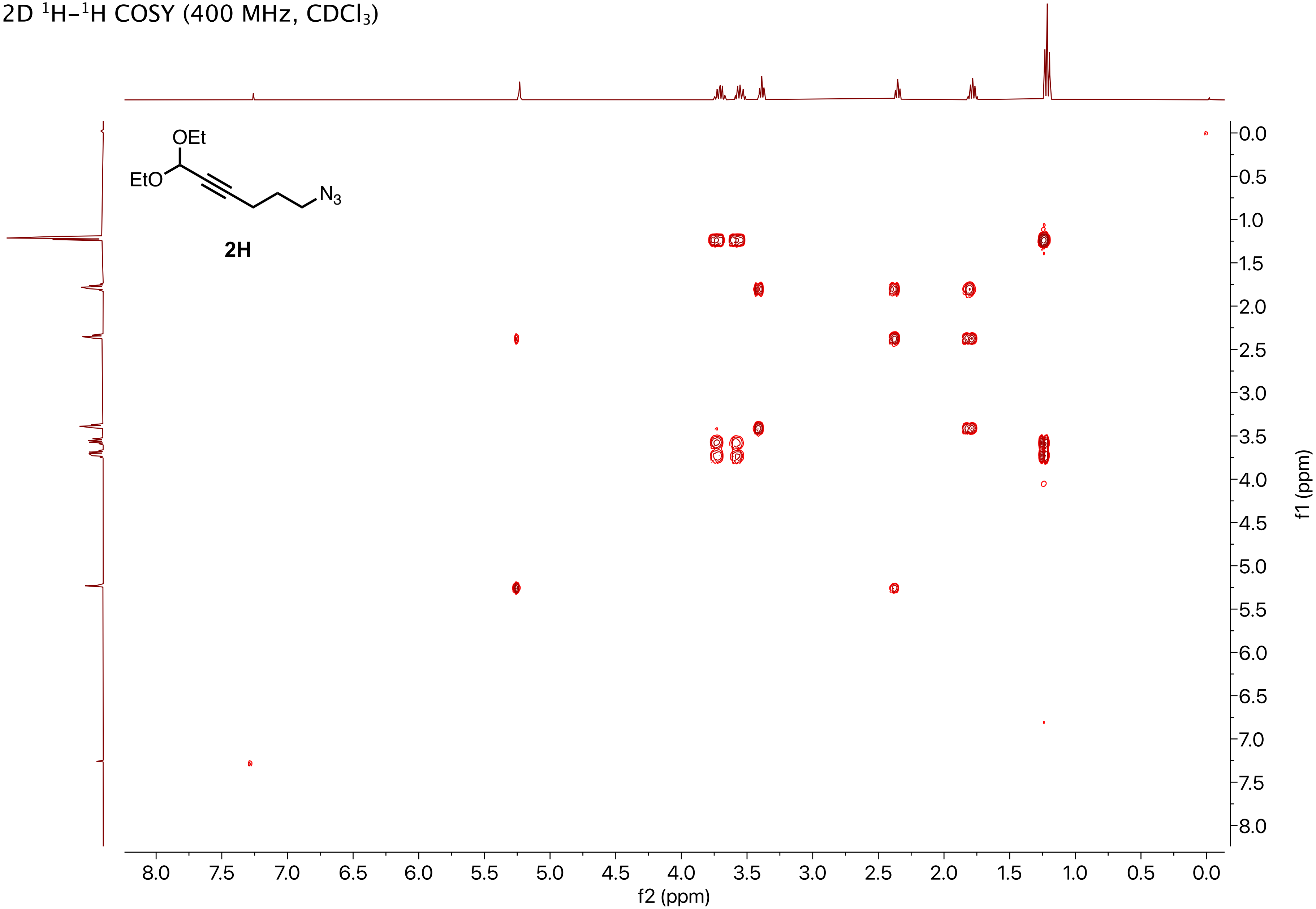

2D ^{1}H–^{13}C HSQC (400 MHz, $CDCl_3$)

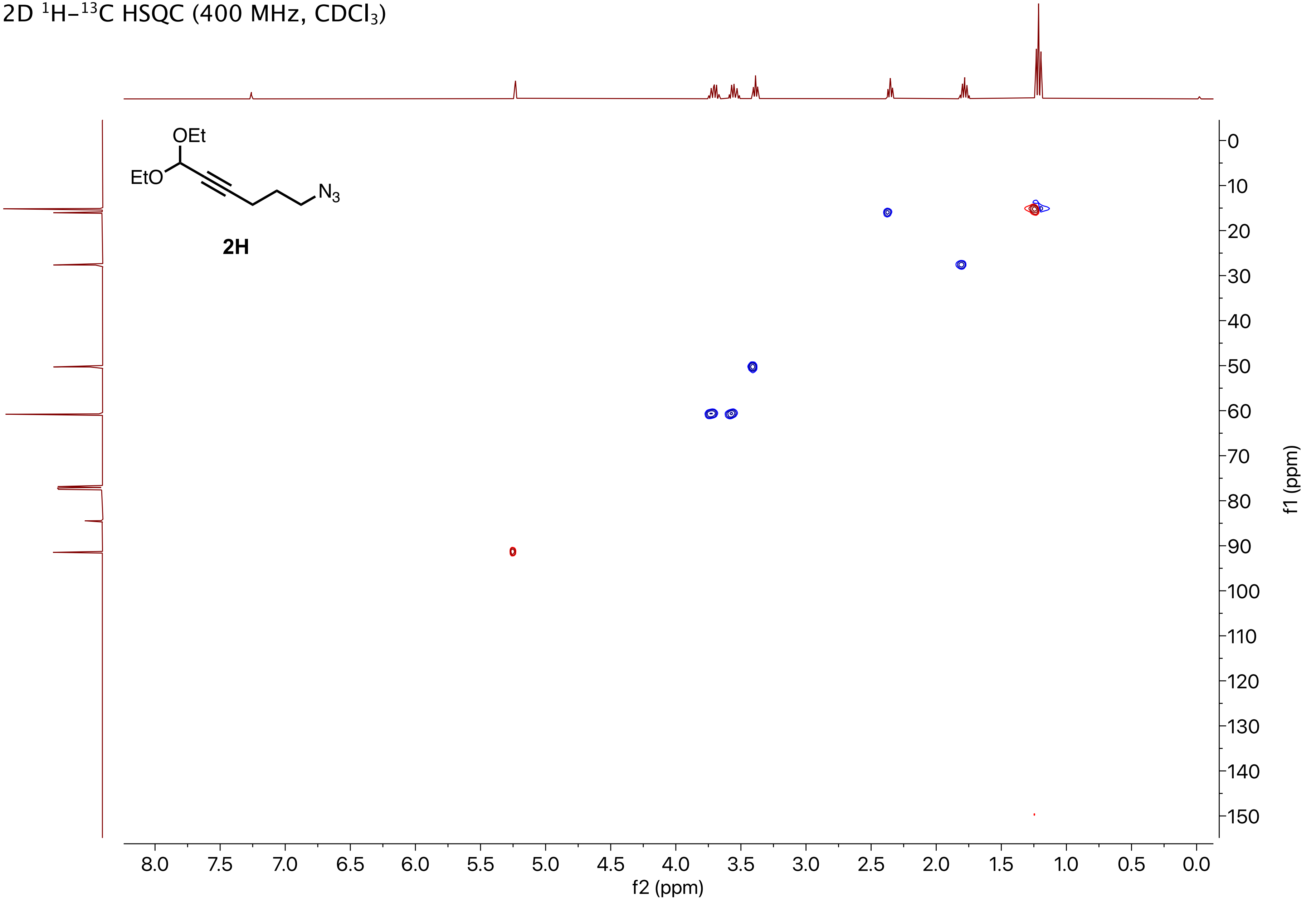

^{1}H NMR (400 MHz, $CDCl_3$)

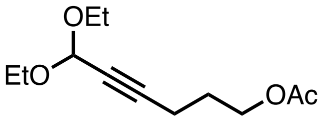

2L

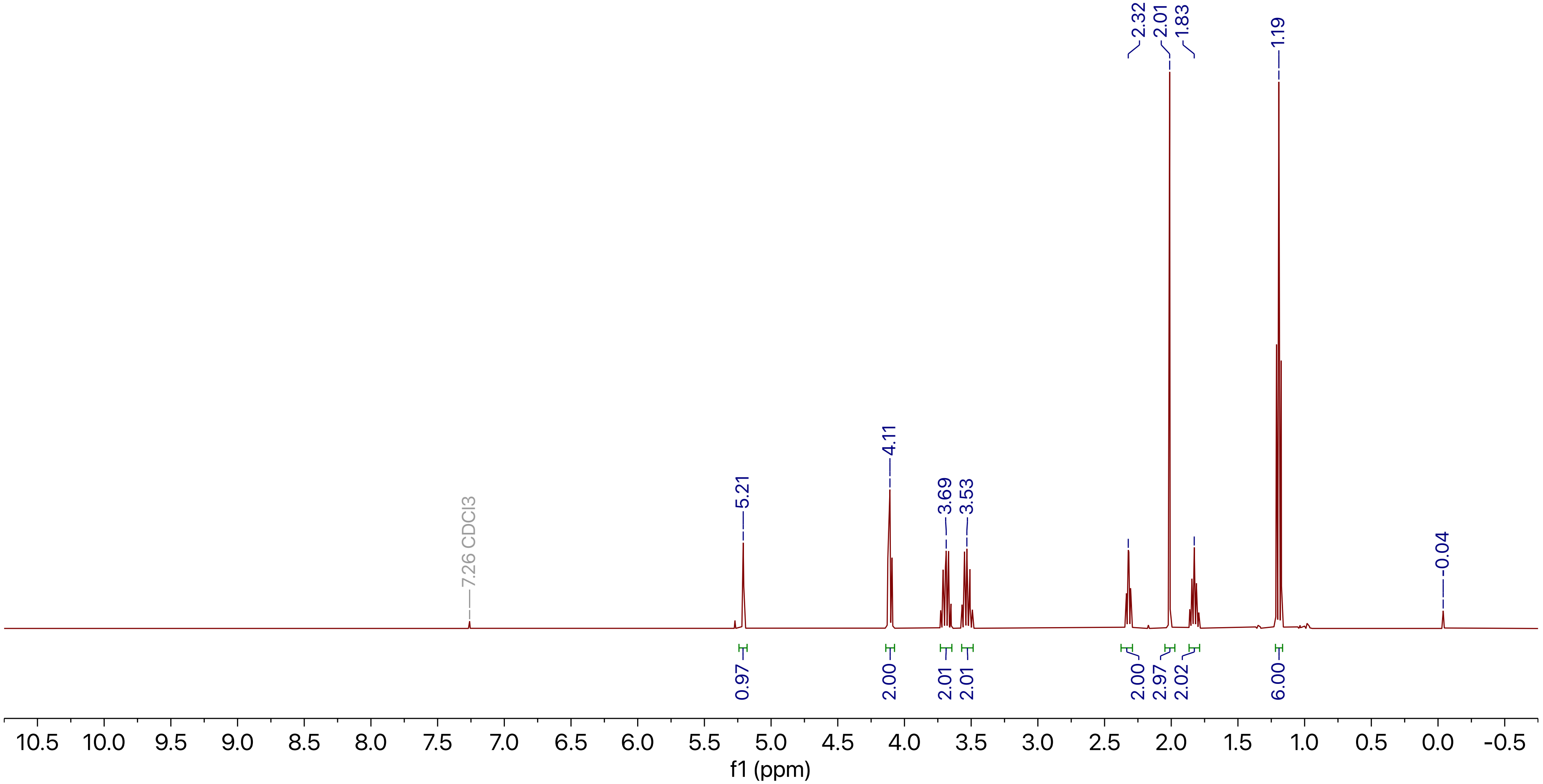

$^{13}C\{^{1}H\}$ NMR (101 MHz, $CDCl_3$)

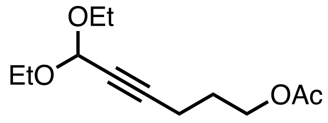

2L

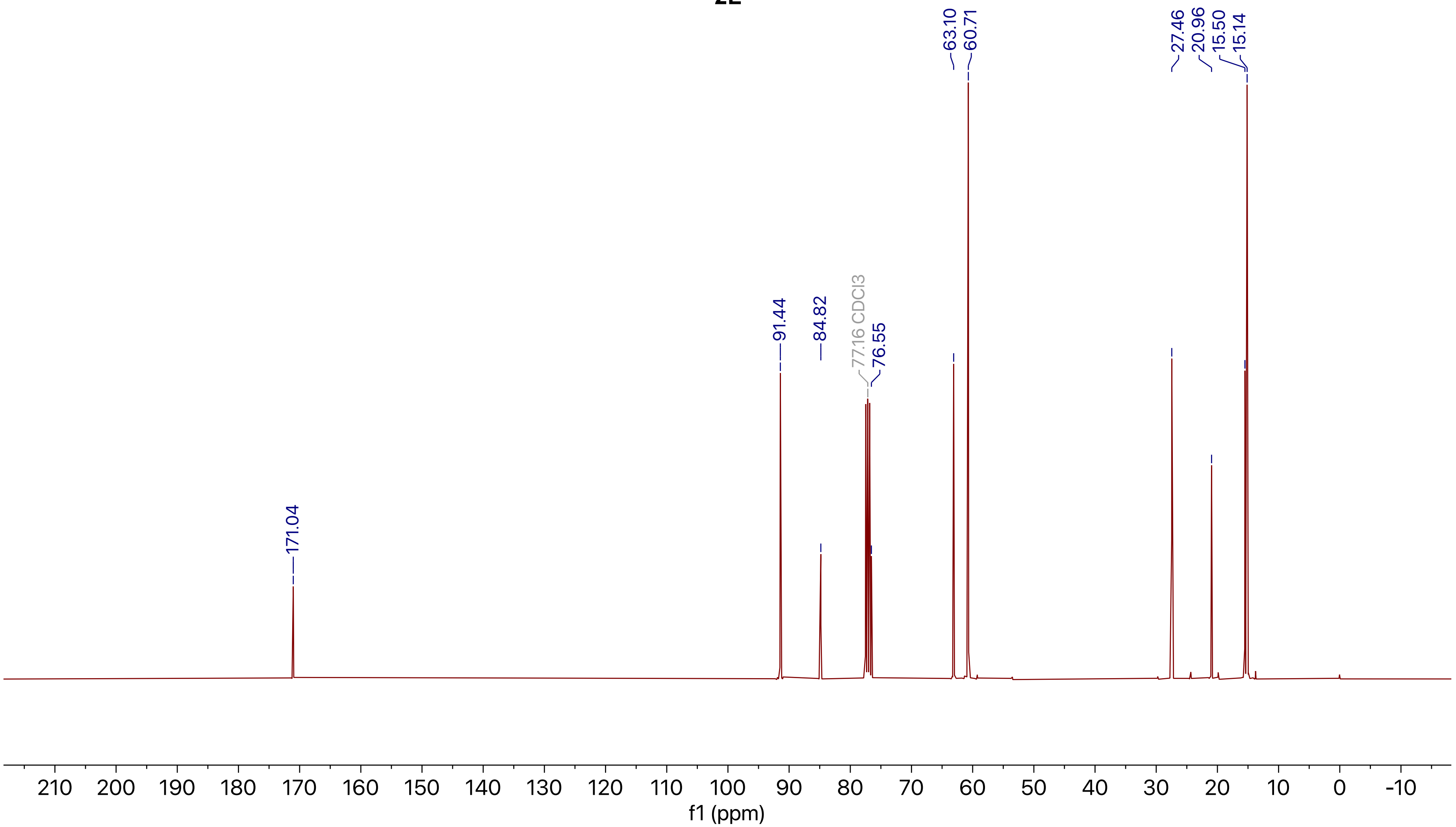

2D ^{1}H–^{1}H COSY (400 MHz, $CDCl_3$)

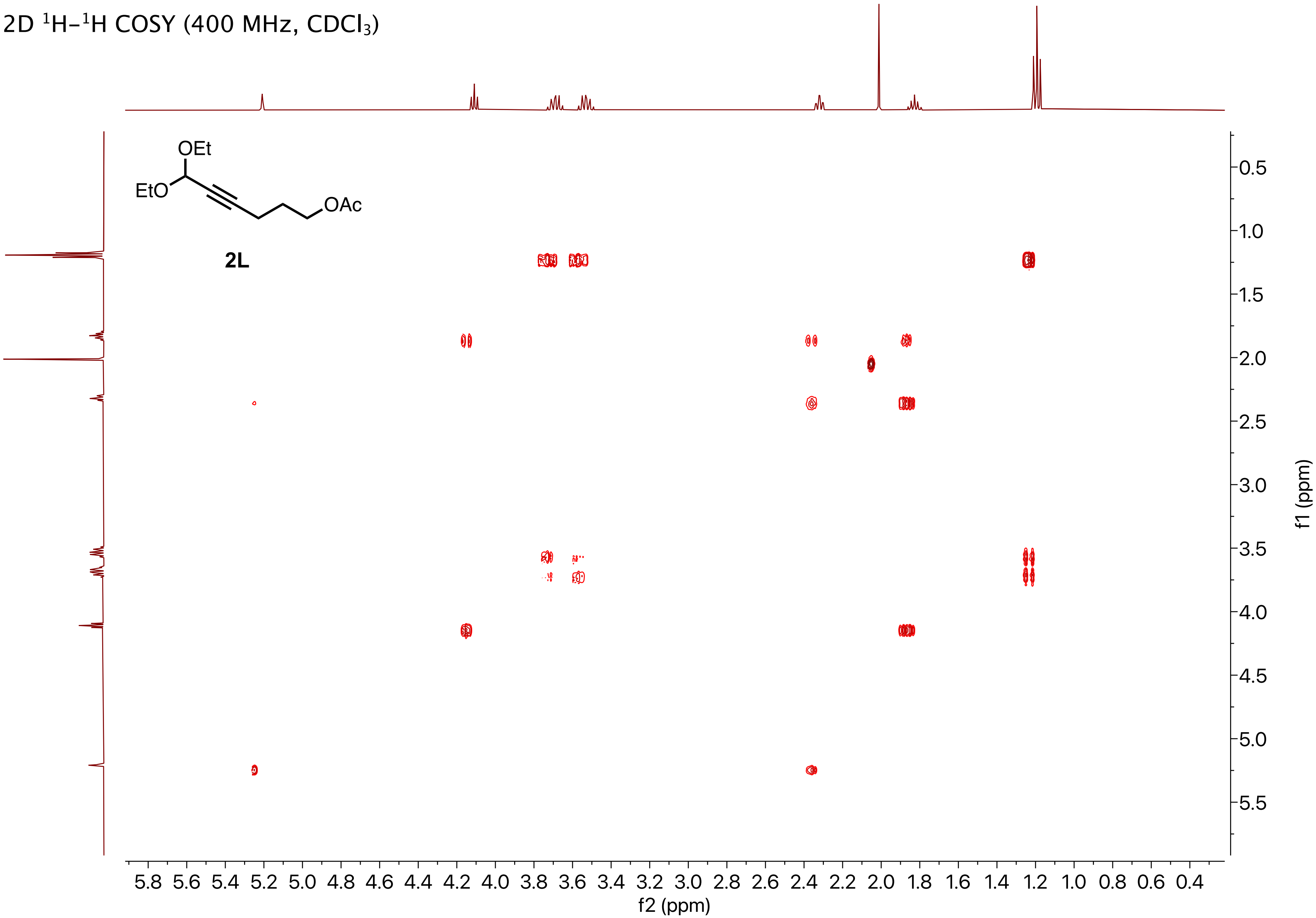

2D ^{1}H–^{13}C HSQC (400 MHz, $CDCl_3$)

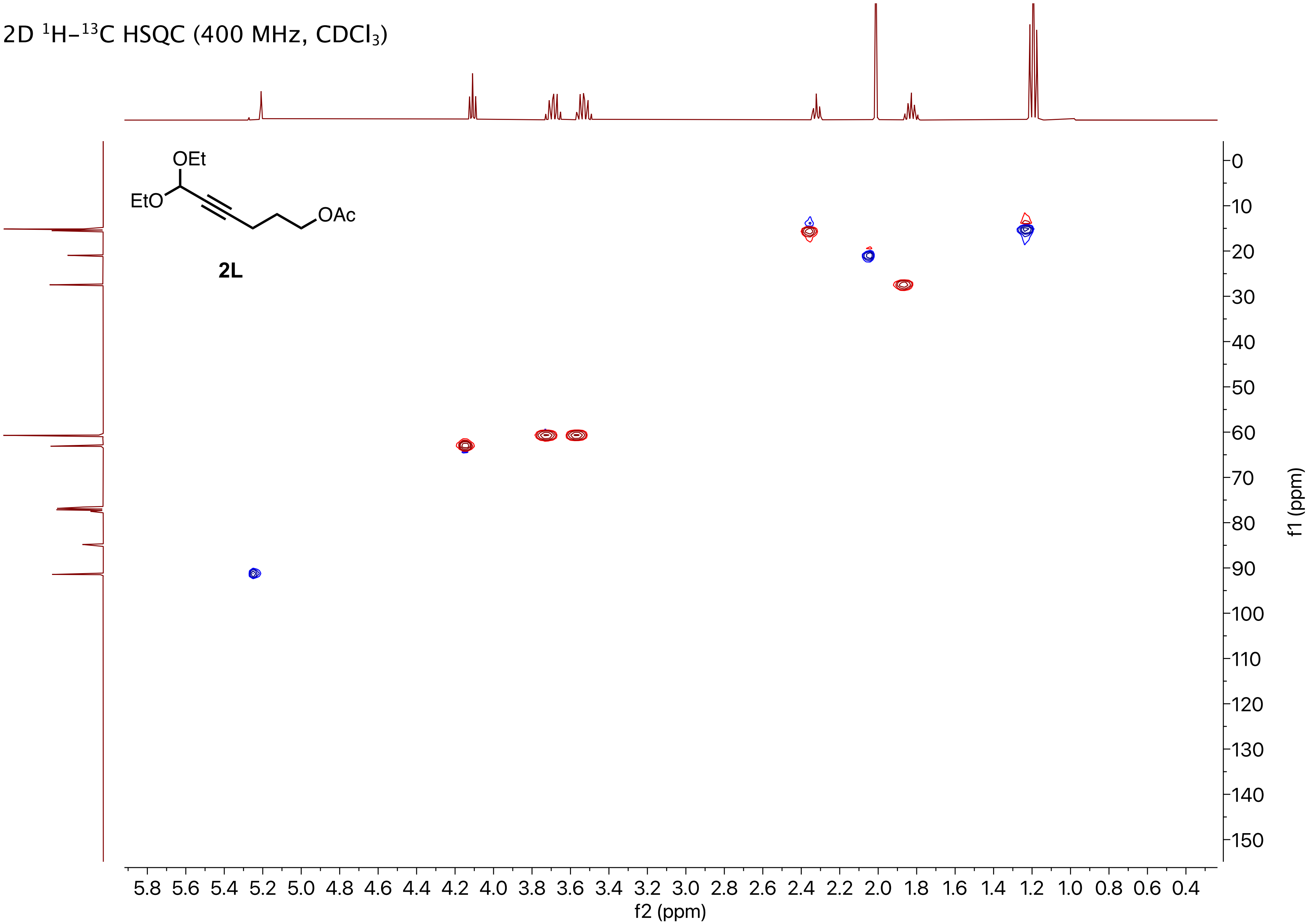

^{1}H NMR (400 MHz, $CDCl_3$)

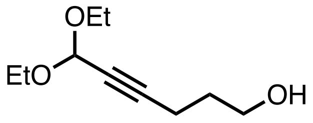

2M

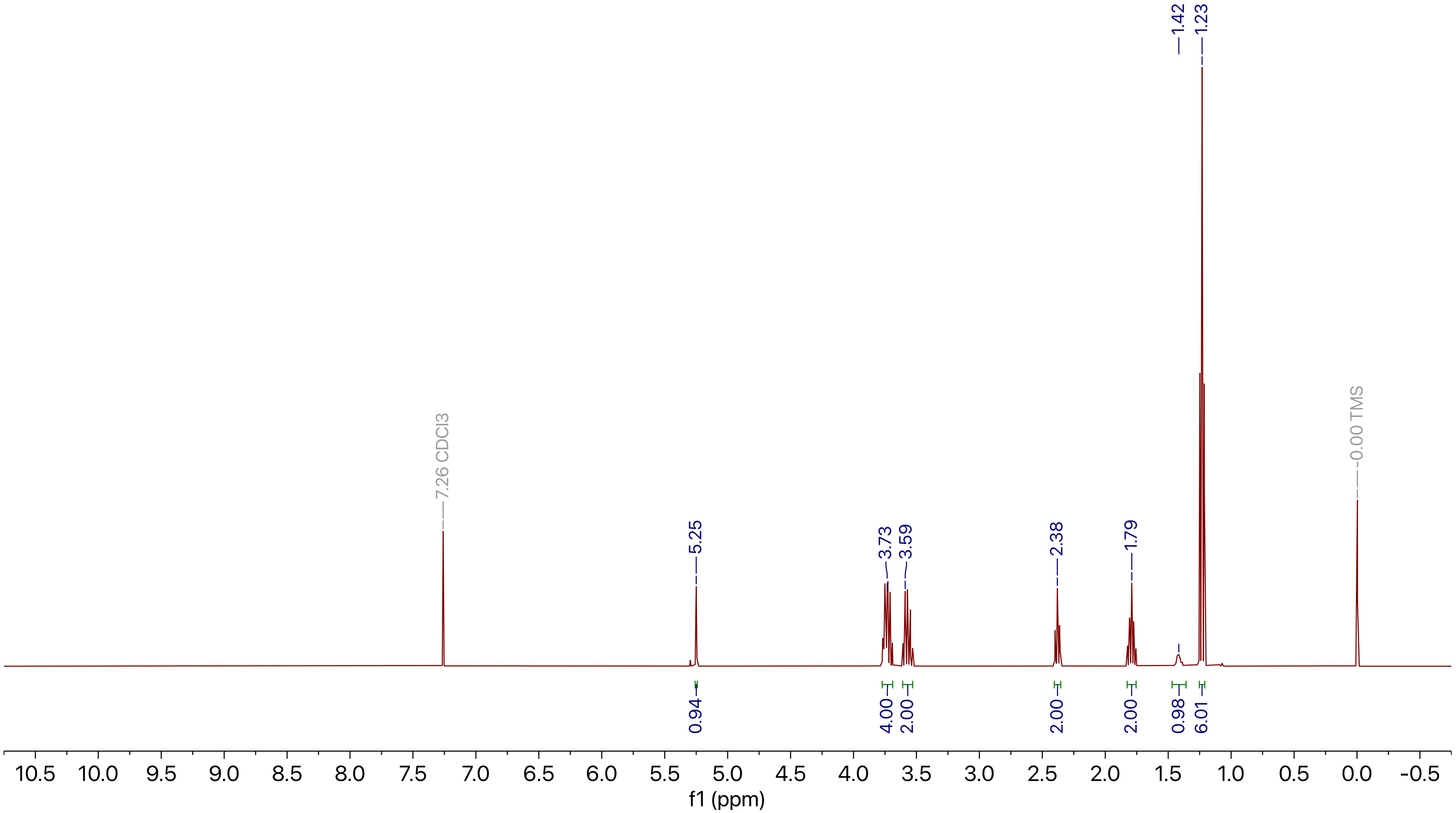

^{1}H NMR (400 MHz, $CDCl_3$)

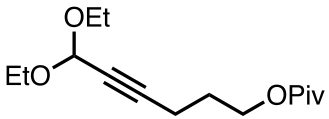

2J

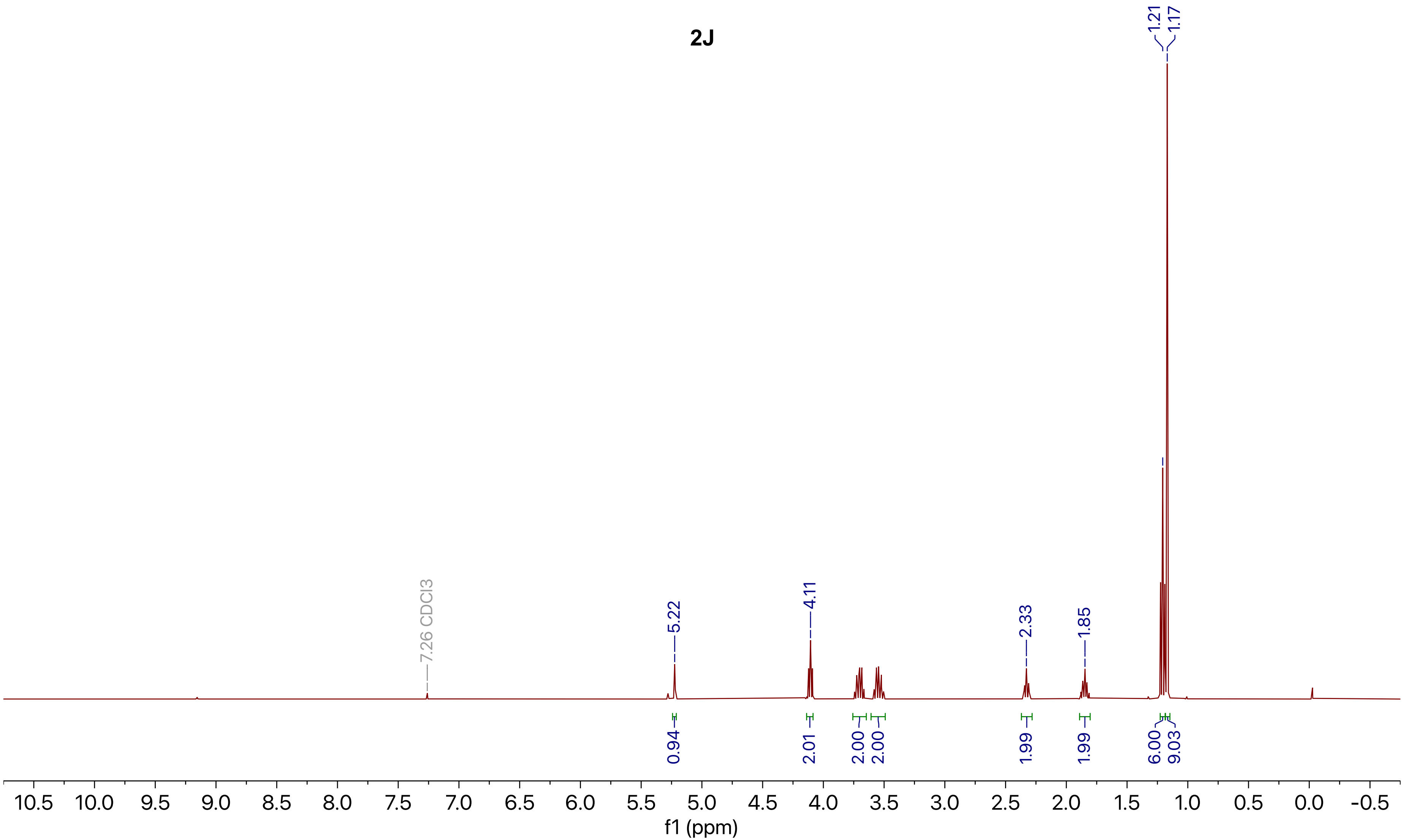

$^{13}C\{^{1}H\}$ NMR (101 MHz, $CDCl_3$)

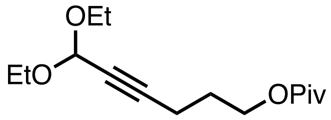

2J

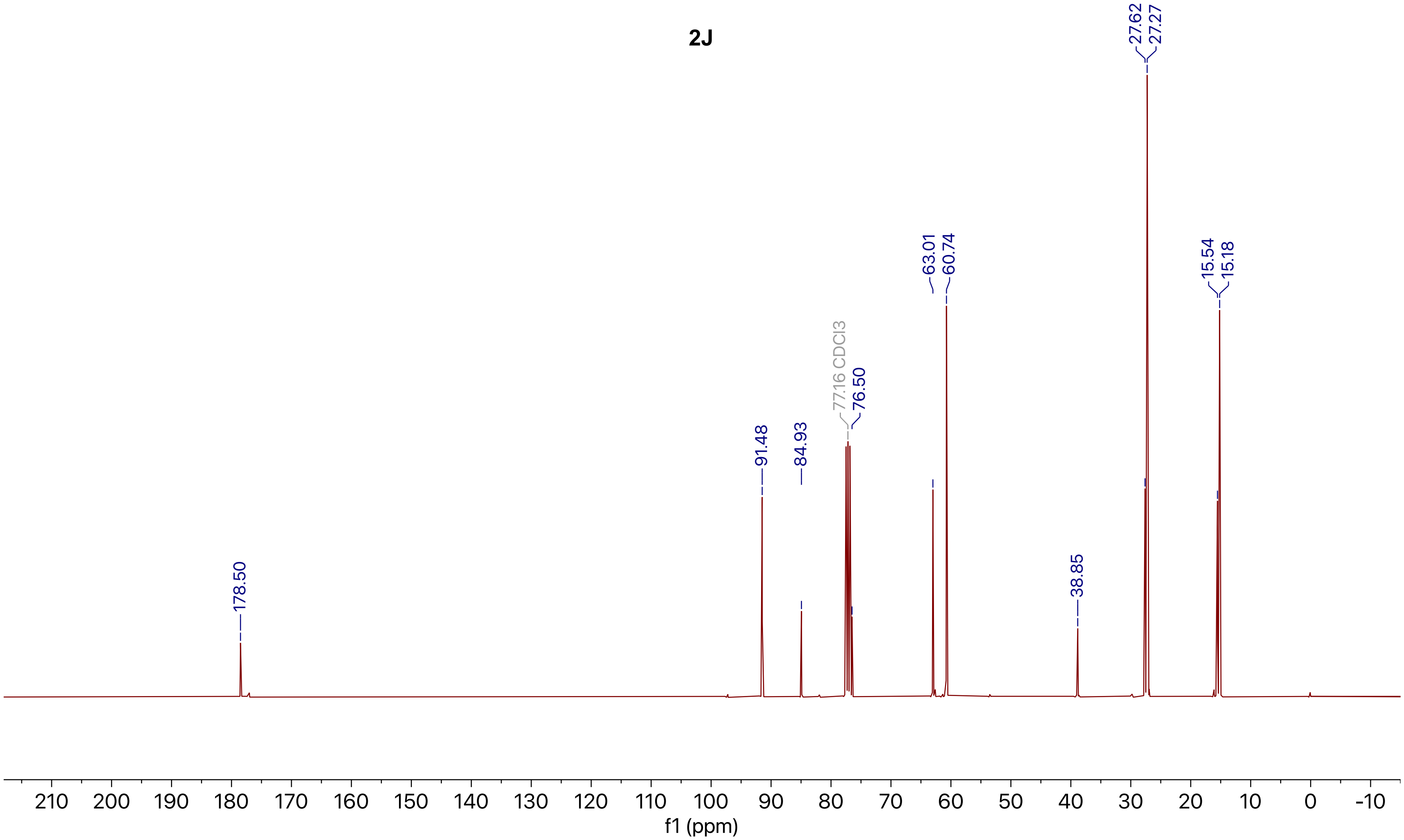

2D ^{1}H–^{1}H COSY (400 MHz, $CDCl_3$)

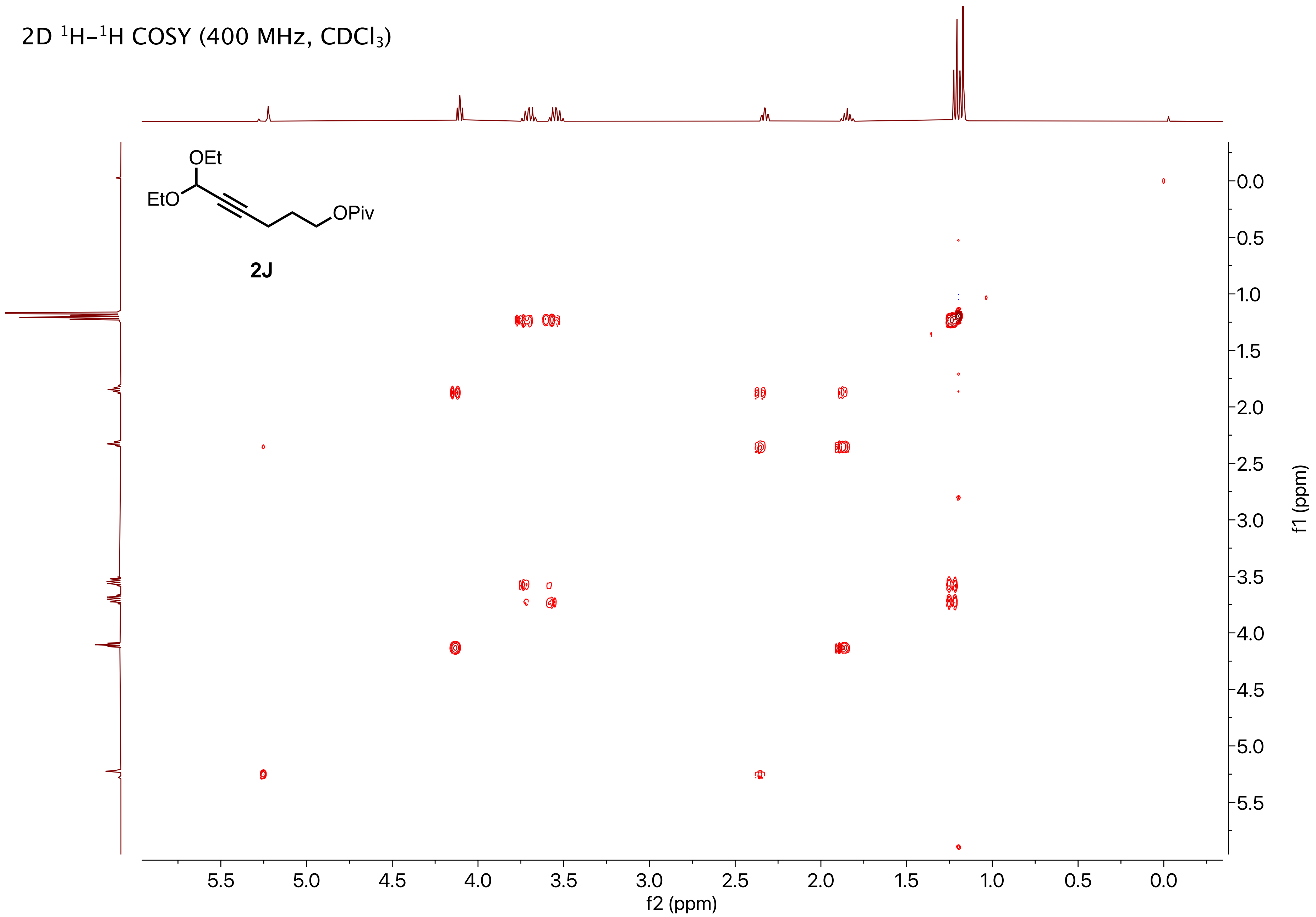

2D ^{1}H–^{13}C HSQC (400 MHz, $CDCl_3$)

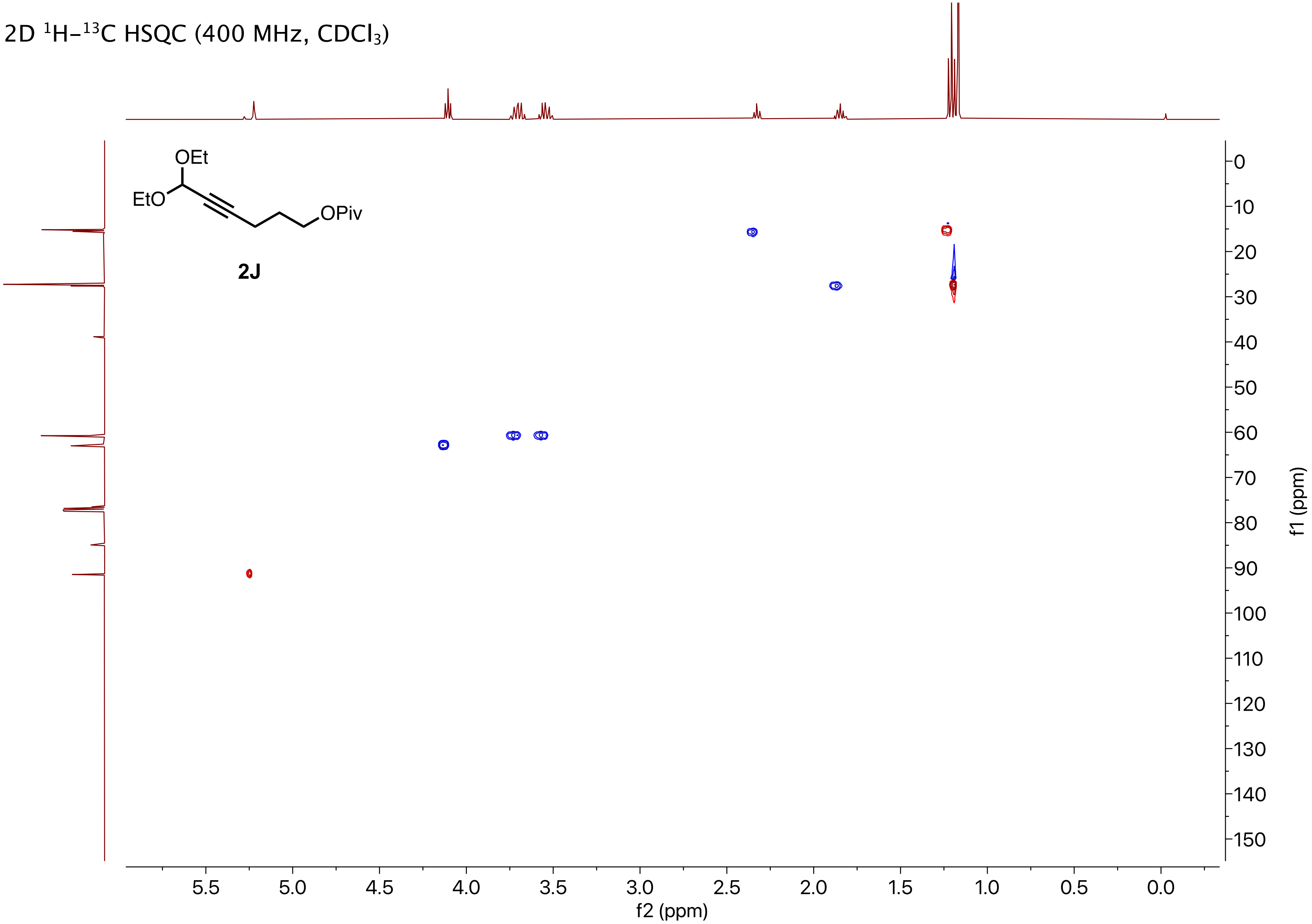

^{1}H NMR (400 MHz, $CDCl_3$)

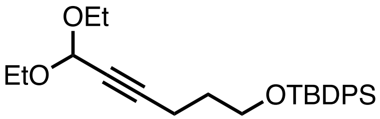

2K

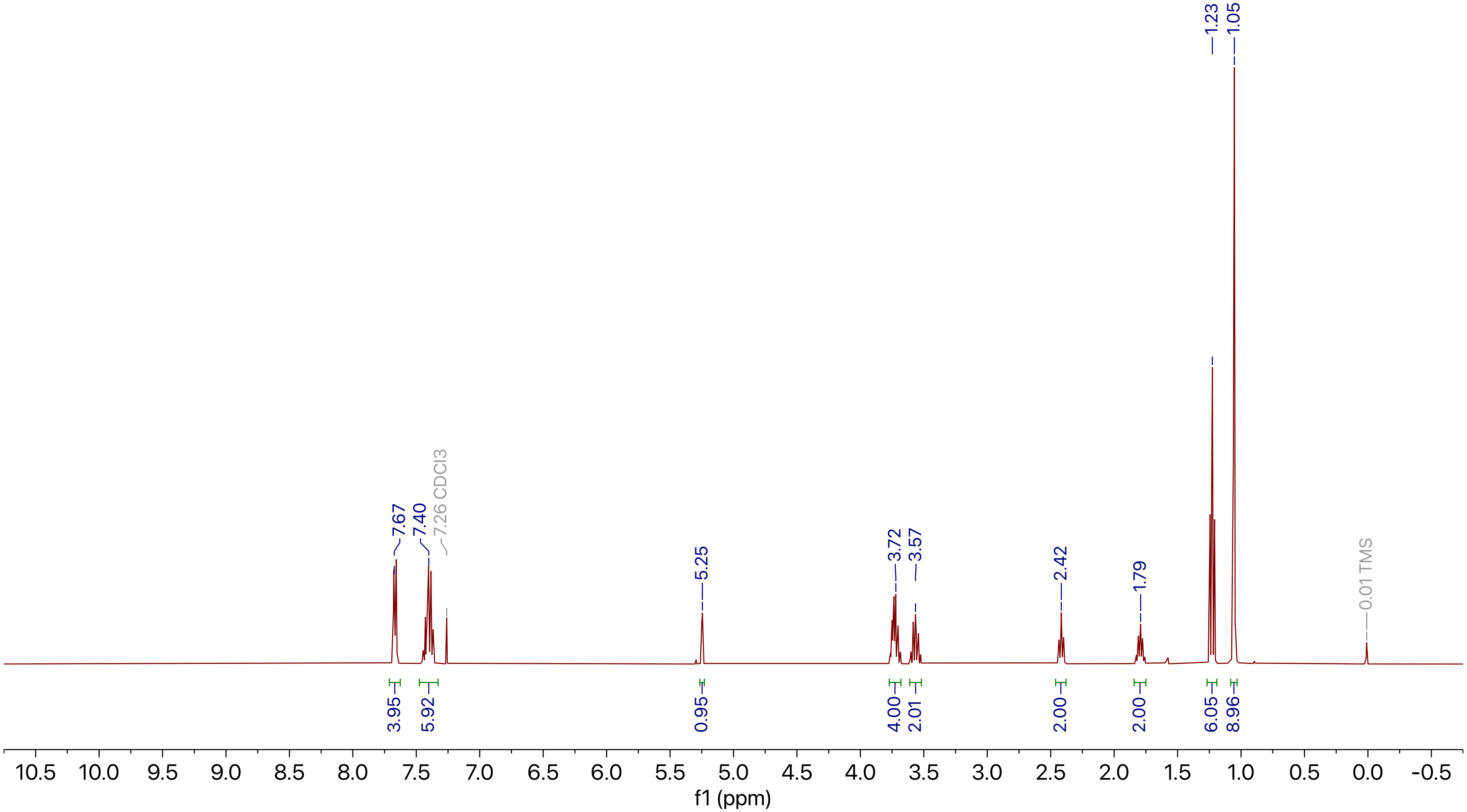

$^{13}C\{^{1}H\}$ NMR (101 MHz, $CDCl_3$)

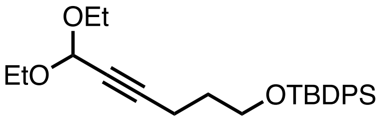

2K

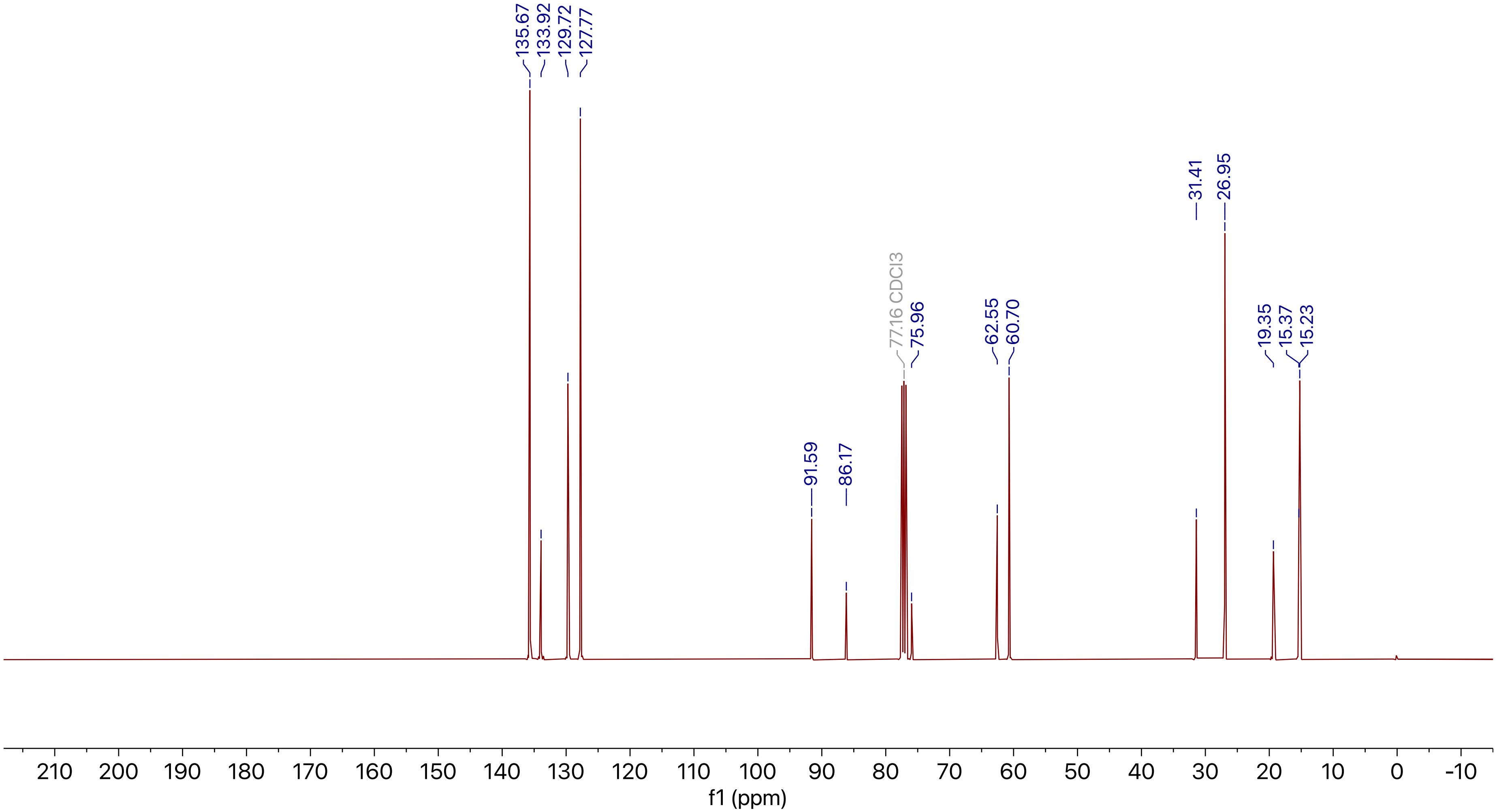

2D ^{1}H–^{1}H COSY (400 MHz, $CDCl_3$)

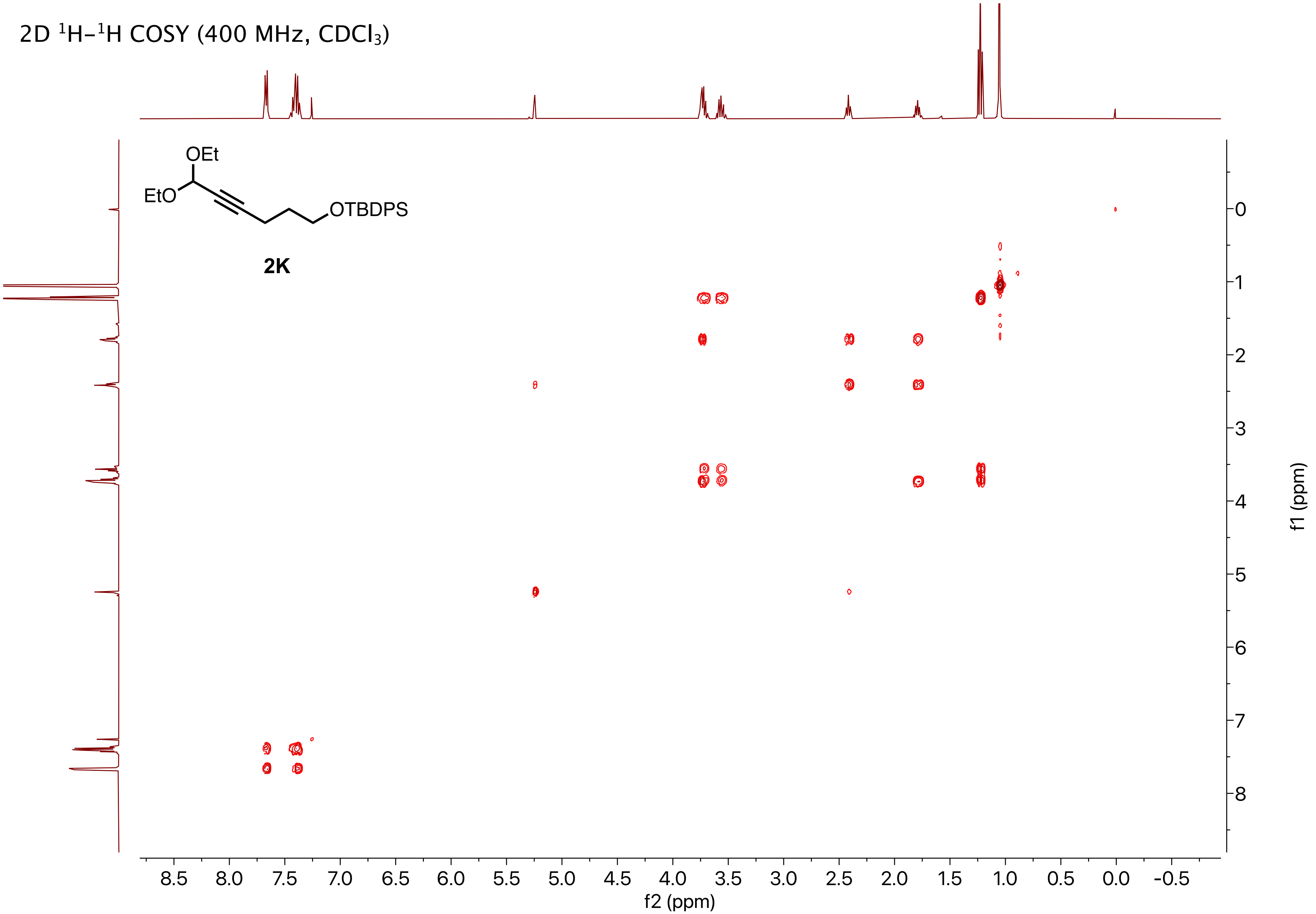

2D ^{1}H–^{13}C HSQC (400 MHz, $CDCl_3$)

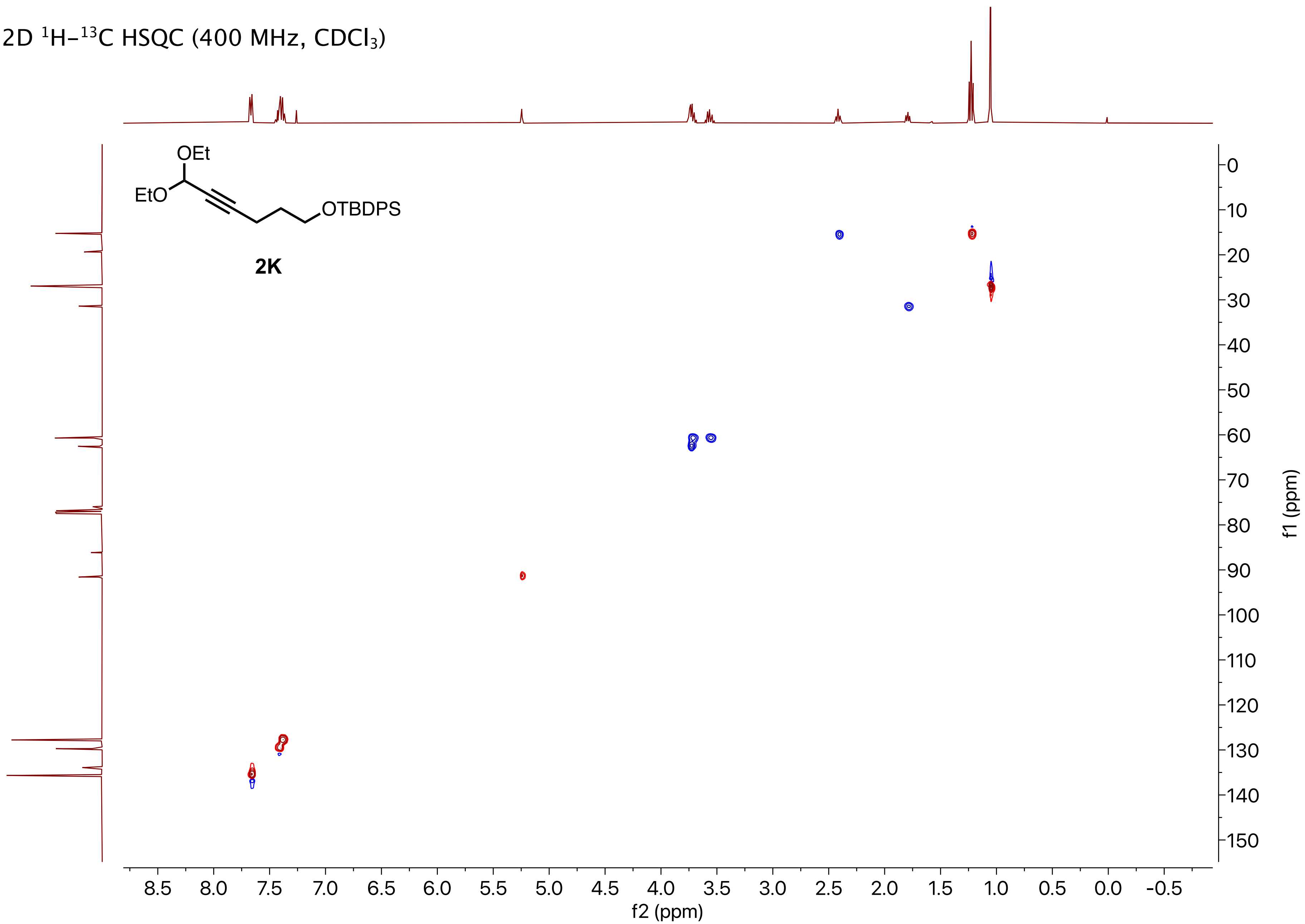

^{1}H NMR (400 MHz, $CDCl_3$)

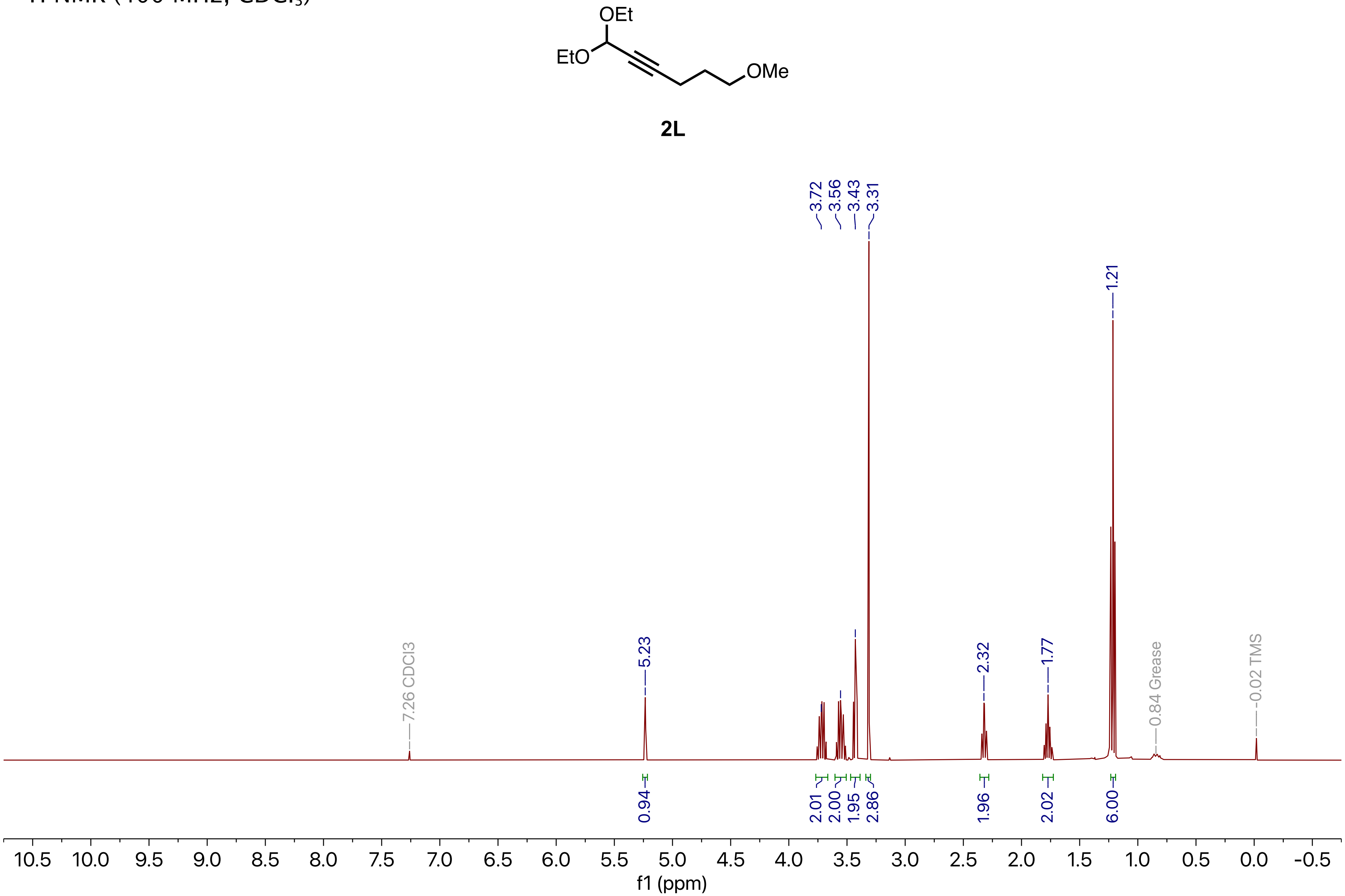

$^{13}C\{^{1}H\}$ NMR (101 MHz, $CDCl_3$)

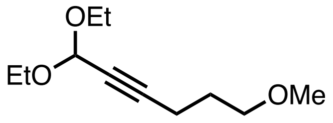

2L

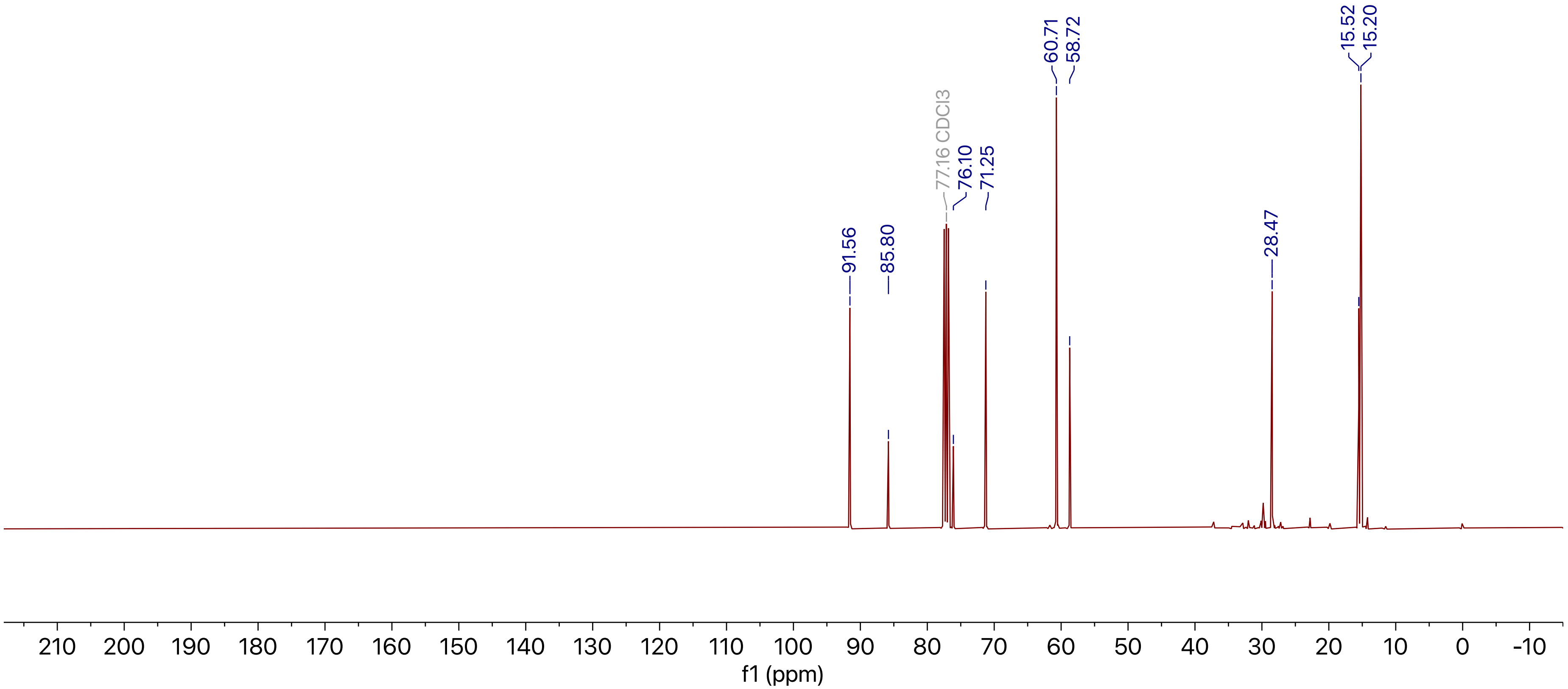

2D ^{1}H–^{1}H COSY (400 MHz, $CDCl_3$)

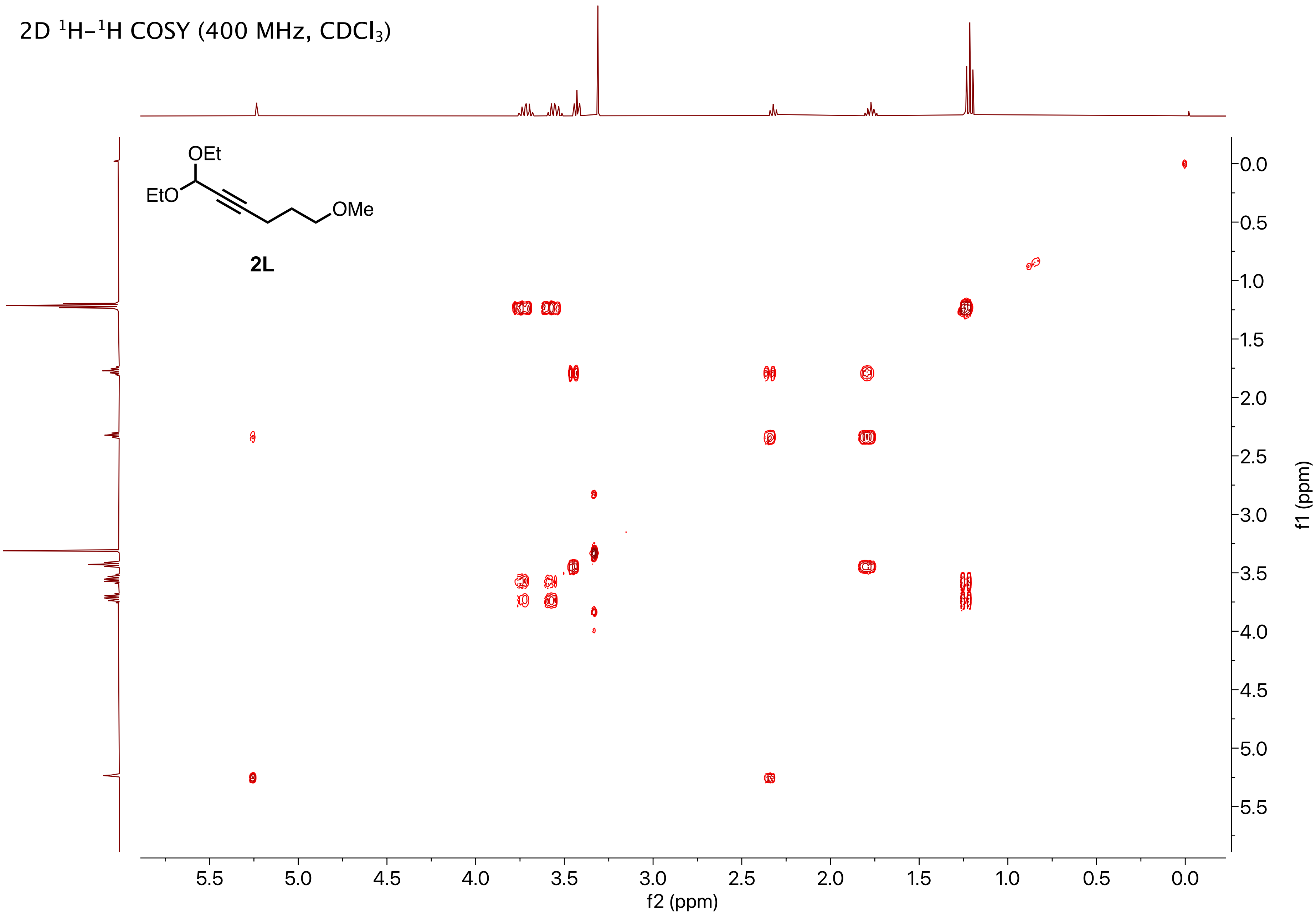

2D ^{1}H–^{13}C HSQC (400 MHz, $CDCl_3$)

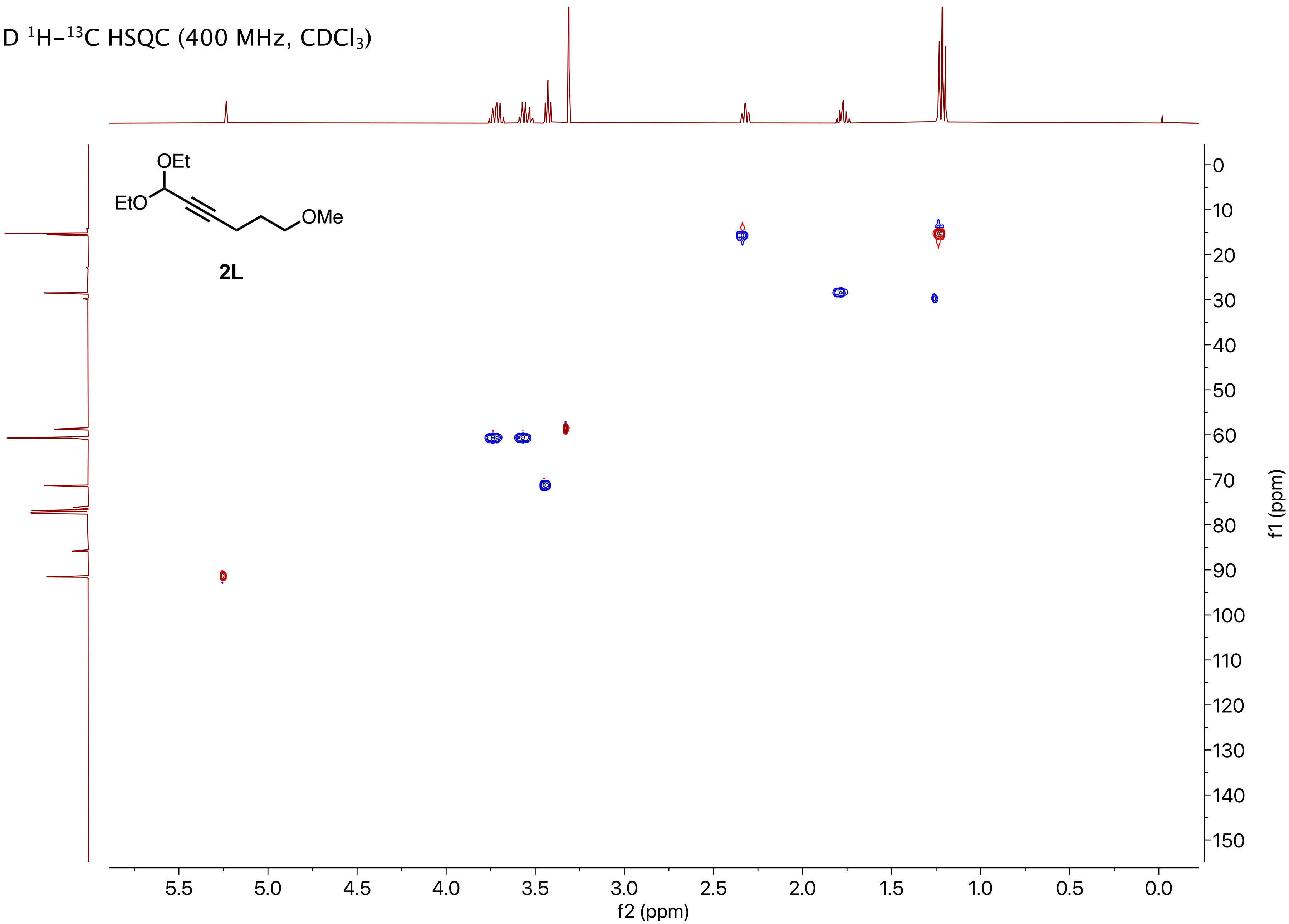

^{1}H NMR (400 MHz, $CDCl_3$)

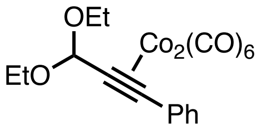

2a

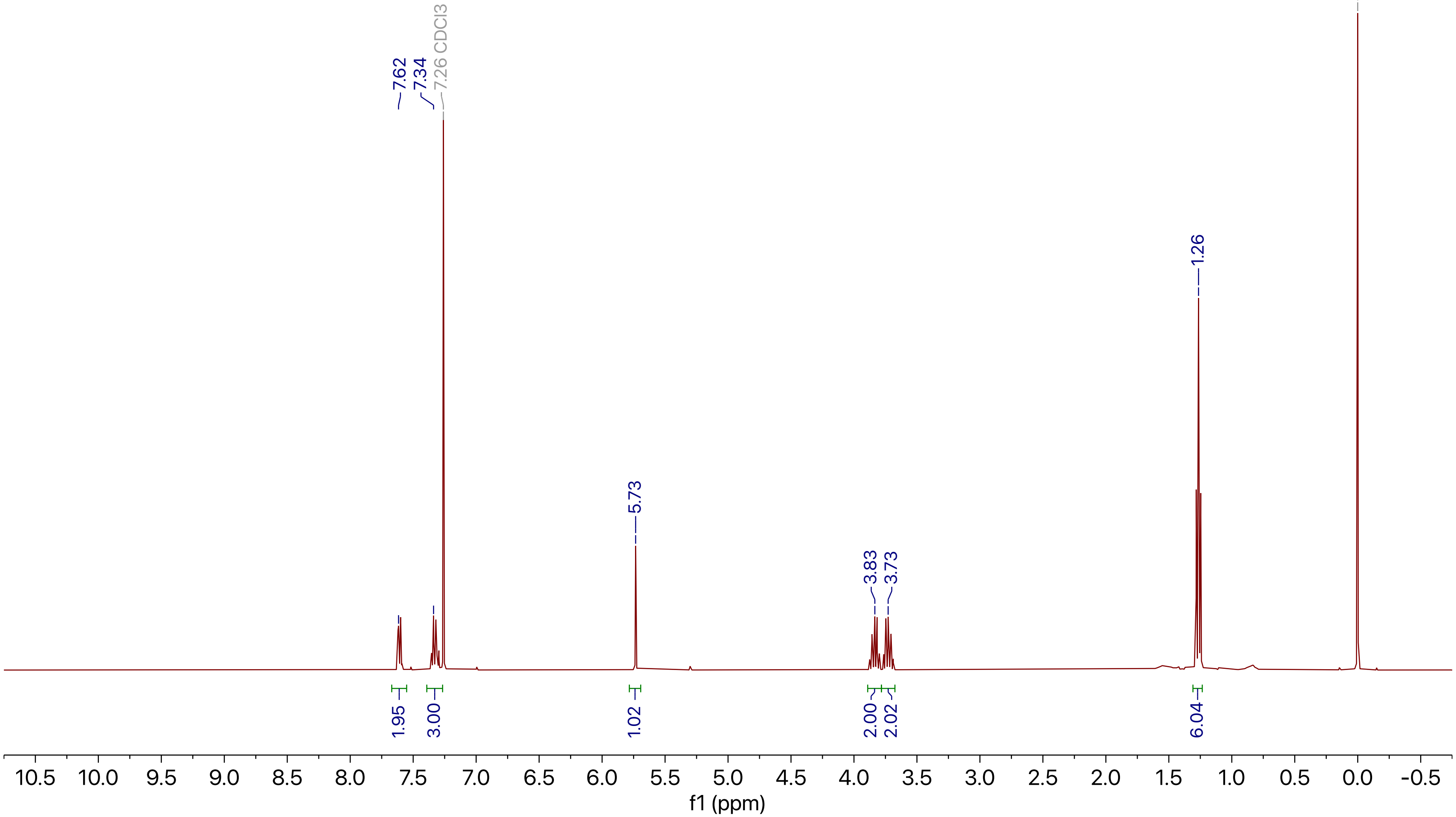

^{1}H NMR (400 MHz, $CDCl_3$)

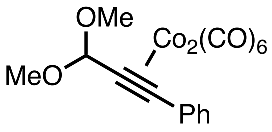

2b

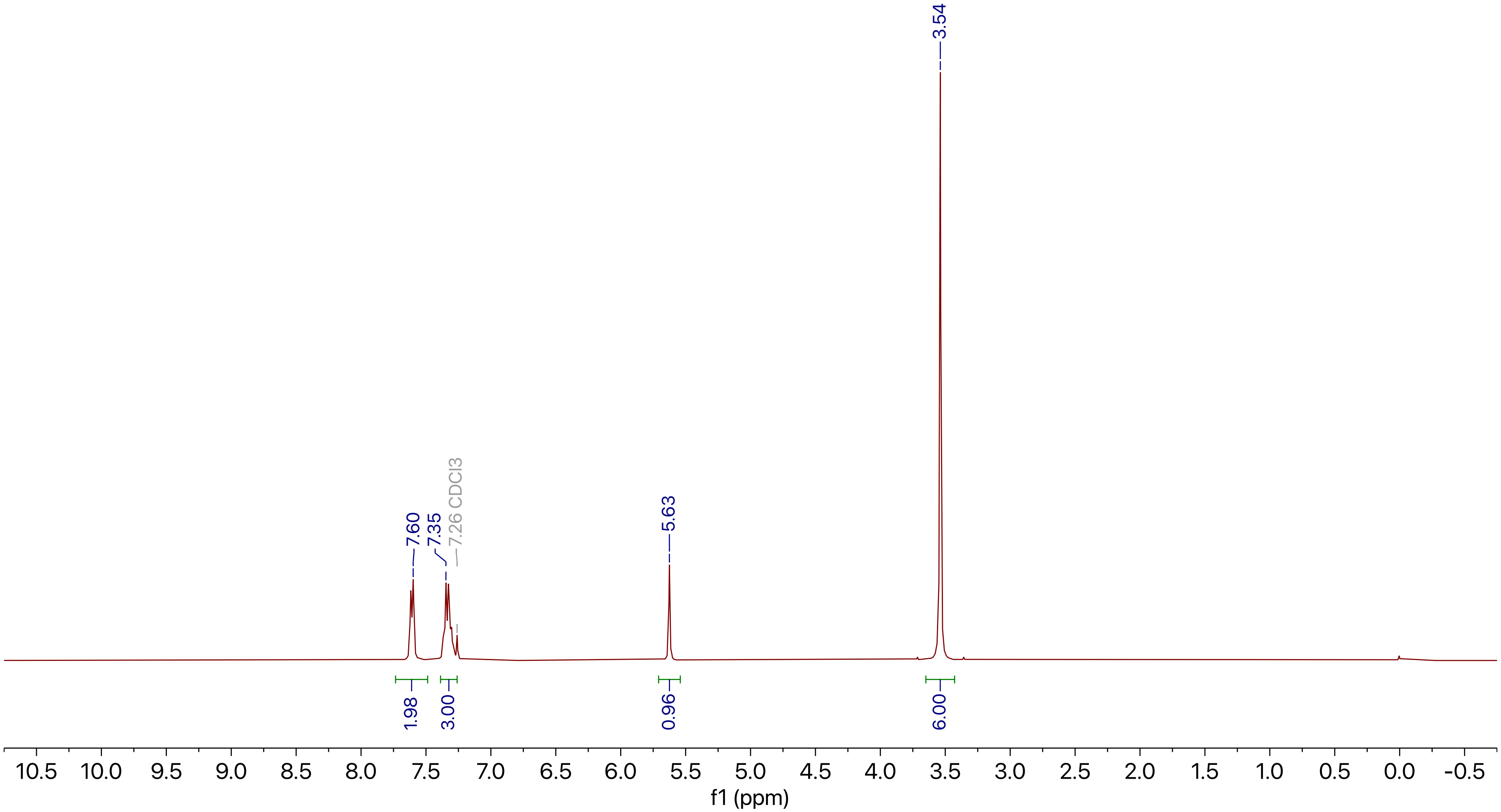

^{1}H NMR (400 MHz, $CDCl_3$)

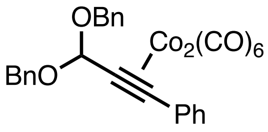

2c

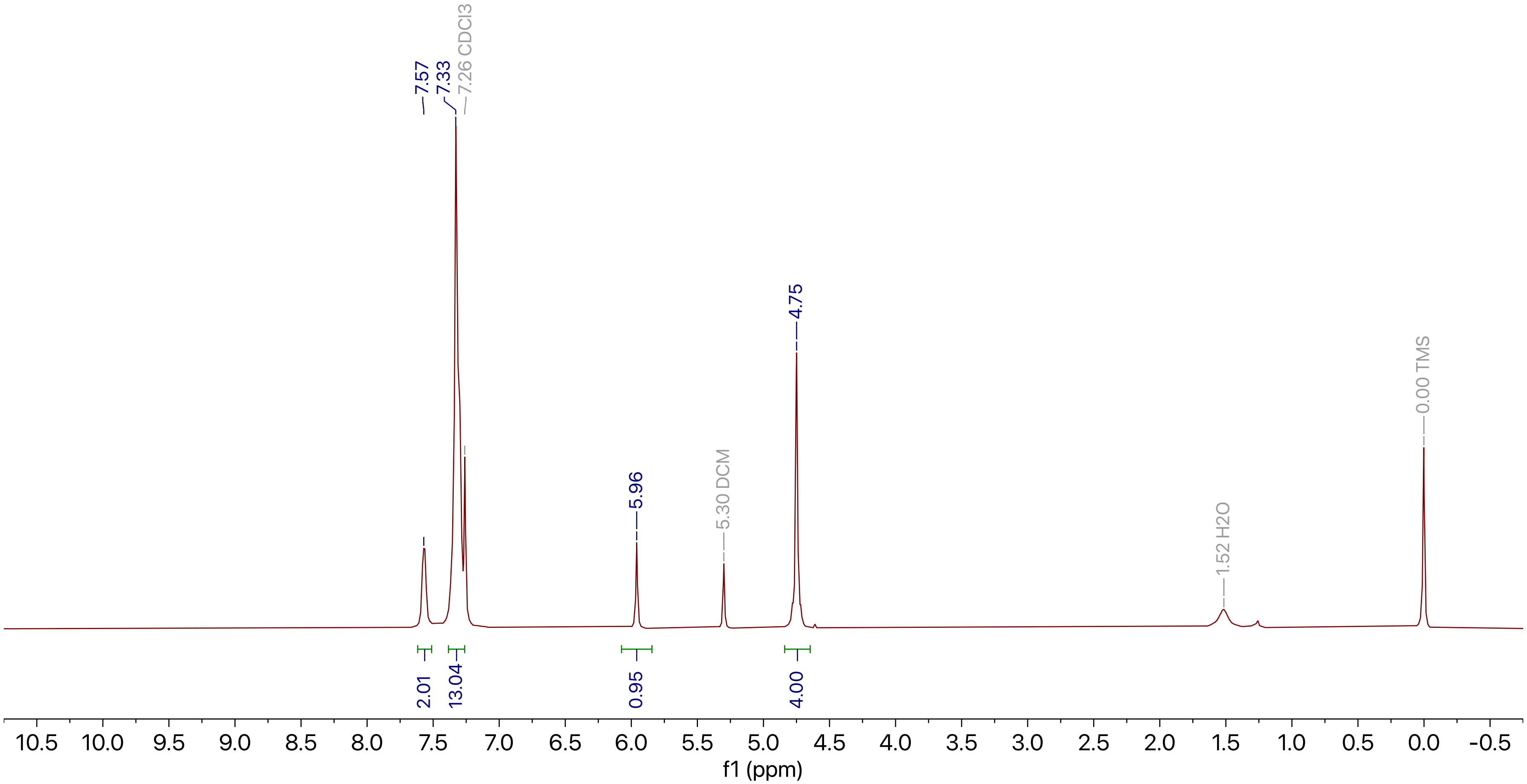

^{1}H NMR (500 MHz, $CDCl_3$)

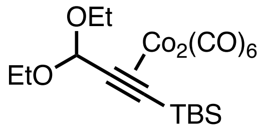

2d

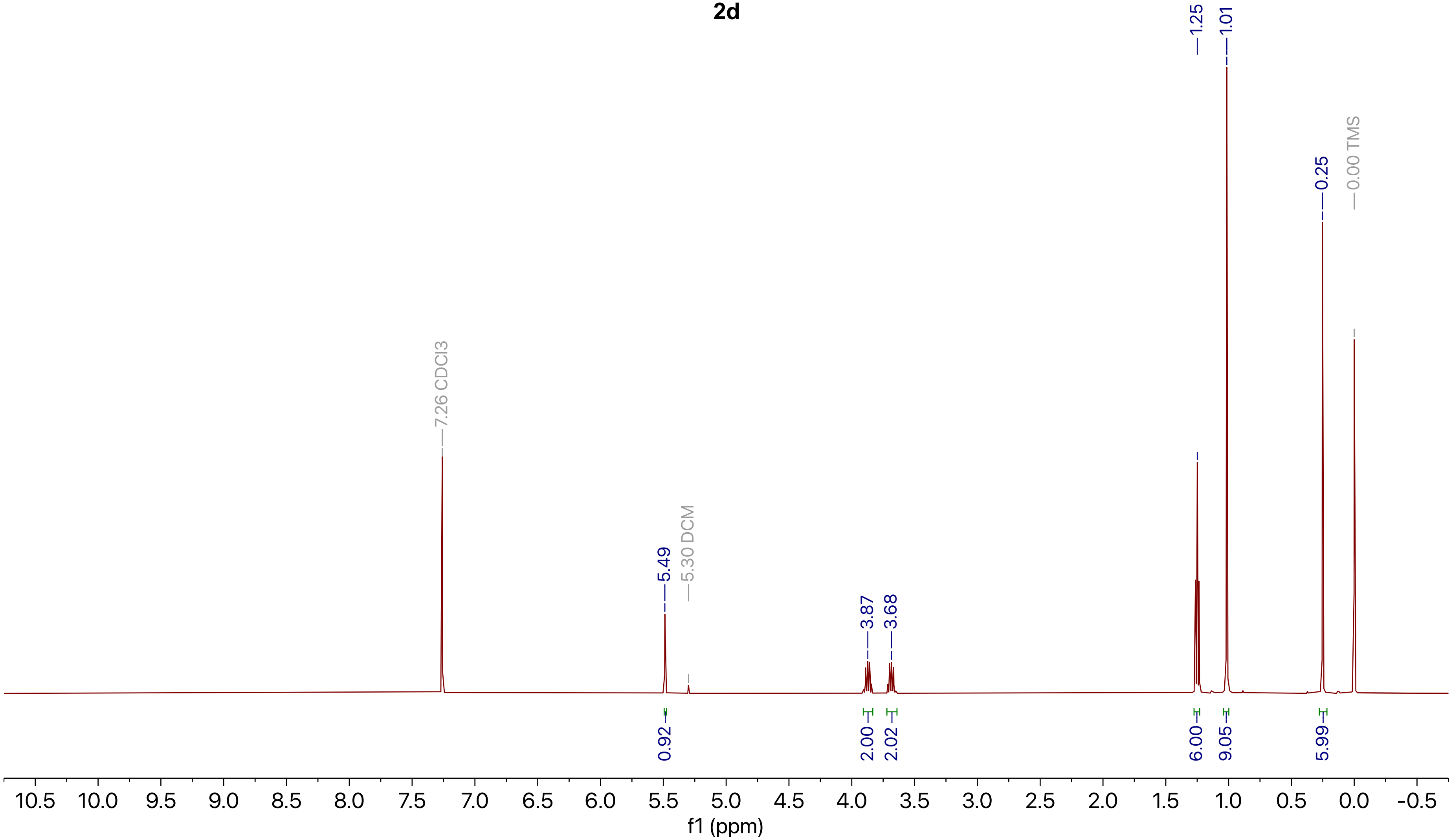

^1H NMR (500 MHz, $CDCl_3$)

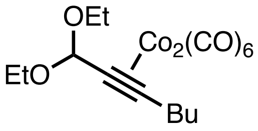

2e

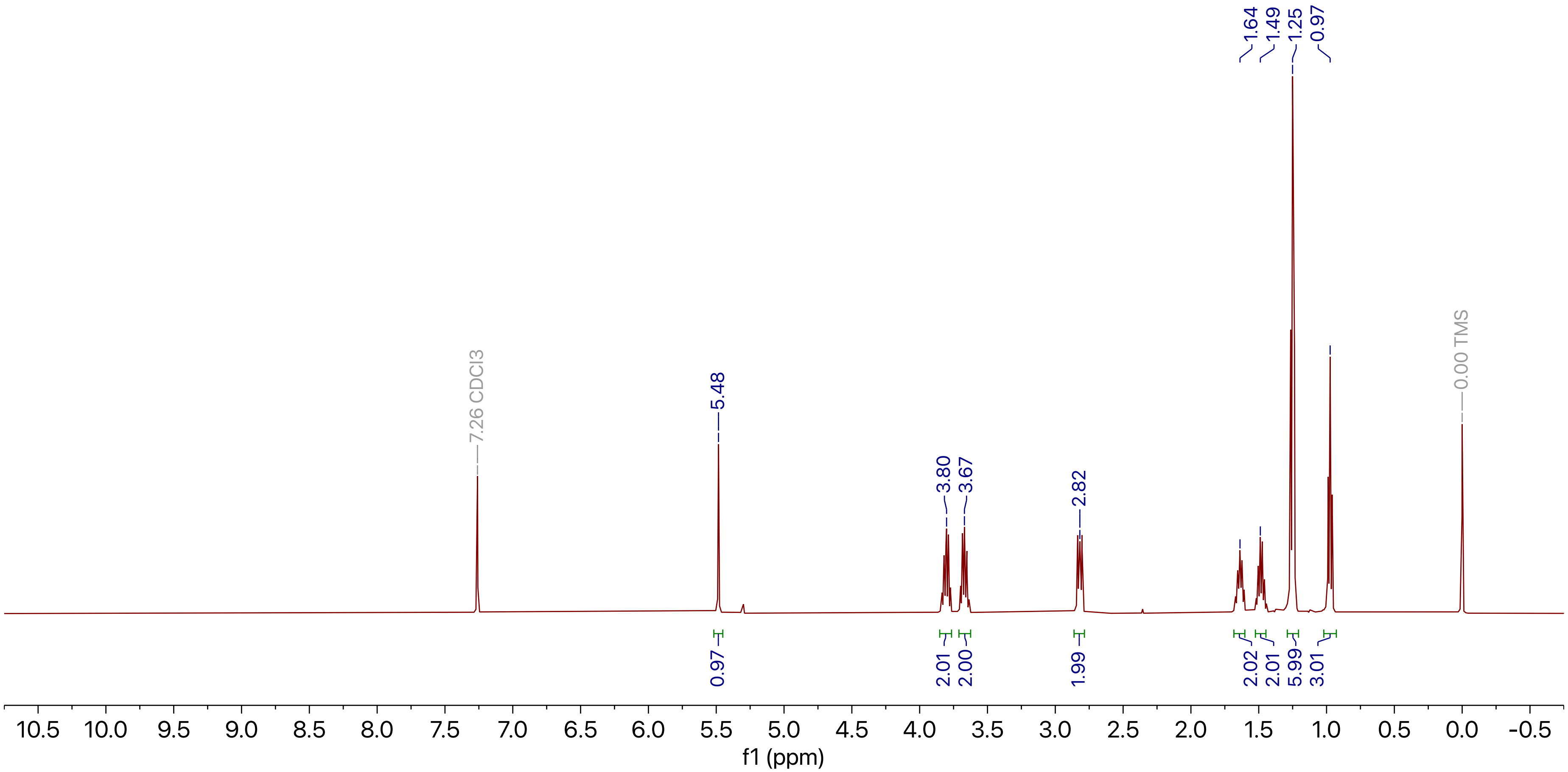

^{1}H NMR (500 MHz, $CDCl_3$)

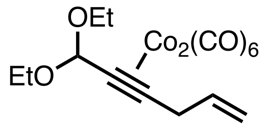

2f

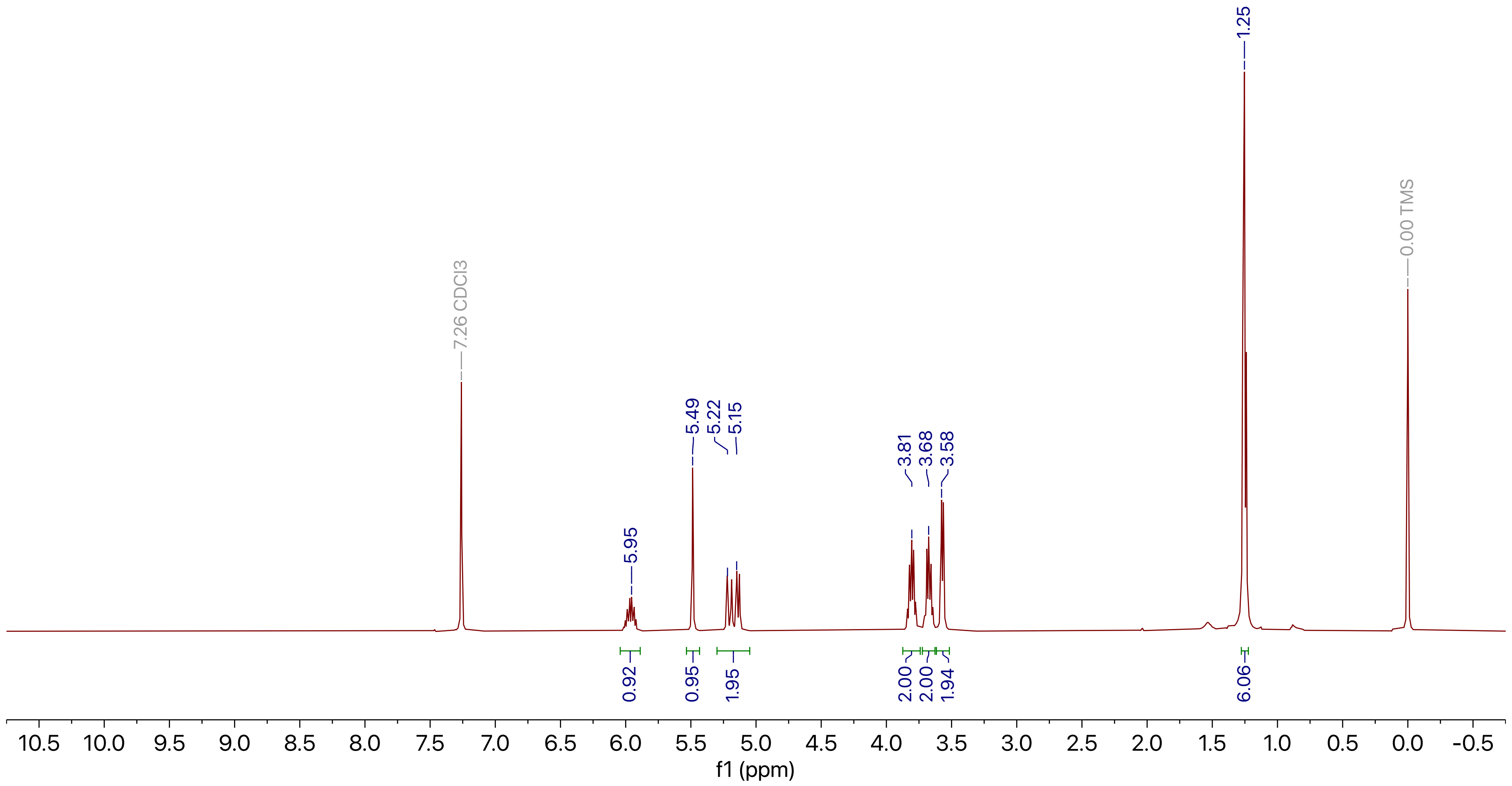

^1H NMR (400 MHz, $CDCl_3$)

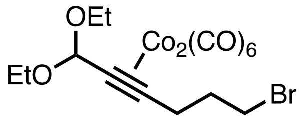

2g

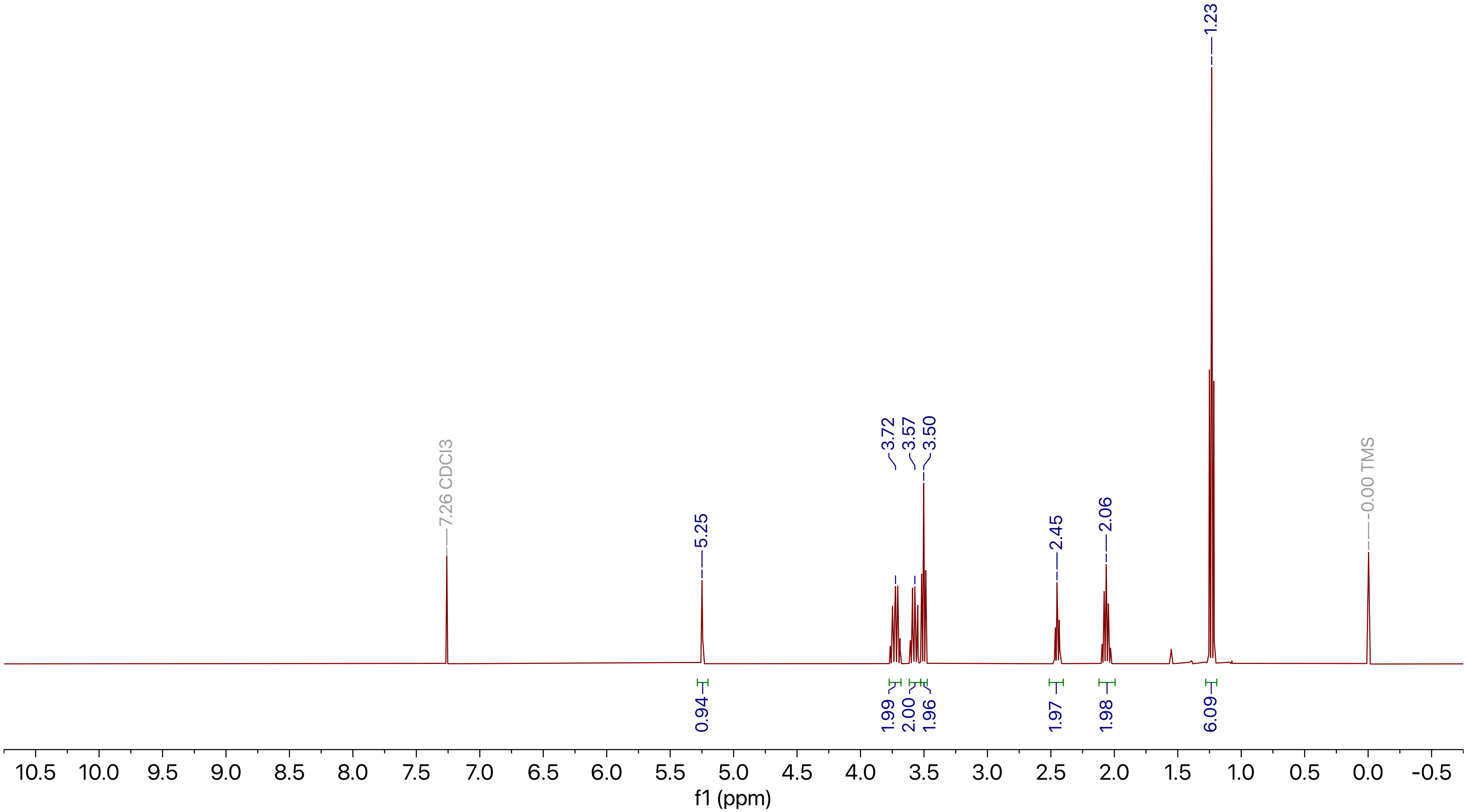

^{1}H NMR (500 MHz, $CDCl_3$)

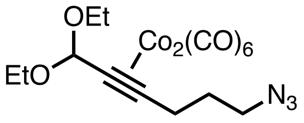

2h

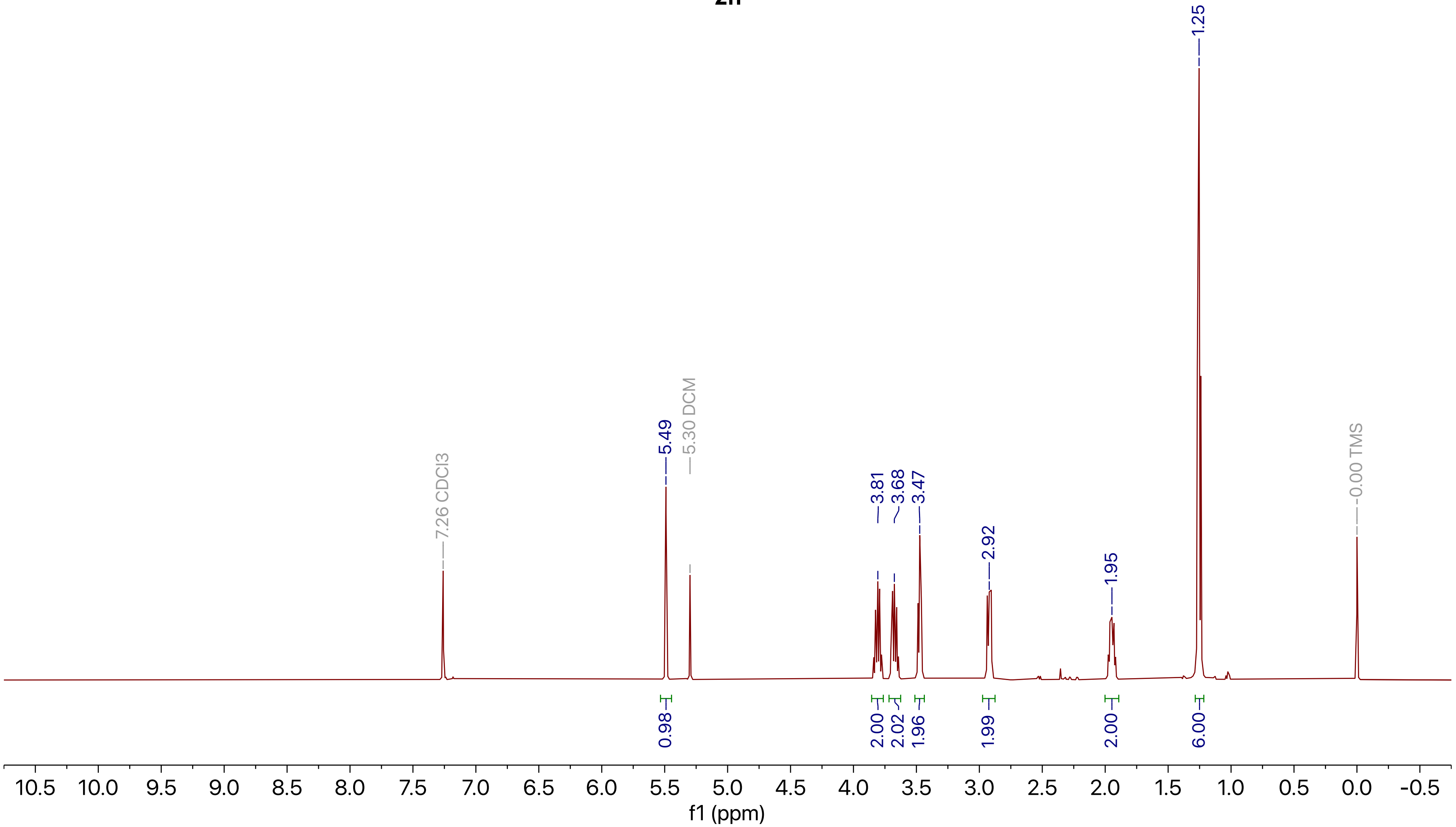

^{1}H NMR (400 MHz, $CDCl_3$)

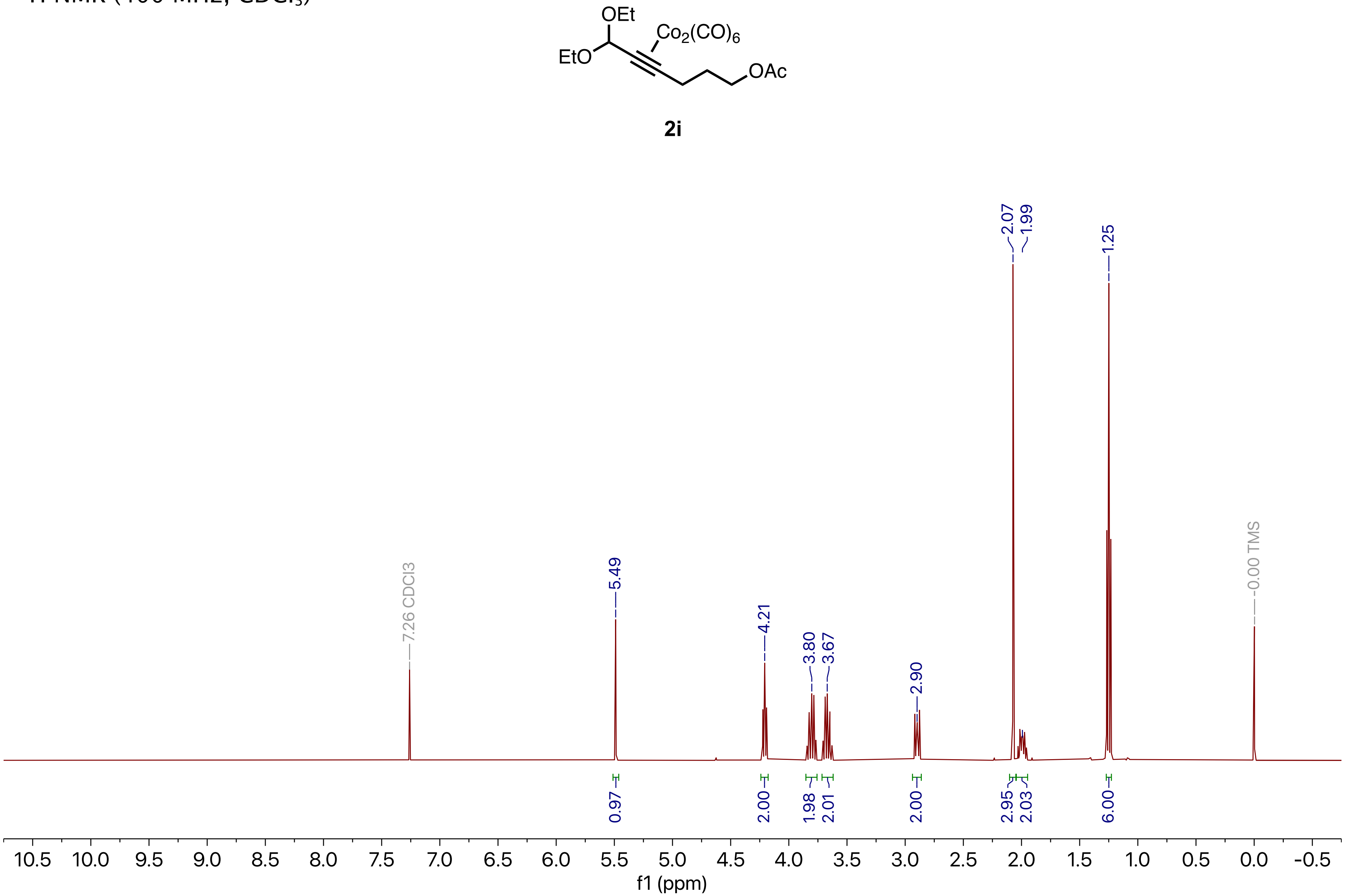

^{1}H NMR (400 MHz, $CDCl_3$)

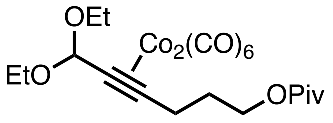

2j

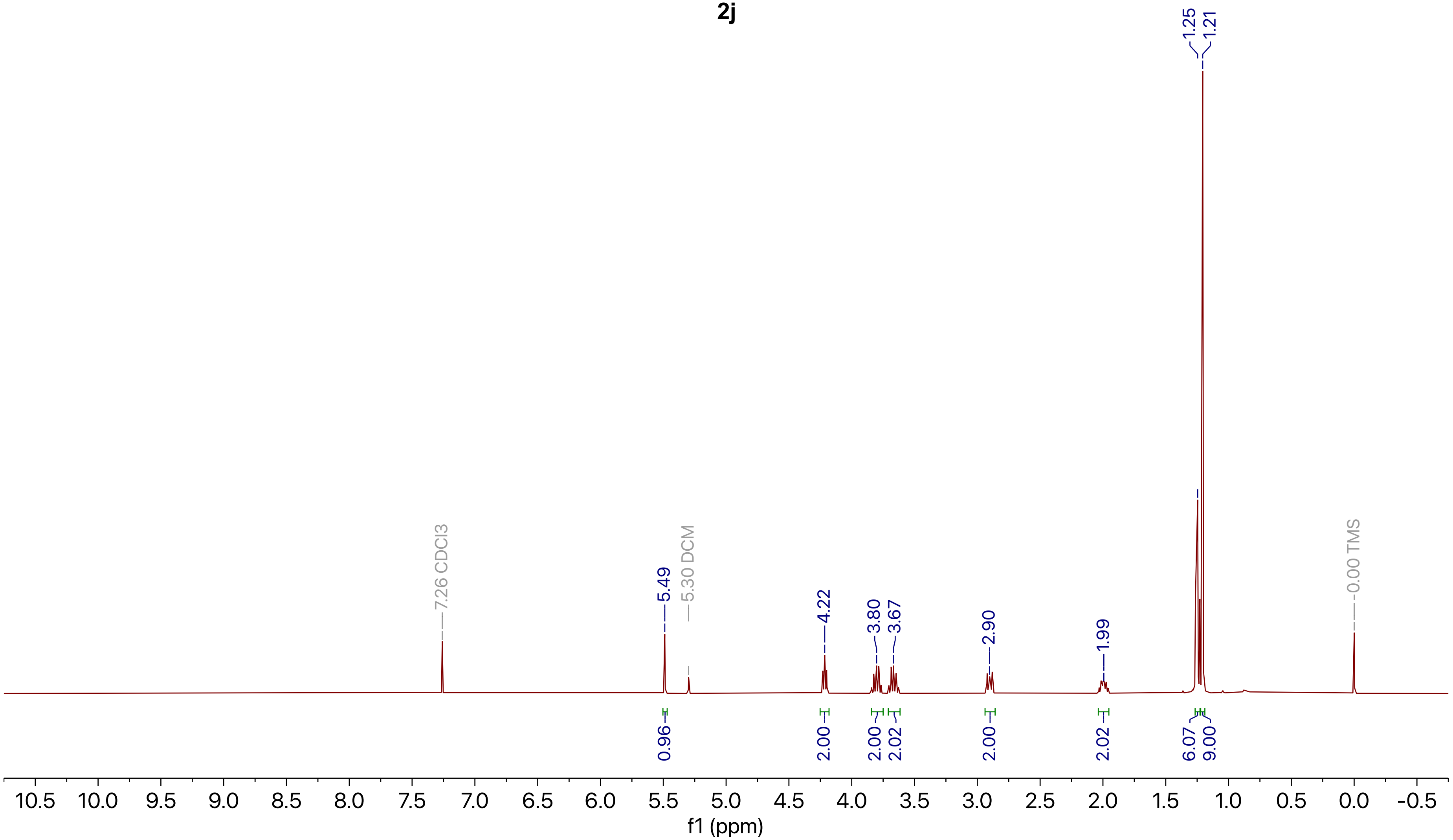

^{1}H NMR (400 MHz, $CDCl_3$)

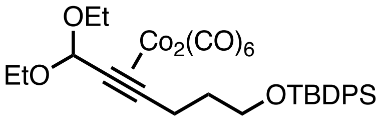

2k

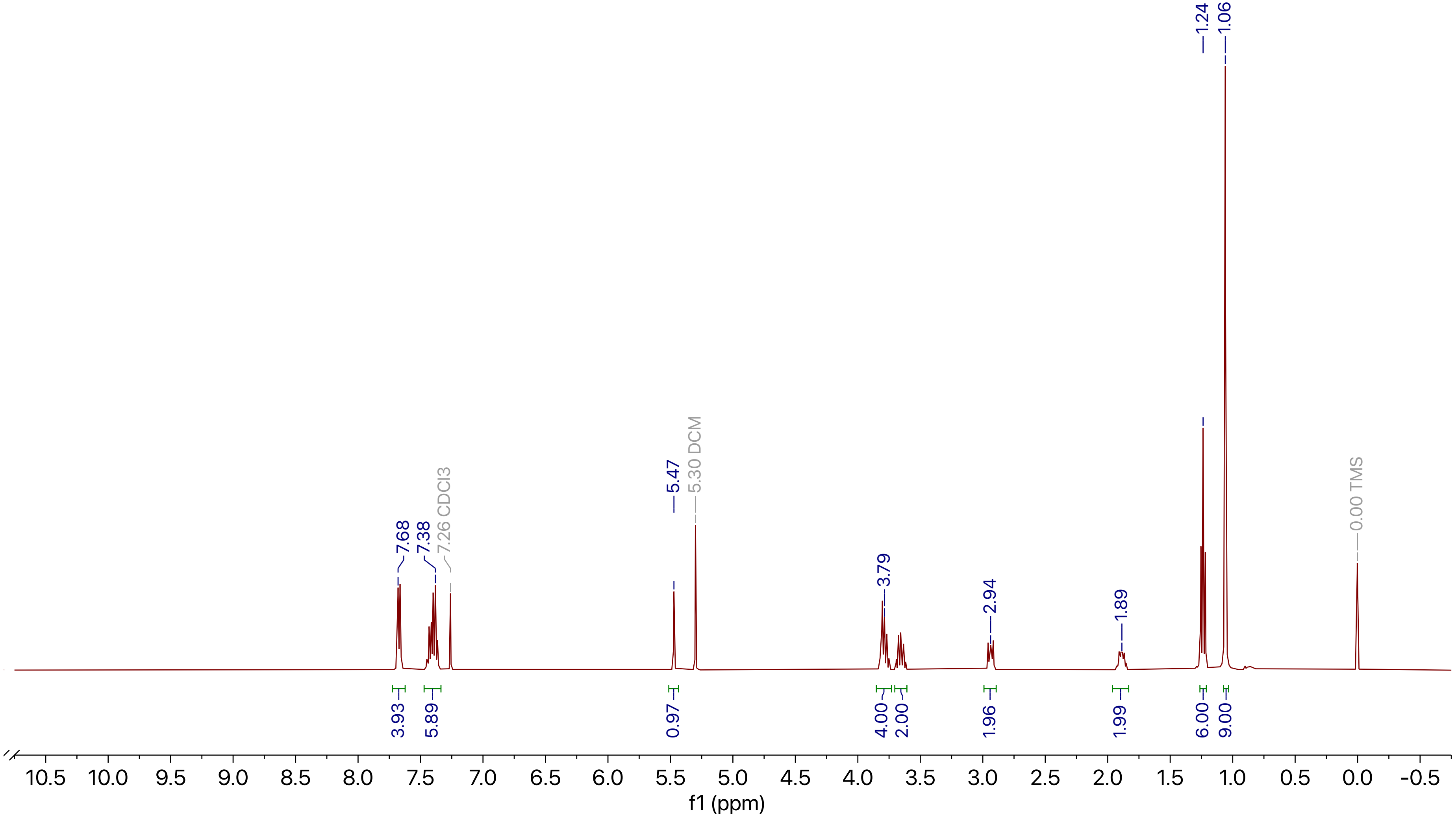

^{1}H NMR (400 MHz, $CDCl_3$)

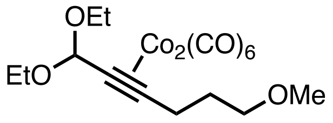

2l

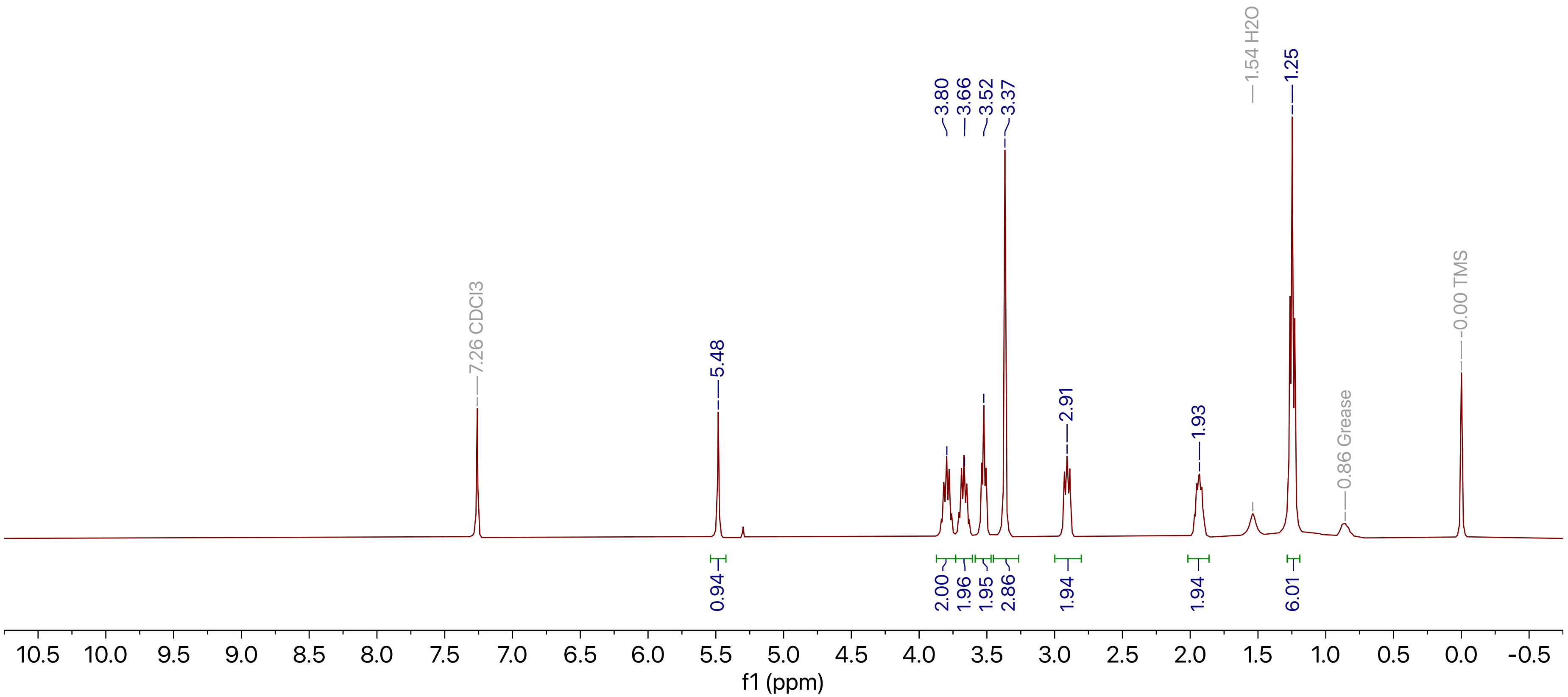

^{1}H NMR (500 MHz, $CDCl_3$)

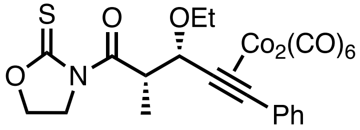

3a

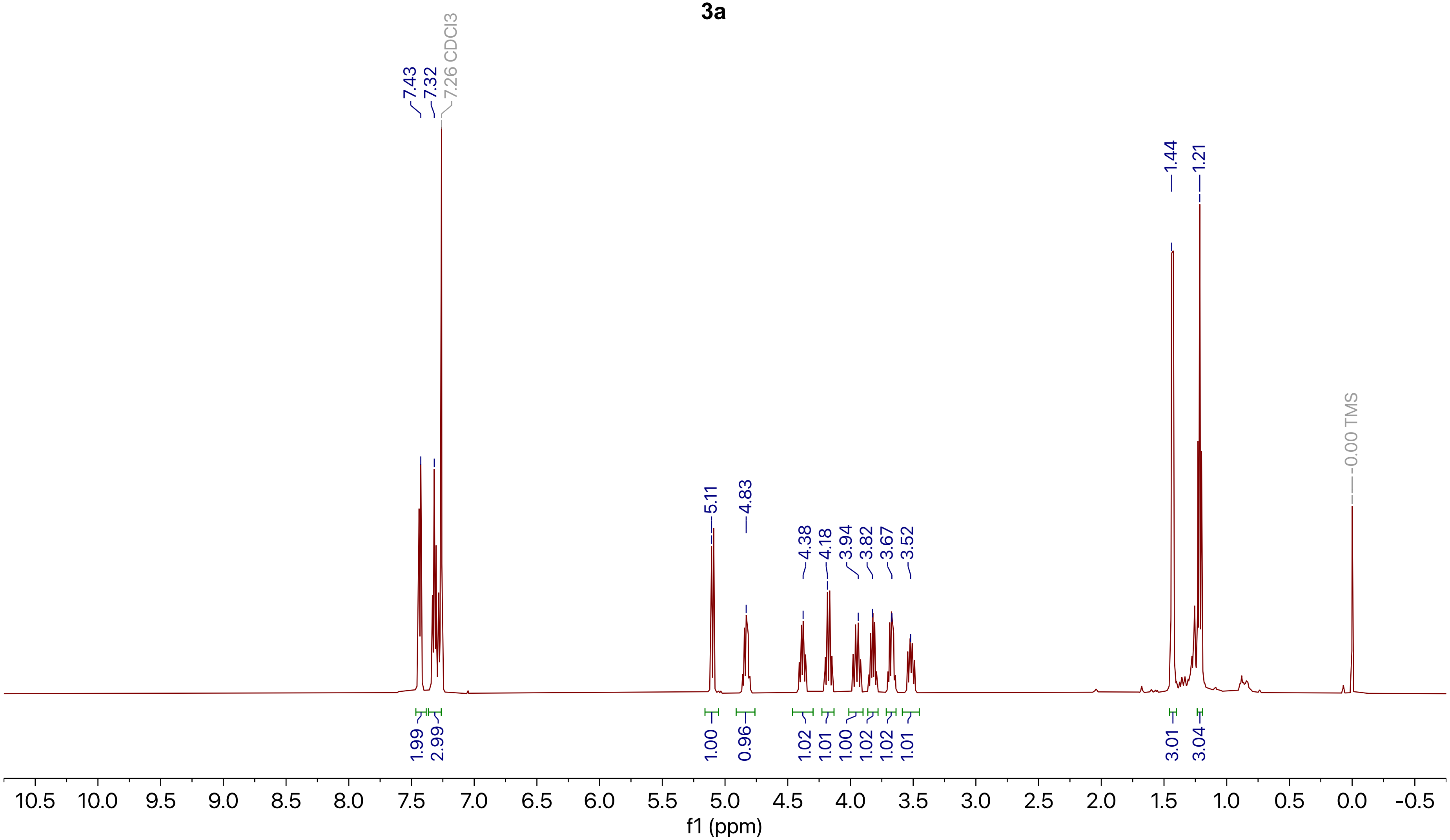

$^{13}C\{^1H\}$ NMR (126 MHz, $CDCl_3$)

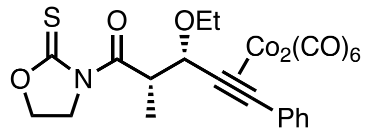

3a

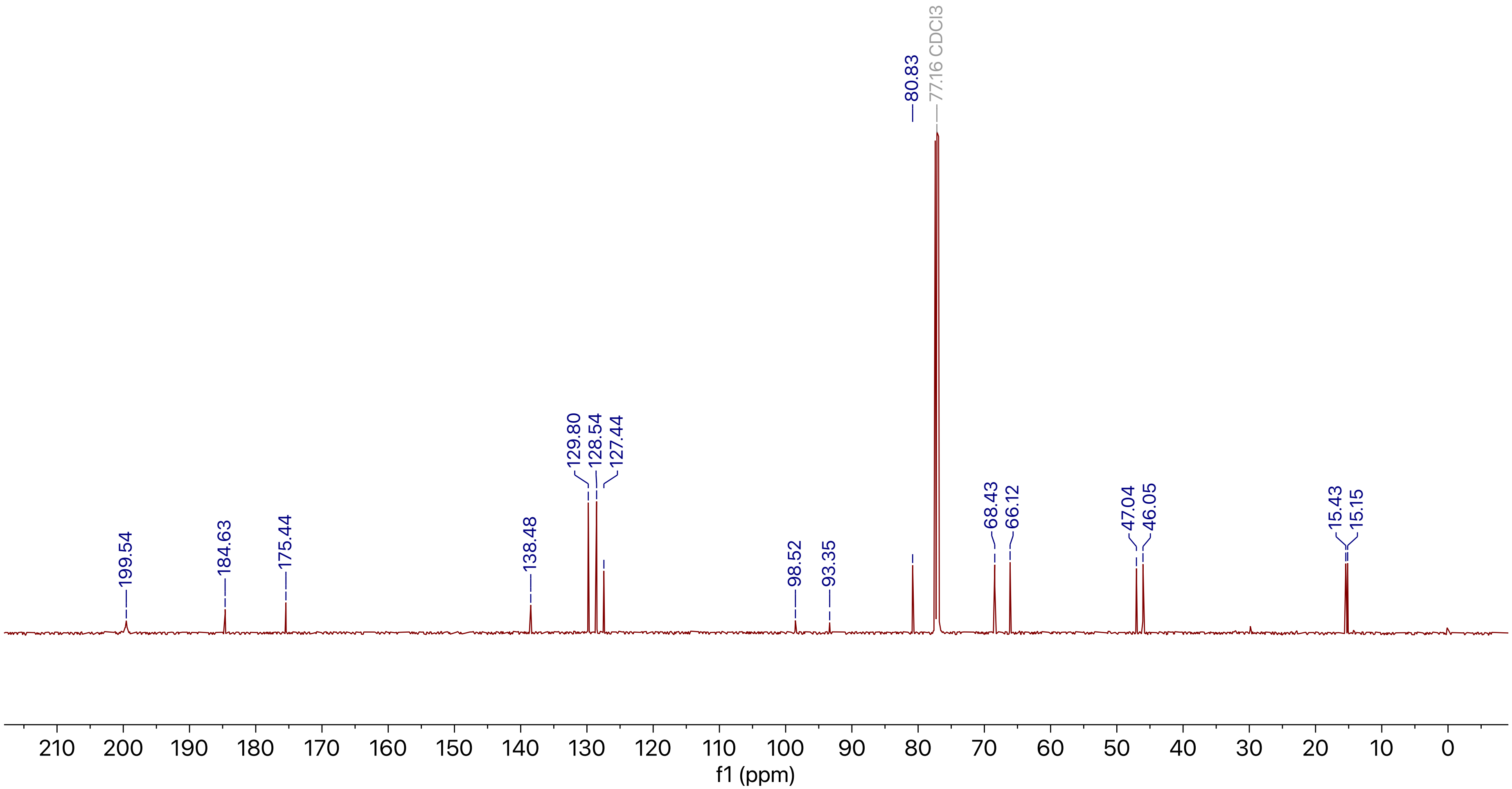

2D ^1H–^1H COSY (500 MHz, $CDCl_3$)

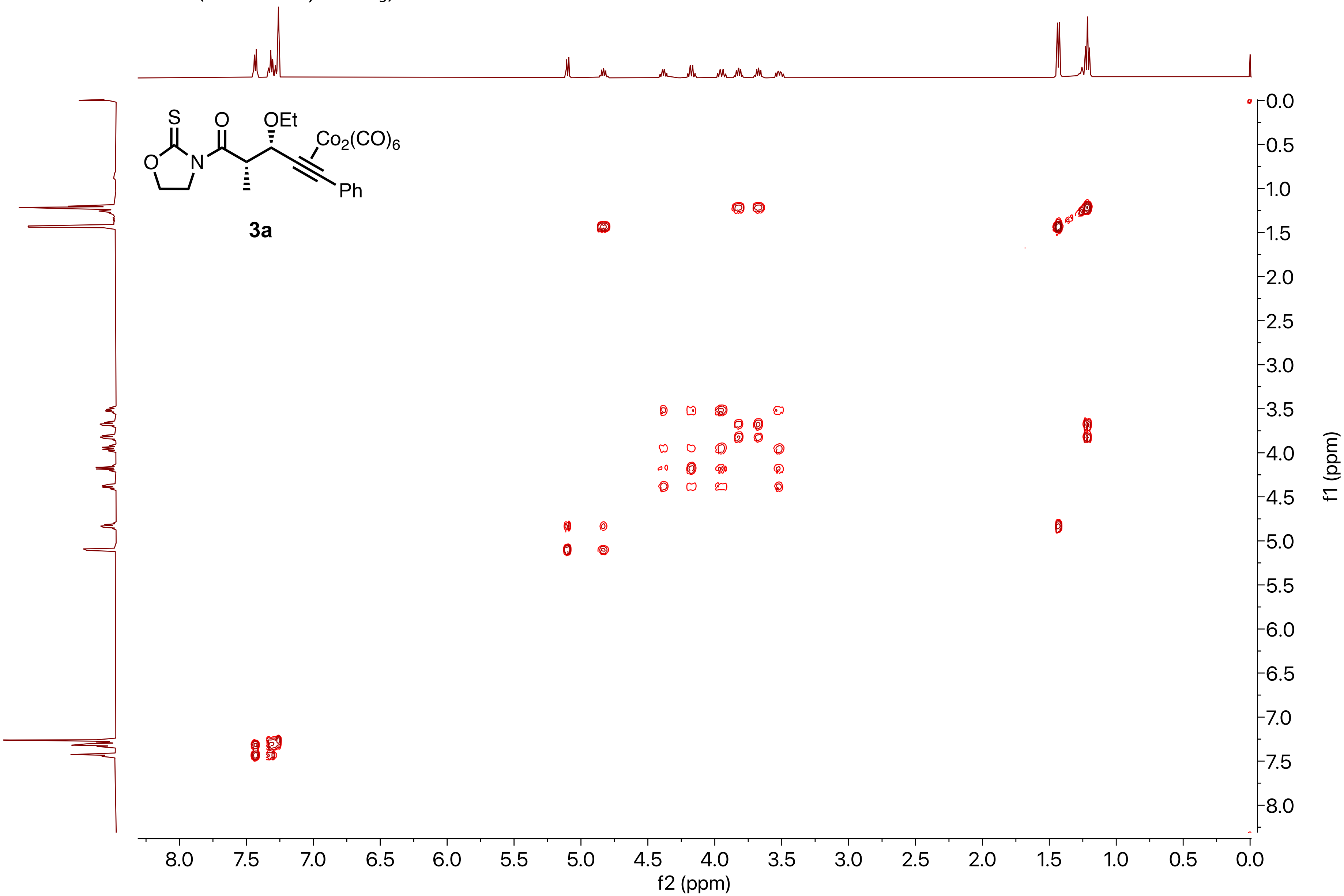

2D ^{1}H–^{13}C HSQC (500 MHz, $CDCl_3$)

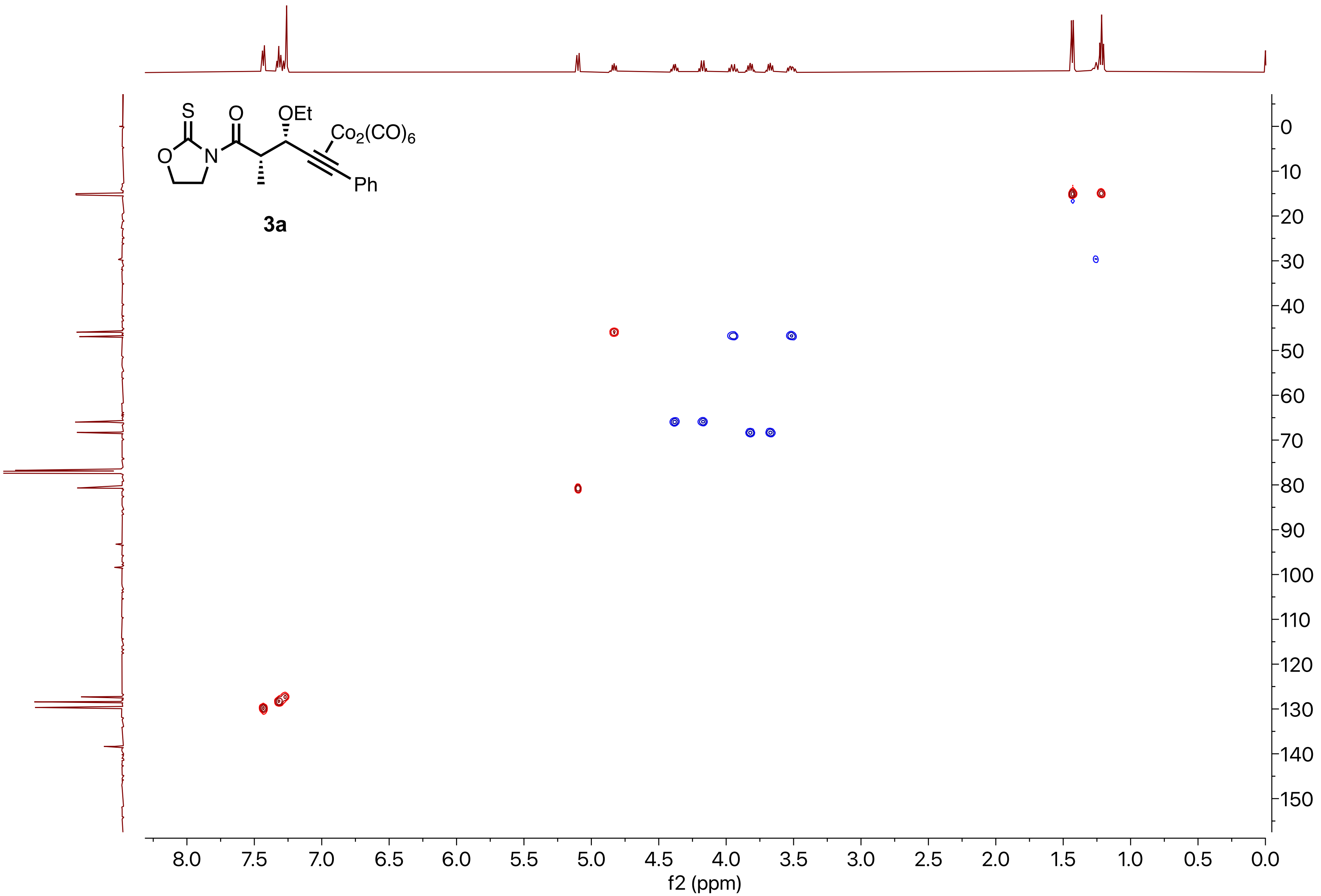

^{1}H NMR (400 MHz, $CDCl_3$)

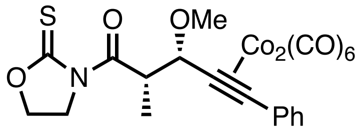

3b

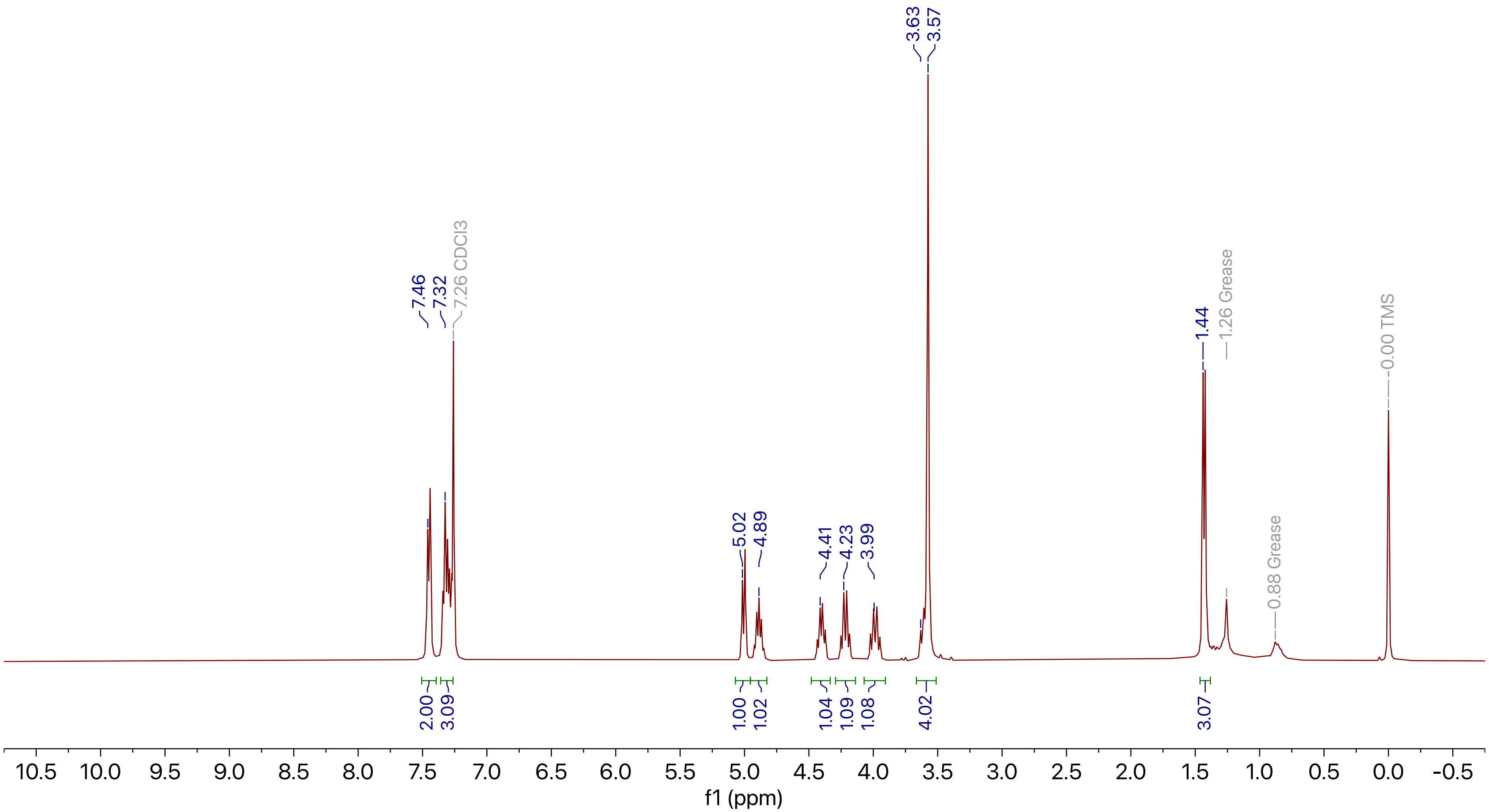

$^{13}C\{^{1}H\}$ NMR (101 MHz, $CDCl_3$)

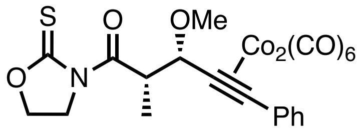

3b

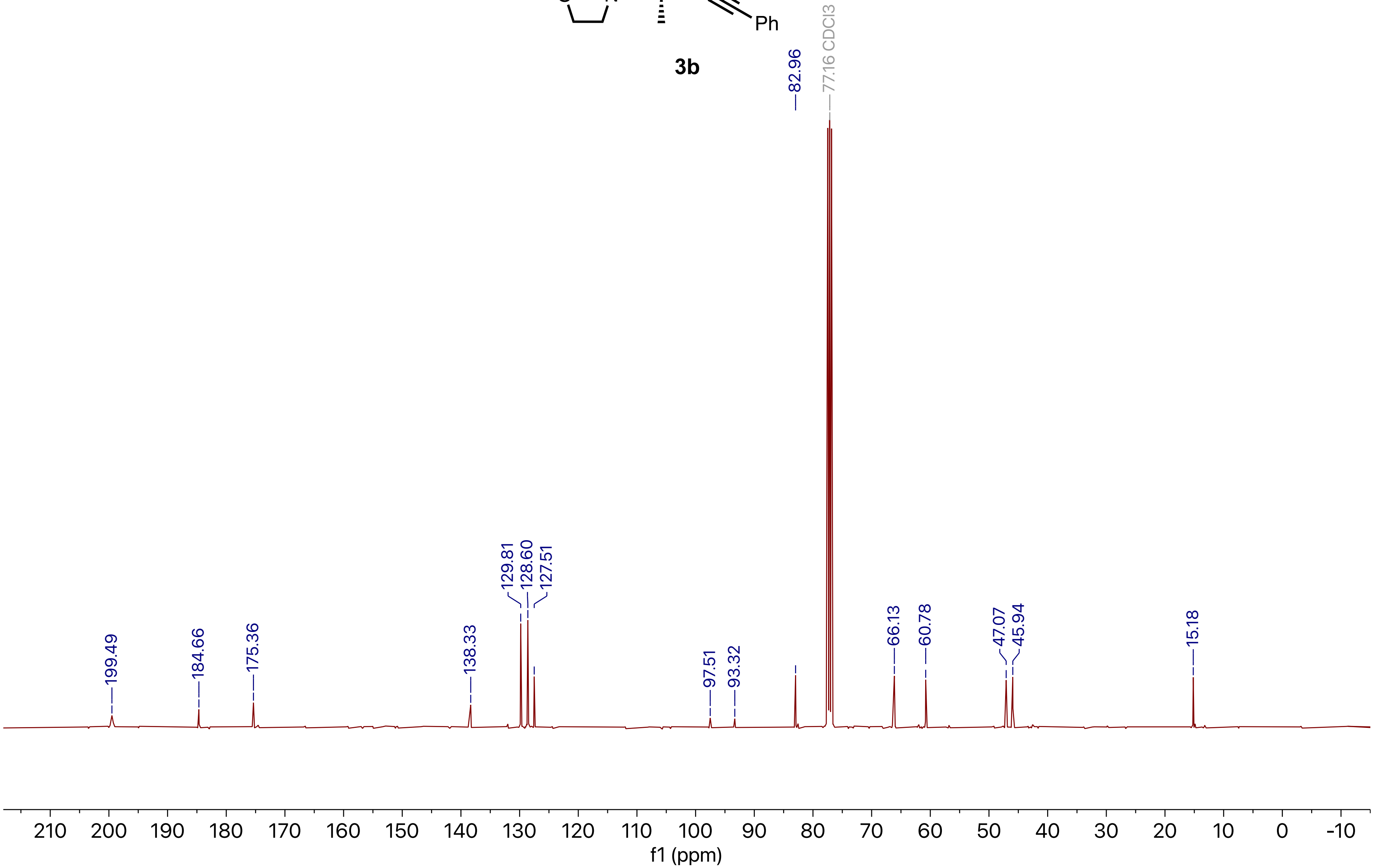

2D ^{1}H–^{1}H COSY (400 MHz, $CDCl_3$)

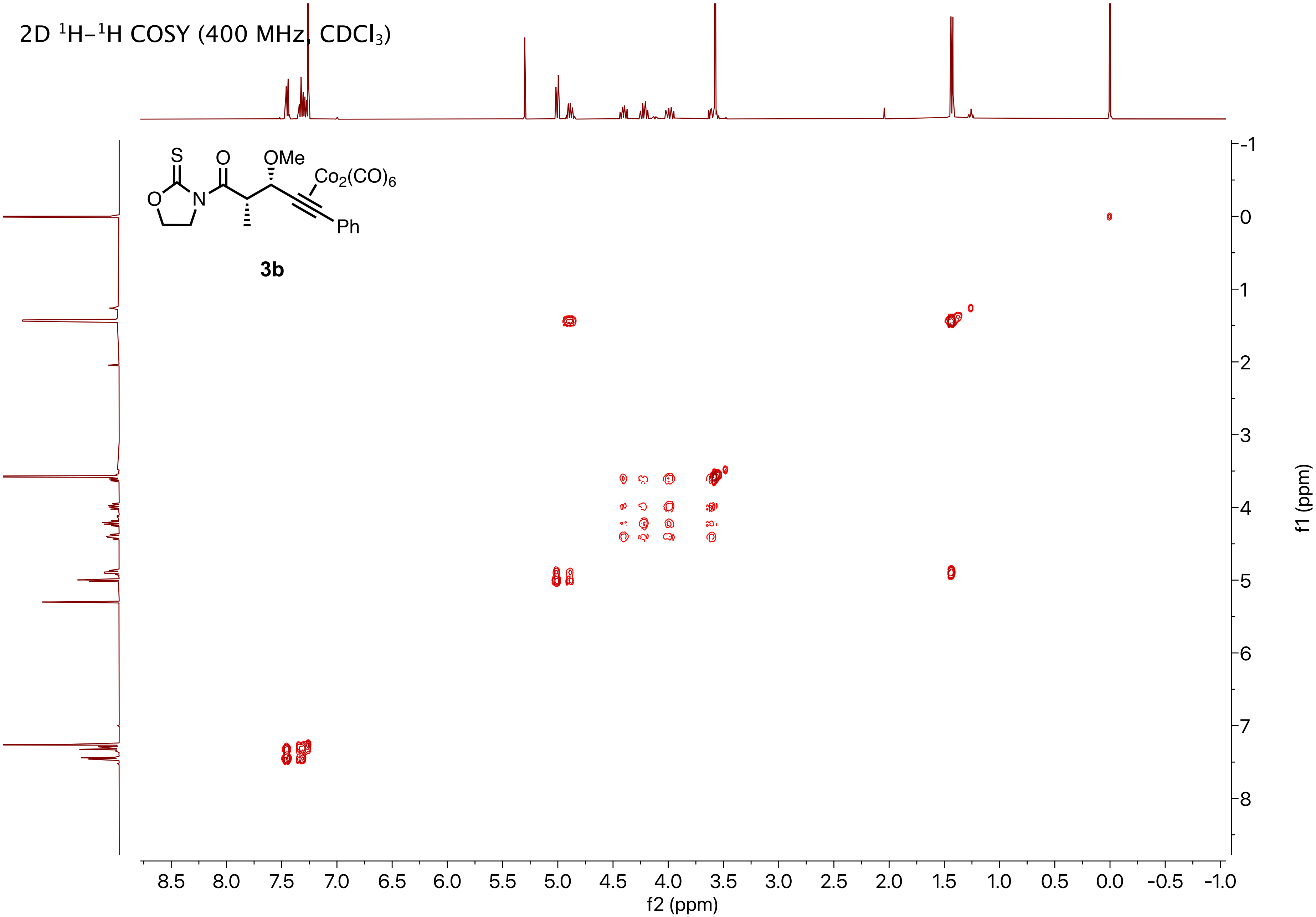

2D ^{1}H–^{13}C HSQC (400 MHz, $CDCl_3$)

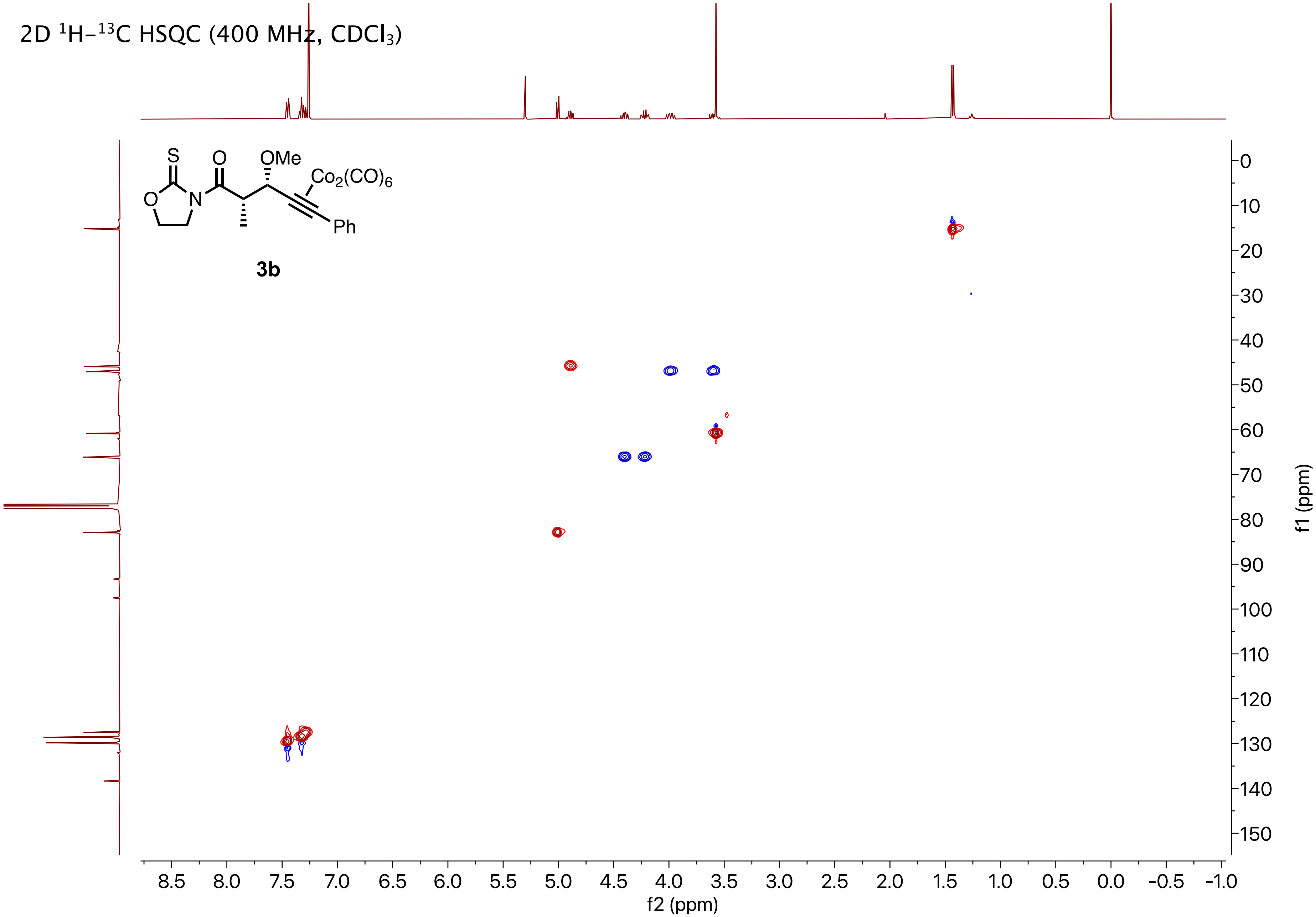

3b

^{1}H NMR (400 MHz, $CDCl_3$)

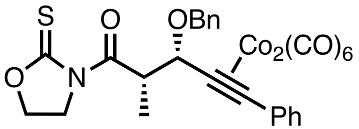

3c

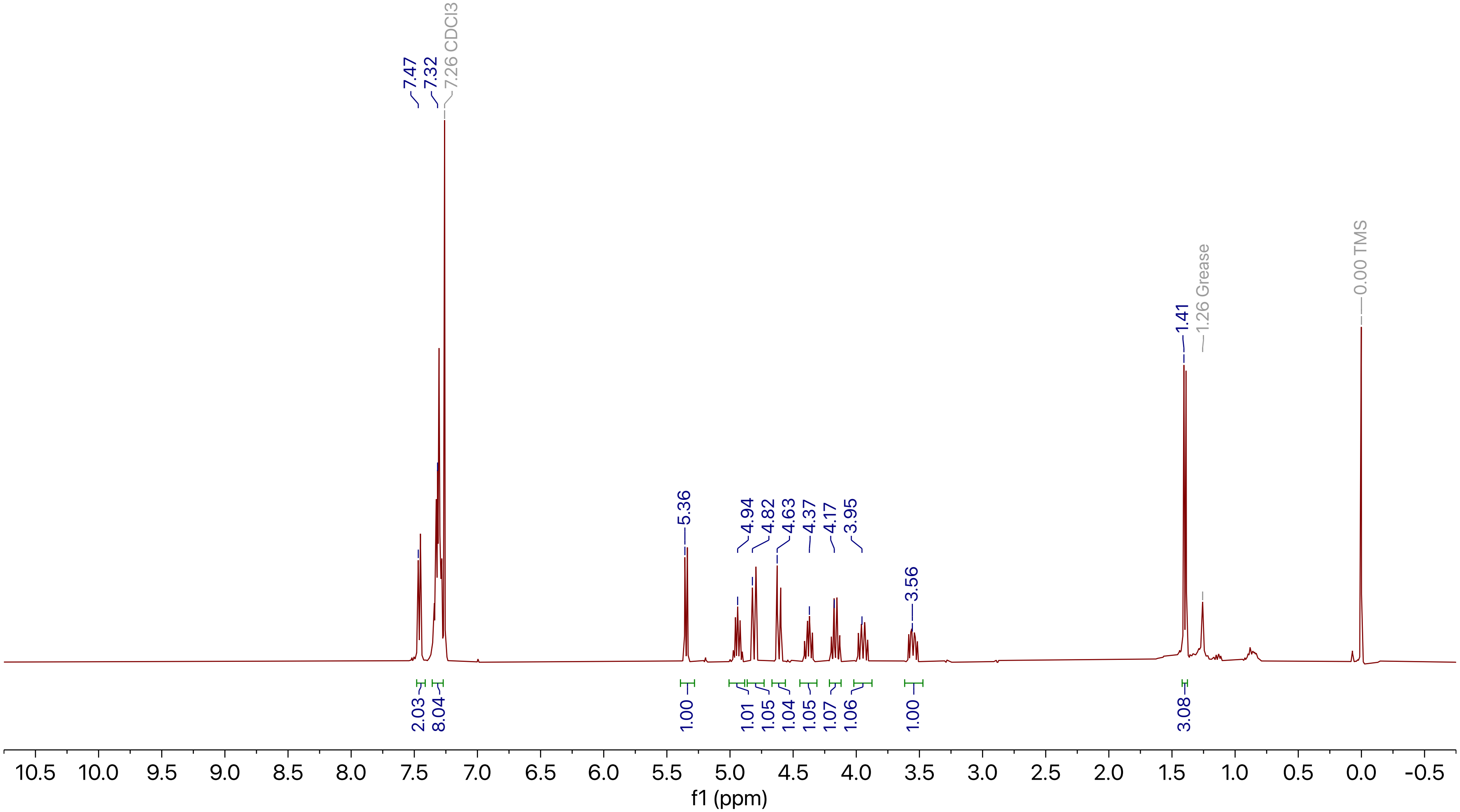

$^{13}C\{^{1}H\}$ NMR (101 MHz, $CDCl_3$)

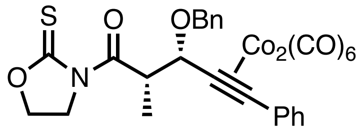

3c

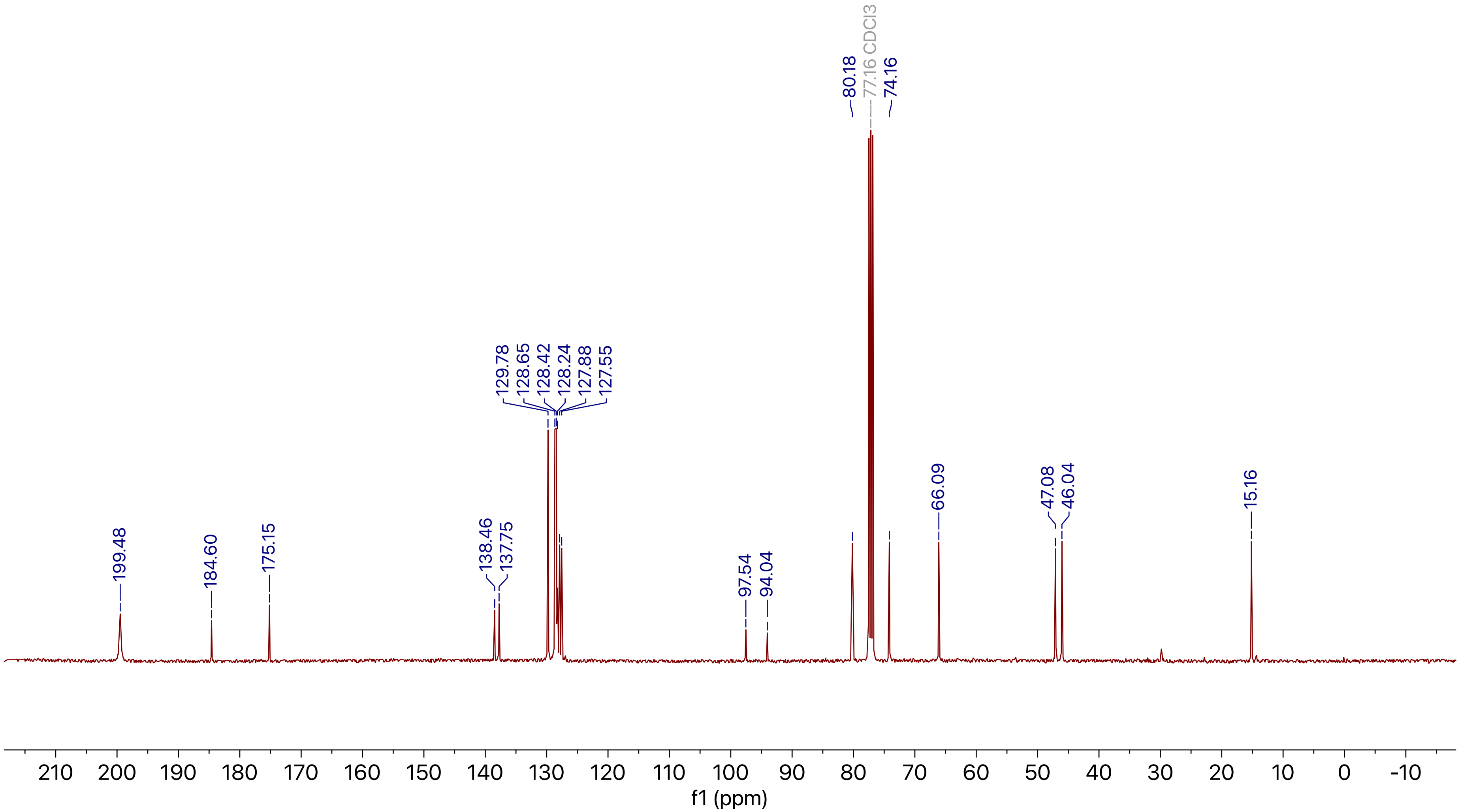

2D ^{1}H–^{1}H COSY (400 MHz, $CDCl_3$)

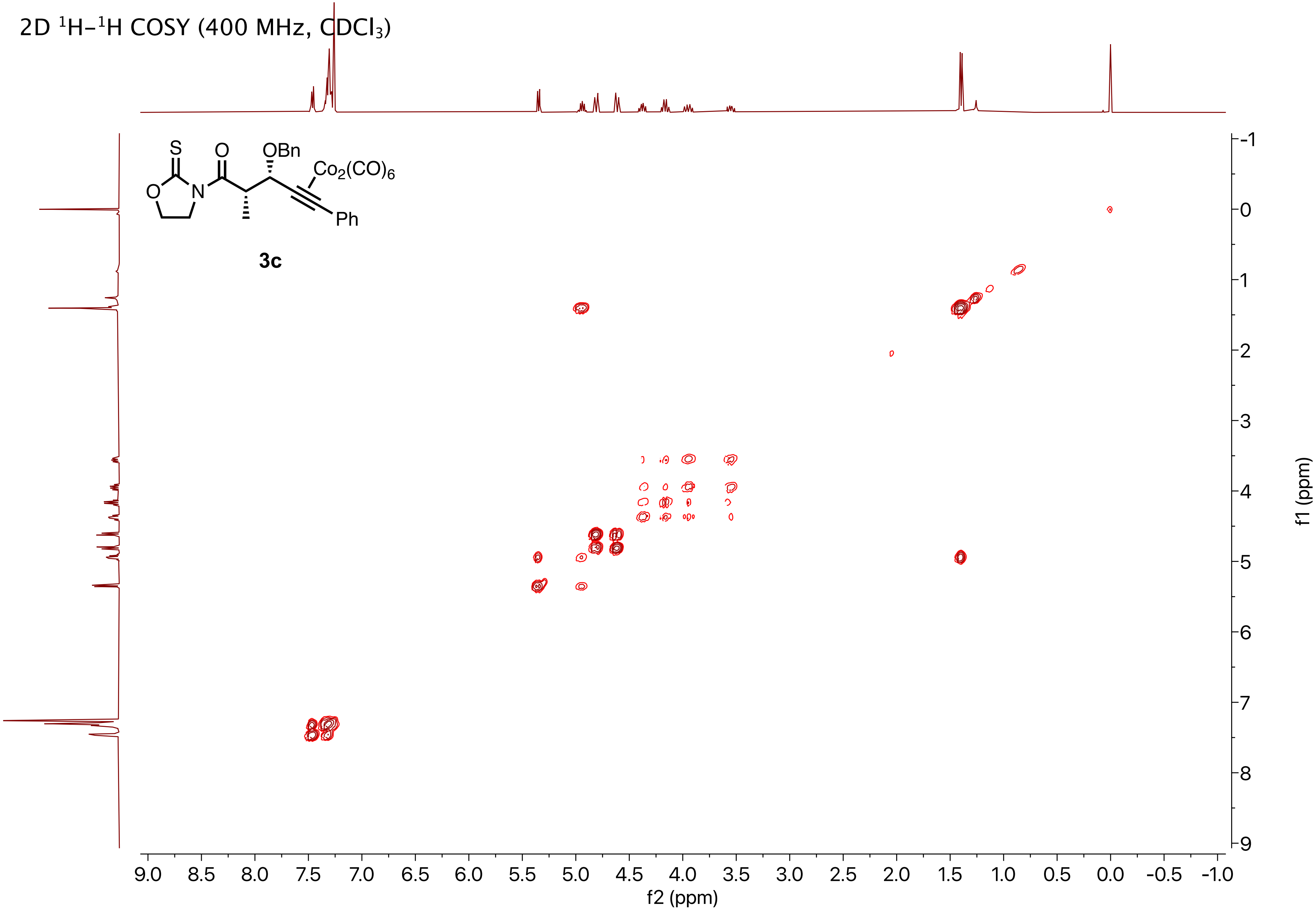

2D ^{1}H–^{13}C HSQC (400 MHz, $CDCl_3$)

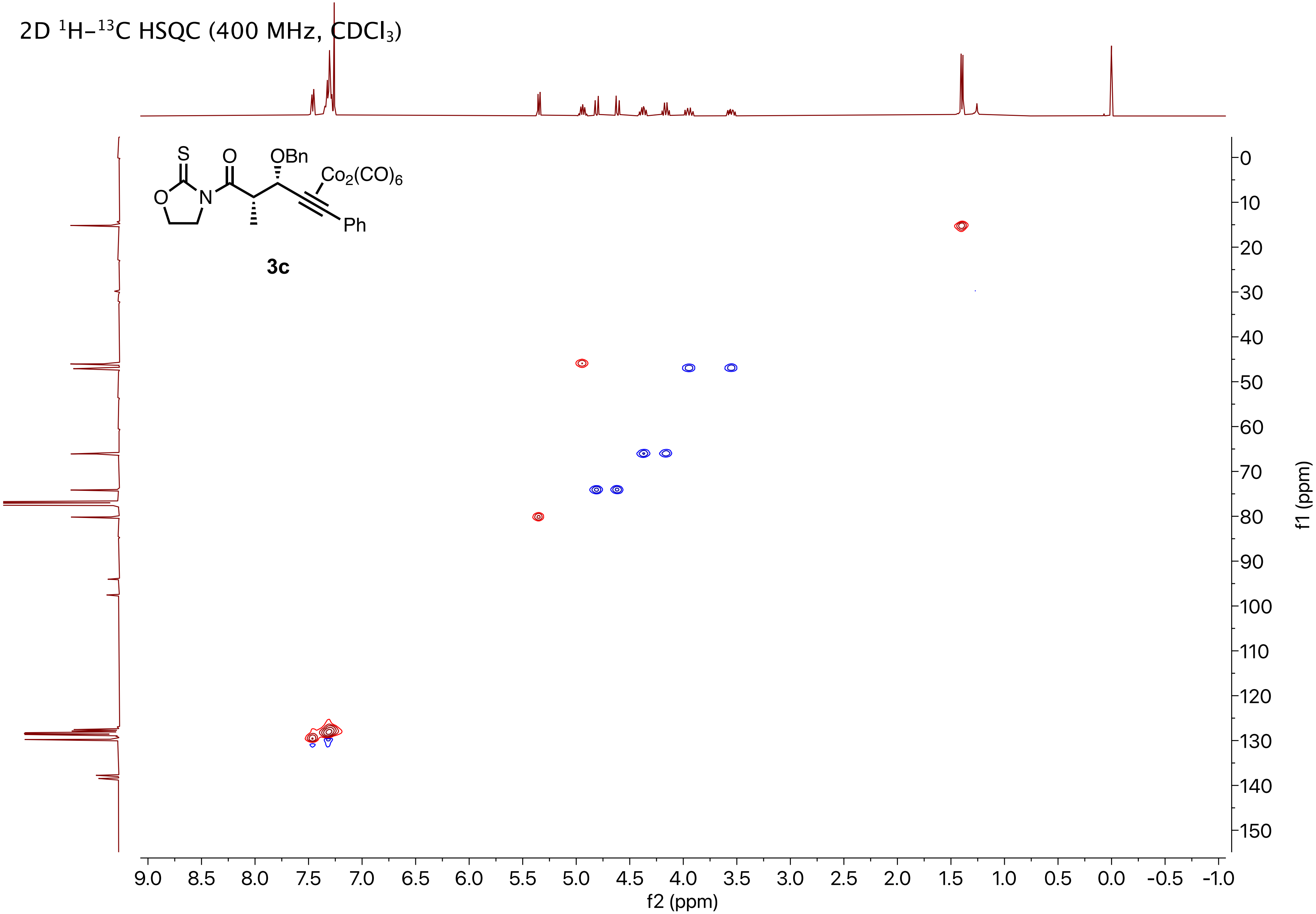

^{1}H NMR (400 MHz, $CDCl_3$)

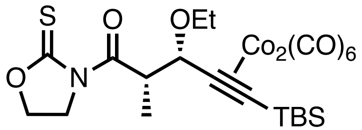

3d

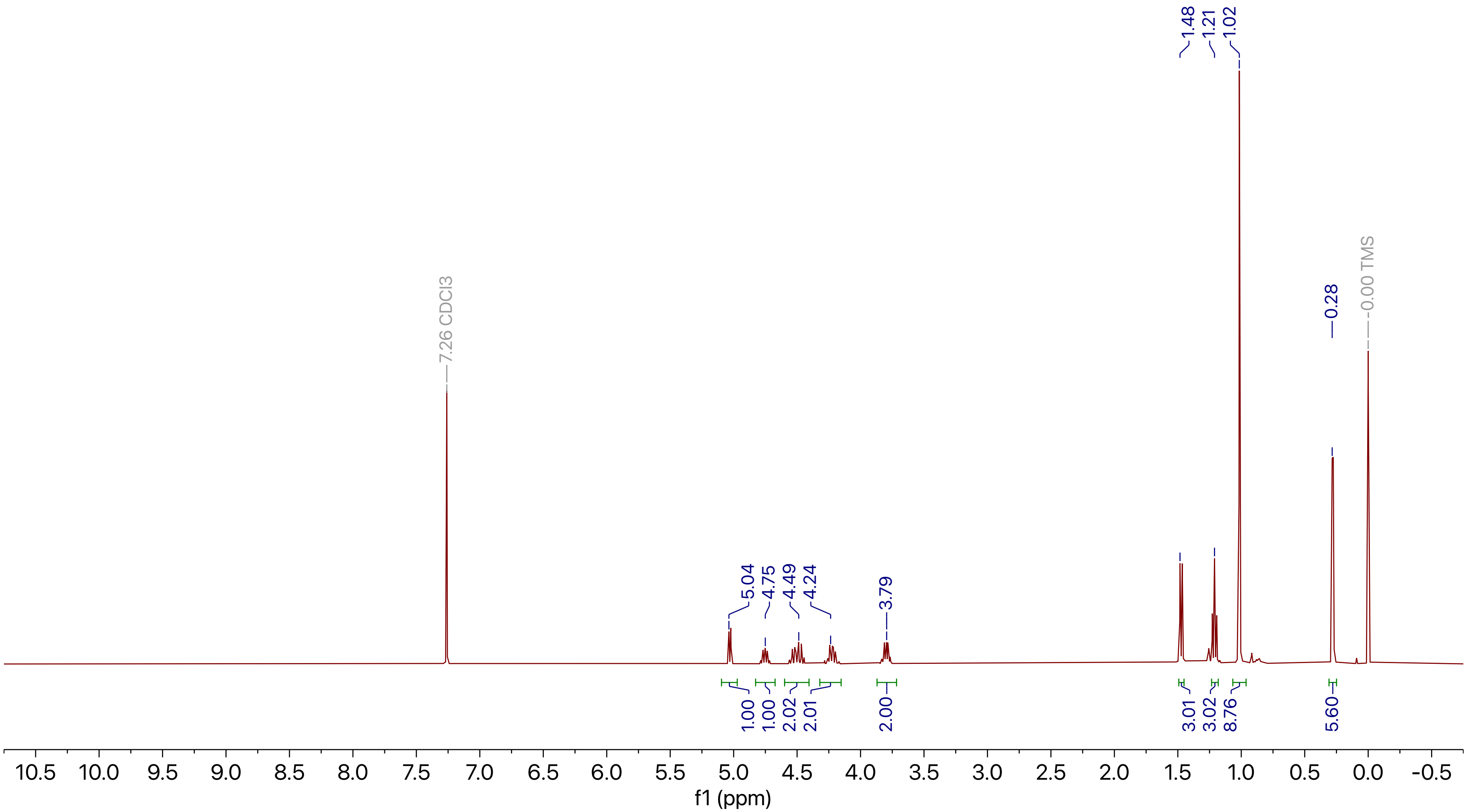

$^{13}C\{^{1}H\}$ NMR (101 MHz, $CDCl_3$)

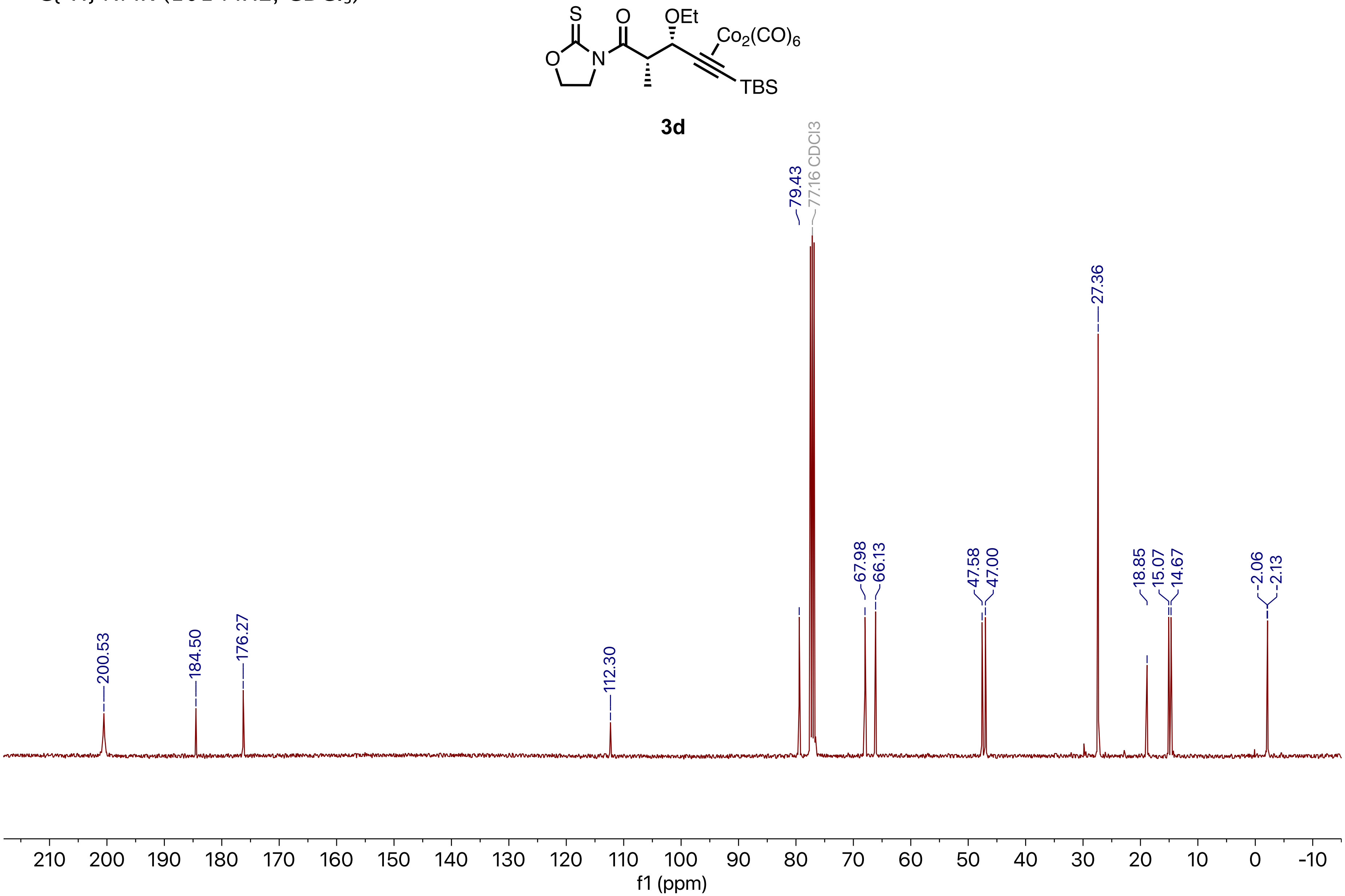

2D ^1H–^1H COSY (400 MHz, $CDCl_3$)

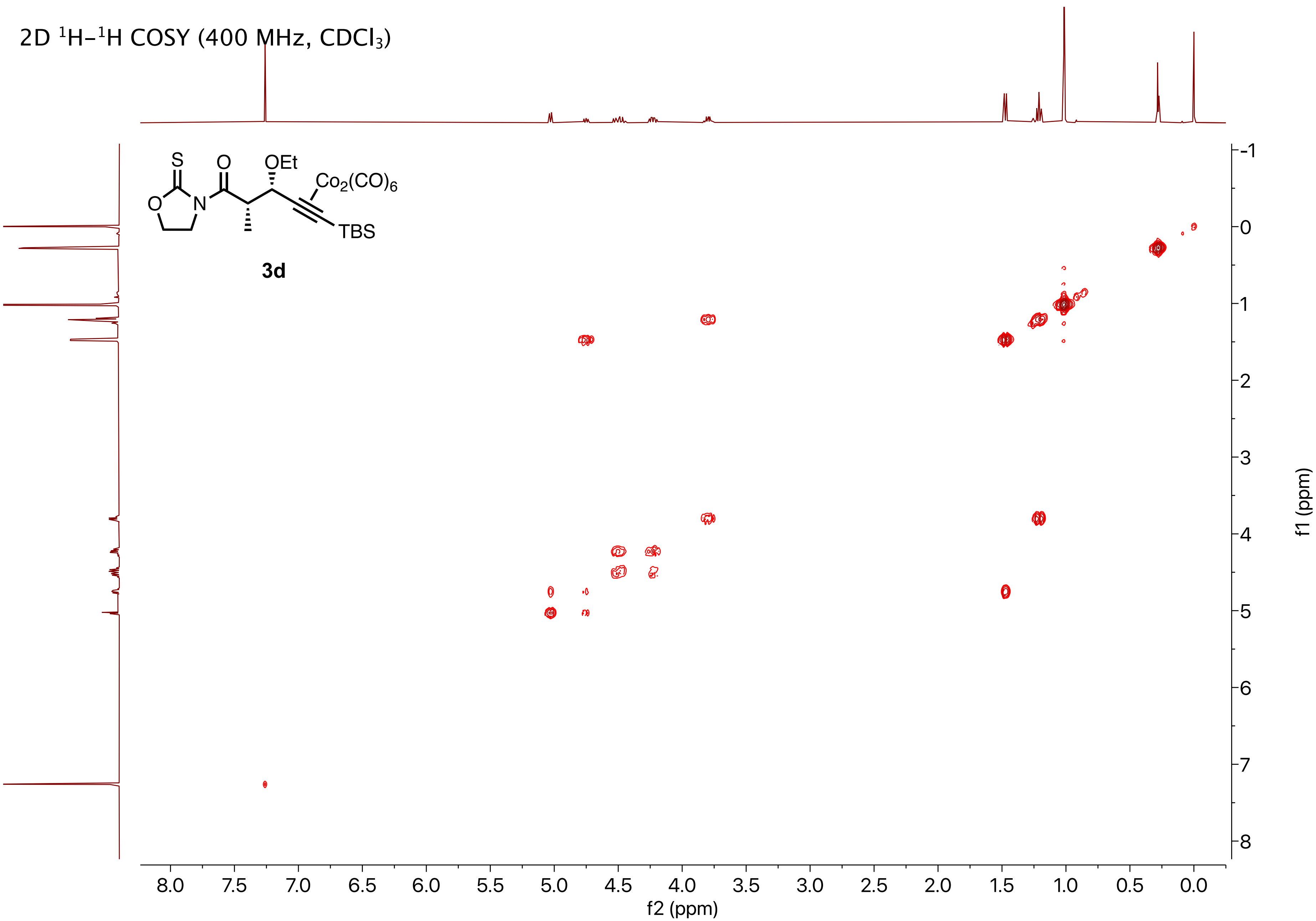

3d

2D ^{1}H–^{13}C HSQC (400 MHz, $CDCl_3$)

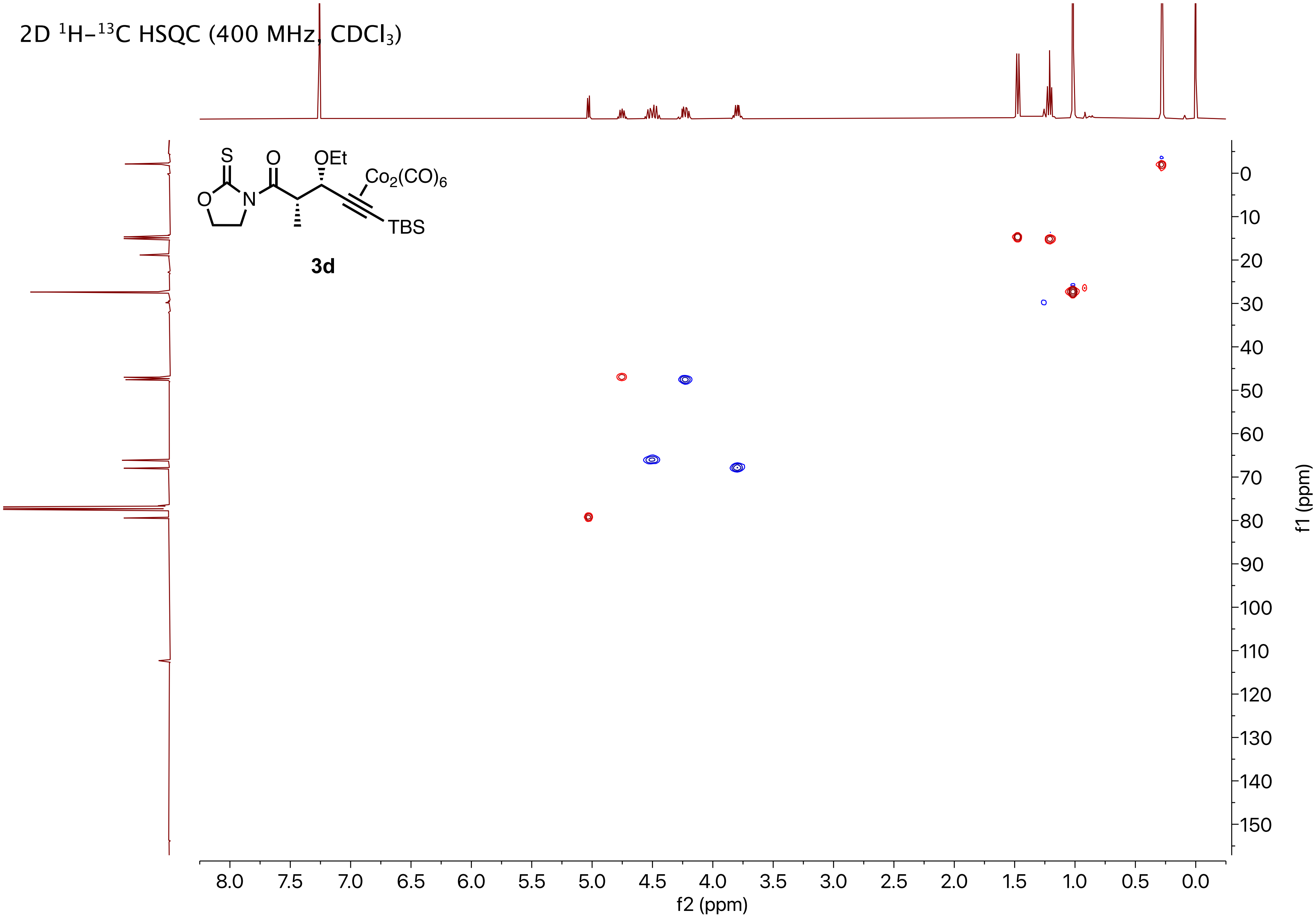

^{1}H NMR (400 MHz, $CDCl_3$)

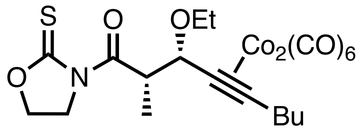

3e

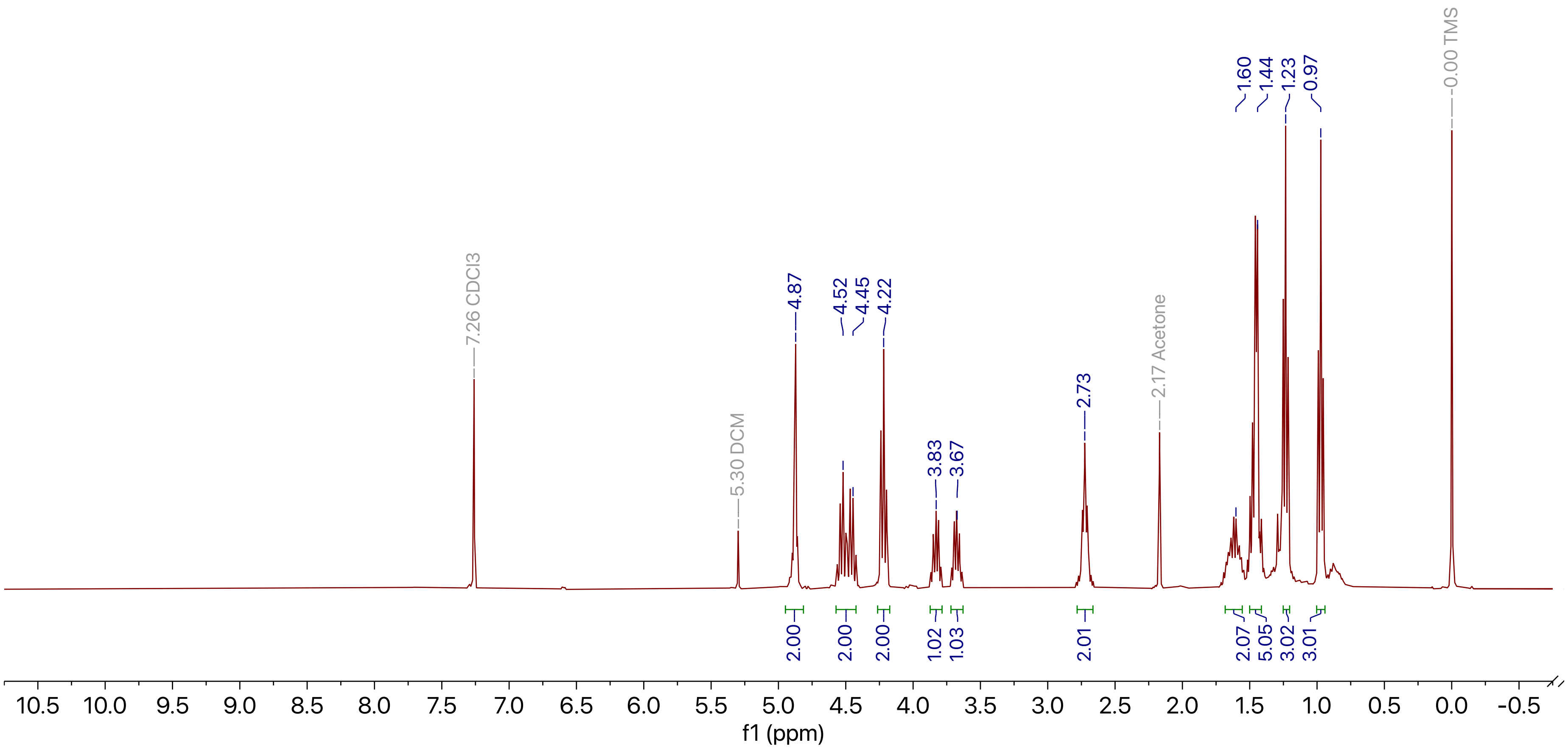

$^{13}C\{^1H\}$ NMR (101 MHz, $CDCl_3$)

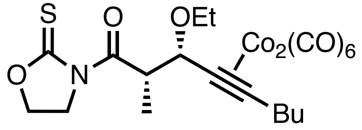

3e

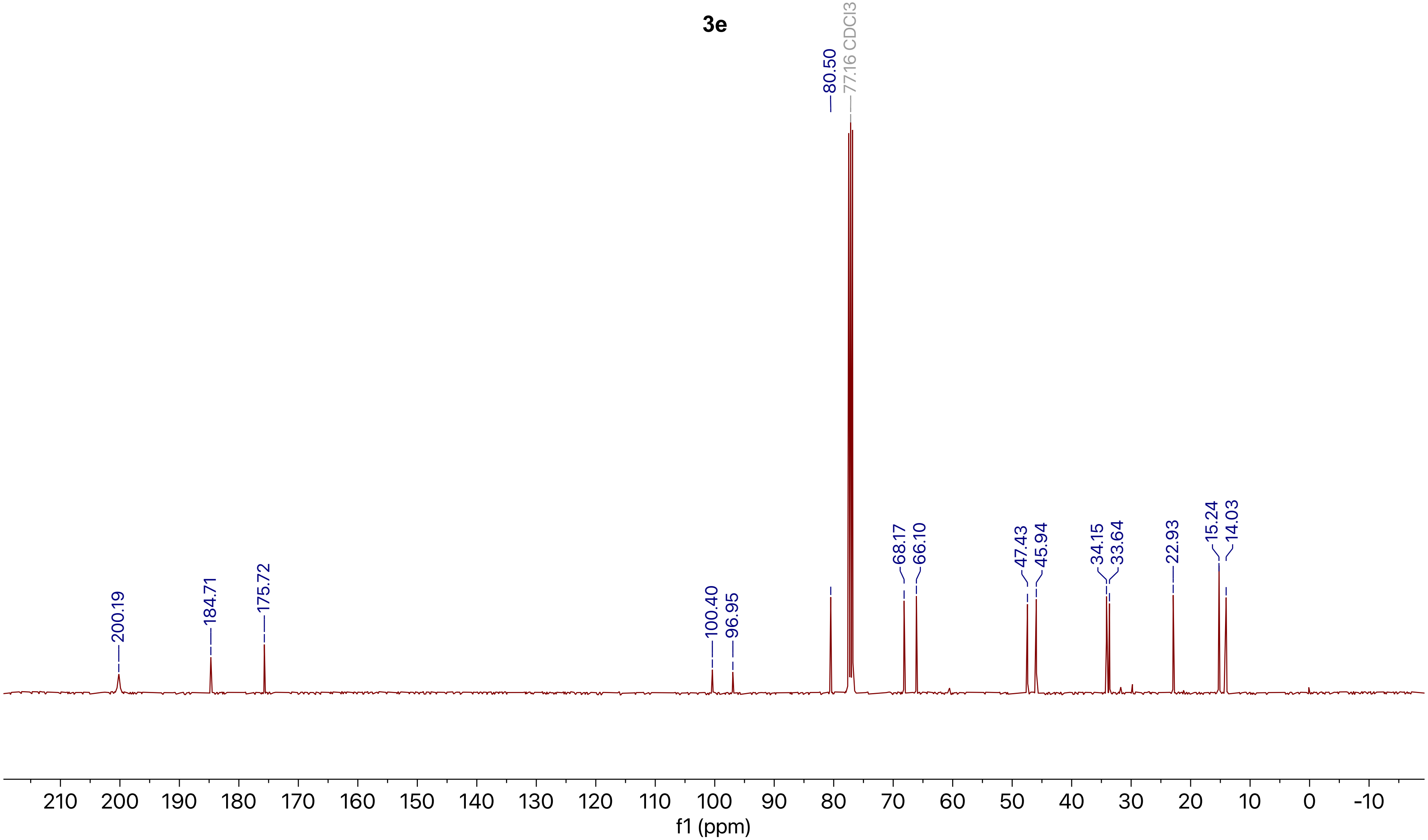

2D ^{1}H–^{1}H COSY (400 MHz, $CDCl_3$)

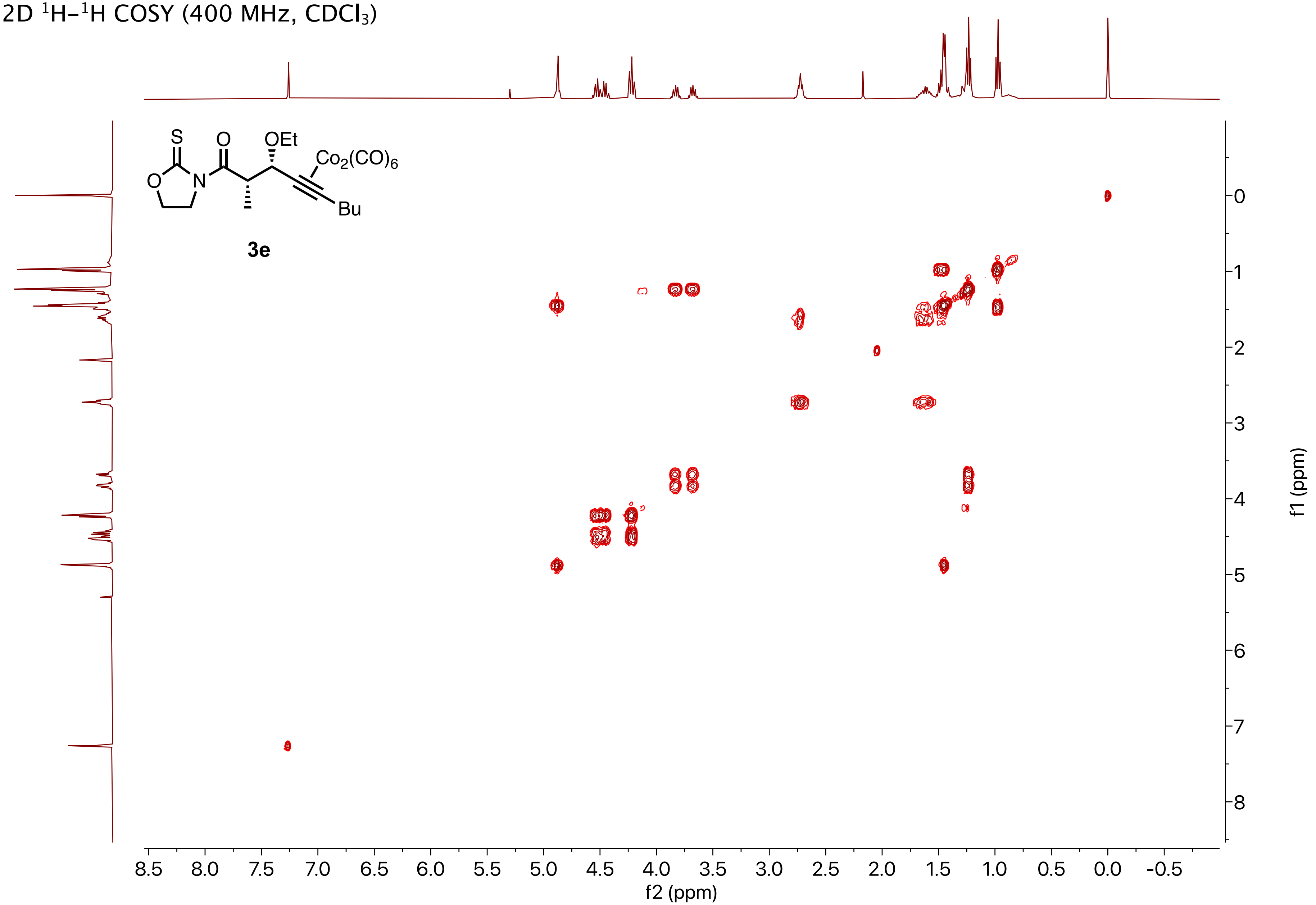

2D $^{1}H-^{13}C$ HSQC (400 MHz, $CDCl_3$)

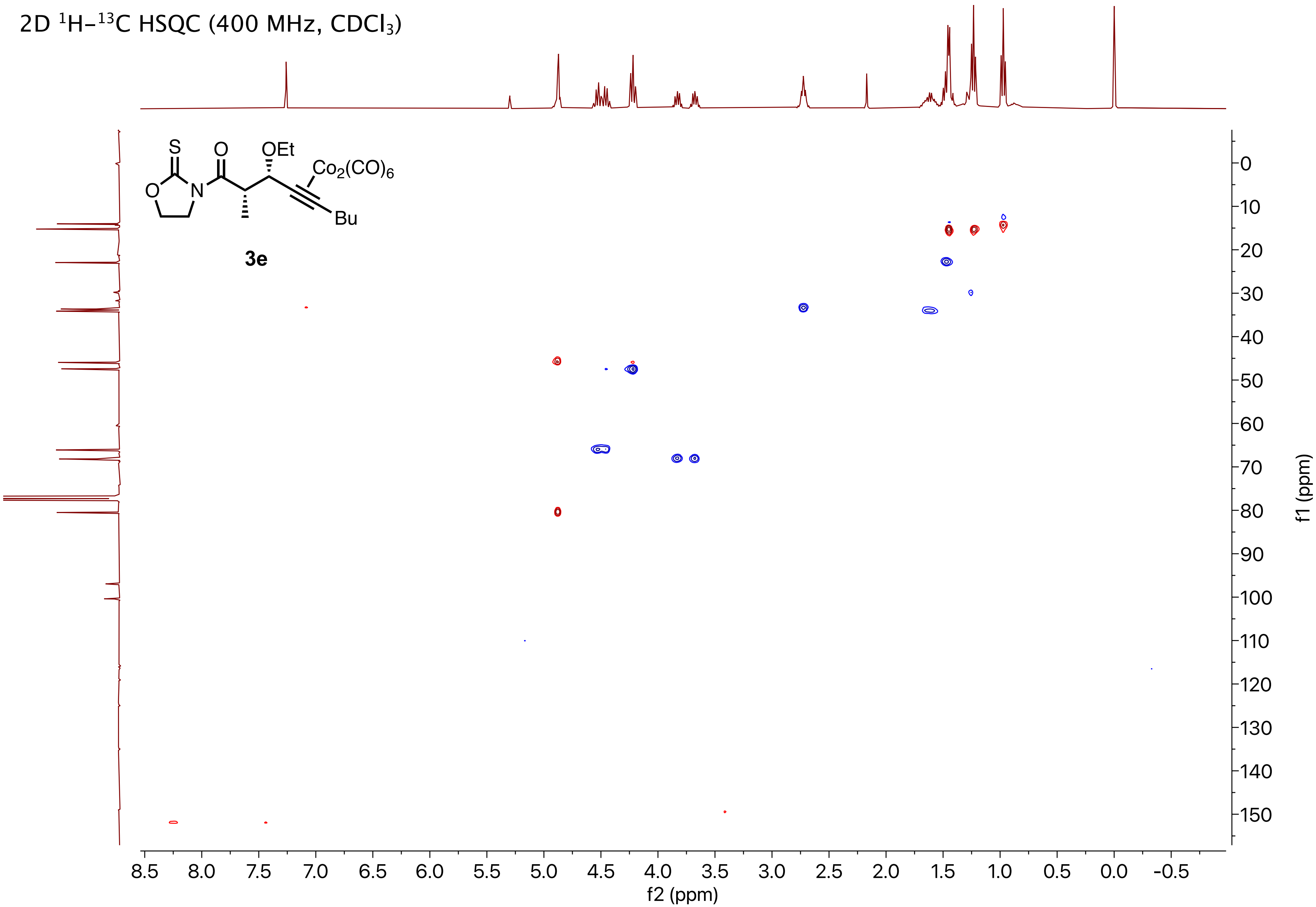

^{1}H NMR (400 MHz, $CDCl_3$)

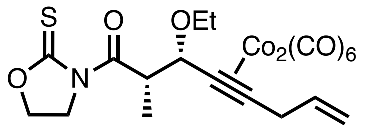

3f

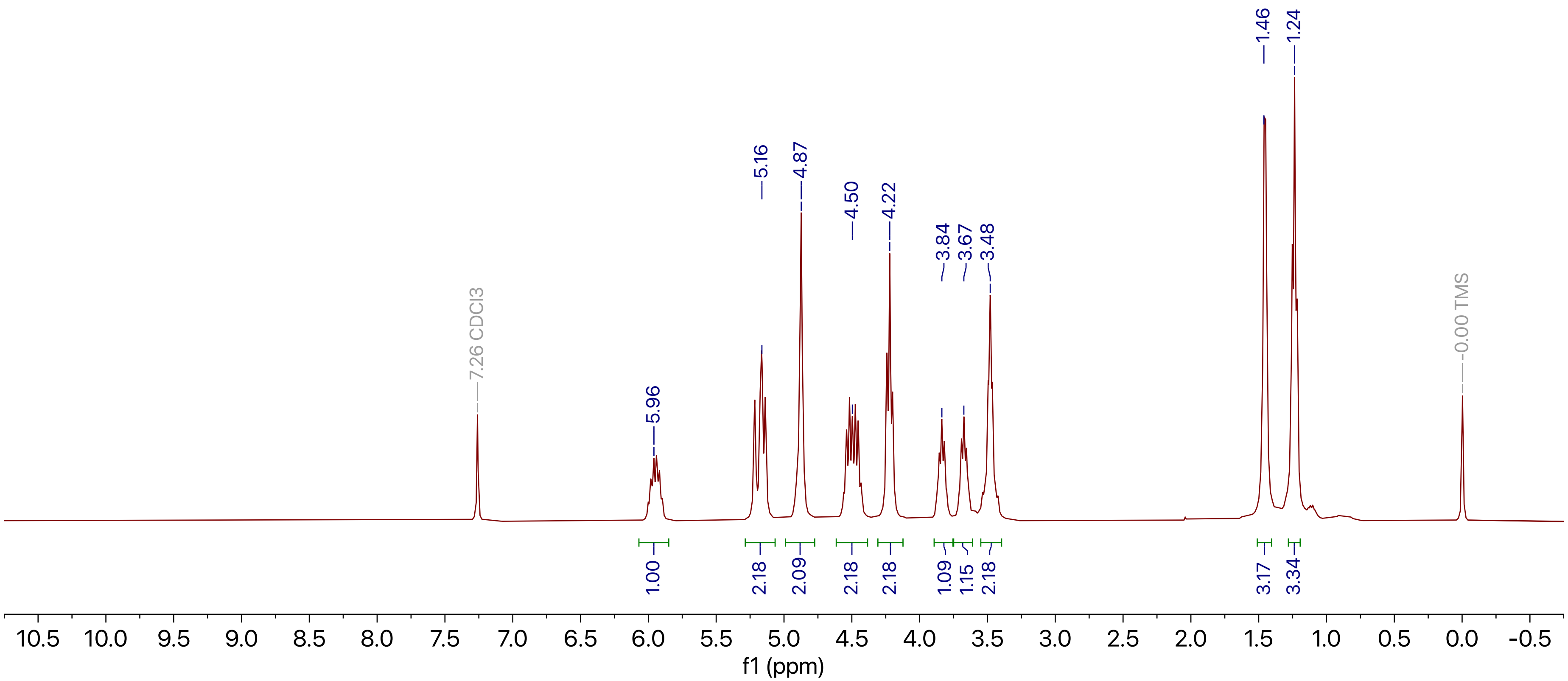

$^{13}C\{^{1}H\}$ NMR (101 MHz, $CDCl_3$)

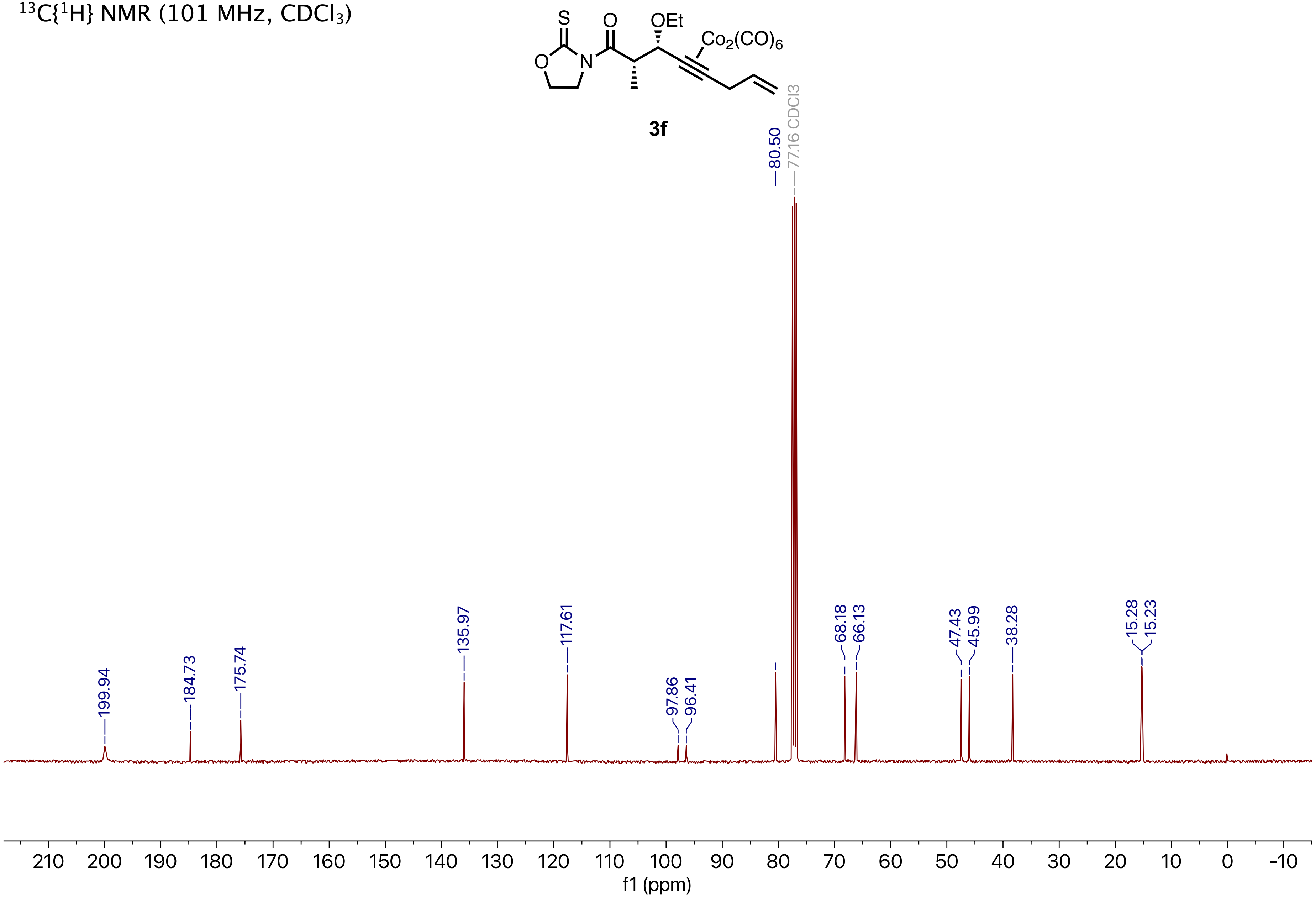

2D ^{1}H–^{1}H COSY (400 MHz, $CDCl_3$)

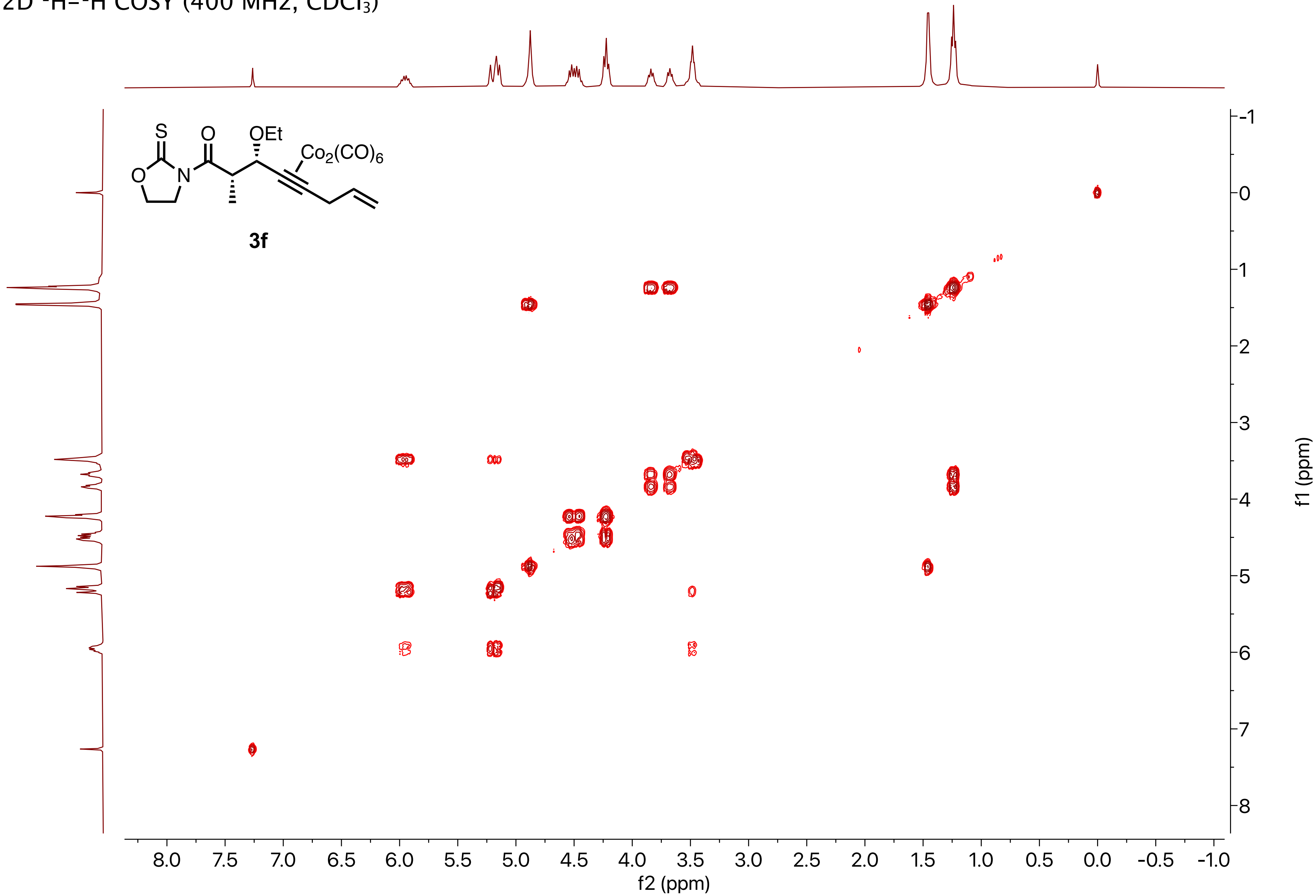

2D ^1H–^{13}C HSQC (400 MHz, $CDCl_3$)

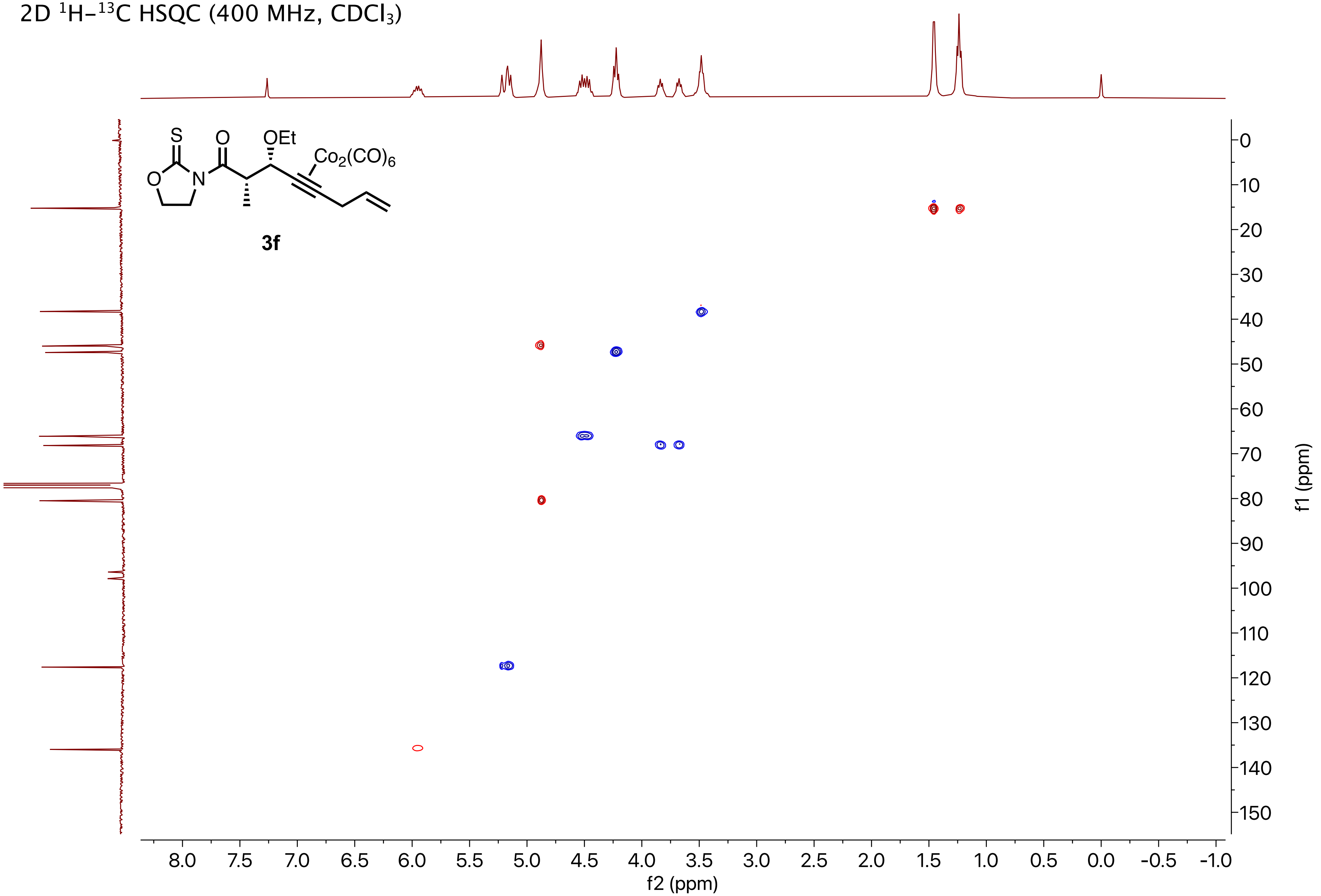

^{1}H NMR (500 MHz, $CDCl_3$)

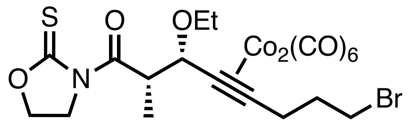

3g

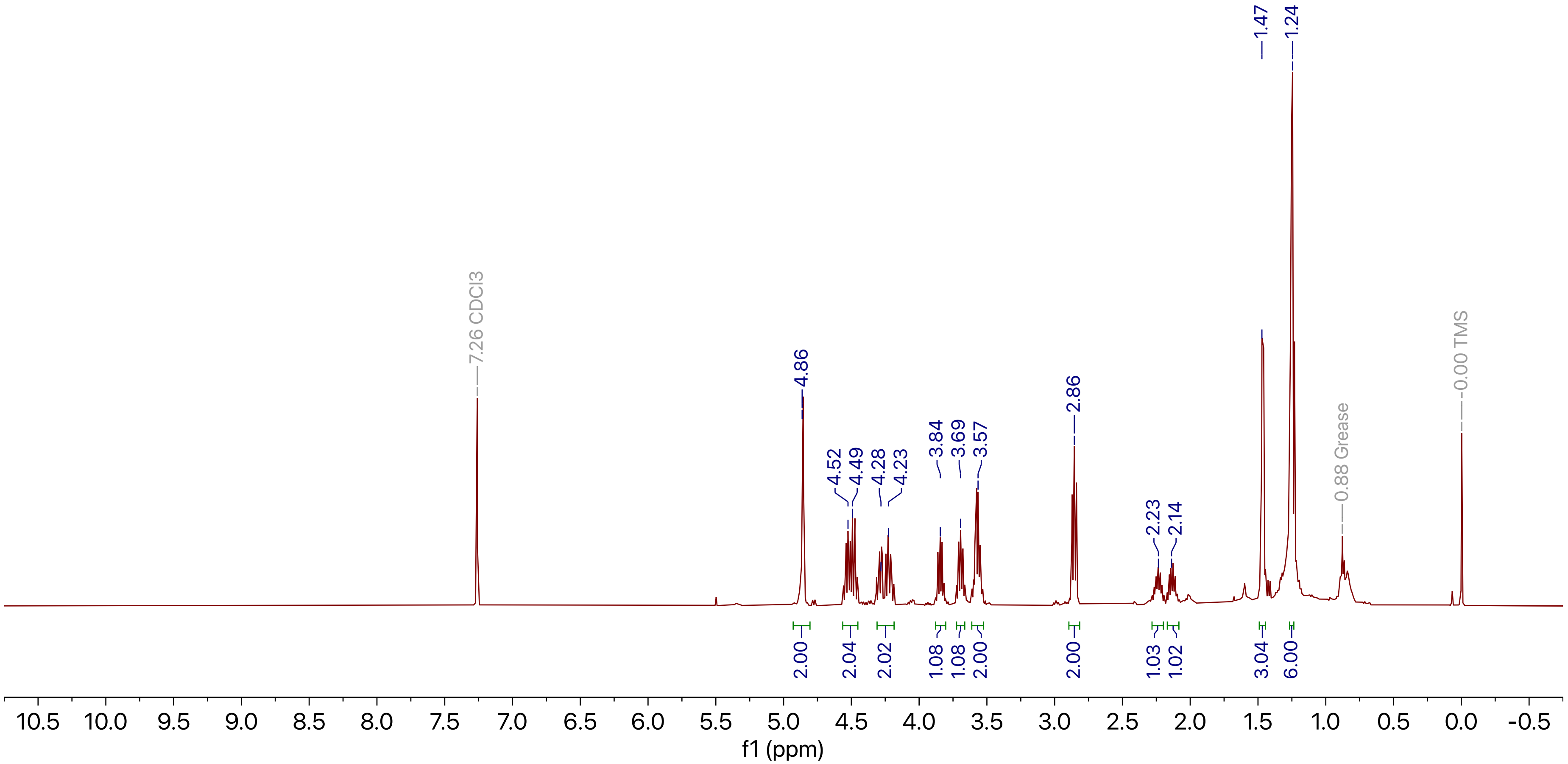

$^{13}C\{^{1}H\}$ NMR (126 MHz, $CDCl_3$)

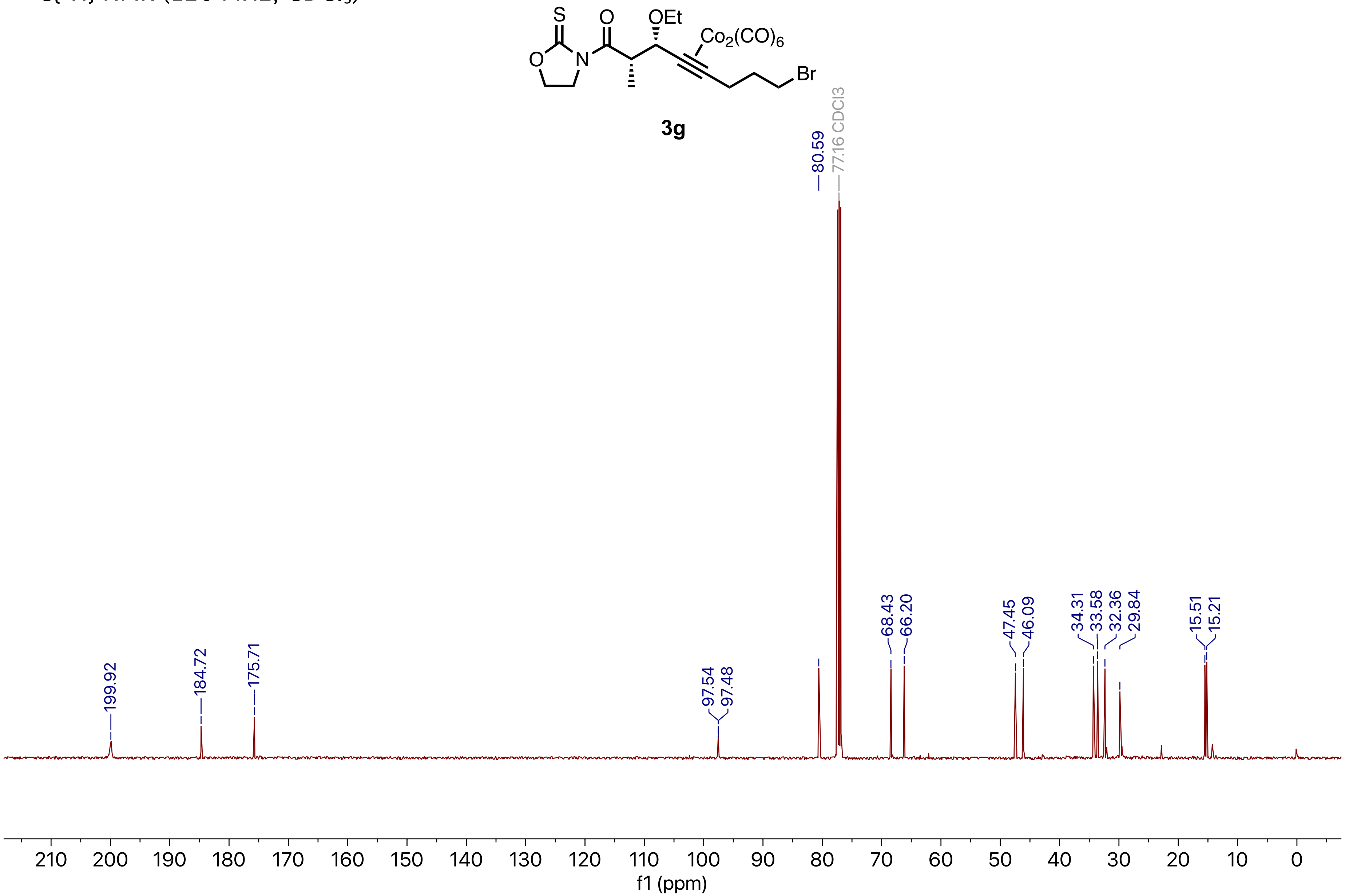

2D ^1H–^1H COSY (500 MHz, $CDCl_3$)

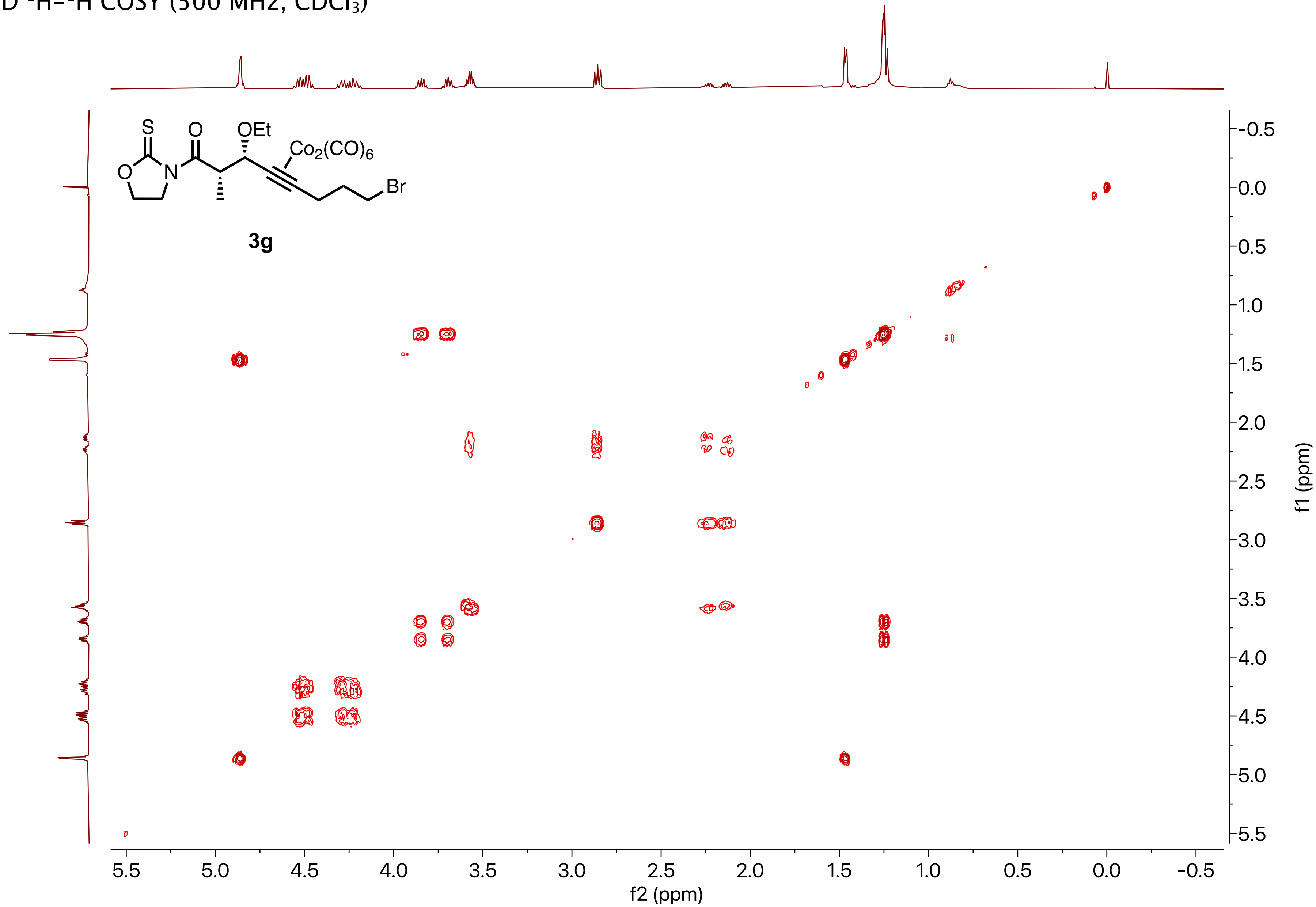

2D ^{1}H–^{13}C HSQC (500 MHz, $CDCl_3$)

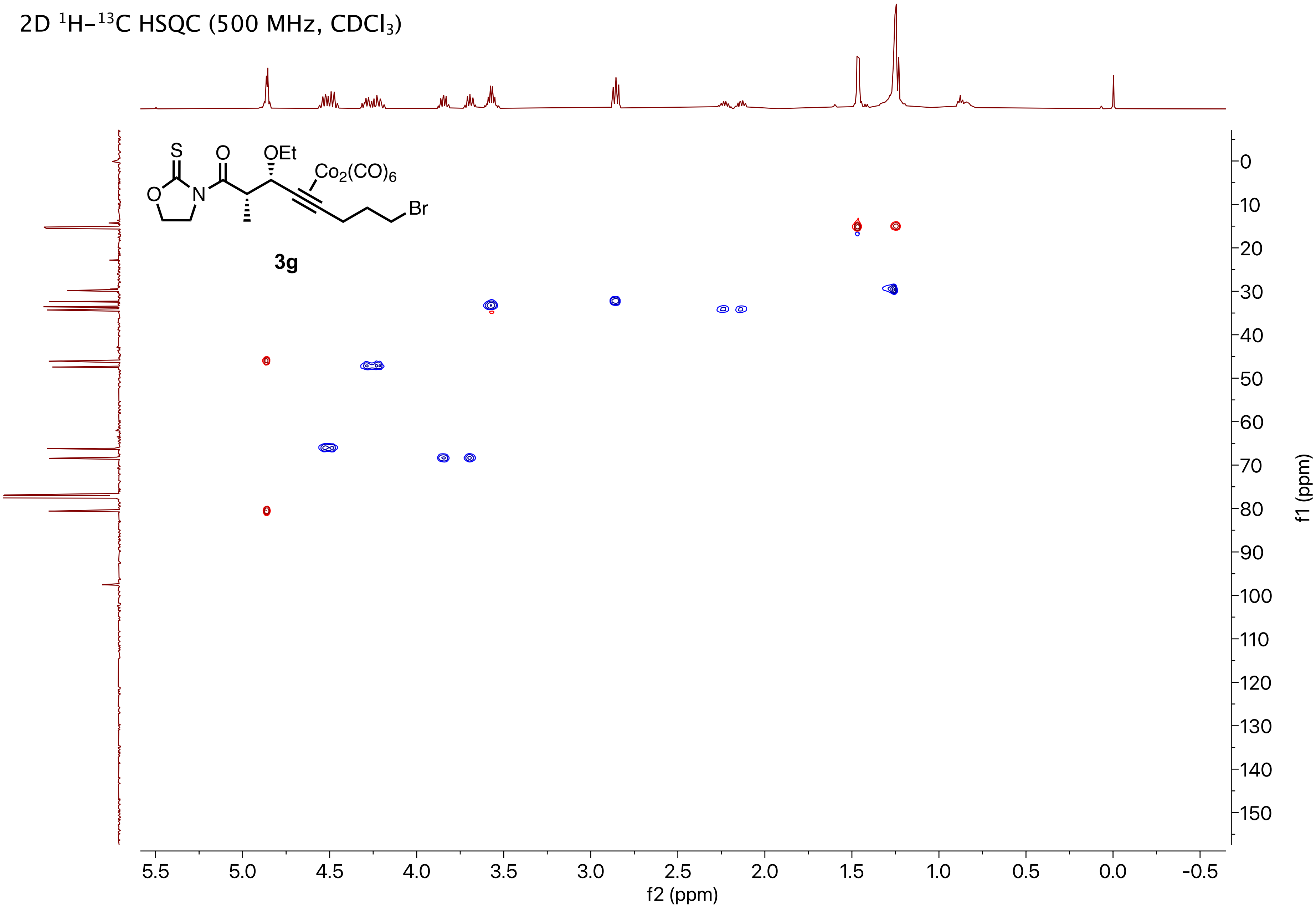

3g

^{1}H NMR (400 MHz, $CDCl_3$)

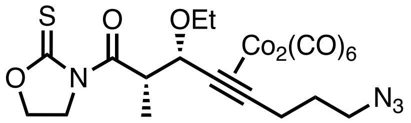

3h

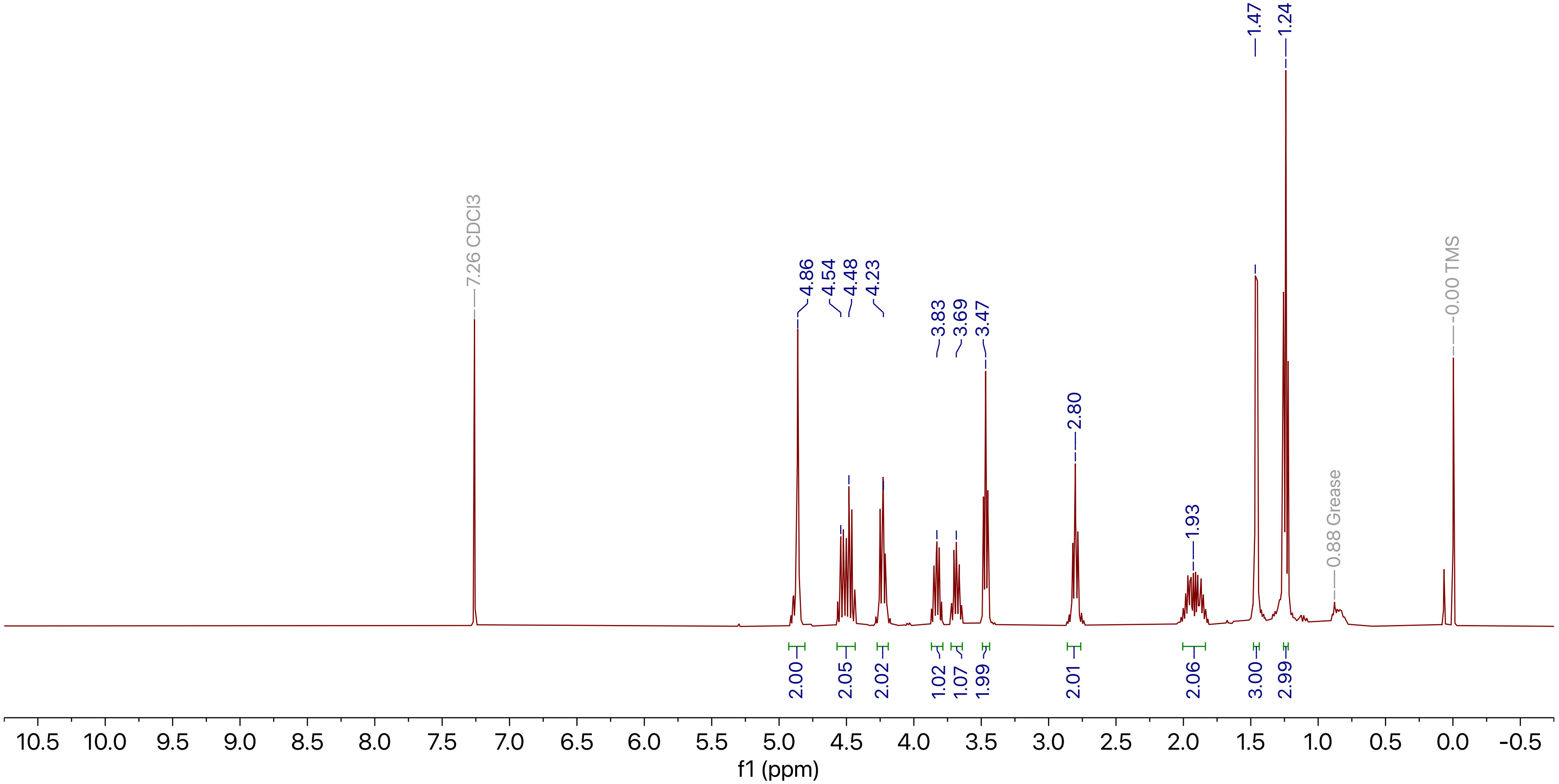

$^{13}C\{^{1}H\}$ NMR (101 MHz, $CDCl_3$)

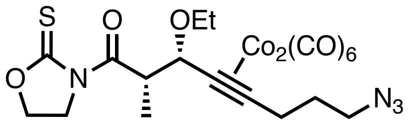

3h

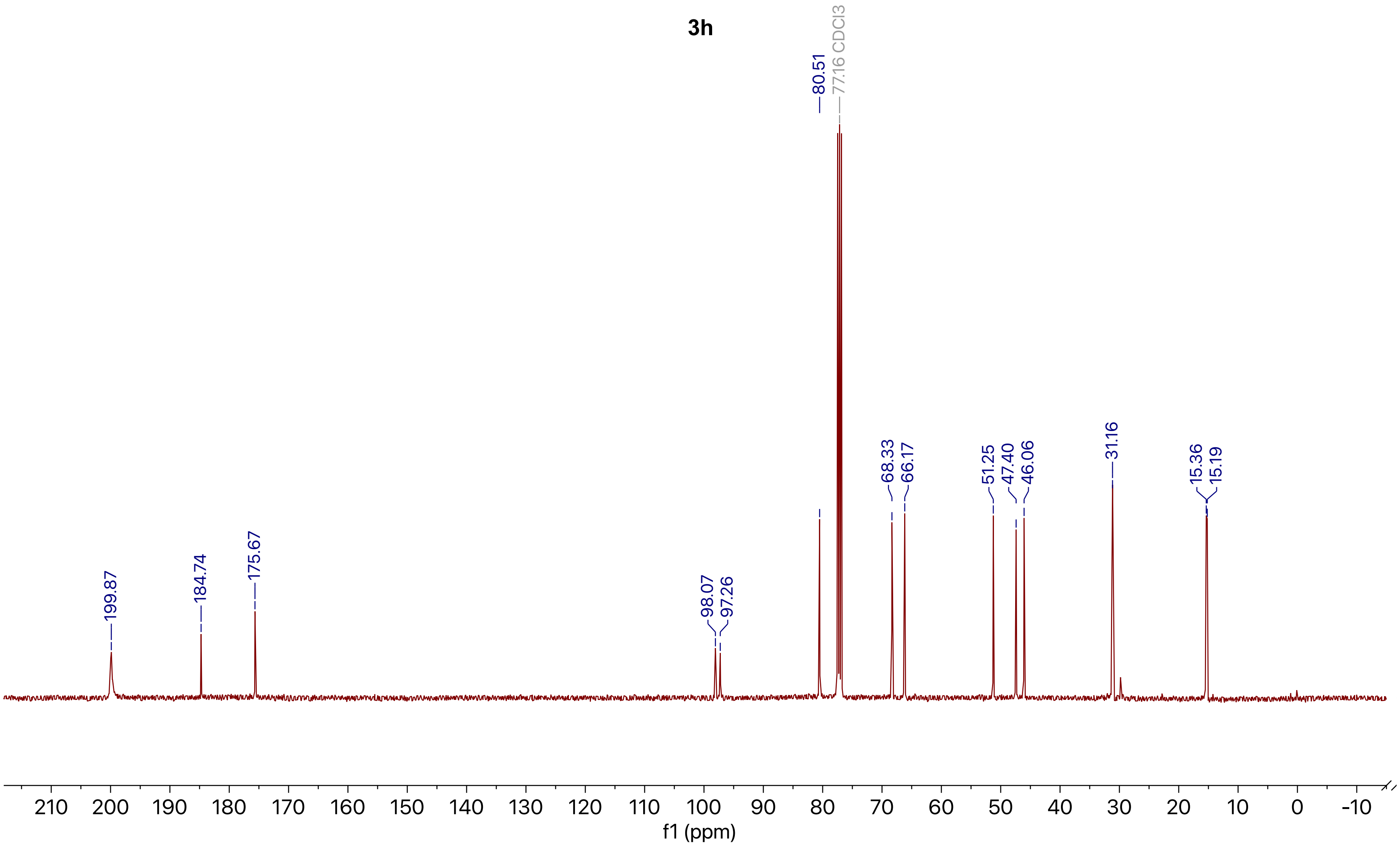

2D ^{1}H–^{1}H COSY (400 MHz, $CDCl_3$)

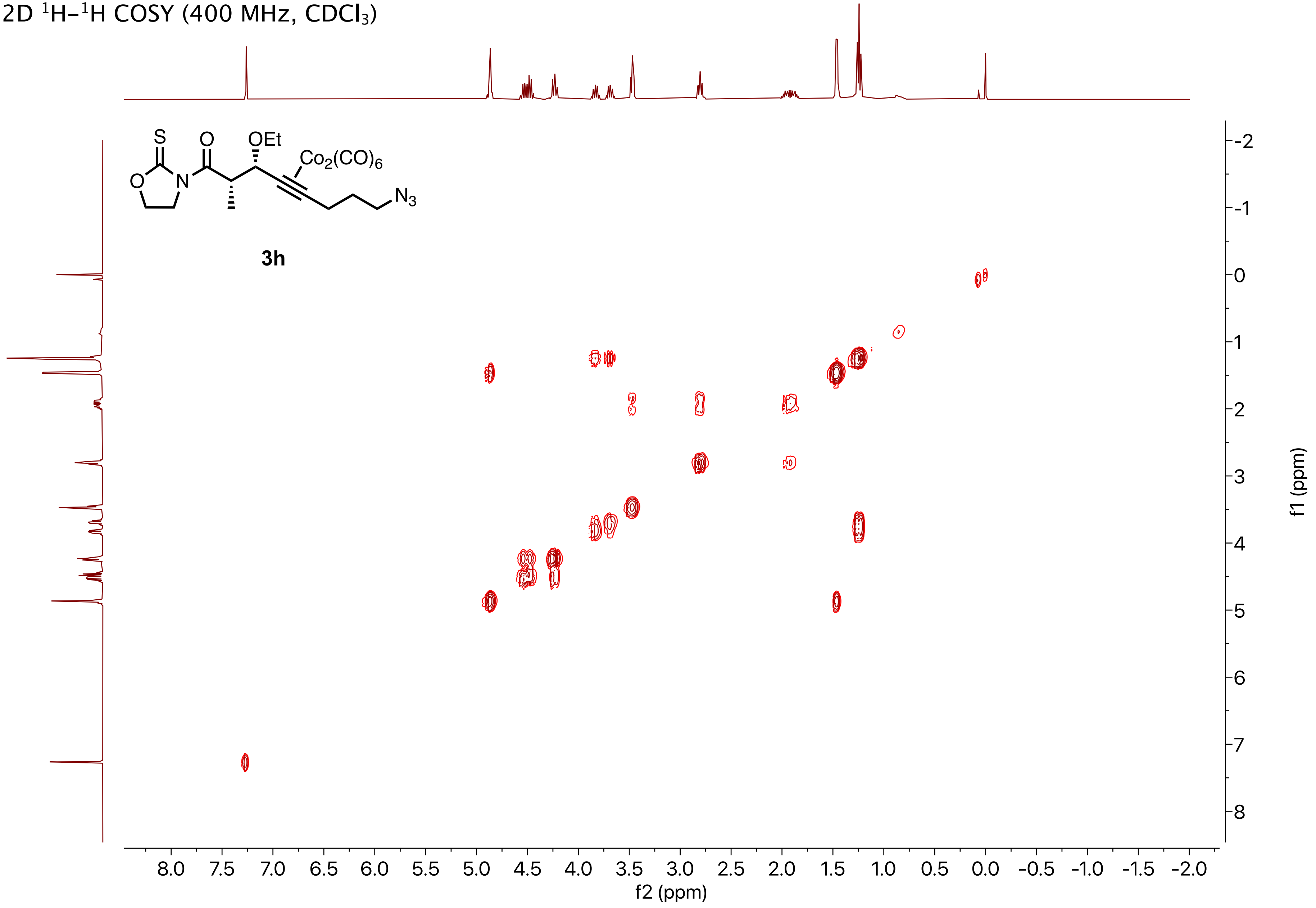

2D ^{1}H-^{13}C HSQC (400 MHz, $CDCl_3$)

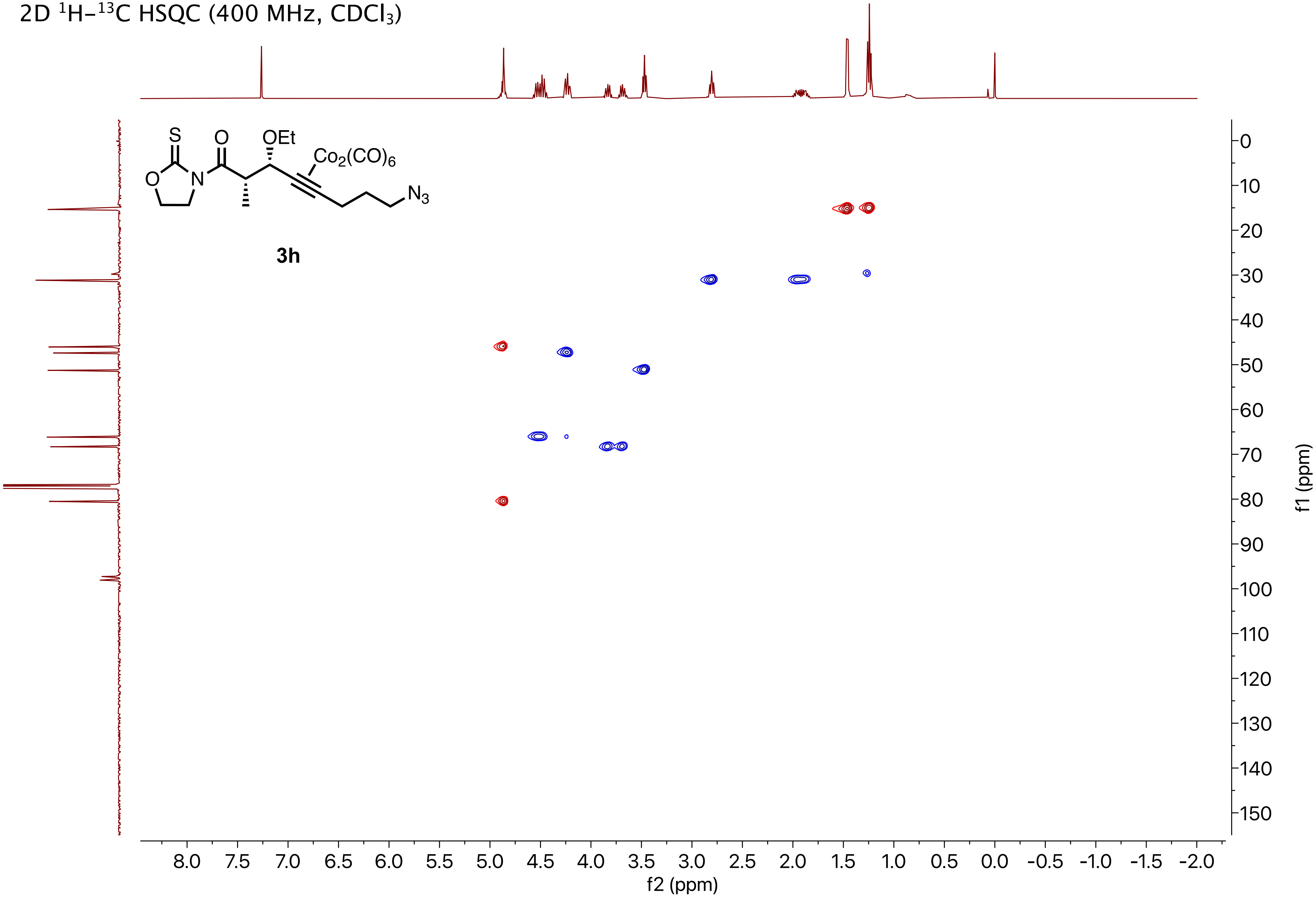

^1H NMR (400 MHz, $CDCl_3$)

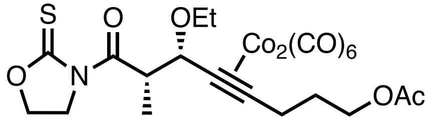

3i

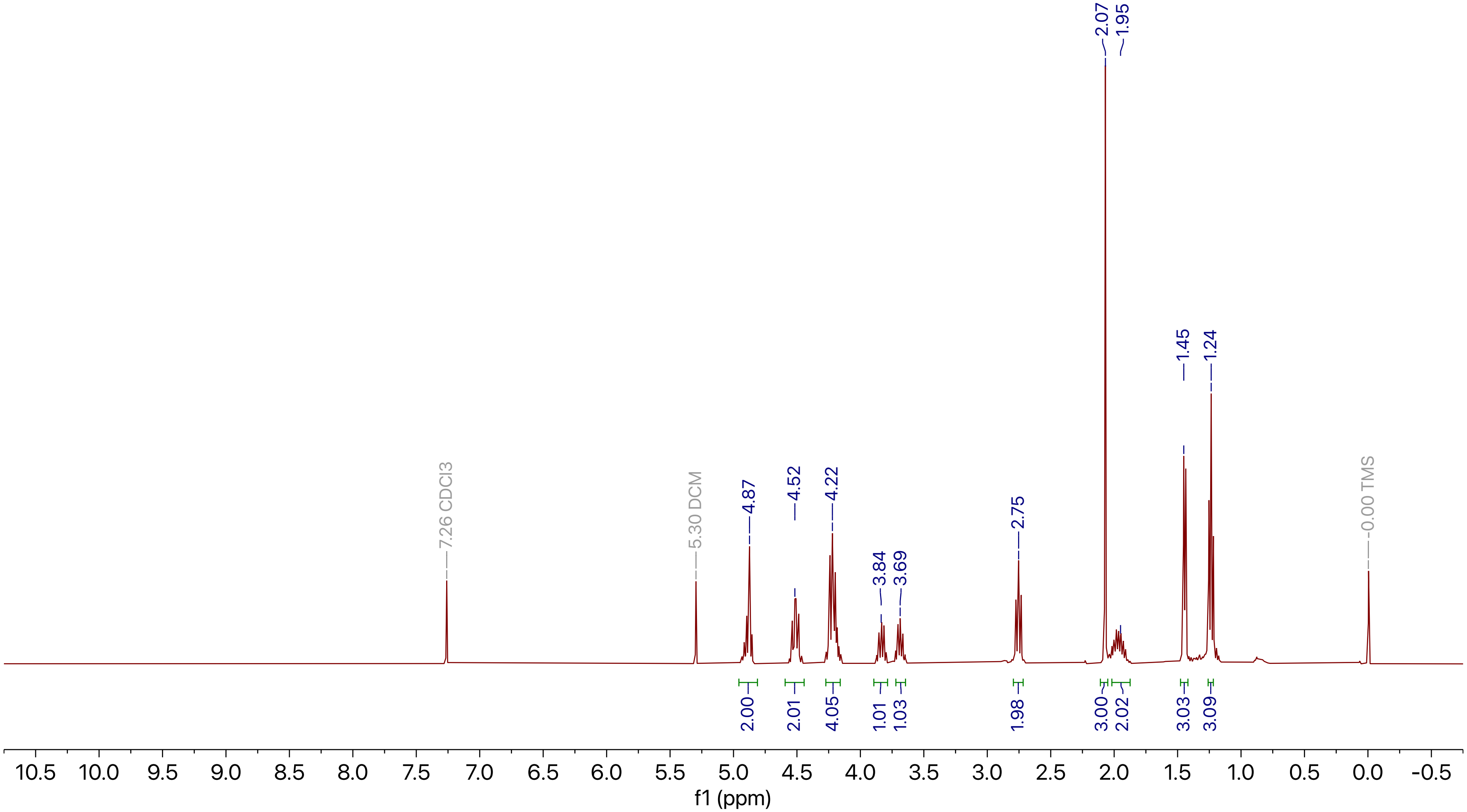

$^{13}C\{^{1}H\}$ NMR (101 MHz, $CDCl_3$)

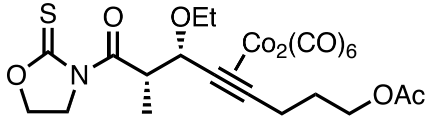

3i

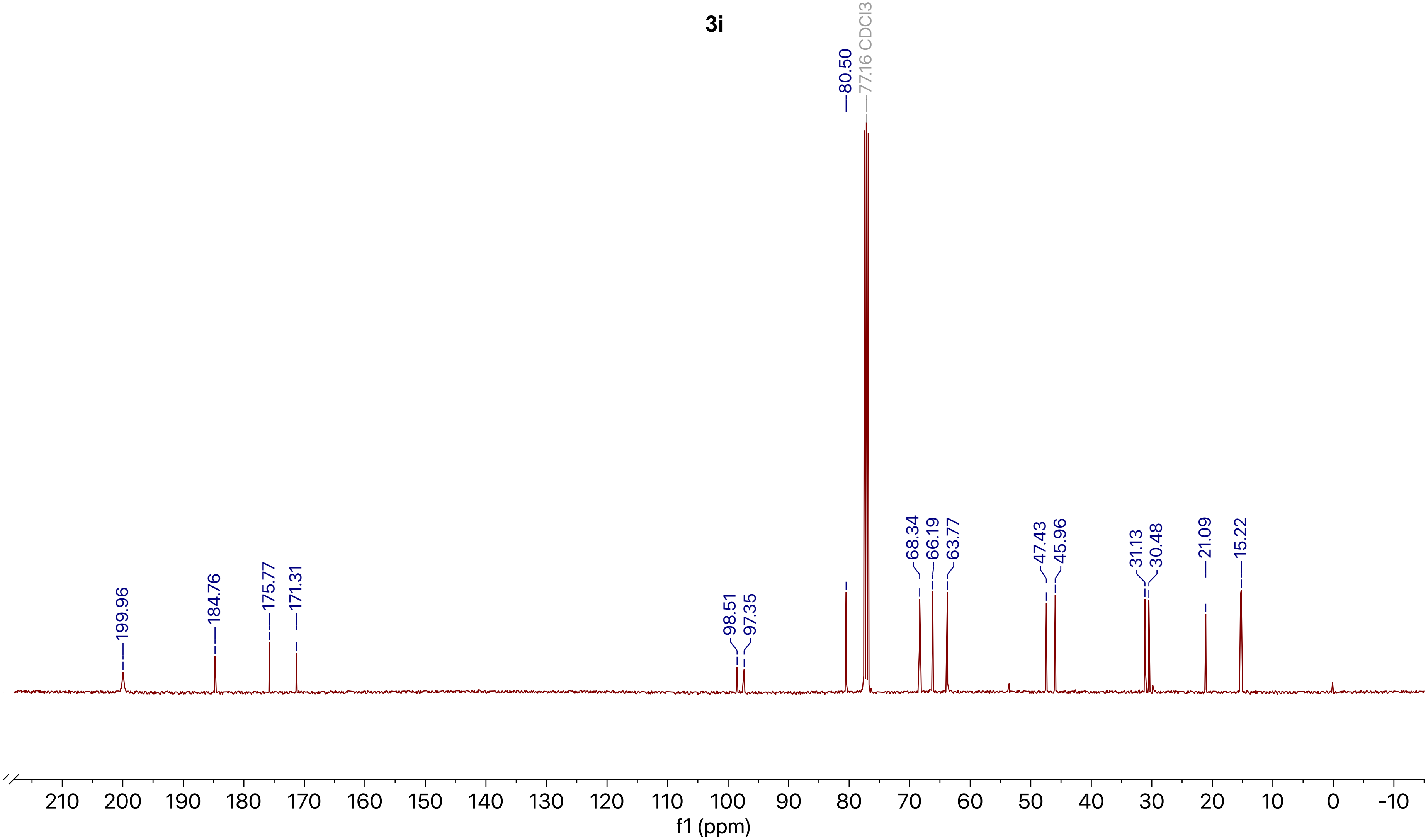

2D ^1H–^1H COSY (400 MHz, $CDCl_3$)

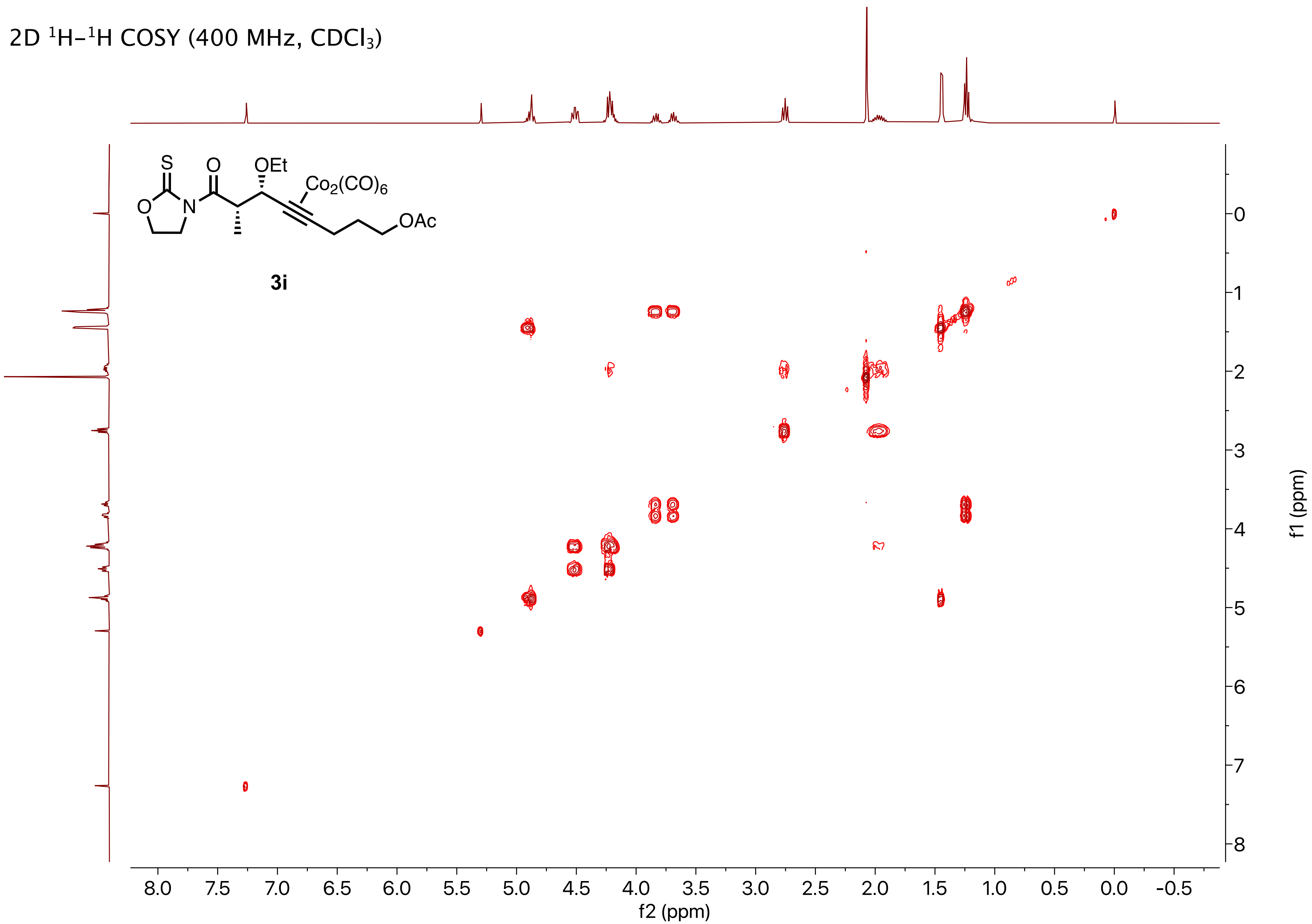

2D ^{1}H–^{13}C HSQC (400 MHz, $CDCl_3$)

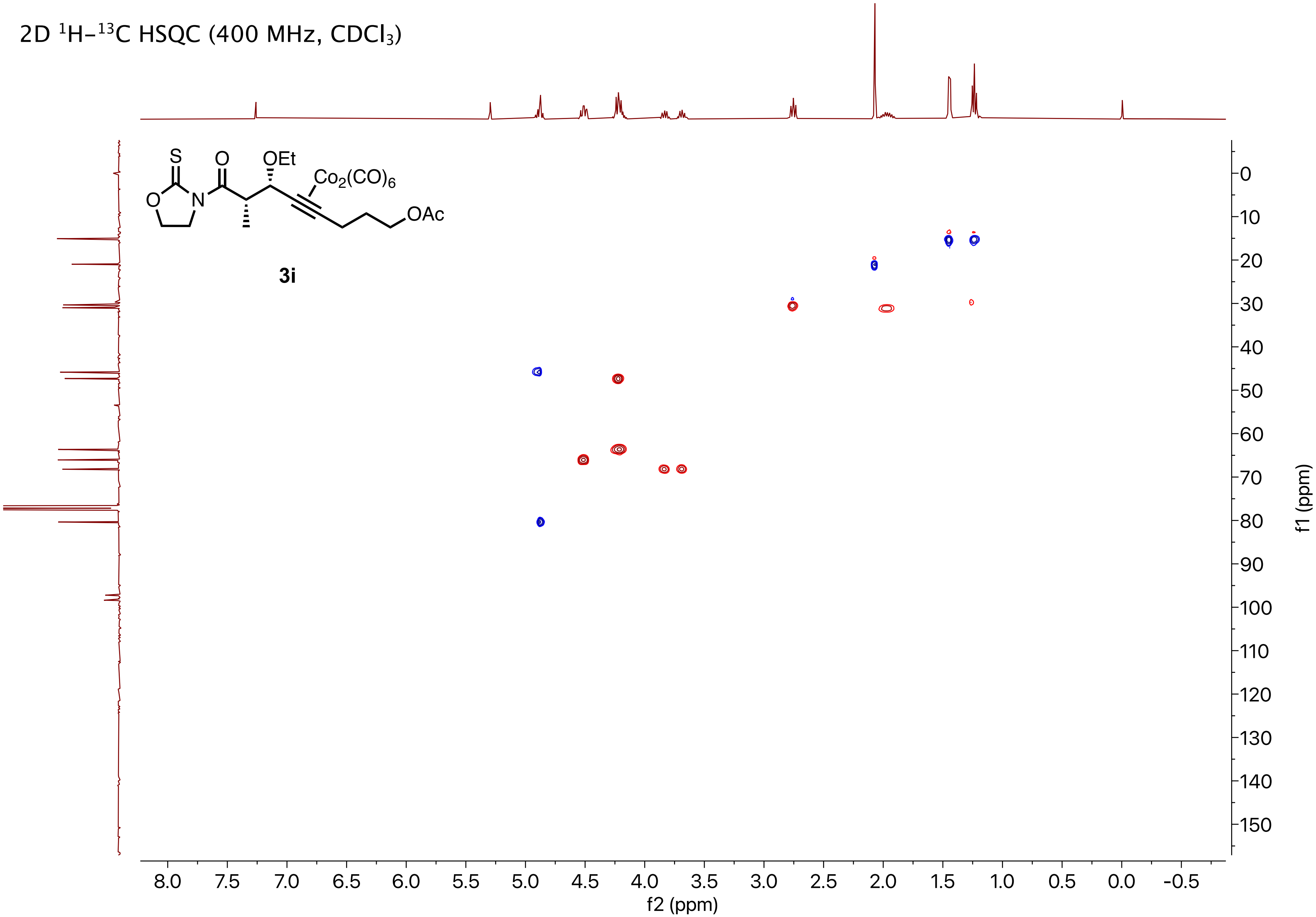

^{1}H NMR (500 MHz, $CDCl_3$)

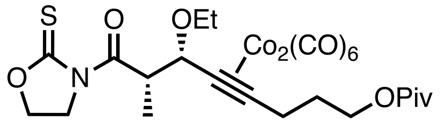

3j

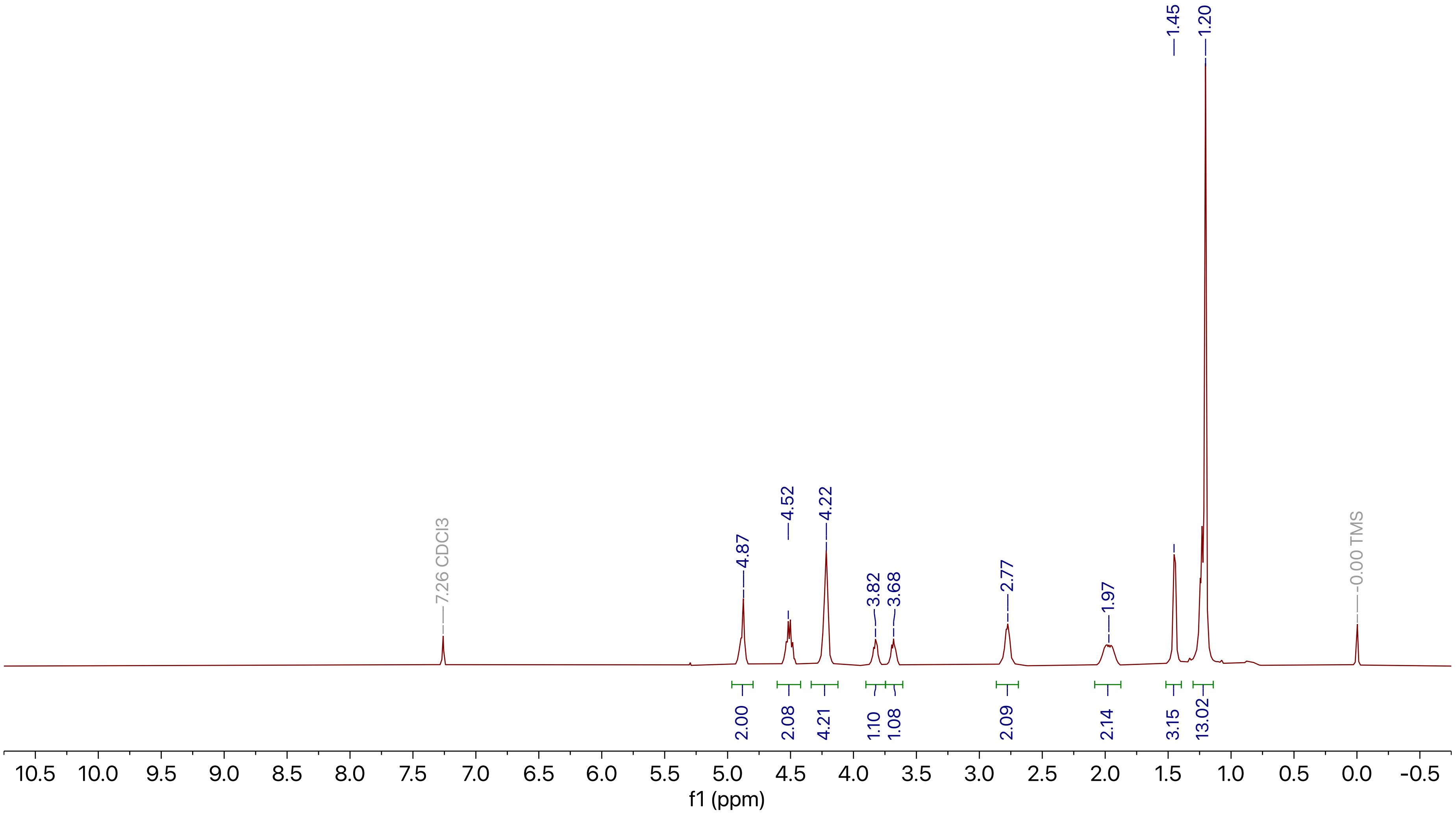

$^{13}C\{^{1}H\}$ NMR (126 MHz, $CDCl_3$)

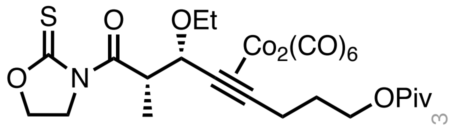

3j

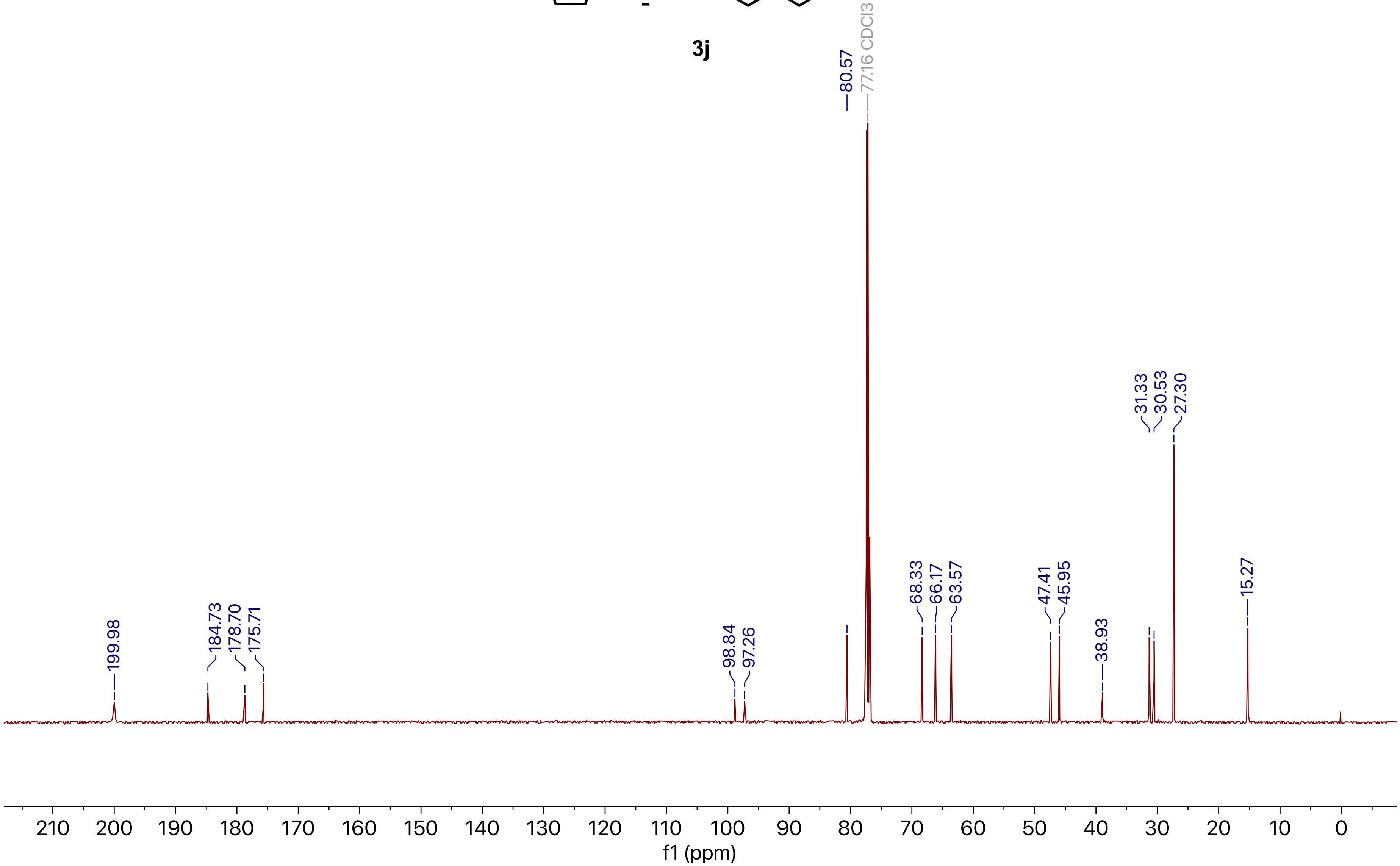

2D ^{1}H–^{1}H COSY (500 MHz, $CDCl_3$)

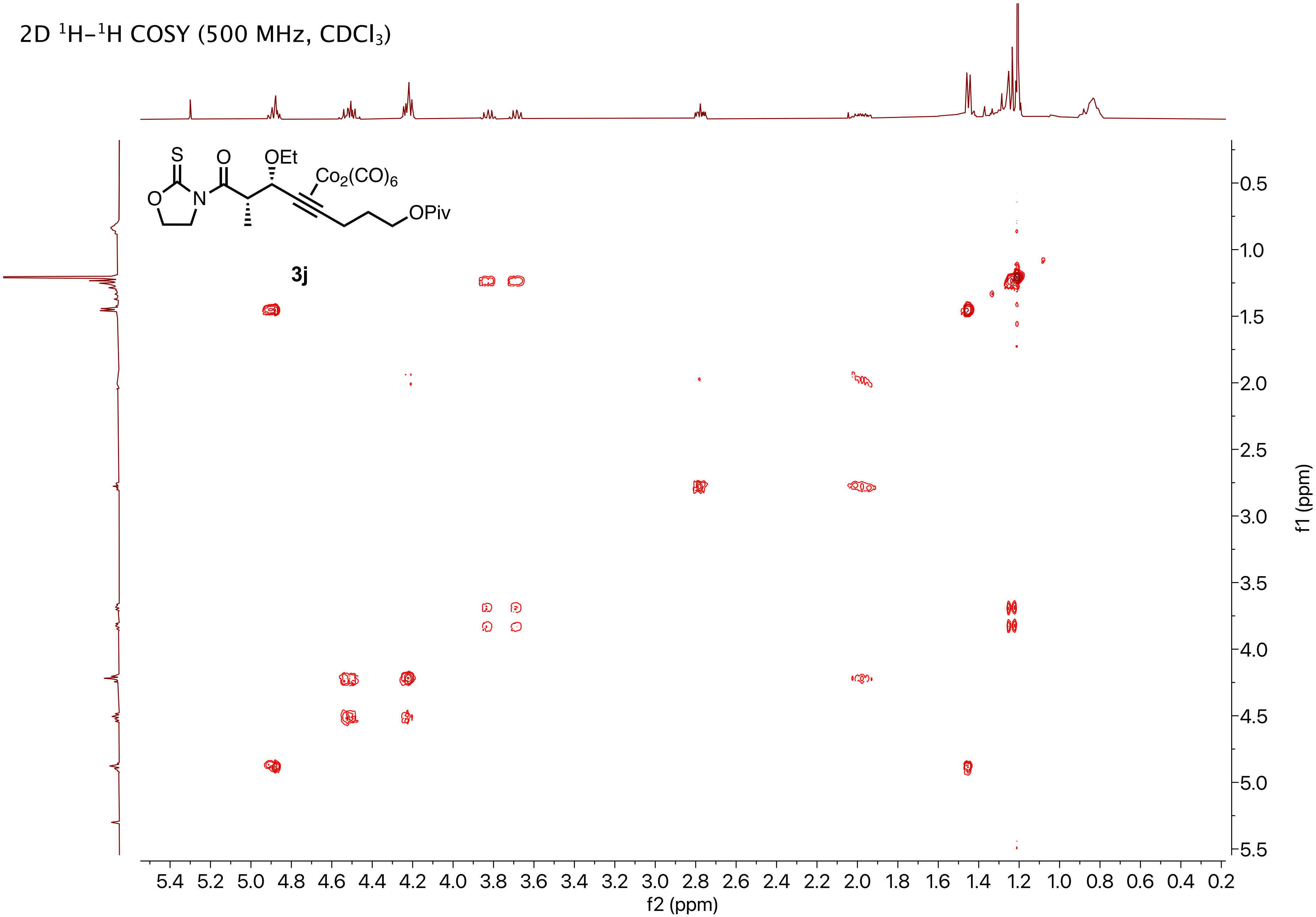

2D ^{1}H–^{13}C HSQC (500 MHz, $CDCl_3$)

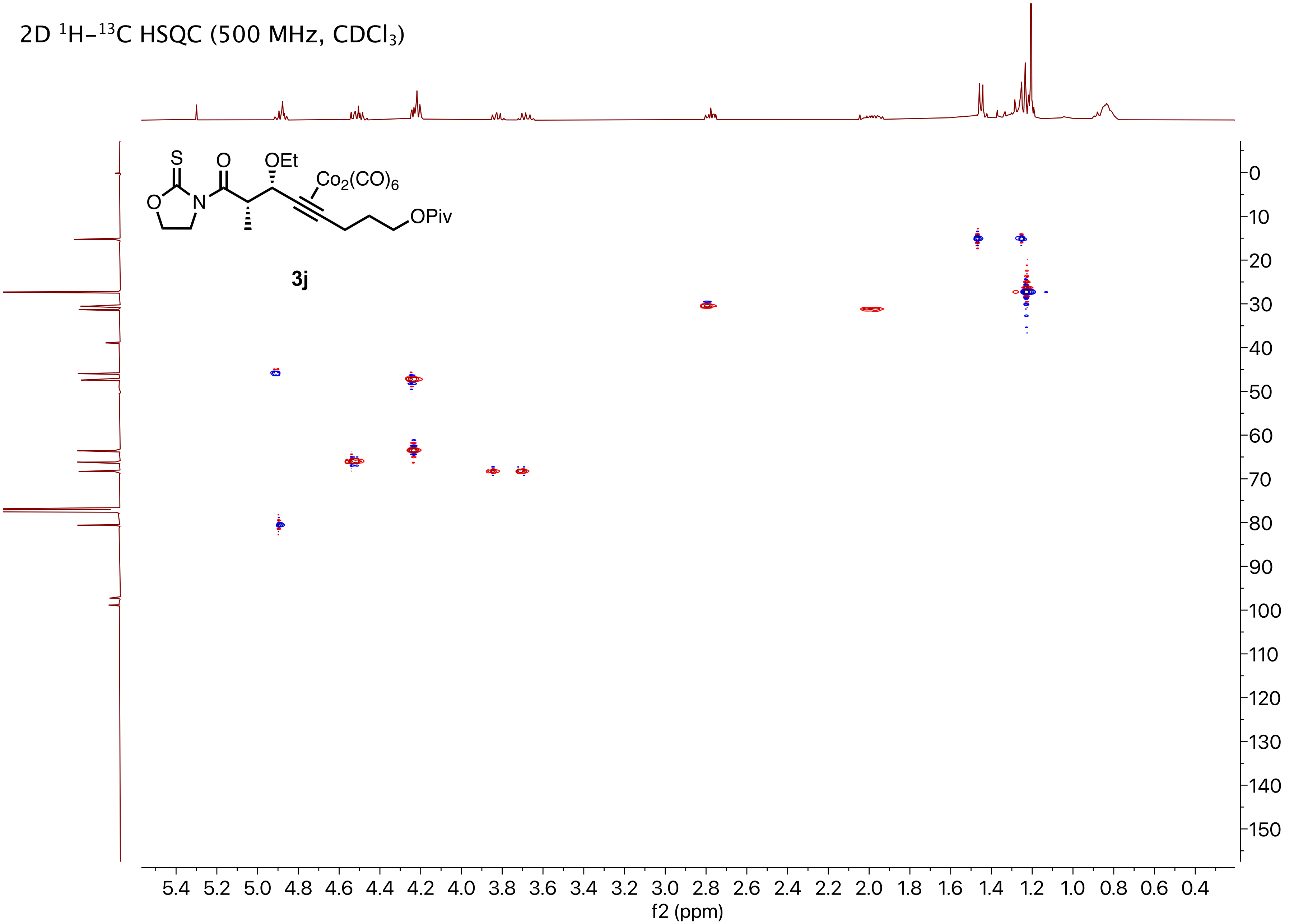

^1H NMR (500 MHz, $CDCl_3$)

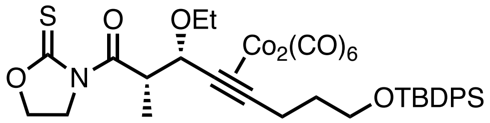

3k

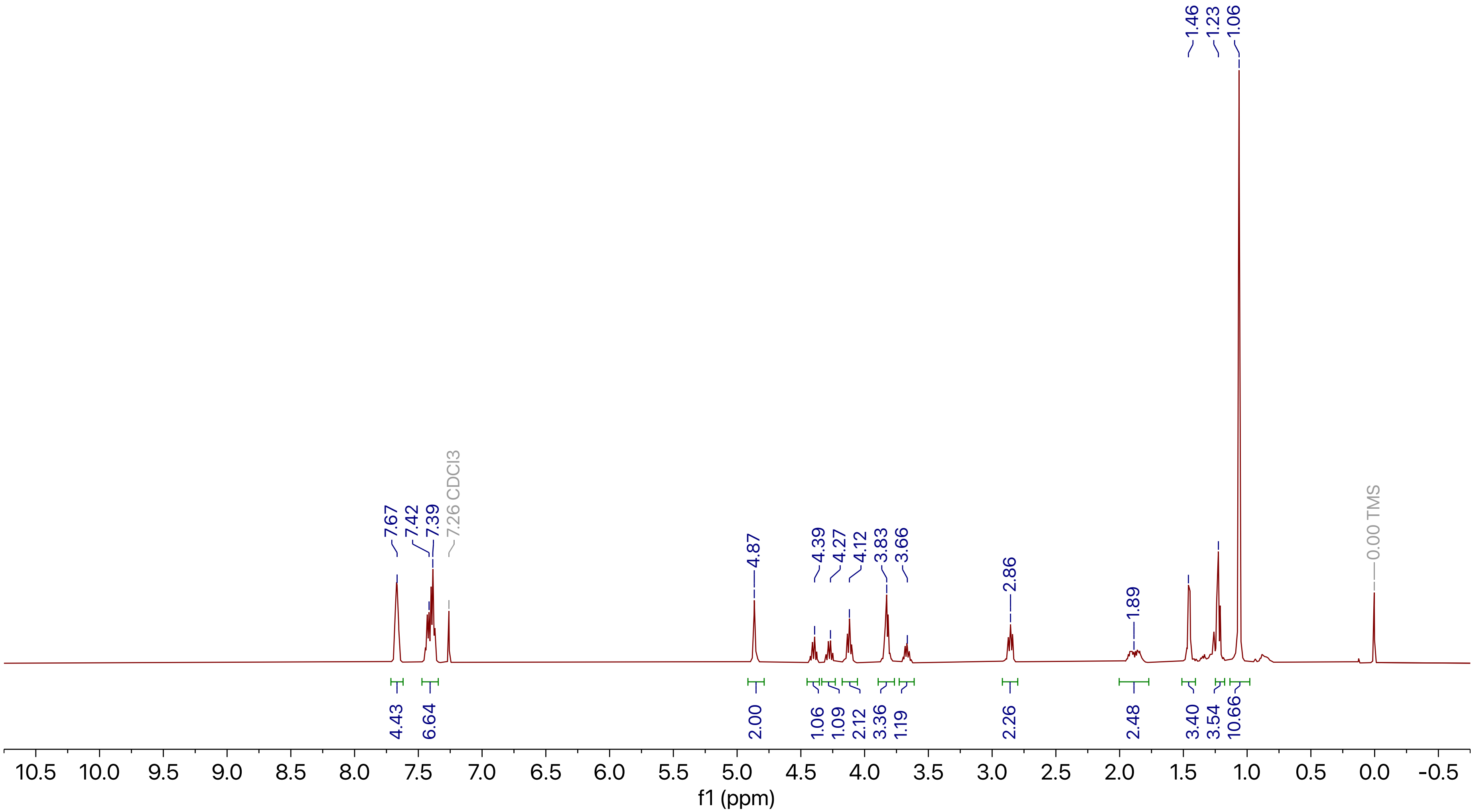

$^{13}C\{^{1}H\}$ NMR (126 MHz, $CDCl_3$)

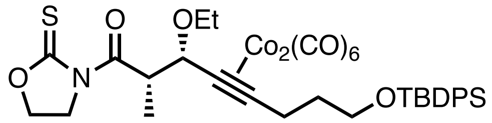

3k

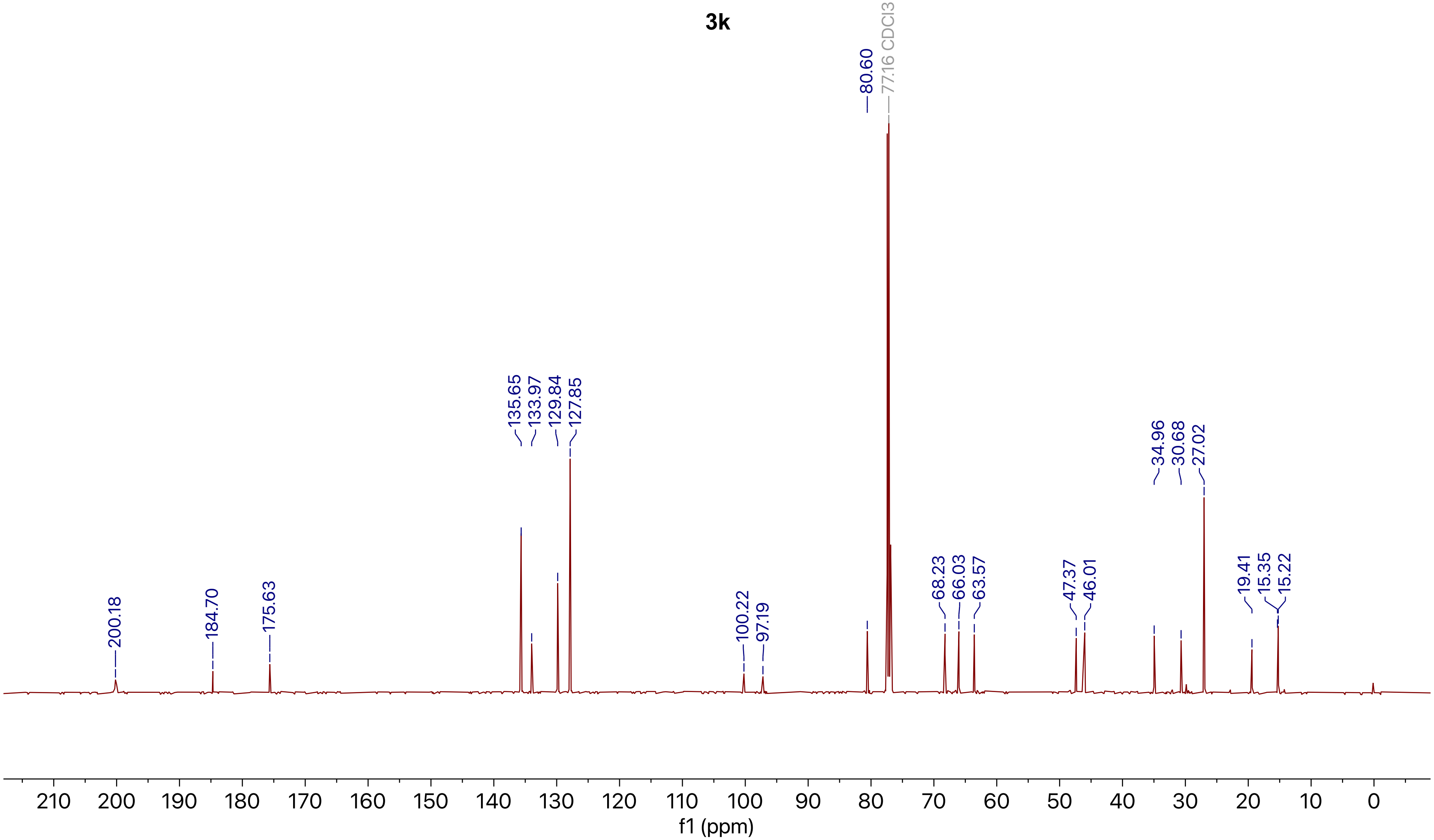

2D ^{1}H–^{1}H COSY (500 MHz, $CDCl_3$)

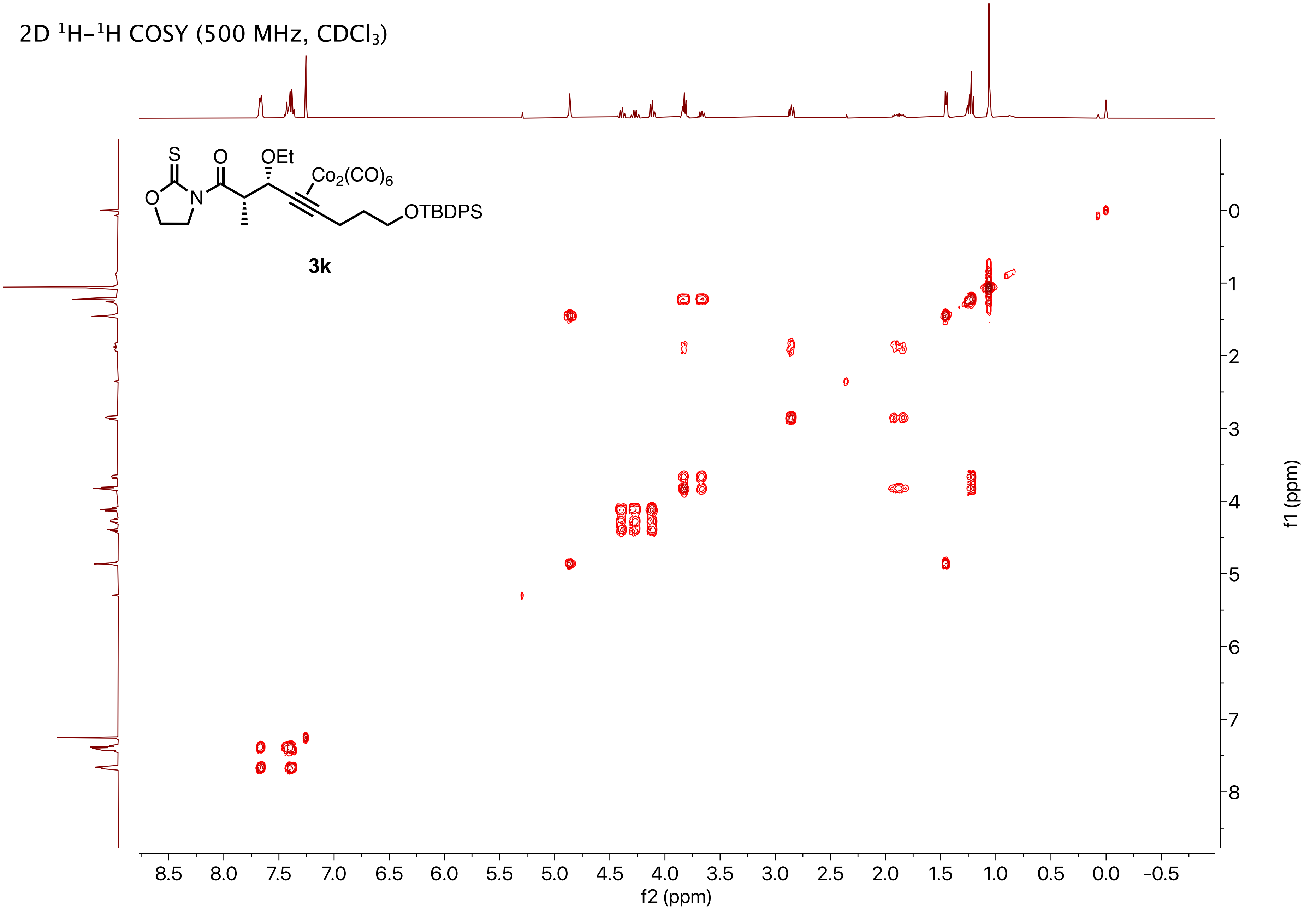

2D ^{1}H–^{13}C HSQC (500 MHz, $CDCl_3$)

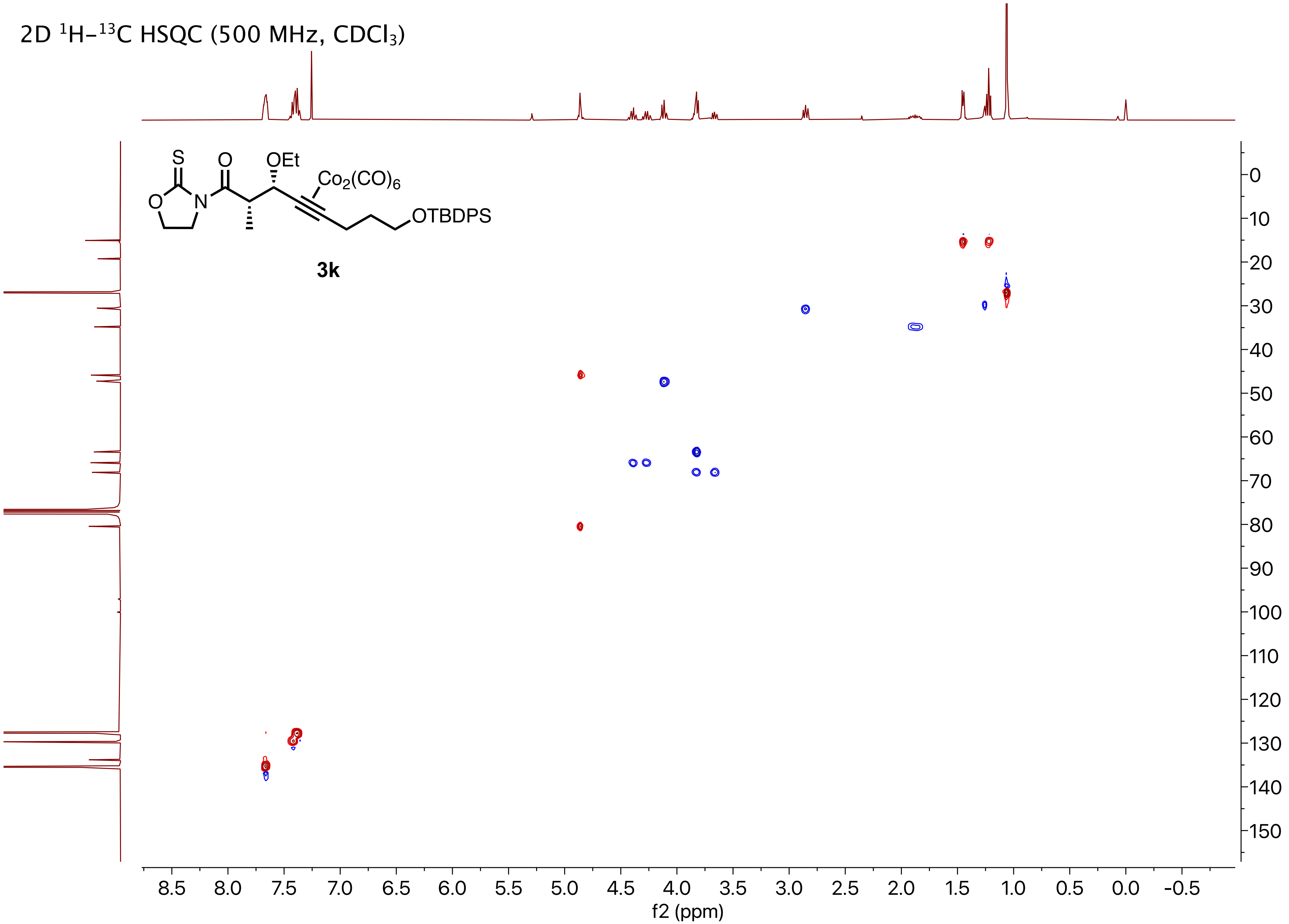

^{1}H NMR (500 MHz, $CDCl_3$)

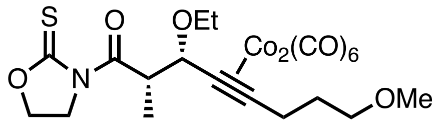

3l

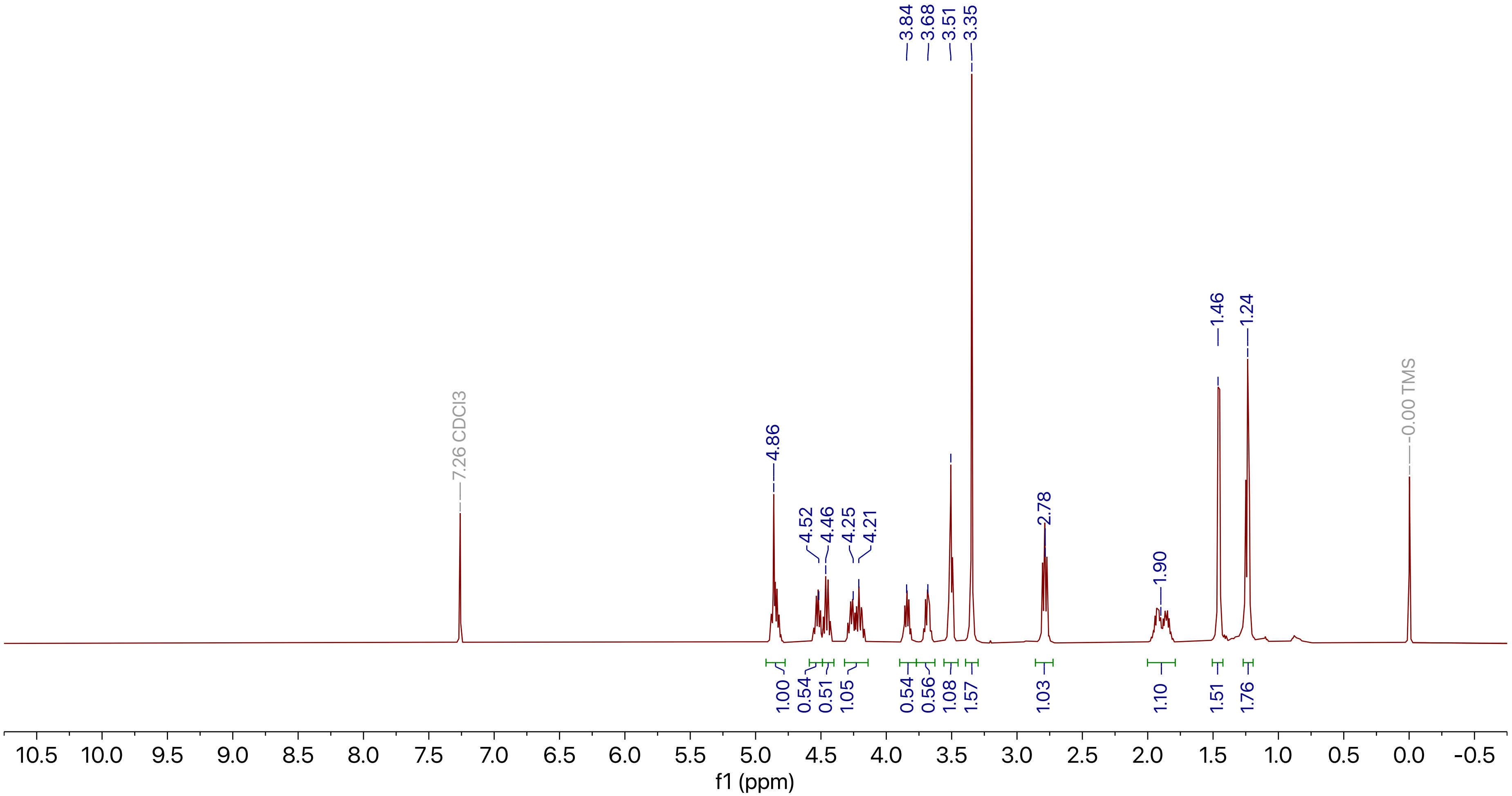

$^{13}C\{^{1}H\}$ NMR (126 MHz, $CDCl_3$)

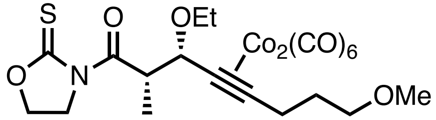

3l

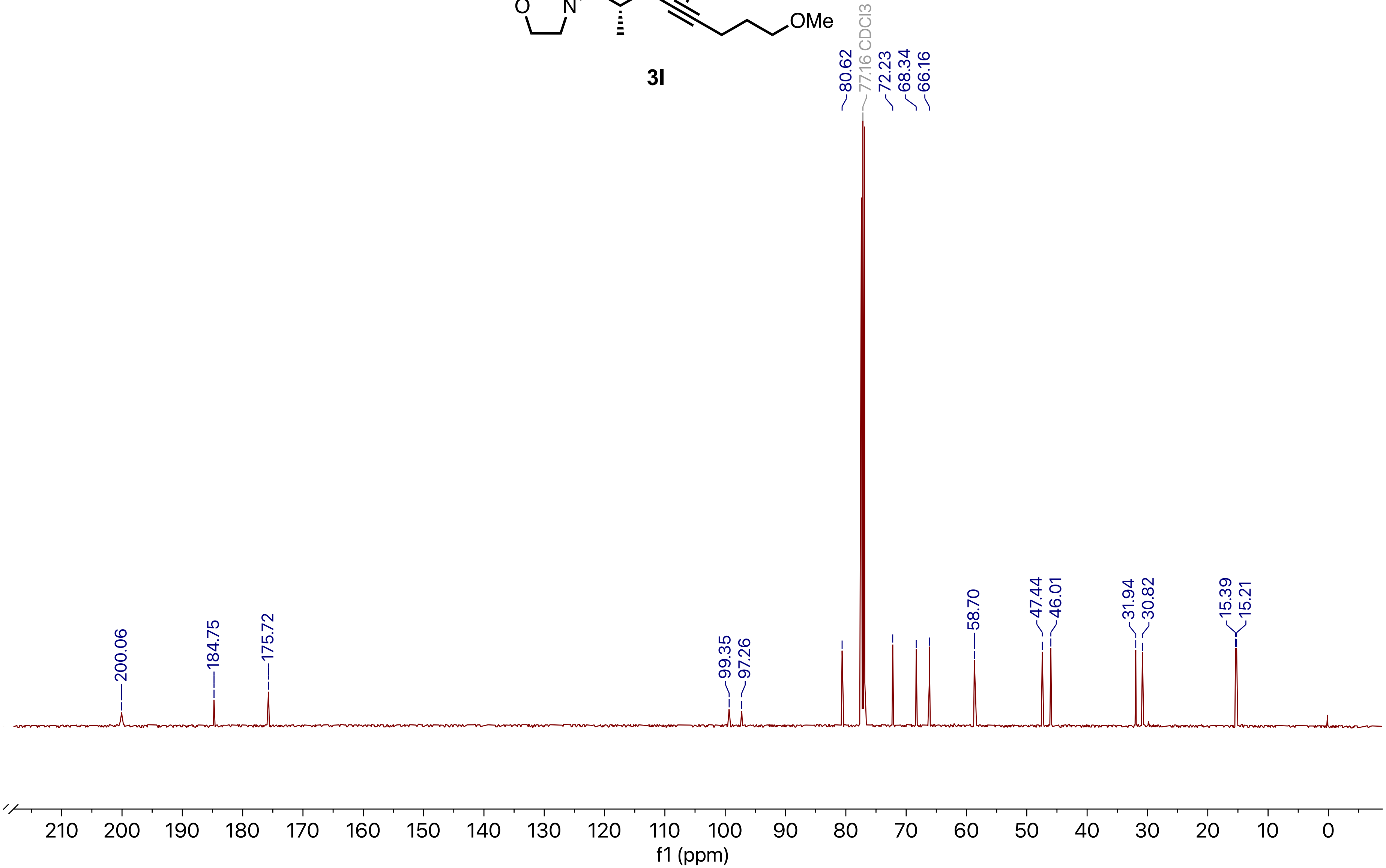

2D ^1H–^1H COSY (500 MHz, $CDCl_3$)

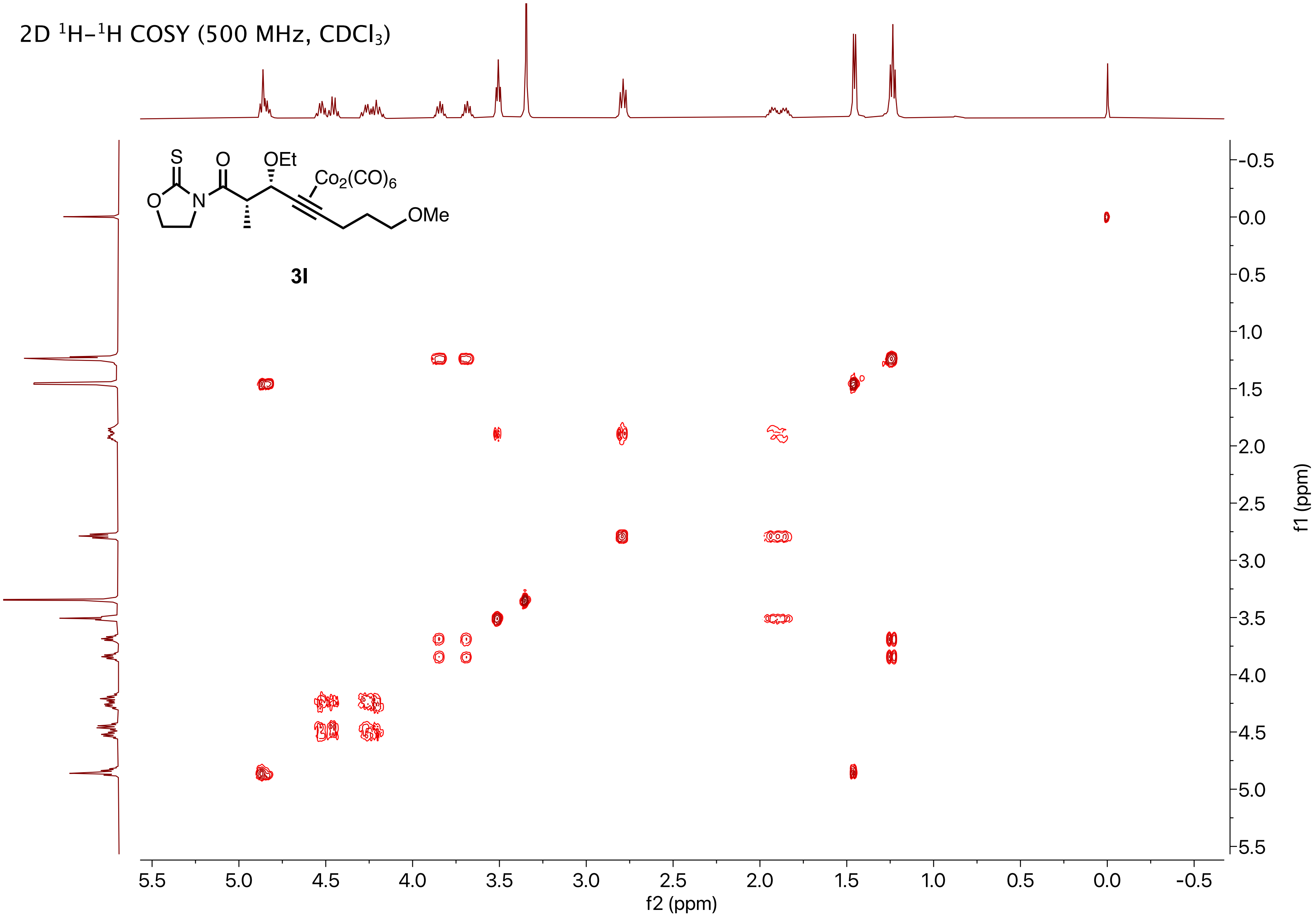

2D ^{1}H–^{13}C HSQC (500 MHz, $CDCl_3$)

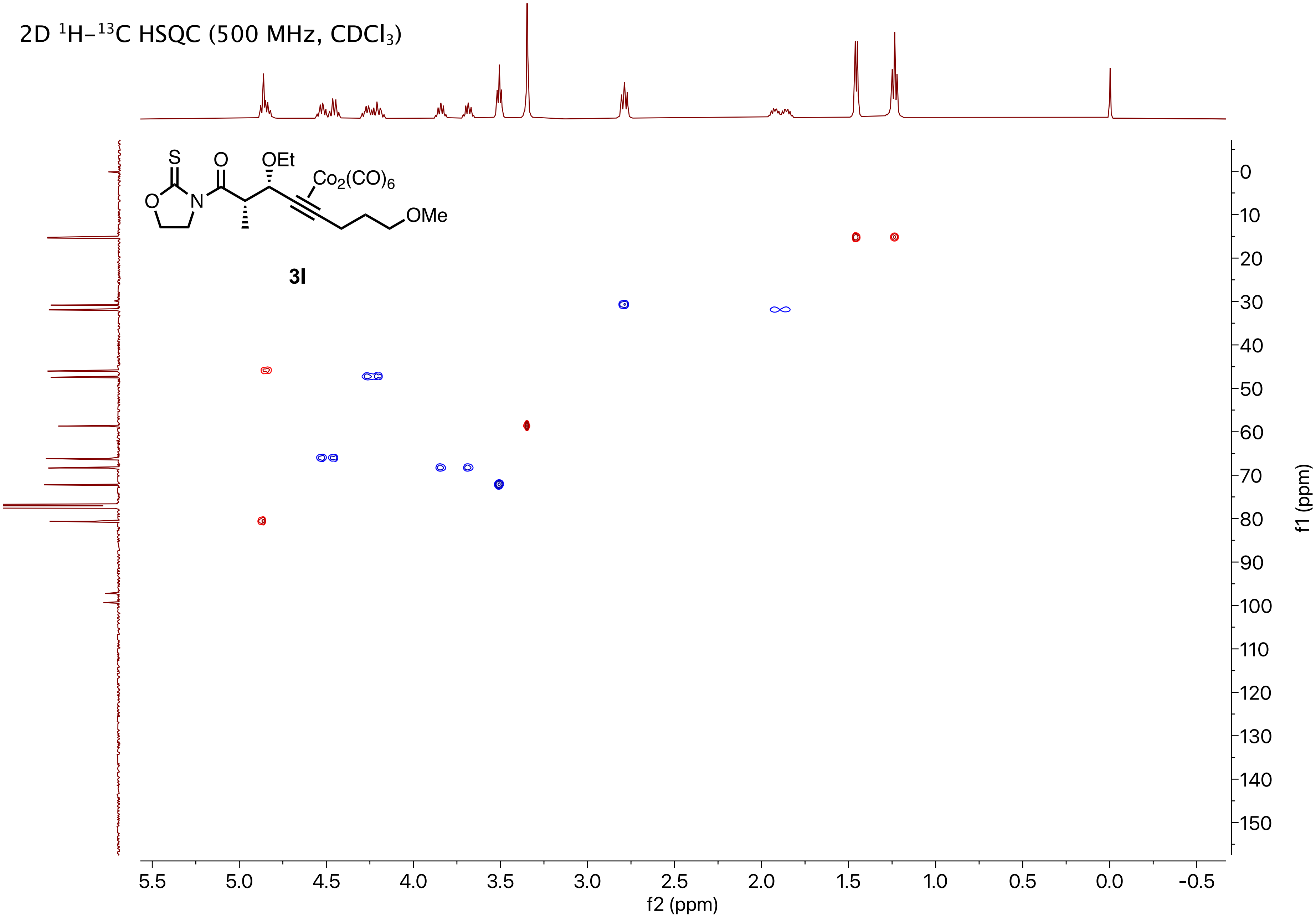

^1H NMR (400 MHz, $CDCl_3$)

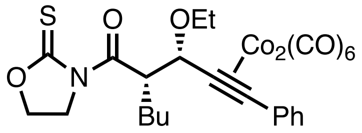

3m

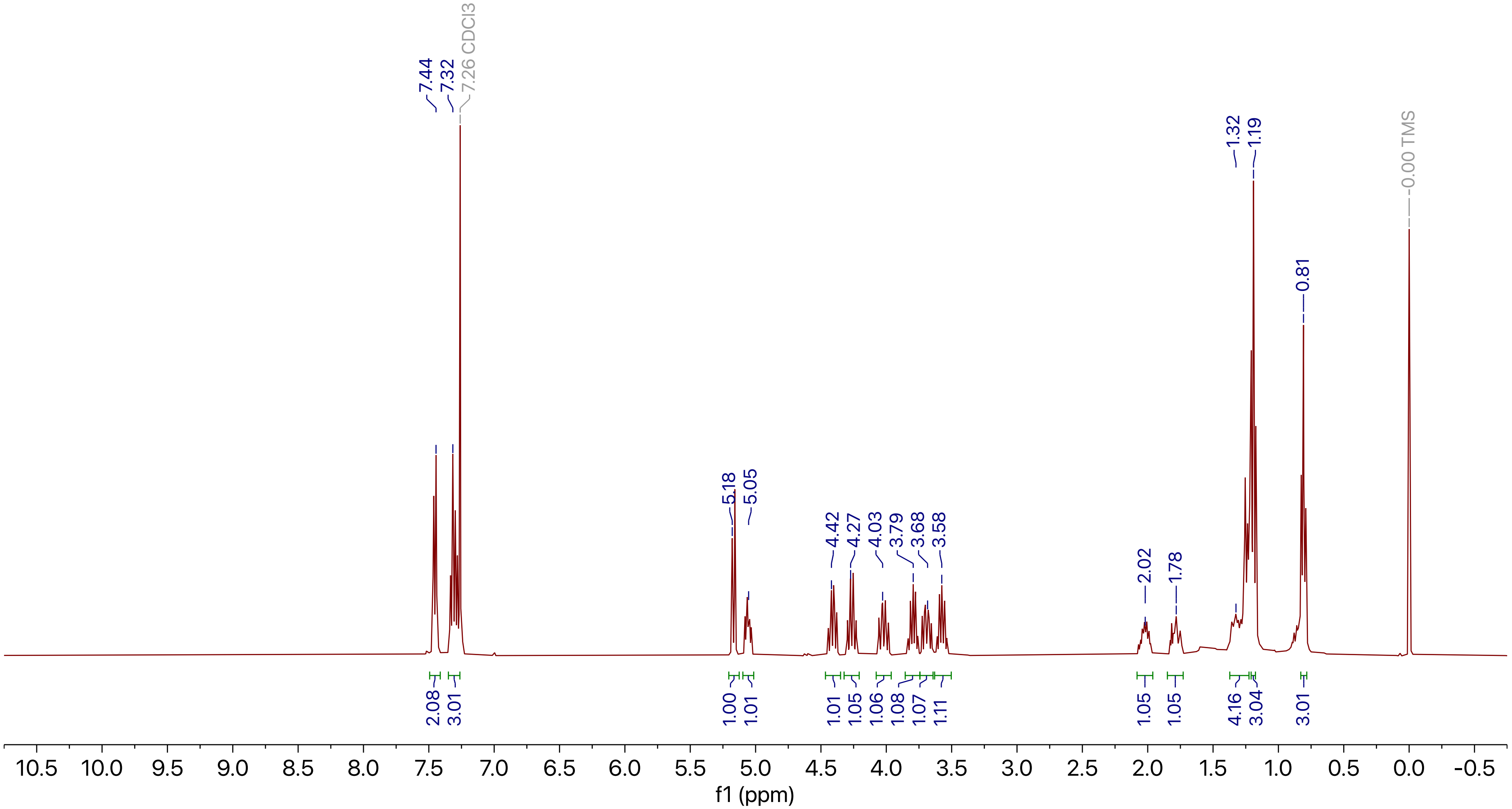

$^{13}C\{^{1}H\}$ NMR (101 MHz, $CDCl_3$)

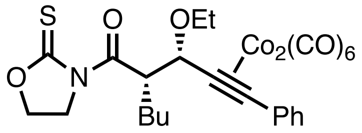

3m

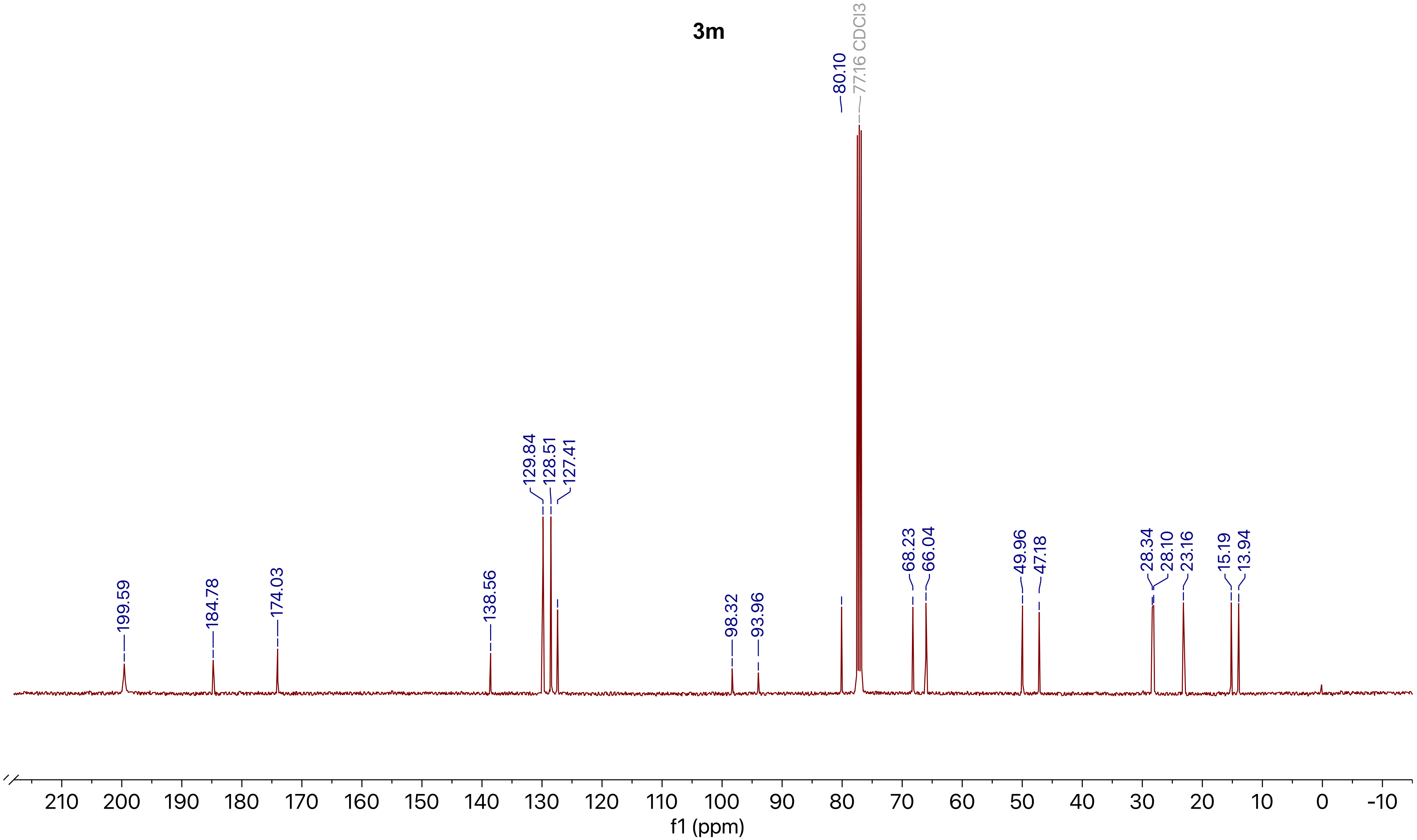

2D ^{1}H–^{1}H COSY (400 MHz, $CDCl_3$)

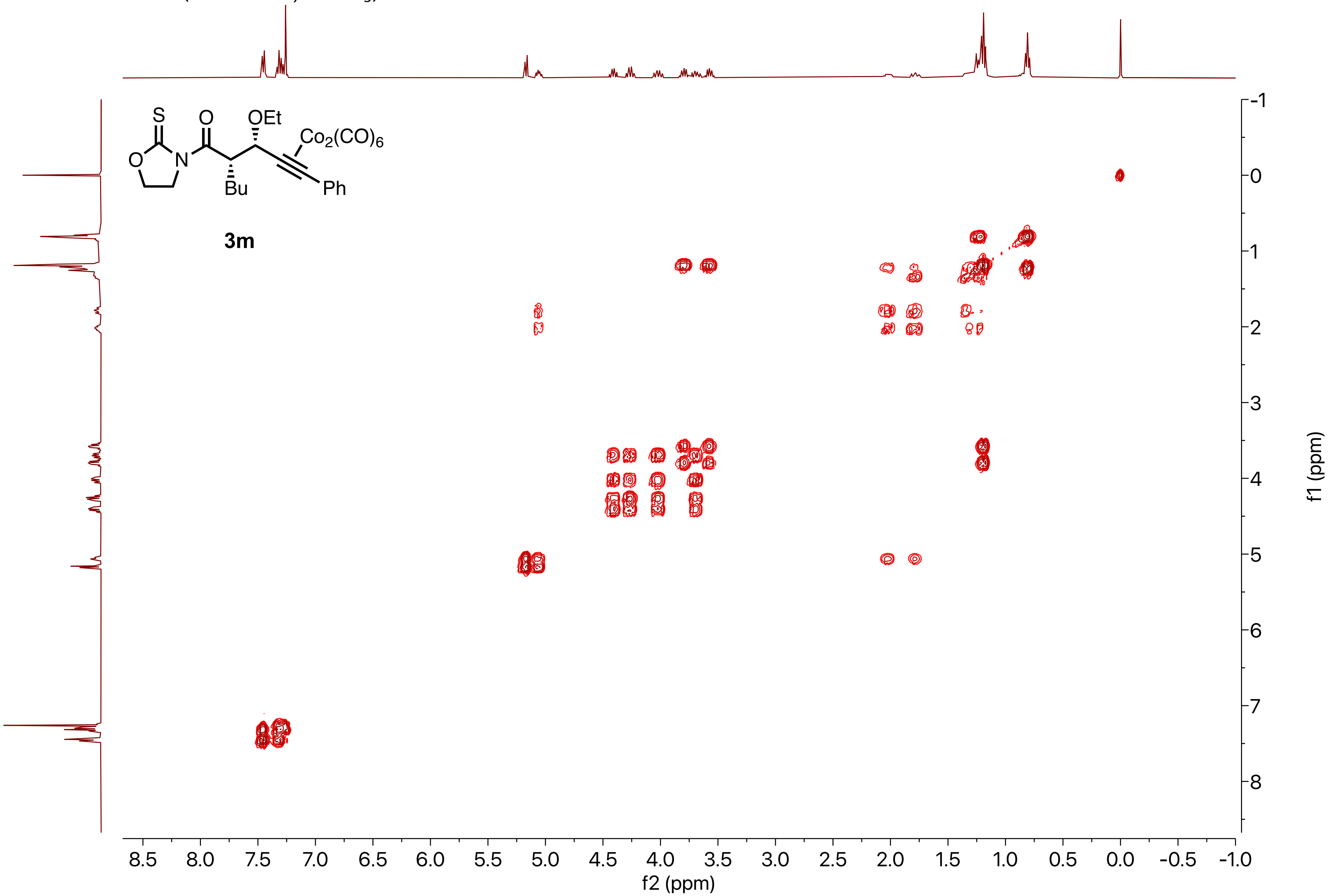

2D $^{1}H-^{13}C$ HSQC (400 MHz, $CDCl_3$)

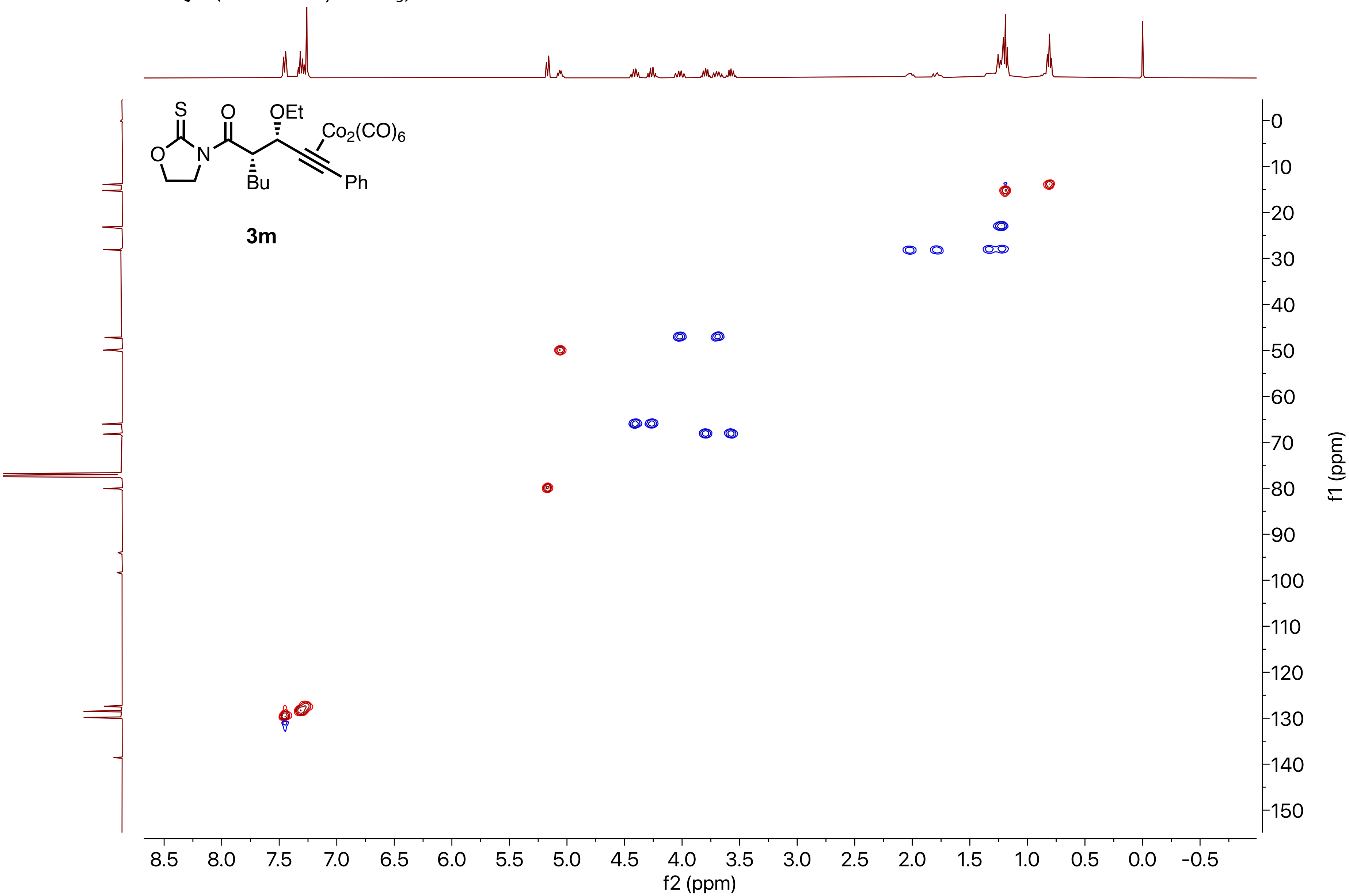

3m

^{1}H NMR (400 MHz, $CDCl_3$)

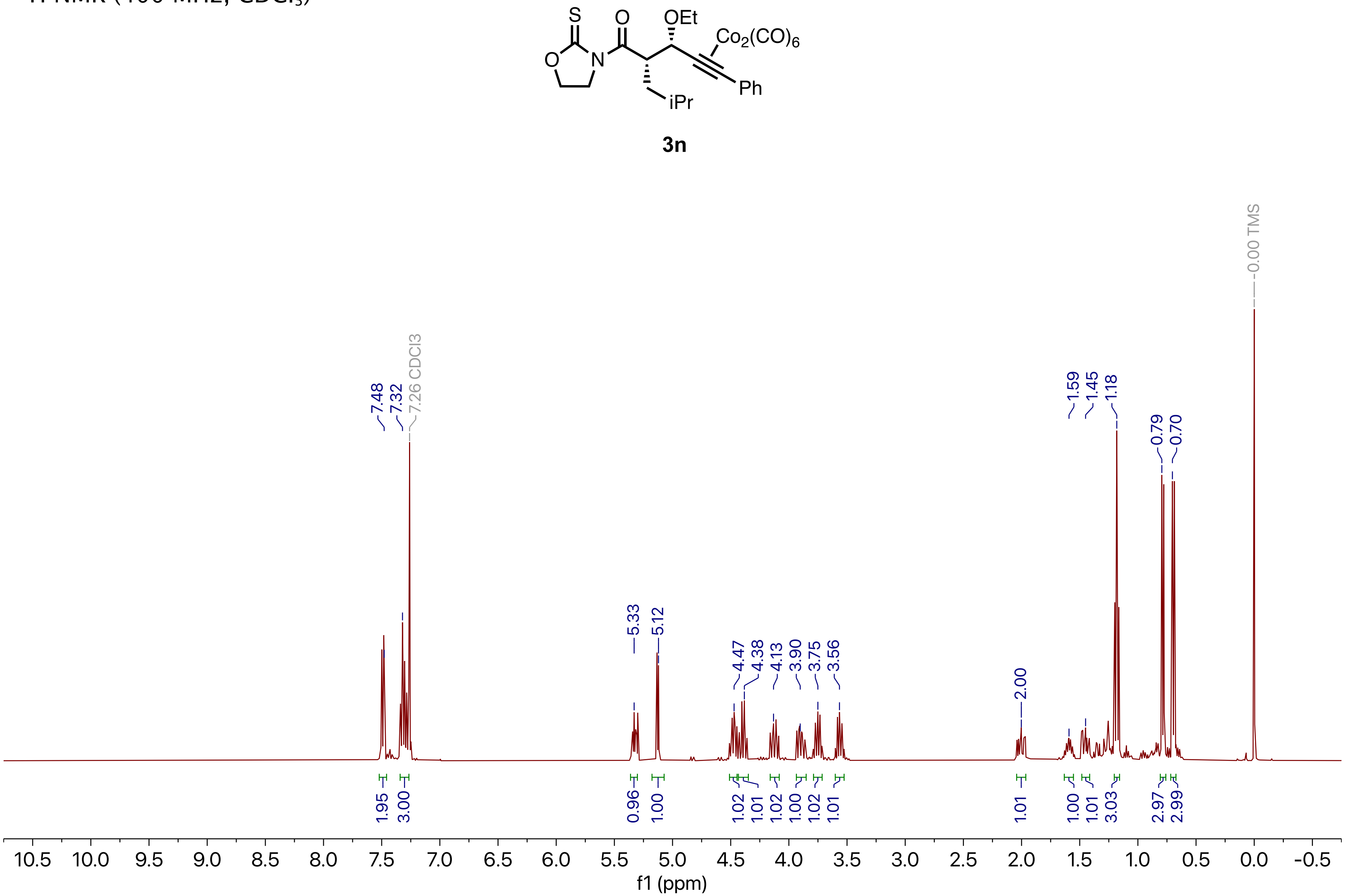

3n

$^{13}C\{^{1}H\}$ NMR (101 MHz, $CDCl_3$)

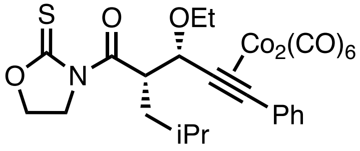

3n

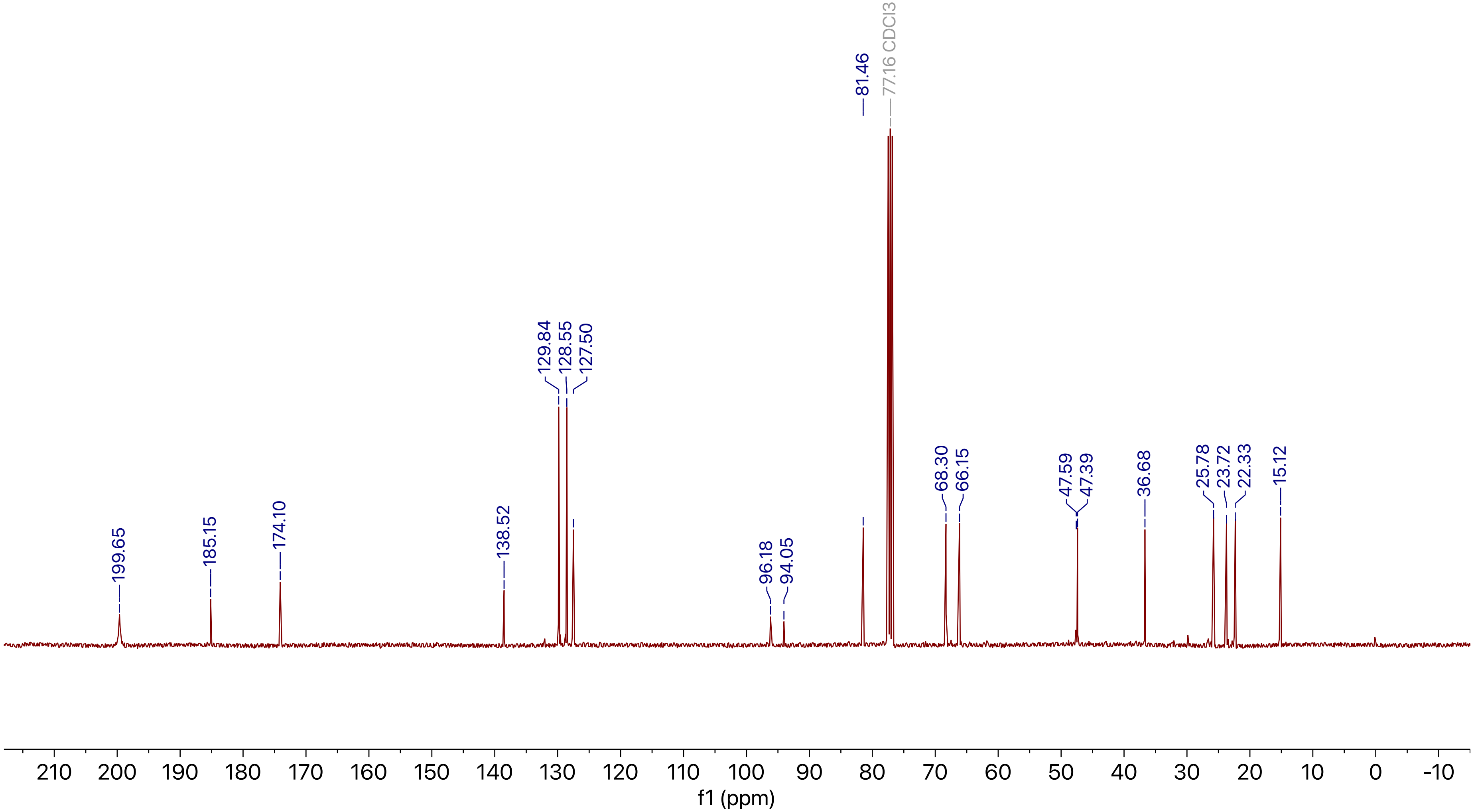

2D ^1H–^1H COSY (400 MHz, $CDCl_3$)

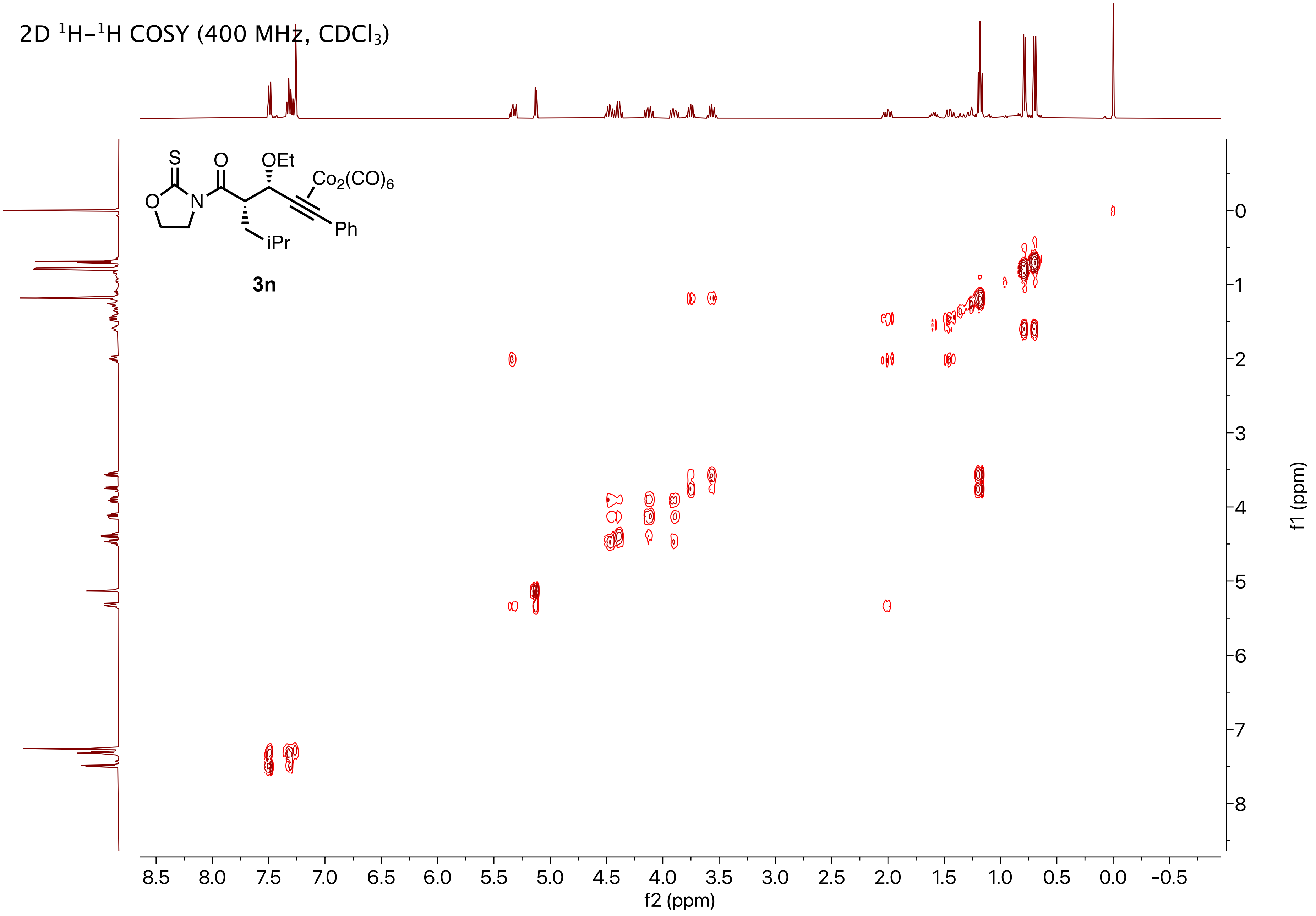

2D ^{1}H–^{13}C HSQC (400 MHz, $CDCl_3$)

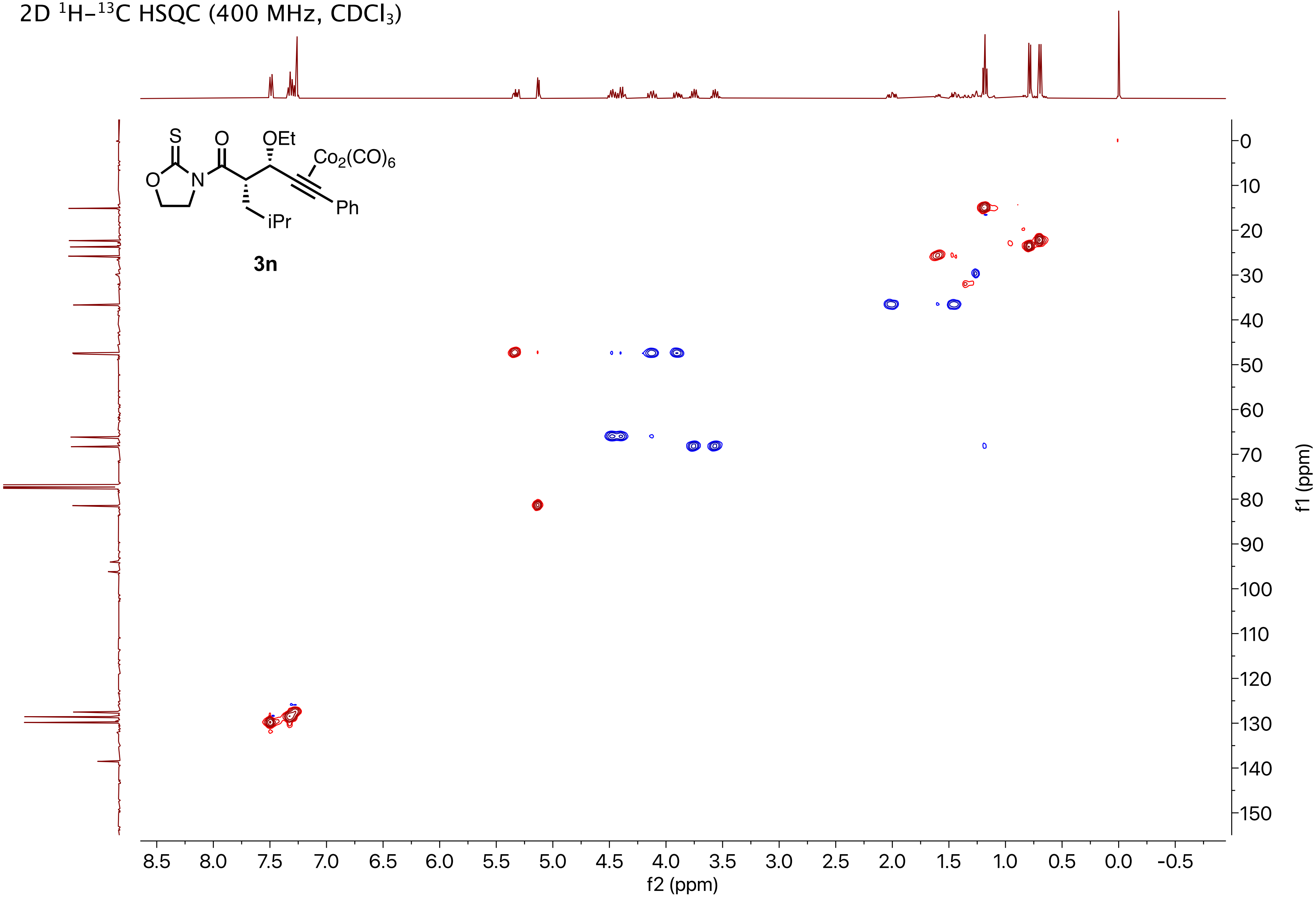

^{1}H NMR (400 MHz, $CDCl_3$)

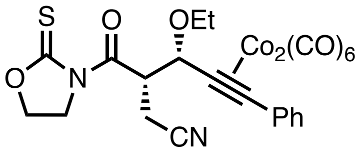

3o

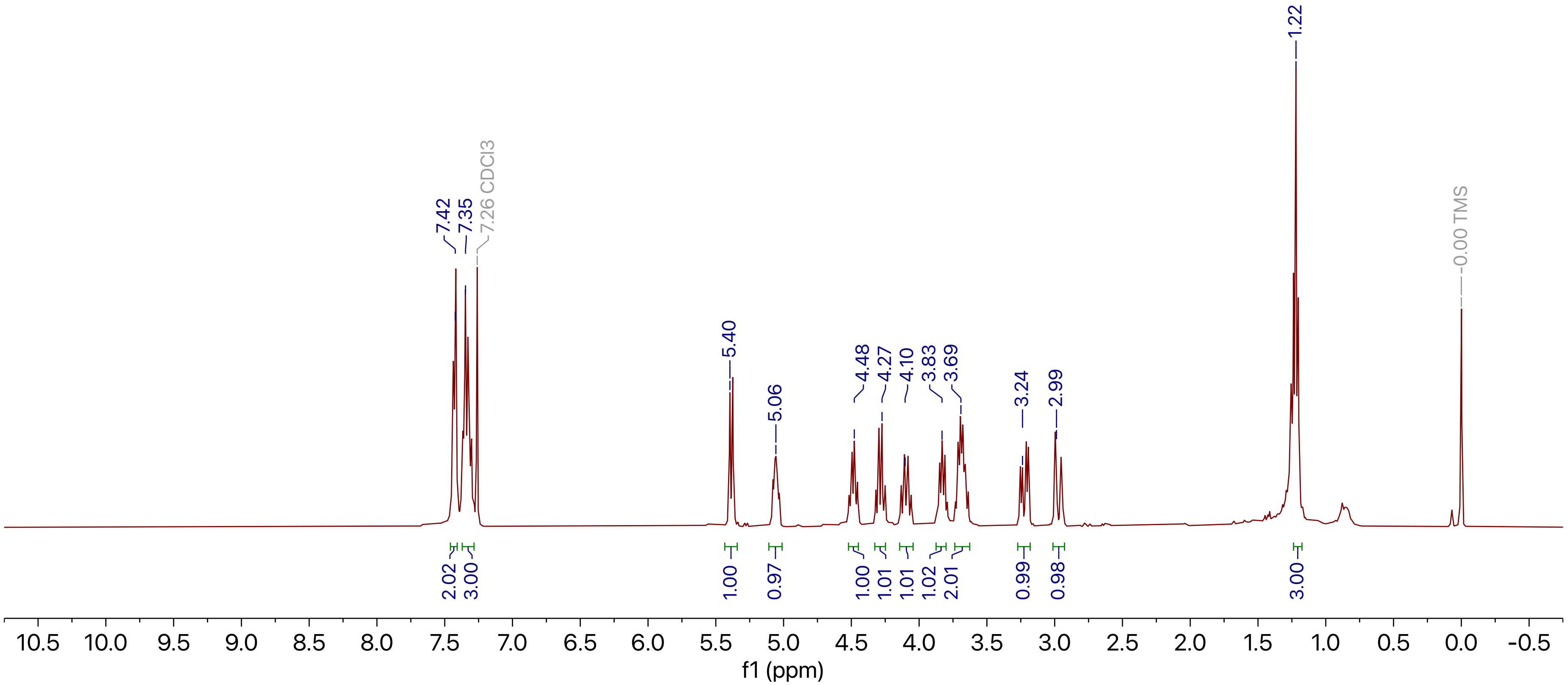

$^{13}C\{^{1}H\}$ NMR (101 MHz, $CDCl_3$)

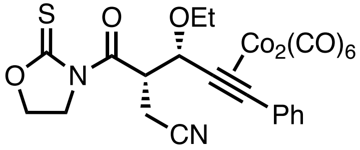

3o

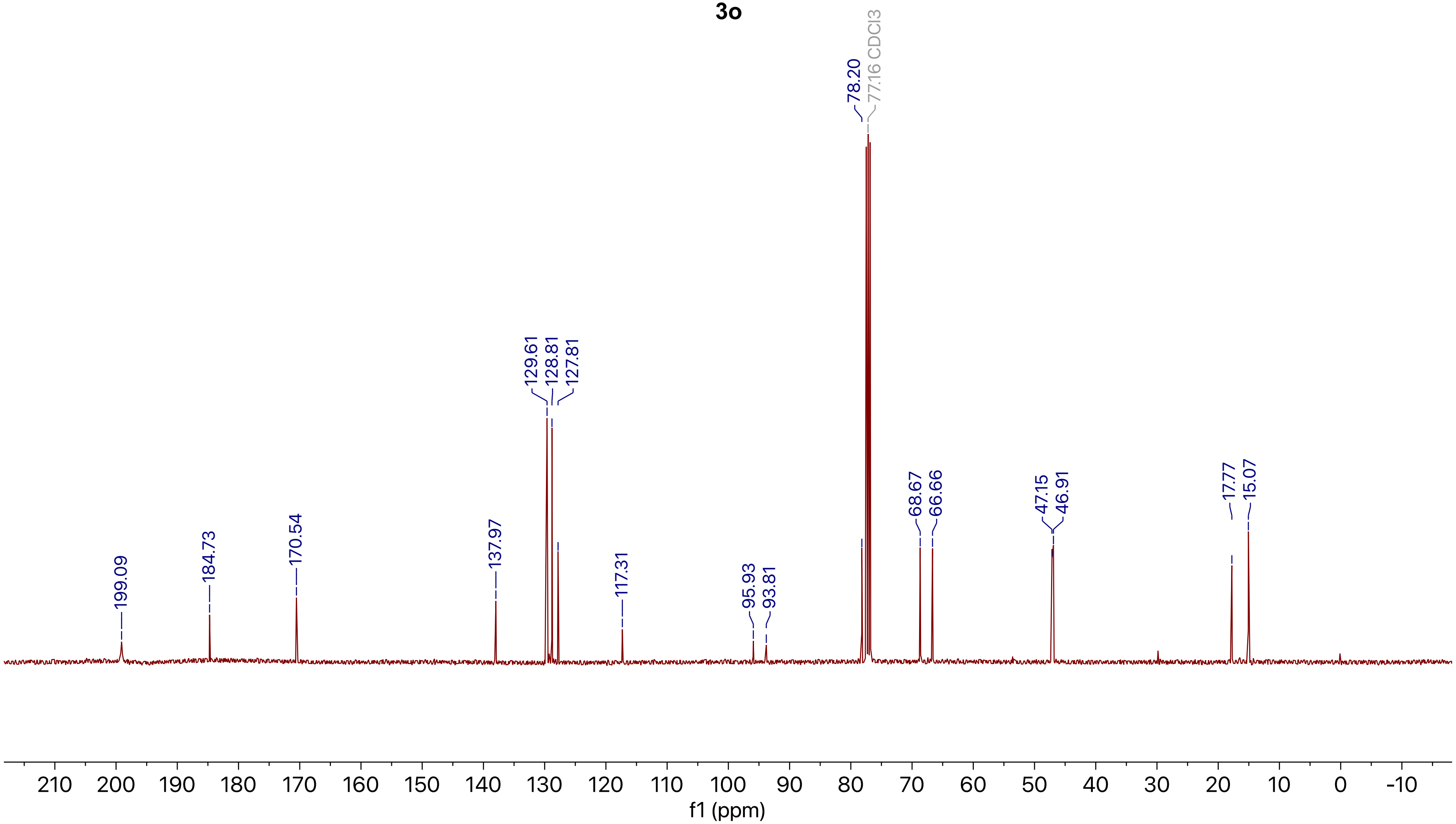

2D ^{1}H–^{1}H COSY (400 MHz, $CDCl_3$)

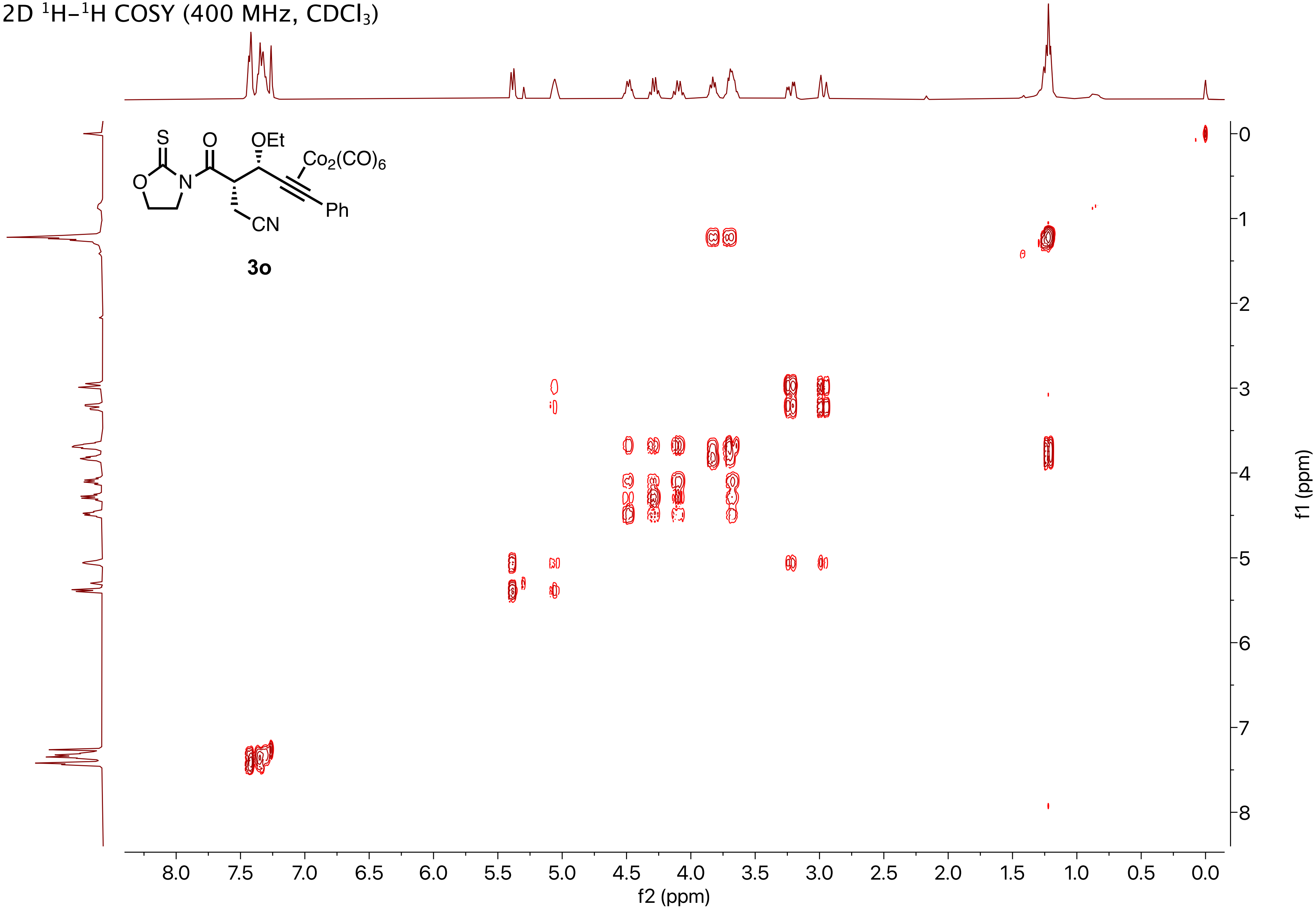

2D ^{1}H–^{13}C HSQC (400 MHz, $CDCl_3$)

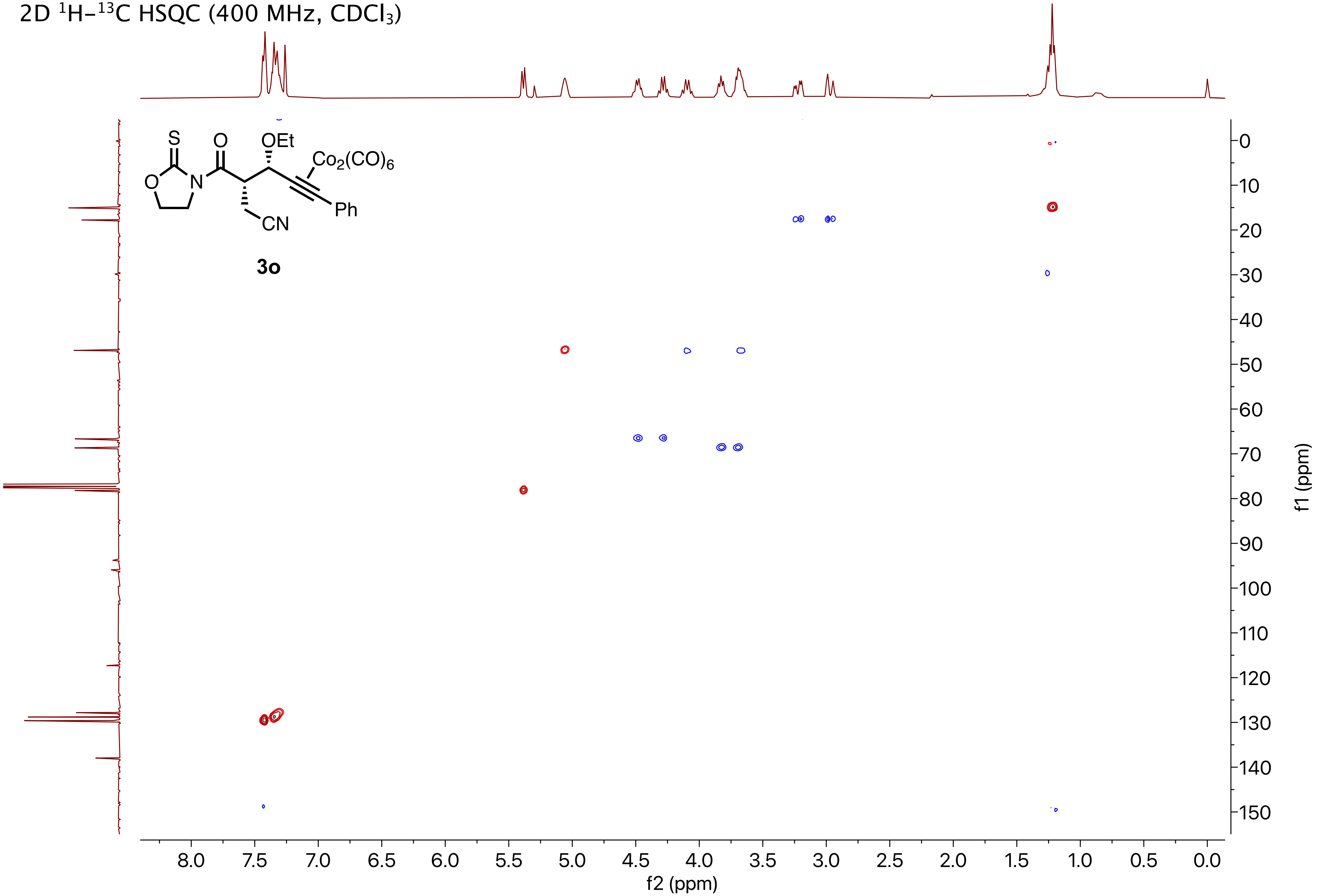

^{1}H NMR (400 MHz, $CDCl_3$)

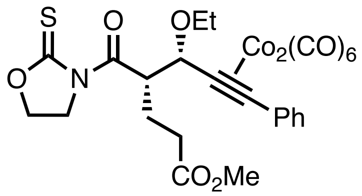

3p

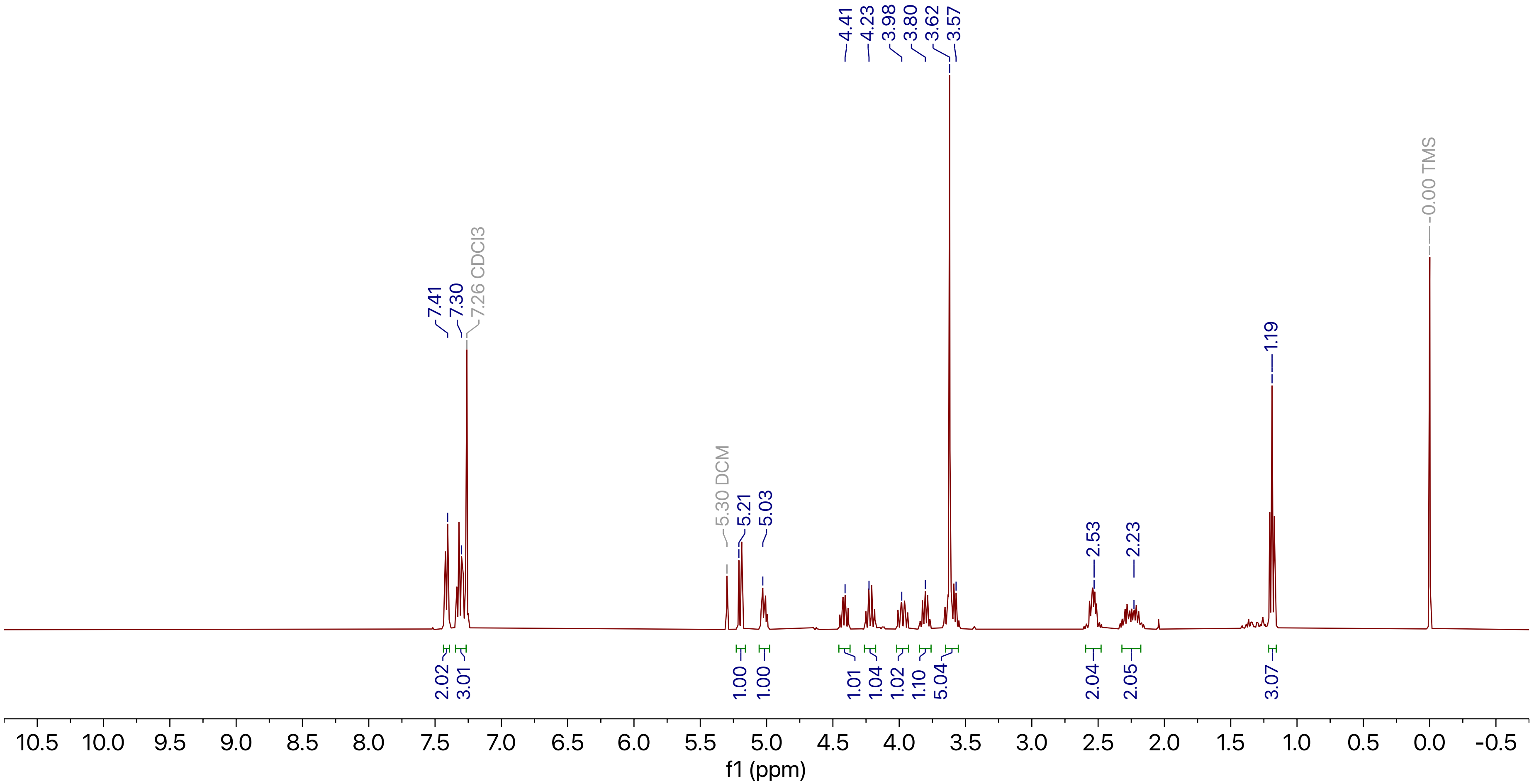

$^{13}C\{^{1}H\}$ NMR (101 MHz, $CDCl_3$)

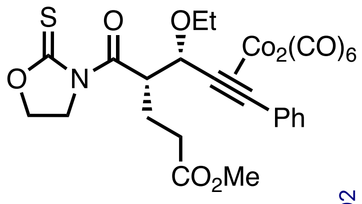

3p

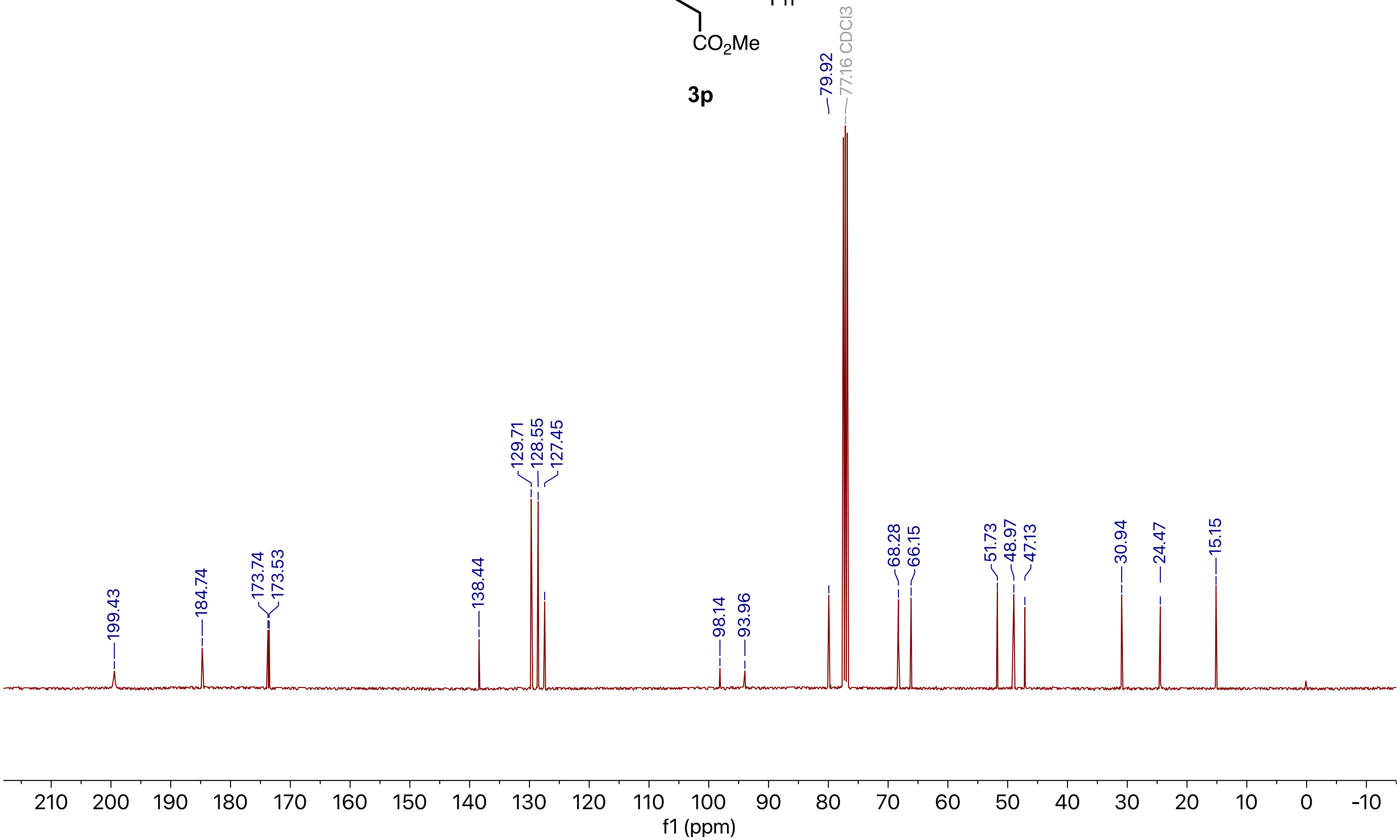

2D $^{1}H-^{1}H$ COSY (400 MHz, $CDCl_3$)

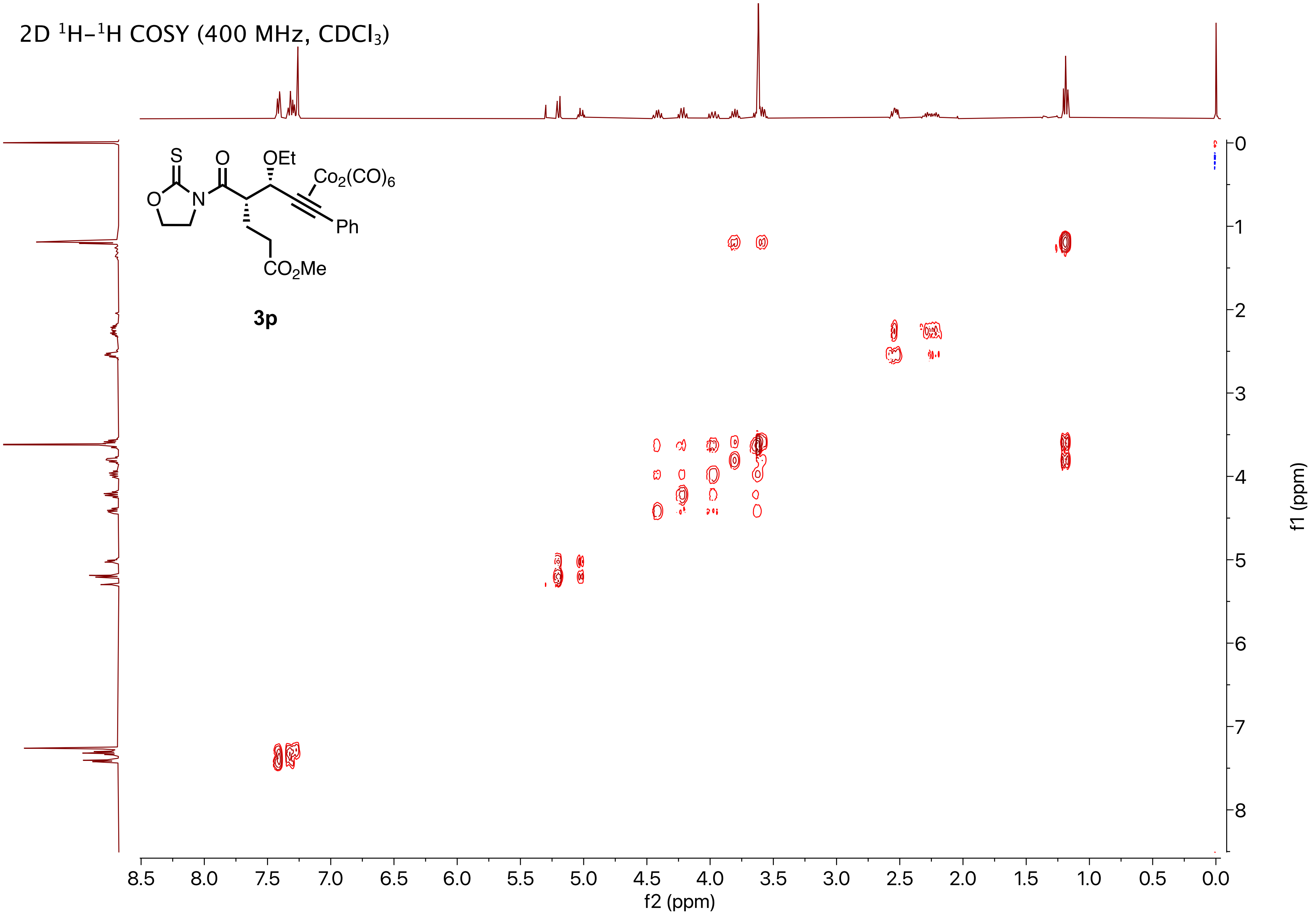

2D ^{1}H–^{13}C HSQC (400 MHz, $CDCl_3$)

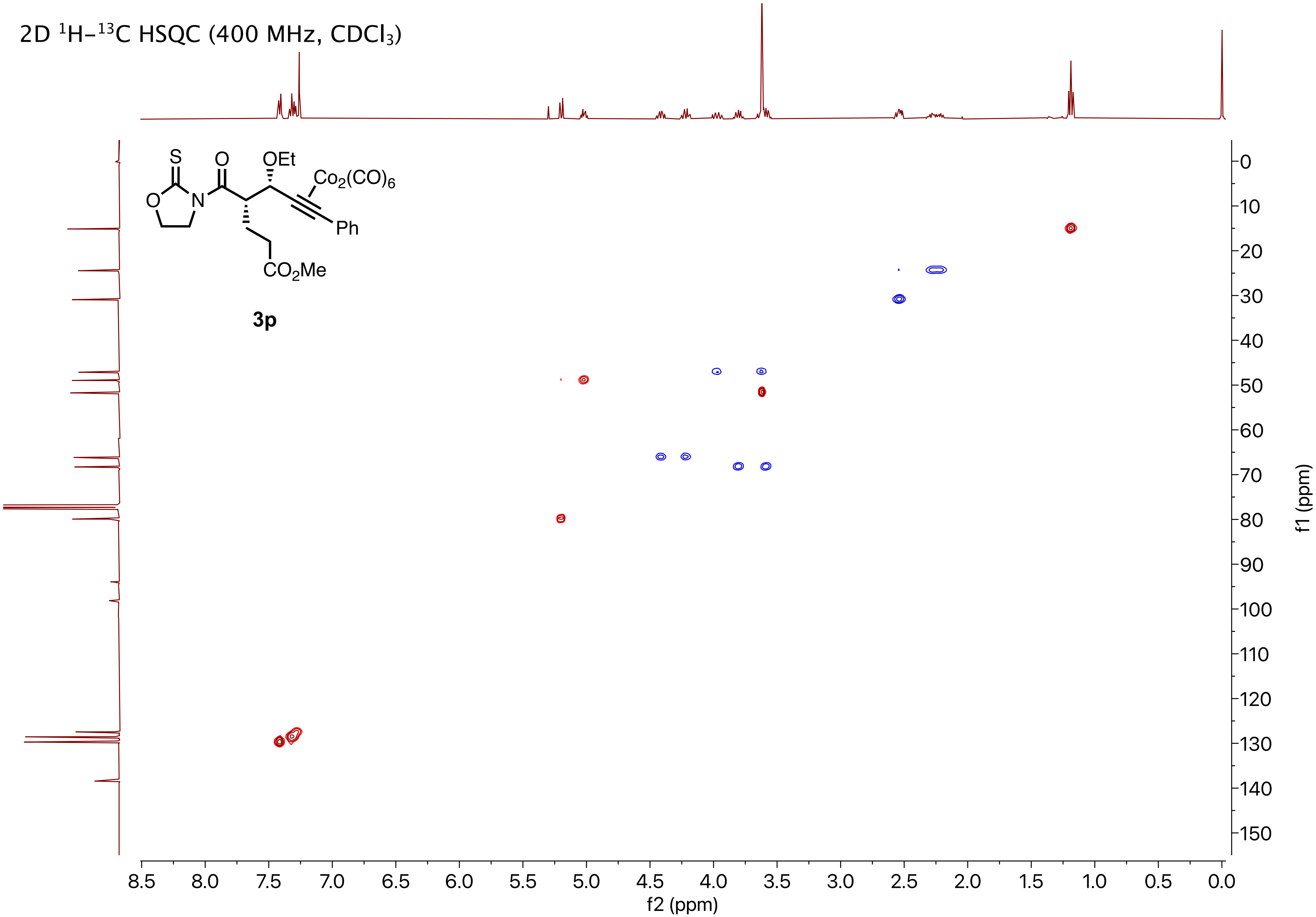

^1H NMR (400 MHz, $CDCl_3$)

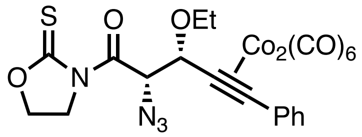

3q

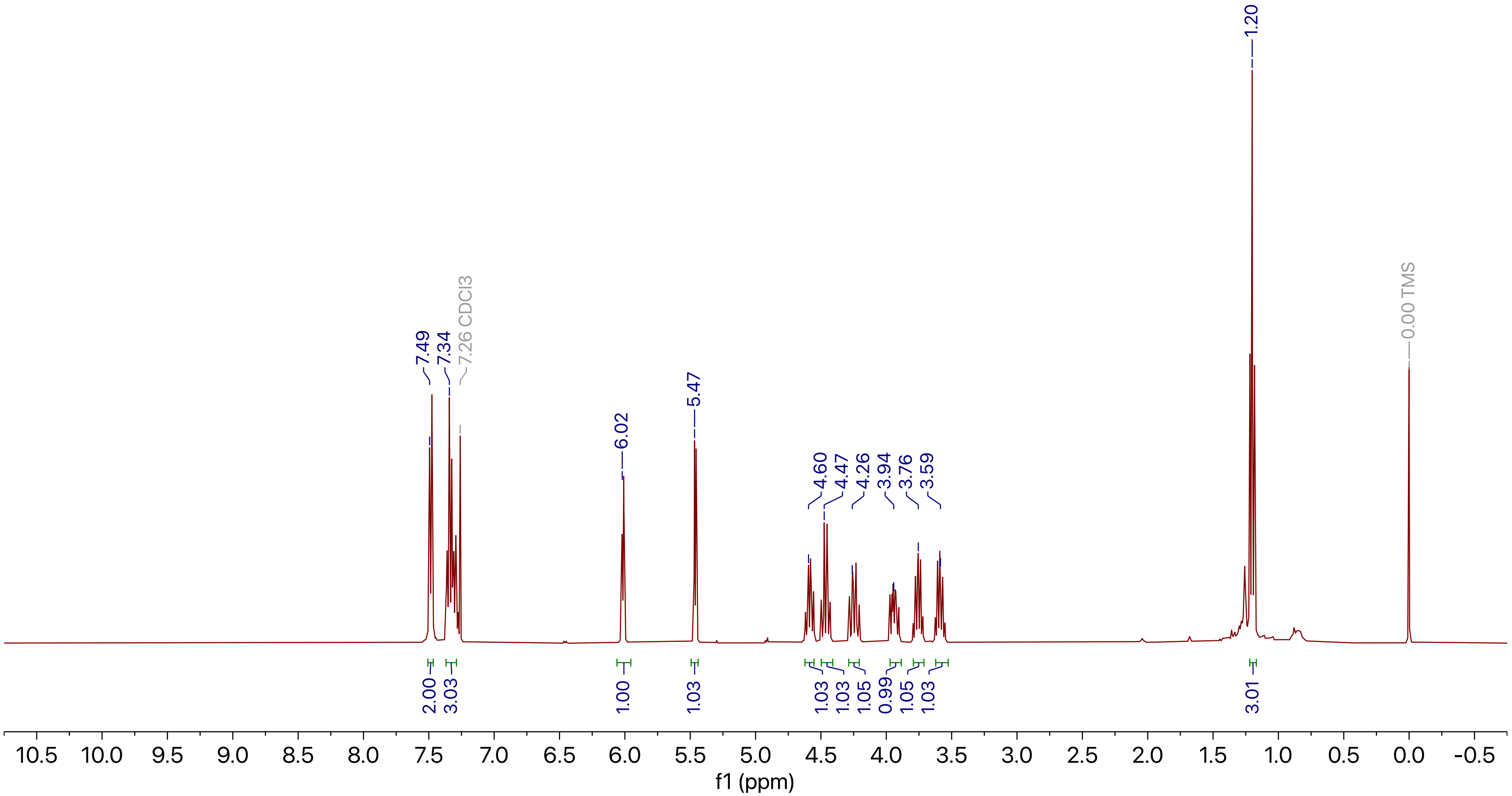

$^{13}C\{^1H\}$ NMR (101 MHz, $CDCl_3$)

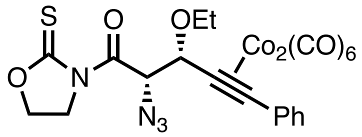

3q

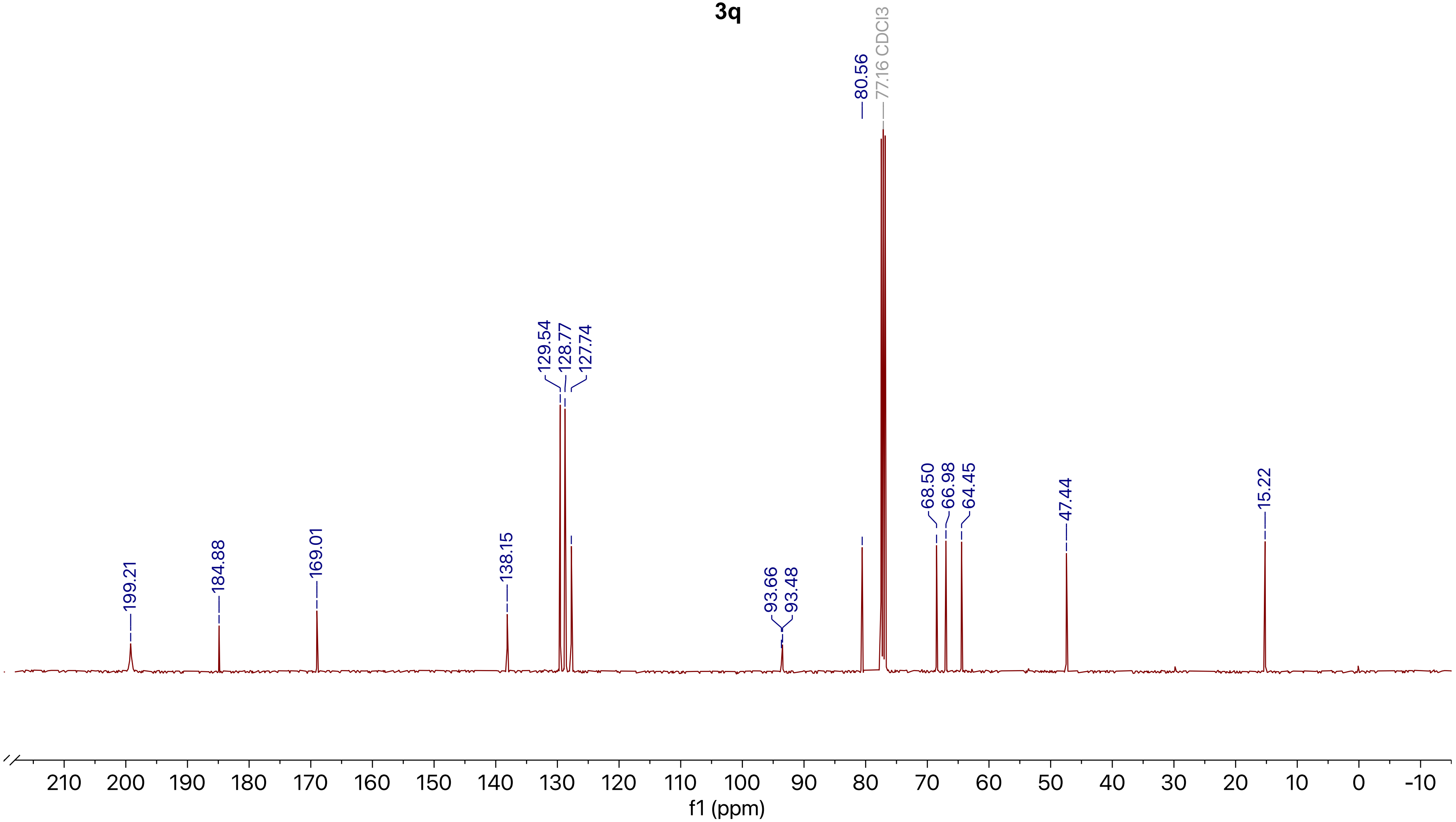

2D ^{1}H–^{1}H COSY (400 MHz, $CDCl_3$)

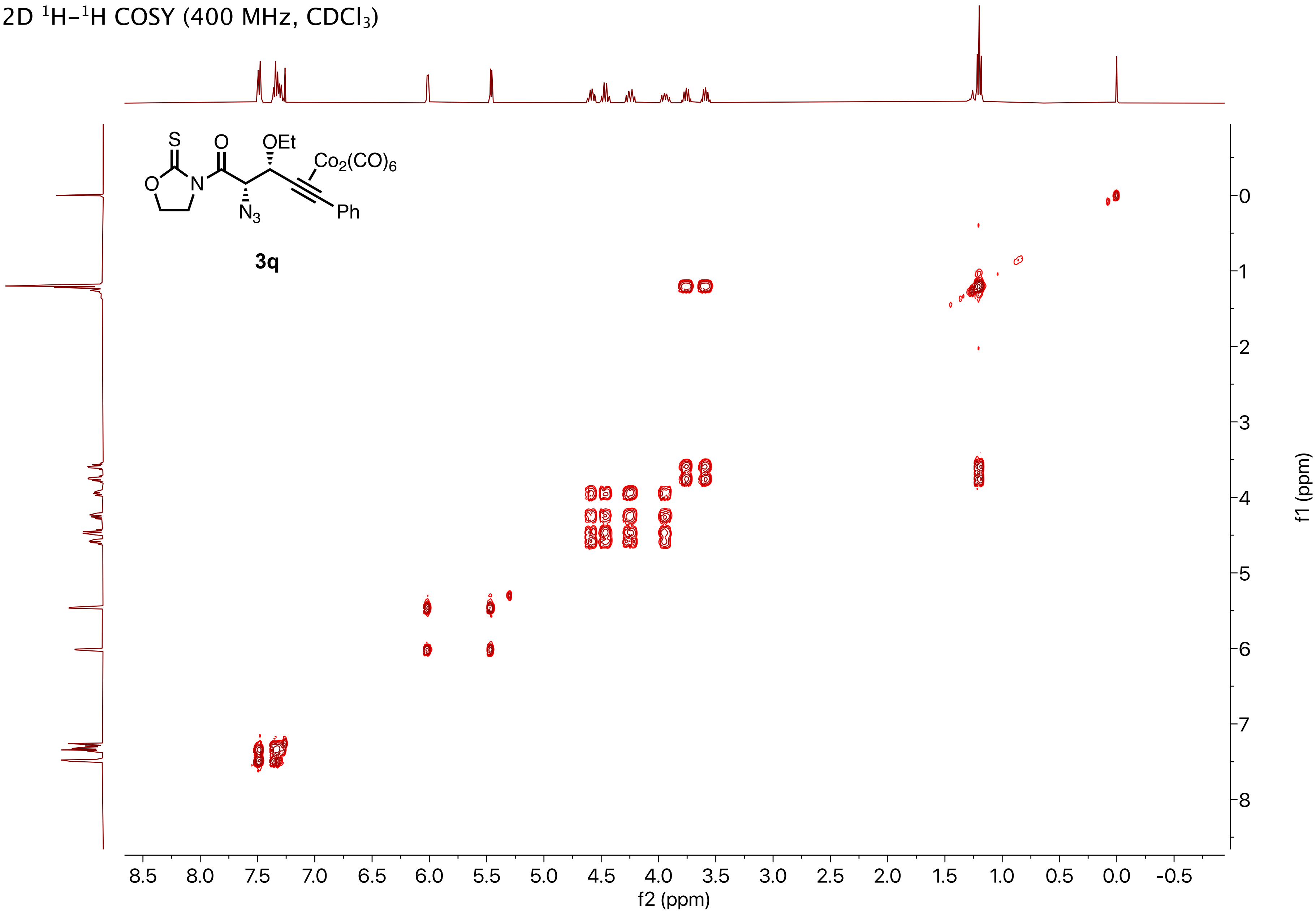

2D ^{1}H–^{13}C HSQC (400 MHz, $CDCl_3$)

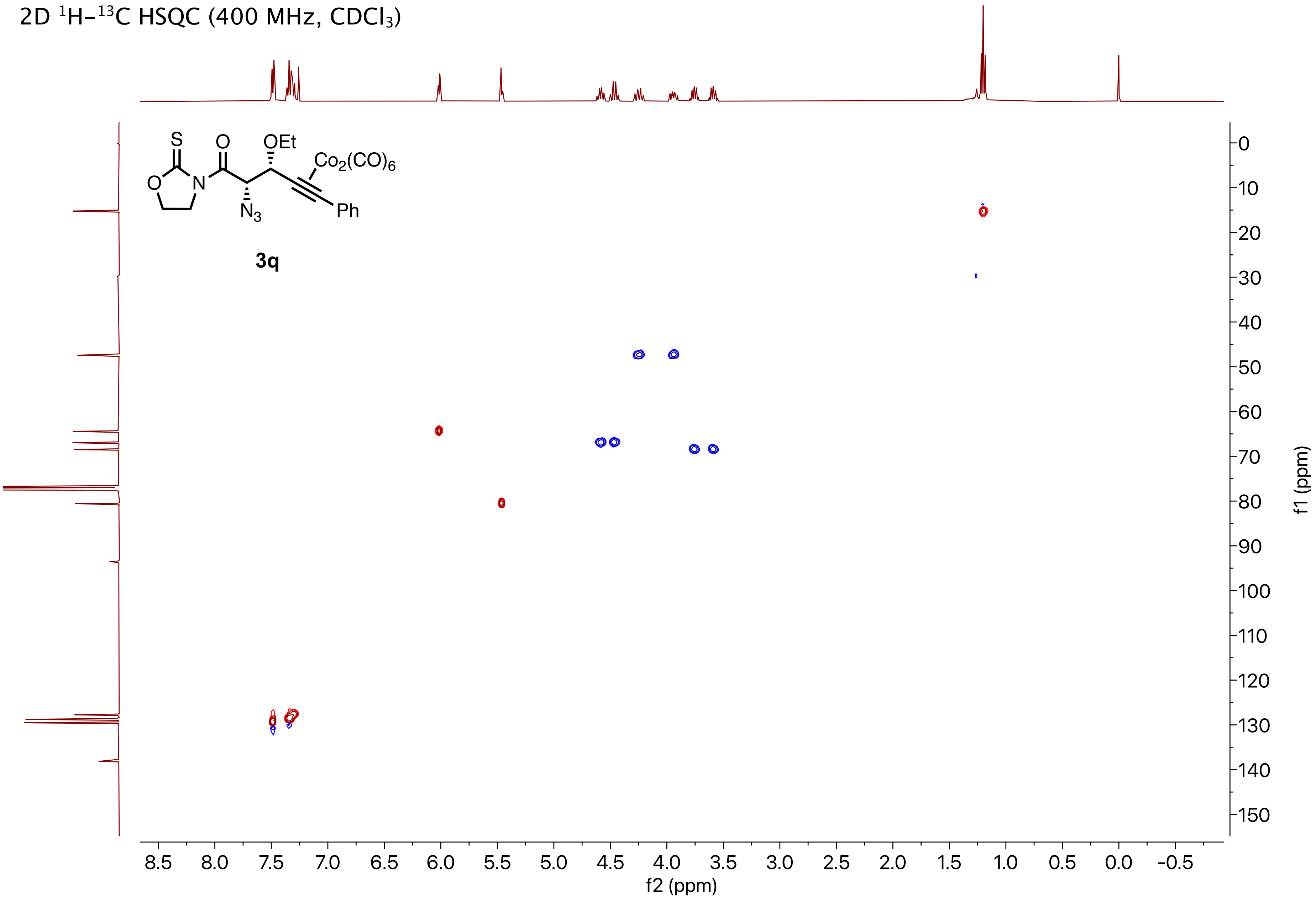

^{1}H NMR (400 MHz, $CDCl_3$)

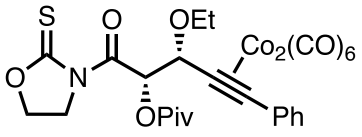

3r

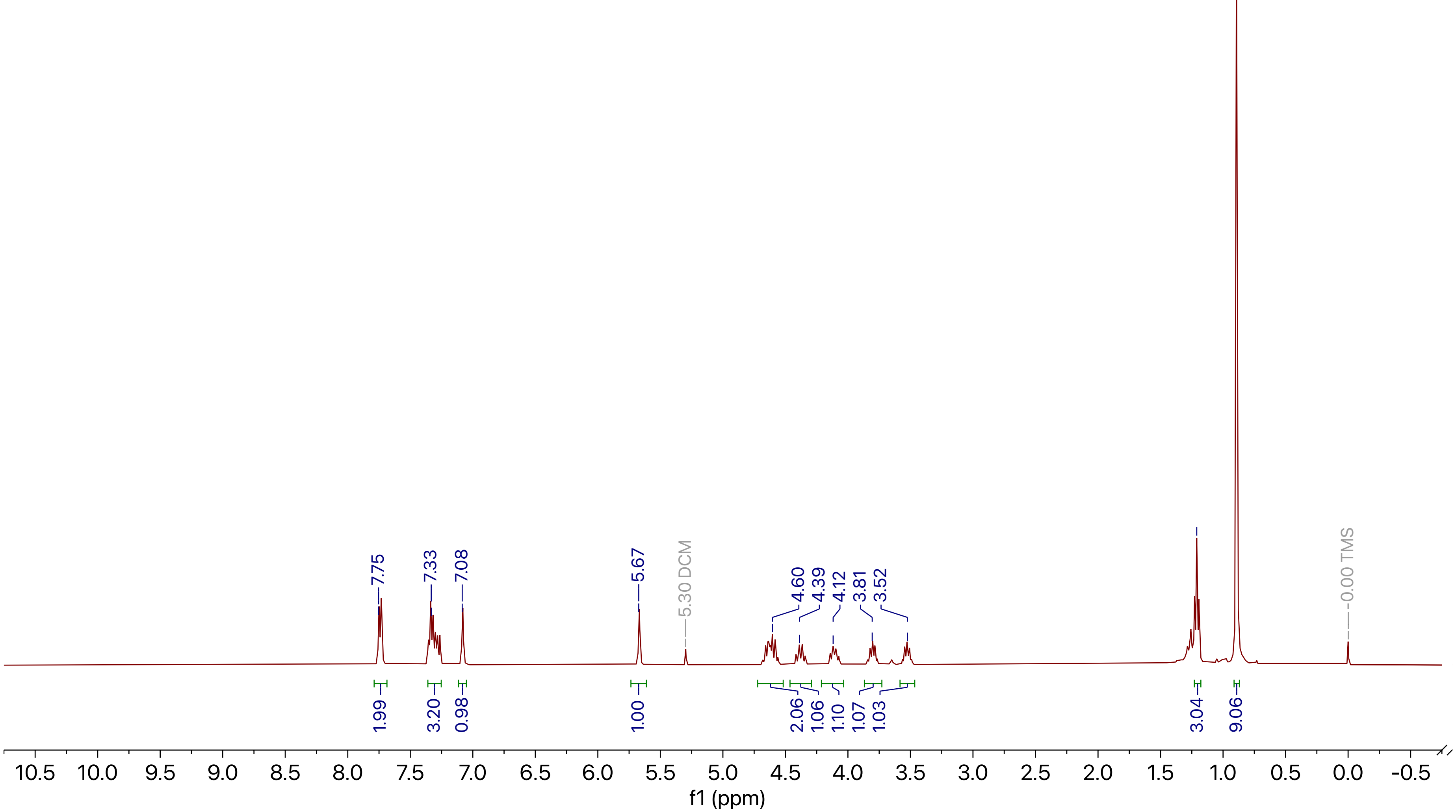

$^{13}C\{^{1}H\}$ NMR (101 MHz, $CDCl_3$)

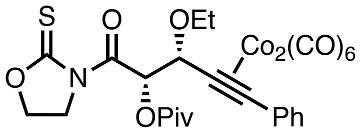

3r

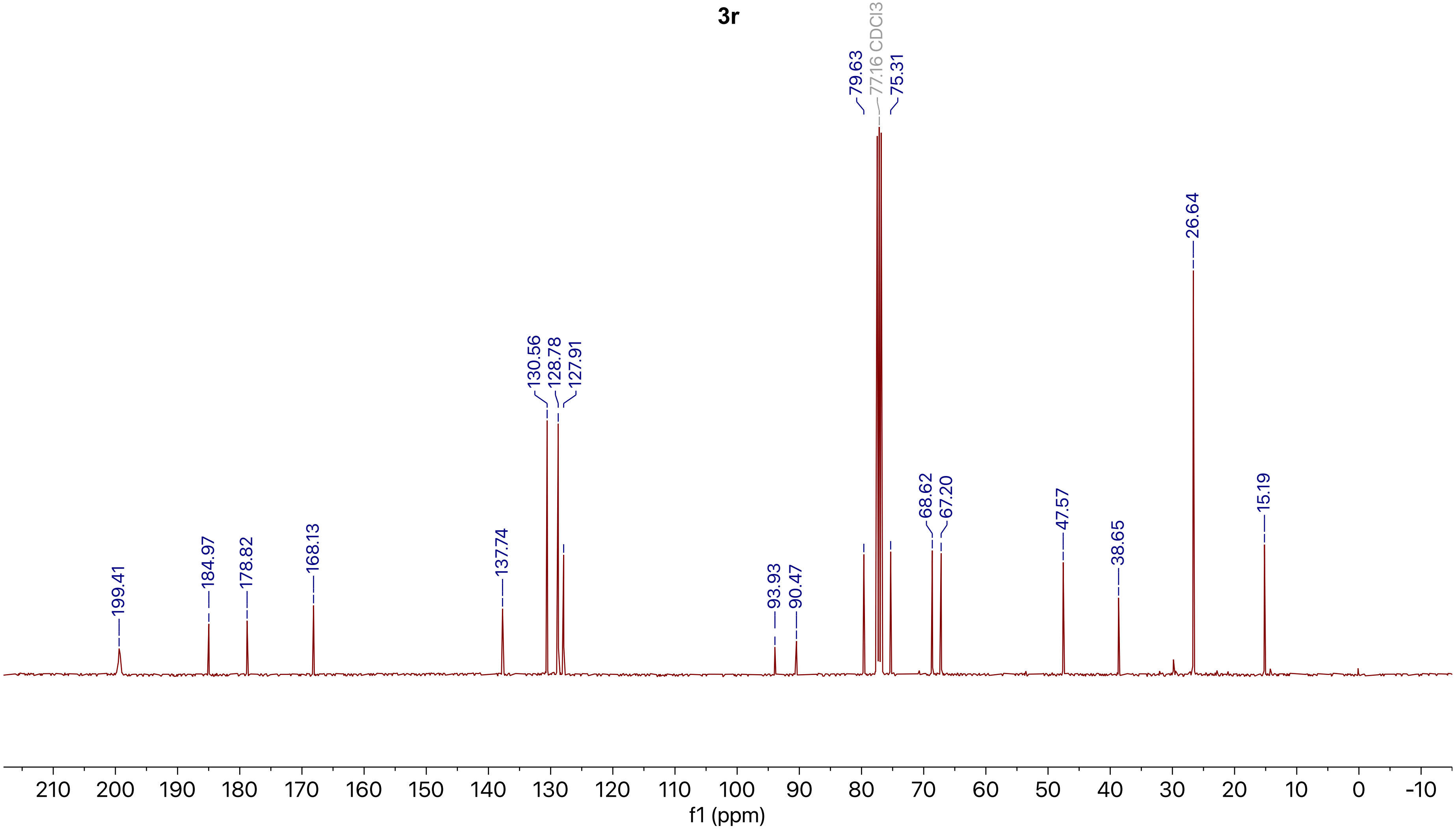

2D ^{1}H–^{1}H COSY (400 MHz, $CDCl_3$)

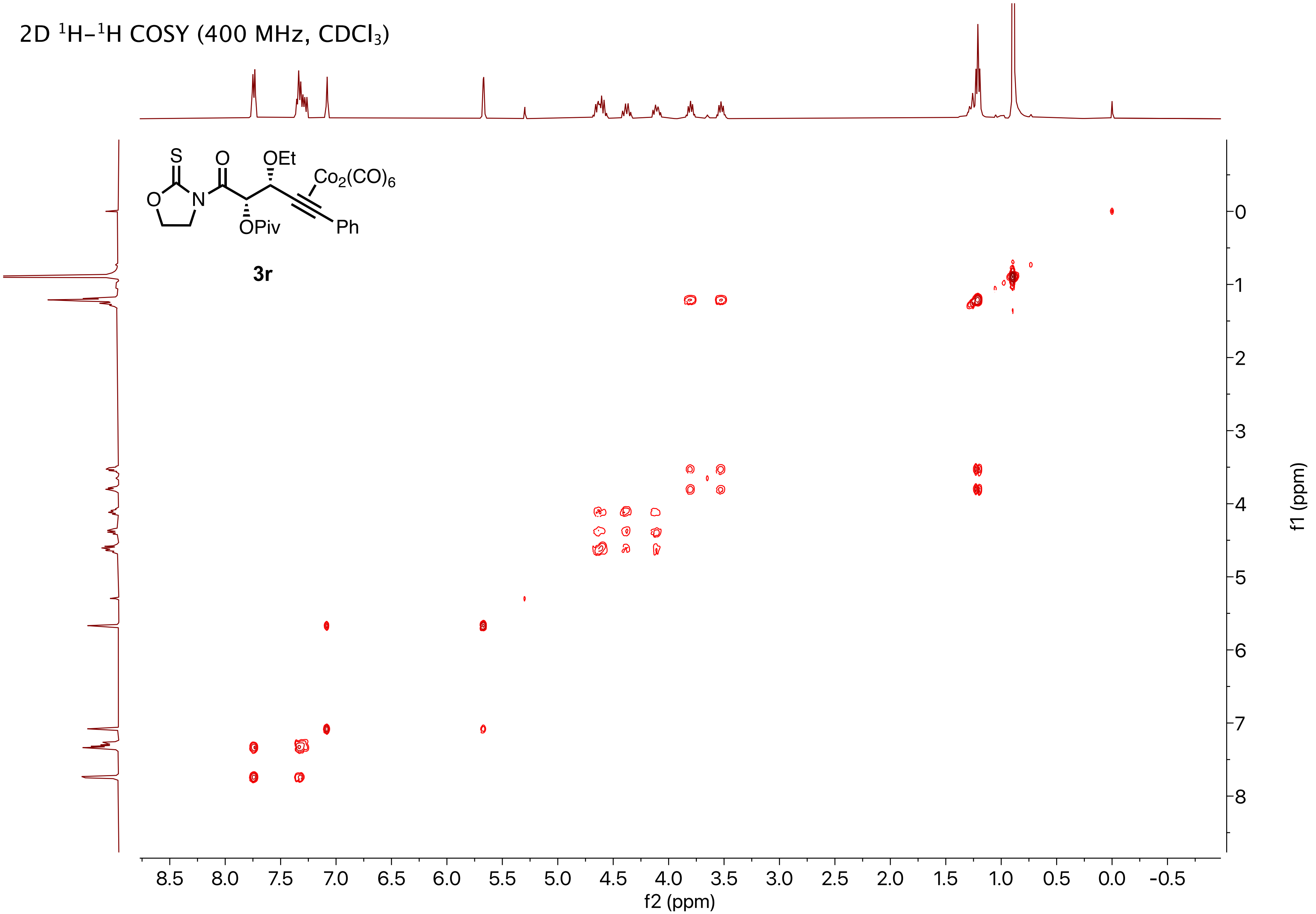

2D ^{1}H–^{13}C HSQC (400 MHz, $CDCl_3$)

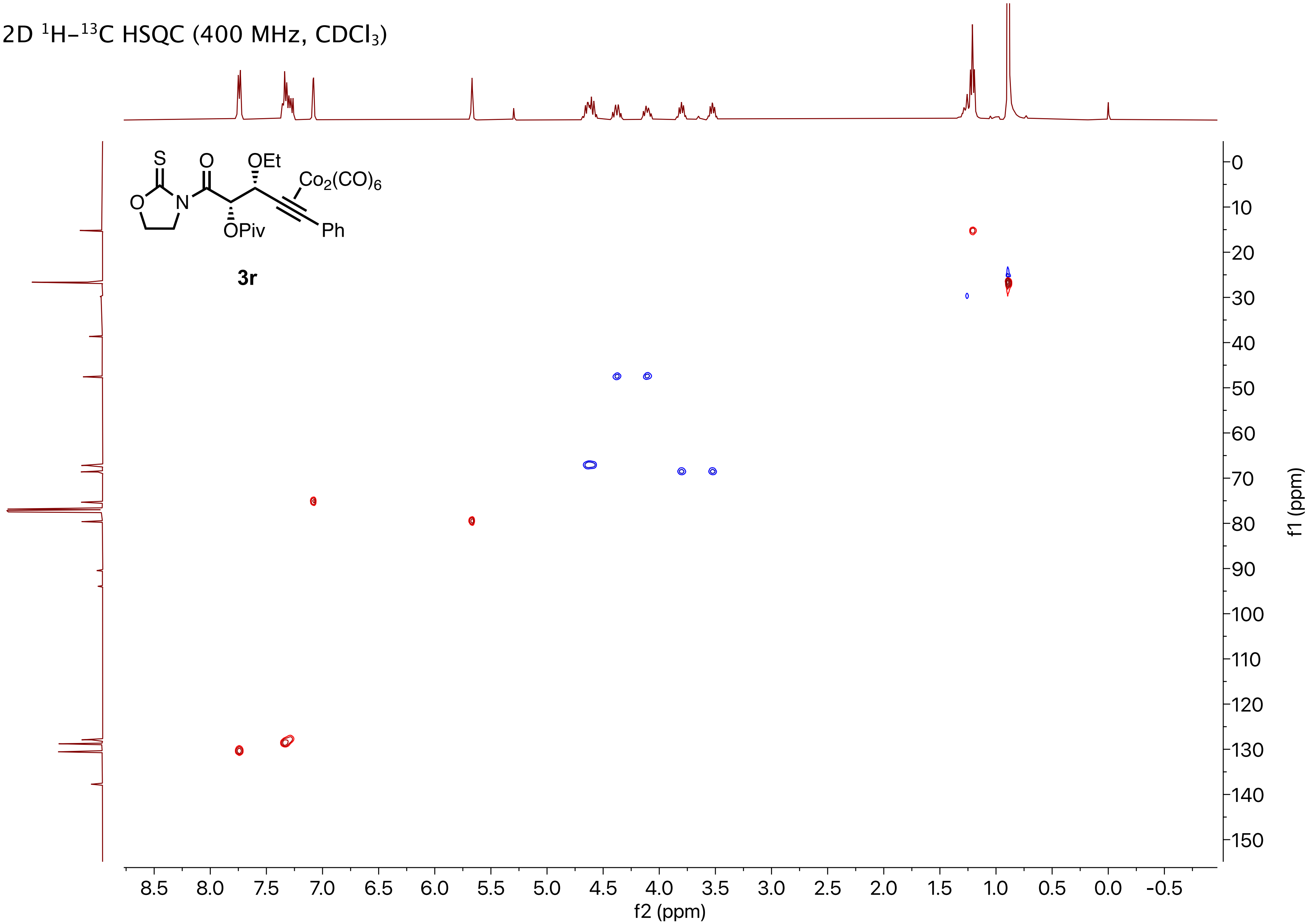

3r

^{1}H NMR (400 MHz, $CDCl_3$)

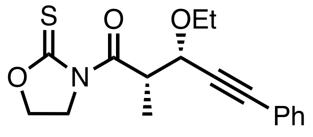

4a

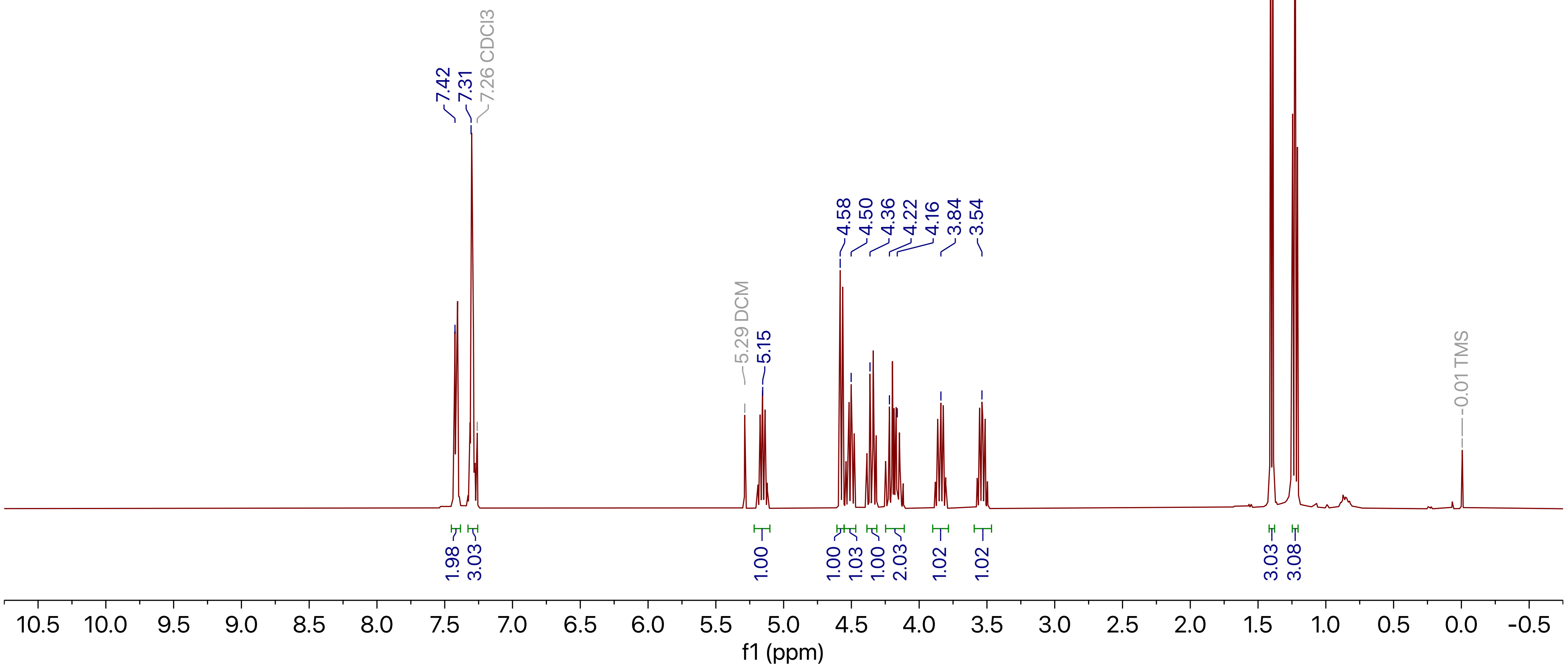

$^{13}C\{^{1}H\}$ NMR (101 MHz, $CDCl_3$)

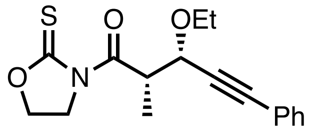

4a

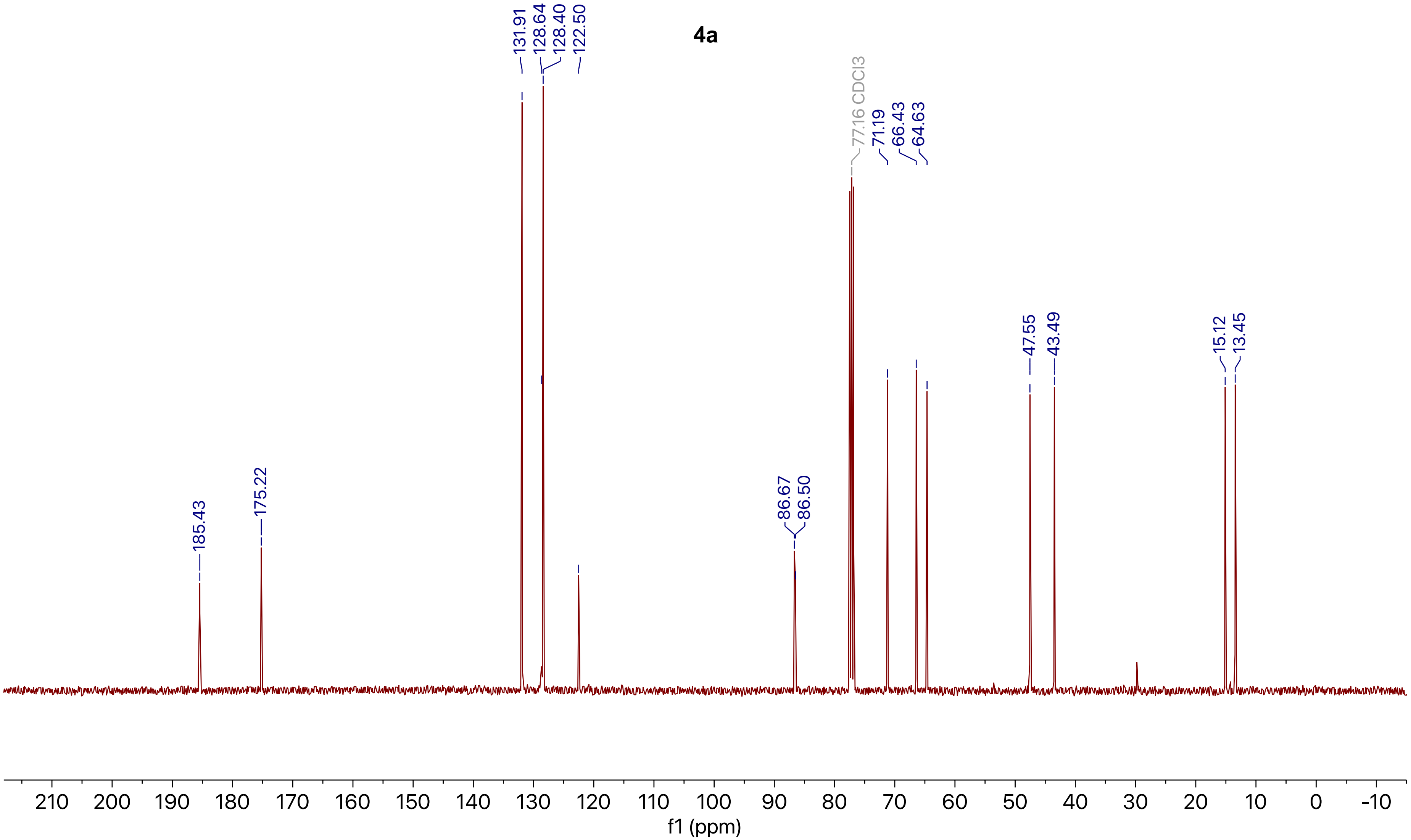

2D ^{1}H–^{1}H COSY (400 MHz, $CDCl_3$)

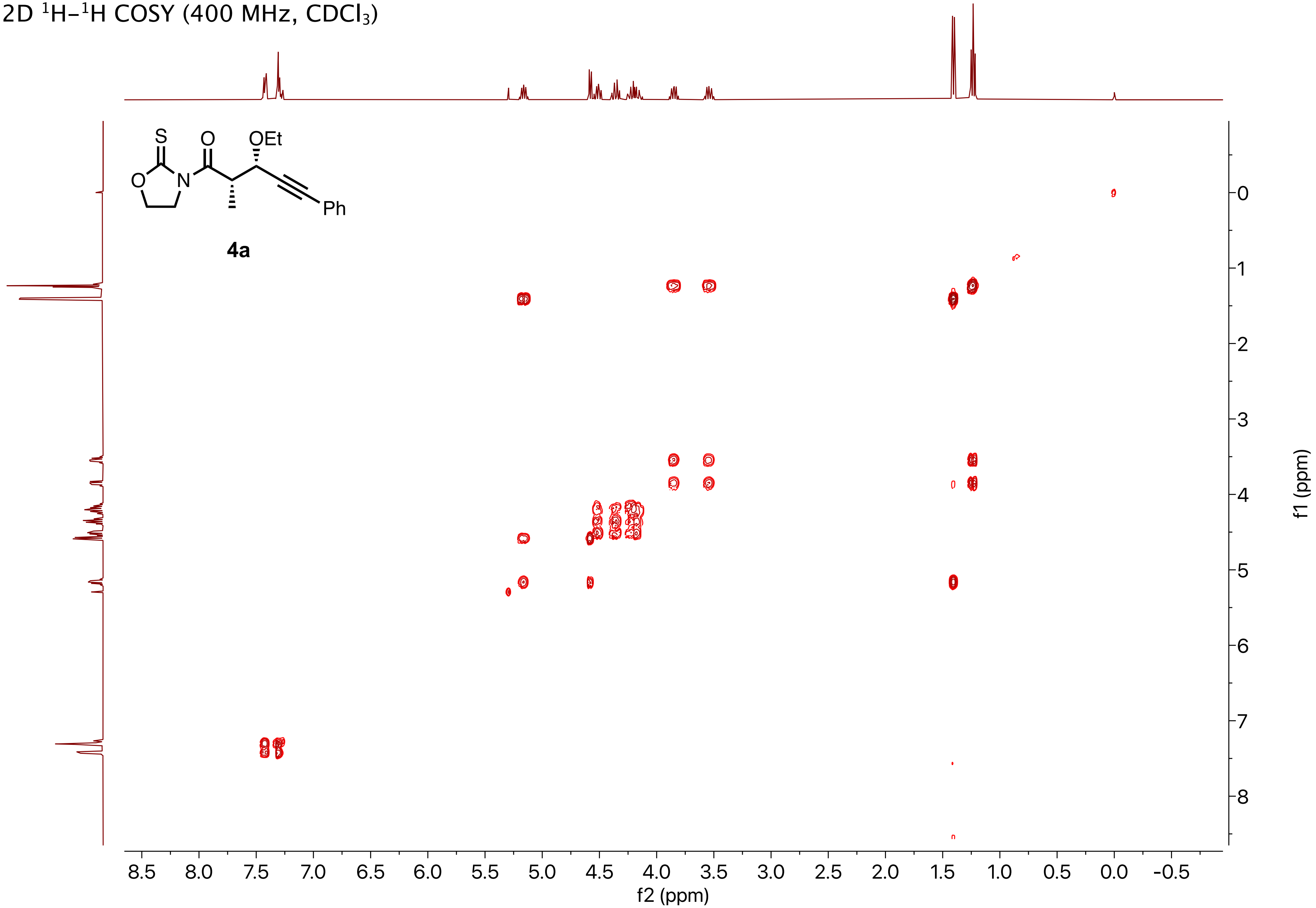

2D $^1H-^{13}C$ HSQC (400 MHz, $CDCl_3$)

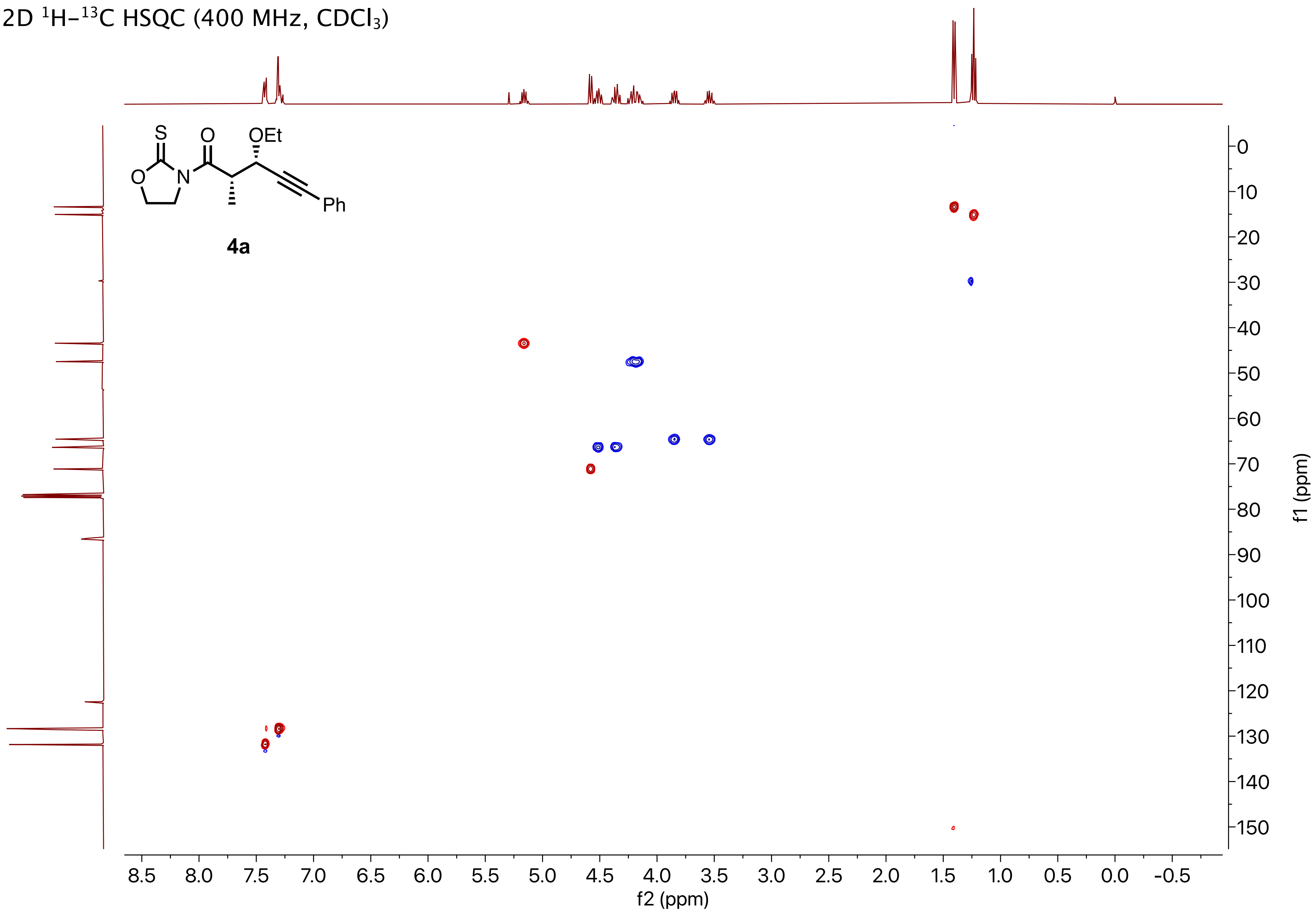

^1H NMR (400 MHz, $CDCl_3$)

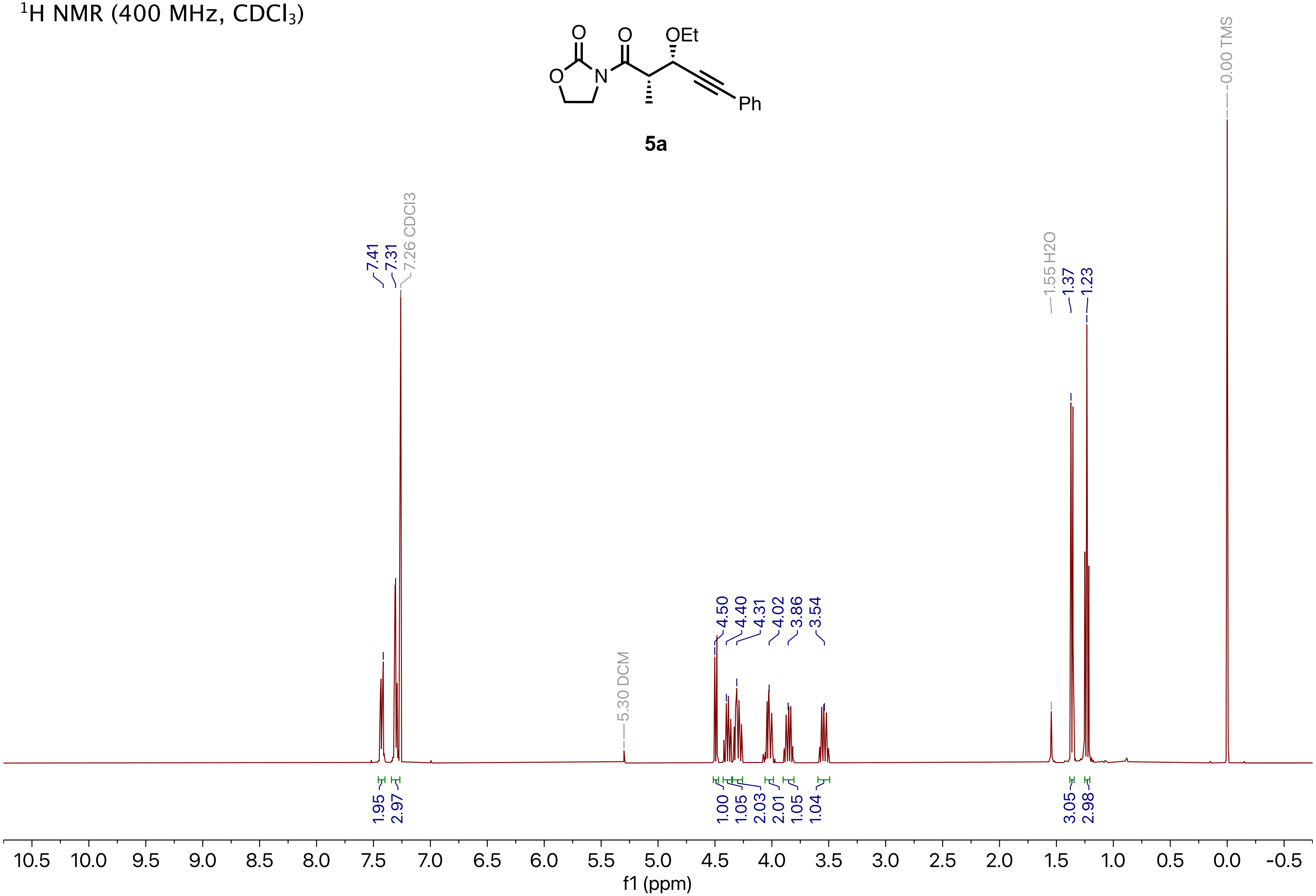

$^{13}C\{^{1}H\}$ NMR (101 MHz, $CDCl_3$)

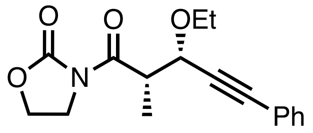

5a

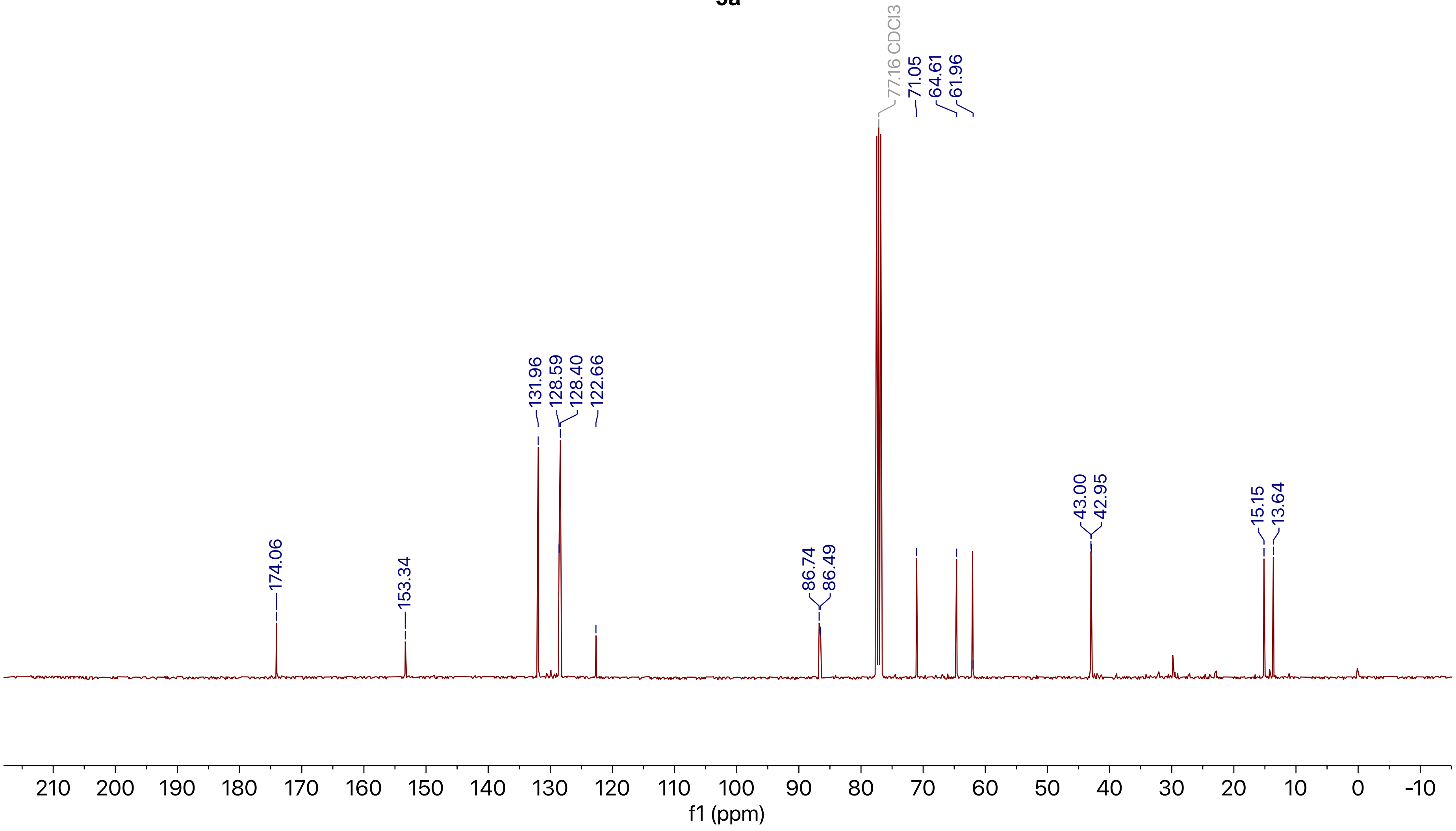

2D ^{1}H–^{1}H COSY (400 MHz, $CDCl_3$)

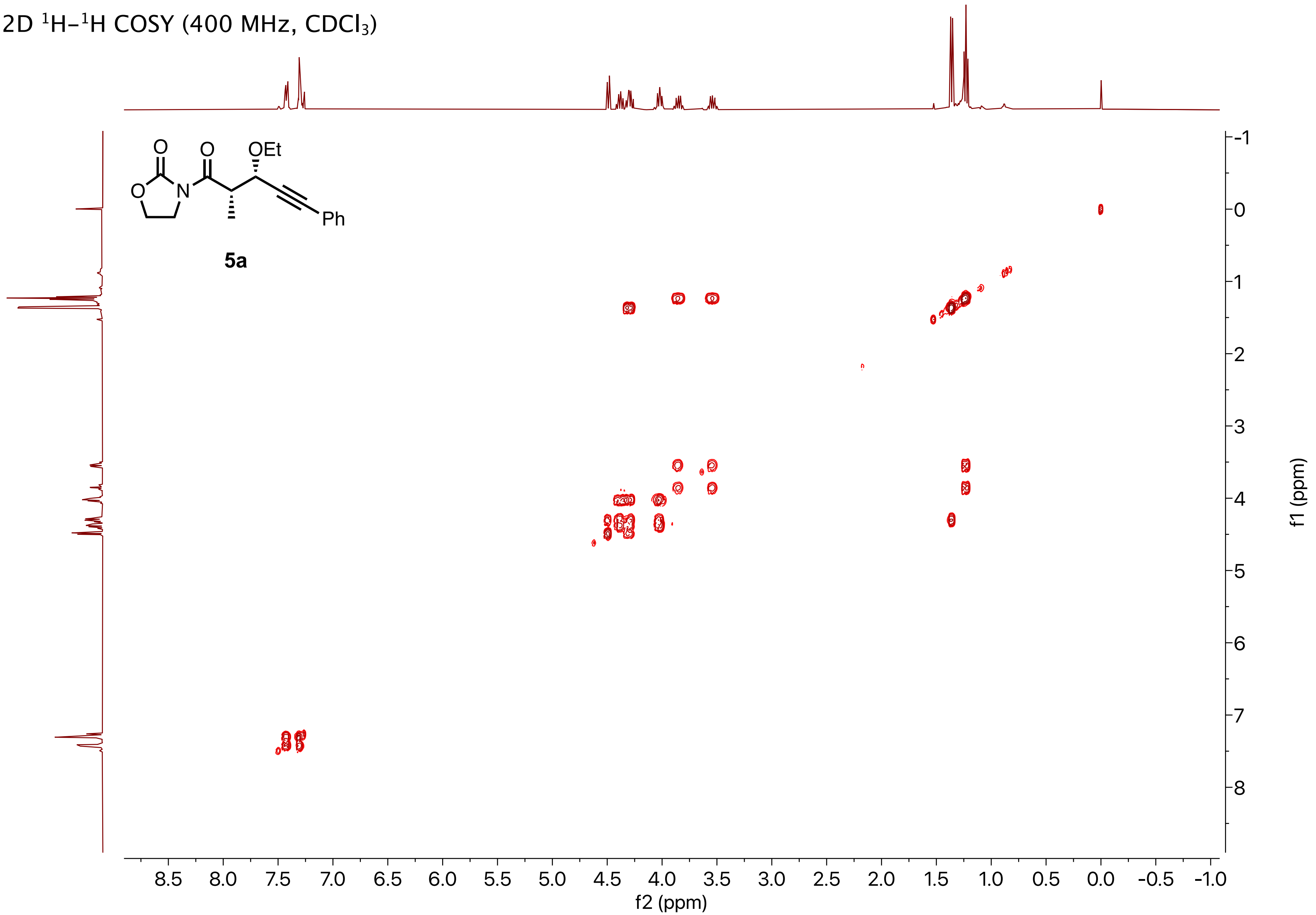

2D ^{1}H-^{13}C HSQC (400 MHz, $CDCl_3$)

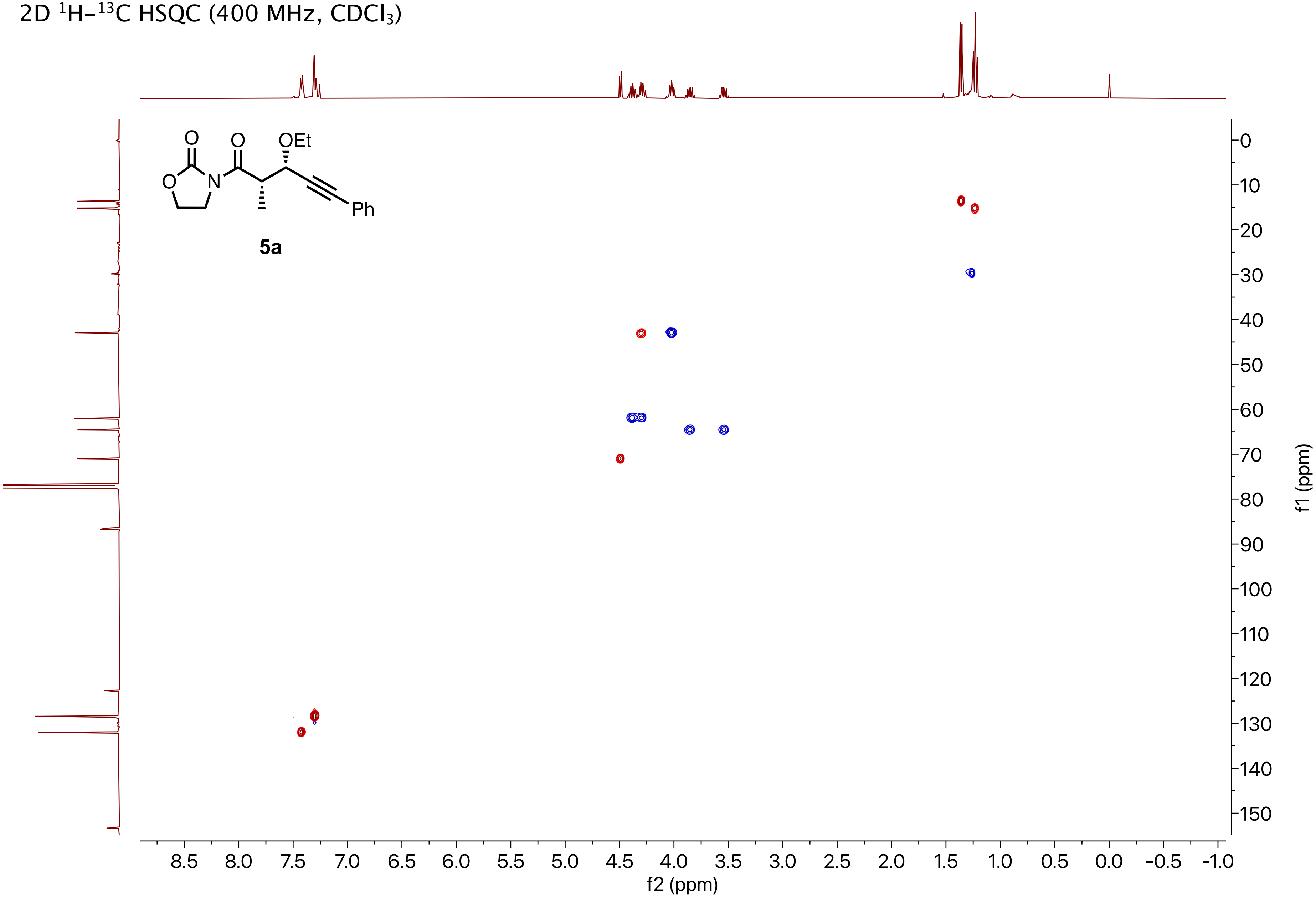

^{1}H NMR (400 MHz, $CDCl_3$)

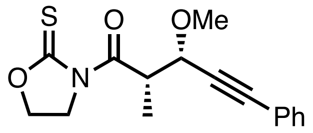

4b

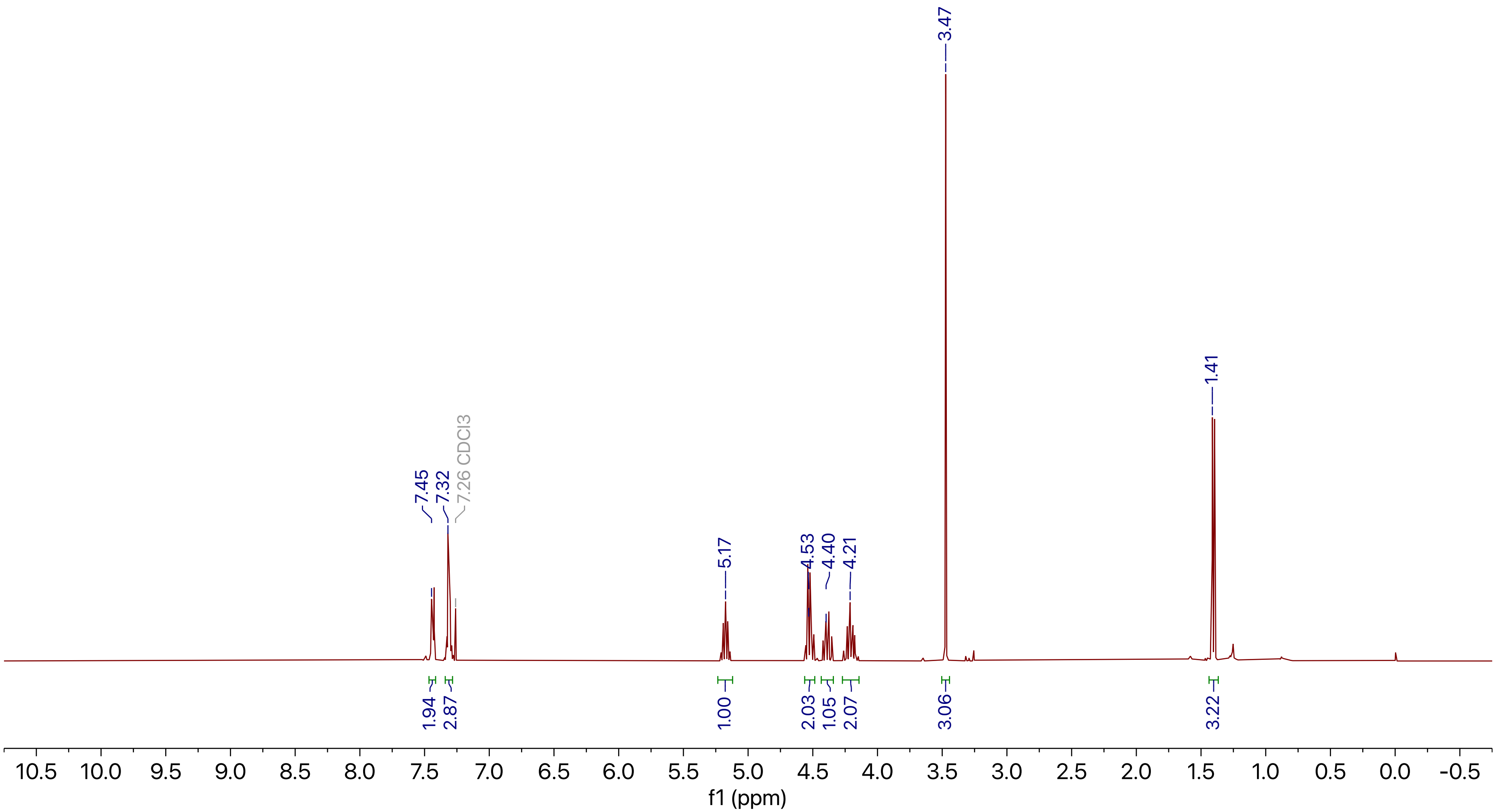

$^{13}C\{^1H\}$ NMR (101 MHz, $CDCl_3$)

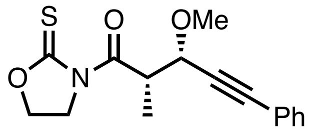

4b

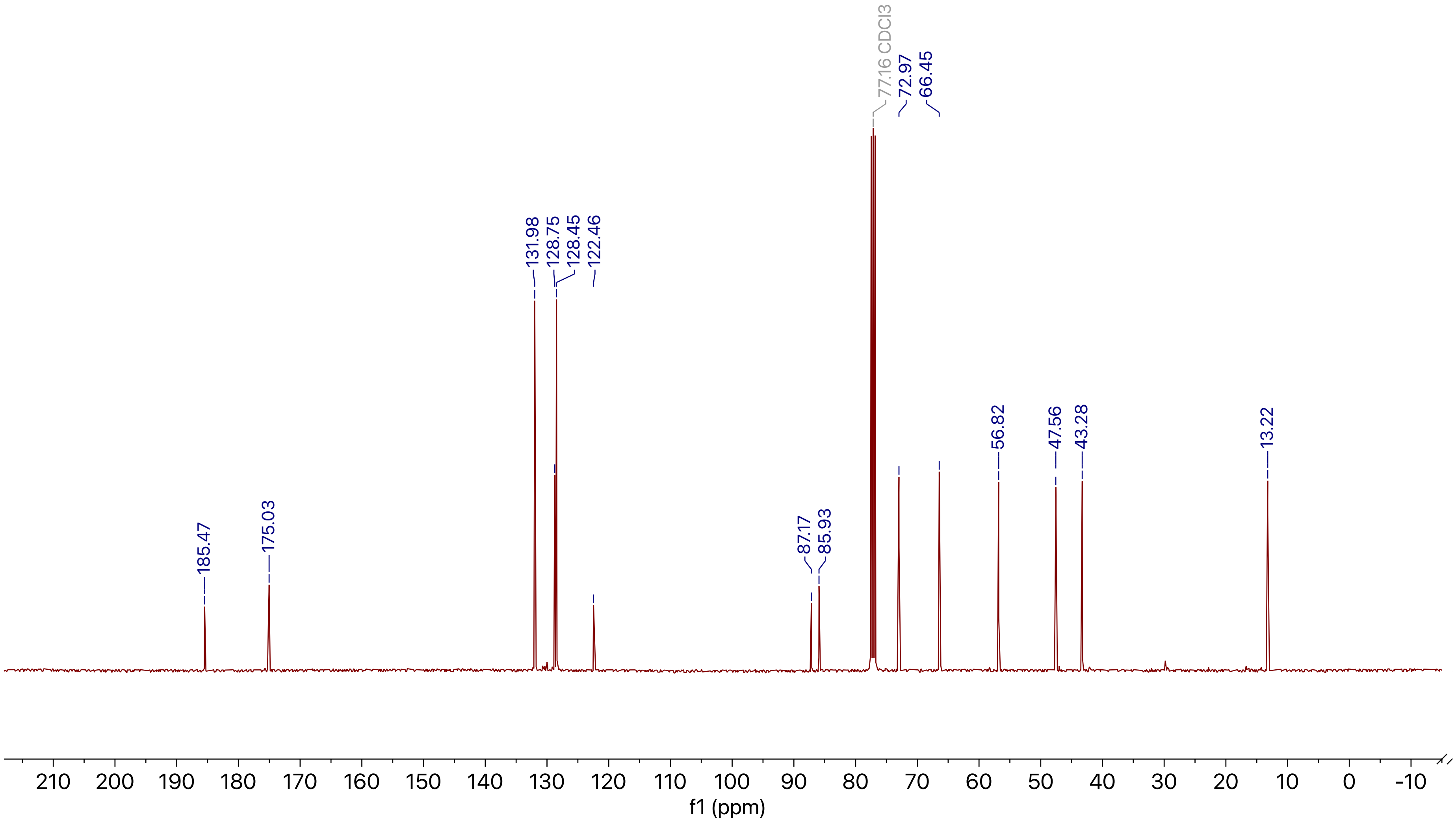

2D ^{1}H–^{1}H COSY (400 MHz, $CDCl_3$)

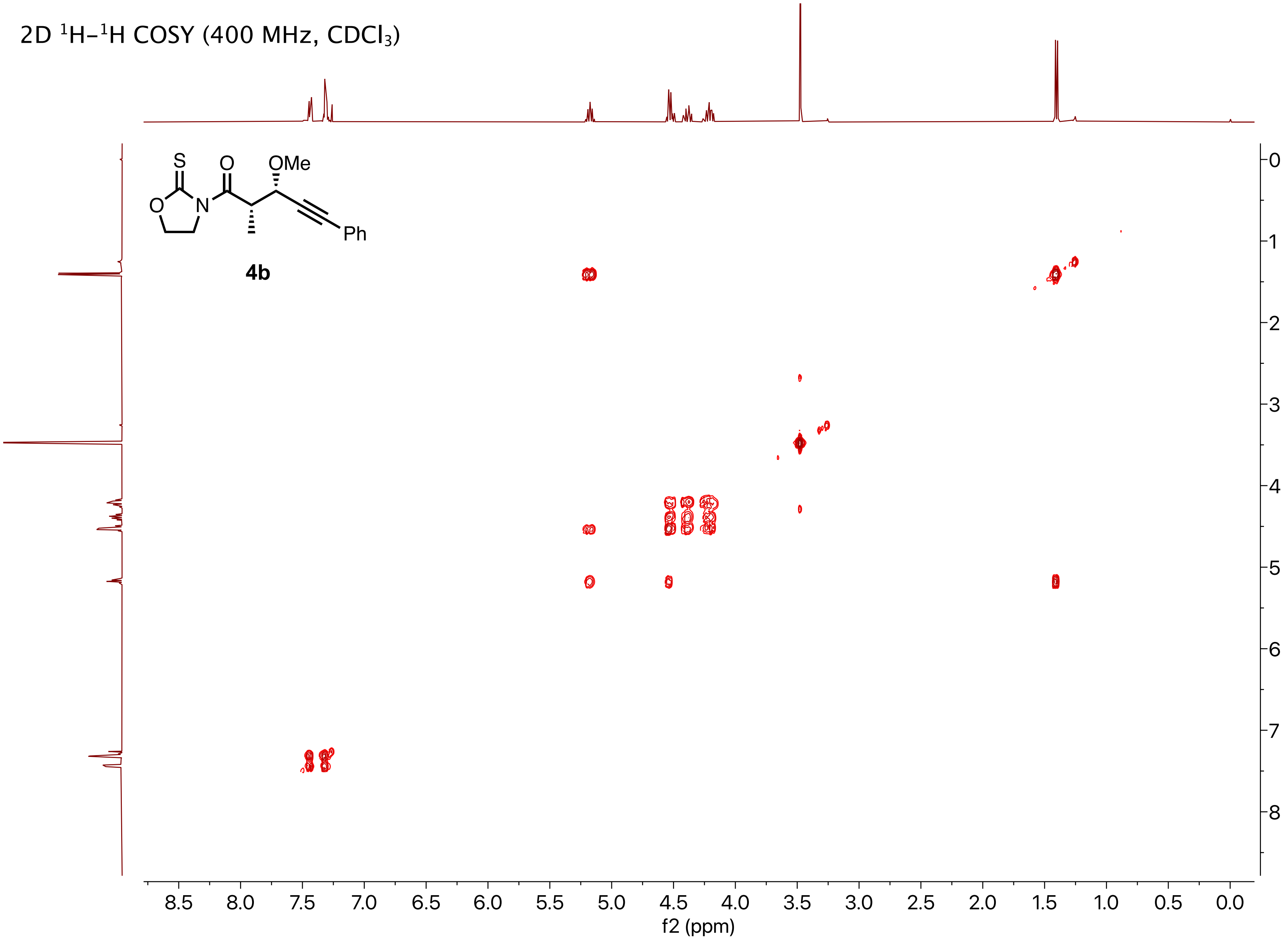

4b

2D ^{1}H–^{13}C HSQC (400 MHz, $CDCl_3$)

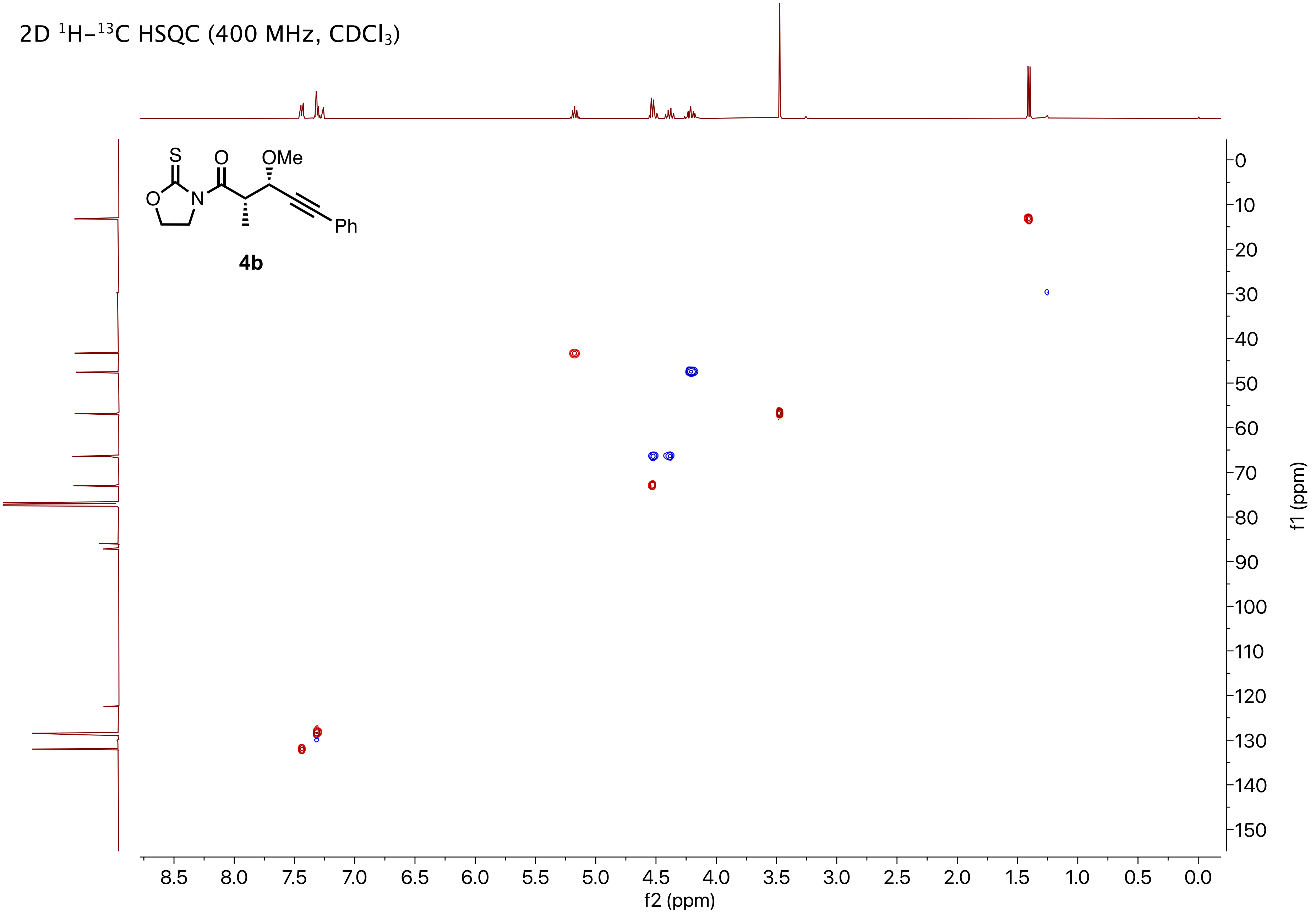

^{1}H NMR (400 MHz, $CDCl_3$)

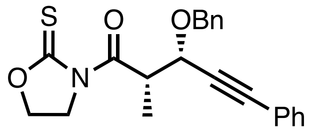

4c

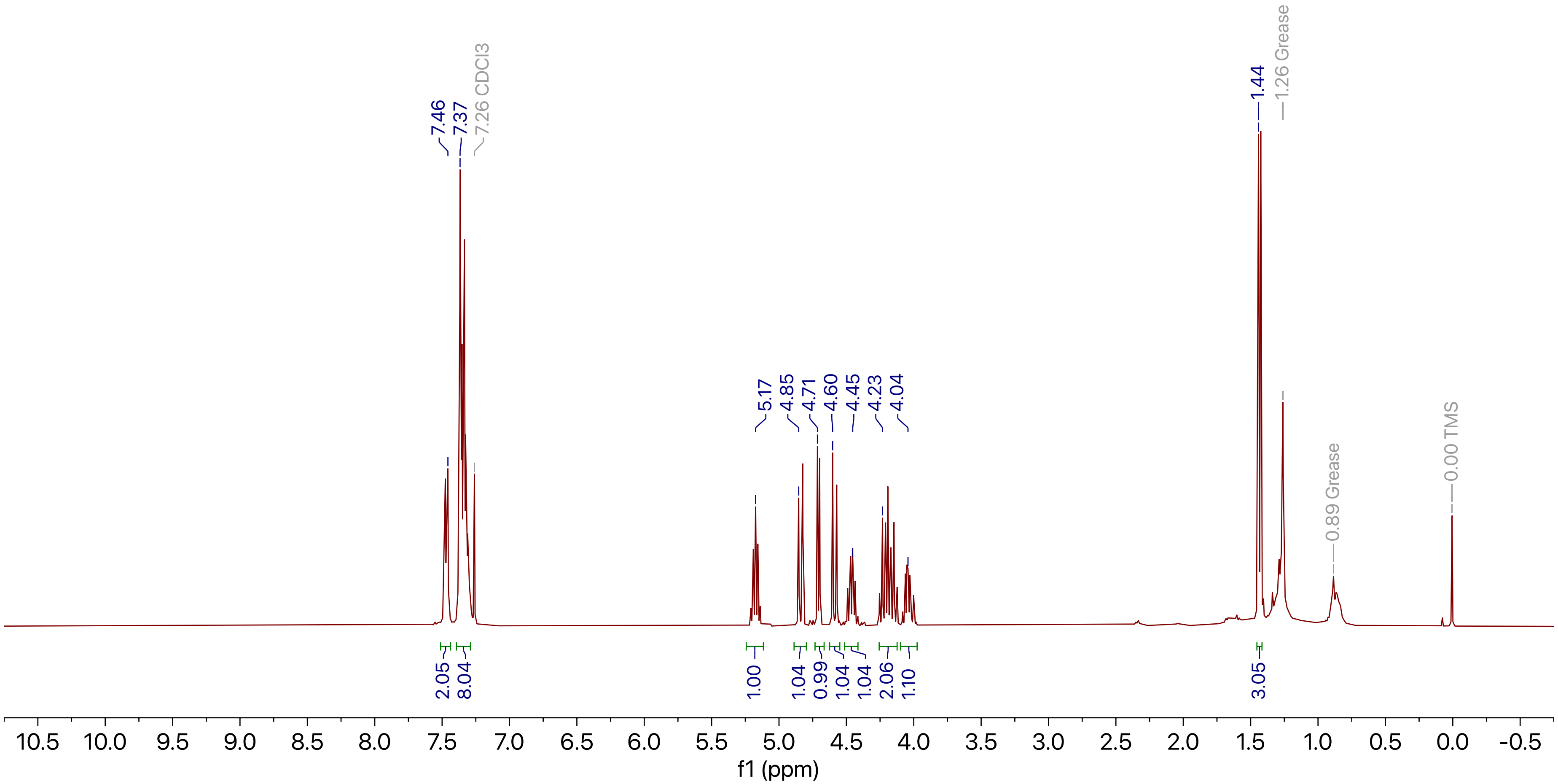

$^{13}C\{^1H\}$ NMR (101 MHz, $CDCl_3$)

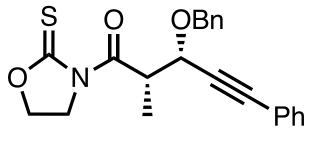

4c

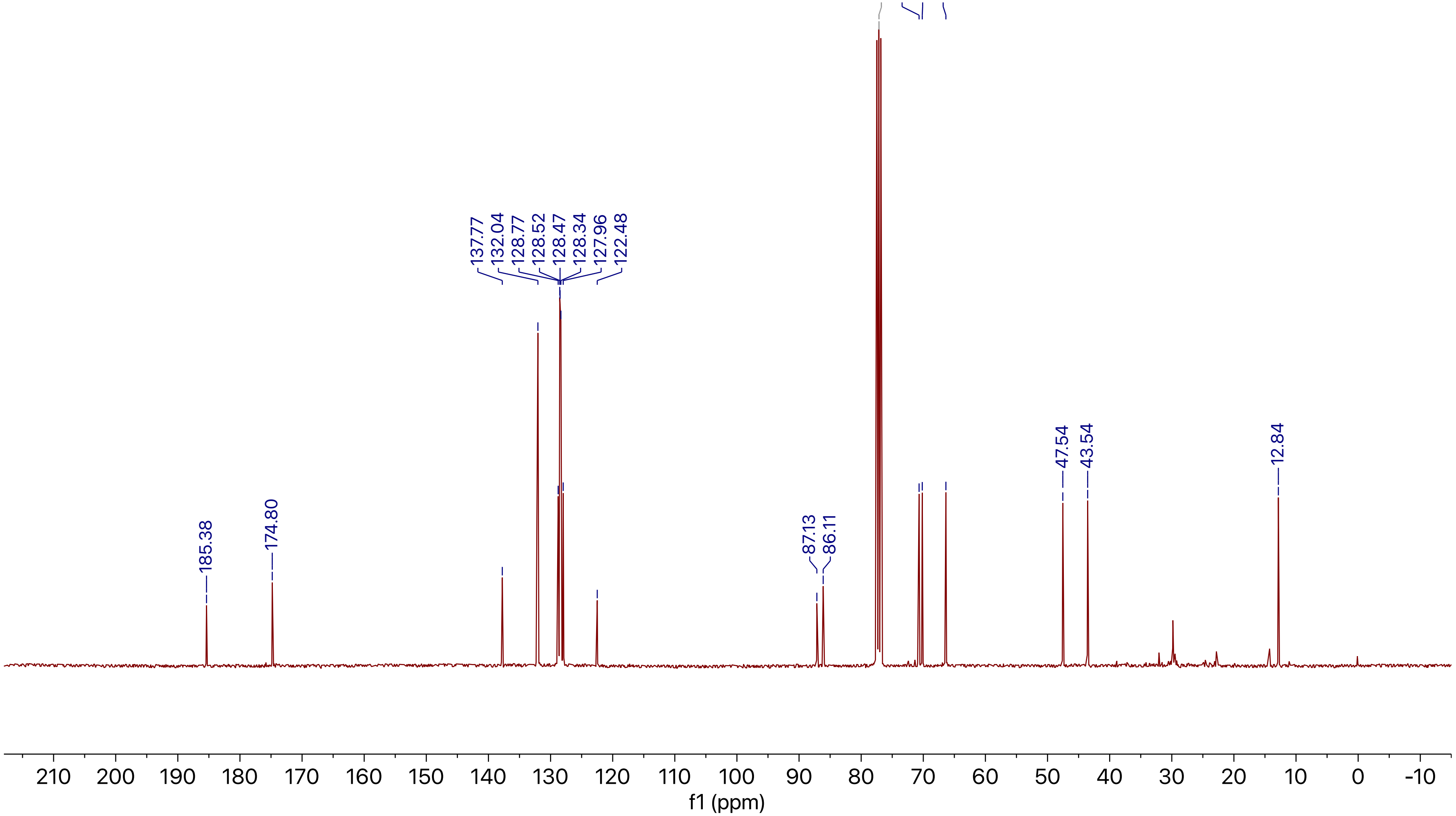

2D ^{1}H–^{1}H COSY (400 MHz, $CDCl_3$)

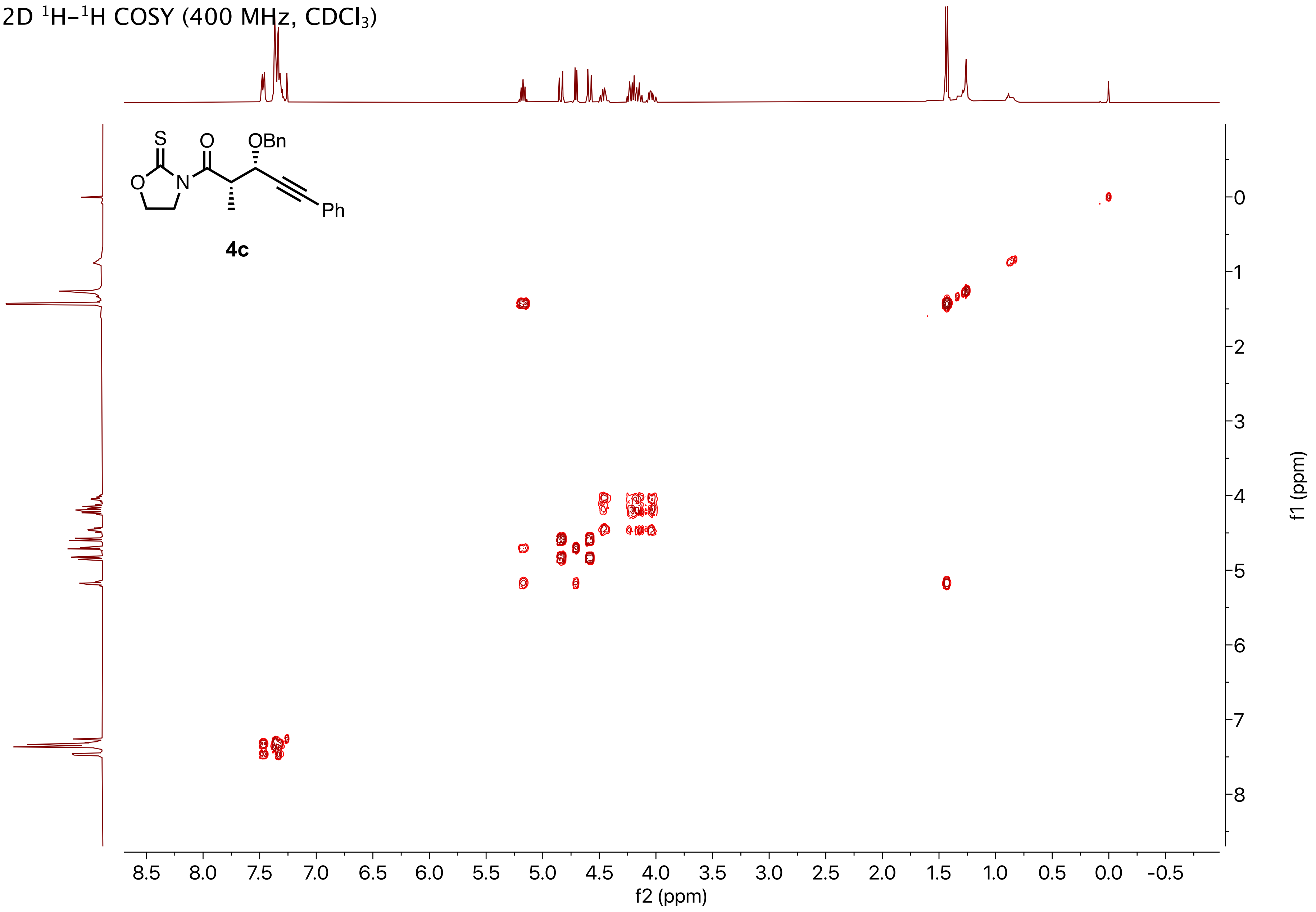

2D ^{1}H–^{13}C HSQC (400 MHz, $CDCl_3$)

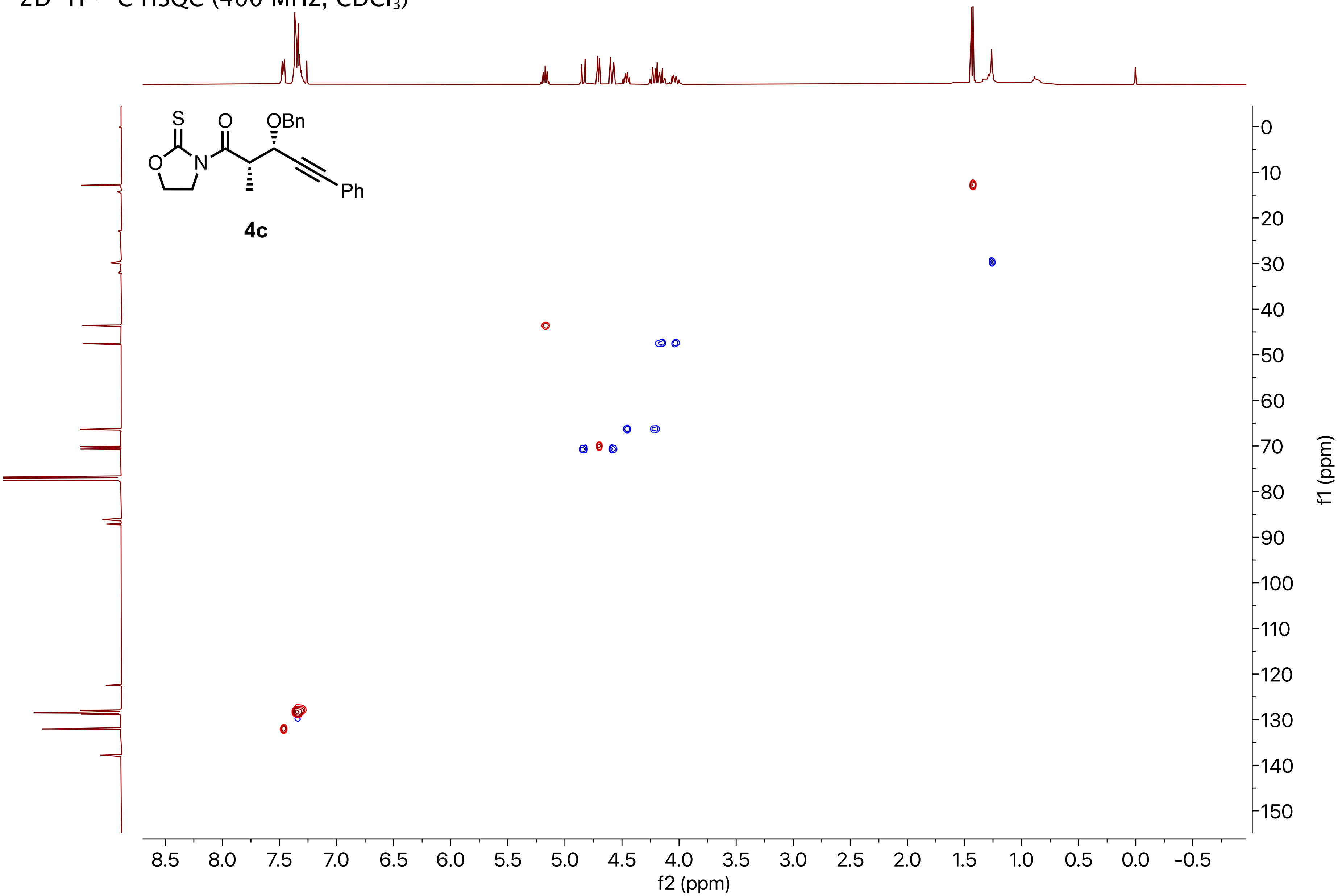

^{1}H NMR (400 MHz, $CDCl_3$)

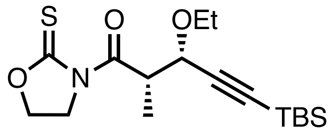

4d

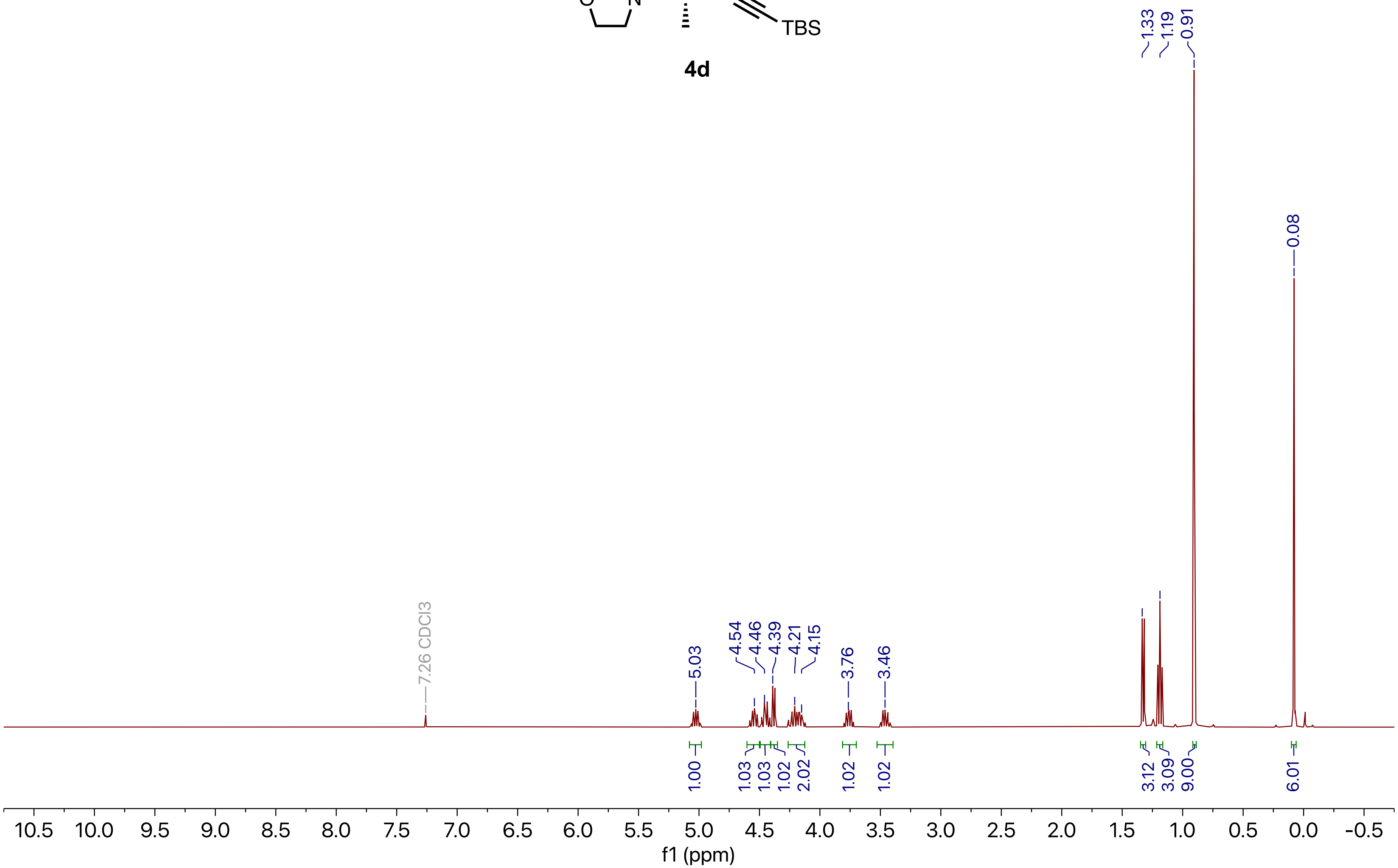

^{13}C NMR (101 MHz, $CDCl_3$)

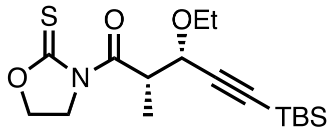

4d

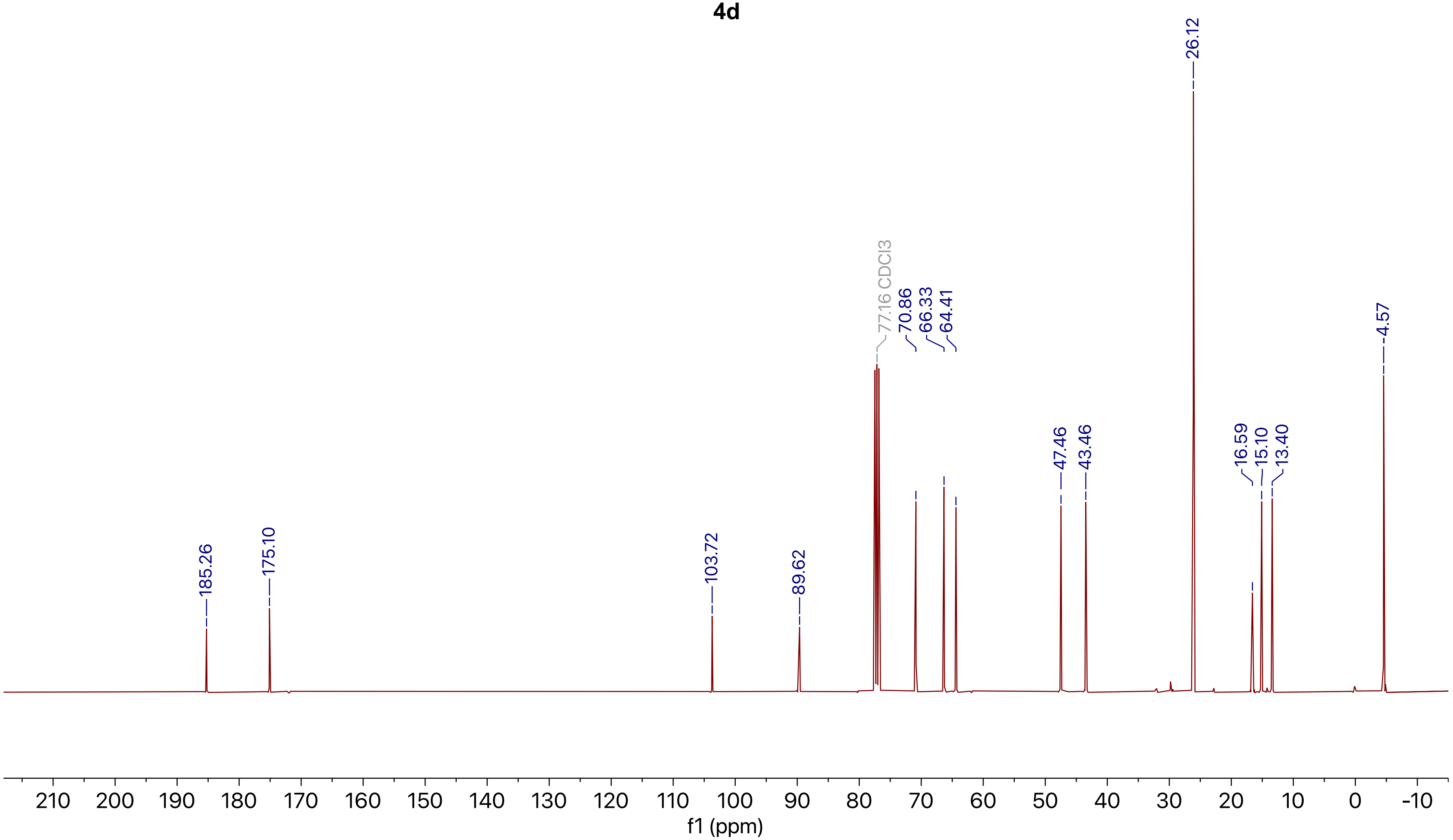

2D ^{1}H–^{1}H COSY (400 MHz, $CDCl_3$)

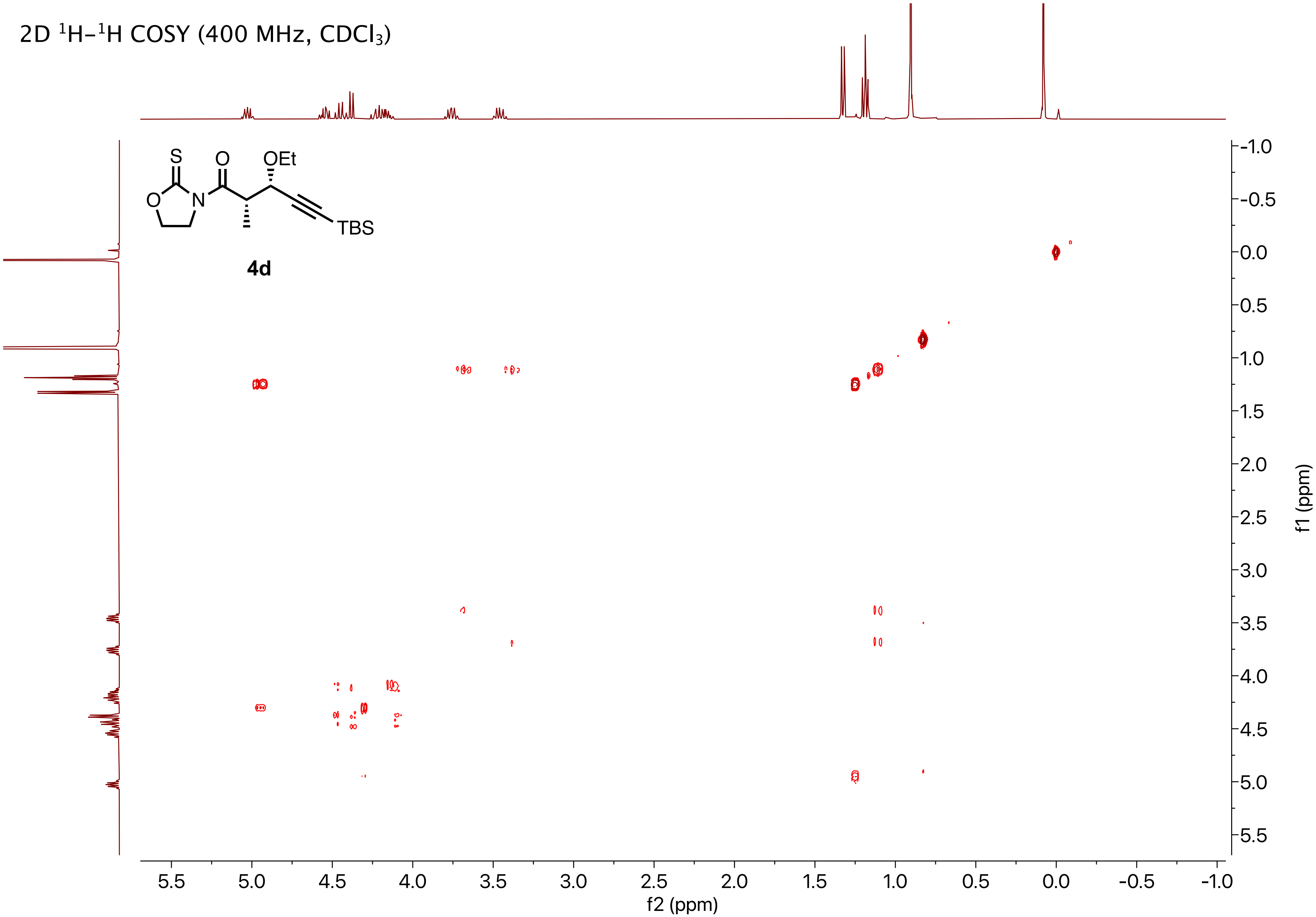

2D ^{1}H–^{13}C HSQC (400 MHz, $CDCl_3$)

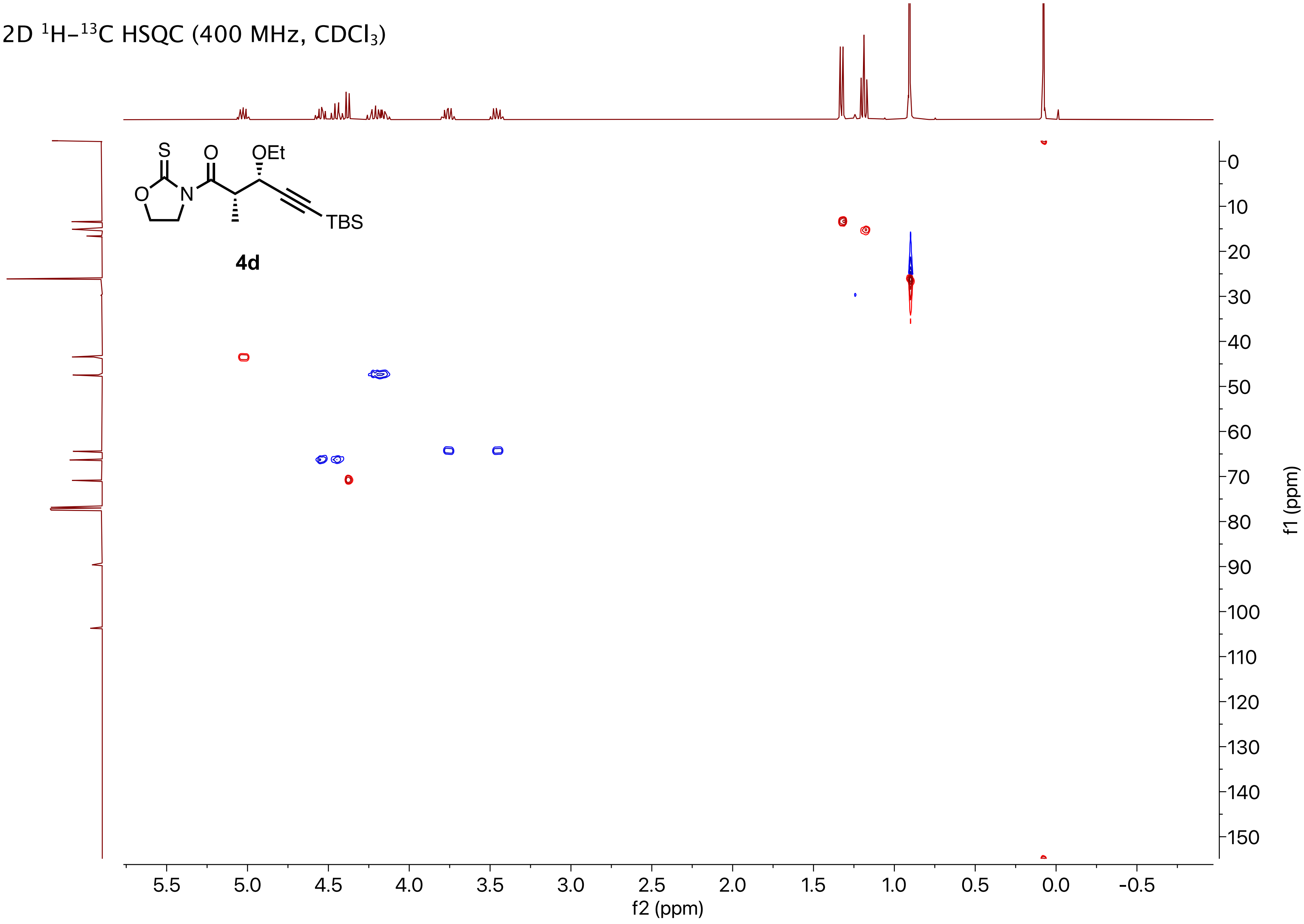

^{1}H NMR (400 MHz, $CDCl_3$)

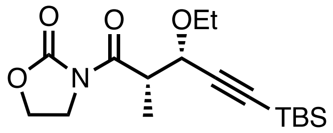

5d

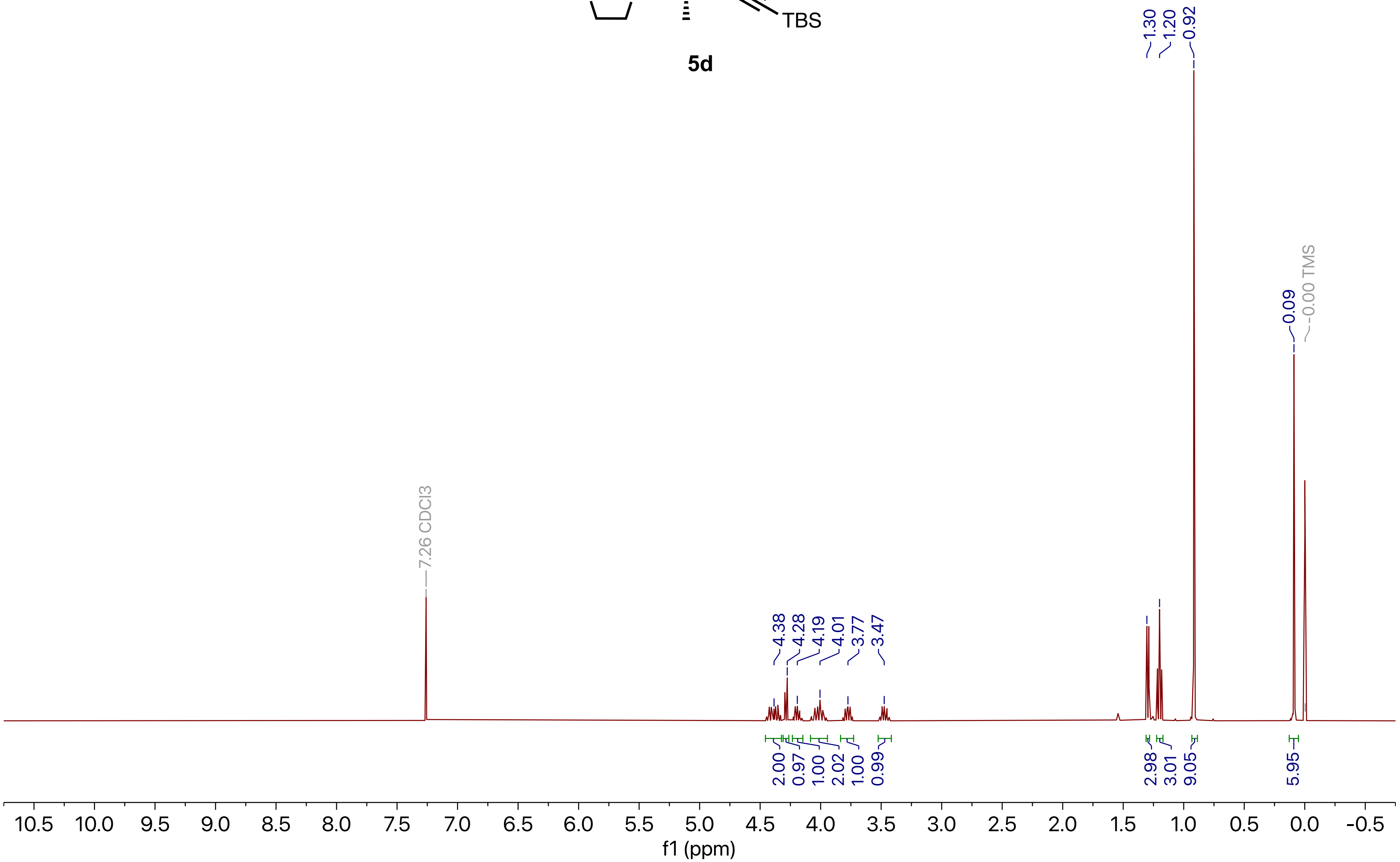

^{1}H NMR (400 MHz, $CDCl_3$)

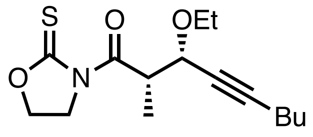

4e

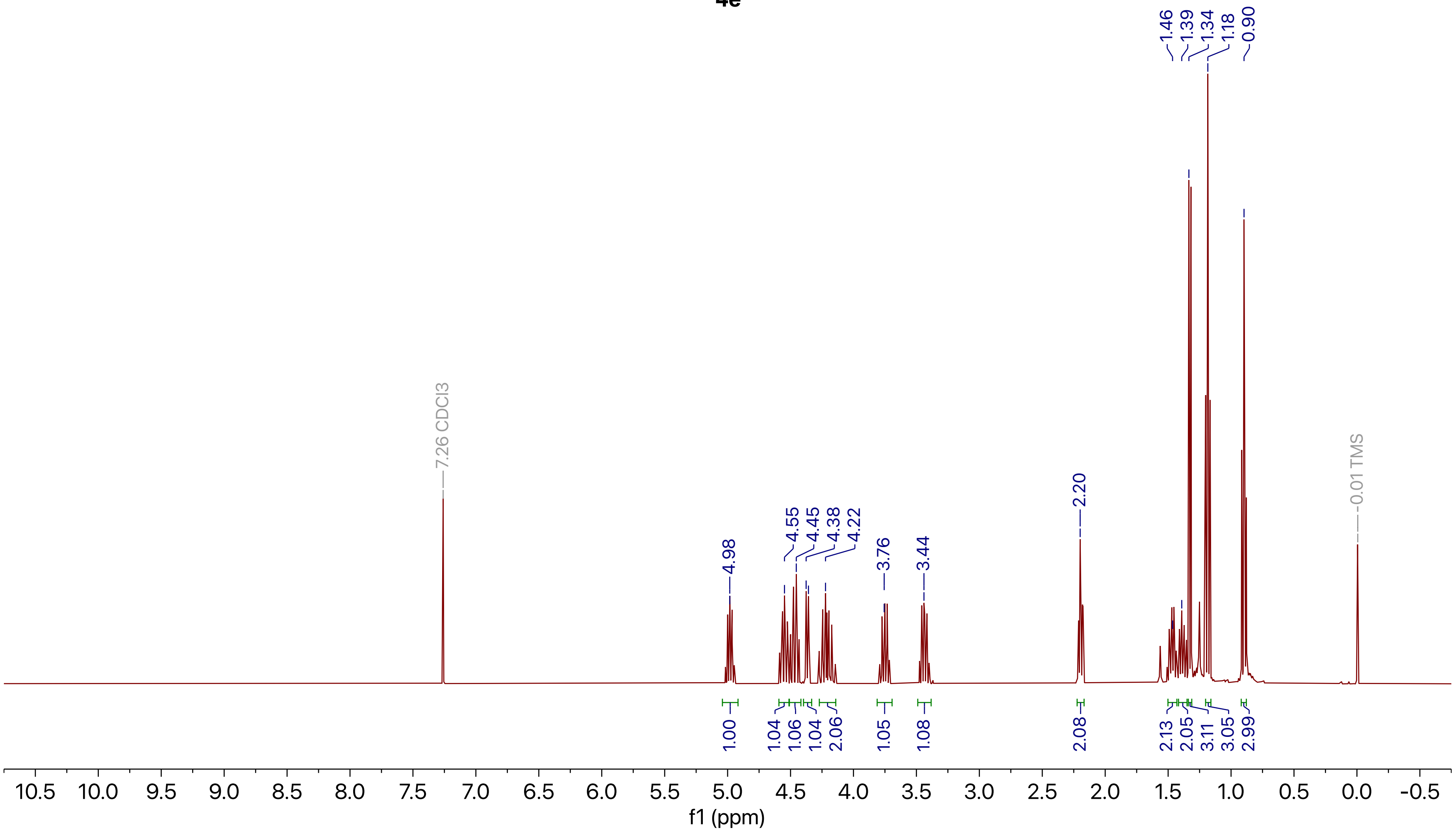

$^{13}C\{^{1}H\}$ NMR (101 MHz, $CDCl_3$)

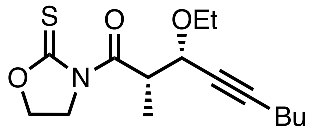

4e

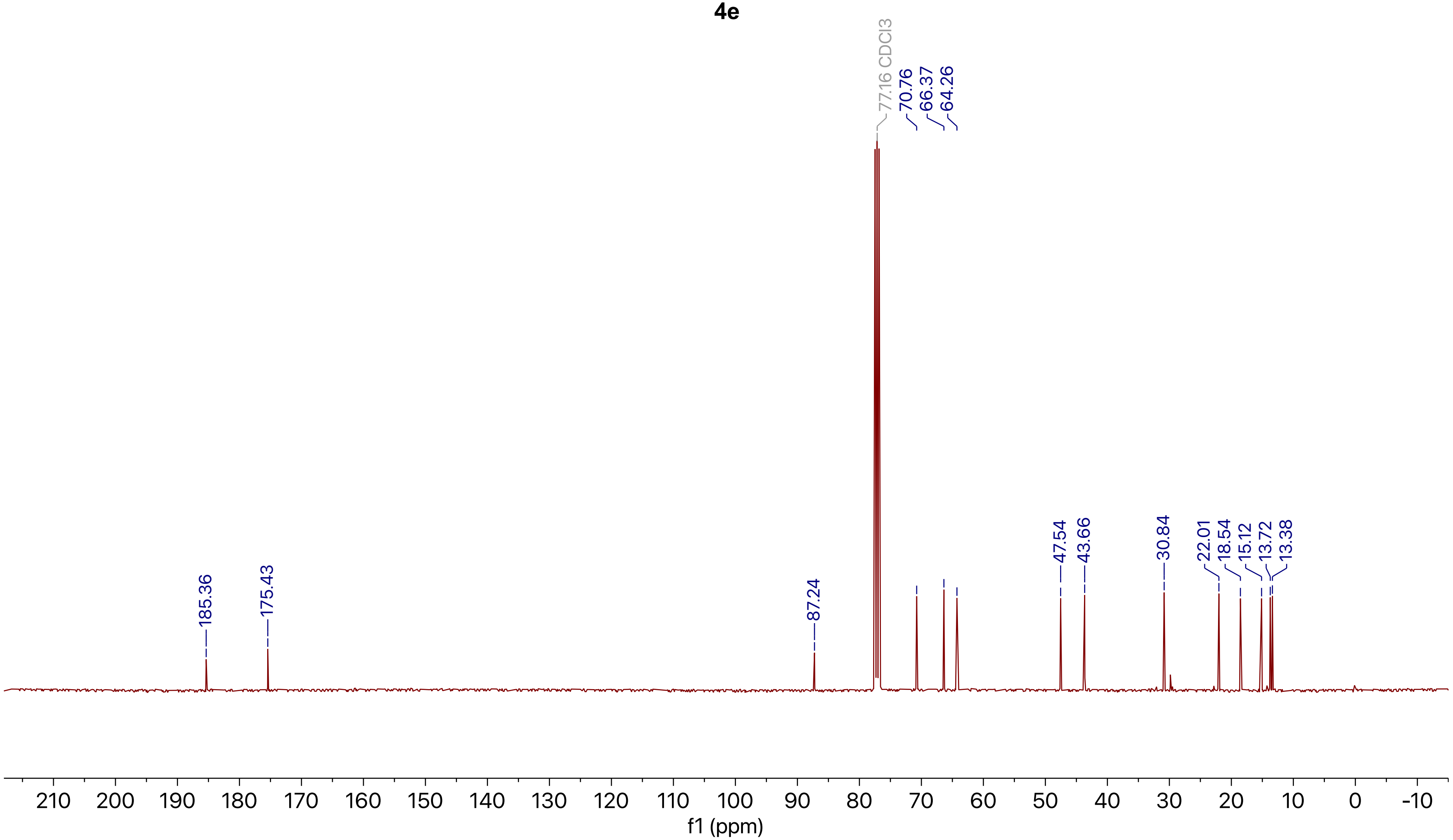

2D ^{1}H–^{1}H COSY (400 MHz, $CDCl_3$)

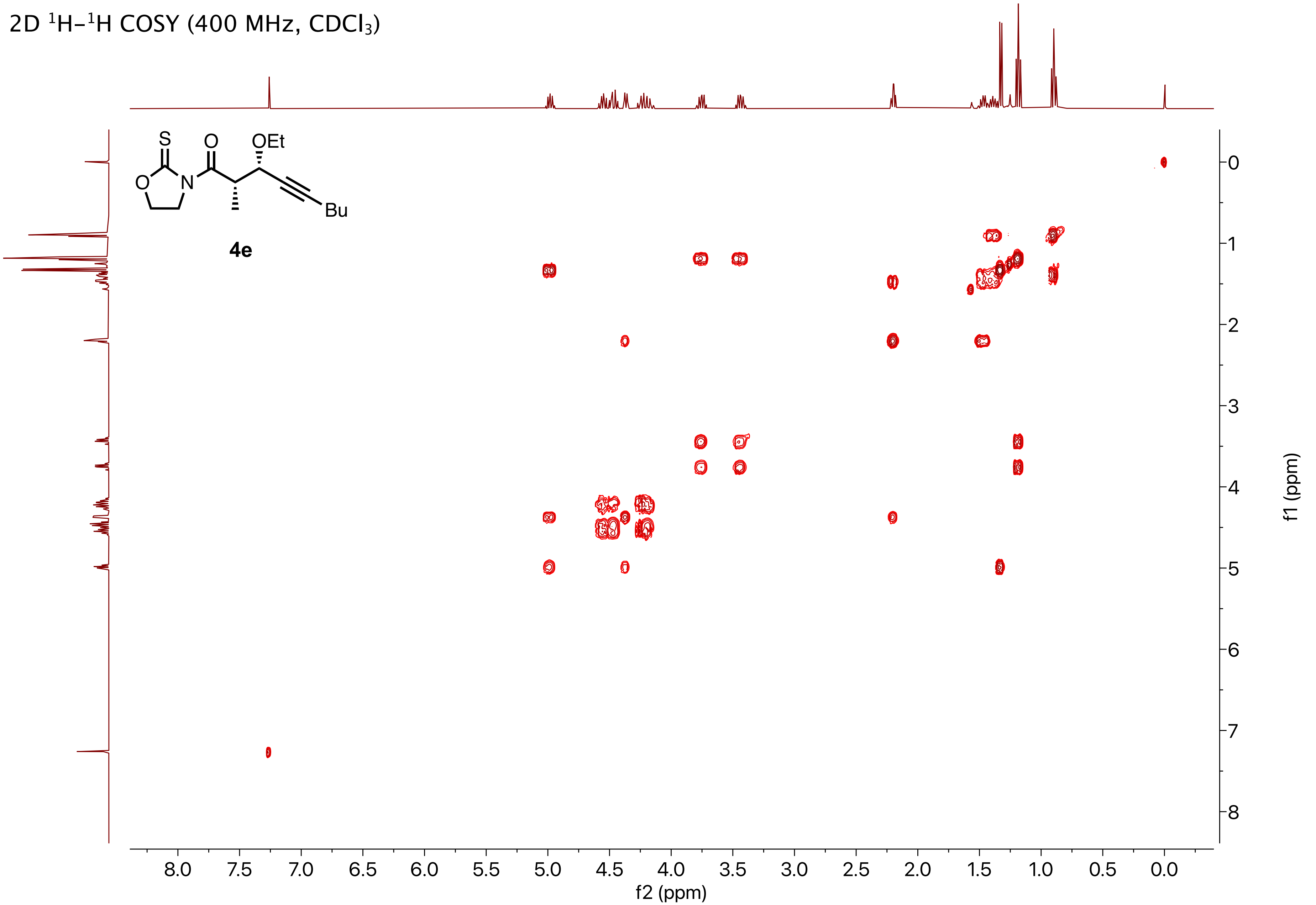

2D ^{1}H–^{13}C HSQC (400 MHz, $CDCl_3$)

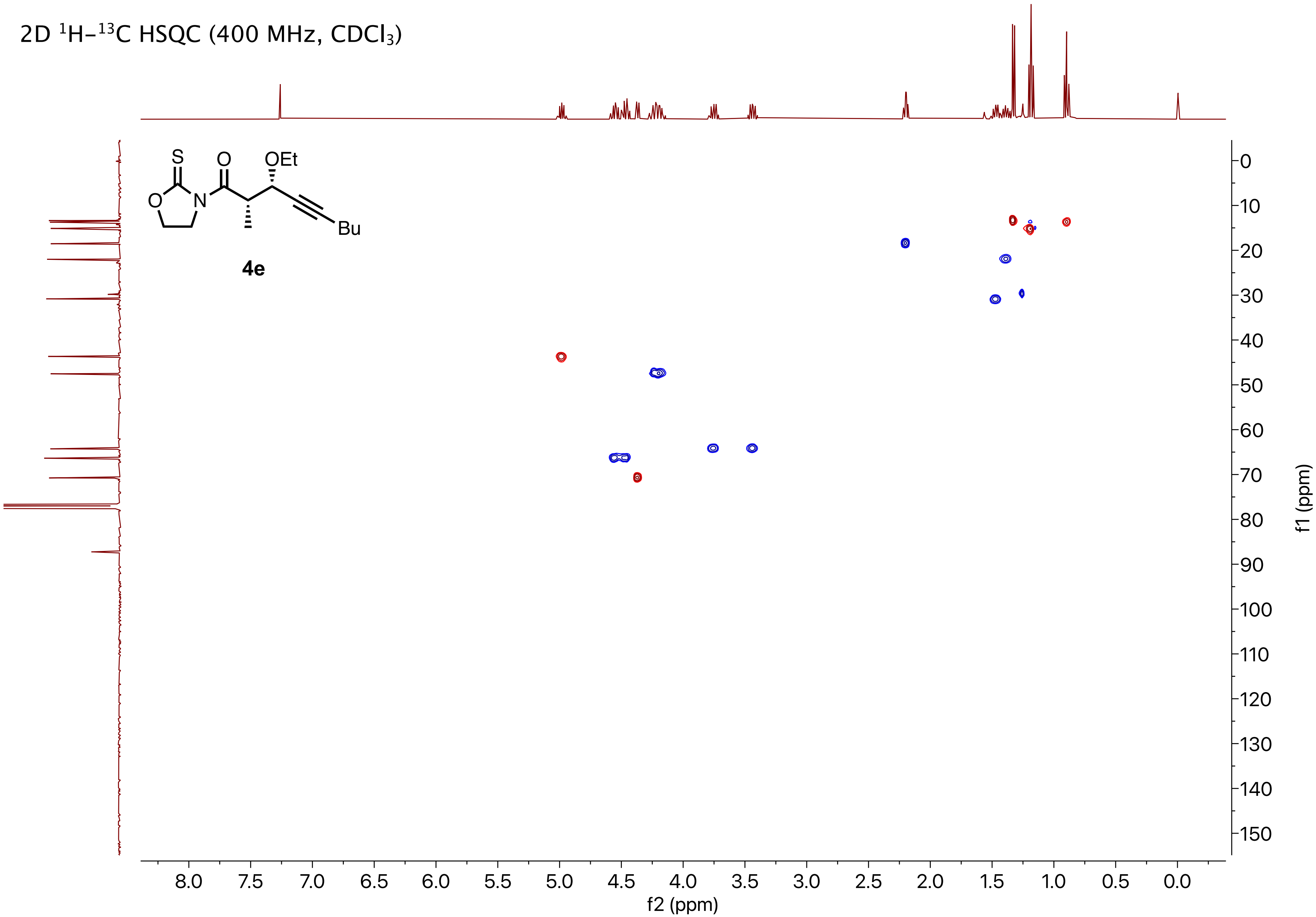

^{1}H NMR (400 MHz, $CDCl_3$)

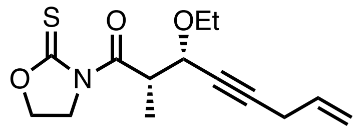

4f

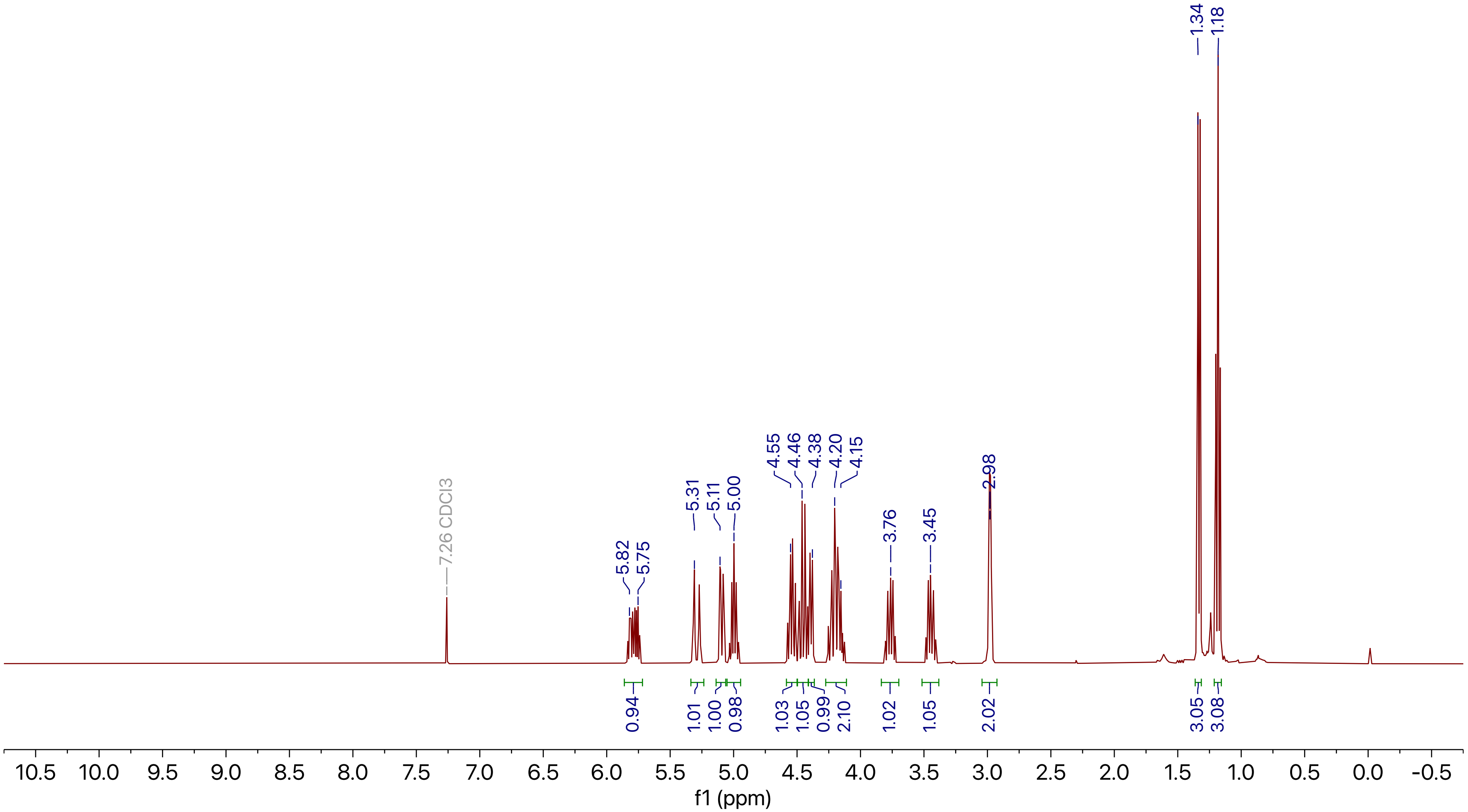

$^{13}C\{^{1}H\}$ NMR (101 MHz, $CDCl_3$)

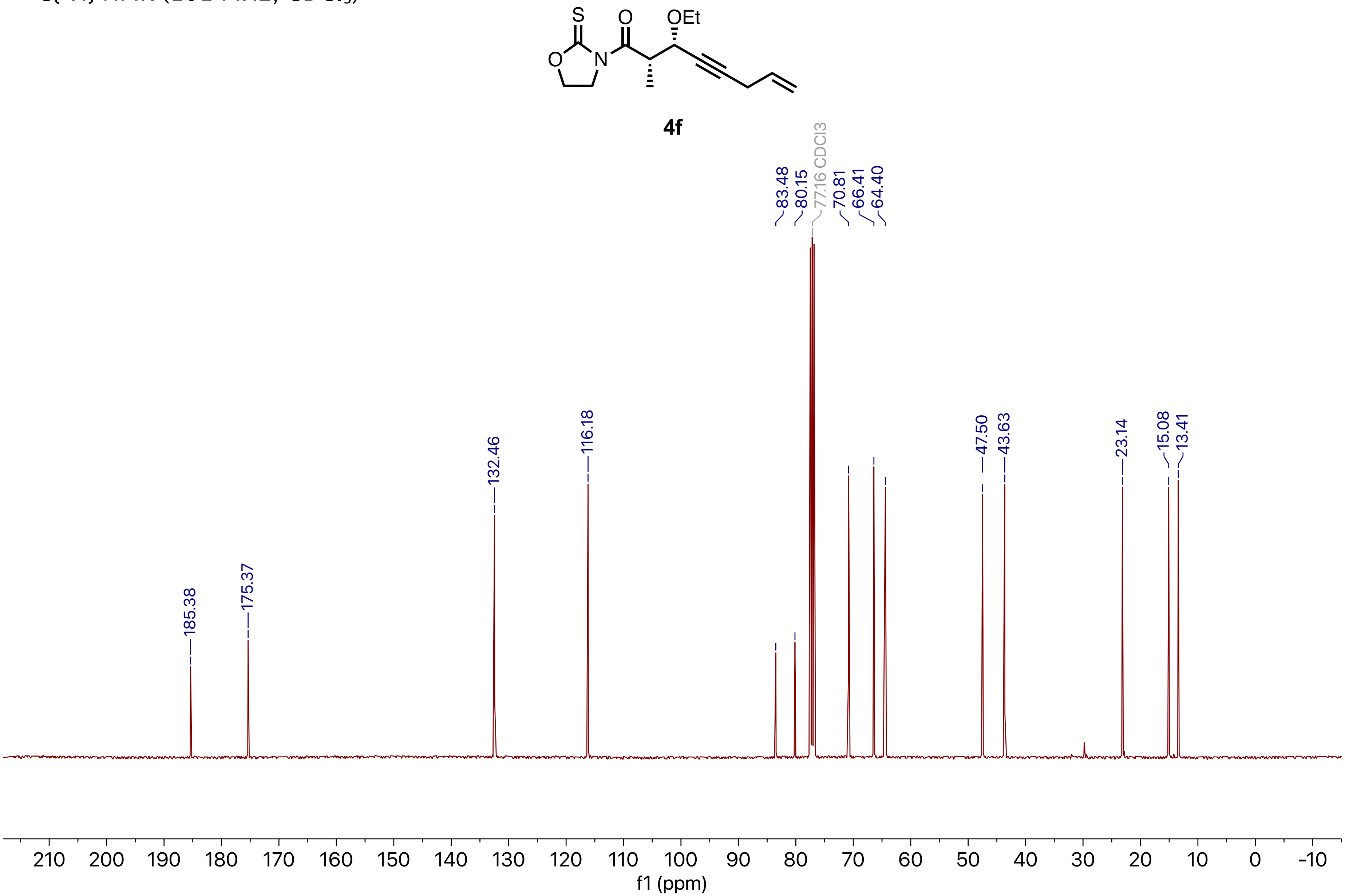

2D ^{1}H–^{1}H COSY (400 MHz, $CDCl_3$)

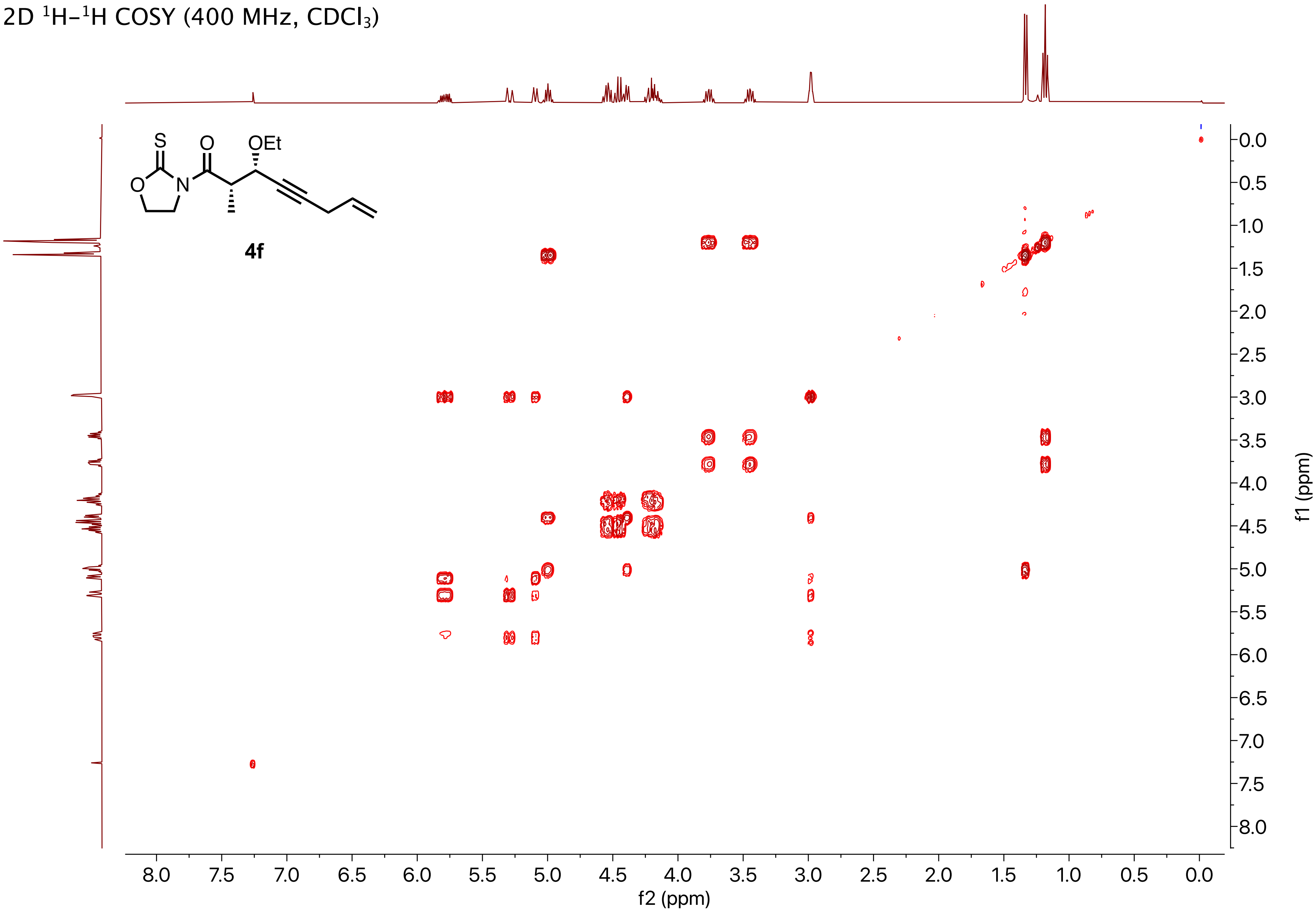

2D ^{1}H–^{13}C HSQC (400 MHz, $CDCl_3$)

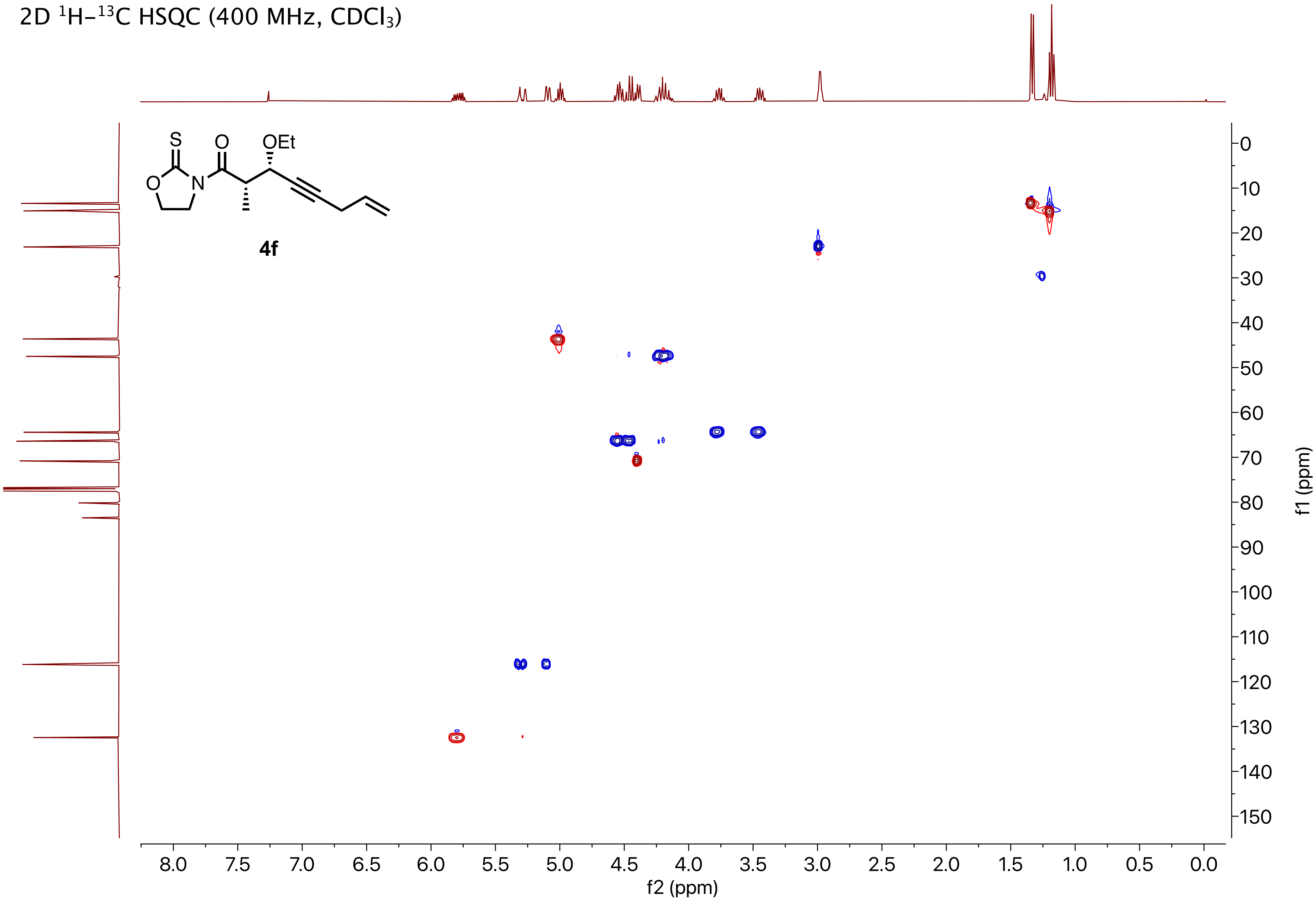

^{1}H NMR (400 MHz, $CDCl_3$)

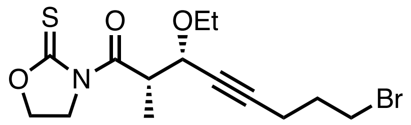

4g

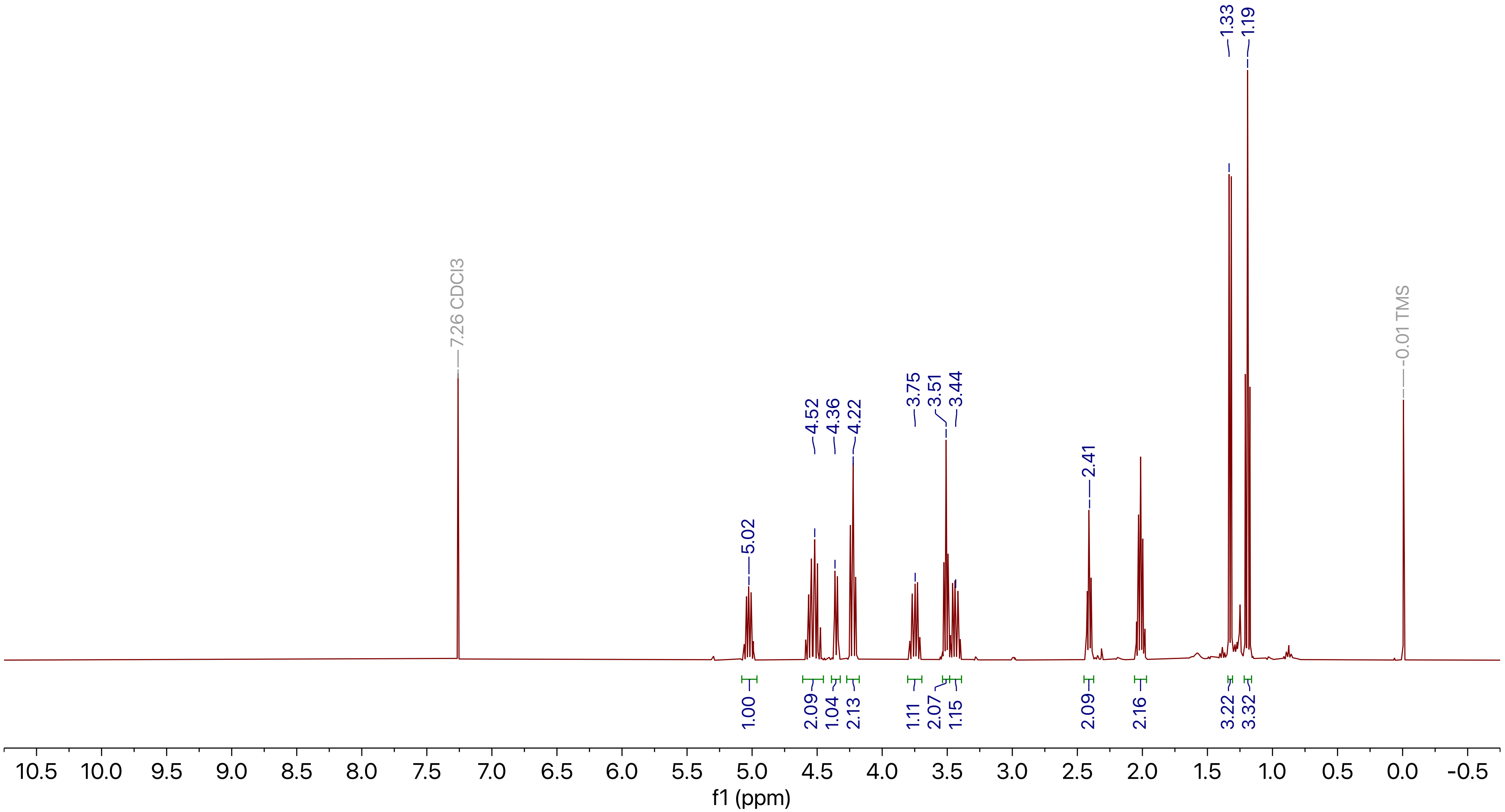

$^{13}C\{^{1}H\}$ NMR (101 MHz, $CDCl_3$)

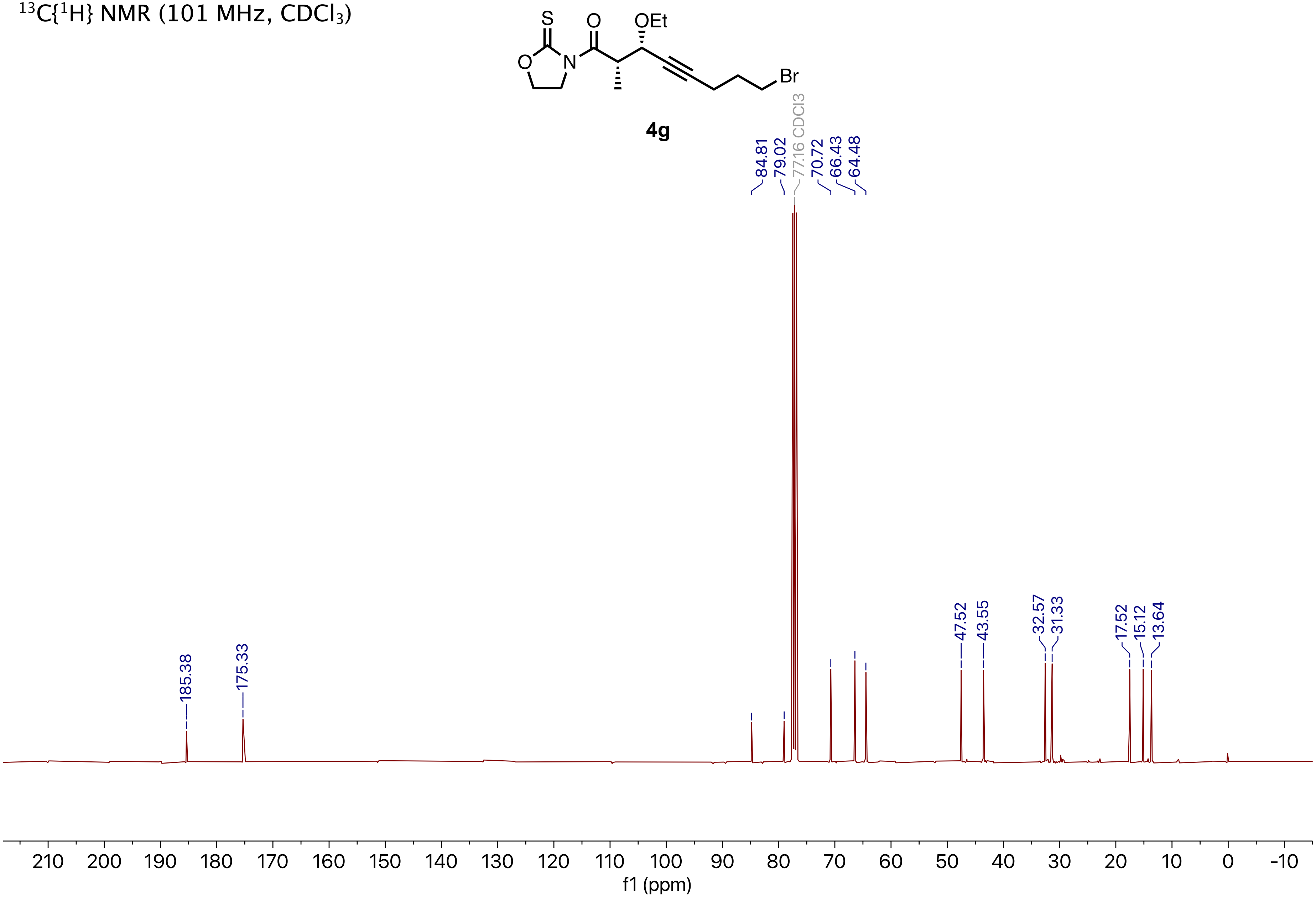

2D ^{1}H–^{1}H COSY (400 MHz, $CDCl_3$)

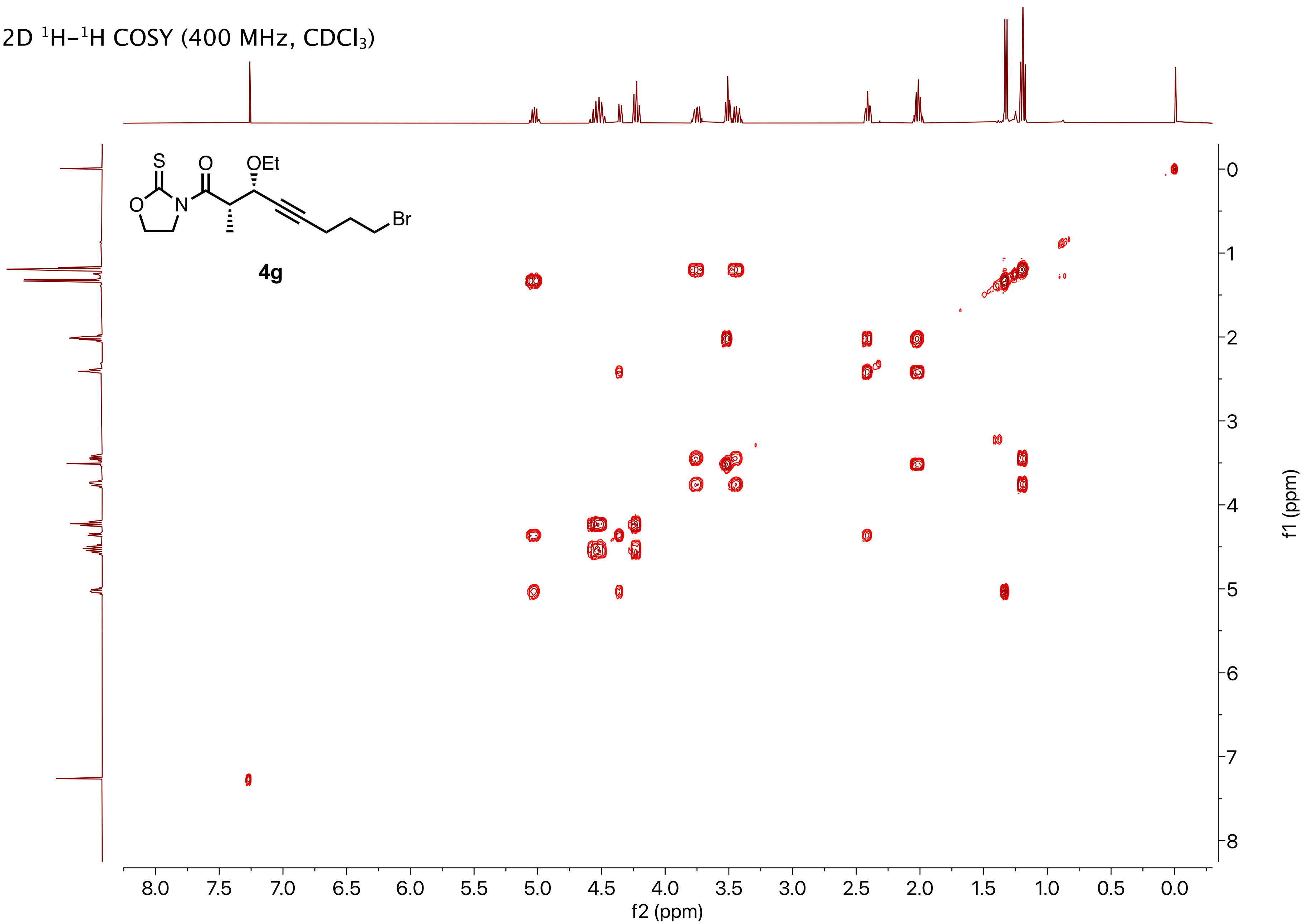

2D ^{1}H–^{13}C HSQC (400 MHz, $CDCl_3$)

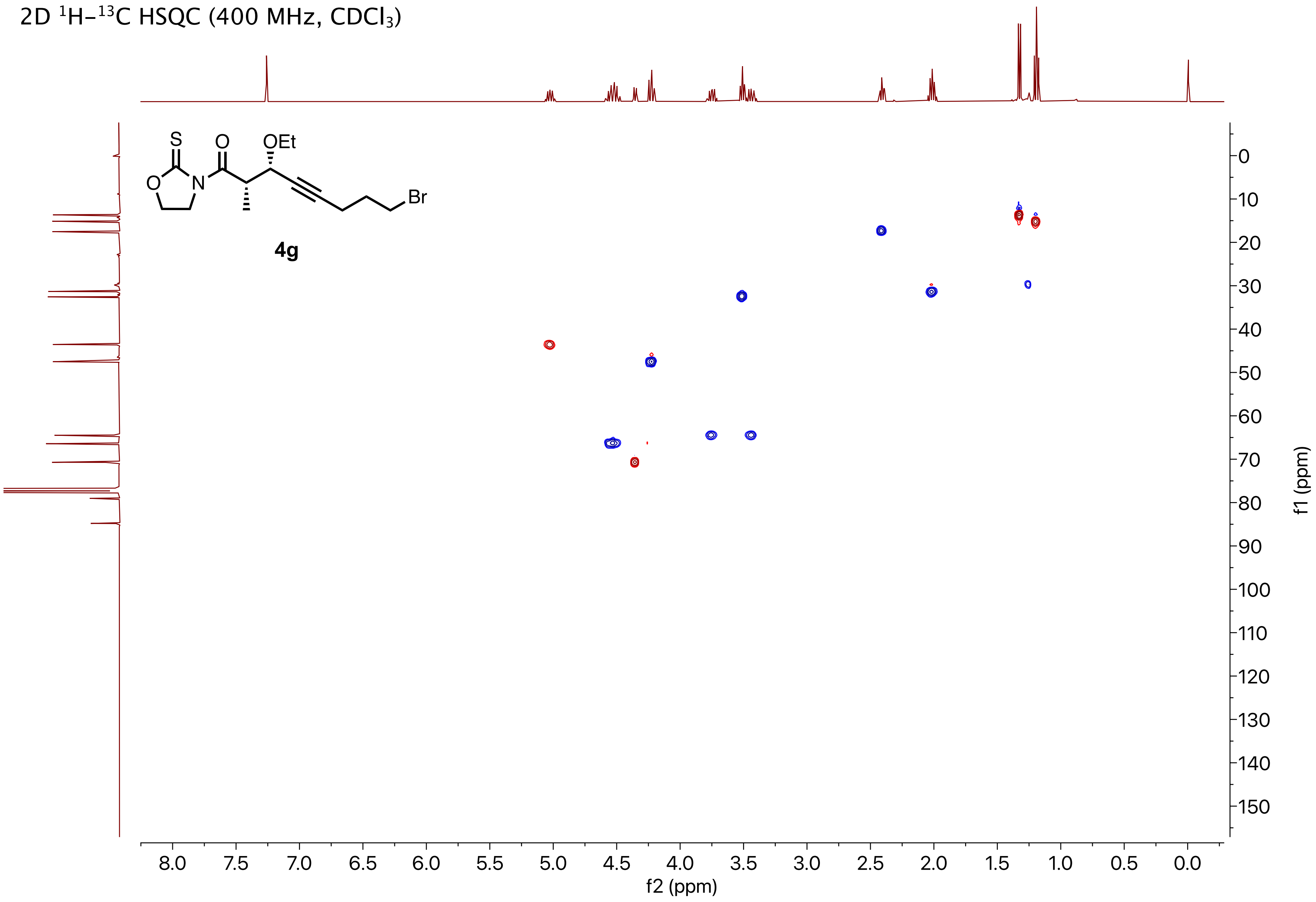

^{1}H NMR (400 MHz, $CDCl_3$)

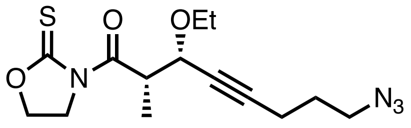

4h

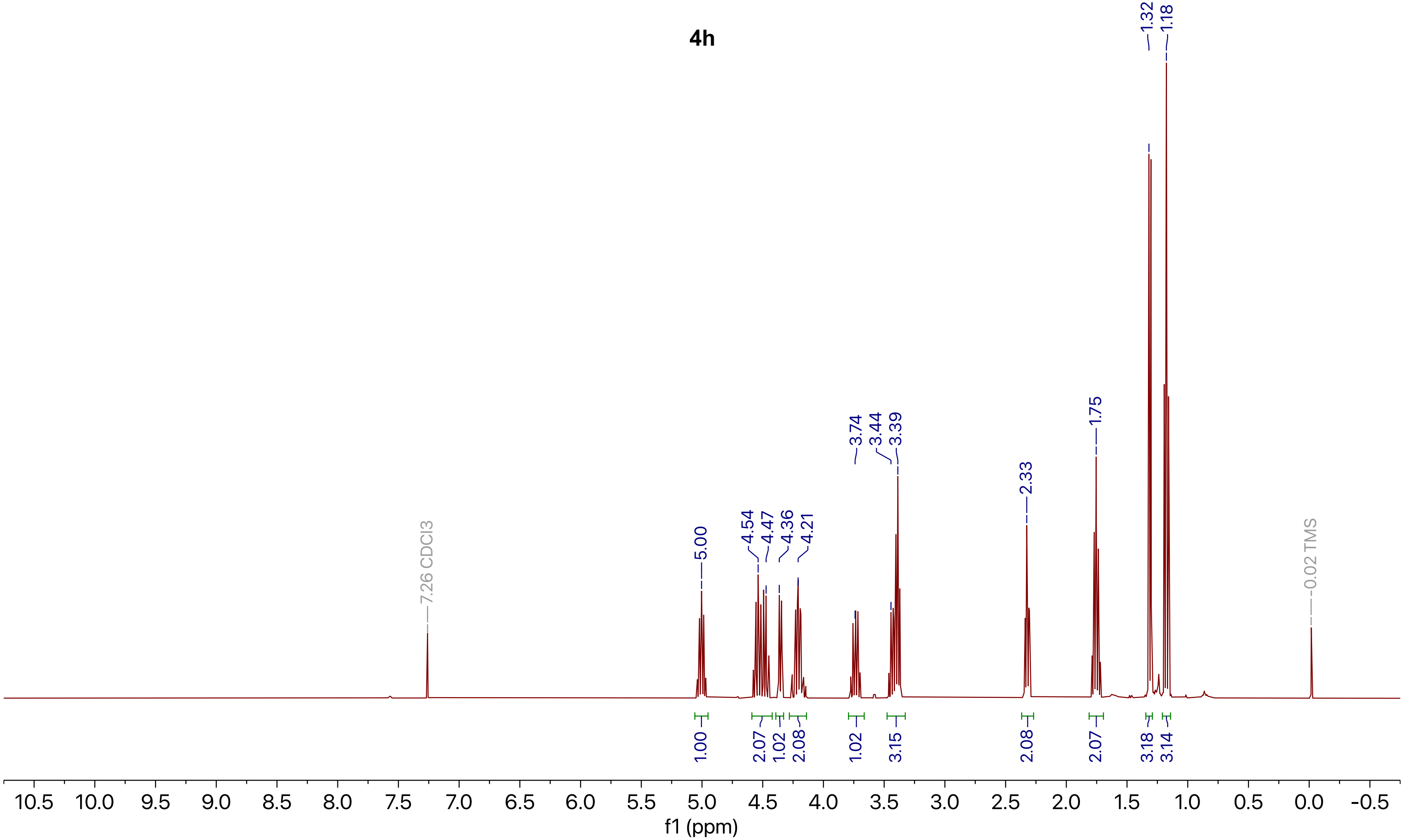

$^{13}C\{^1H\}$ NMR (101 MHz, $CDCl_3$)

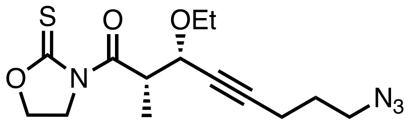

4h

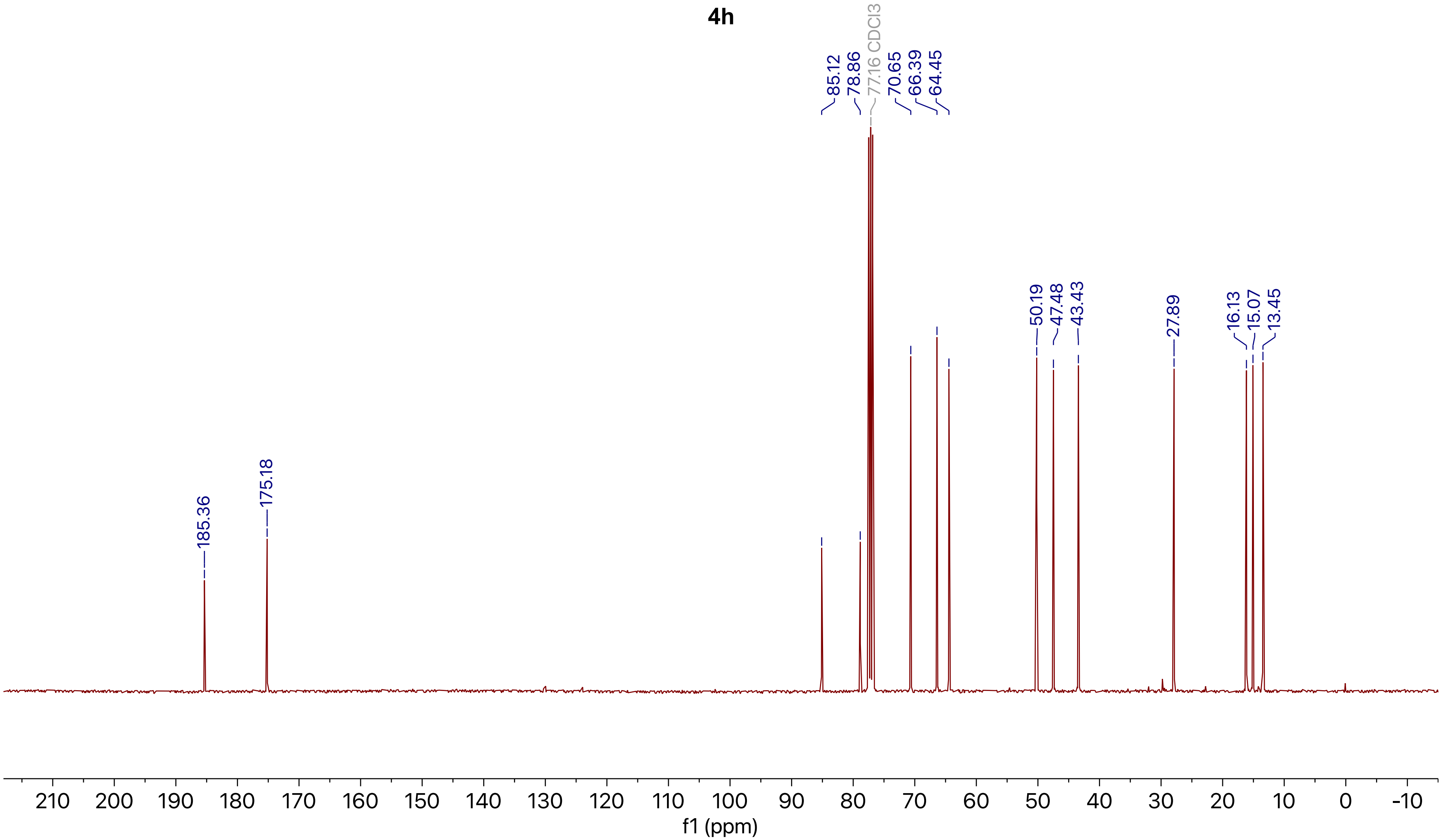

2D ^{1}H–^{1}H COSY (400 MHz, $CDCl_3$)

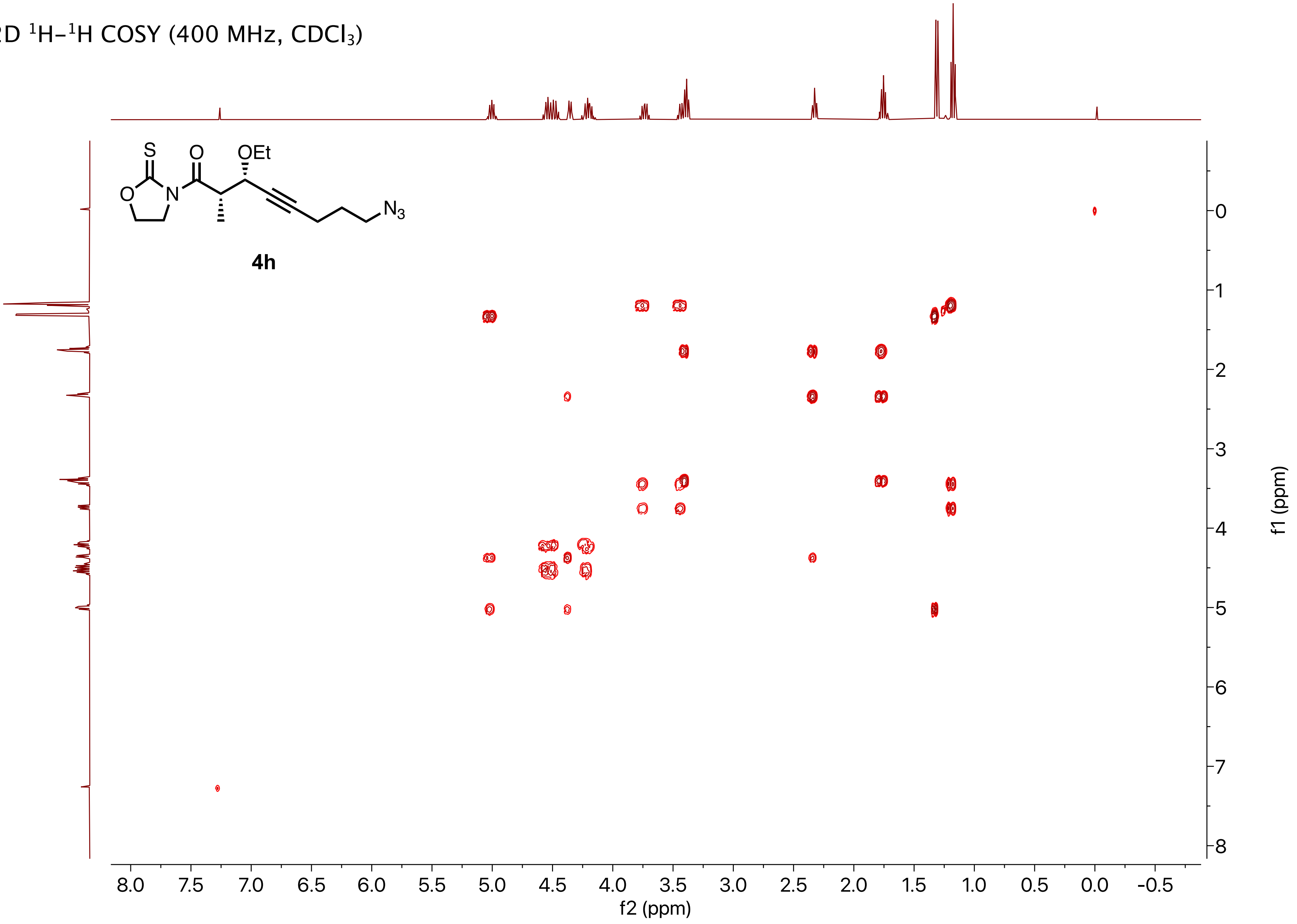

2D ^{1}H–^{13}C HSQC (400 MHz, $CDCl_3$)

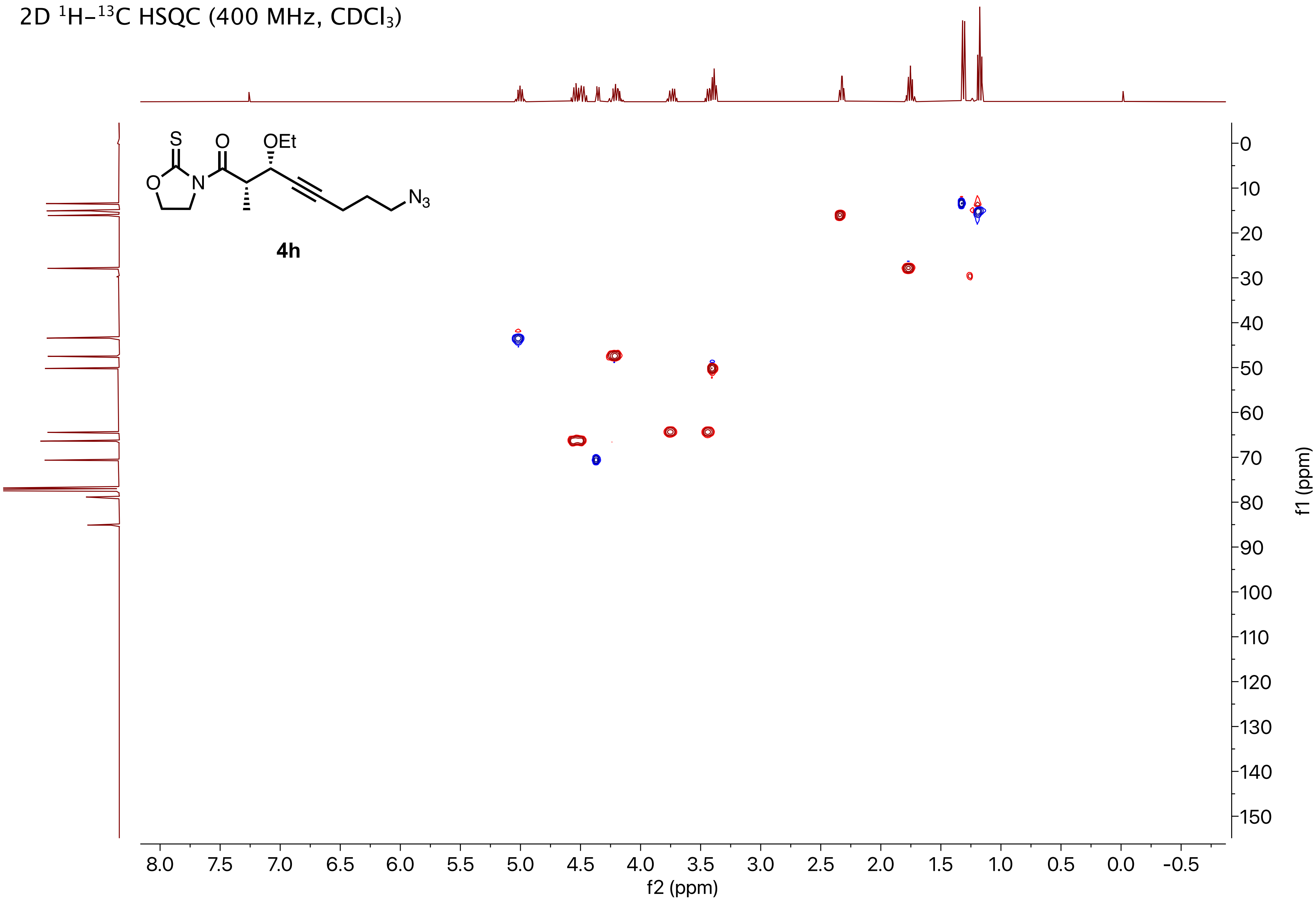

^{1}H NMR (400 MHz, $CDCl_3$)

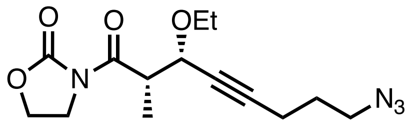

5h

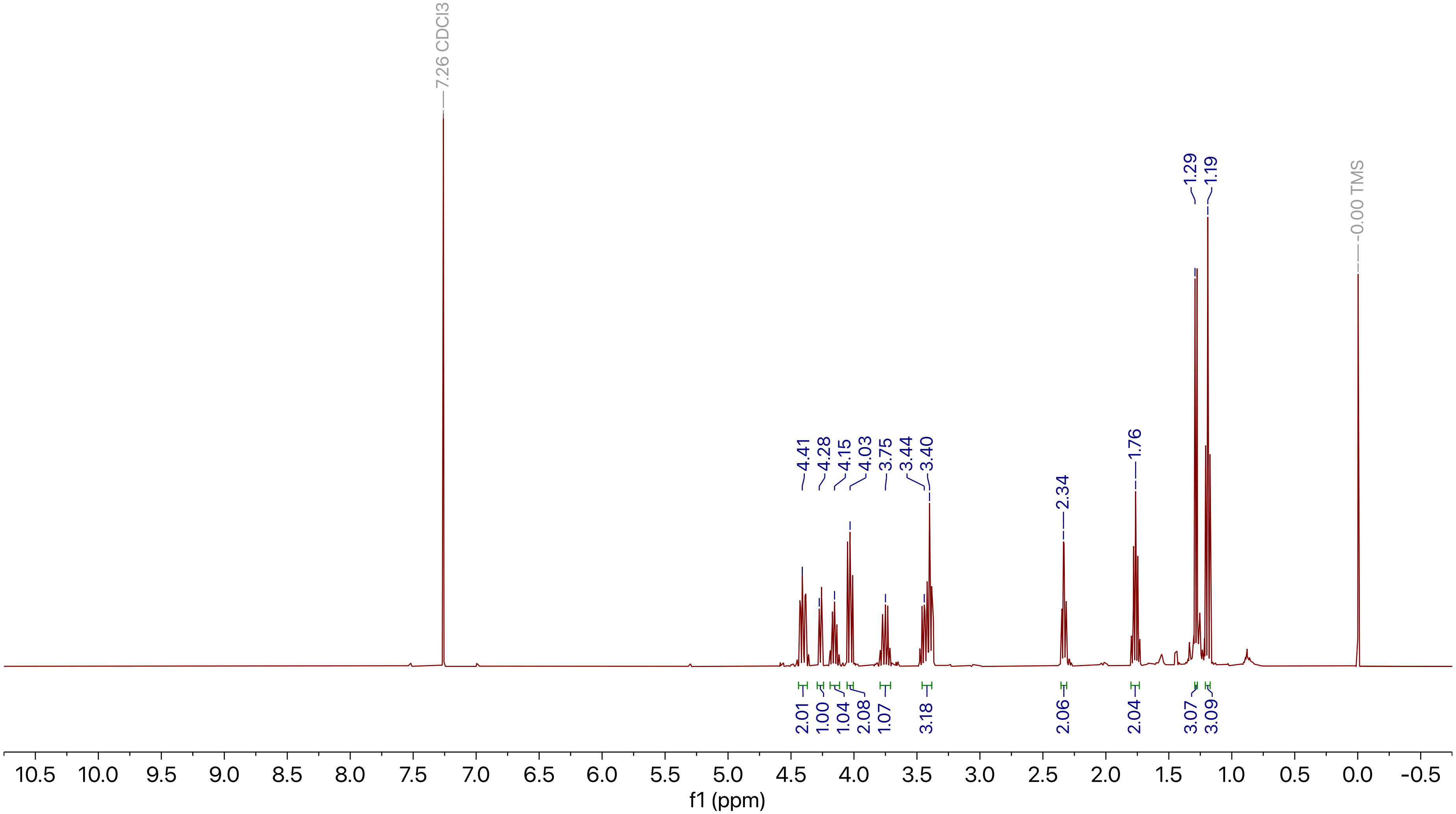

^{1}H NMR (400 MHz, $CDCl_3$)

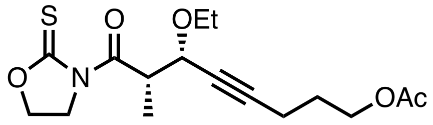

4i

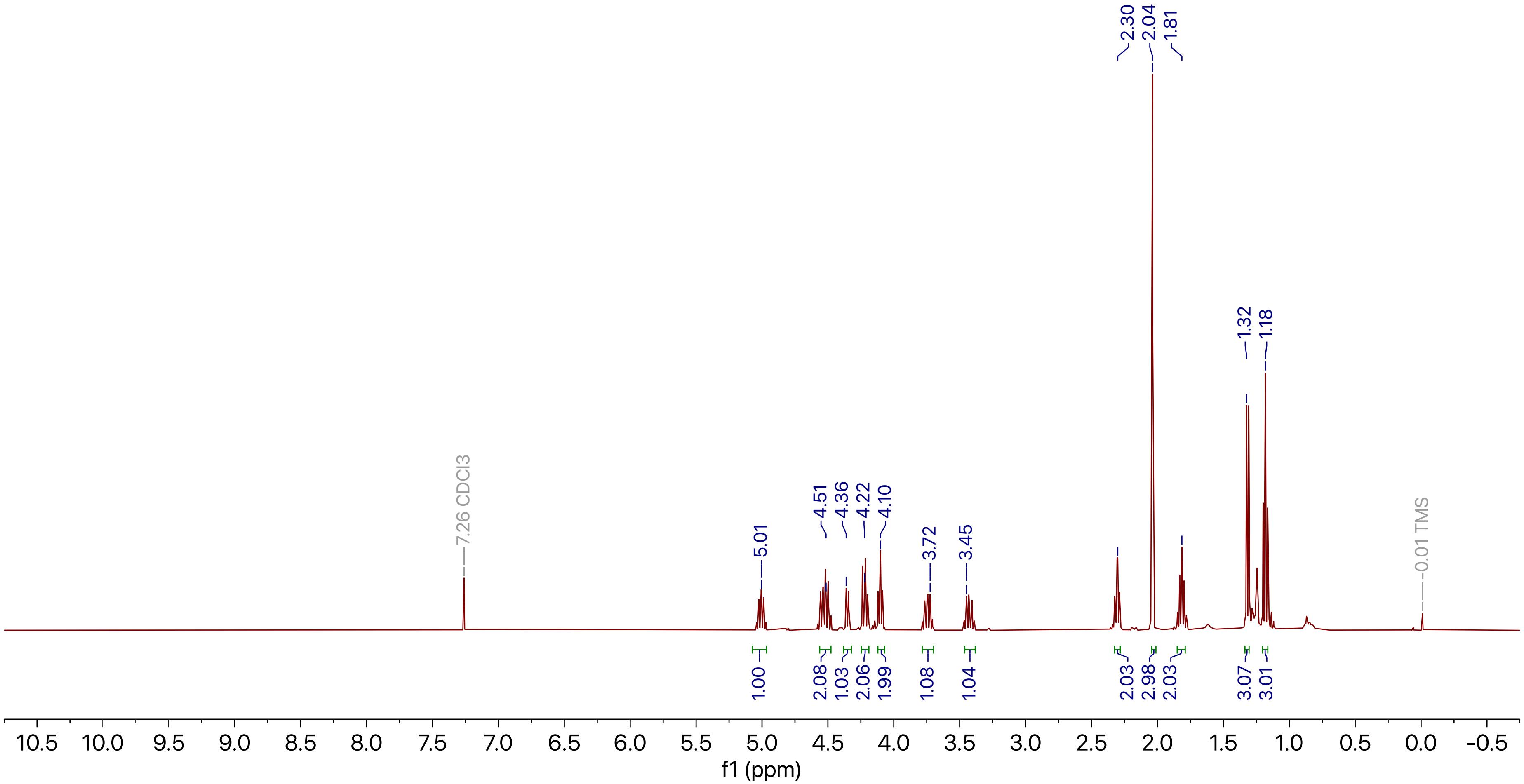

$^{13}C\{^{1}H\}$ NMR (101 MHz, $CDCl_3$)

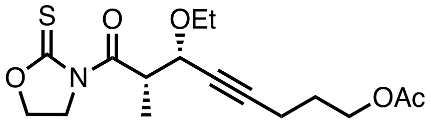

4i

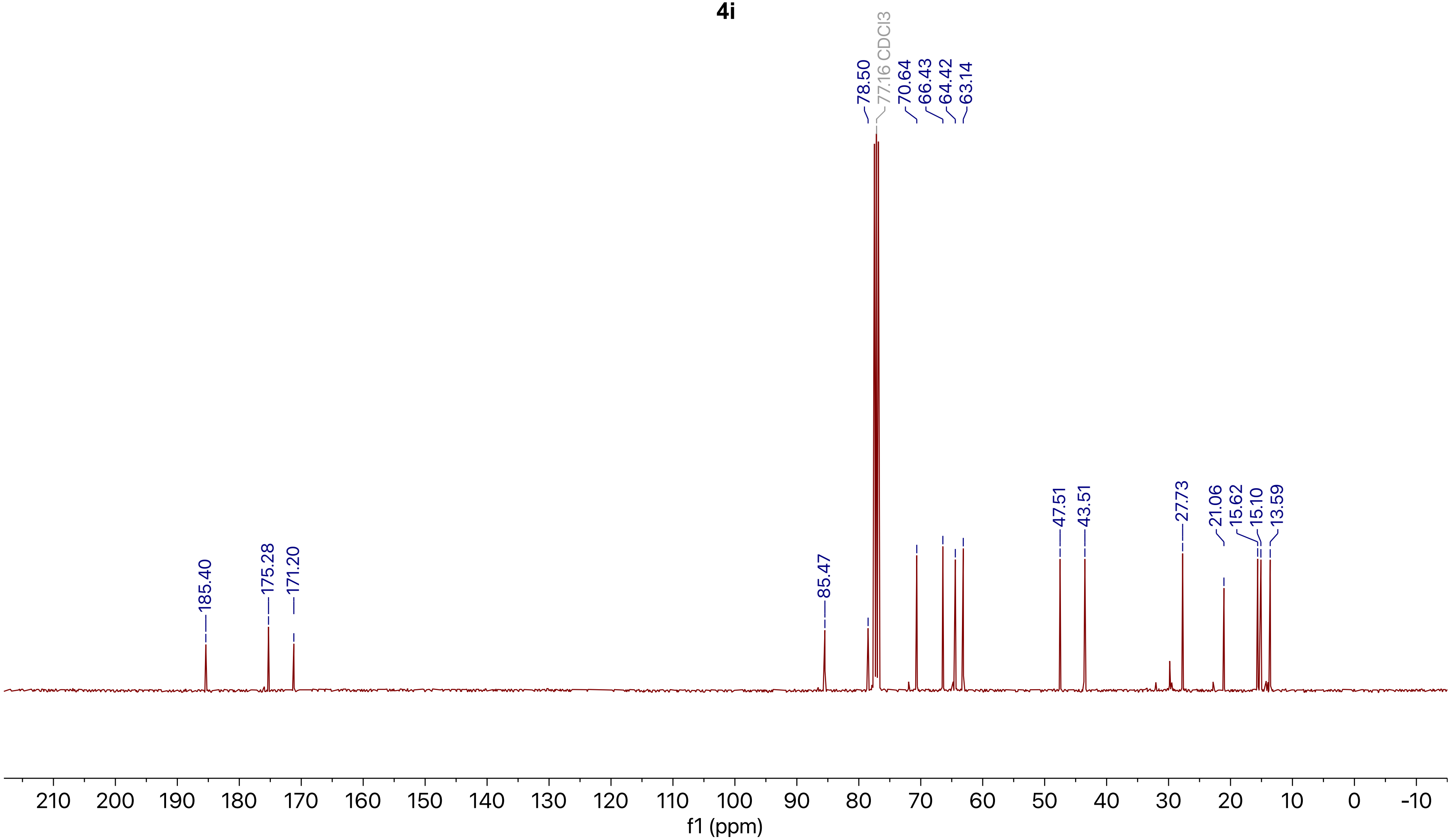

2D ^{1}H–^{1}H COSY (400 MHz, $CDCl_3$)

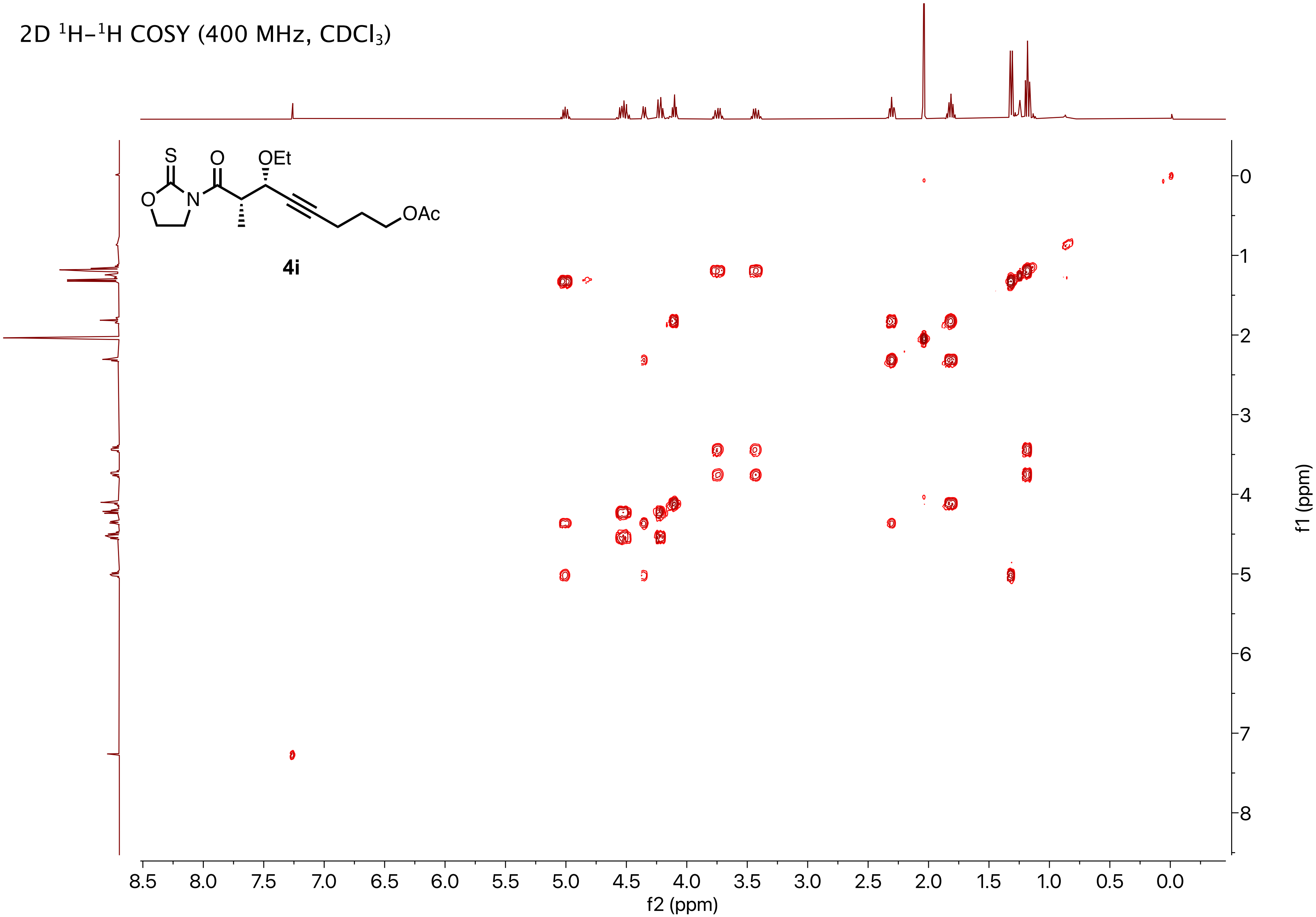

2D ^{1}H–^{13}C HSQC (400 MHz, $CDCl_3$)

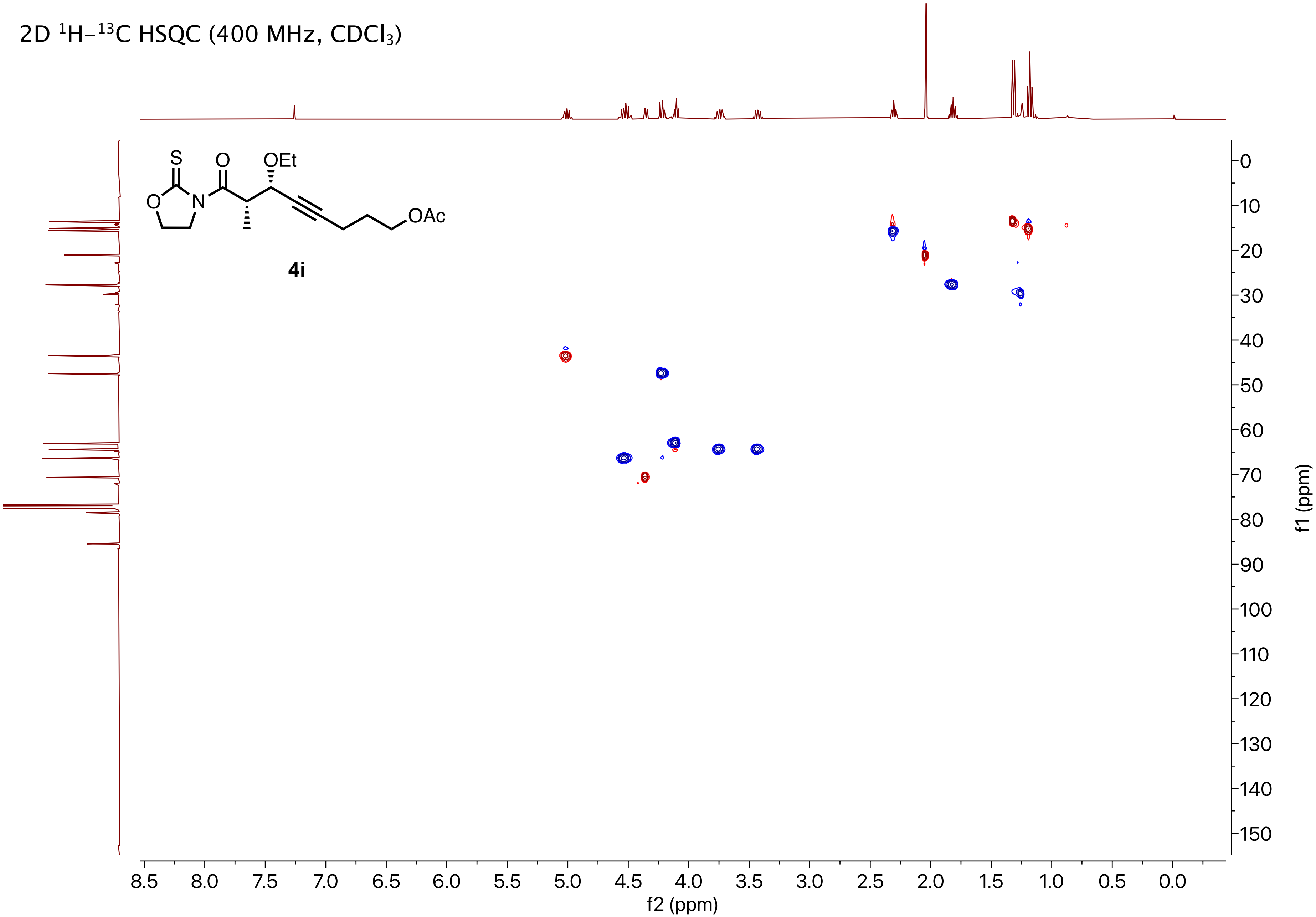

^{1}H NMR (500 MHz, $CDCl_3$)

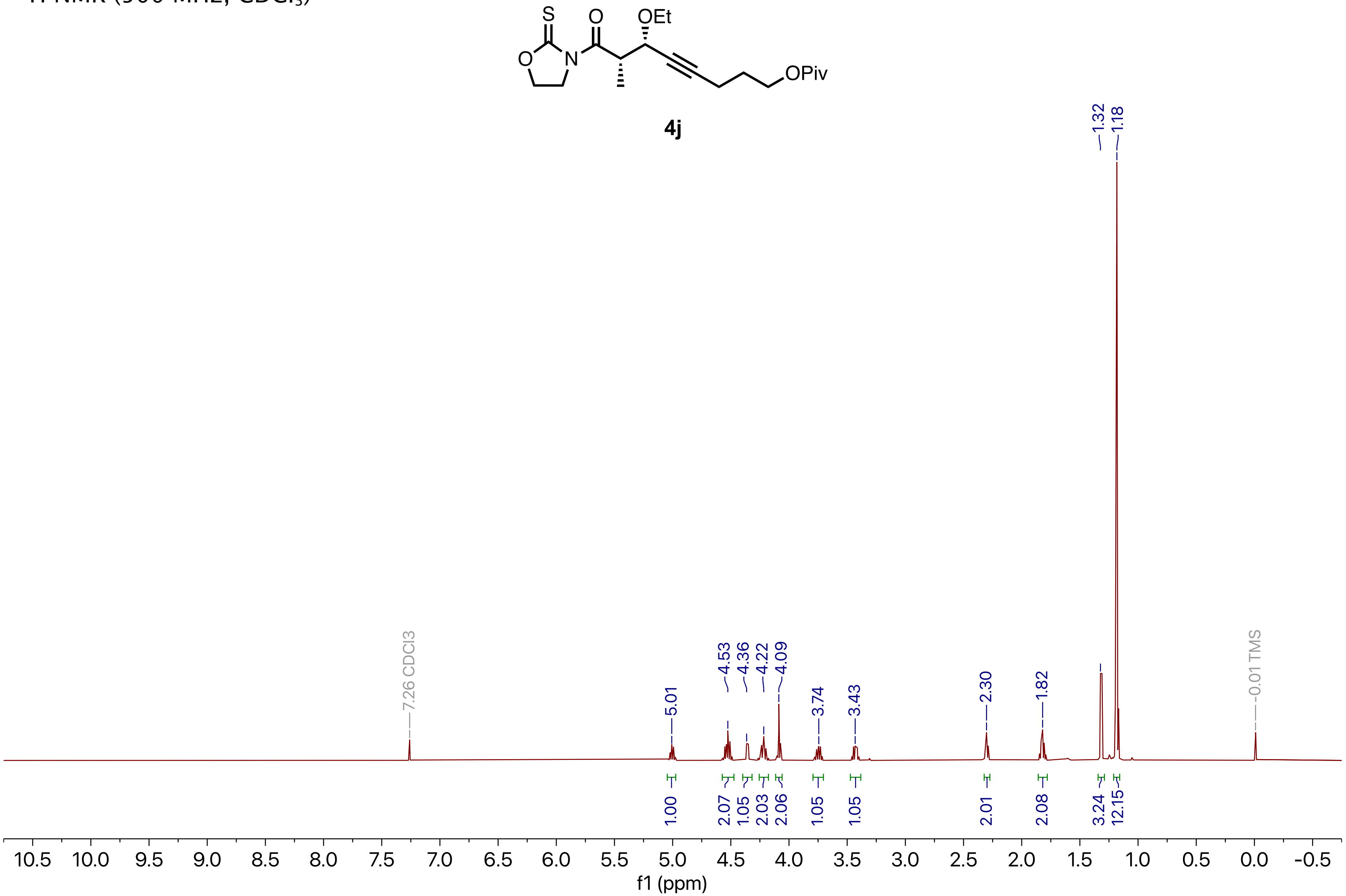

^{13}C NMR (126 MHz, $CDCl_3$)

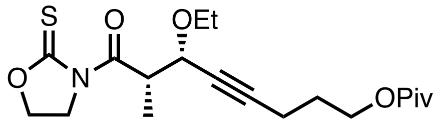

4j

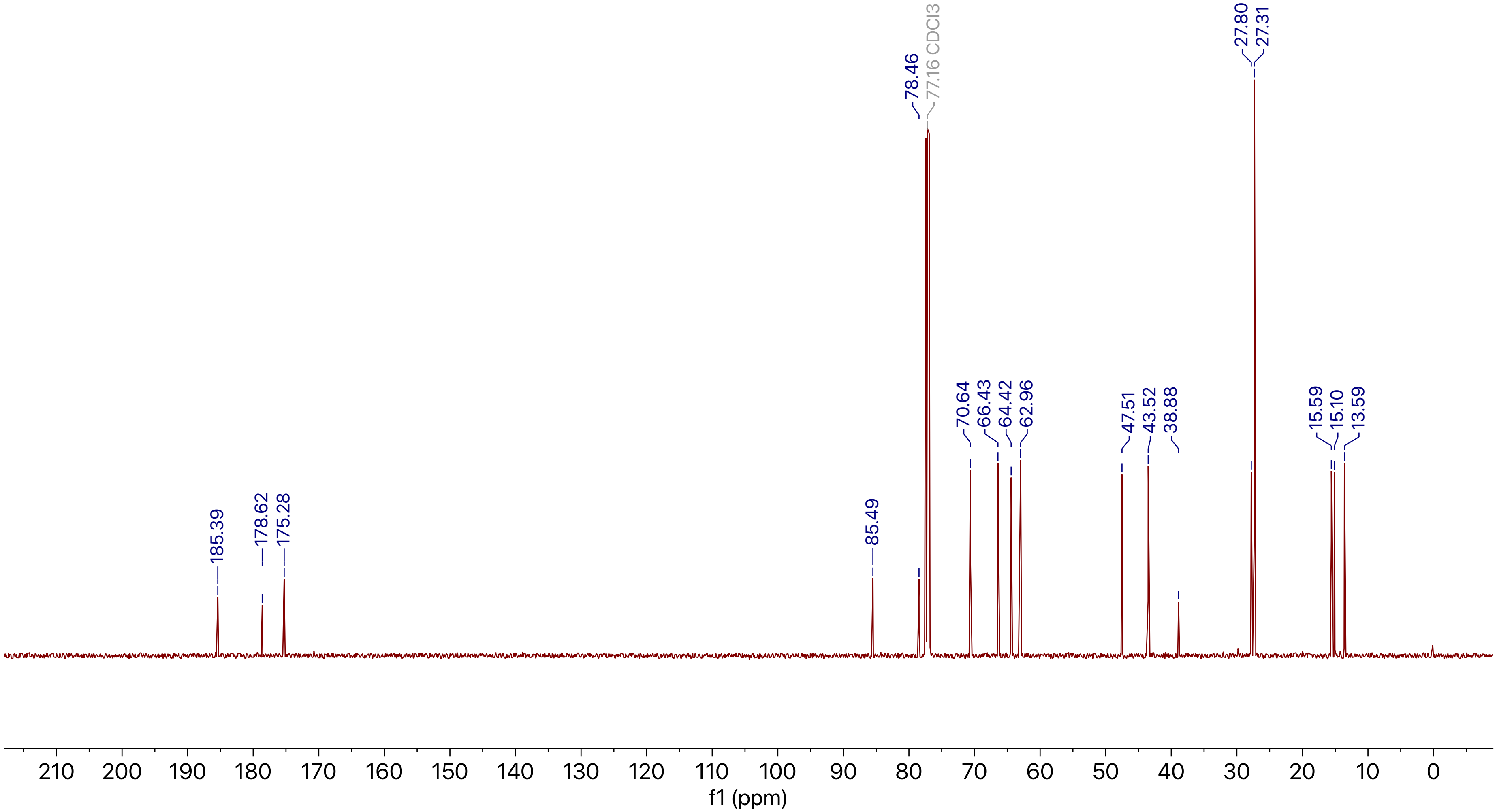

2D ^{1}H–^{1}H COSY (500 MHz, $CDCl_3$)

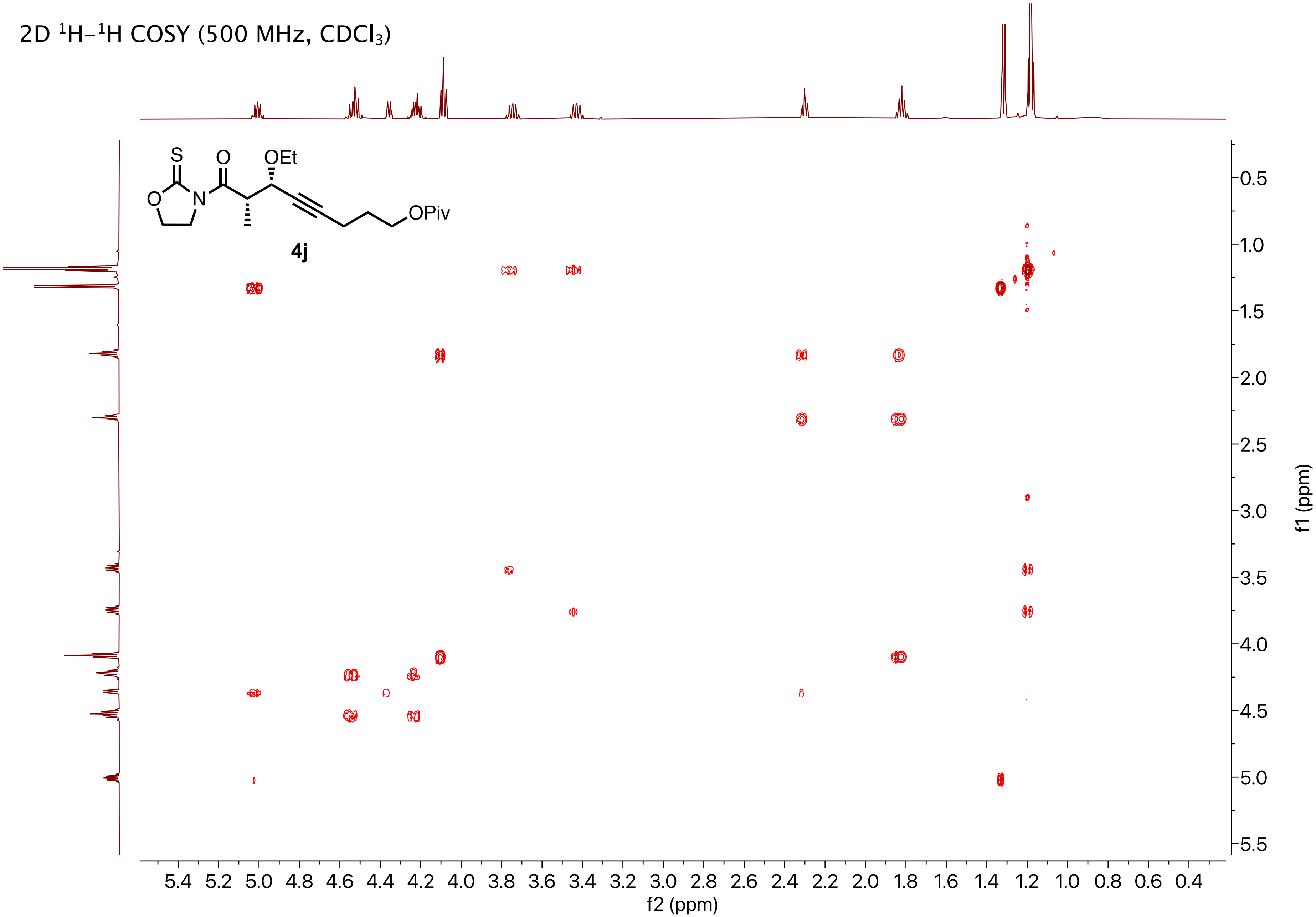

2D $^{1}H-^{13}C$ HSQC (500 MHz, $CDCl_3$)

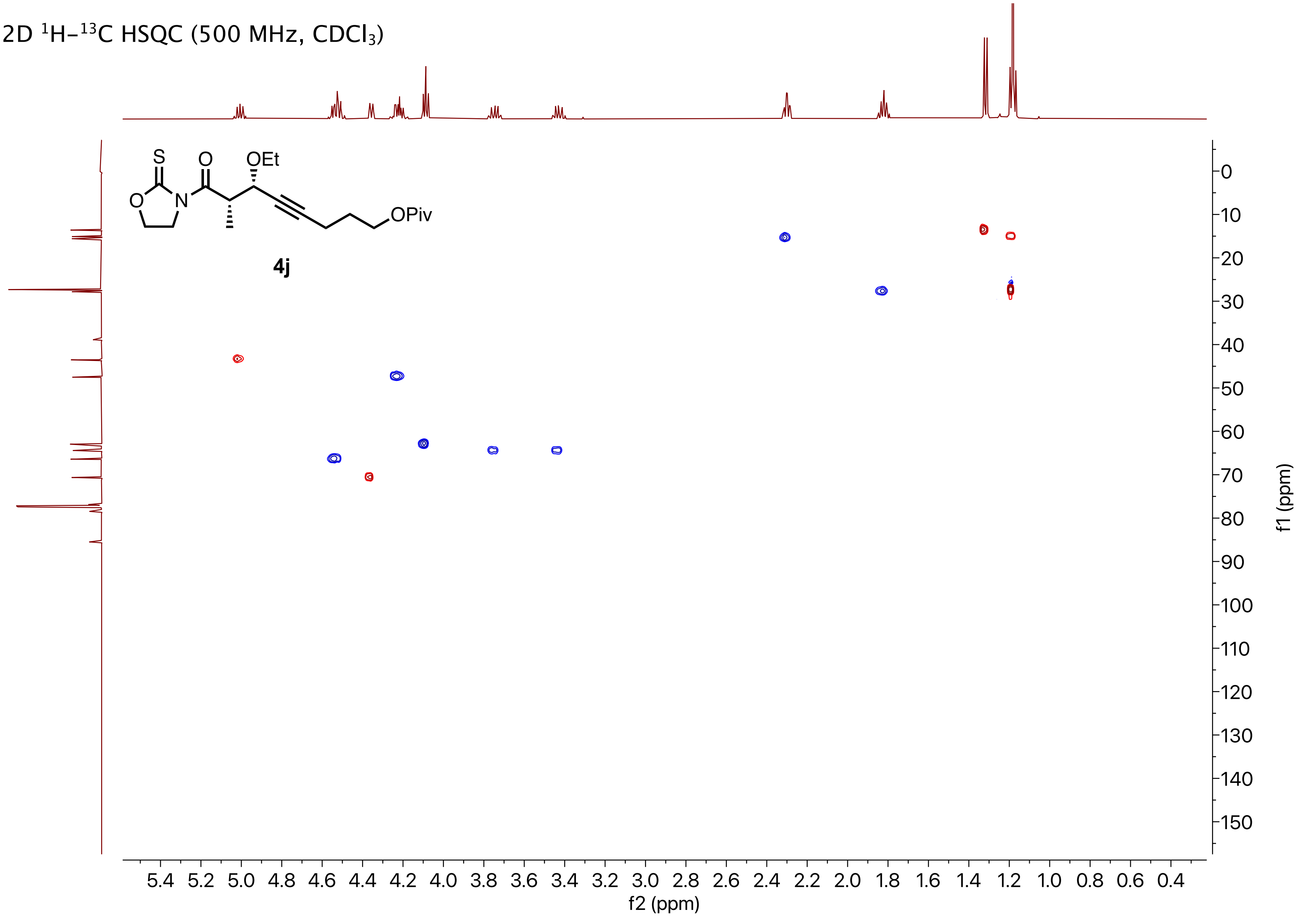

^{1}H NMR (400 MHz, $CDCl_3$)

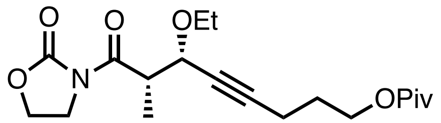

5j

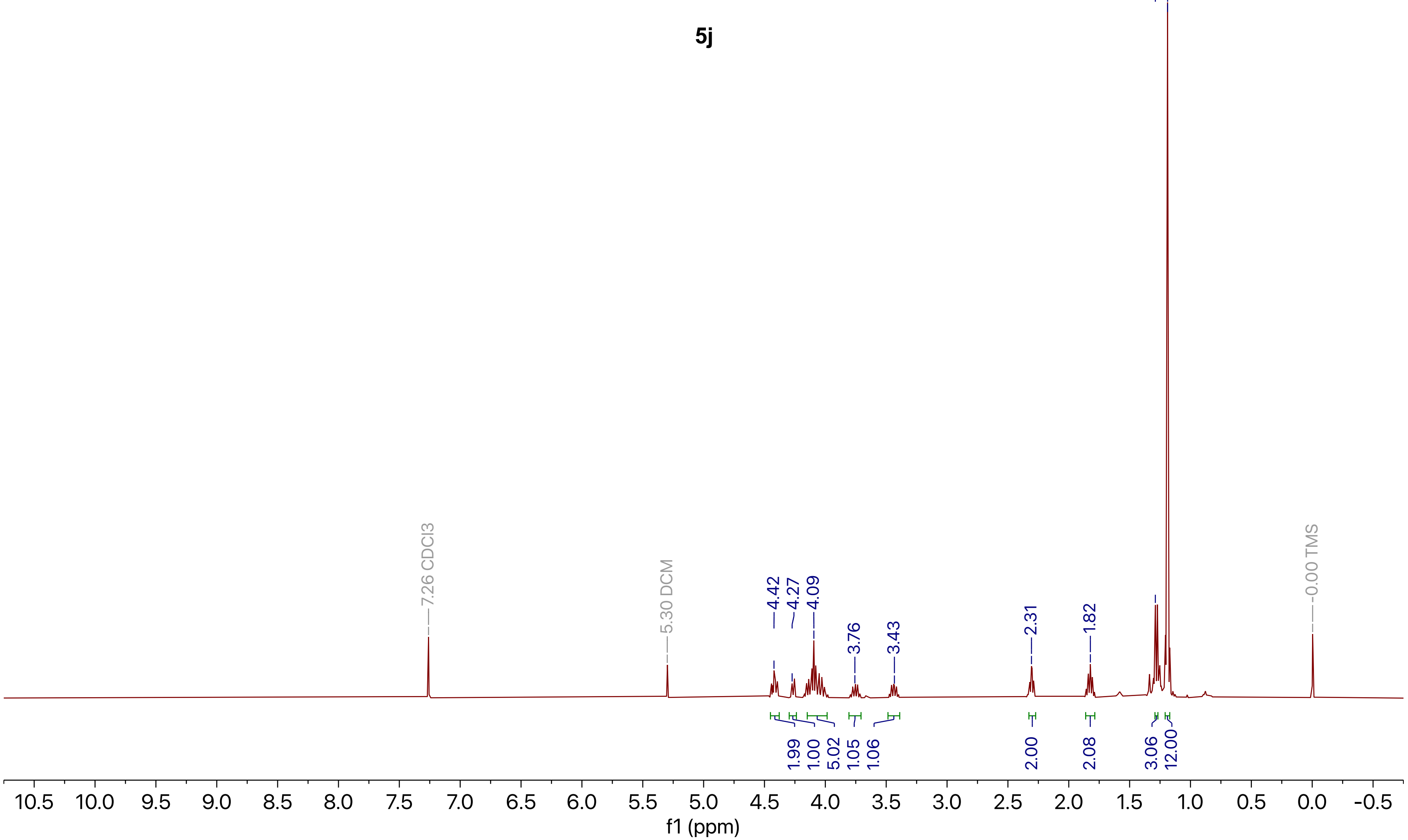

^{1}H NMR (500 MHz, $CDCl_3$)

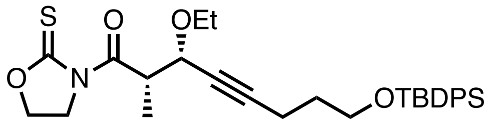

4k

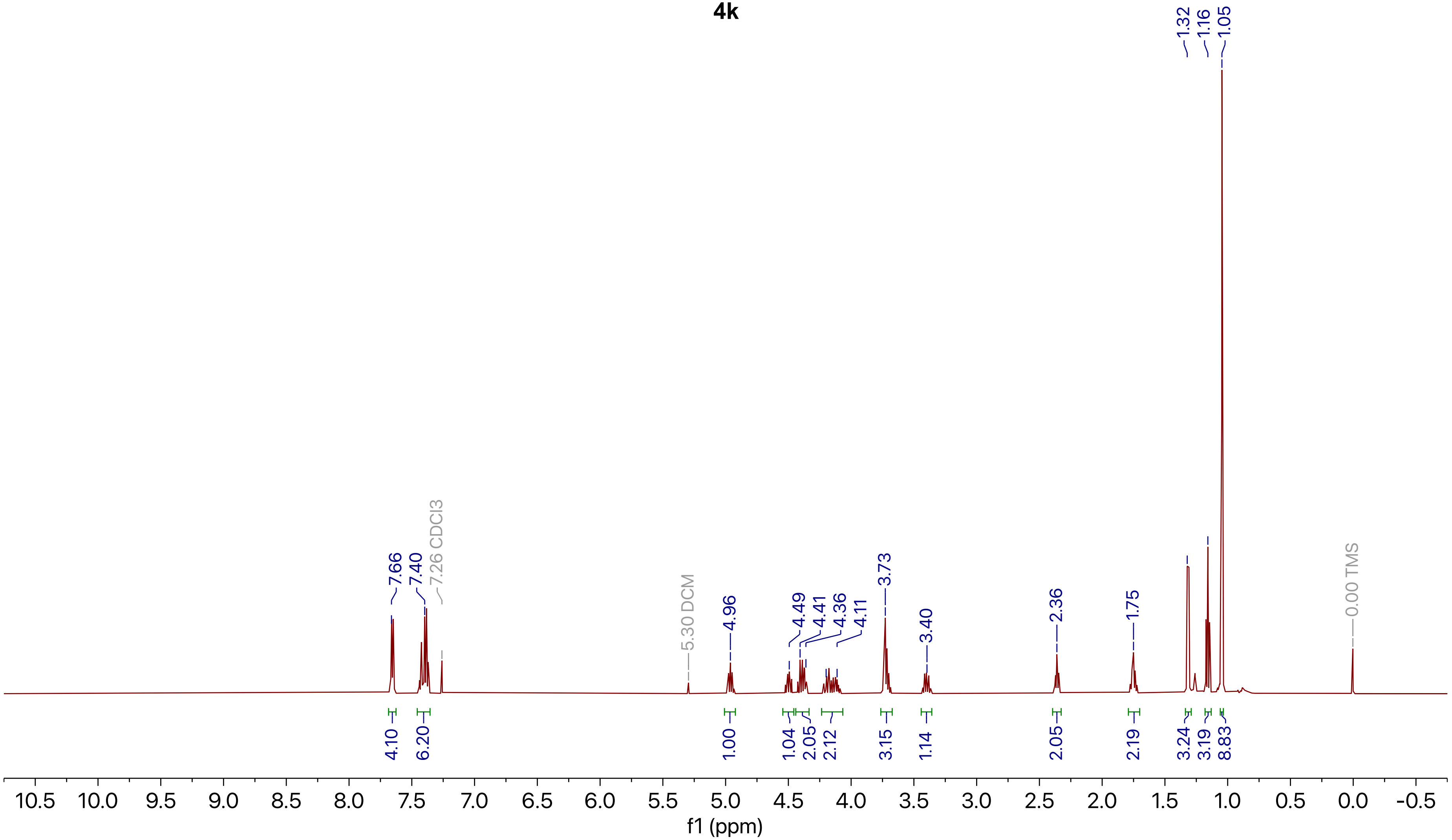

$^{13}C\{^{1}H\}$ NMR (126 MHz, $CDCl_3$)

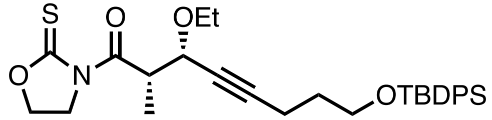

4k

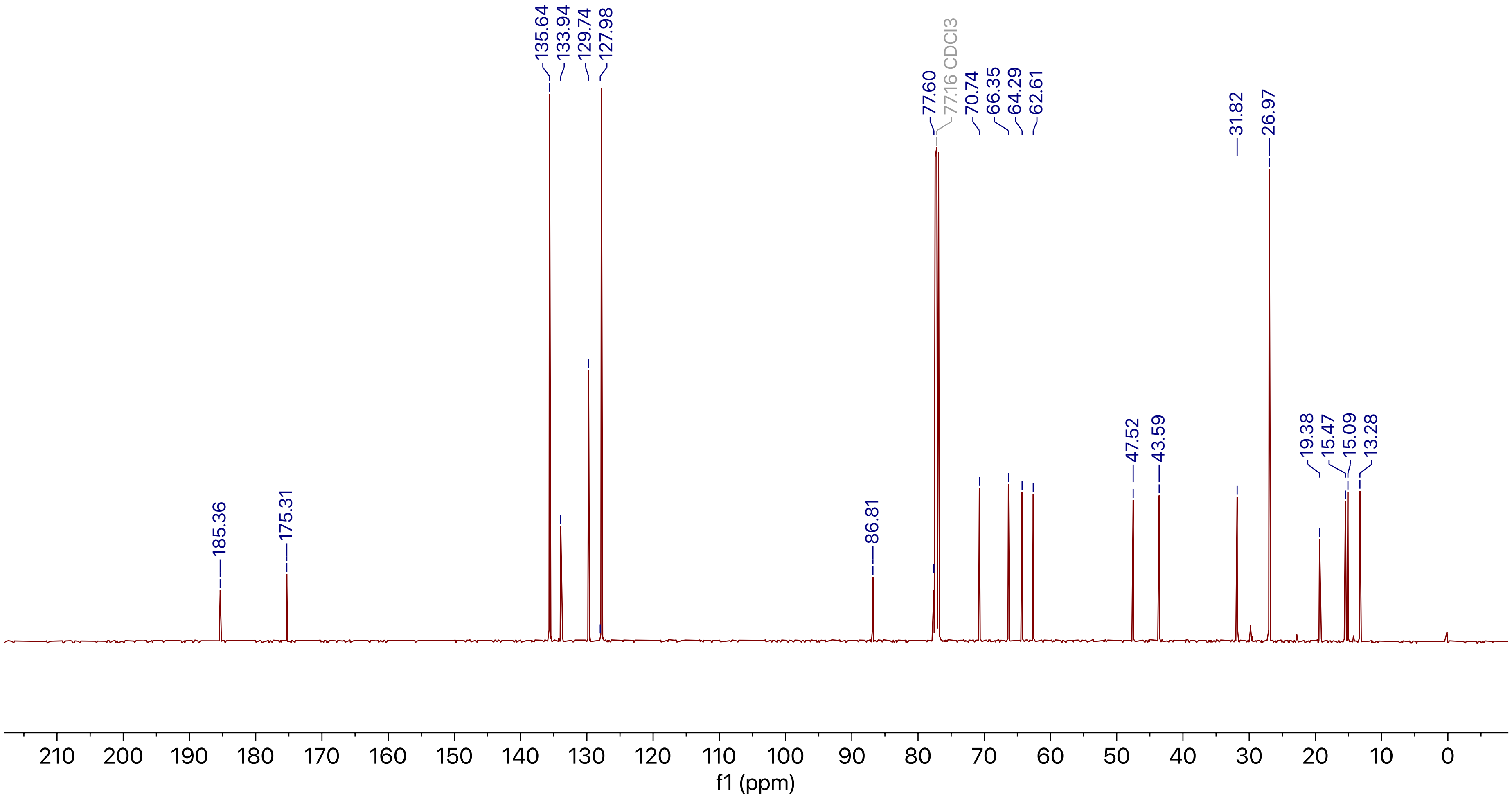

2D ^{1}H–^{1}H COSY (500 MHz, $CDCl_3$)

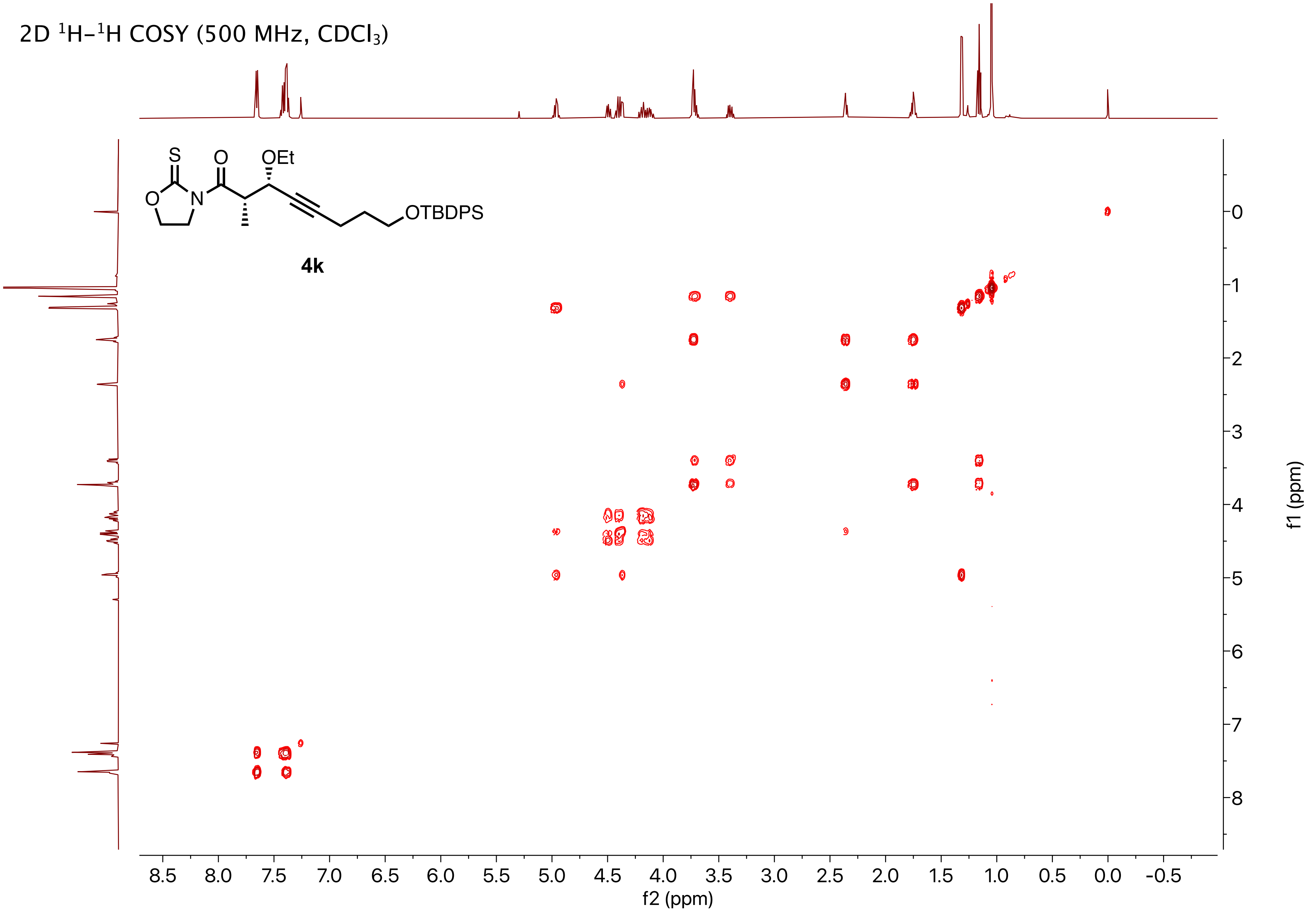

2D ^{1}H–^{13}C HSQC (500 MHz, $CDCl_3$)

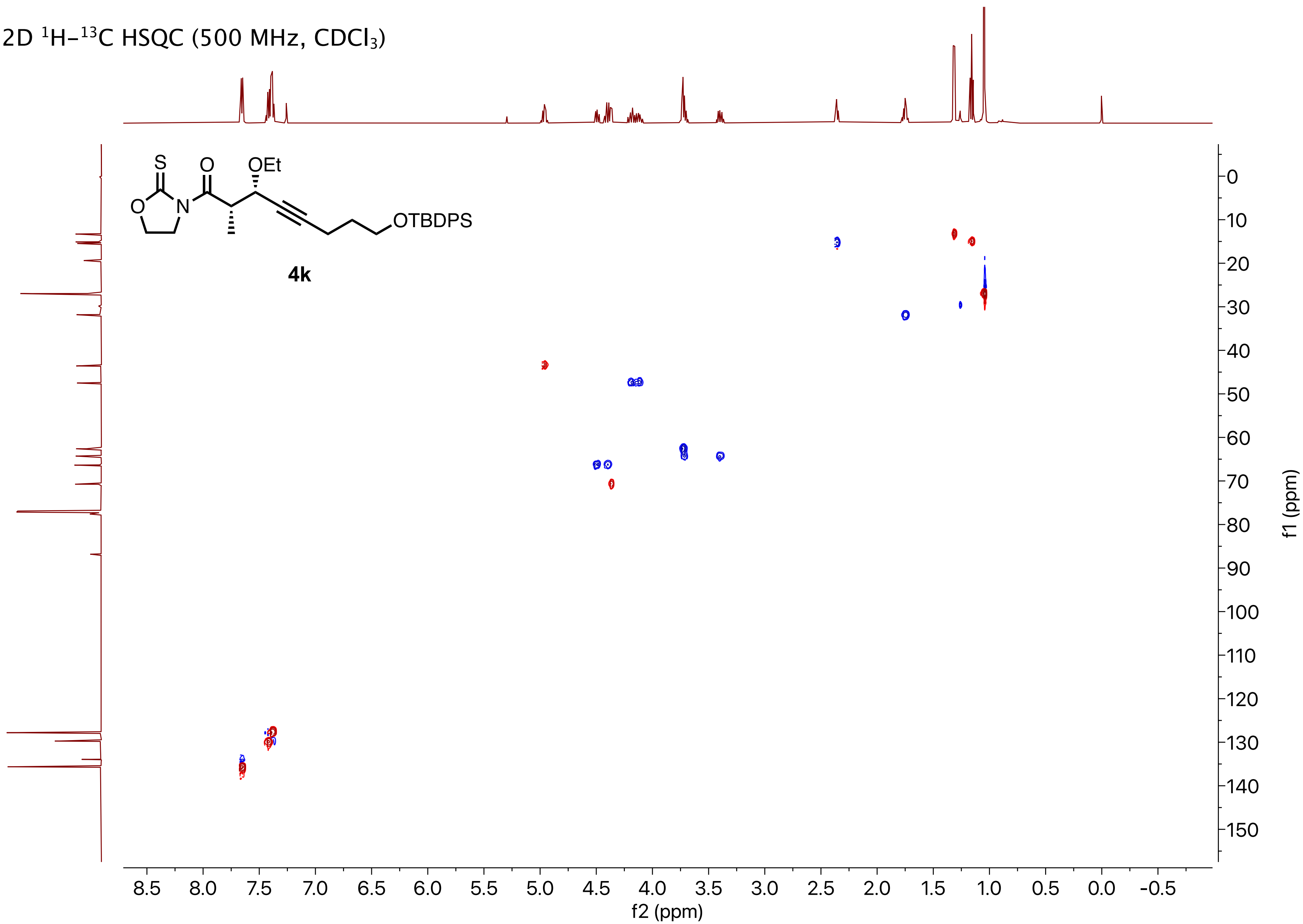

^{1}H NMR (400 MHz, $CDCl_3$)

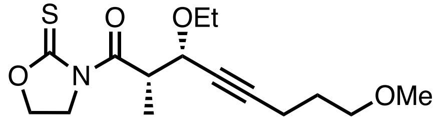

4l

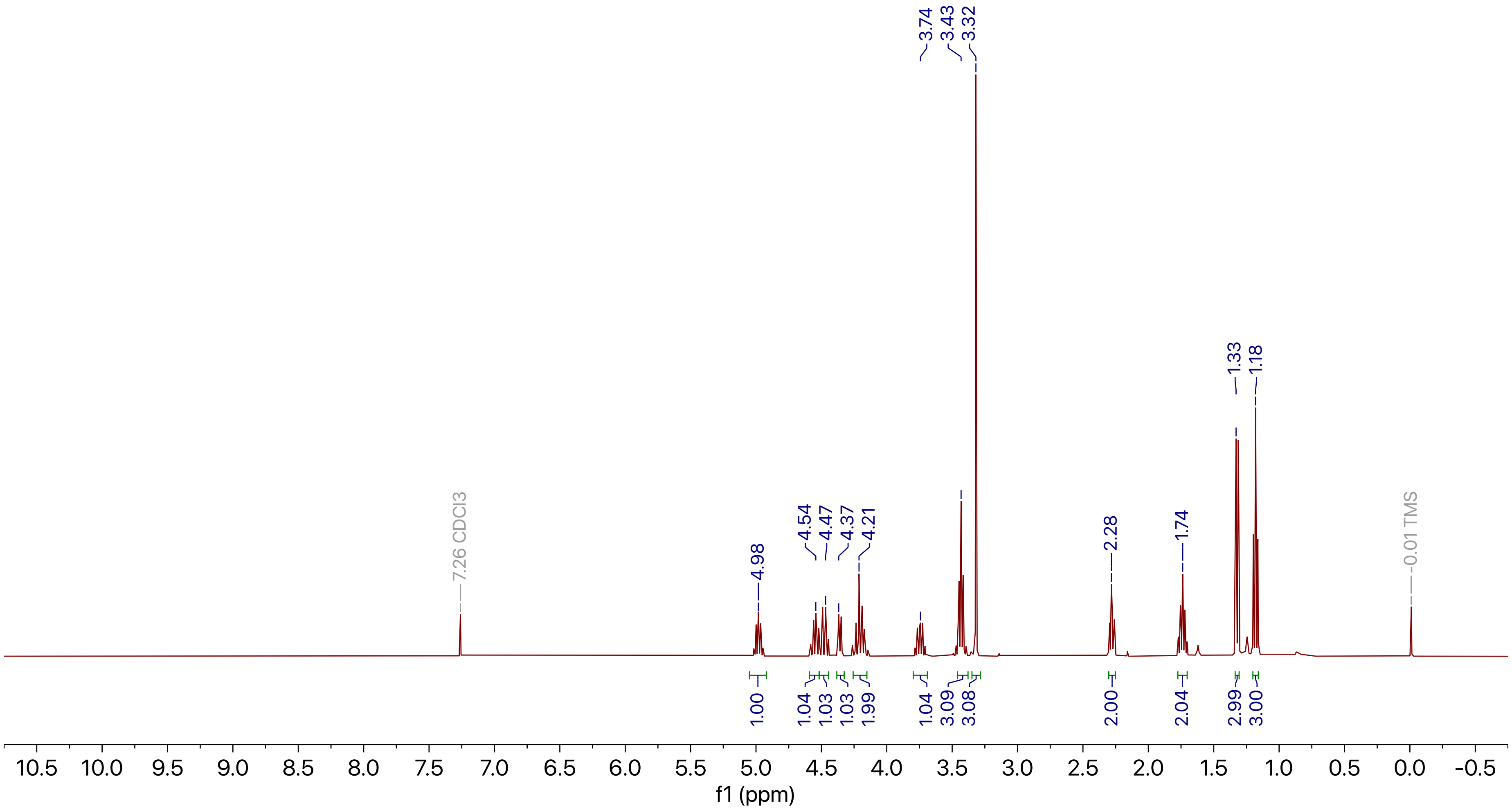

$^{13}C\{^{1}H\}$ NMR (101 MHz, $CDCl_3$)

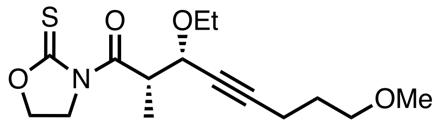

4l

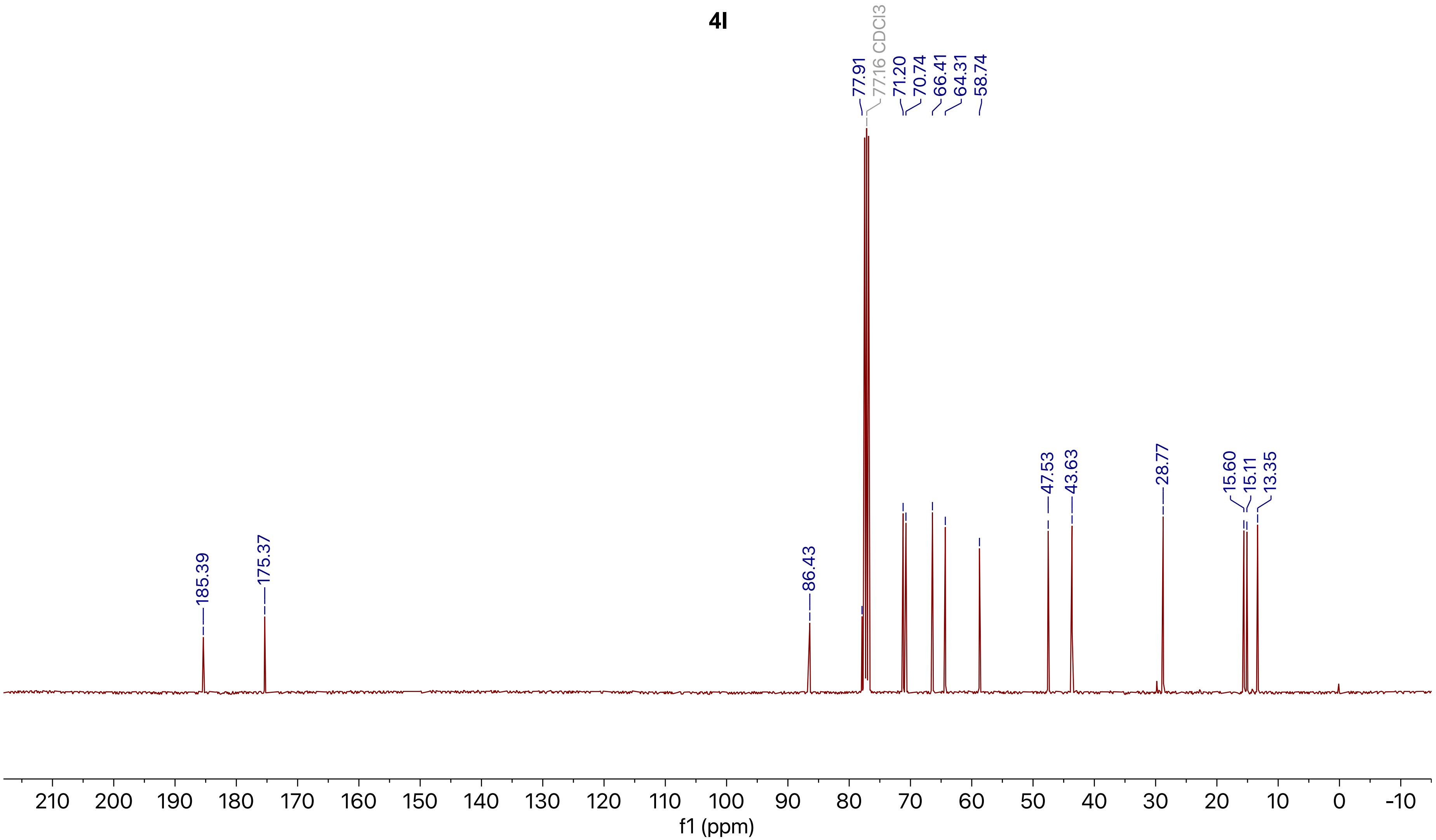

2D ^{1}H–^{1}H COSY (400 MHz, $CDCl_3$)

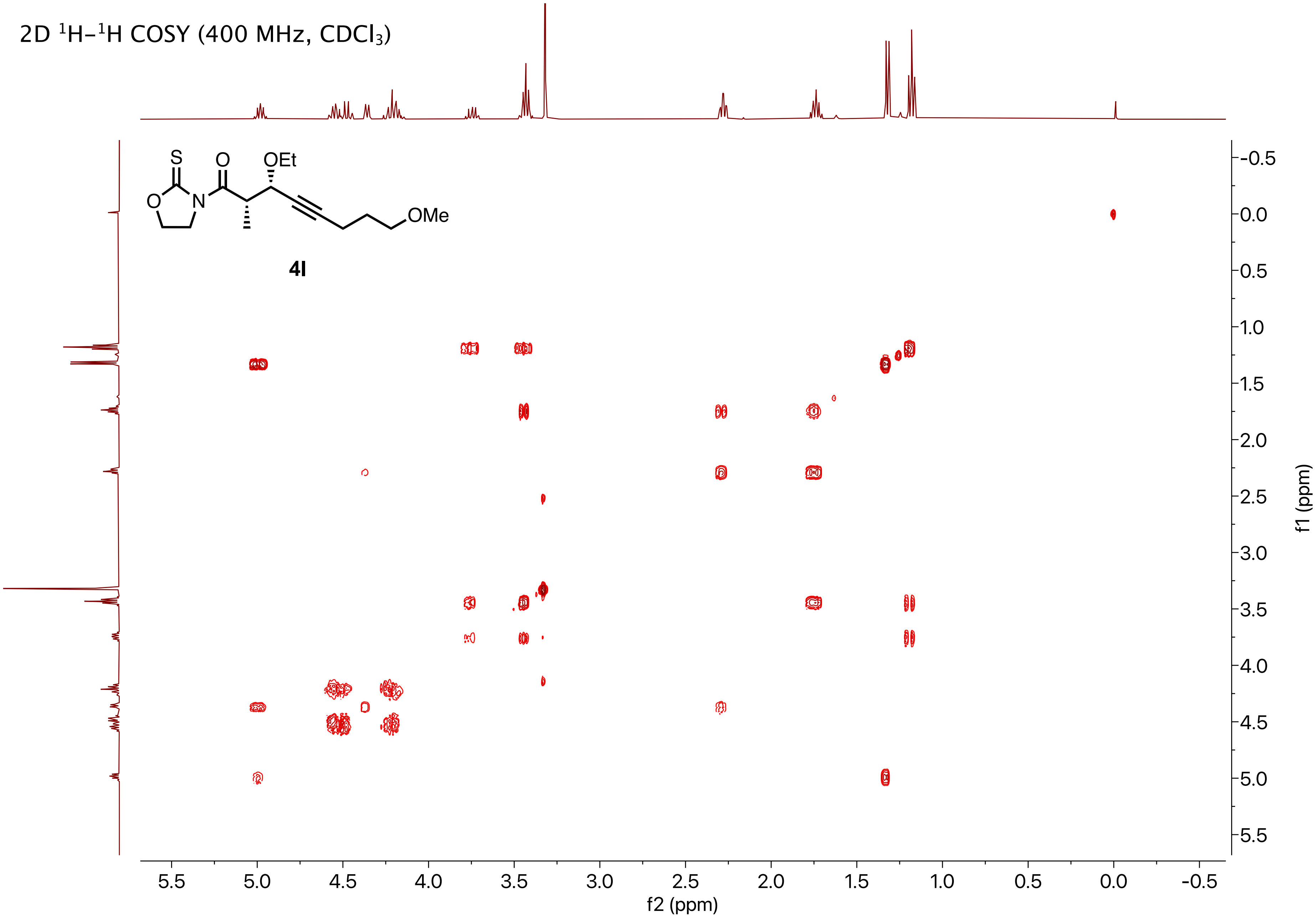

2D ^{1}H–^{13}C HSQC (400 MHz, $CDCl_3$)

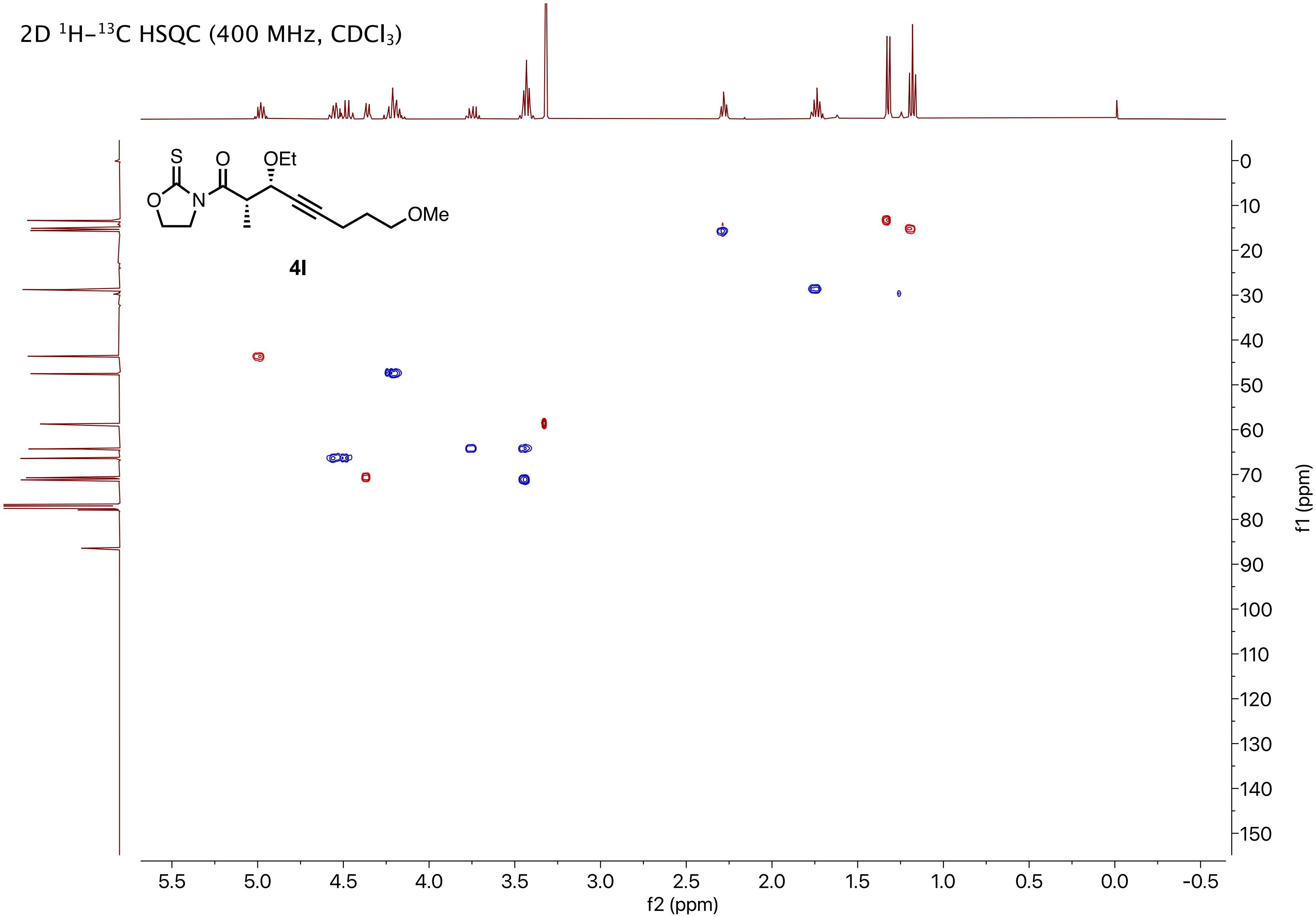

^{1}H NMR (400 MHz, $CDCl_3$)

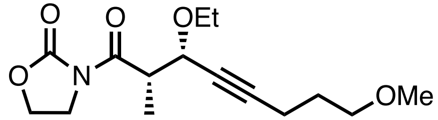

5l

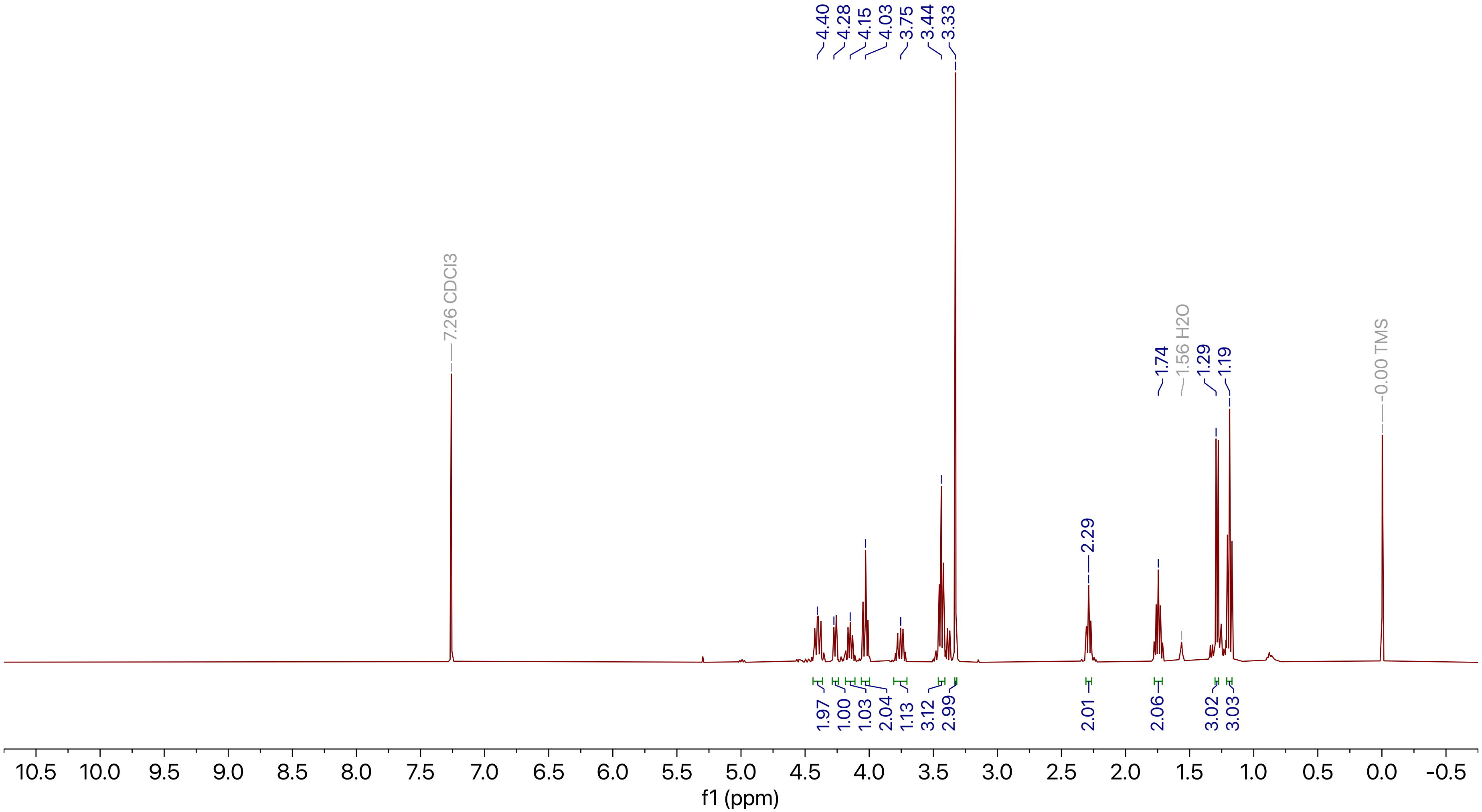

^{1}H NMR (400 MHz, $CDCl_3$)

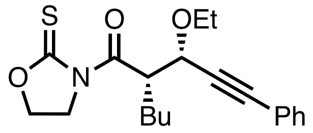

4m

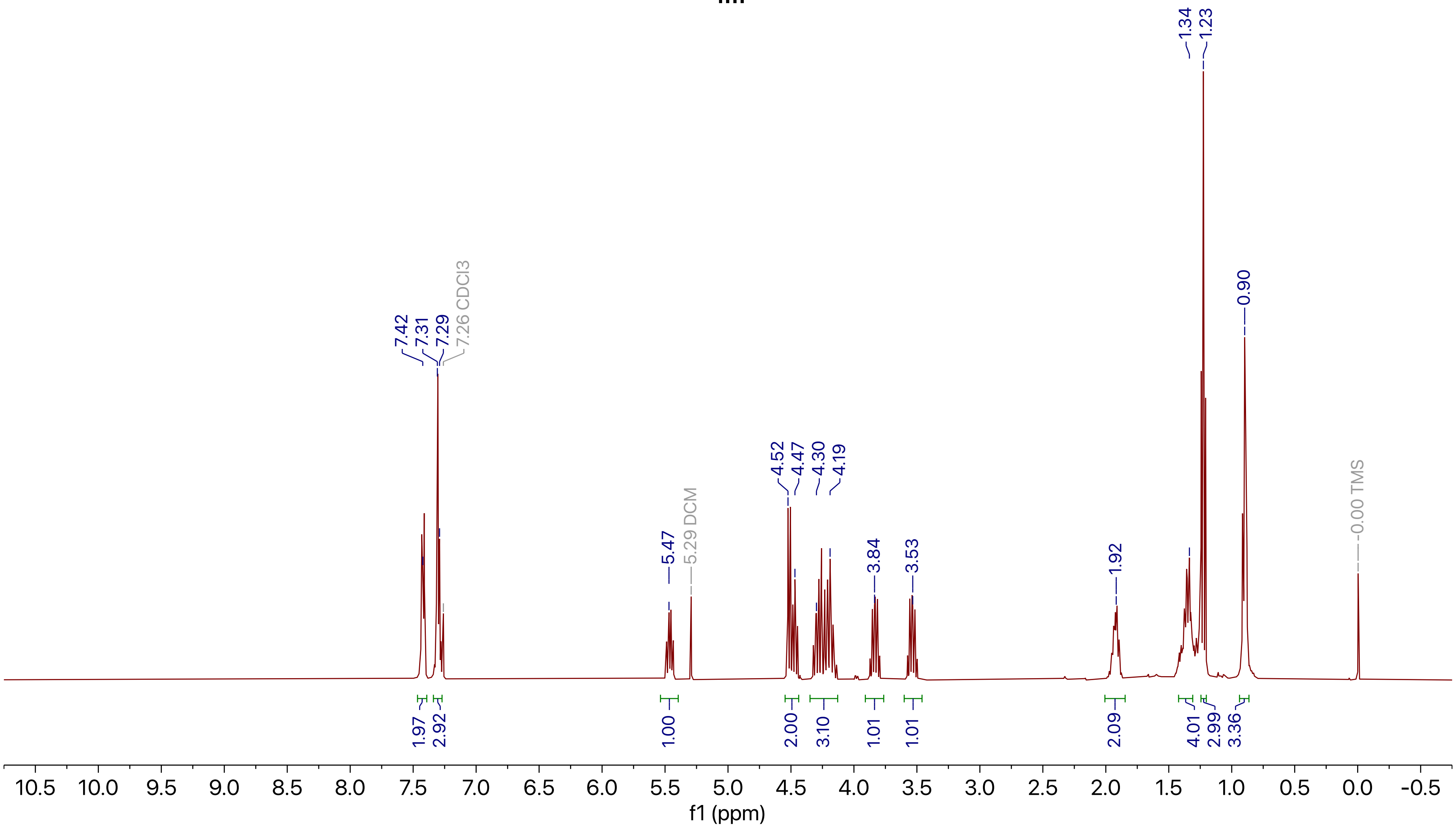

$^{13}C\{^{1}H\}$ NMR (101 MHz, $CDCl_3$)

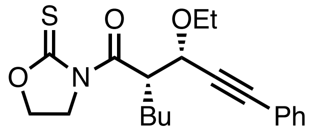

4m

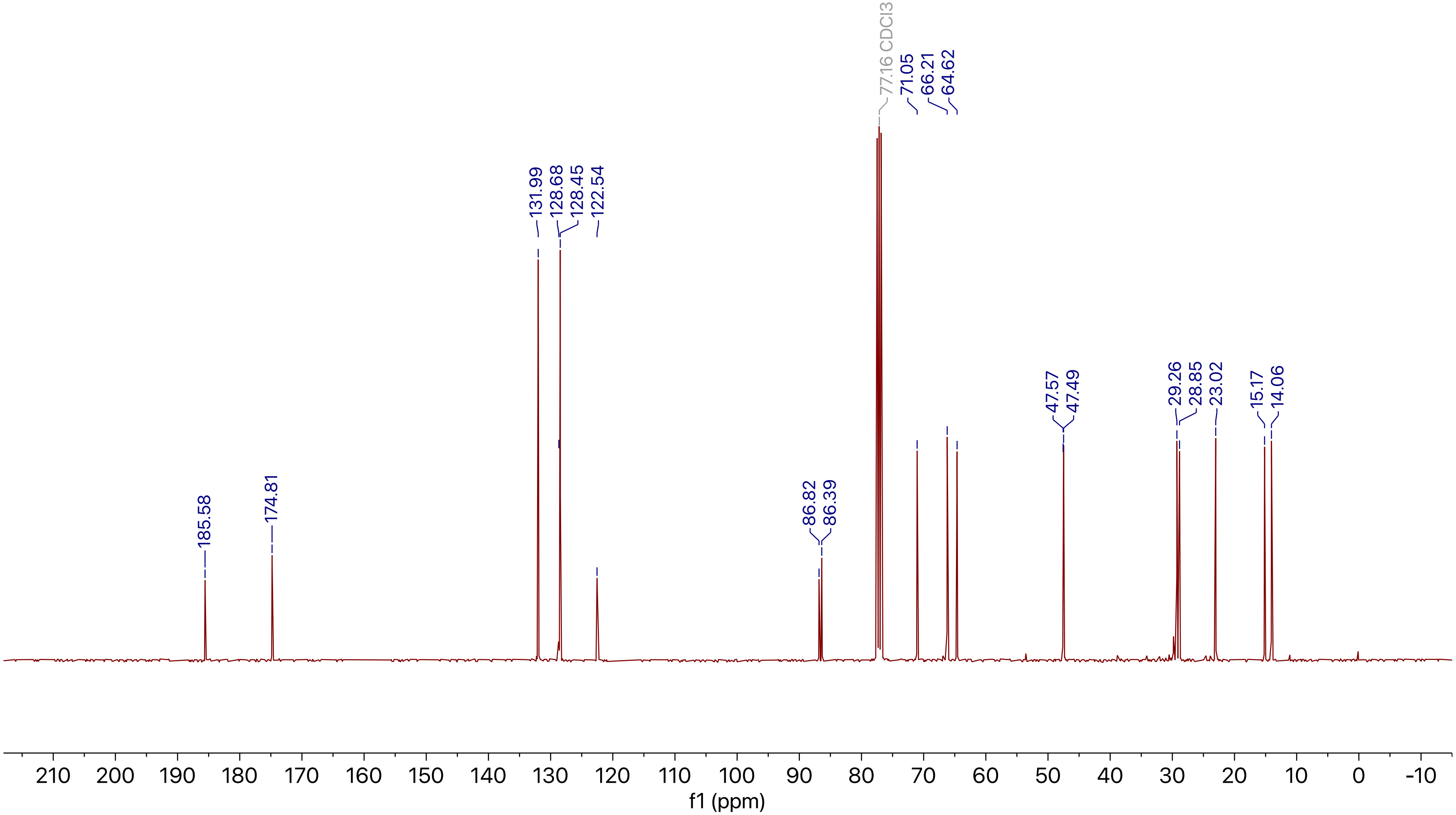

2D ^1H–^1H COSY (400 MHz, $CDCl_3$)

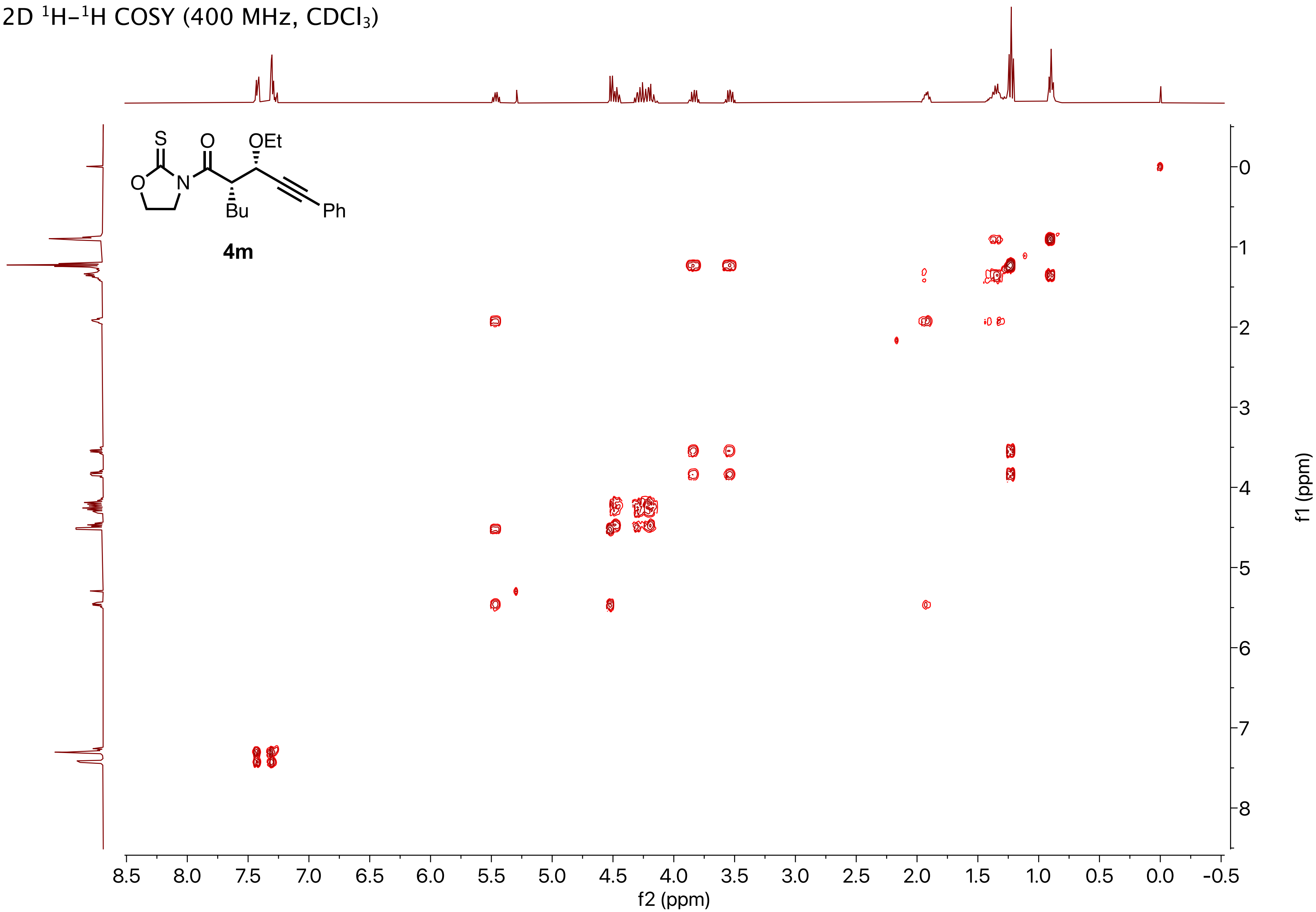

2D ^{1}H–^{13}C HSQC (400 MHz, $CDCl_3$)

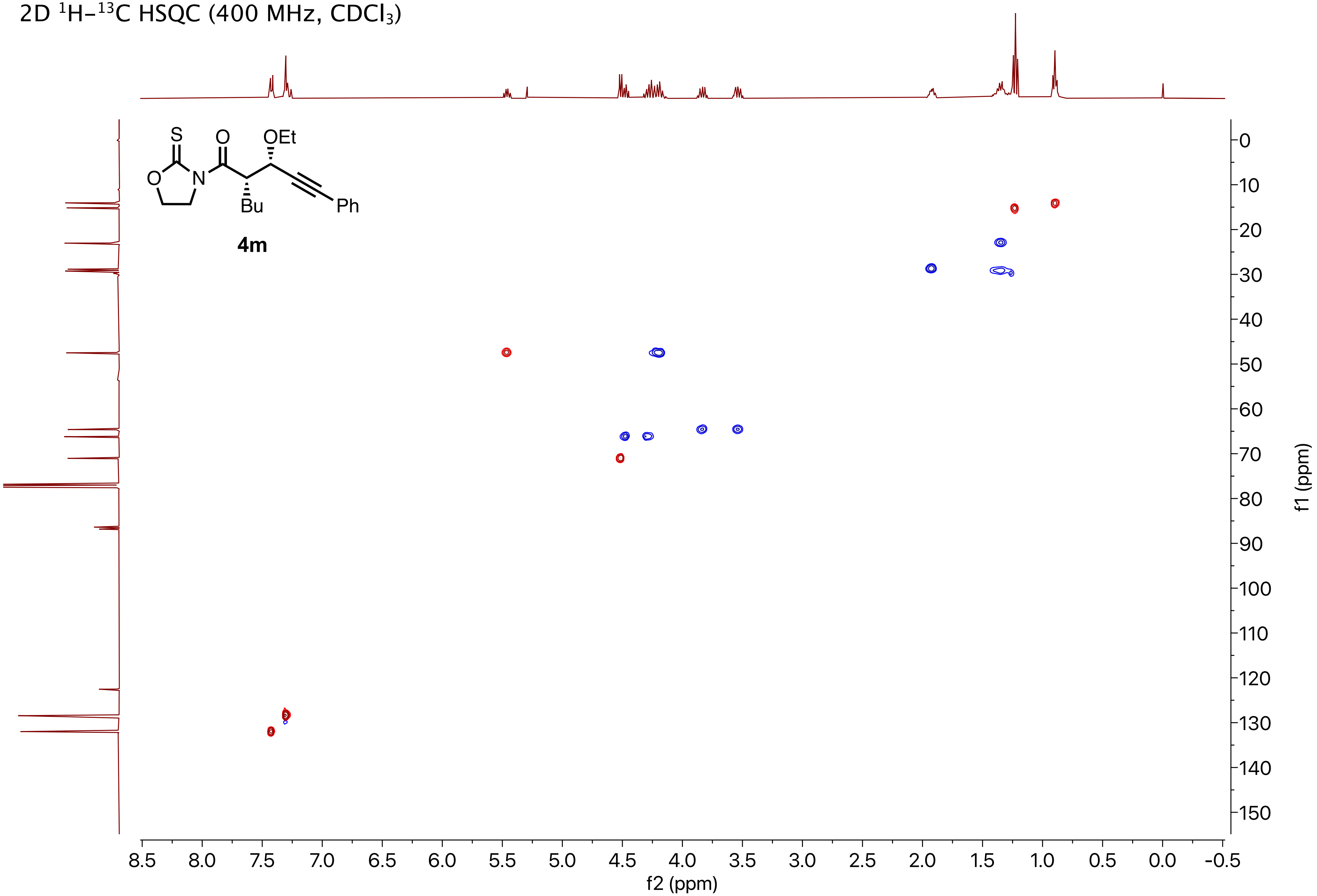

^{1}H NMR (400 MHz, $CDCl_3$)

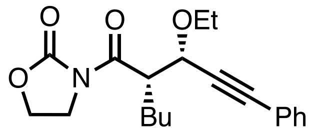

5m

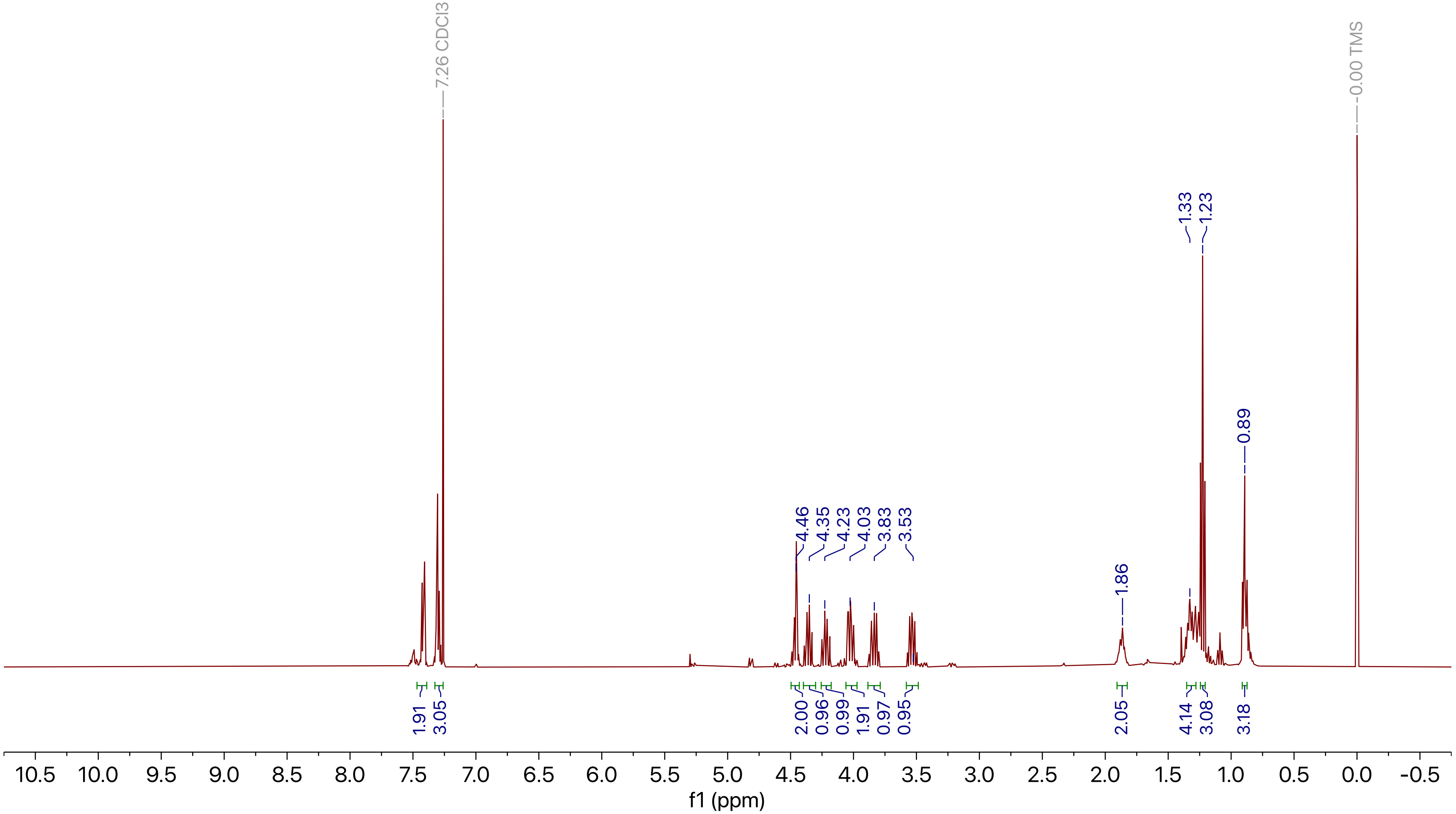

^{1}H NMR (400 MHz, $CDCl_3$)

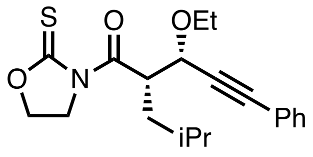

4n

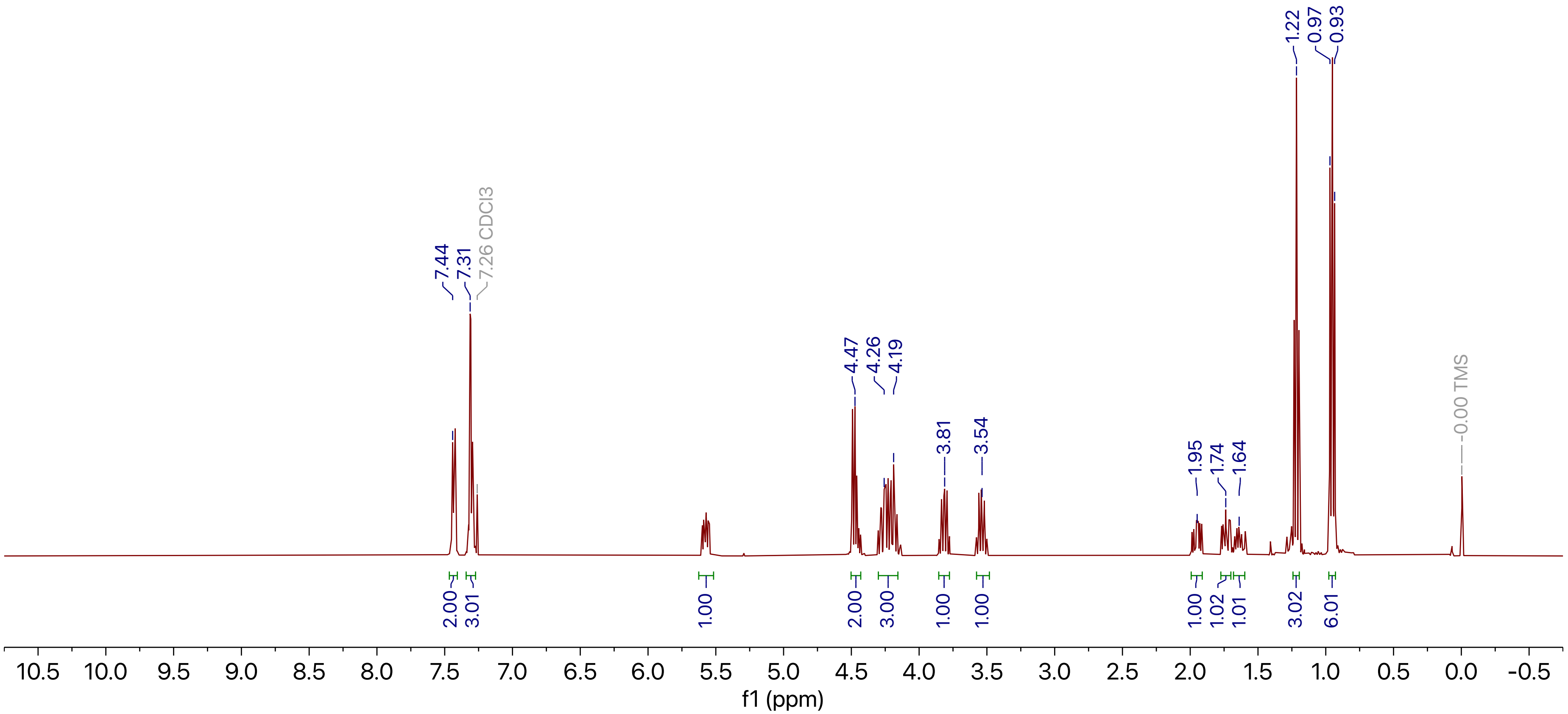

$^{13}C\{^1H\}$ NMR (101 MHz, $CDCl_3$)

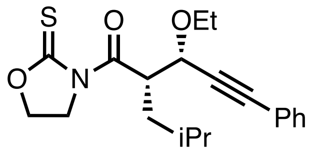

4n

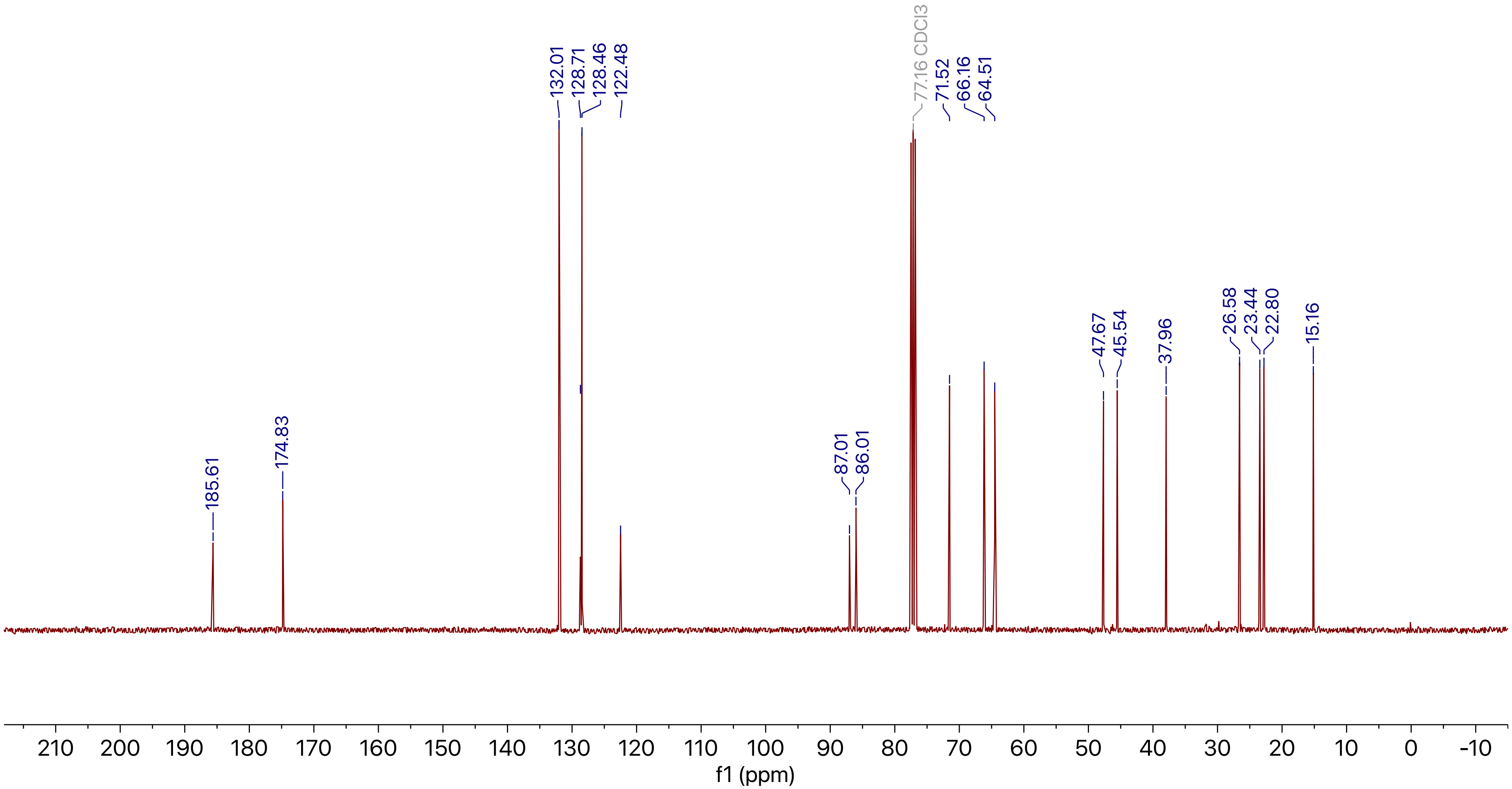

2D ^1H–^1H COSY (400 MHz, $CDCl_3$)

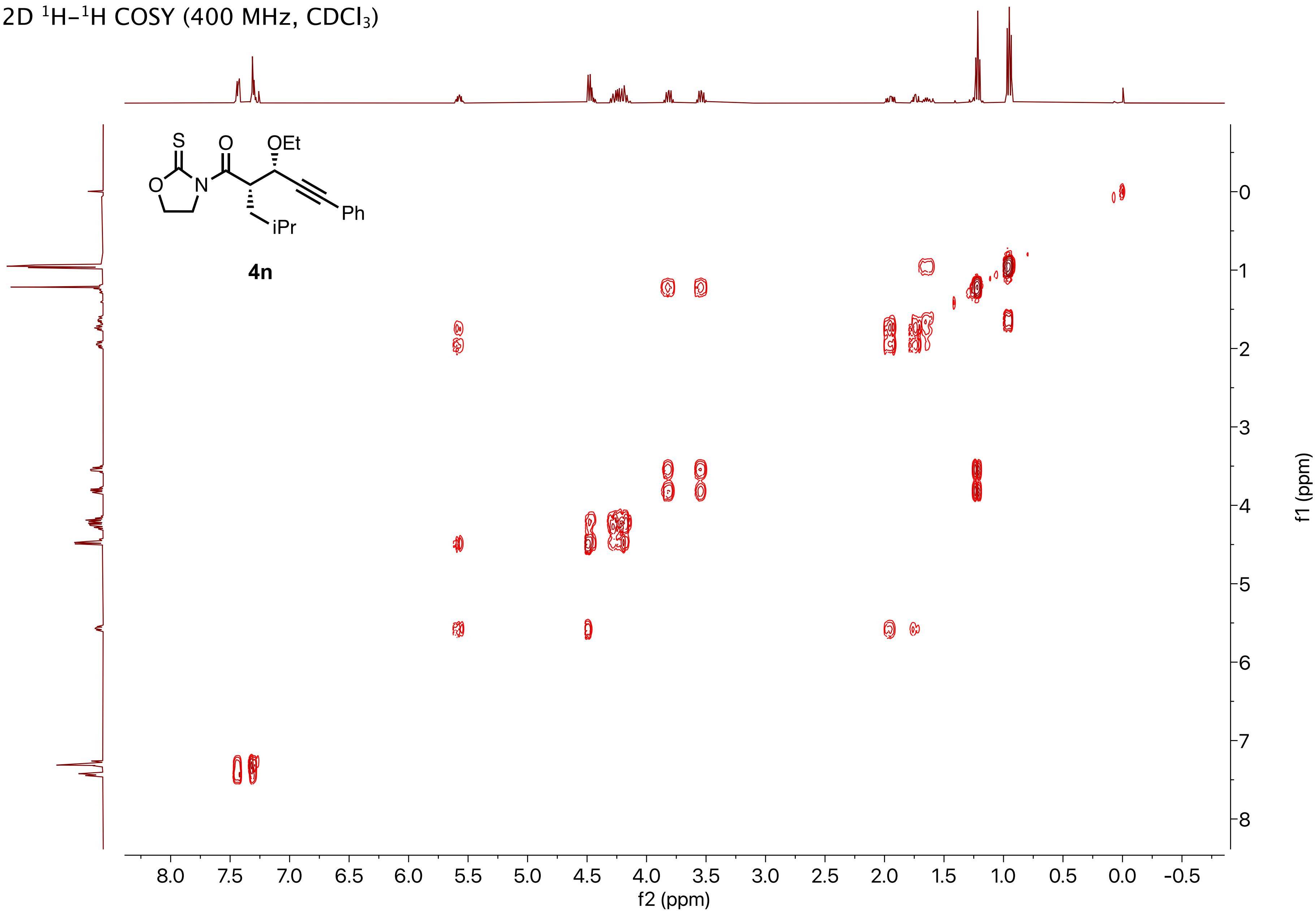

2D ^{1}H–^{13}C HSQC (400 MHz, $CDCl_3$)

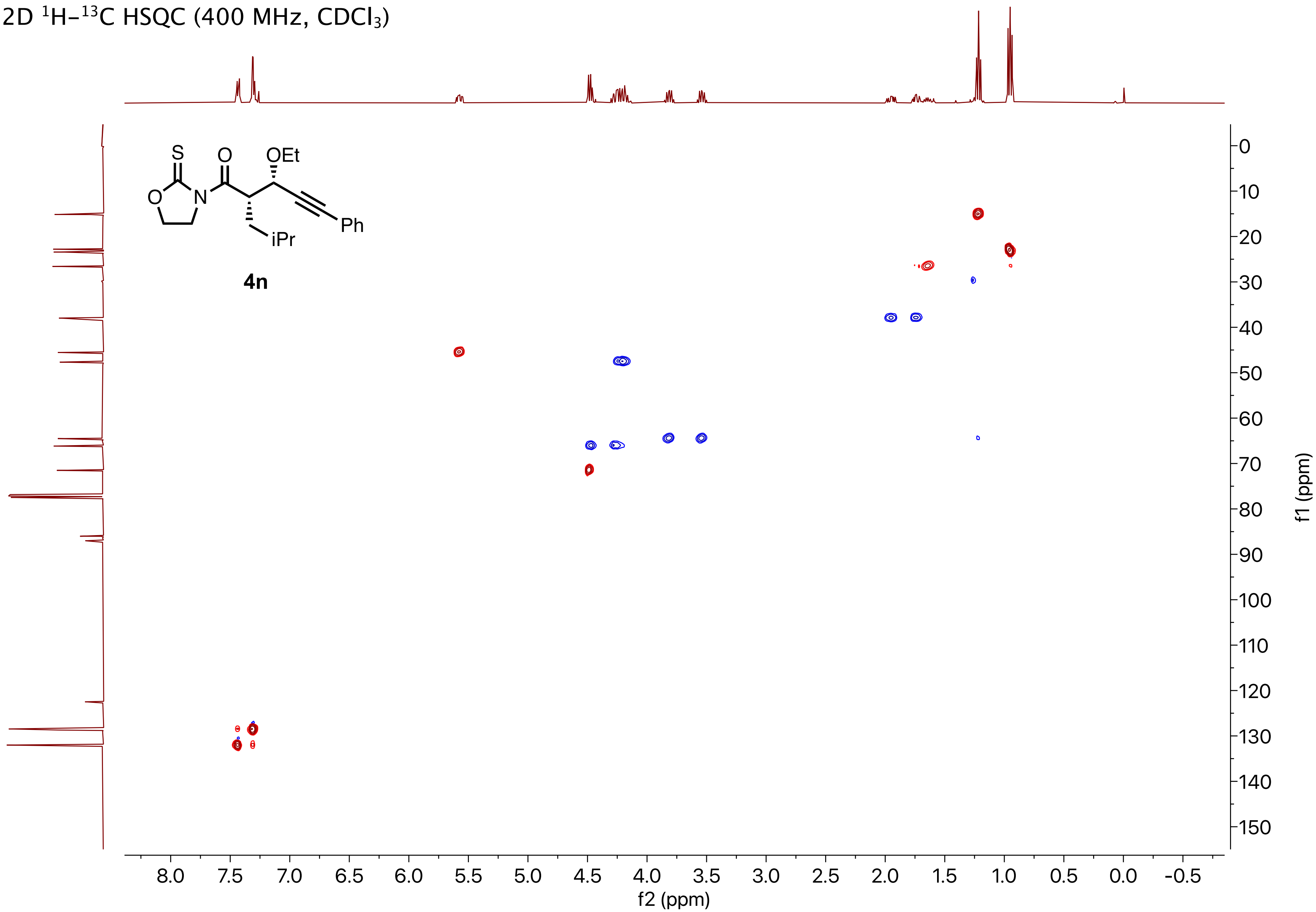

^1H NMR (400 MHz, $CDCl_3$)

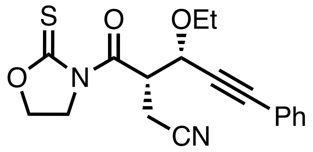

4o

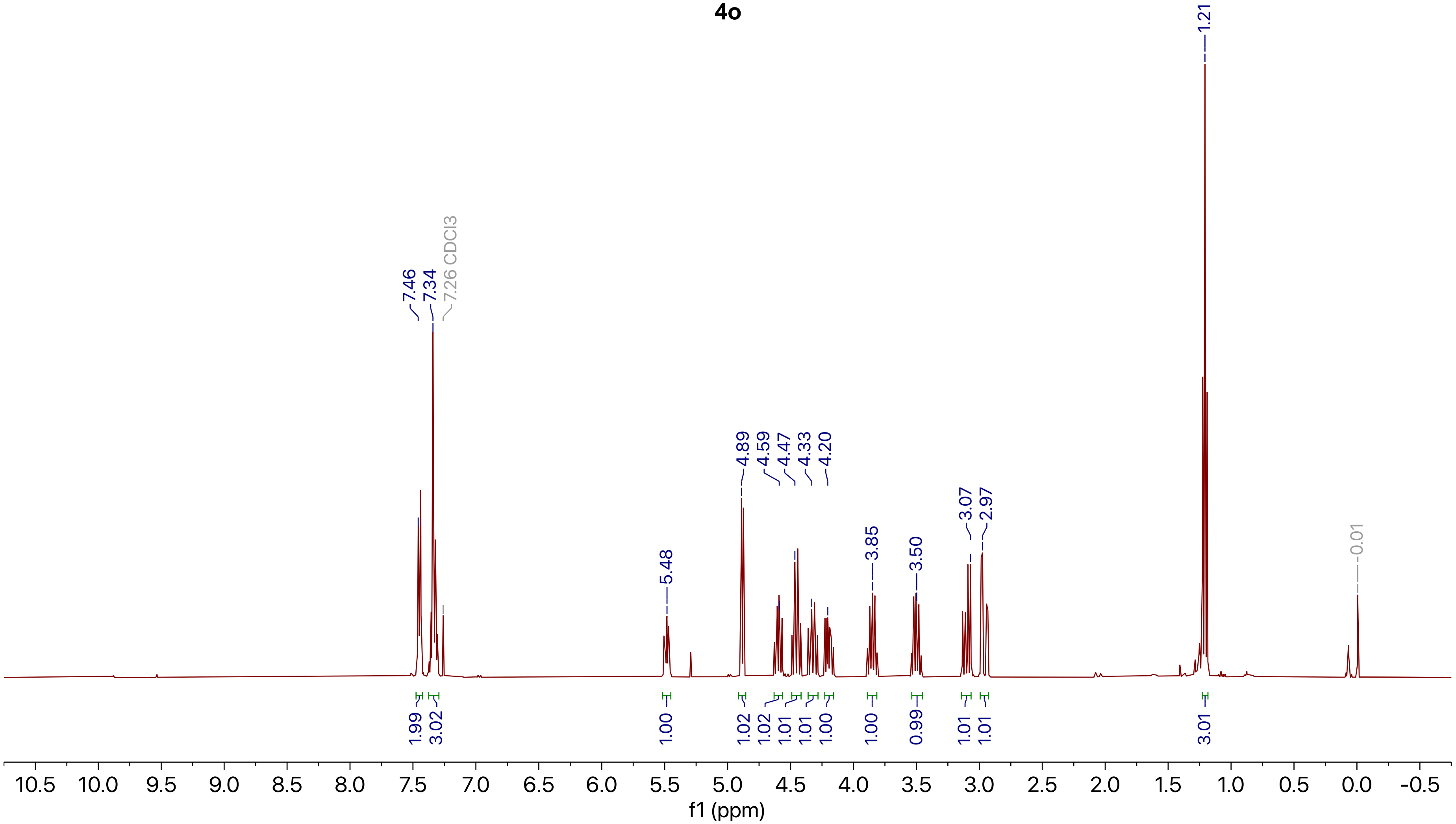

$^{13}C\{^{1}H\}$ NMR (101 MHz, $CDCl_3$)

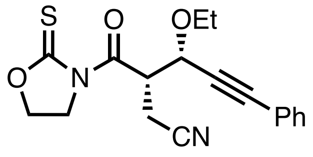

4o

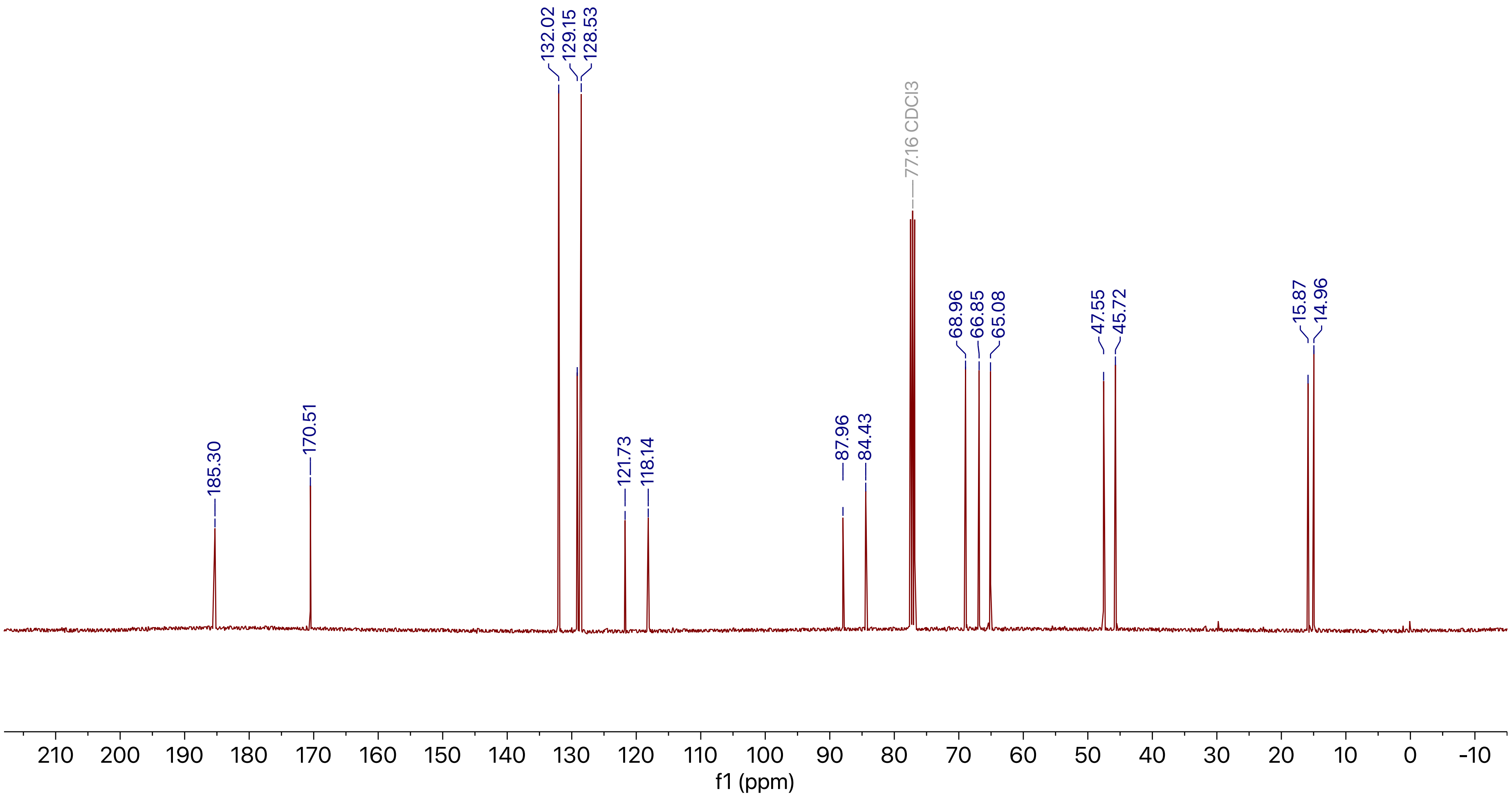

2D ^{1}H–^{1}H COSY (400 MHz, $CDCl_3$)

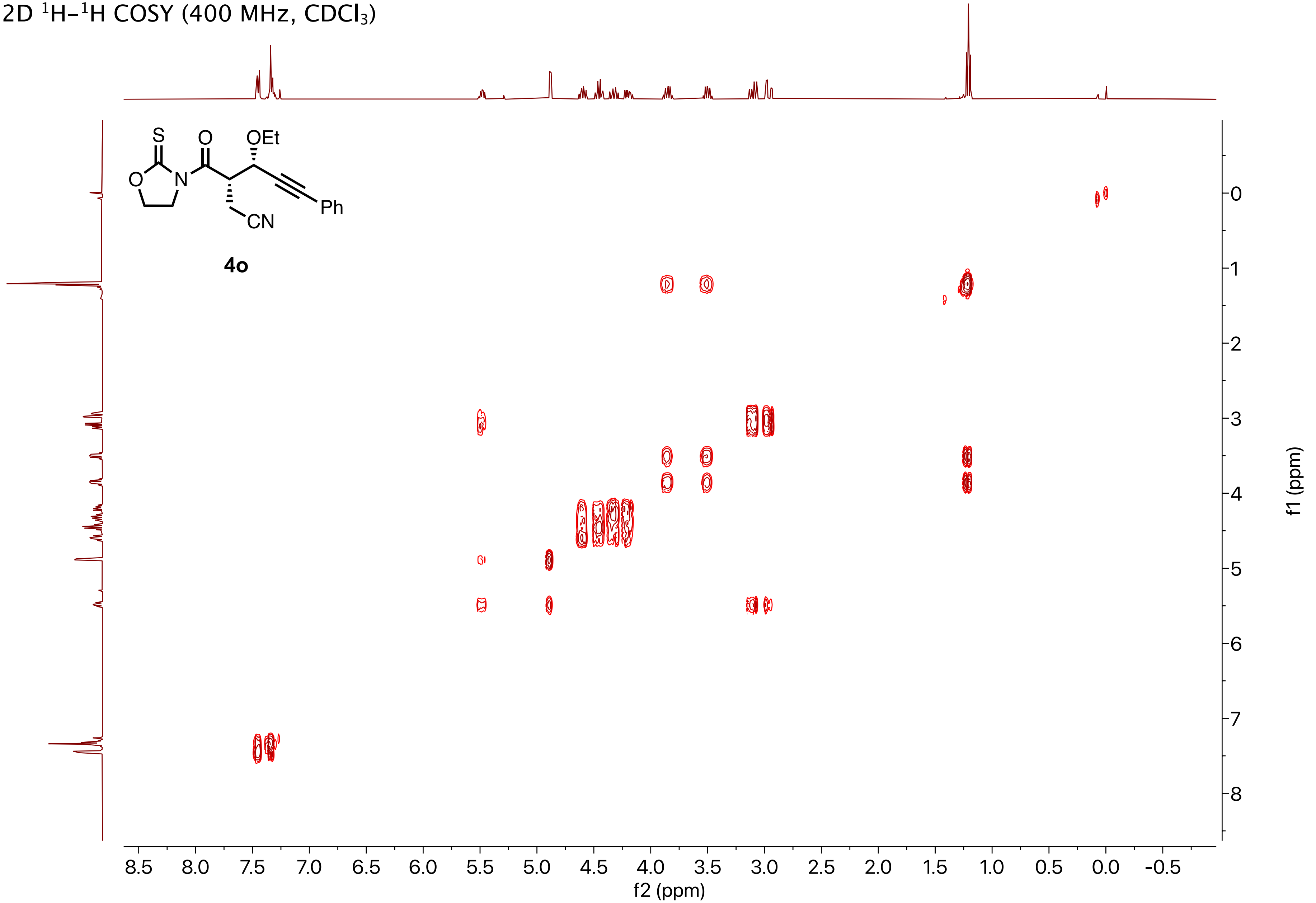

2D ^{1}H–^{13}C HSQC (400 MHz, $CDCl_3$)

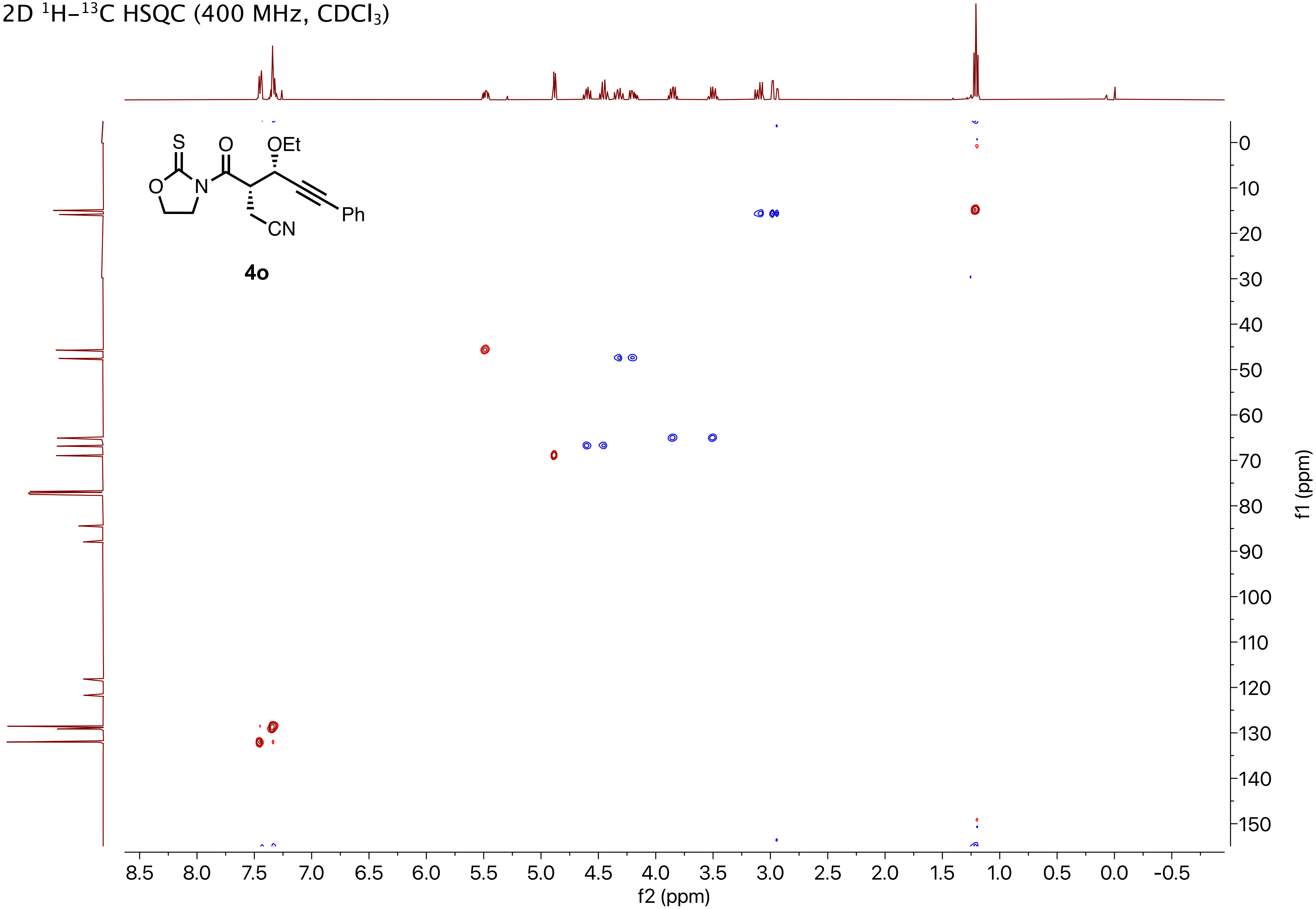

^{1}H NMR (400 MHz, $CDCl_3$)

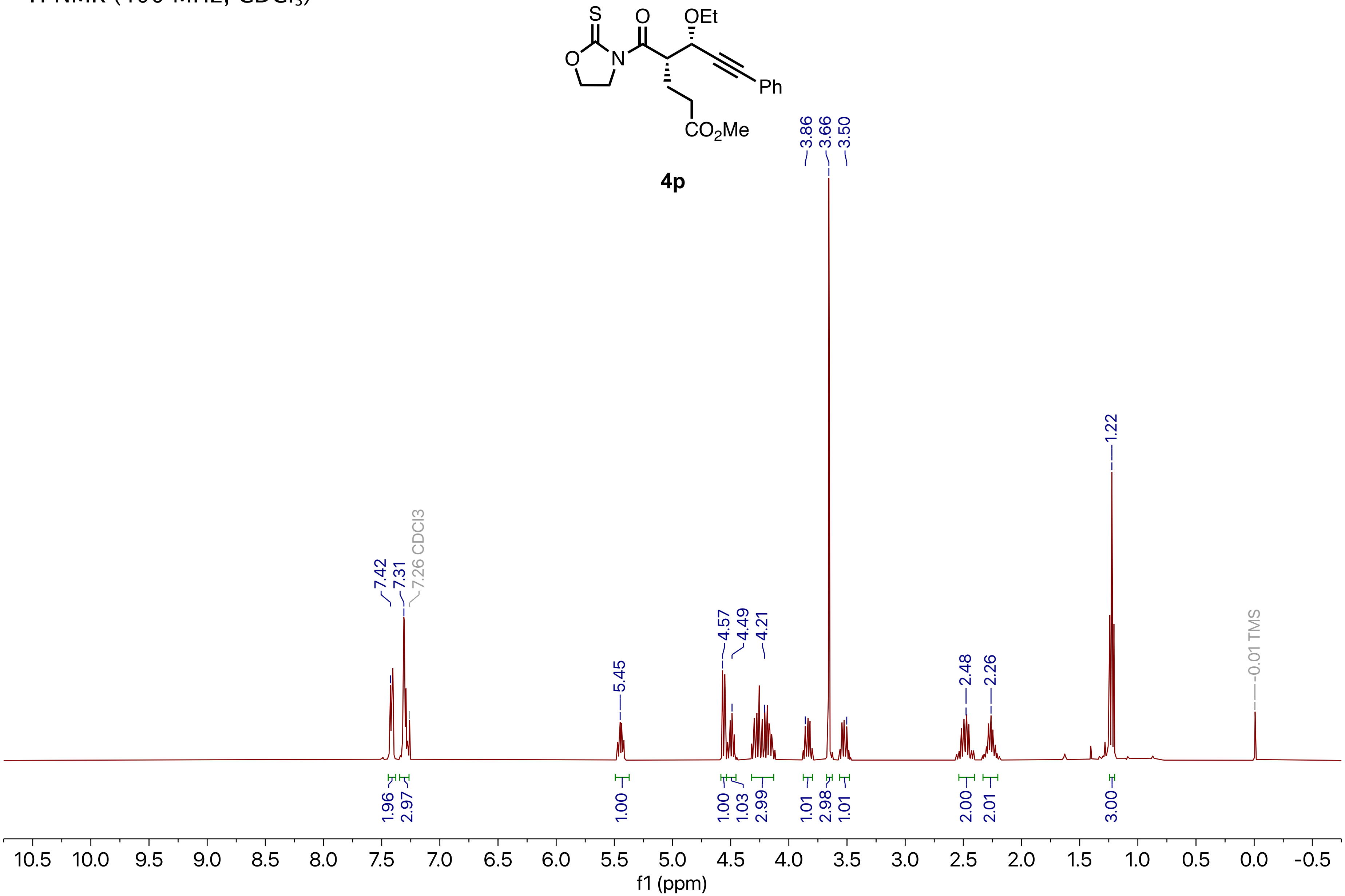

$^{13}C\{^1H\}$ NMR (101 MHz, $CDCl_3$)

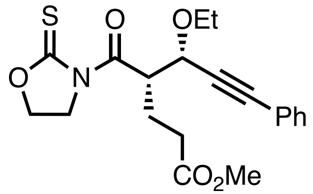

4p

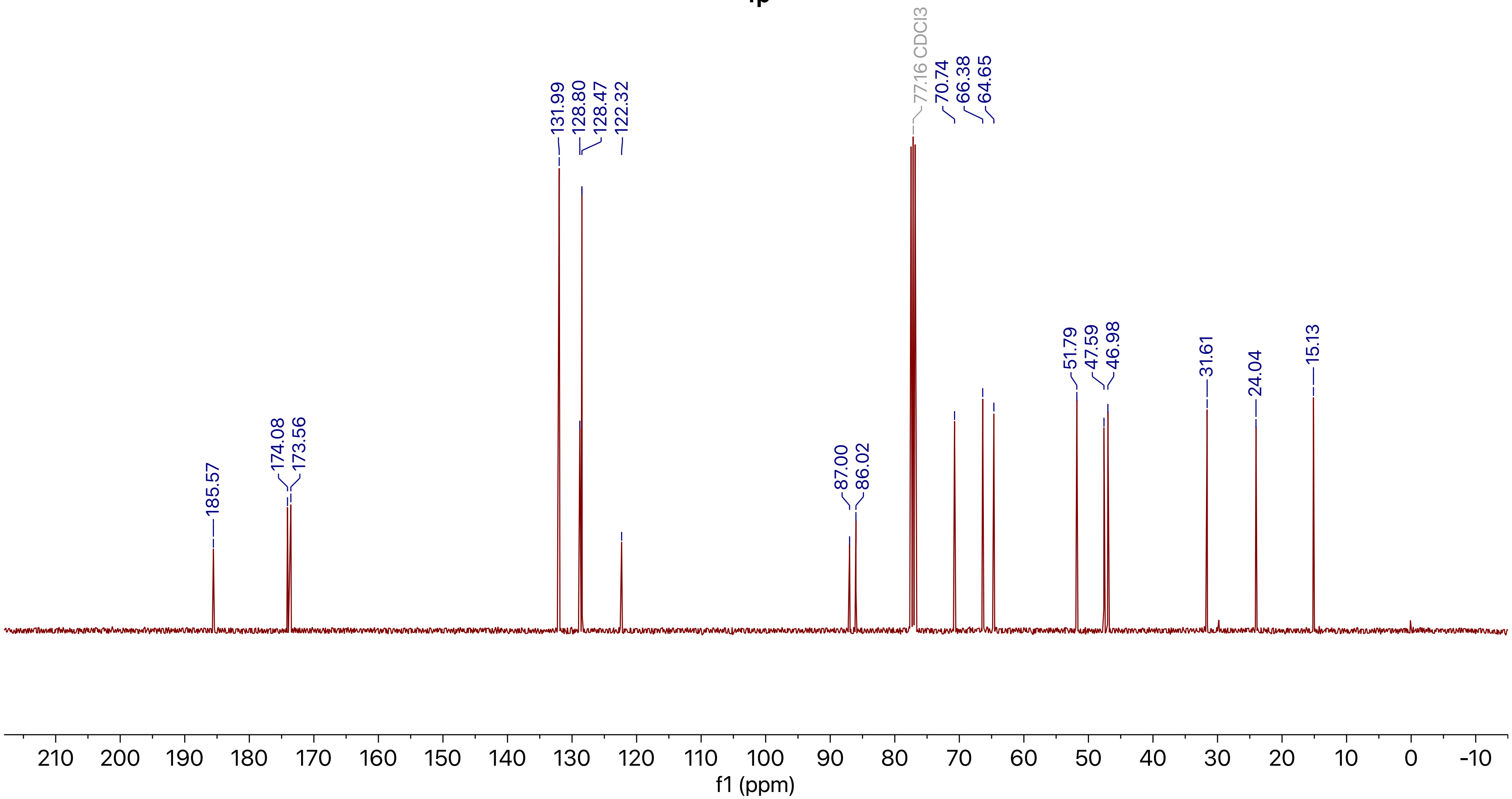

2D ^1H–^1H COSY (400 MHz, $CDCl_3$)

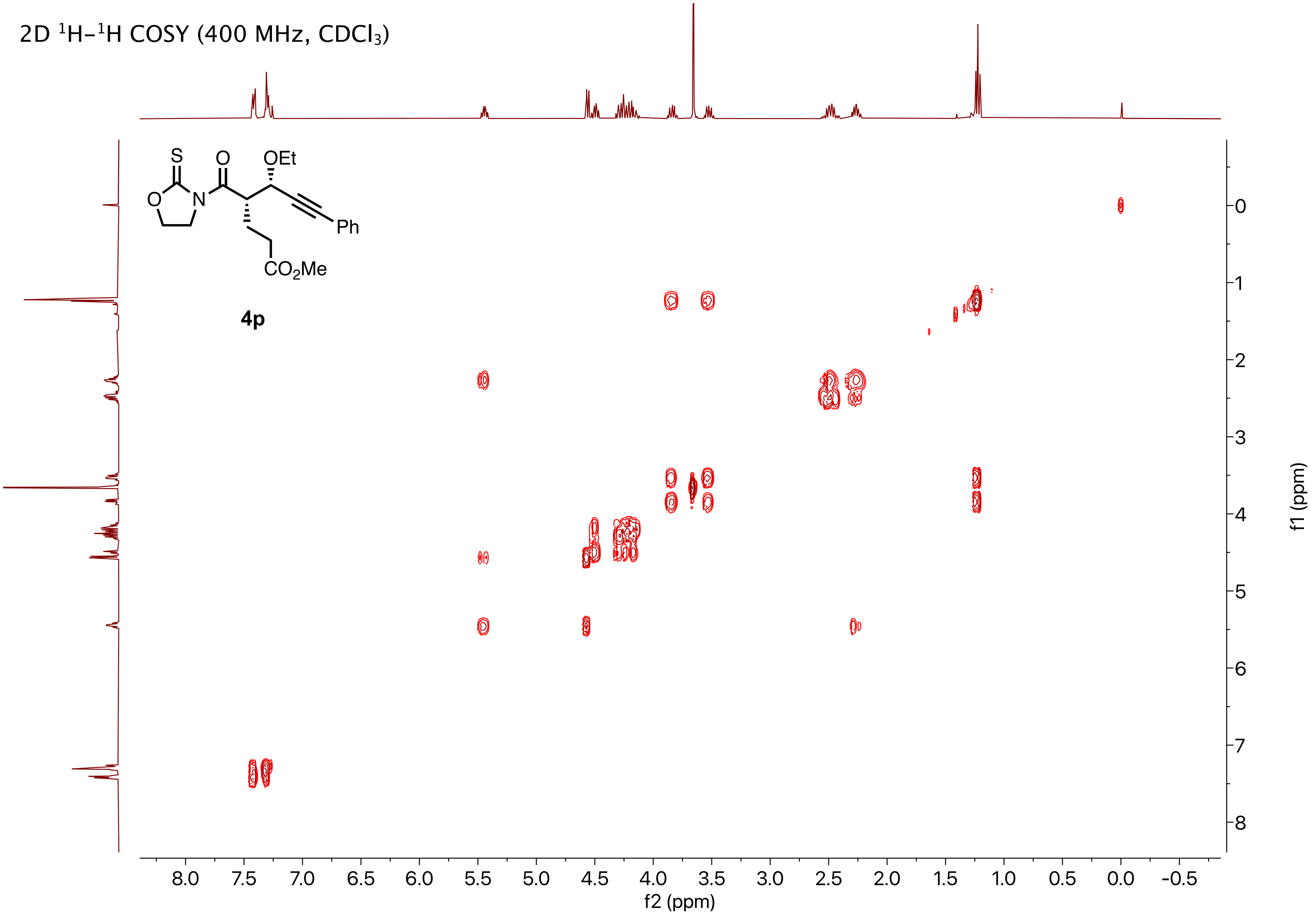

4p

2D ^{1}H–^{13}C HSQC (400 MHz, $CDCl_3$)

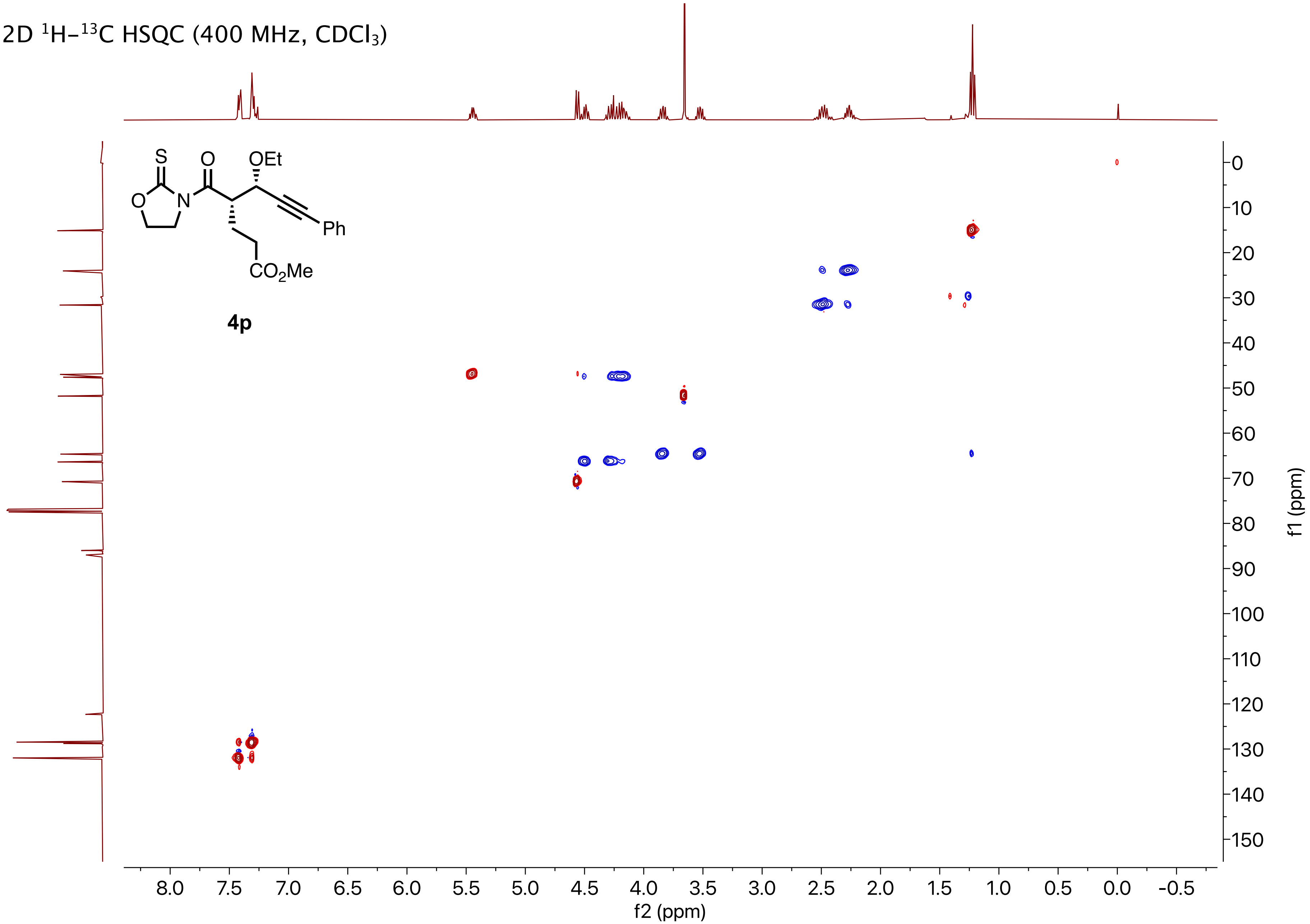

^{1}H NMR (400 MHz, $CDCl_3$)

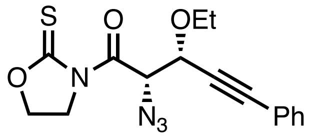

4q

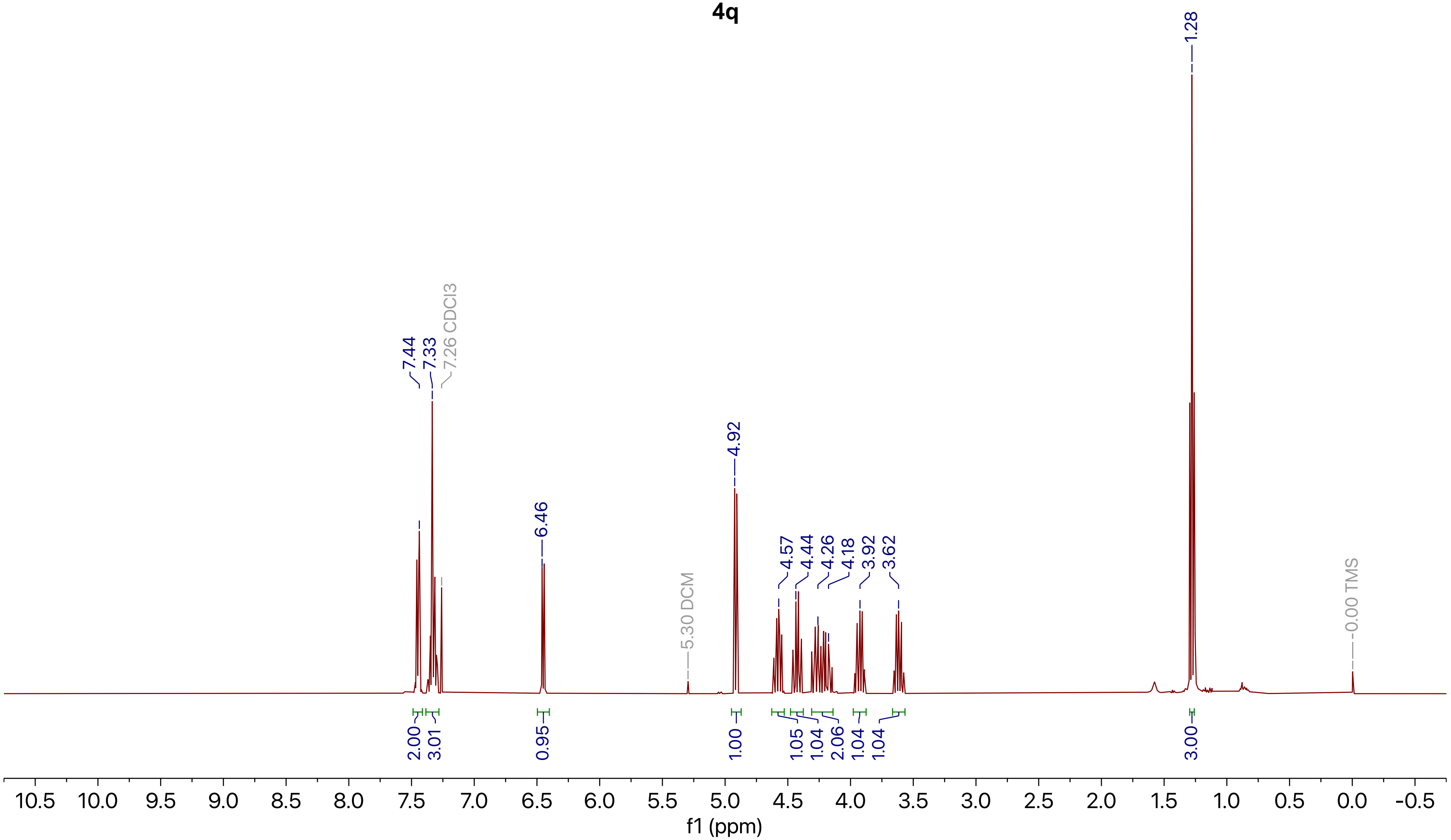

$^{13}C\{^{1}H\}$ NMR (101 MHz, $CDCl_3$)

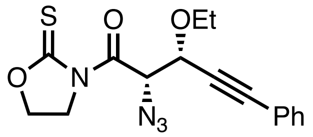

4q

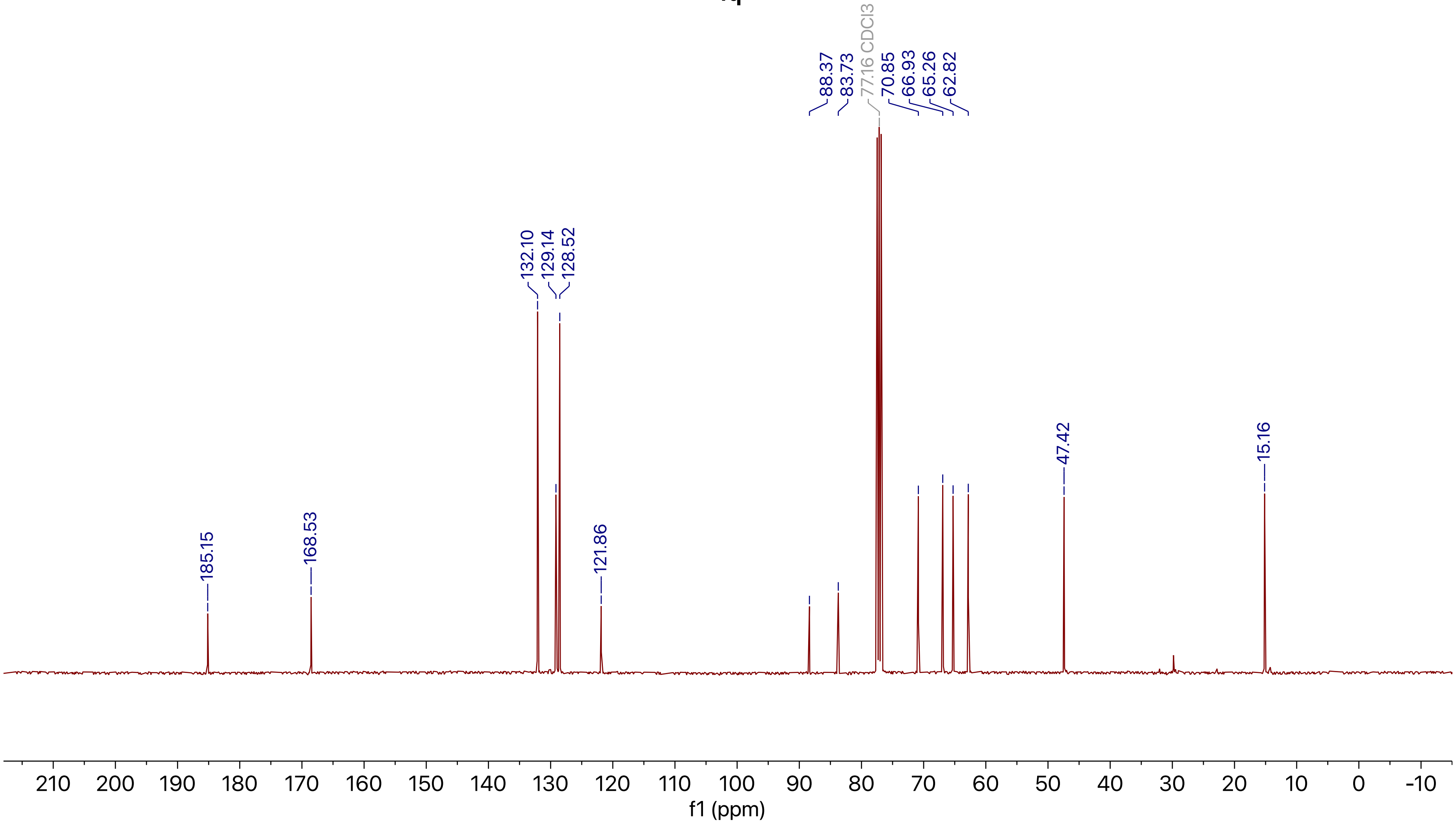

2D ^{1}H–^{1}H COSY (400 MHz, $CDCl_3$)

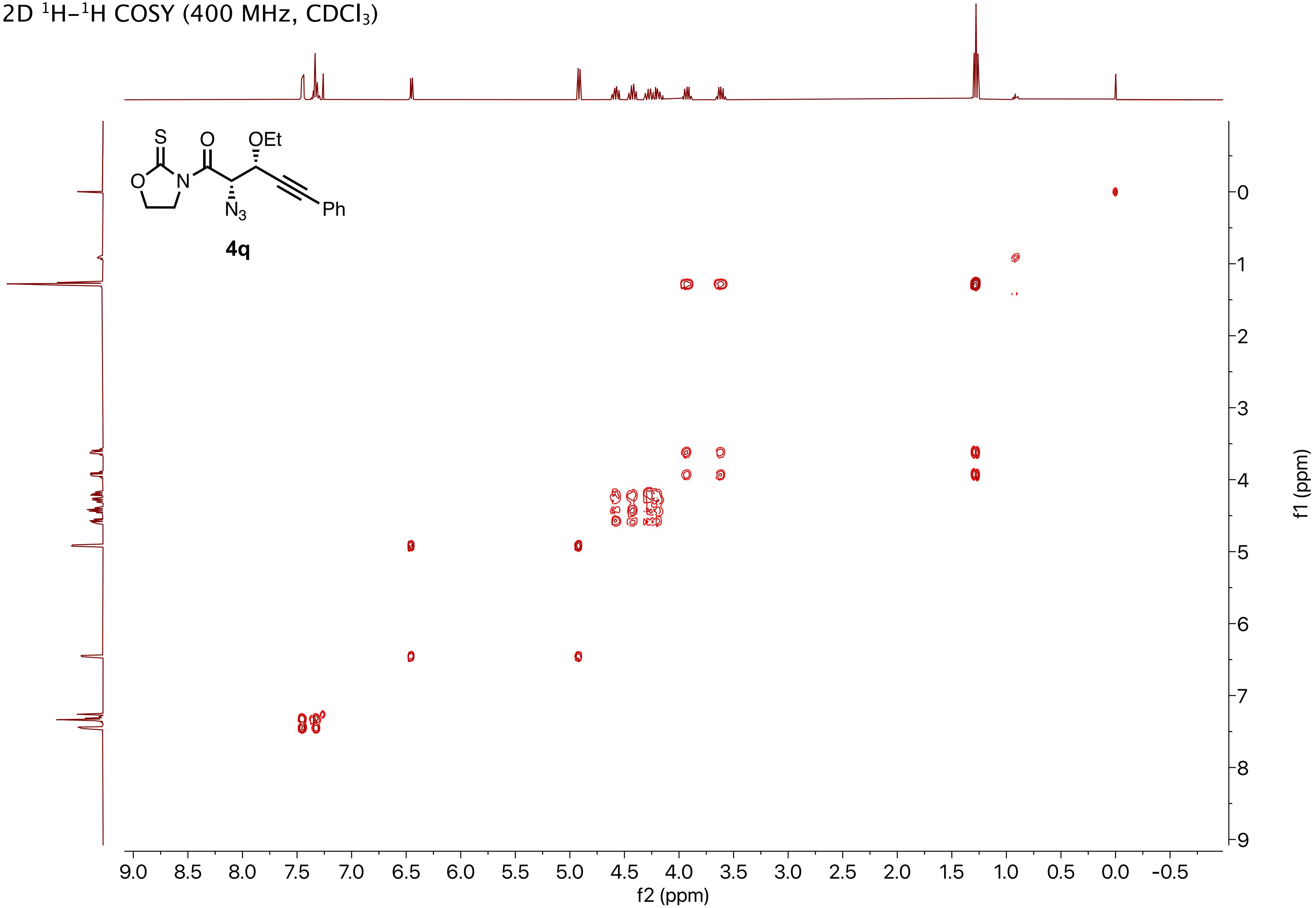

2D ^{1}H–^{13}C HSQC (400 MHz, $CDCl_3$)

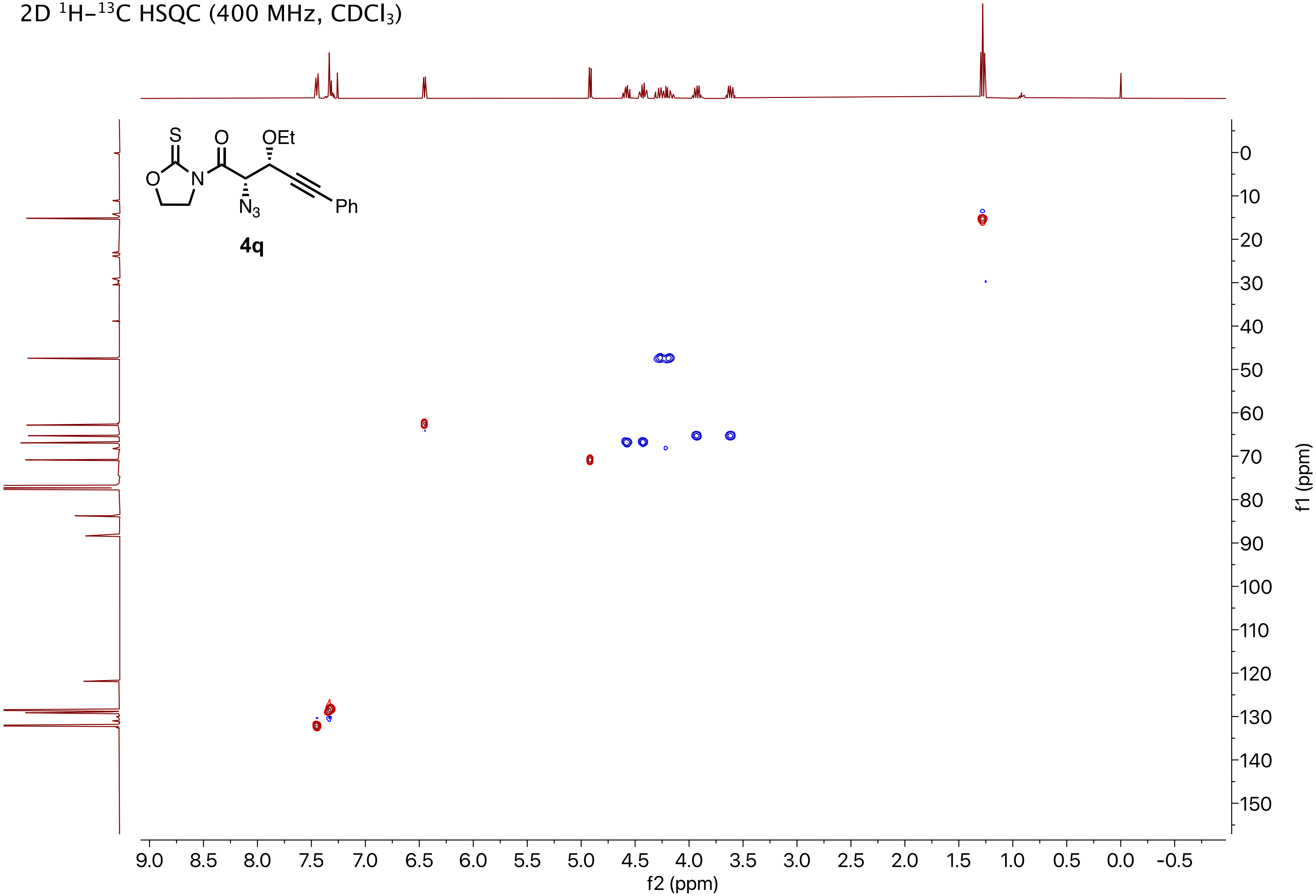

^1H NMR (400 MHz, $CDCl_3$)

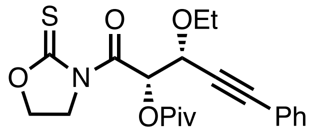

4r

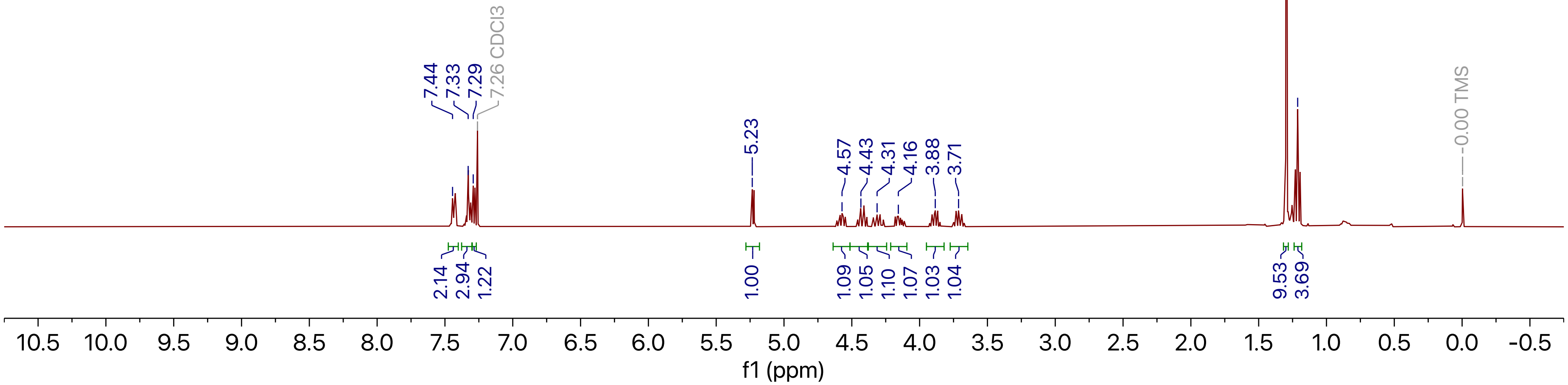

$^{13}C\{^{1}H\}$ NMR (101 MHz, $CDCl_3$)

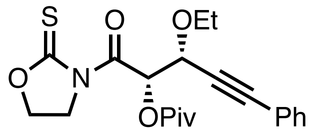

4r

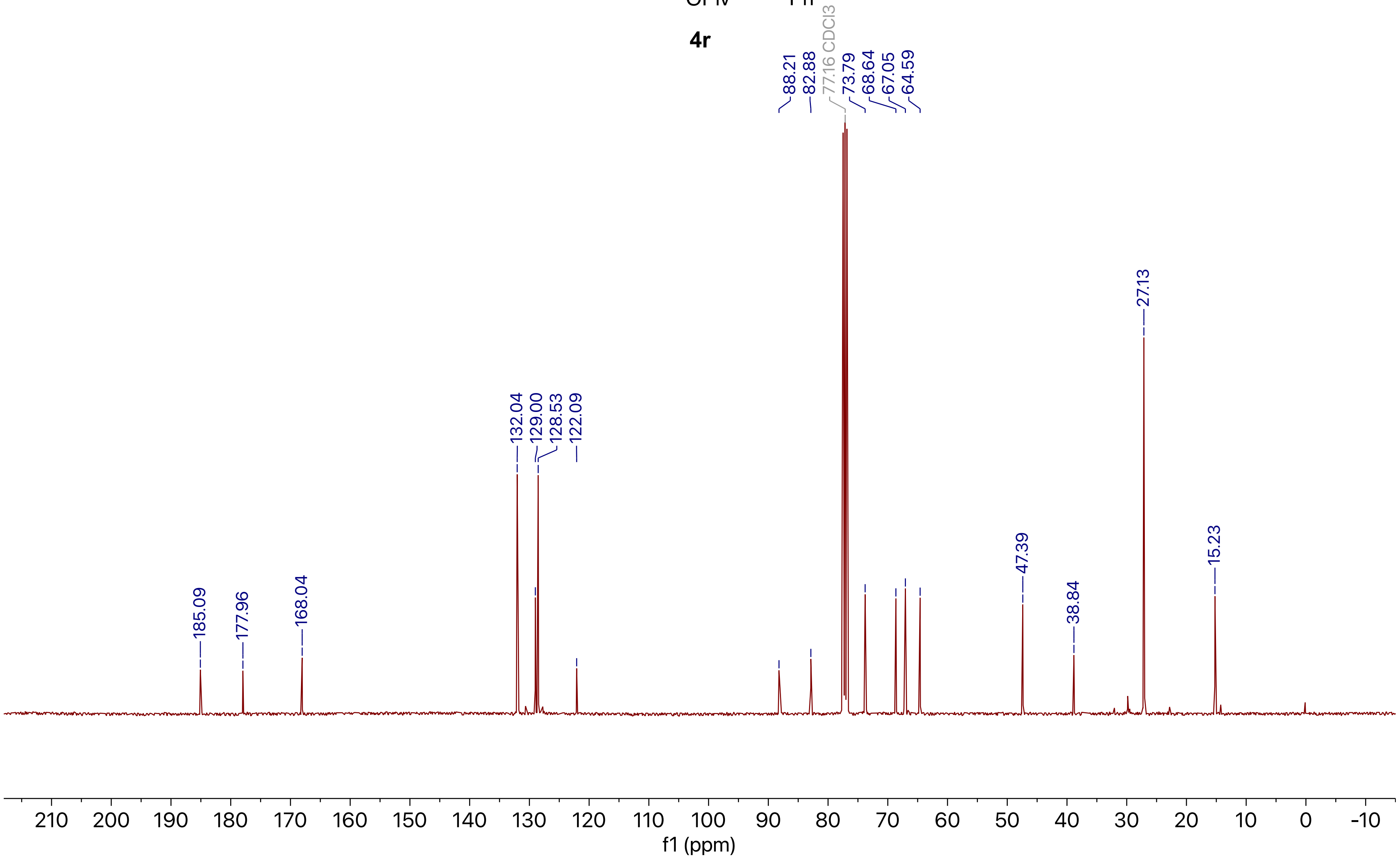

2D ^{1}H–^{1}H COSY (400 MHz, $CDCl_3$)

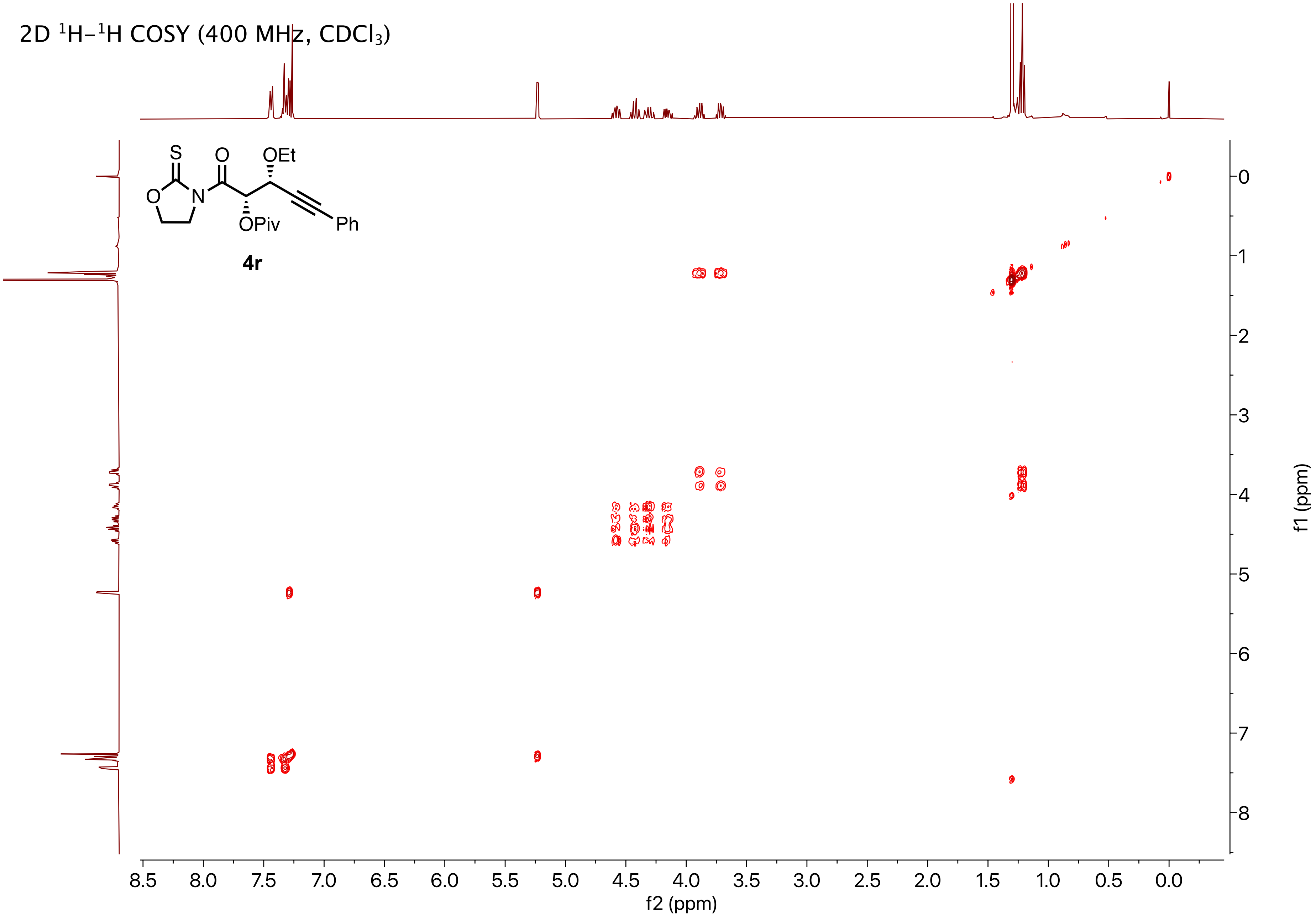

2D ^{1}H–^{13}C HSQC (400 MHz, $CDCl_3$)

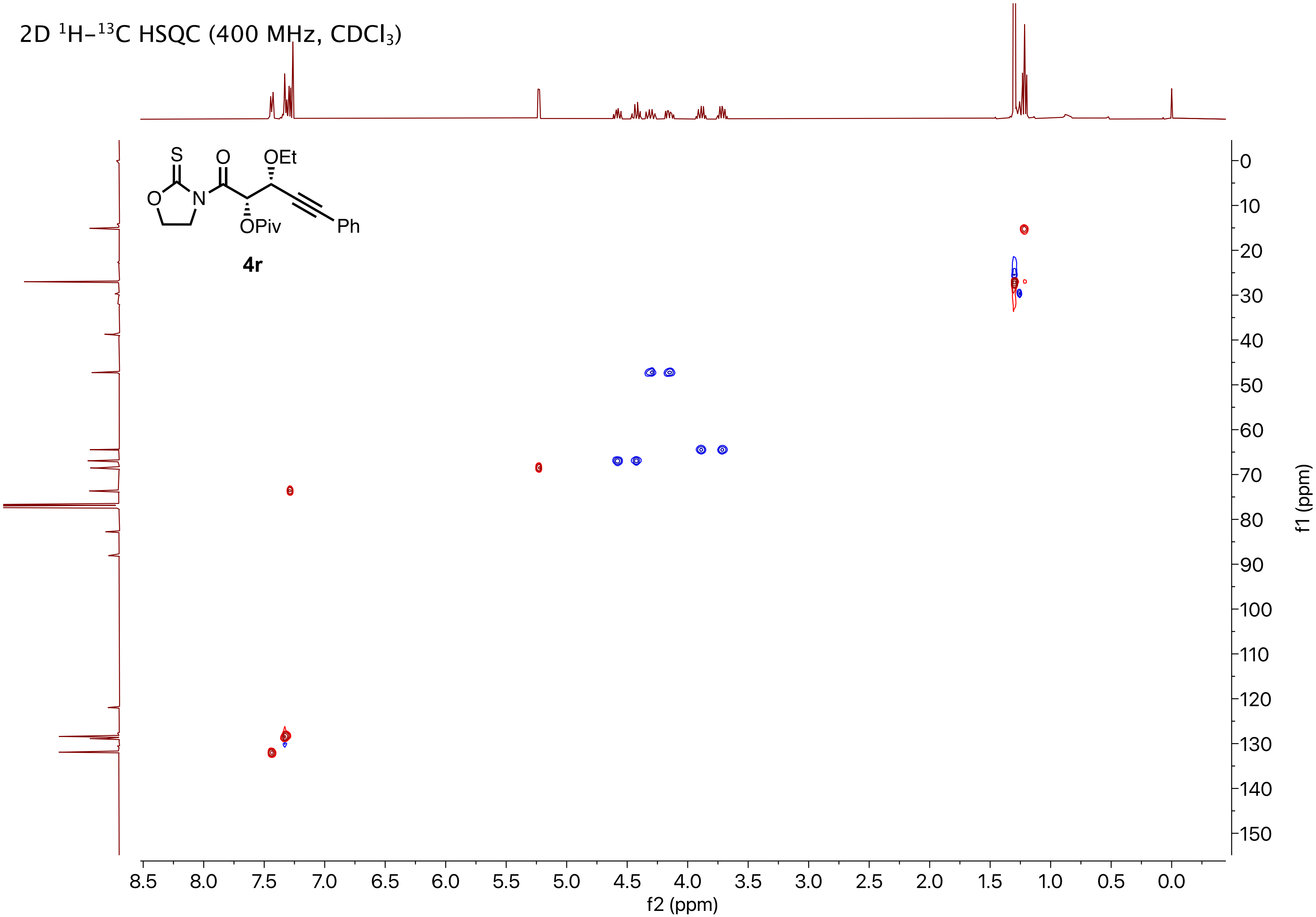

^{1}H NMR (400 MHz, $CDCl_3$)

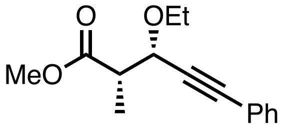

6a

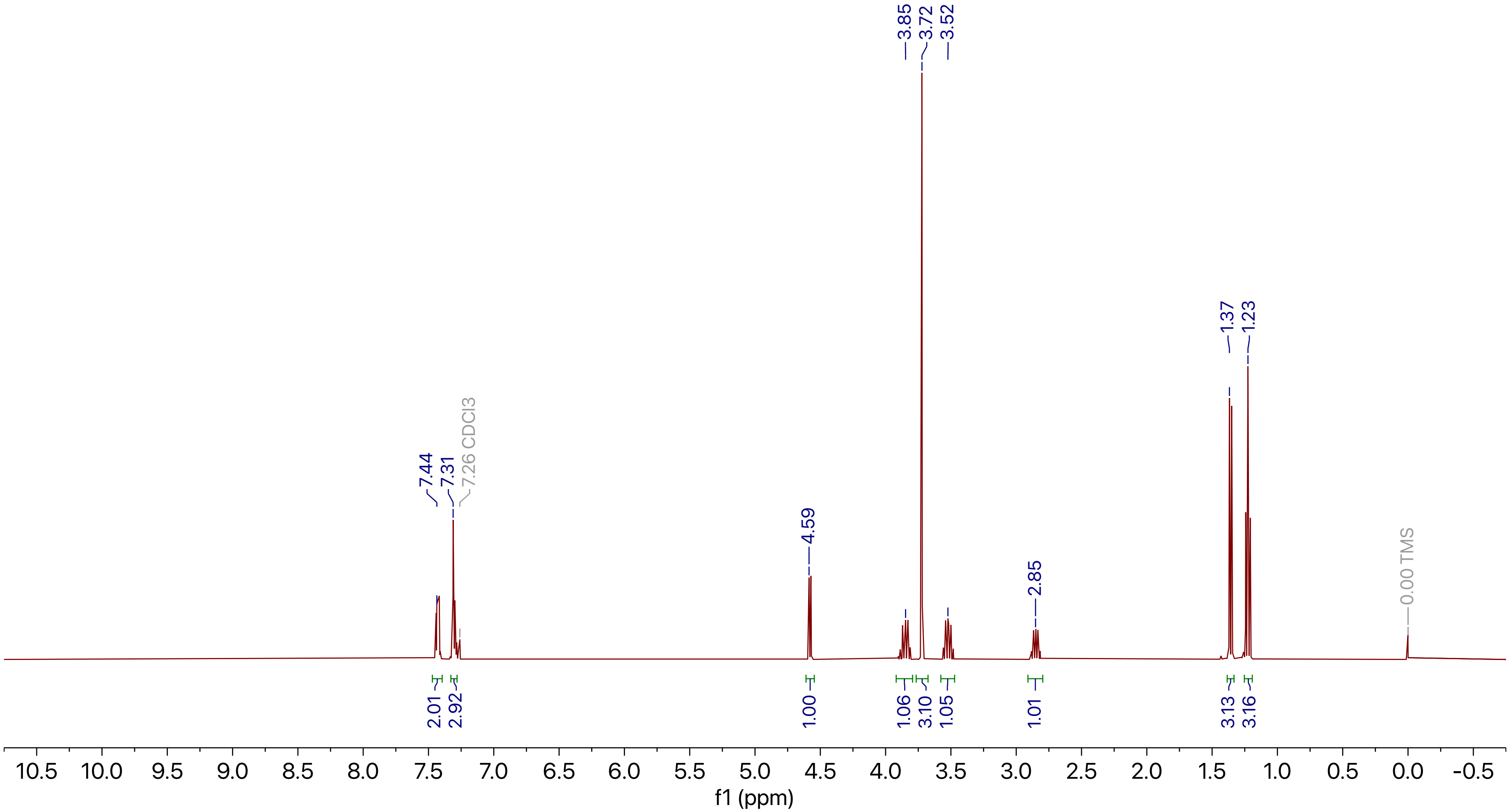

$^{13}C\{^{1}H\}$ NMR (101 MHz, $CDCl_3$)

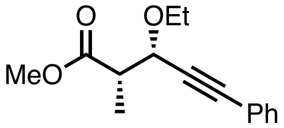

6a

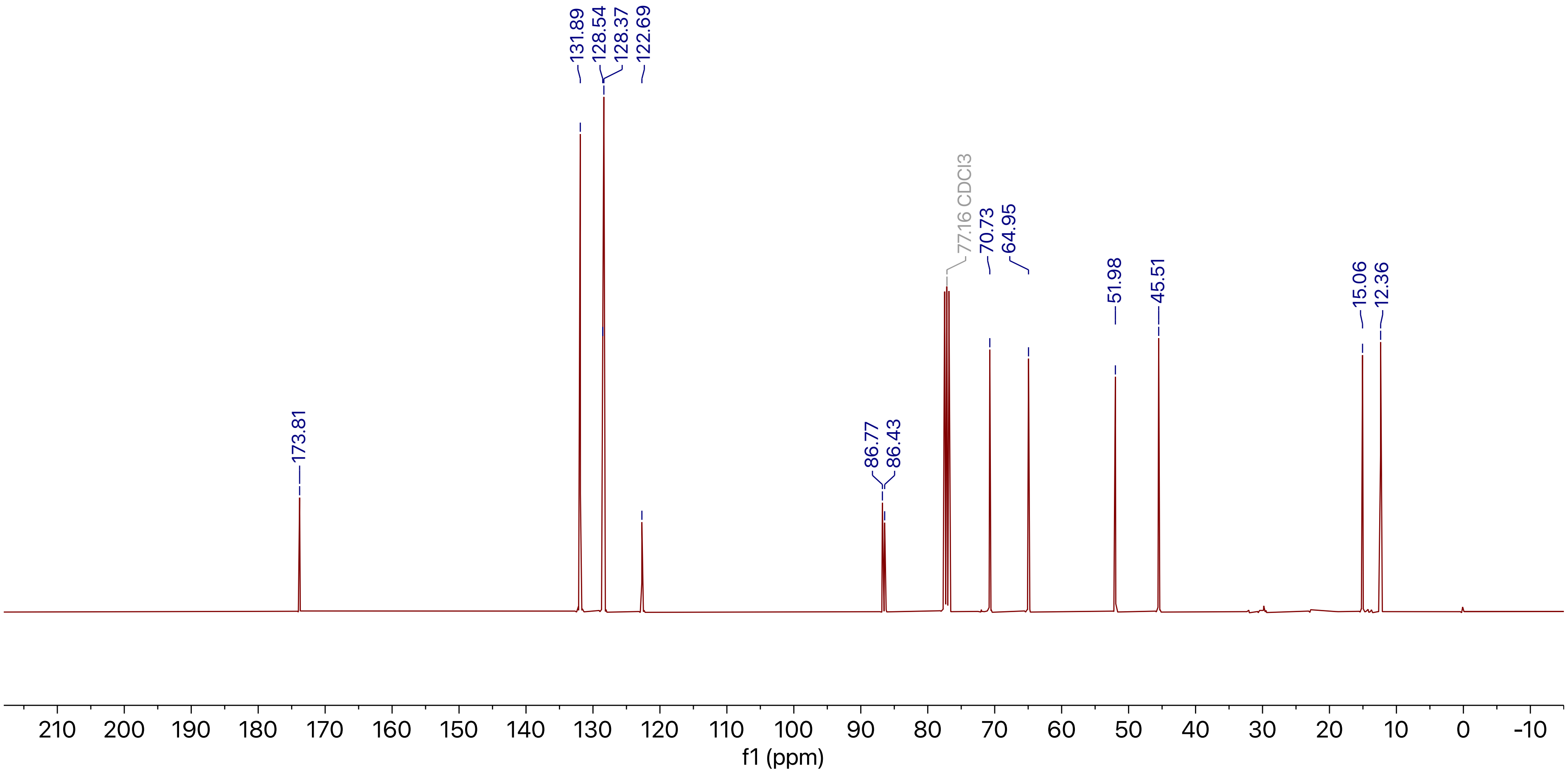

2D ^{1}H–^{1}H COSY (400 MHz, $CDCl_3$)

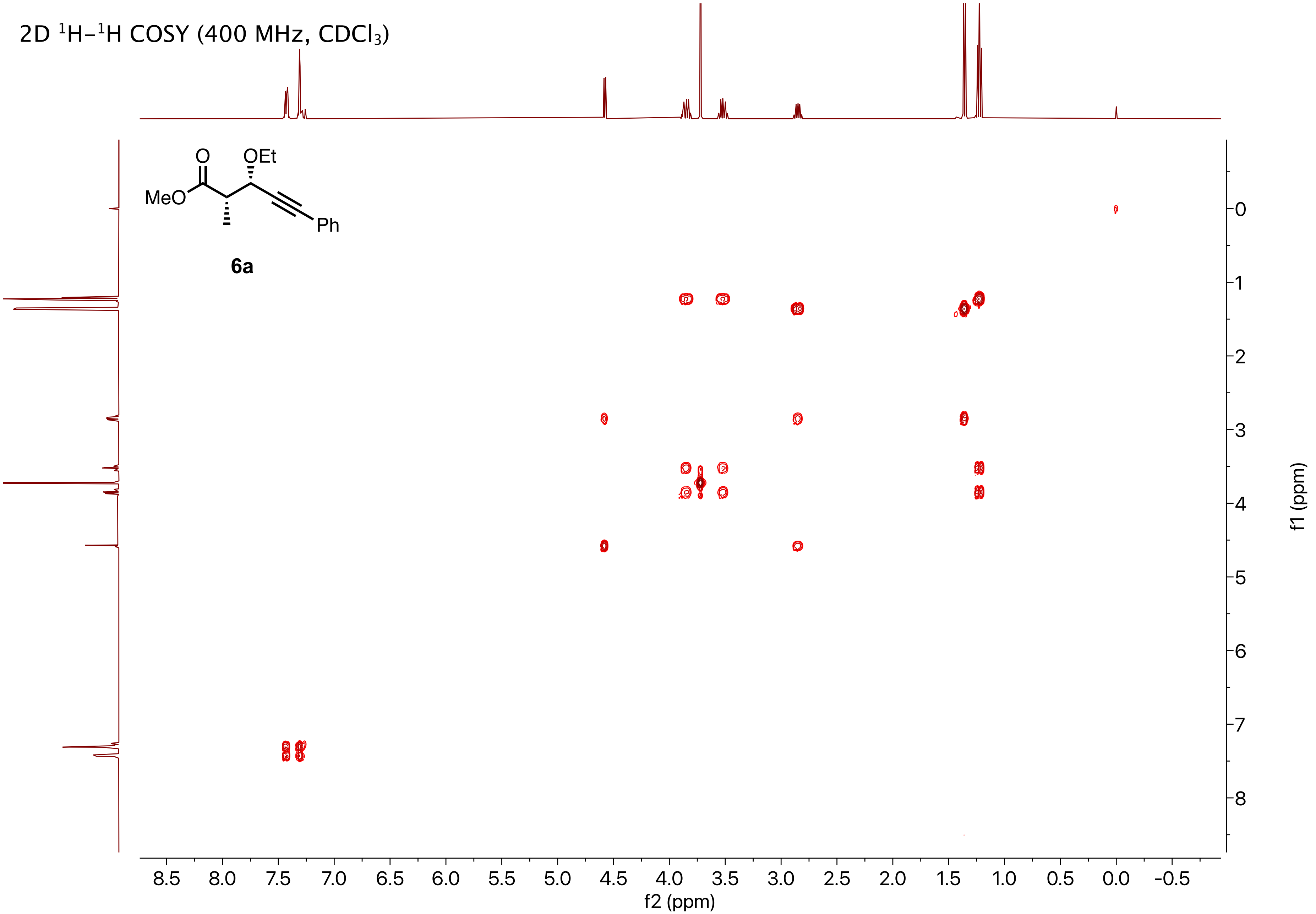

2D ^{1}H–^{13}C HSQC (400 MHz, $CDCl_3$)

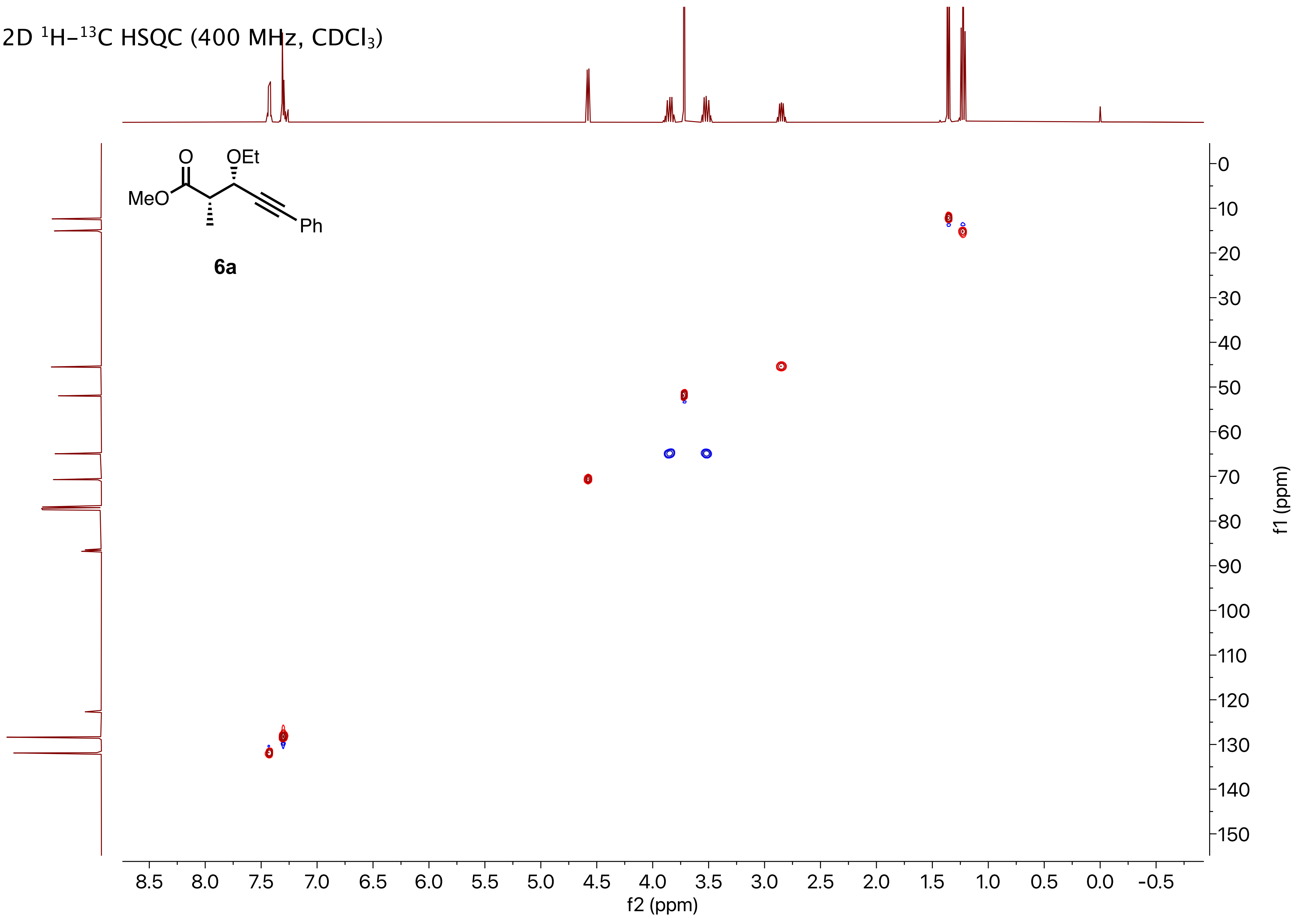

^{1}H NMR (400 MHz, $CDCl_3$)

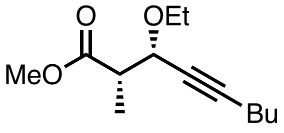

6e

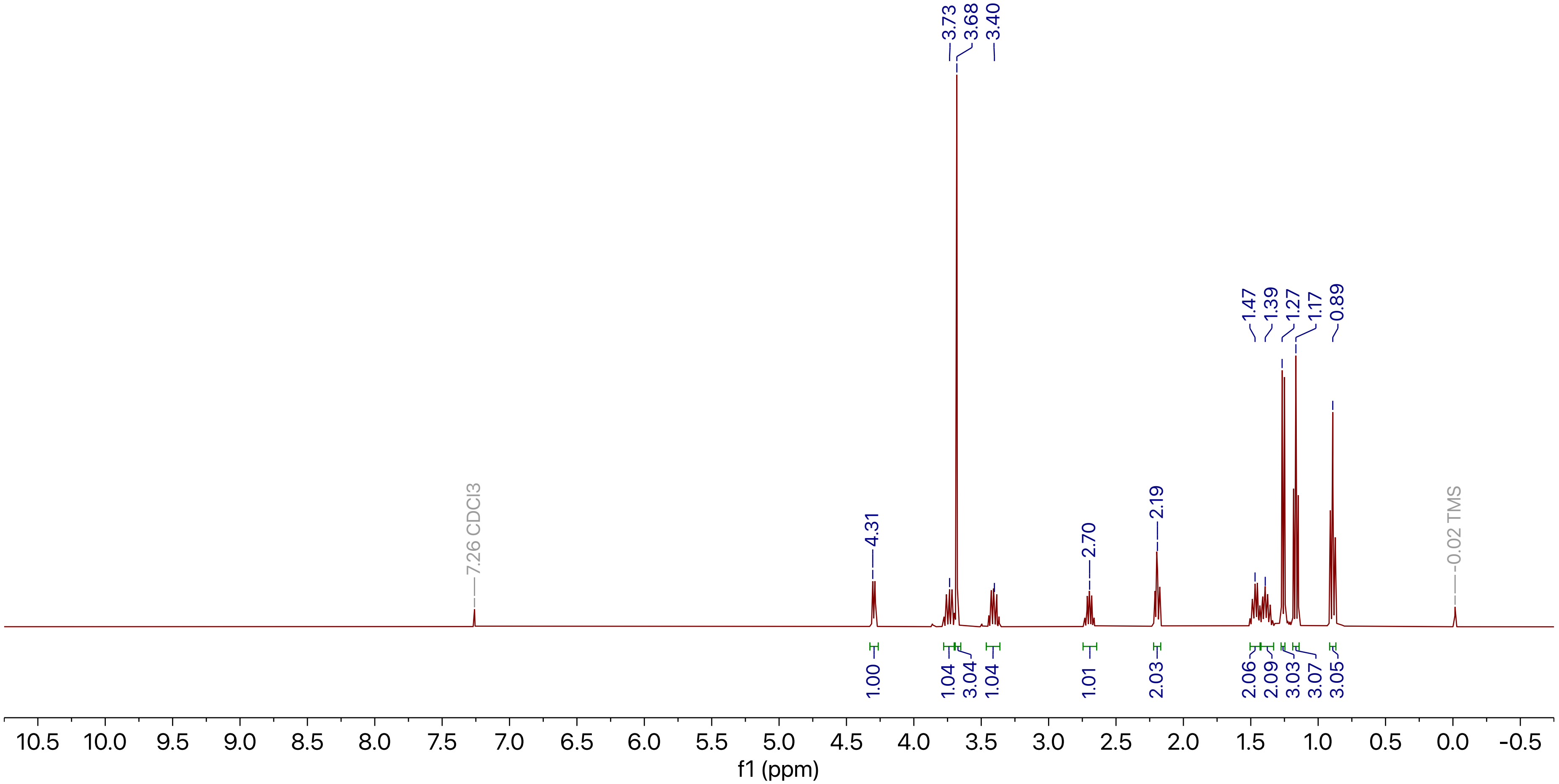

$^{13}C\{^{1}H\}$ NMR (101 MHz, $CDCl_3$)

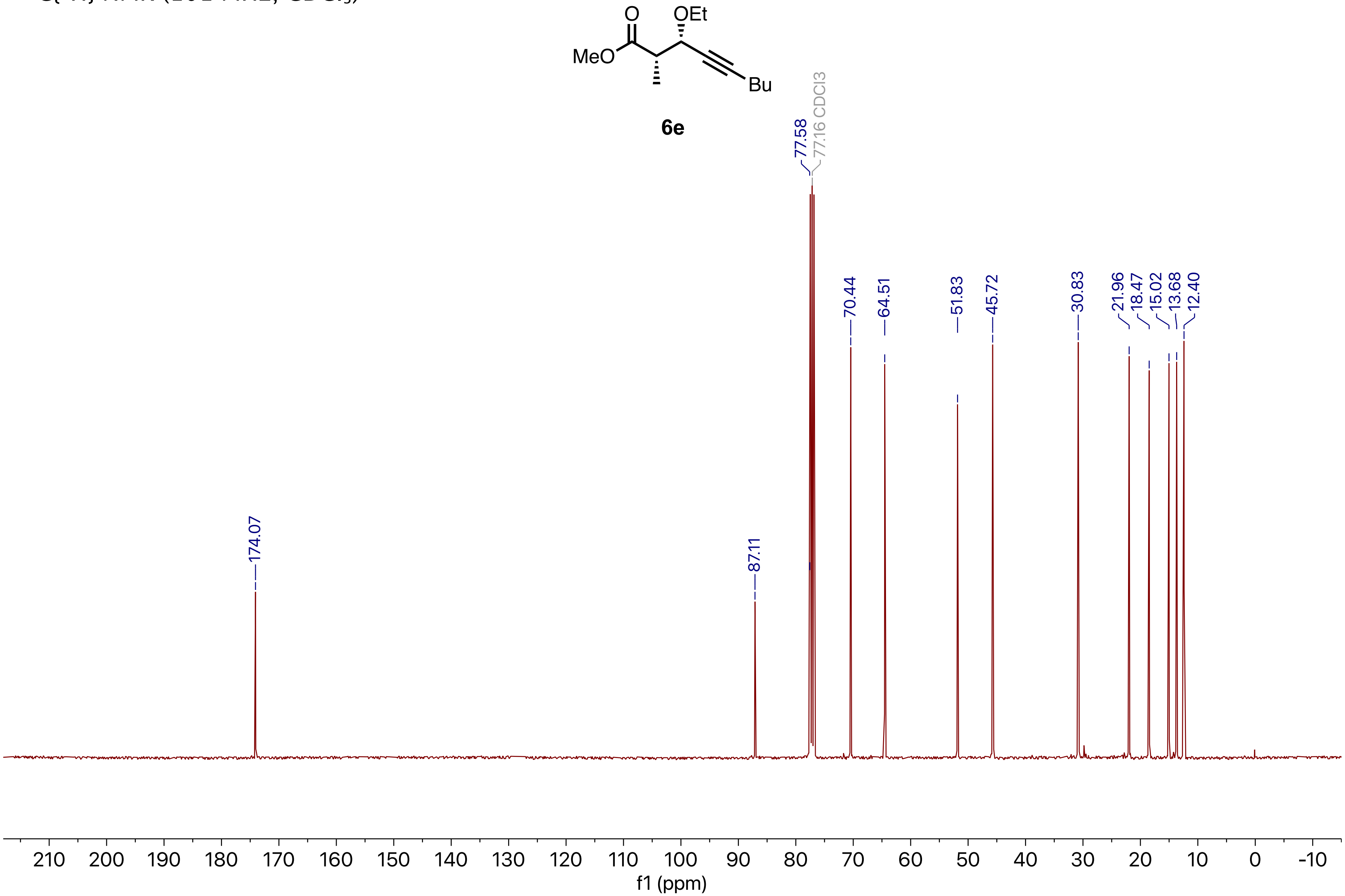

2D ^{1}H–^{1}H COSY (400 MHz, $CDCl_3$)

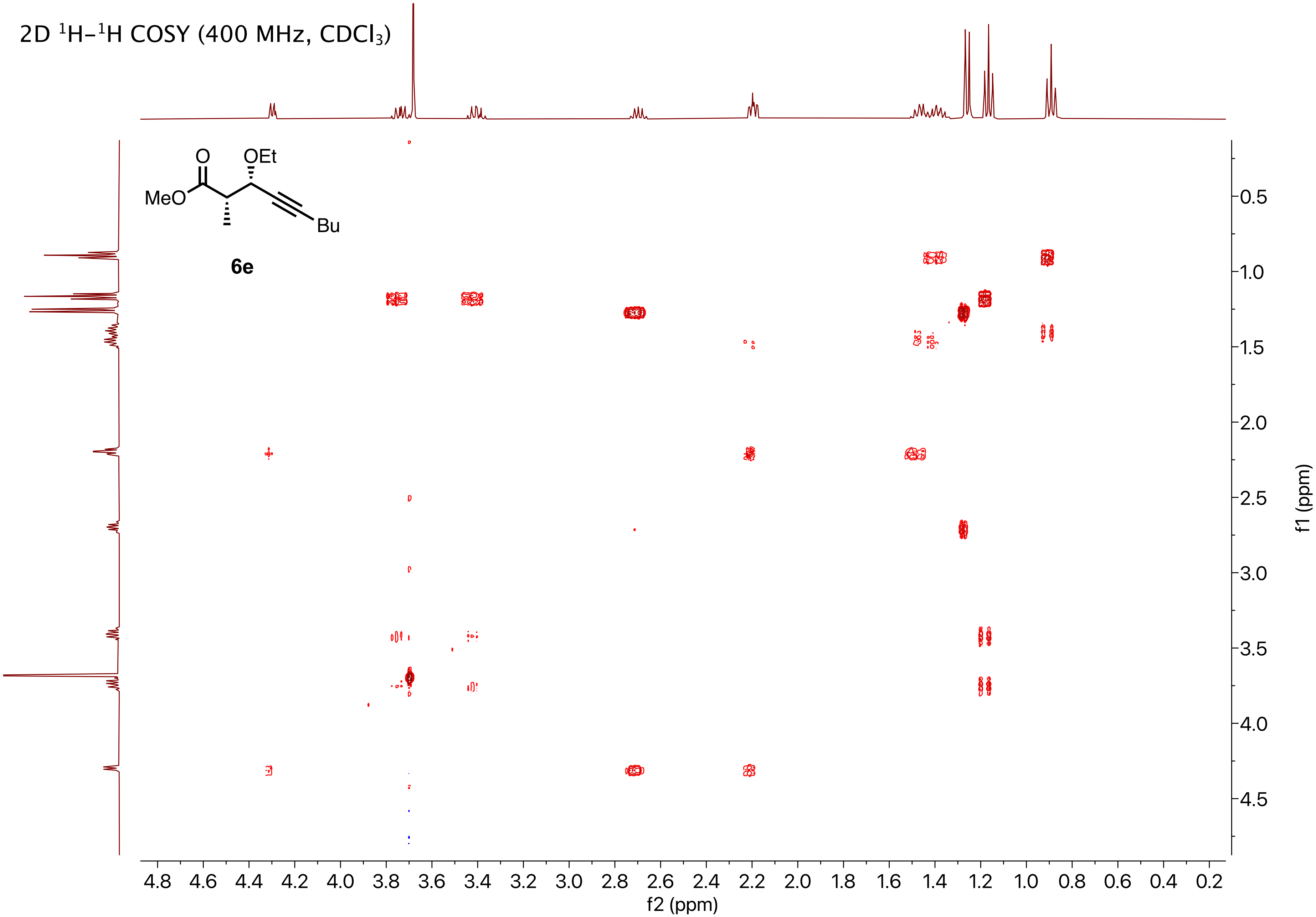

2D ^1H–^{13}C HSQC (400 MHz, $CDCl_3$)

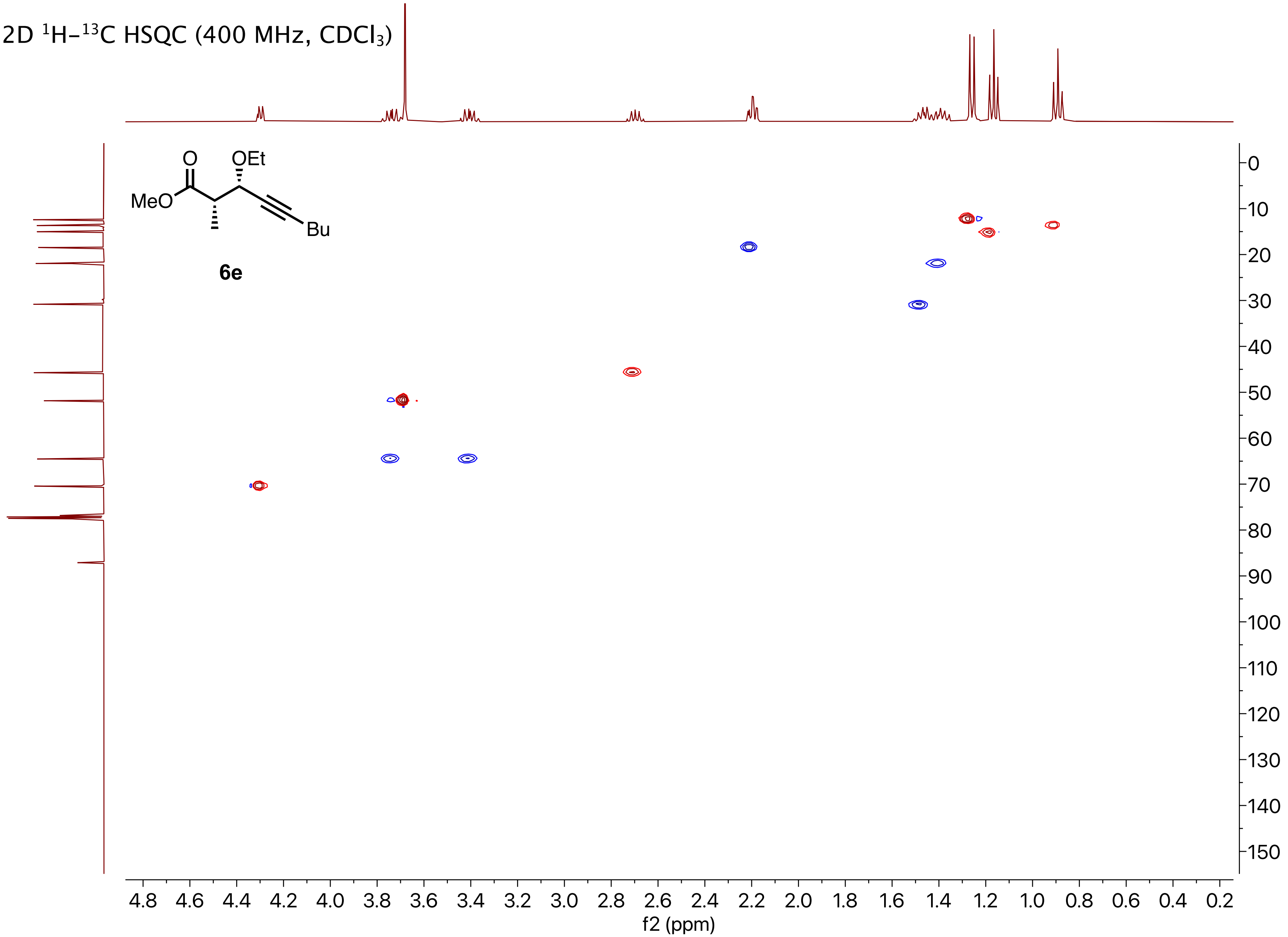

^{1}H NMR (400 MHz, $CDCl_3$)

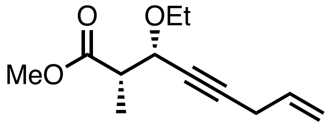

6f

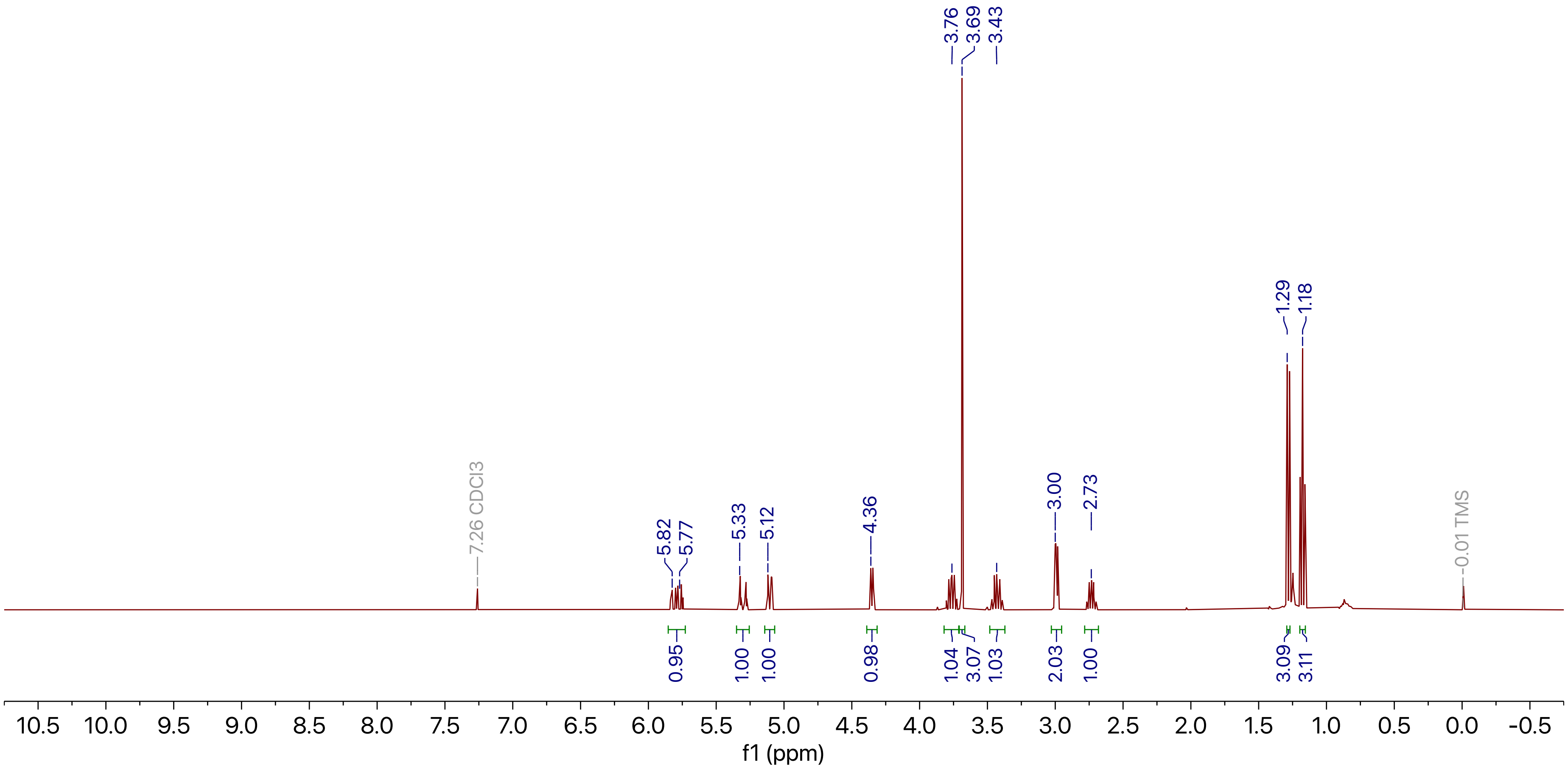

$^{13}C\{^{1}H\}$ NMR (101 MHz, $CDCl_3$)

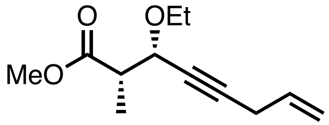

6f

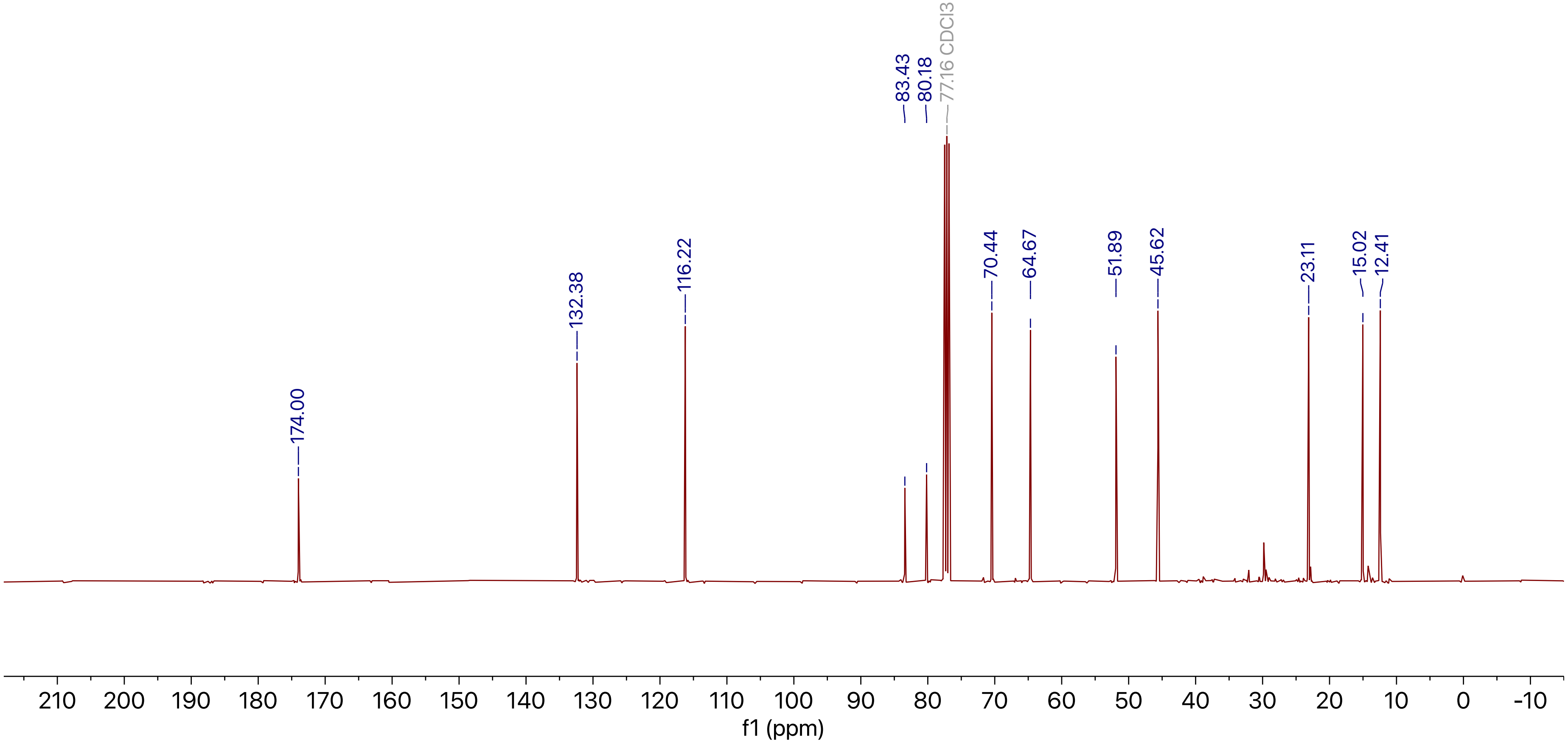

2D ^1H–^1H COSY (400 MHz, $CDCl_3$)

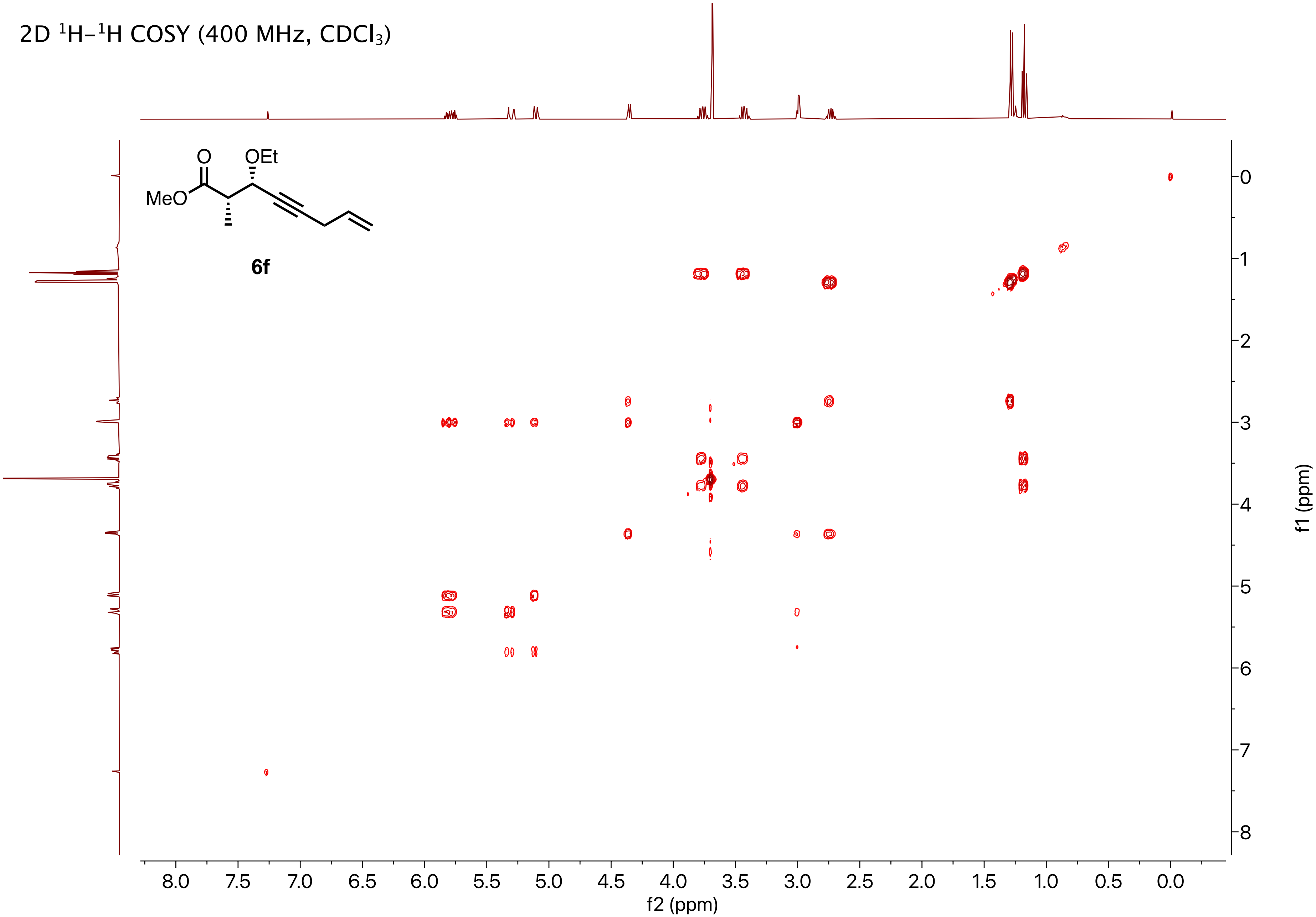

2D ^{1}H–^{13}C HSQC (400 MHz, $CDCl_3$)

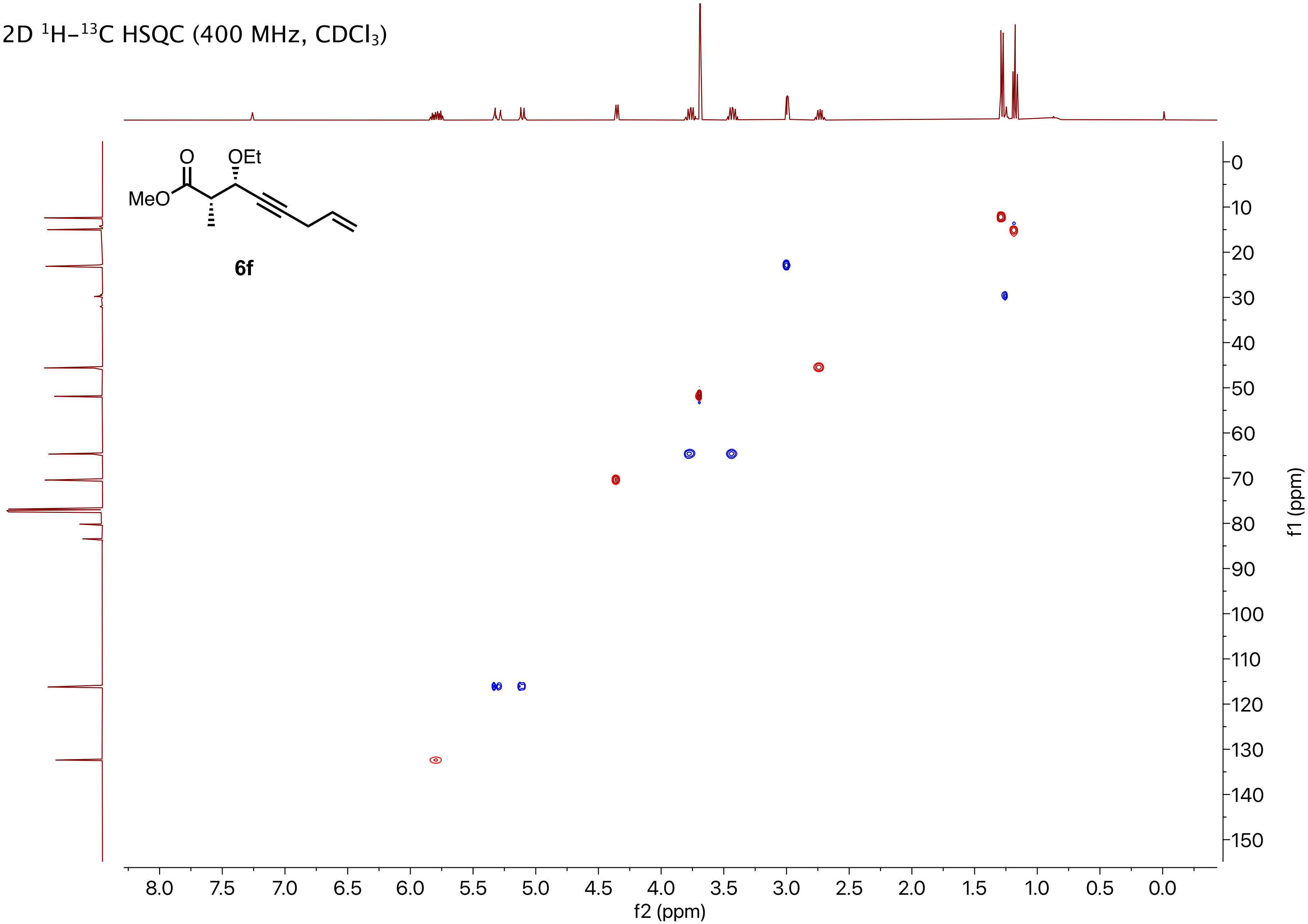

^{1}H NMR (400 MHz, $CDCl_3$)

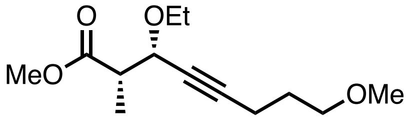

6l

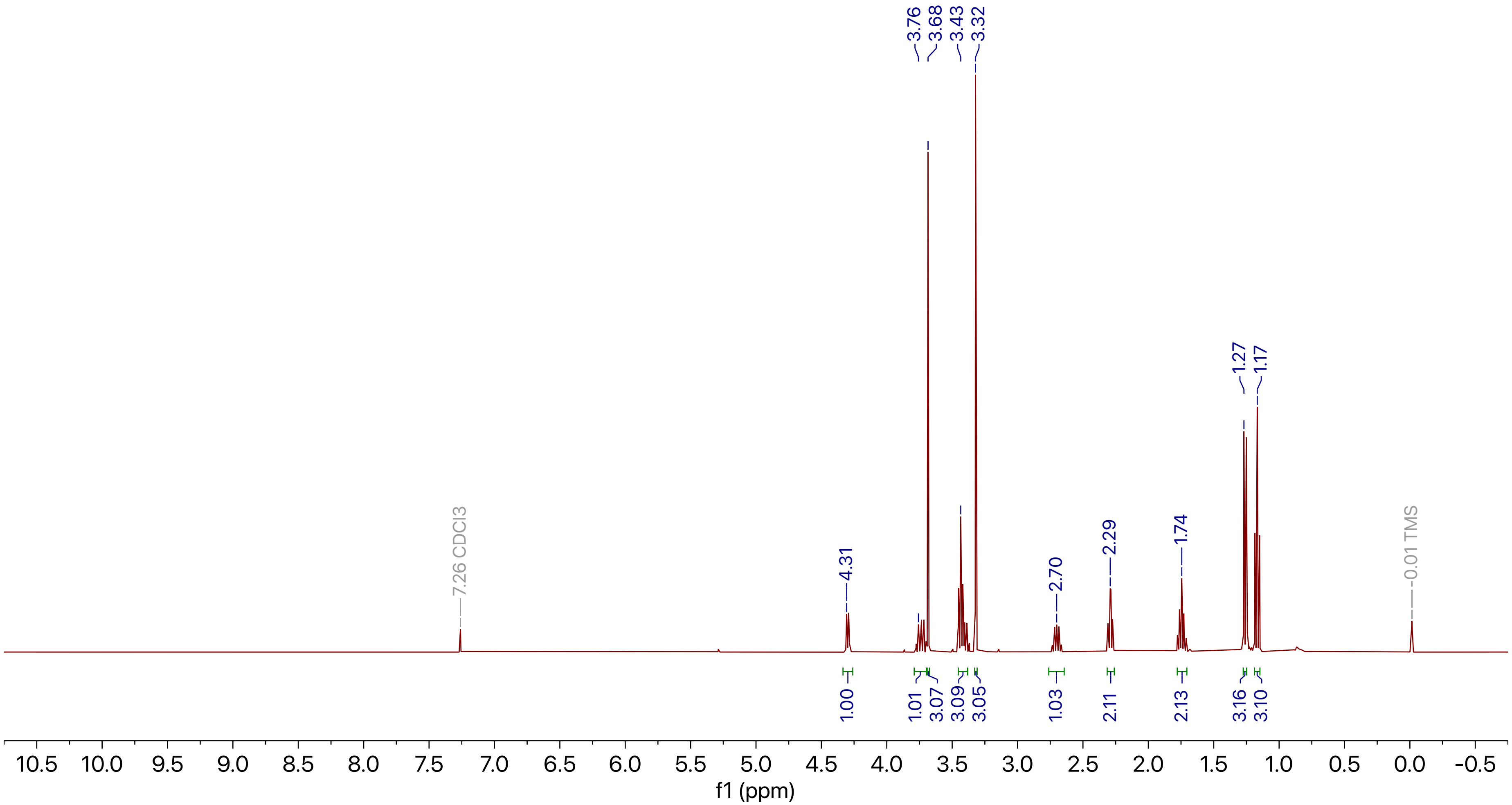

$^{13}C\{^1H\}$ NMR (101 MHz, $CDCl_3$)

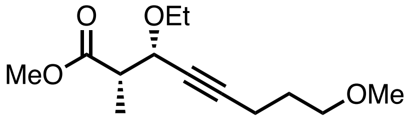

6l

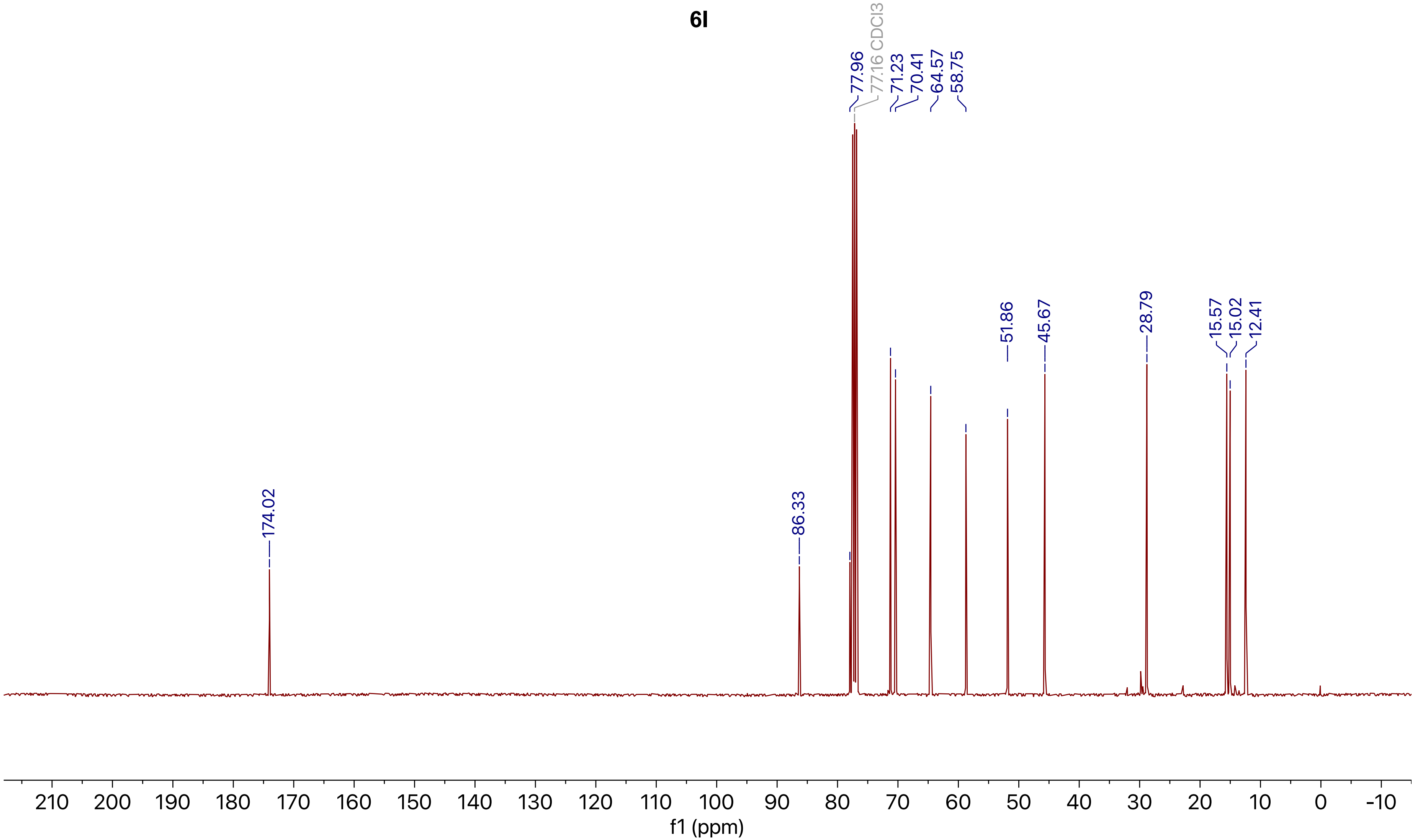

2D $^{1}H-^{1}H$ COSY (400 MHz, $CDCl_3$)

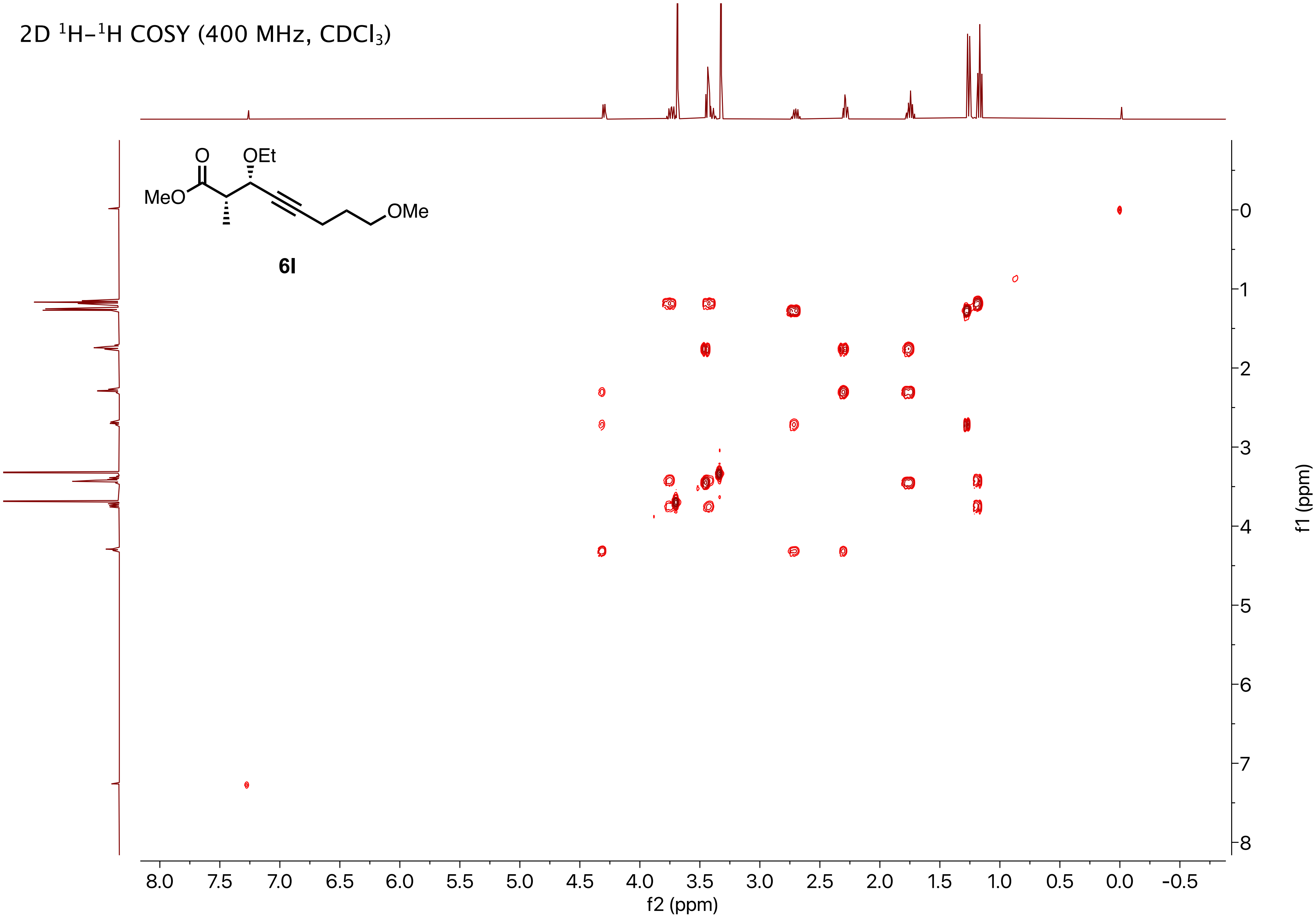

2D $^{1}H-^{13}C$ HSQC (400 MHz, $CDCl_3$)

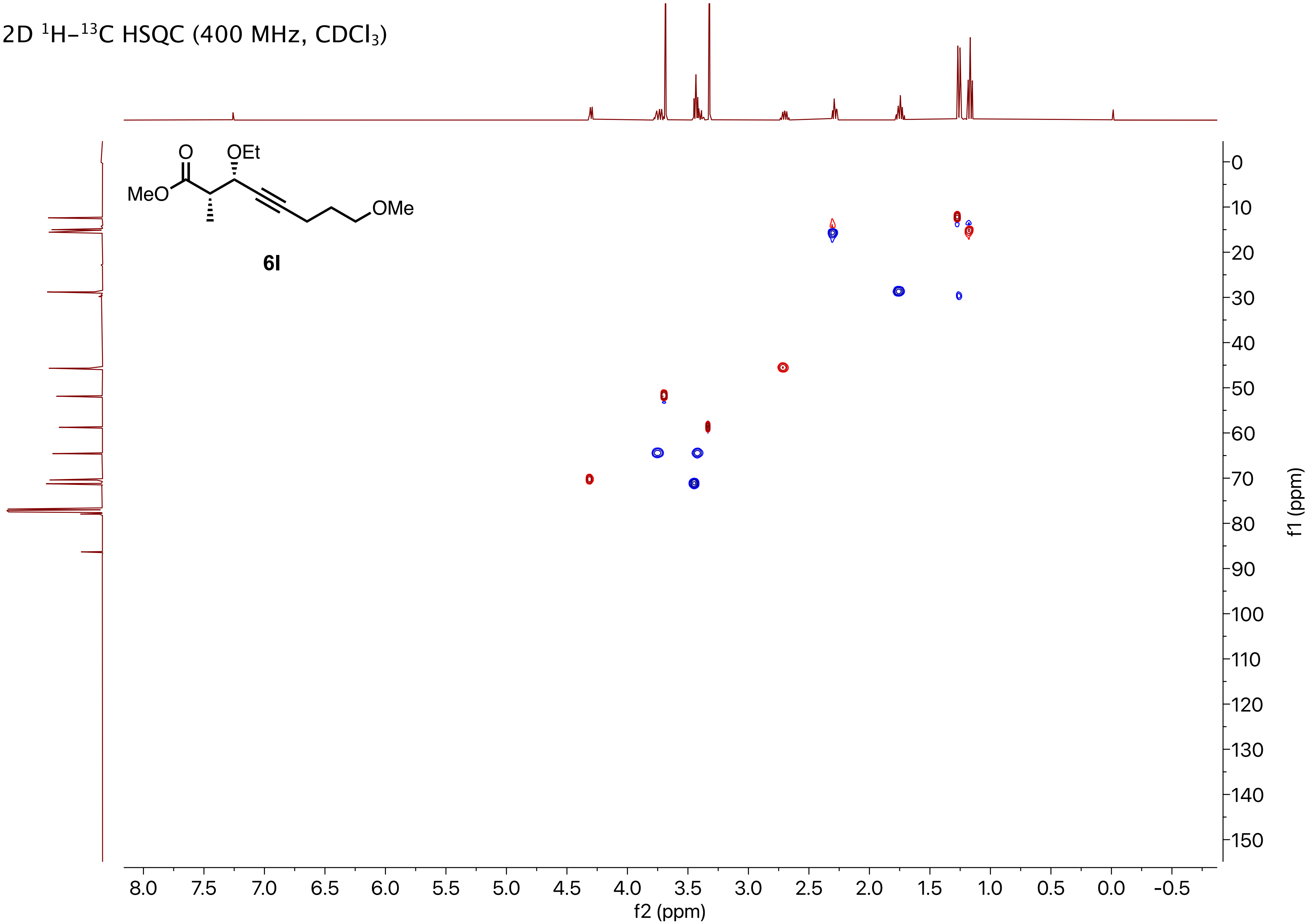

6l

^{1}H NMR (400 MHz, $CDCl_3$)

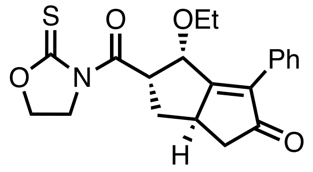

10a

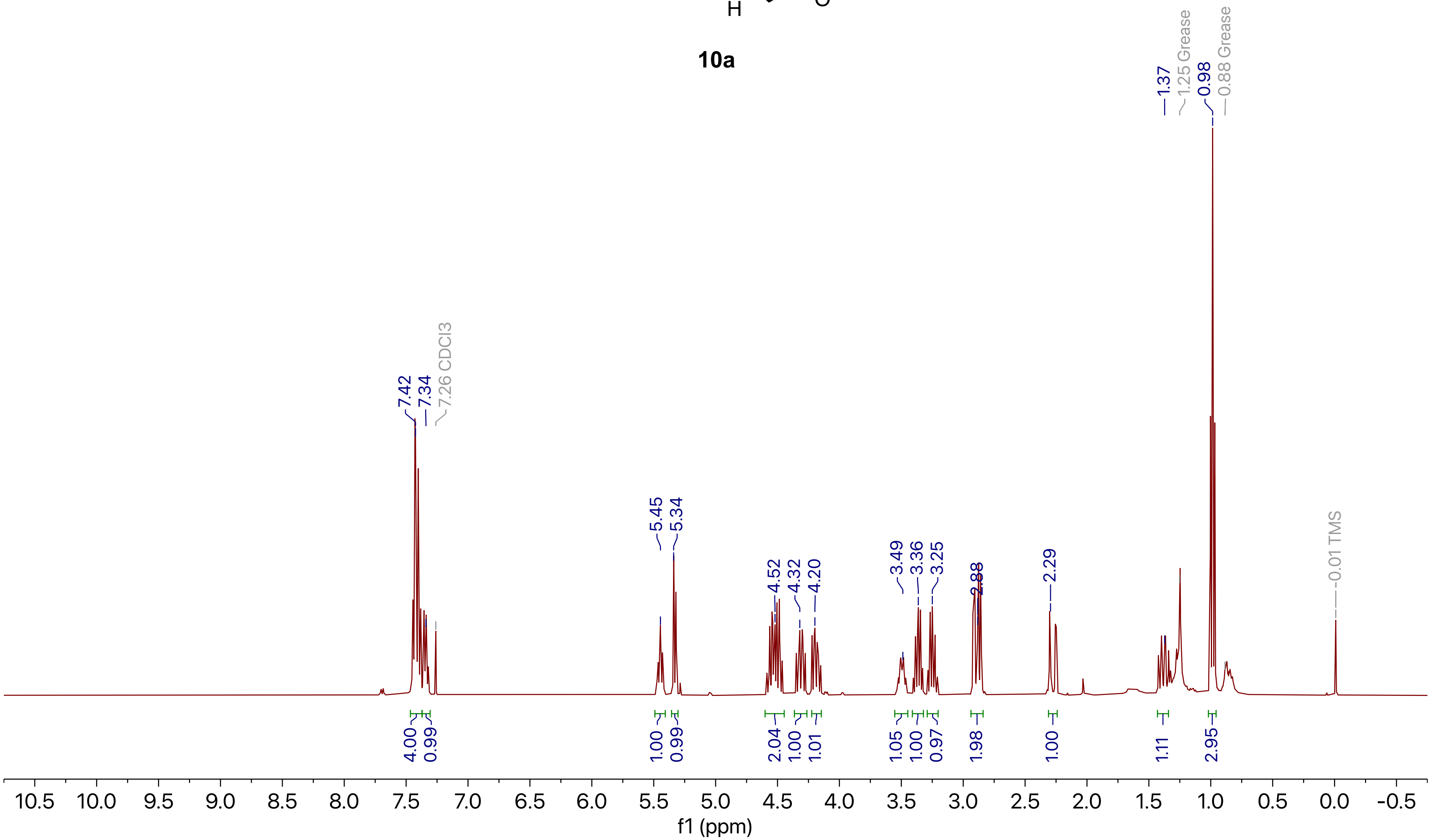

$^{13}C\{^{1}H\}$ NMR (101 MHz, $CDCl_3$)

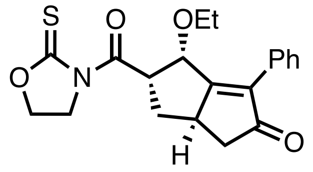

10a

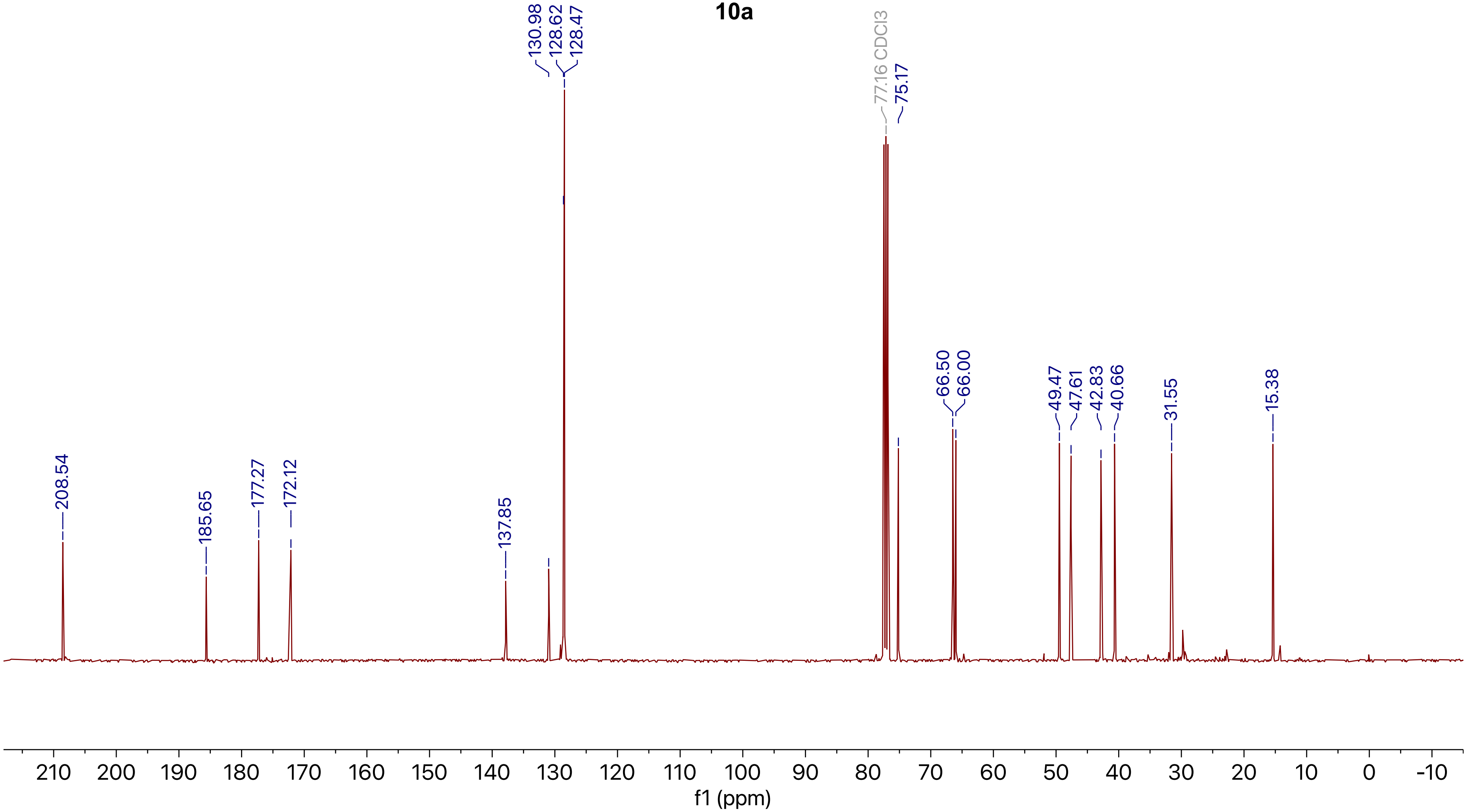

2D ^{1}H–^{1}H COSY (400 MHz, $CDCl_3$)

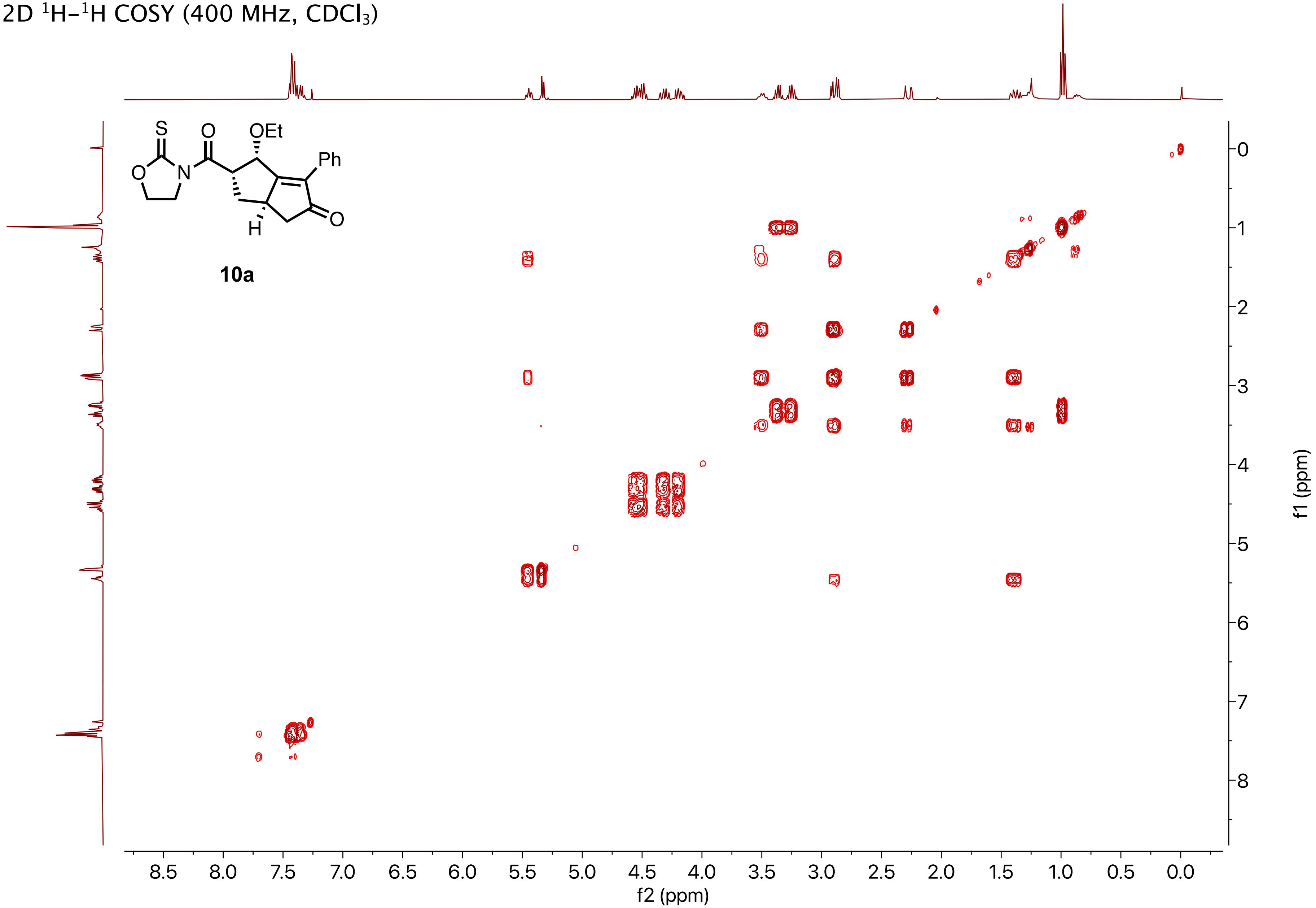

2D ^{1}H–^{13}C HSQC (400 MHz, $CDCl_3$)

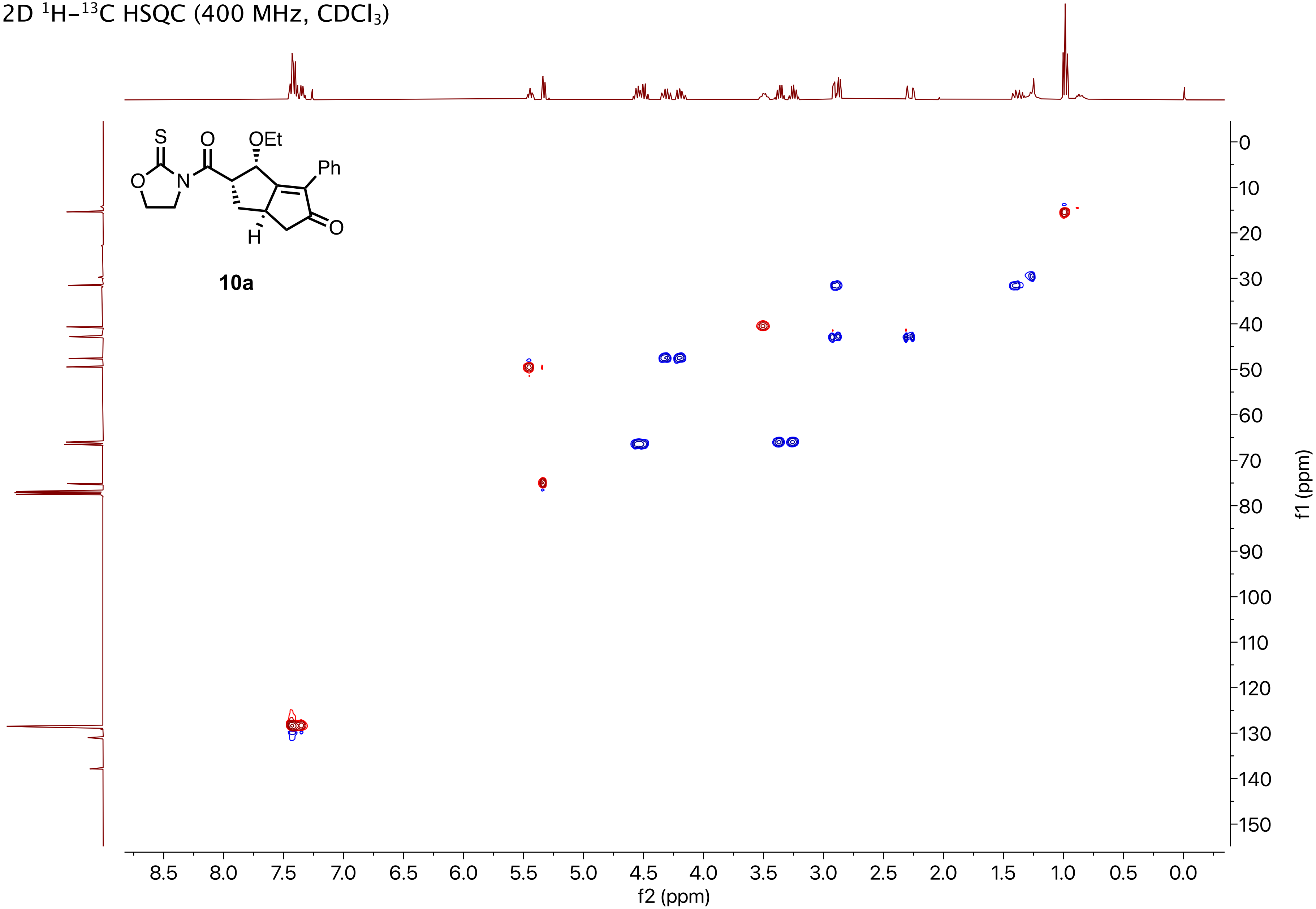

^{1}H NMR (400 MHz, $CDCl_3$)

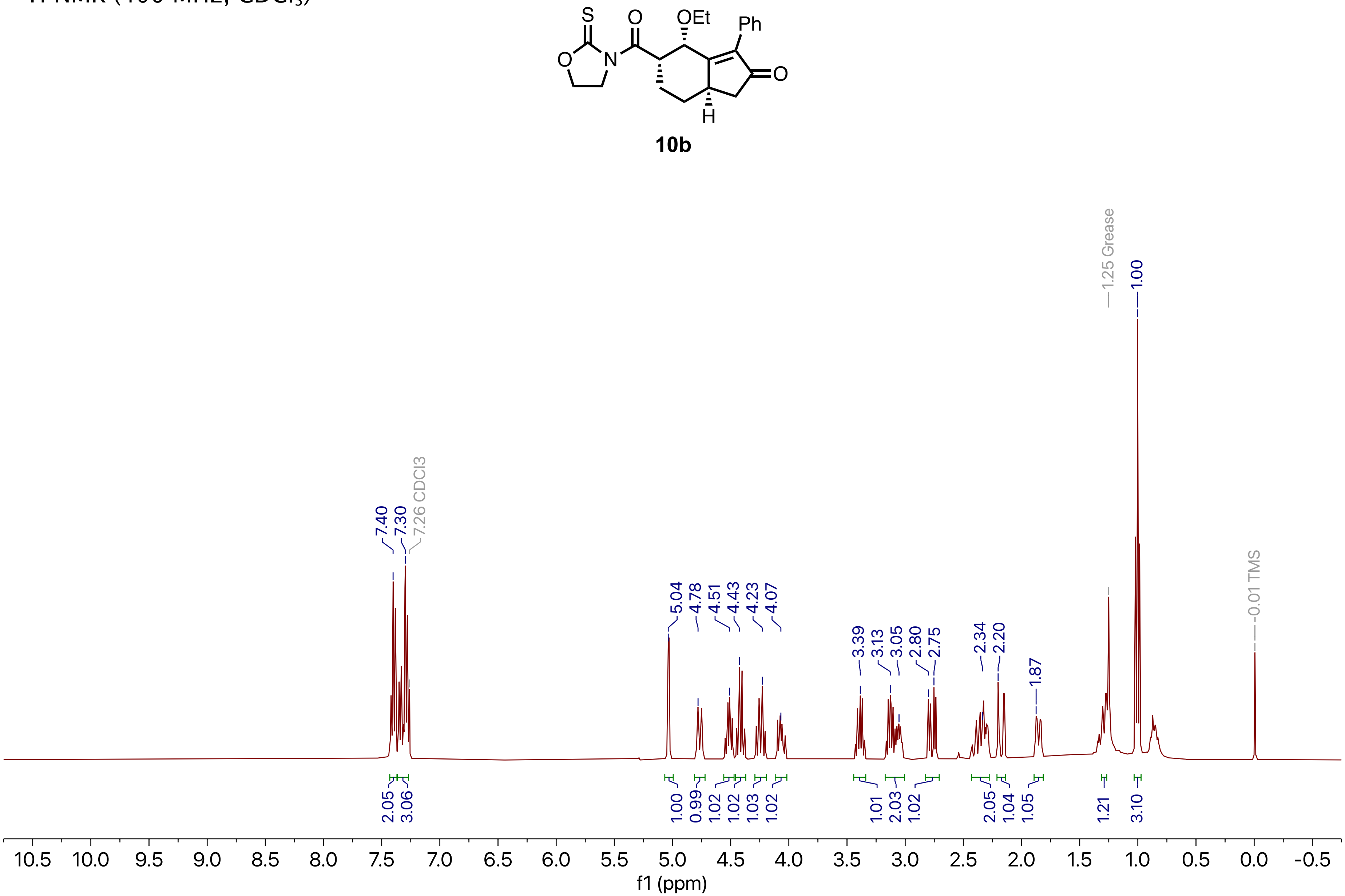

$^{13}C\{^{1}H\}$ NMR (101 MHz, $CDCl_3$)

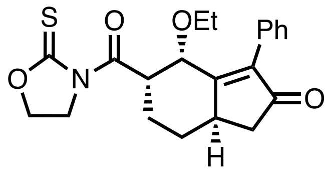

10b

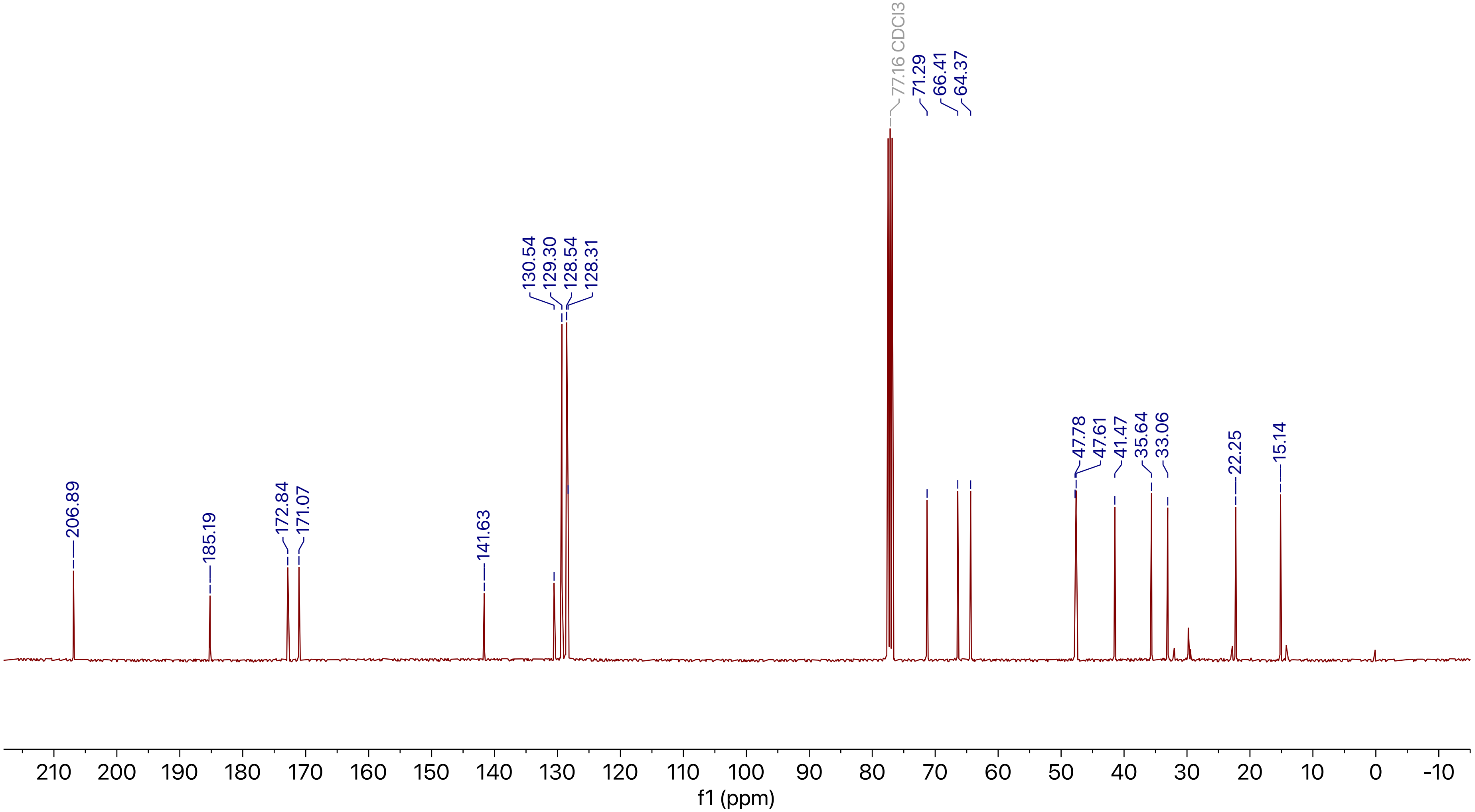

2D ^{1}H–^{1}H COSY (400 MHz, $CDCl_3$)

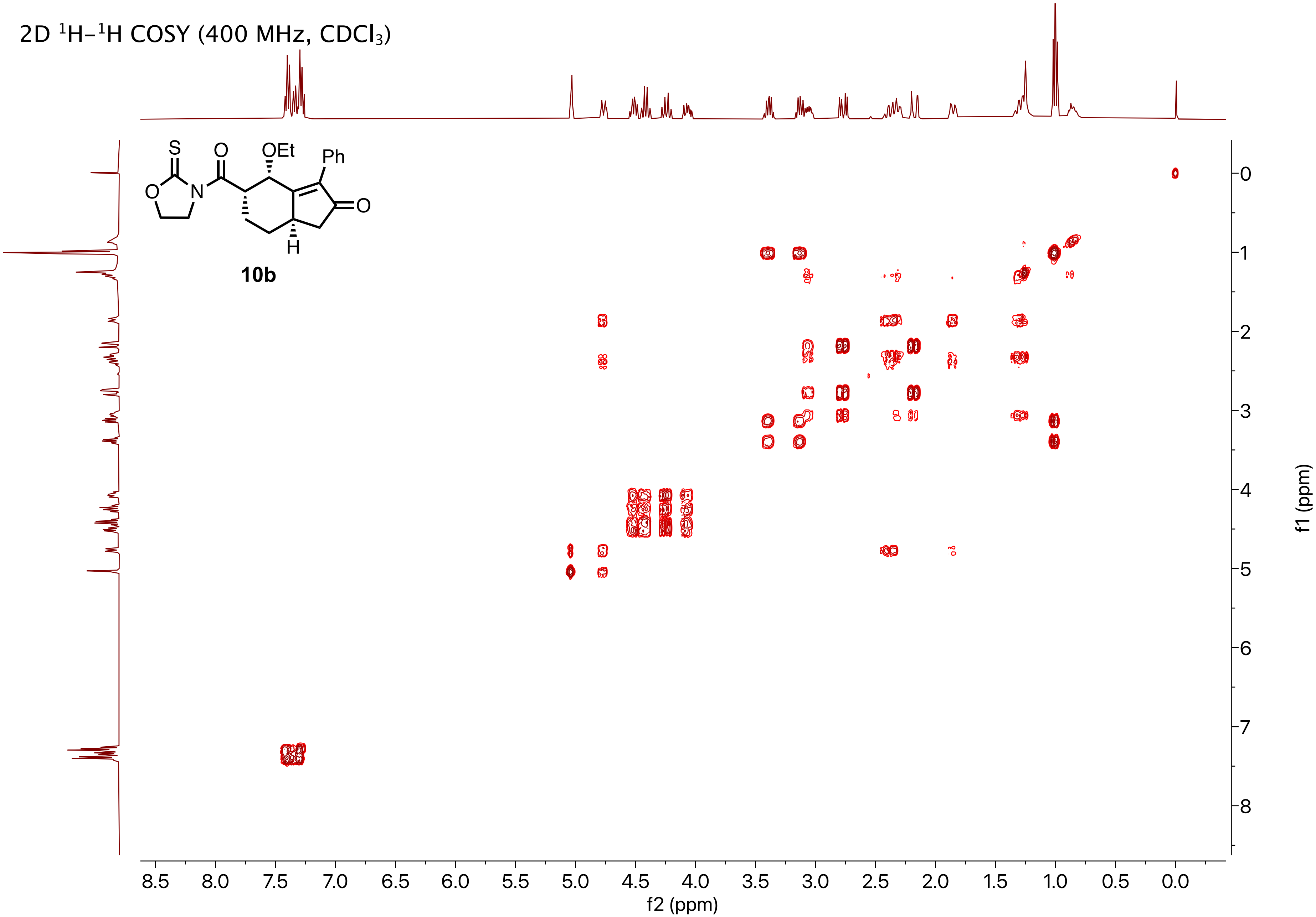

2D ^{1}H-^{13}C HSQC (400 MHz, $CDCl_3$)

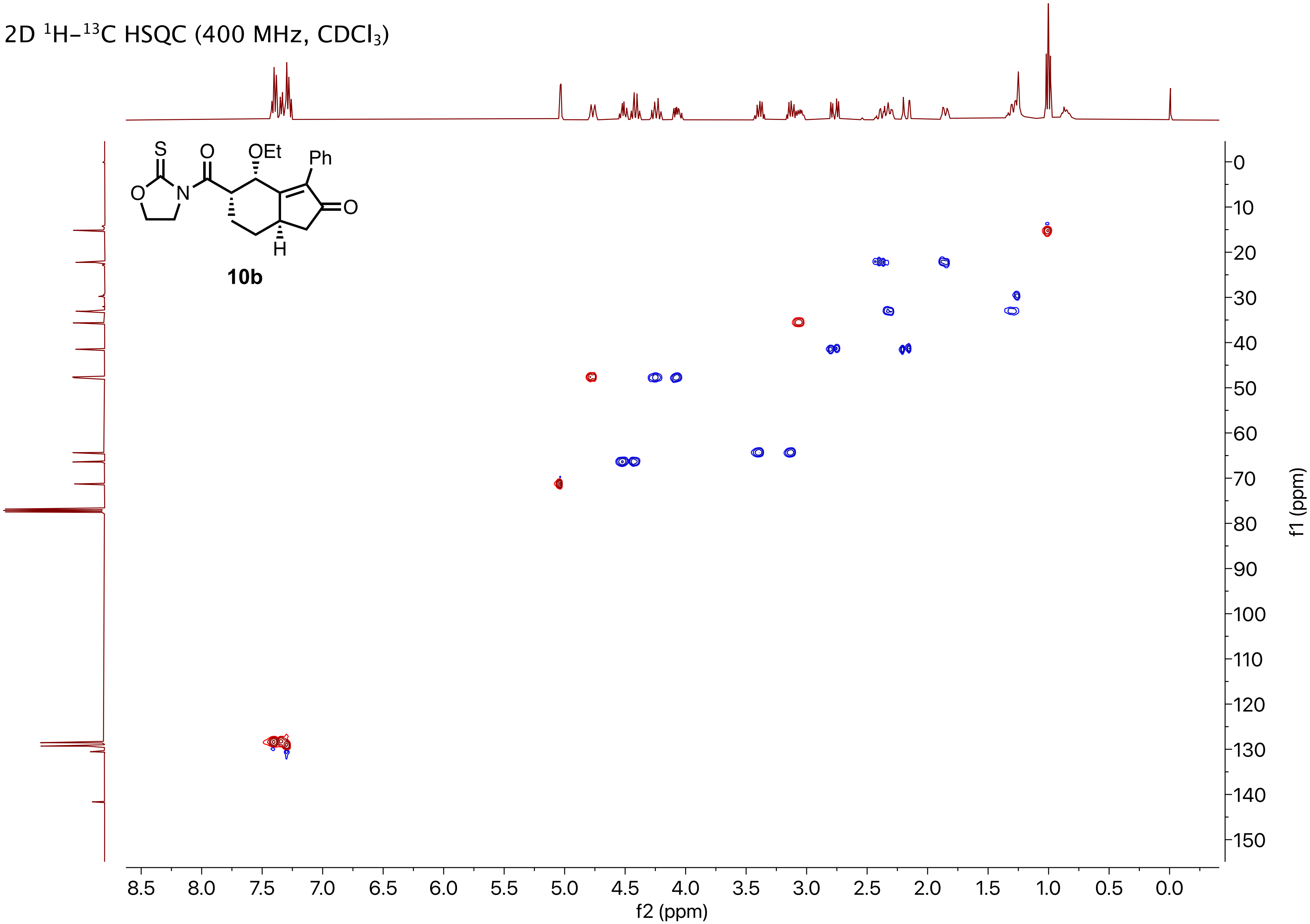

^{1}H NMR (400 MHz, $CDCl_3$)

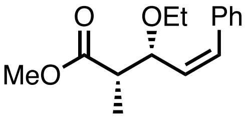

9a

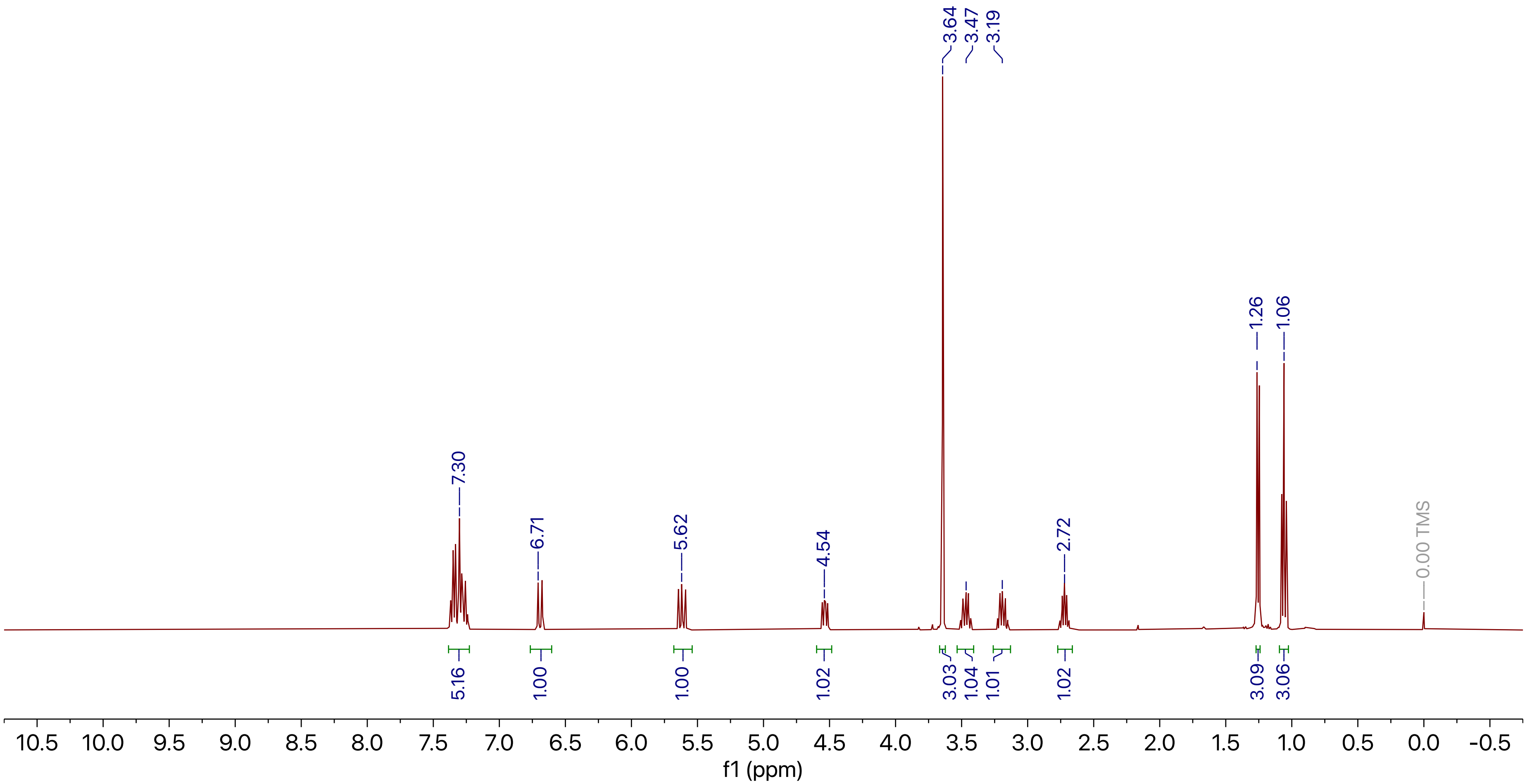

$^{13}C\{^{1}H\}$ NMR (101 MHz, $CDCl_3$)

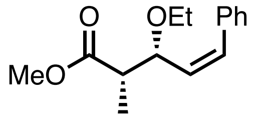

9a

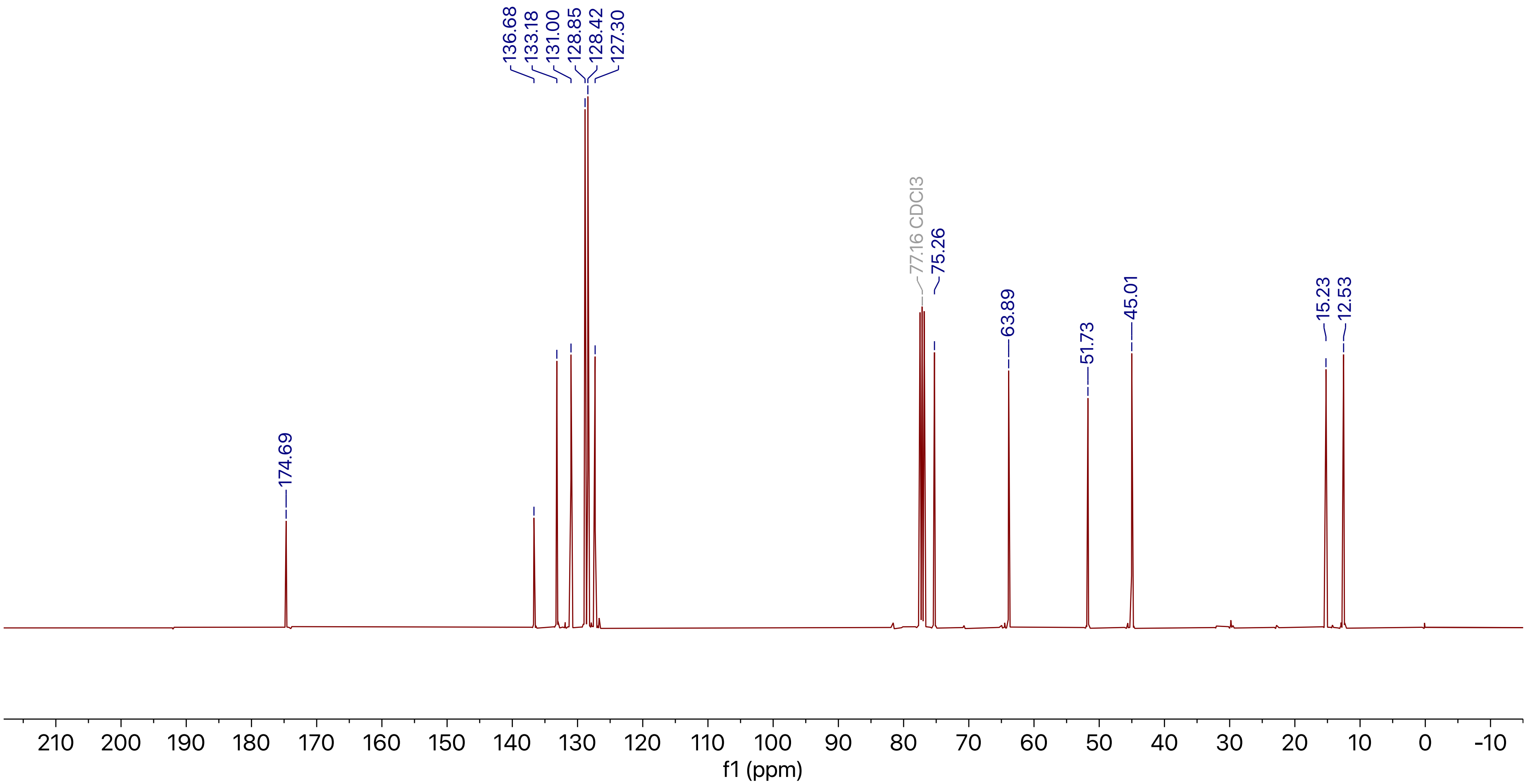

2D ^{1}H–^{1}H COSY (400 MHz, $CDCl_3$)

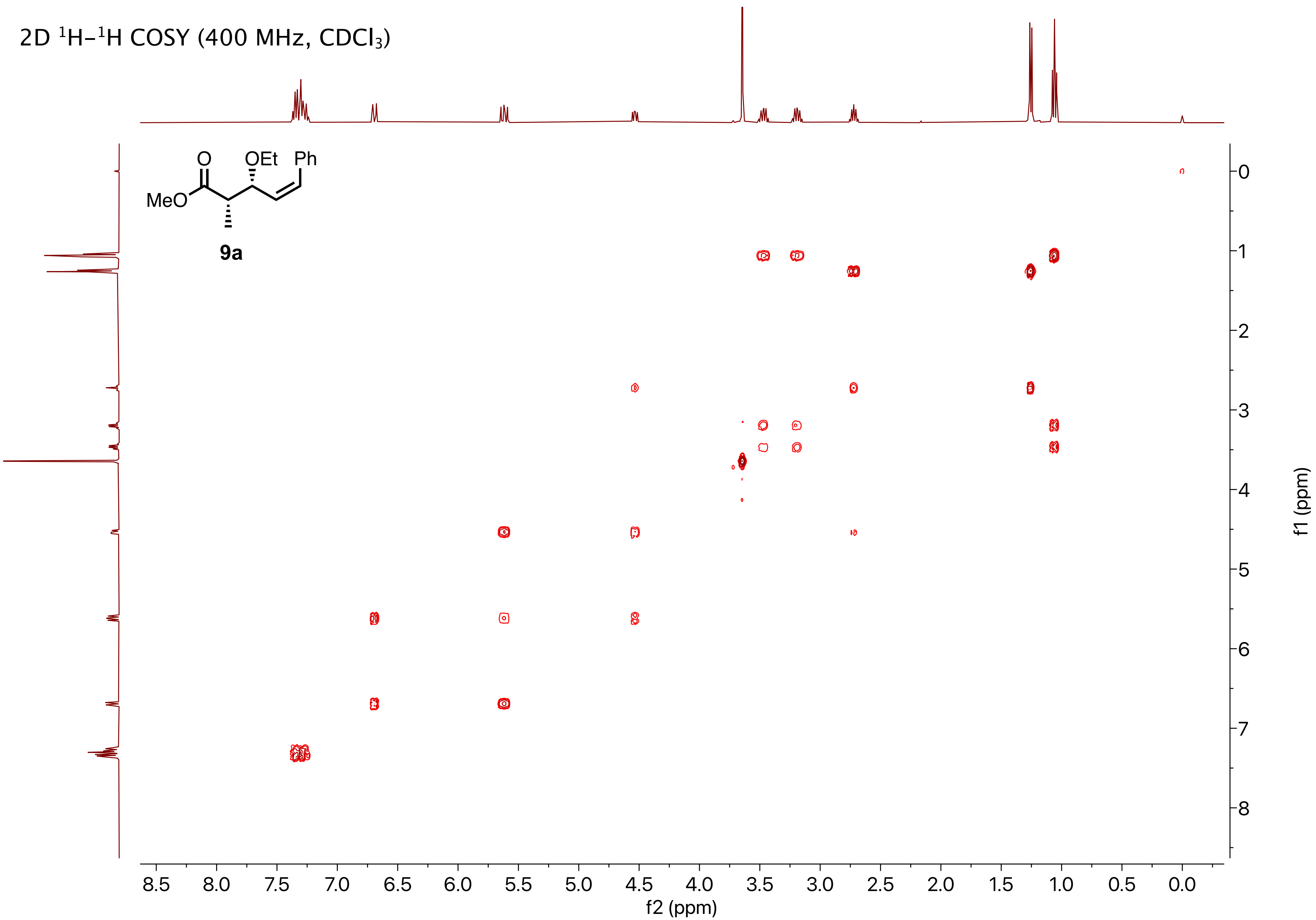

2D ^{1}H–^{13}C HSQC (400 MHz, $CDCl_3$)

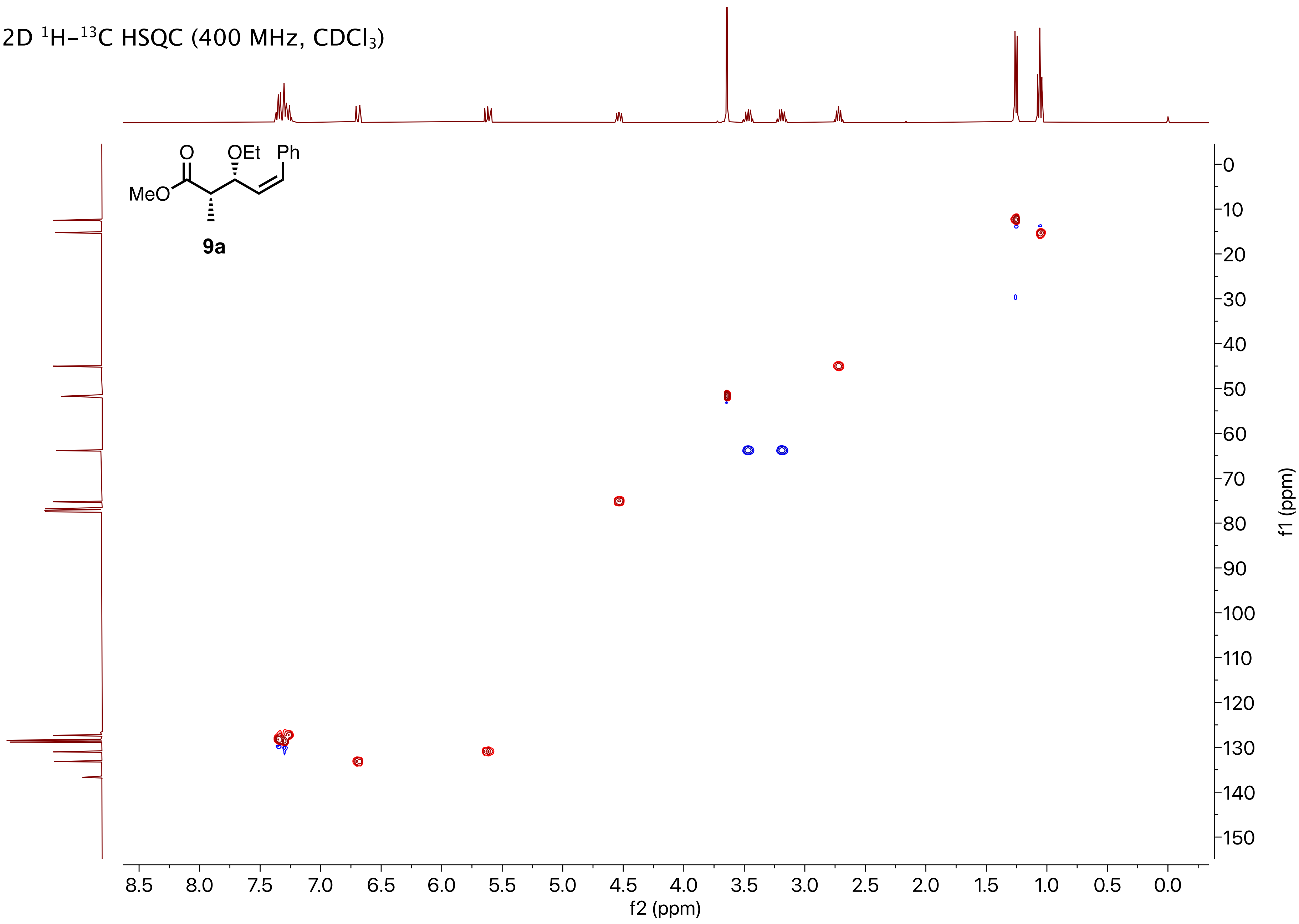

^{1}H NMR (400 MHz, CDCl$_3$)

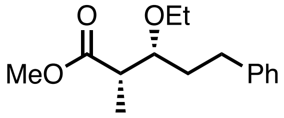

8a

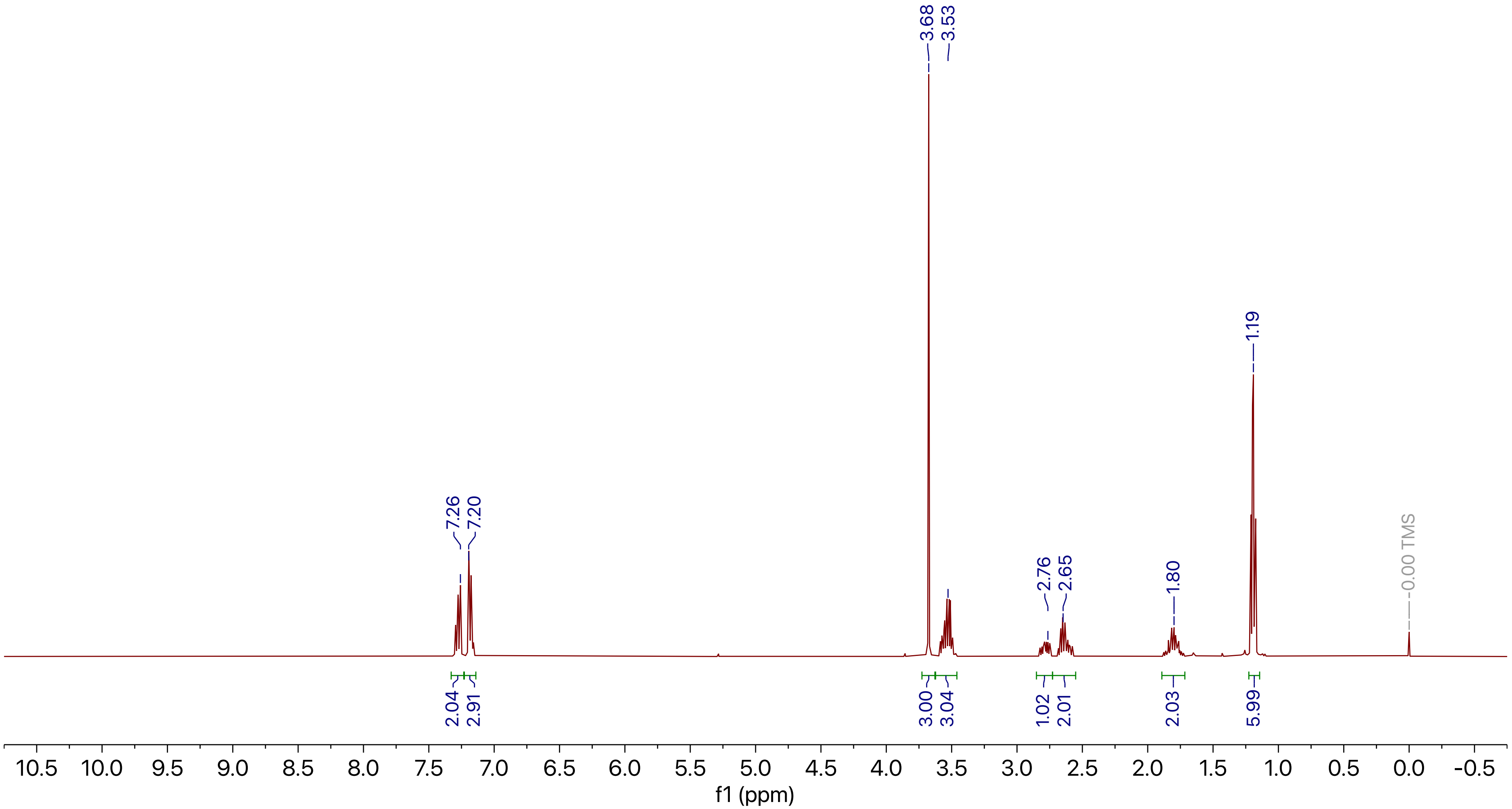

$^{13}C\{^{1}H\}$ NMR (101 MHz, $CDCl_3$)

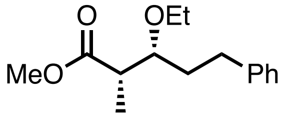

8a

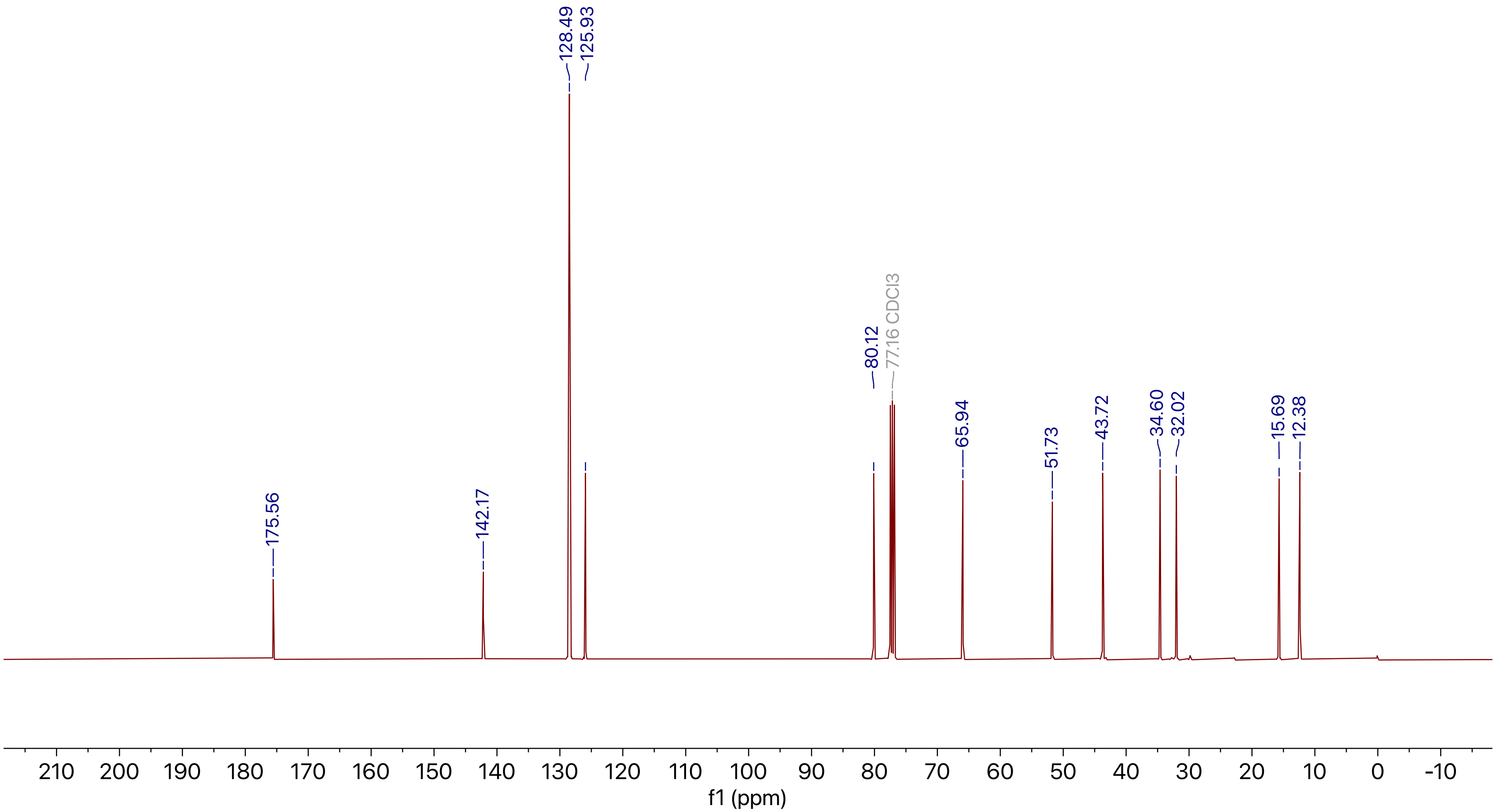

2D ^1H–^1H COSY (400 MHz, $CDCl_3$)

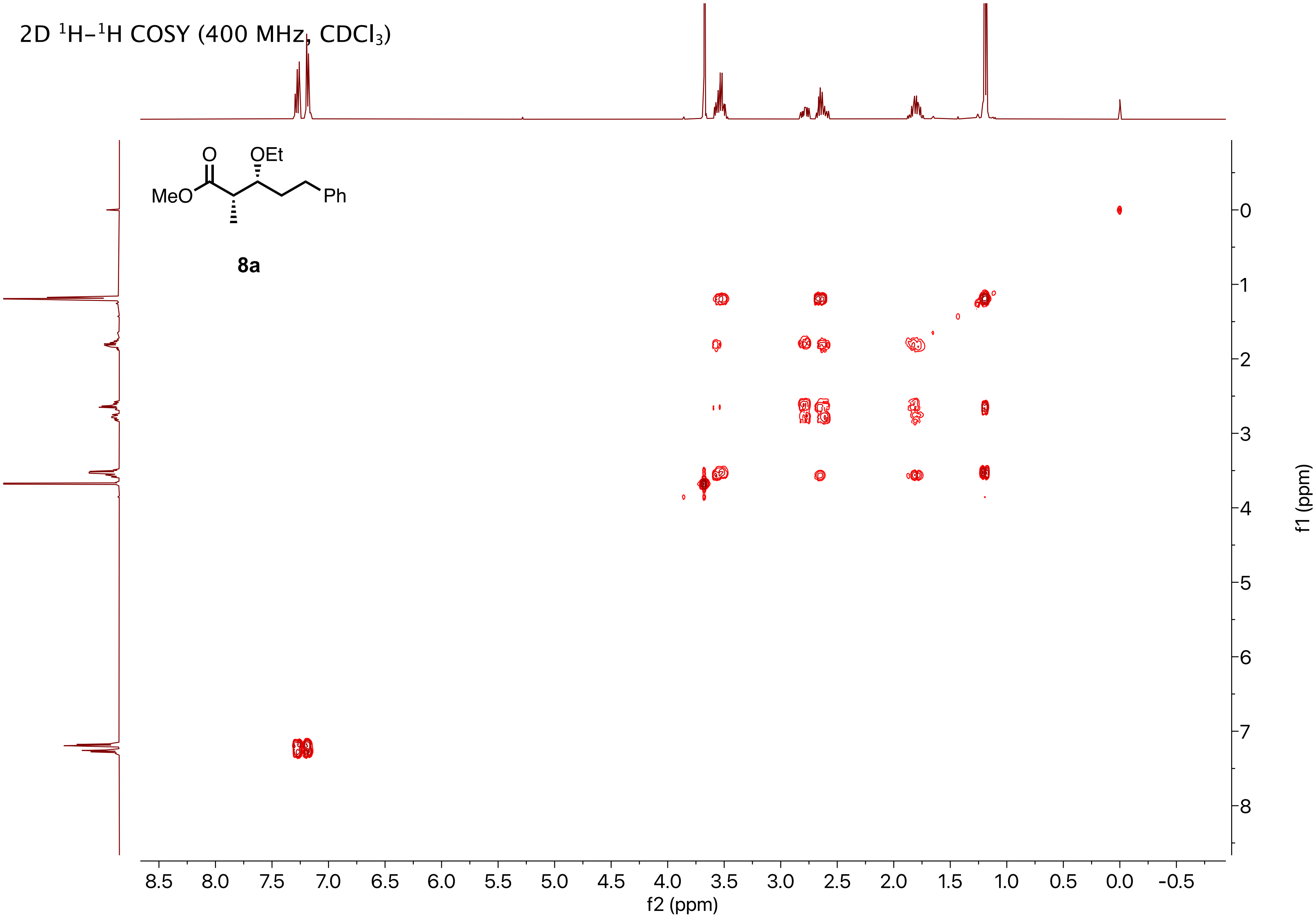

2D ^{1}H–^{13}C HSQC (400 MHz, $CDCl_3$)

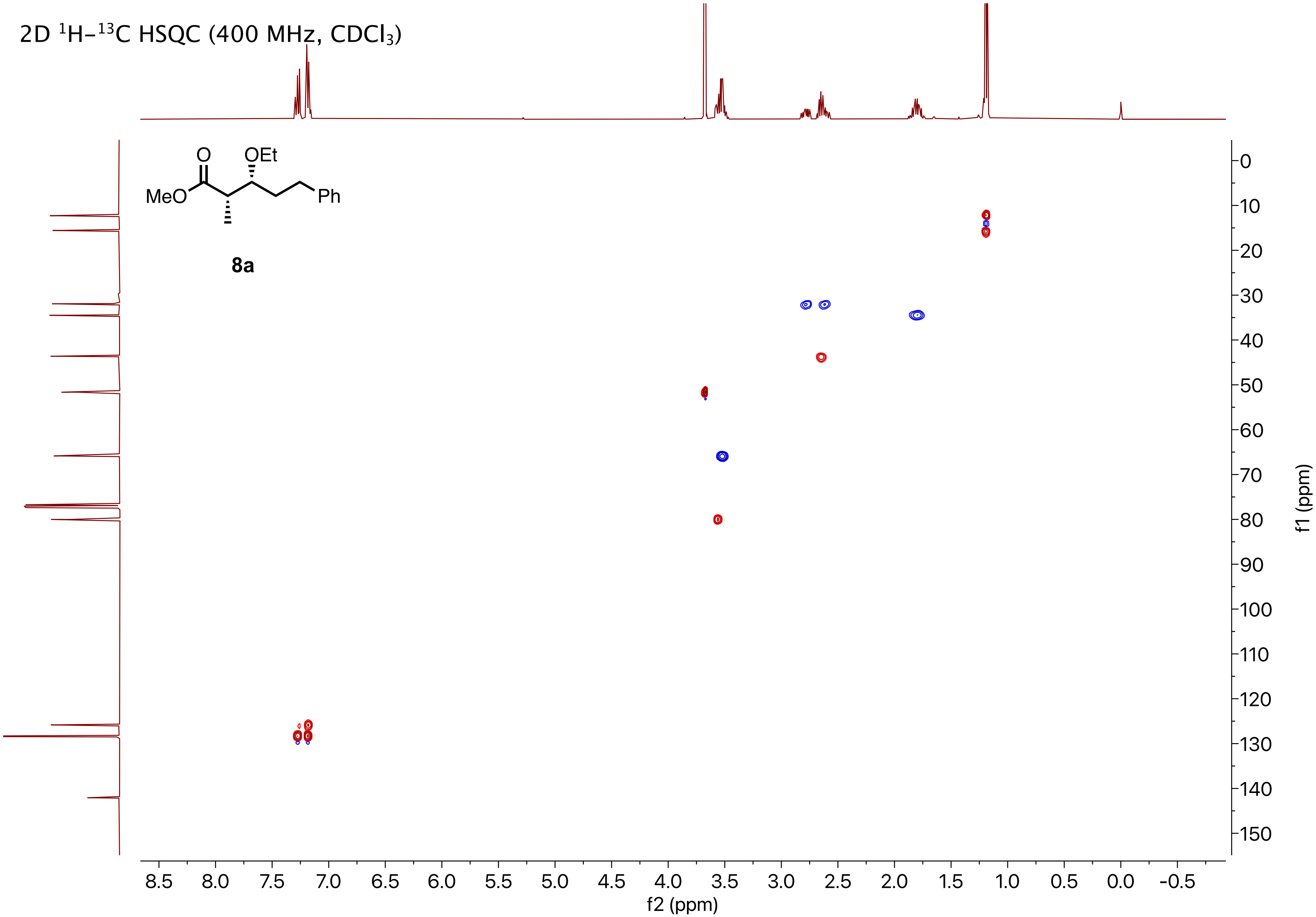

^{1}H NMR (400 MHz, $CDCl_3$)

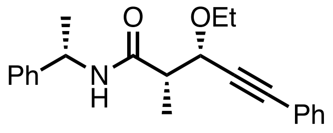

7a

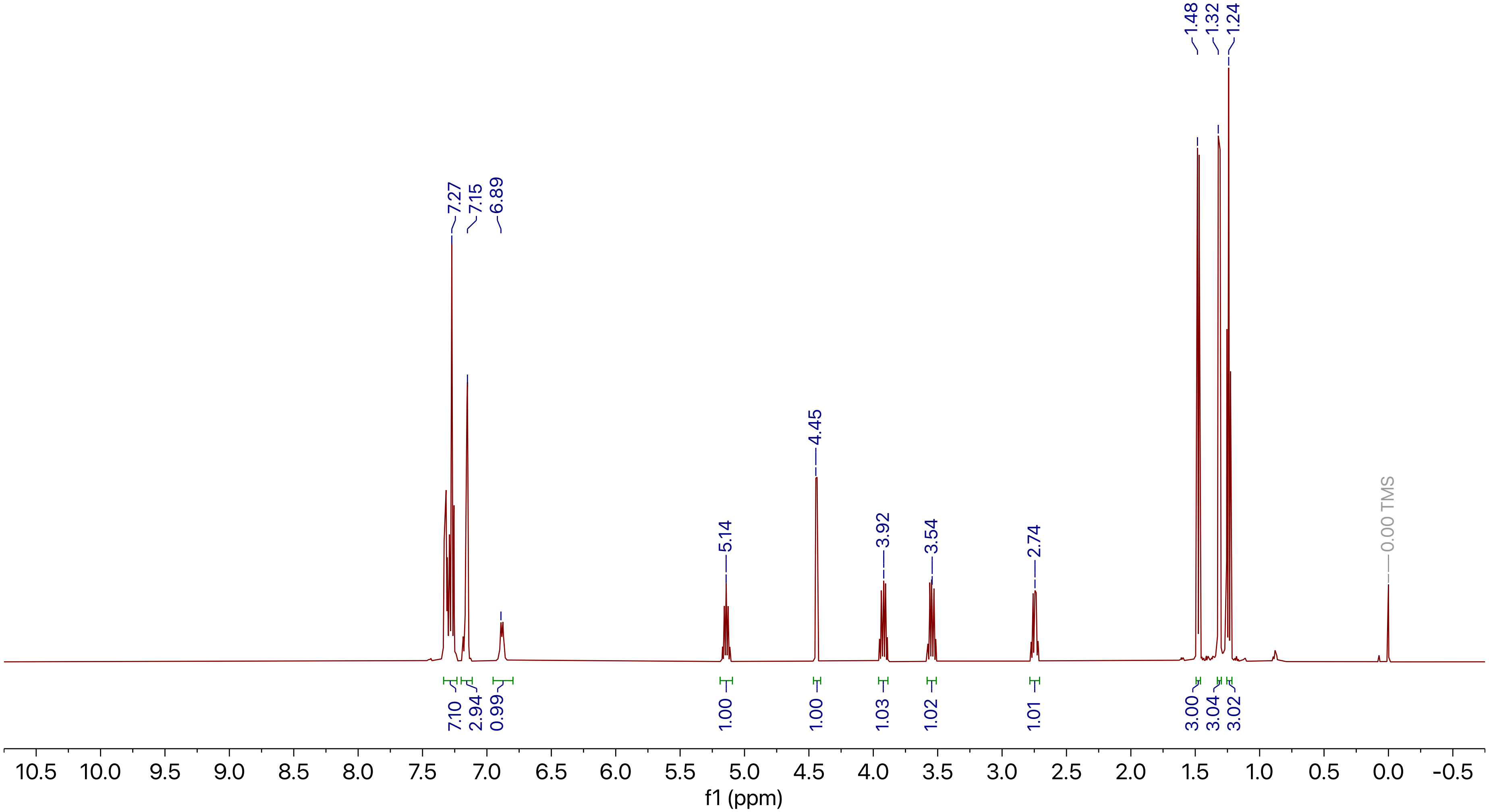

$^{13}C\{^{1}H\}$ NMR (101 MHz, $CDCl_3$)

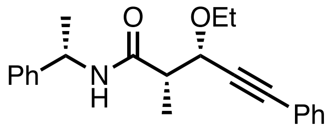

7a

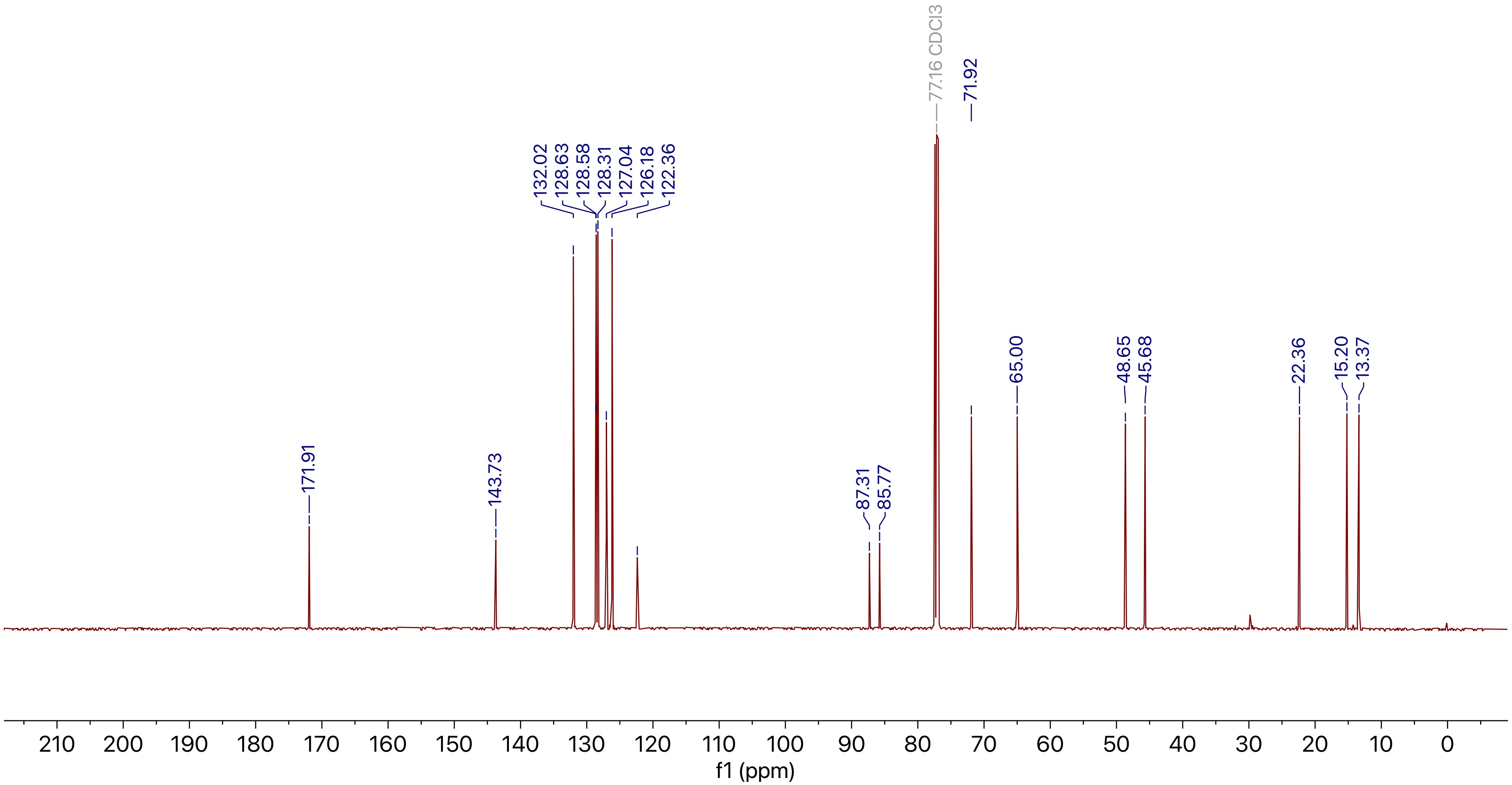

2D ^{1}H–^{1}H COSY (400 MHz, $CDCl_3$)

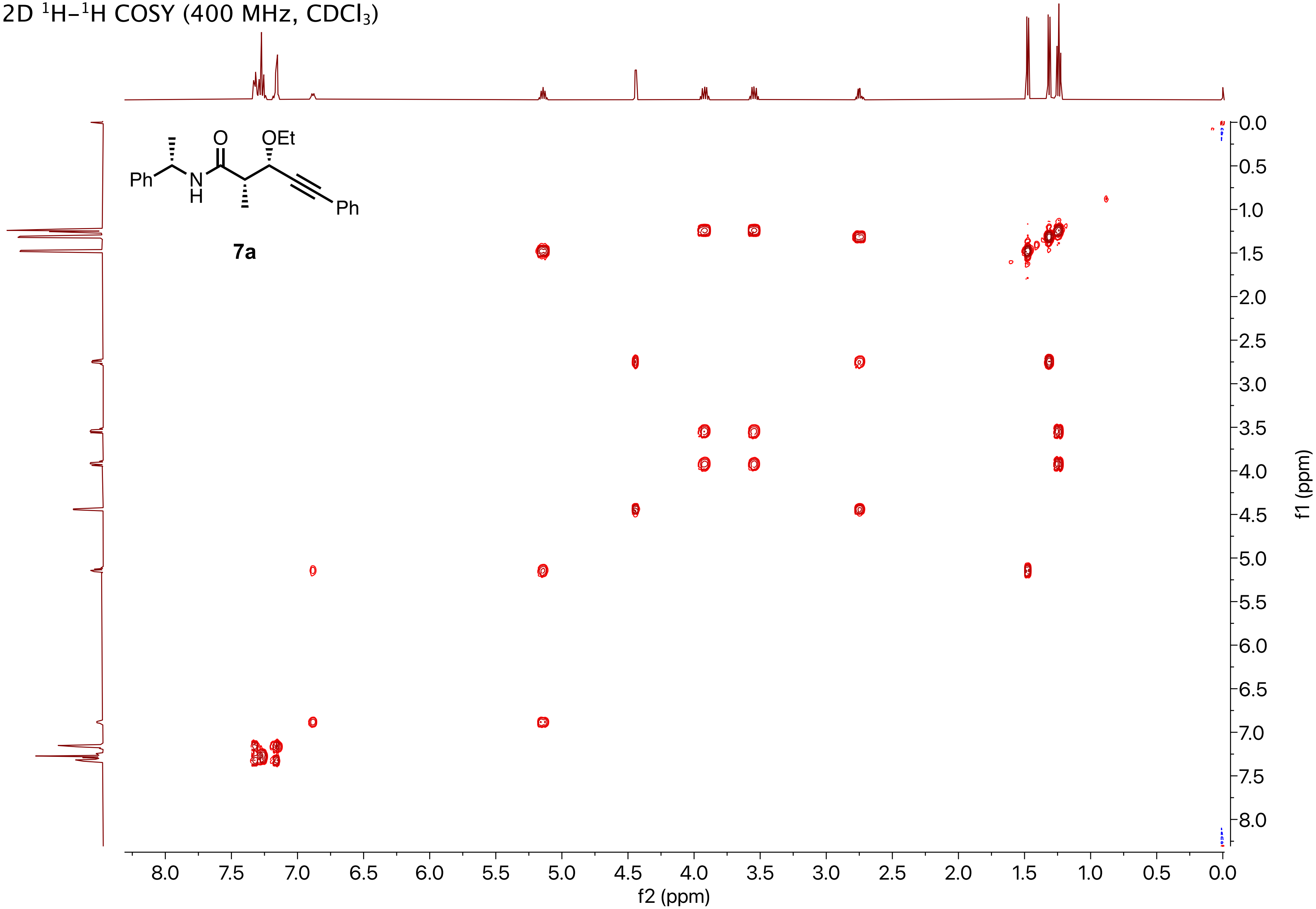

2D ^{1}H–^{13}C HSQC (400 MHz, $CDCl_3$)

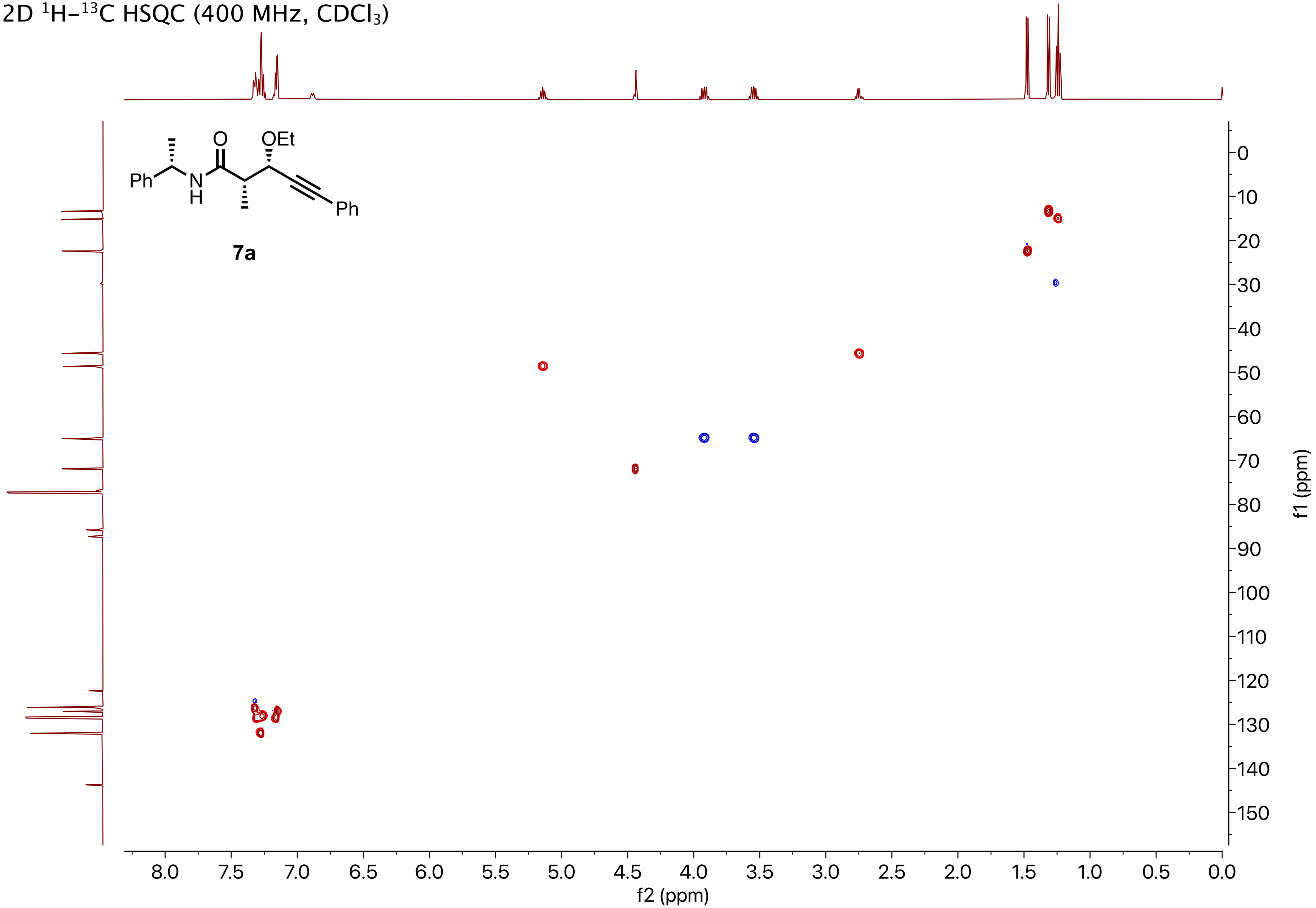

^{1}H NMR (400 MHz, $CDCl_3$)

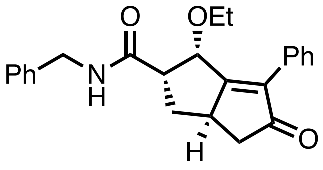

11a

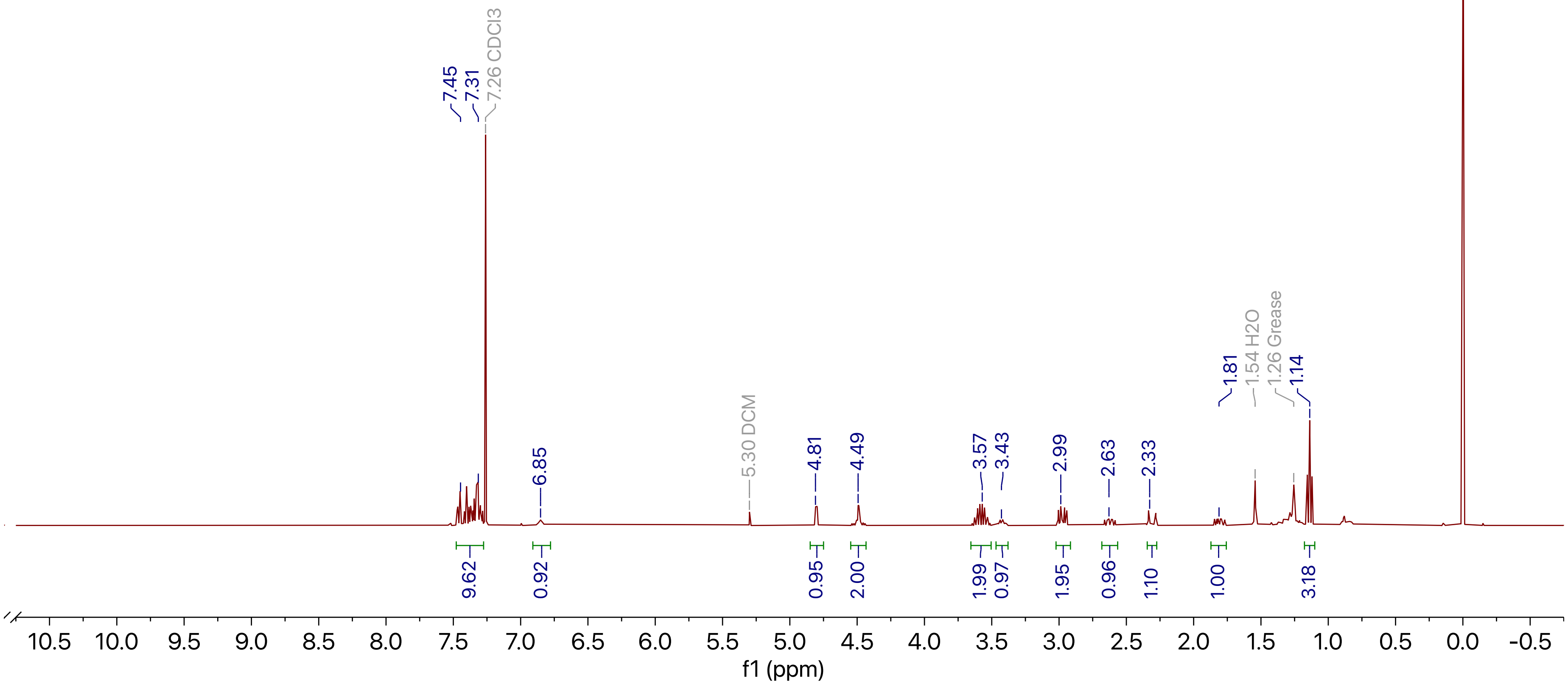

$^{13}C\{^{1}H\}$ NMR (101 MHz, $CDCl_3$)

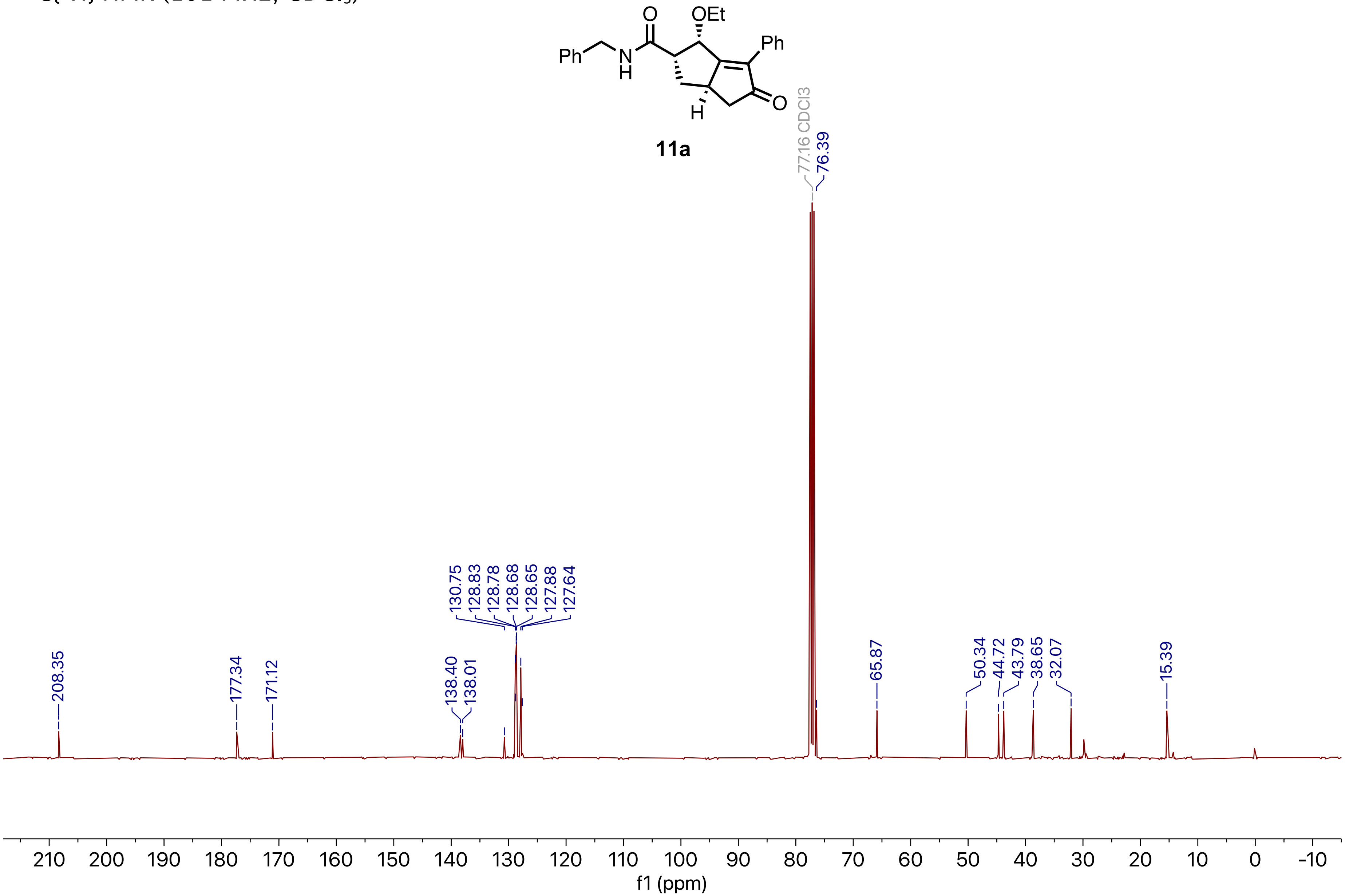

2D ^{1}H–^{1}H COSY (400 MHz, $CDCl_3$)

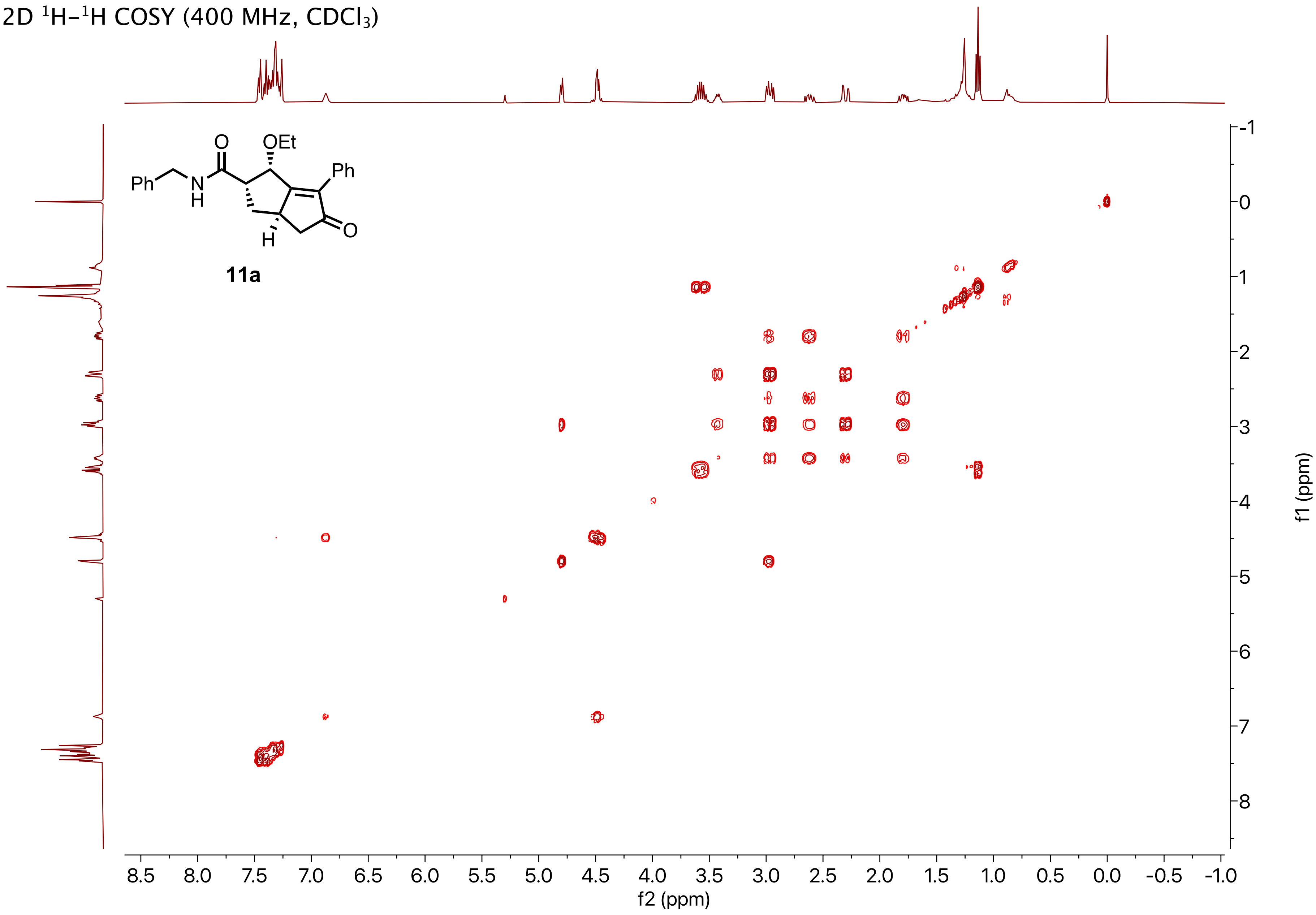

2D ^{1}H–^{13}C HSQC (400 MHz, $CDCl_3$)

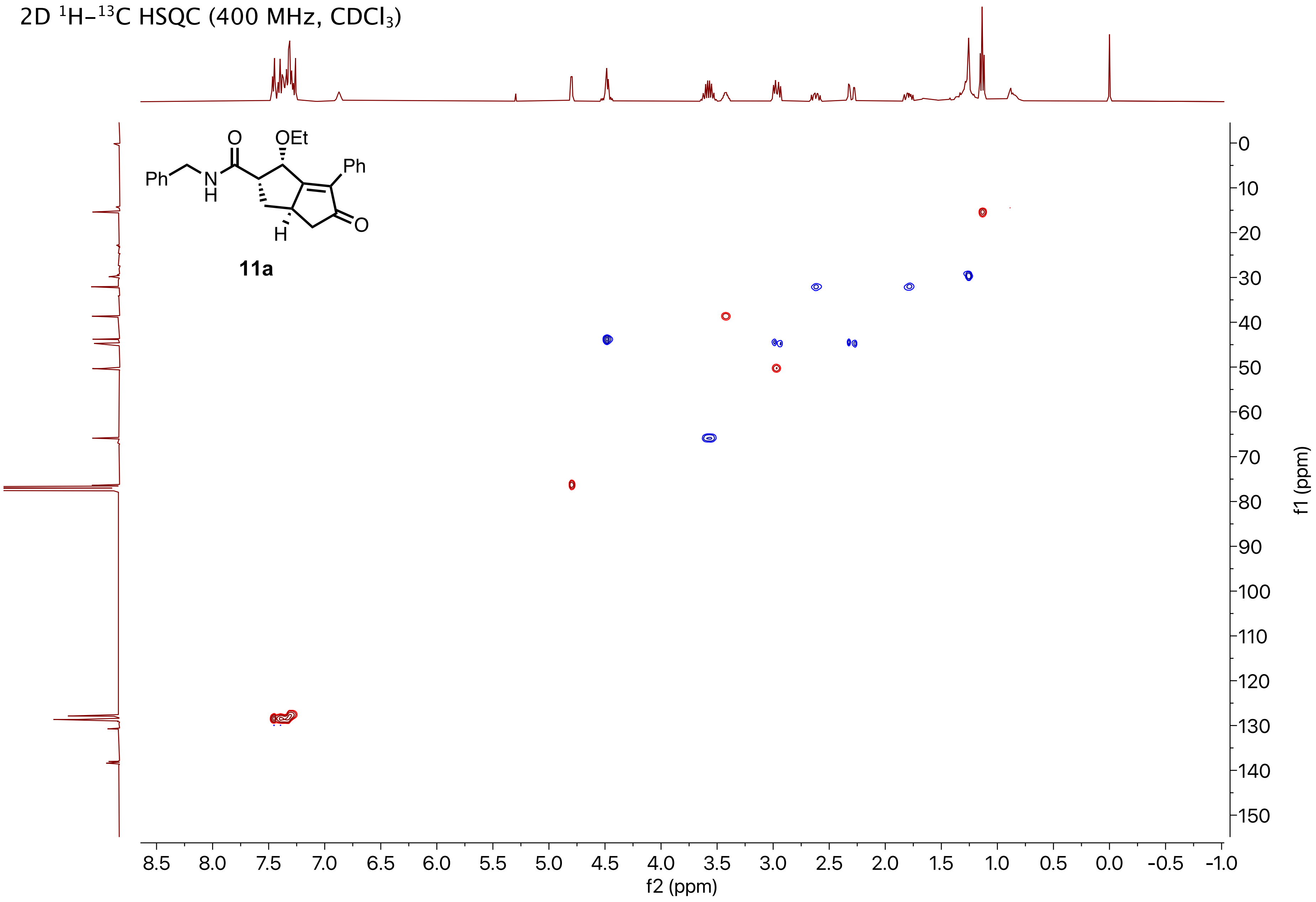

12. HPLC/GC Chromatograms

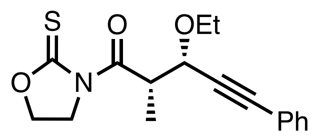

4a

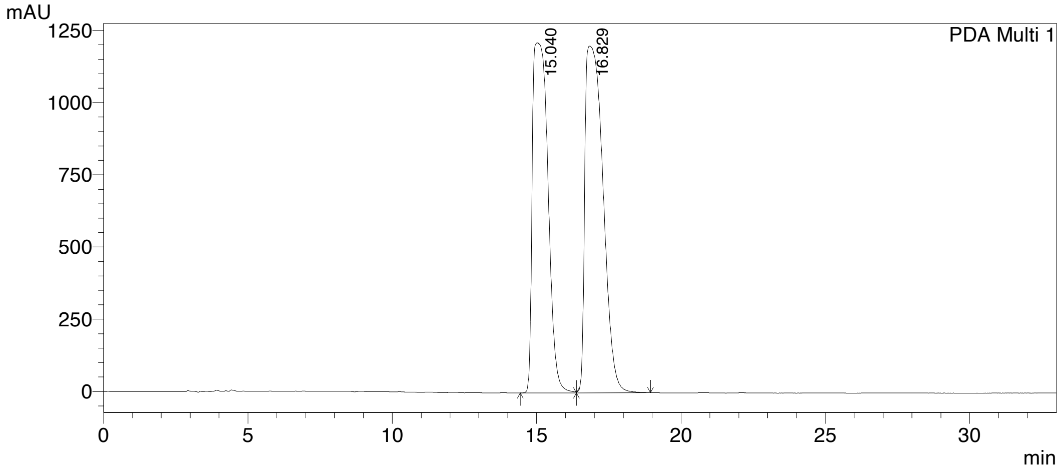

1 PDA Multi 1/254nm 4nm

PeakTable

PDA Ch1 254nm 4nm

Peak#	Ret. Time	Area	Height	Area %	Height %
1	15.040	45655994	1211858	46.596	50.230
2	16.829	52327122	1200783	53.404	49.770
Total		97983116	2412641	100.000	100.000

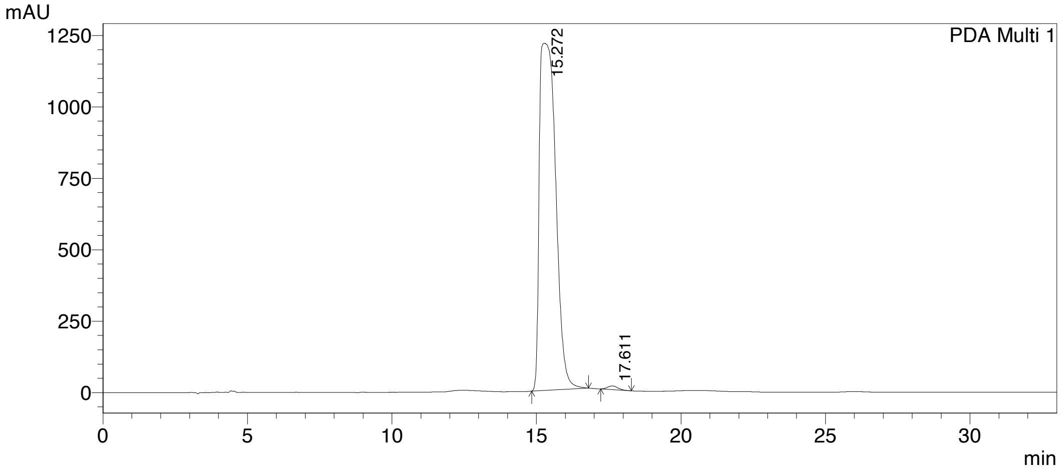

1 PDA Multi 1/254nm 4nm

PeakTable

PDA Ch1 254nm 4nm

Peak#	Ret. Time	Area	Height	Area %	Height %
1	15.272	47847645	1214901	99.344	98.966
2	17.611	315947	12691	0.656	1.034
Total		48163592	1227592	100.000	100.000

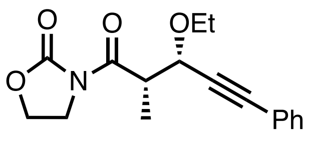

5a

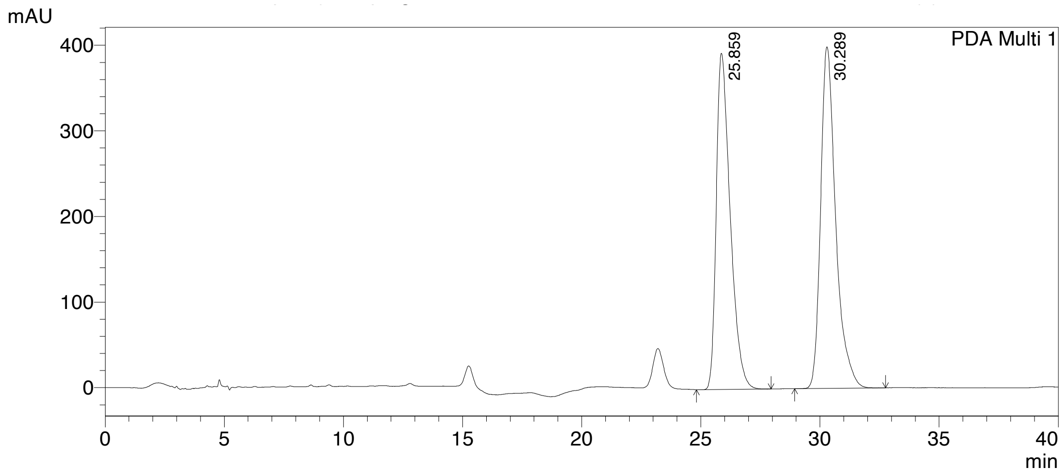

1 PDA Multi 1/254nm 4nm

PeakTable

PDA Ch1 254nm 4nm

Peak#	Ret. Time	Area	Height	Area %	Height %
1	25.859	15965573	392831	47.666	49.610
2	30.289	17528927	399008	52.334	50.390
Total		33494500	791839	100.000	100.000

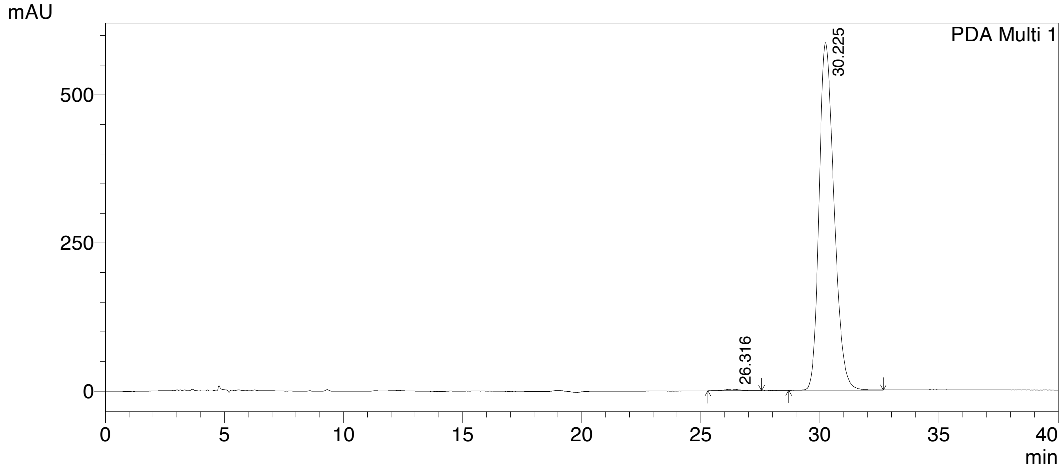

1 PDA Multi 1/254nm 4nm

PeakTable

PDA Ch1 254nm 4nm

Peak#	Ret. Time	Area	Height	Area %	Height %
1	26.316	106129	2701	0.412	0.458
2	30.225	25623854	586580	99.588	99.542
Total		25729983	589282	100.000	100.000

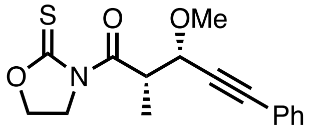

4b

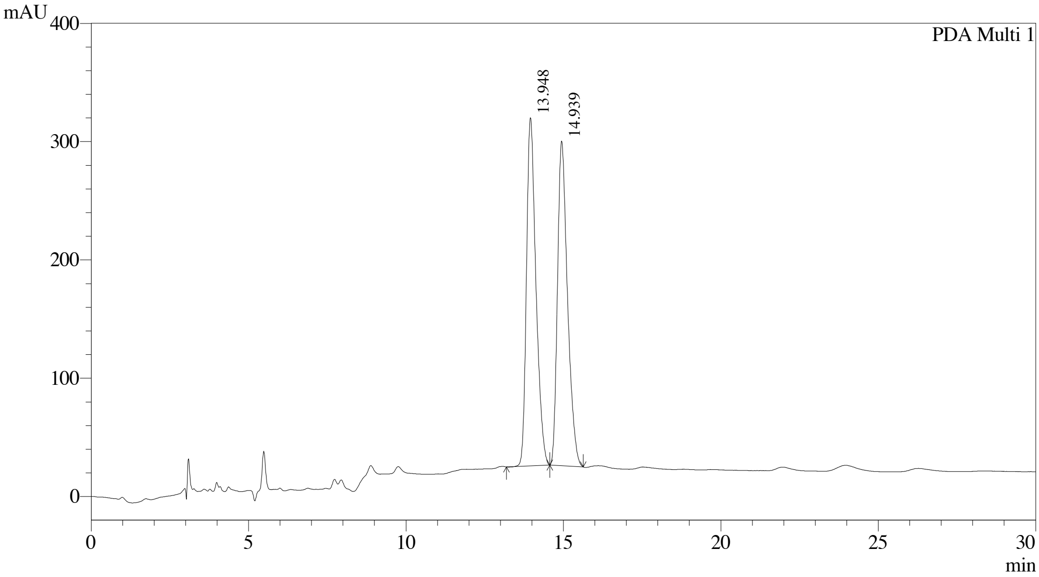

1 PDA Multi 1 / 271nm 4nm

PeakTable

PDA Ch1 271nm 4nm

Peak#	Ret. Time	Area	Height	Area %	Height %
1	13.948	6156245	294415	50.339	51.757
2	14.939	6073280	274423	49.661	48.243
Total		12229525	568838	100.000	100.000

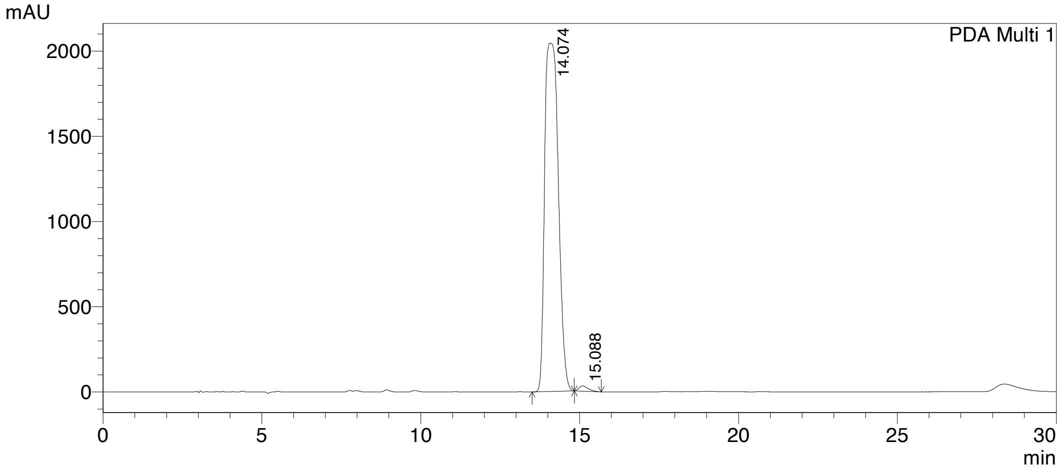

PeakTable

PDA Ch1 271nm 4nm

Peak#	Ret. Time	Area	Height	Area %	Height %
1	14.074	61522501	2044668	98.998	98.509
2	15.088	622820	30944	1.002	1.491
Total		62145320	2075612	100.000	100.000

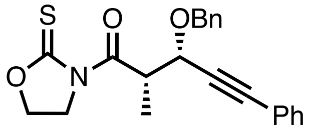

4c

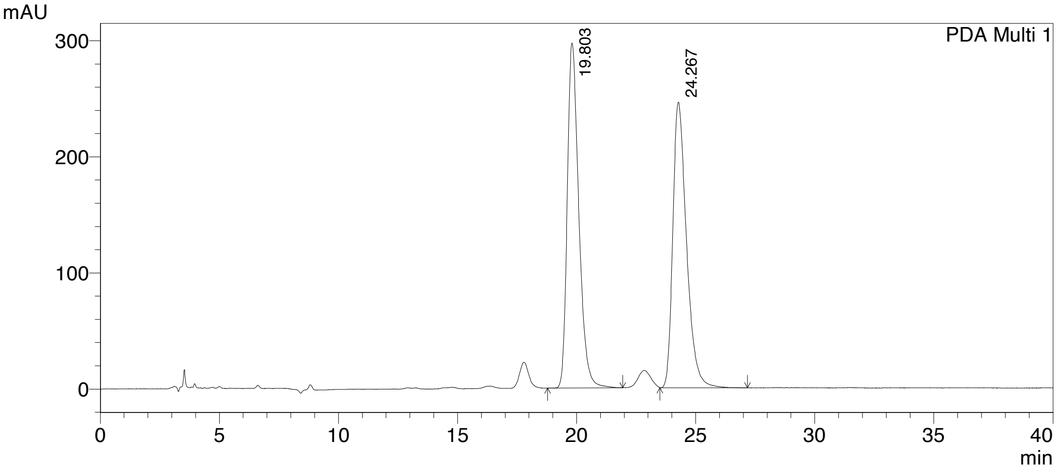

PeakTable

PDA Ch1 254nm 4nm

Peak#	Ret. Time	Area	Height	Area %	Height %
1	19.803	10020816	297246	50.448	54.701
2	24.267	9842897	246160	49.552	45.299
Total		19863713	543406	100.000	100.000

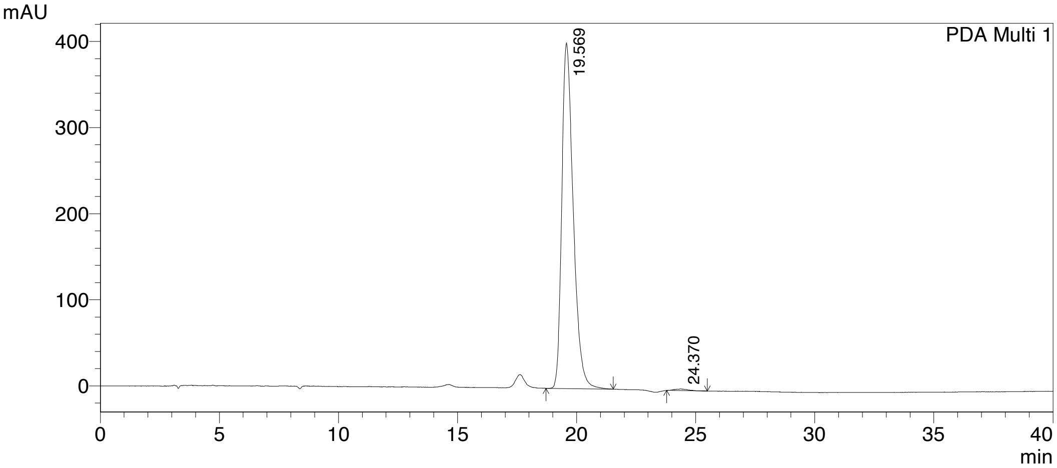

PeakTable

PDA Ch1 254nm 4nm

Peak#	Ret. Time	Area	Height	Area %	Height %
1	19.569	13451913	401800	99.428	99.521
2	24.370	77419	1934	0.572	0.479
Total		13529332	403735	100.000	100.000

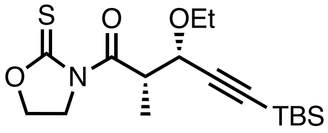

4d

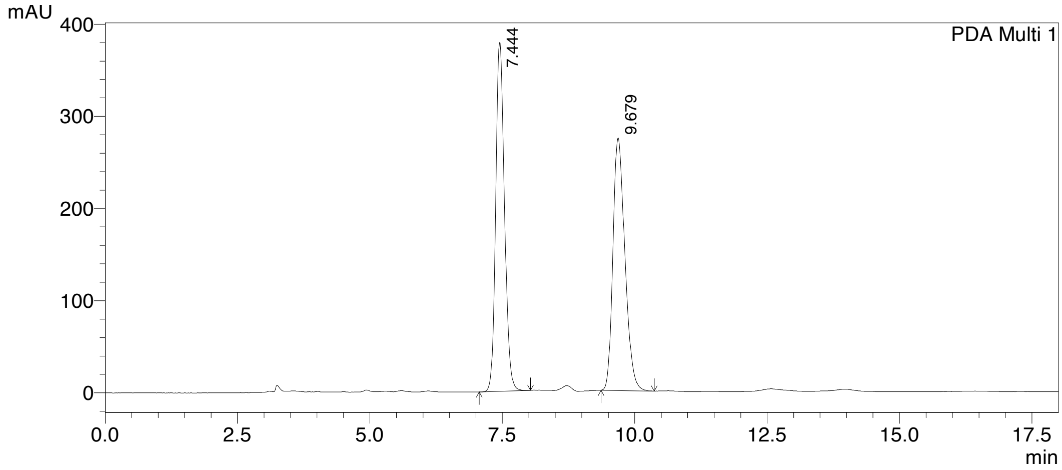

1 PDA Multi 1/254nm 4nm

PeakTable

PDA Ch1 254nm 4nm

Peak#	Ret. Time	Area	Height	Area %	Height %
1	7.444	4374003	378576	50.242	57.974
2	9.679	4331890	274430	49.758	42.026
Total		8705893	653005	100.000	100.000

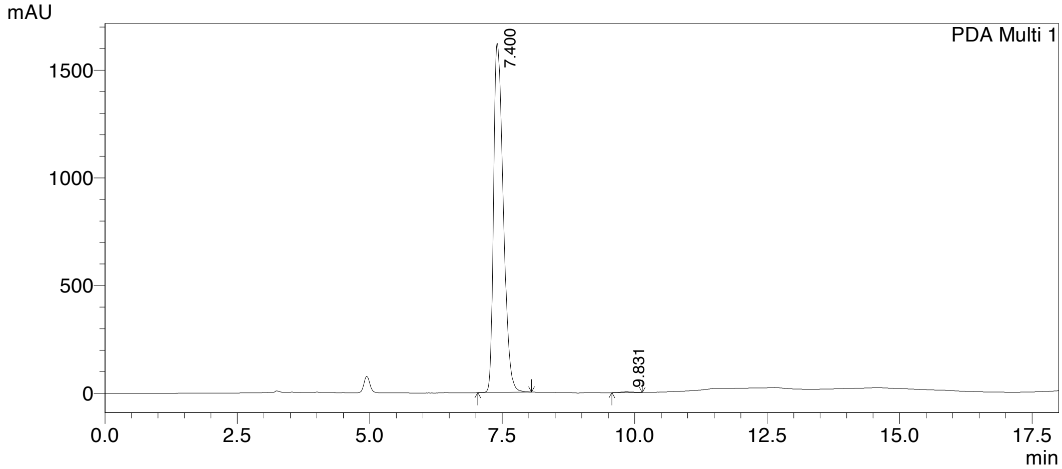

1 PDA Multi 1/254nm 4nm

PeakTable

PDA Ch1 254nm 4nm

Peak#	Ret. Time	Area	Height	Area %	Height %
1	7.400	20695839	1621456	99.719	99.791
2	9.831	58406	3400	0.281	0.209
Total		20754245	1624855	100.000	100.000

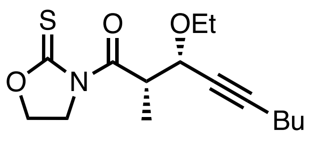

4e

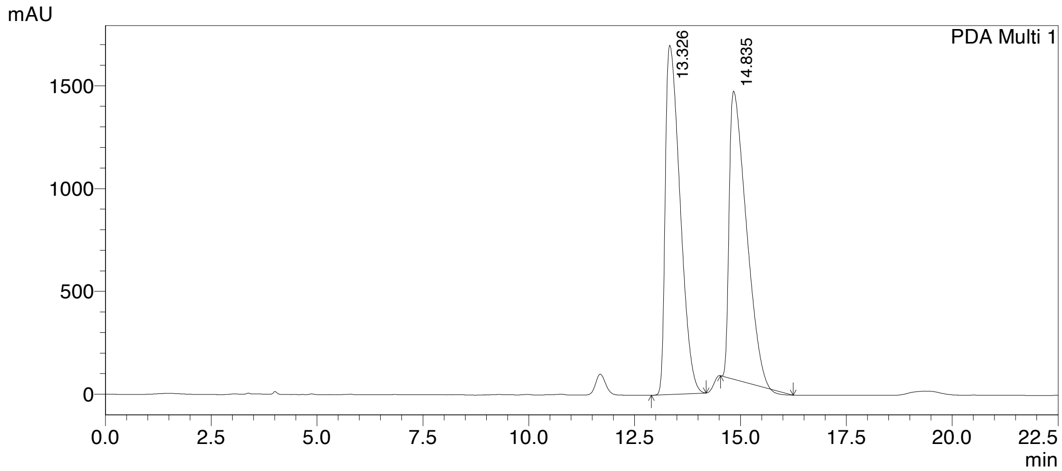

PeakTable

PDA Ch1 254nm 4nm

Peak#	Ret. Time	Area	Height	Area %	Height %
1	13.326	41316426	1698291	51.384	54.805
2	14.835	39090117	1400513	48.616	45.195
Total		80406543	3098804	100.000	100.000

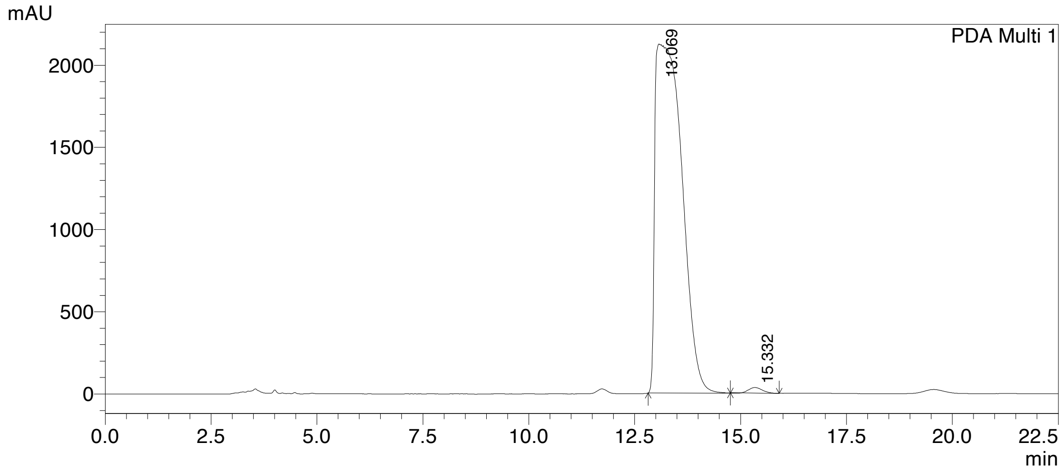

PeakTable

PDA Ch1 254nm 4nm

Peak#	Ret. Time	Area	Height	Area %	Height %
1	13.069	93806232	2122161	99.251	98.459
2	15.332	707461	33220	0.749	1.541
Total		94513692	2155381	100.000	100.000

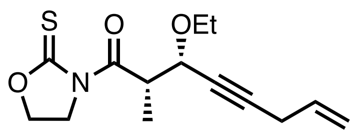

4f

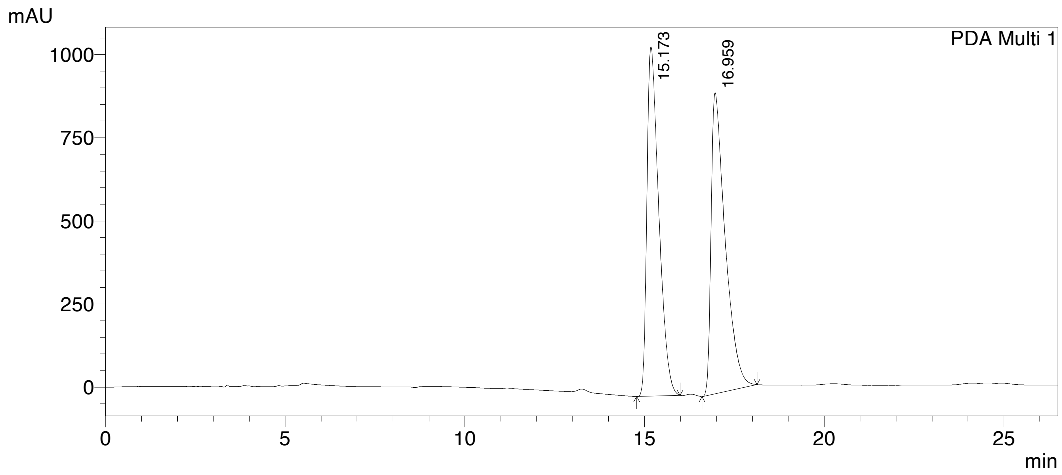

PeakTable

PDA Ch1 254nm 4nm

Peak#	Ret. Time	Area	Height	Area %	Height %
1	15.173	24160776	1050303	49.442	53.709
2	16.959	24706256	905225	50.558	46.291
Total		48867032	1955528	100.000	100.000

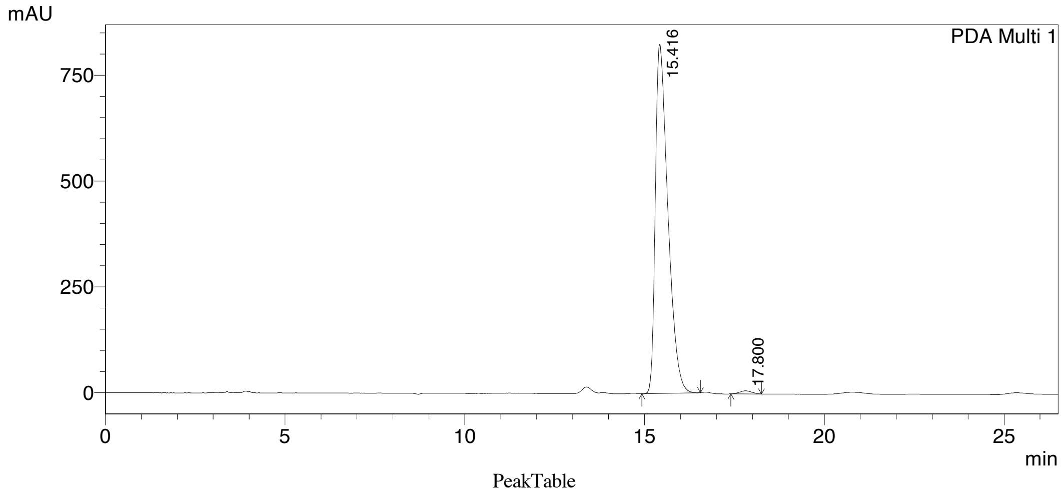

PeakTable

PDA Ch1 254nm 4nm

Peak#	Ret. Time	Area	Height	Area %	Height %
1	15.416	19978105	824323	99.152	99.125
2	17.800	170955	7280	0.848	0.875
Total		20149059	831603	100.000	100.000

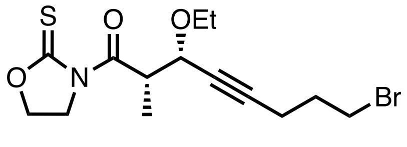

4g

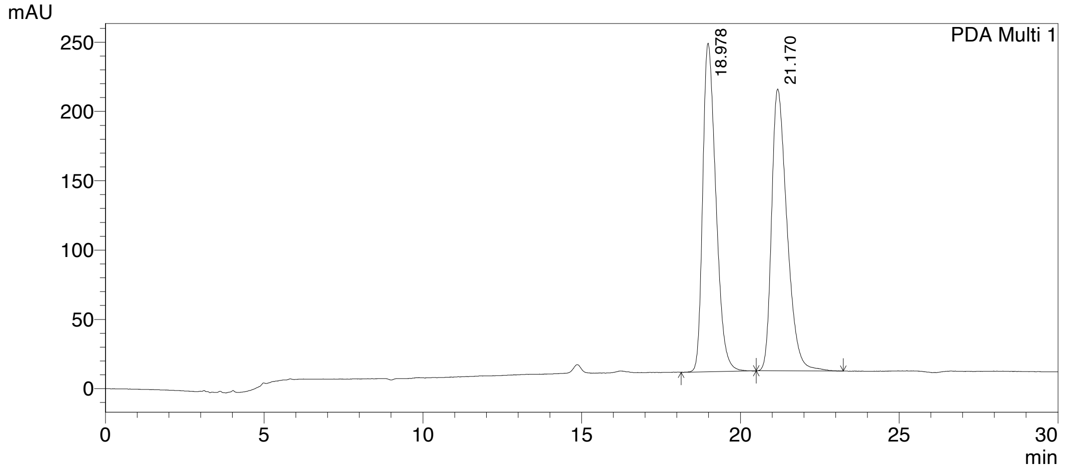

1 PDA Multi 1/254nm 4nm

PeakTable

PDA Ch1 254nm 4nm

Peak#	Ret. Time	Area	Height	Area %	Height %
1	18.978	6812376	237113	49.846	53.817
2	21.170	6854443	203476	50.154	46.183
Total		13666819	440589	100.000	100.000

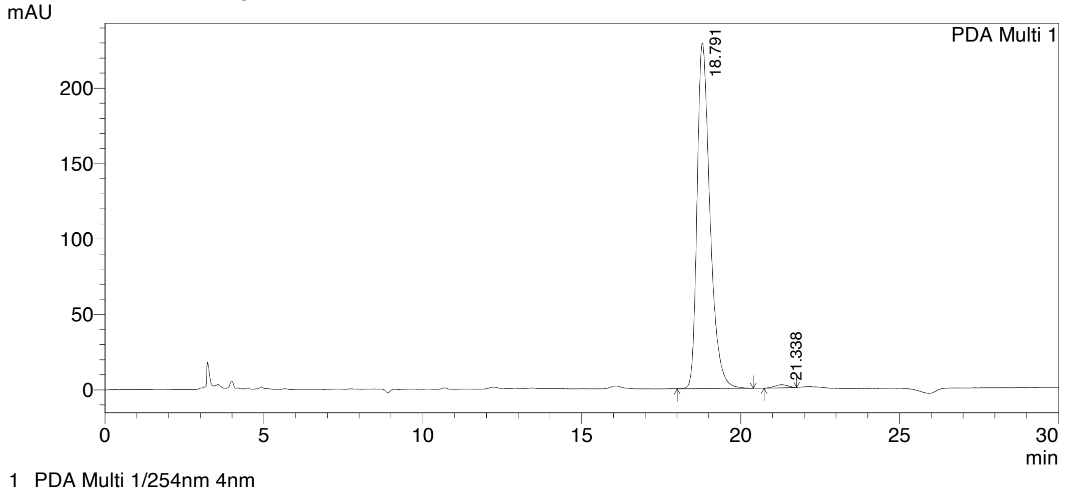

1 PDA Multi 1/254nm 4nm

PeakTable

PDA Ch1 254nm 4nm

Peak#	Ret. Time	Area	Height	Area %	Height %
1	18.791	6467128	229201	99.105	99.150
2	21.338	58435	1966	0.895	0.850
Total		6525563	231167	100.000	100.000

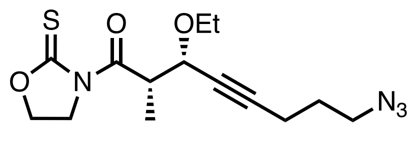

4h

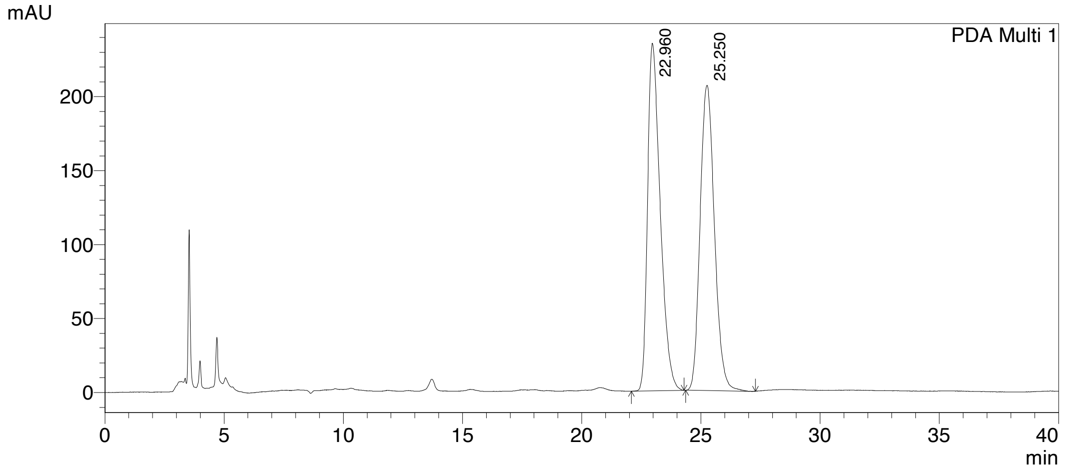

PeakTable

PDA Ch1 254nm 4nm

Peak#	Ret. Time	Area	Height	Area %	Height %
1	22.960	8665845	235065	50.154	53.260
2	25.250	8612495	206290	49.846	46.740
Total		17278341	441355	100.000	100.000

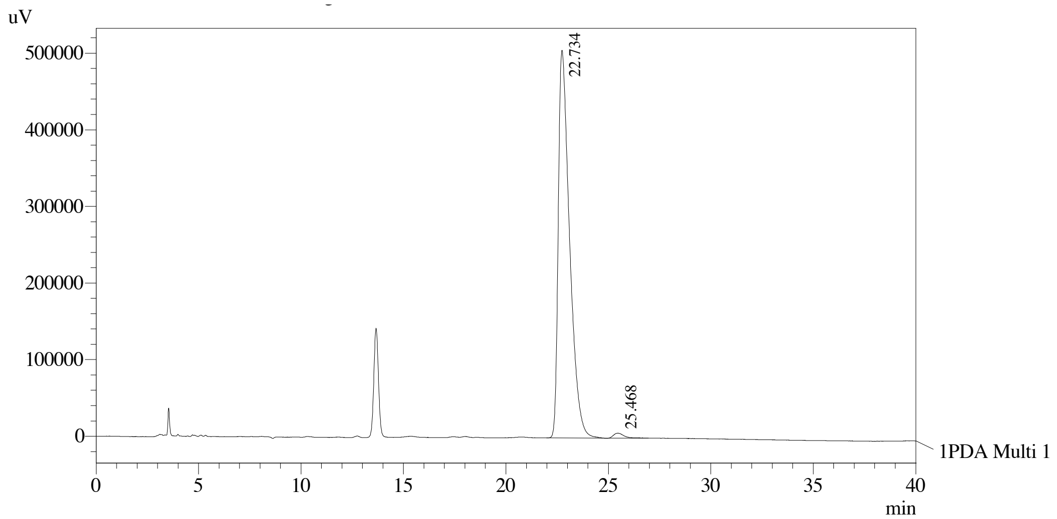

1 PDA Multi 1 / 254nm 4nm

PeakTable

PDA Ch1 254nm 4nm

Peak#	Ret. Time	Area	Height	Area %	Height %
1	22.734	19434194	506154	98.824	98.716
2	25.468	231252	6585	1.176	1.284
Total		19665446	512739	100.000	100.000

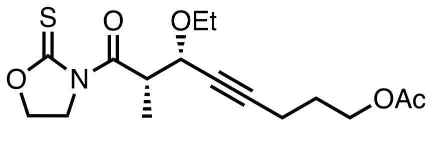

4i

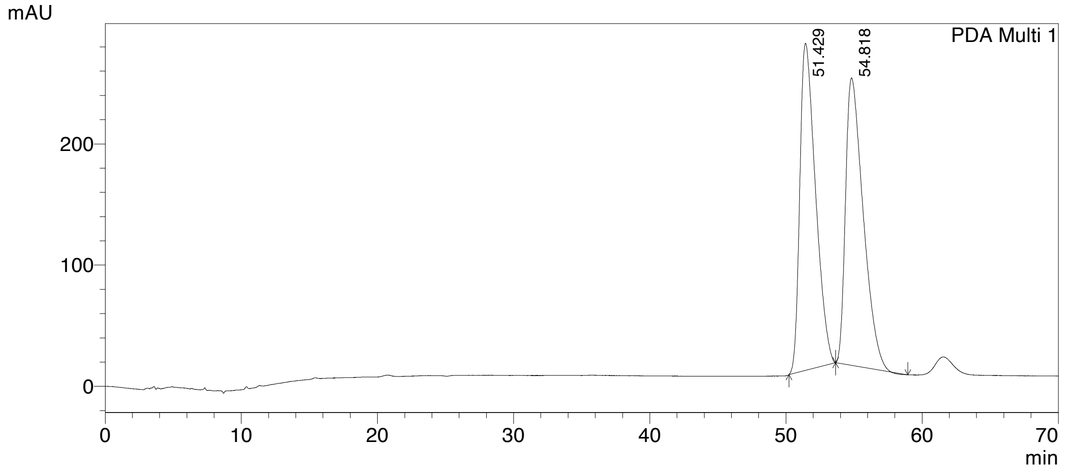

PeakTable

PDA Ch1 271nm 4nm

Peak#	Ret. Time	Area	Height	Area %	Height %
1	51.429	21621849	269885	50.216	53.223
2	54.818	21435550	237199	49.784	46.777
Total		43057399	507084	100.000	100.000

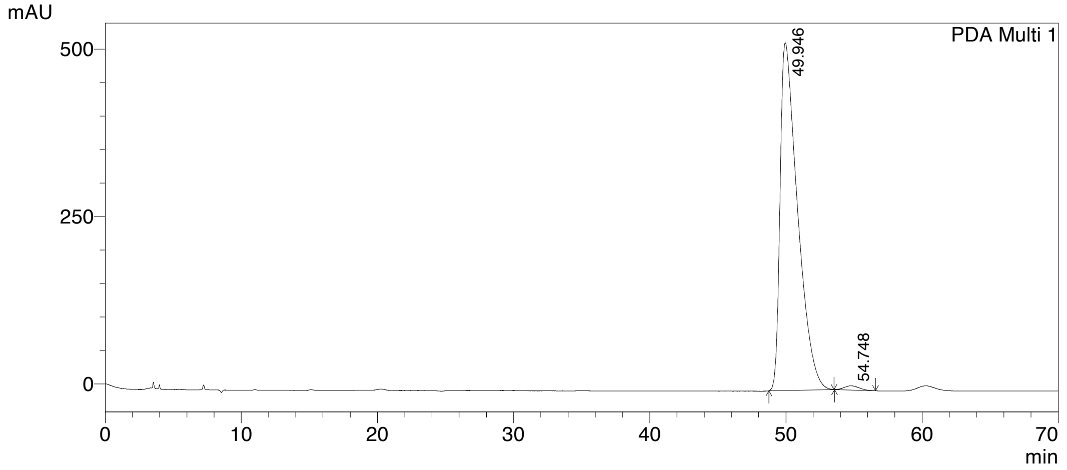

PeakTable

PDA Ch1 271nm 4nm

Peak#	Ret. Time	Area	Height	Area %	Height %
1	49.946	46322576	519534	98.974	98.805
2	54.748	480277	6281	1.026	1.195
Total		46802853	525814	100.000	100.000

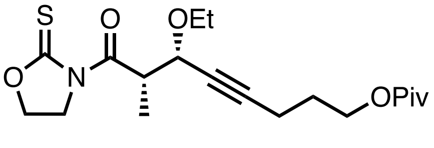

4j

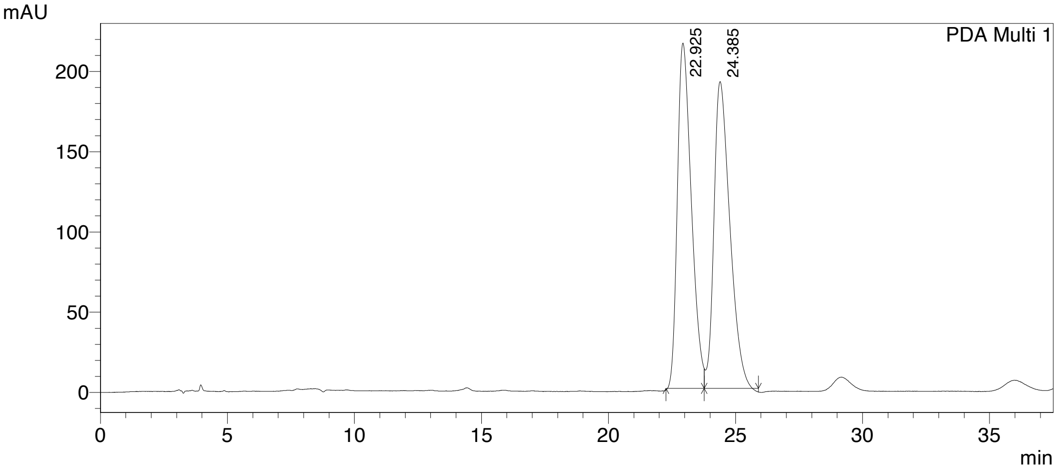

PeakTable

PDA Ch1 254nm 4nm

Peak#	Ret. Time	Area	Height	Area %	Height %
1	22.925	8193836	215085	49.563	52.960
2	24.385	8338453	191041	50.437	47.040
Total		16532288	406126	100.000	100.000

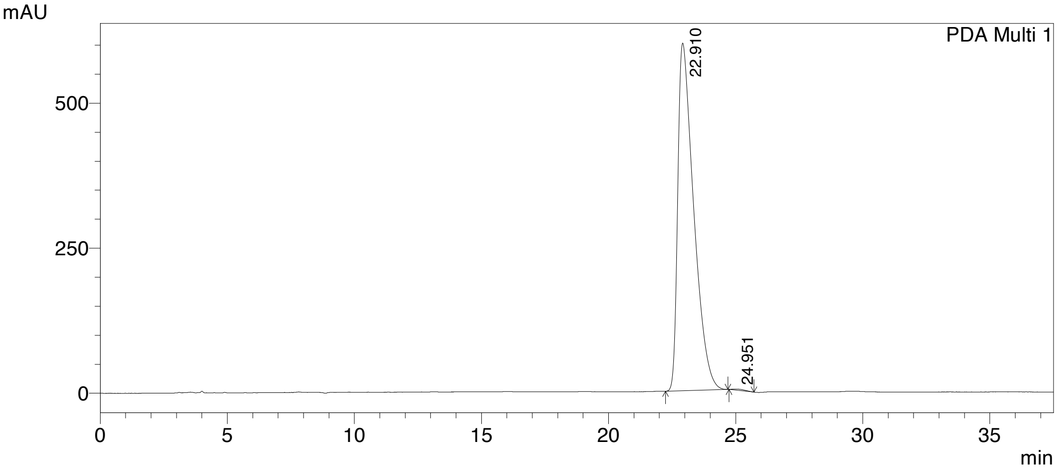

PeakTable

PDA Ch1 254nm 4nm

Peak#	Ret. Time	Area	Height	Area %	Height %
1	22.910	26084903	599418	99.726	99.717
2	24.951	71599	1702	0.274	0.283
Total		26156502	601121	100.000	100.000

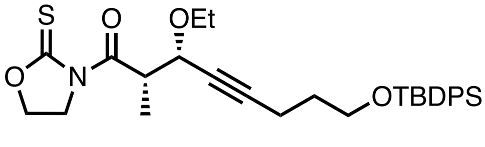

4k

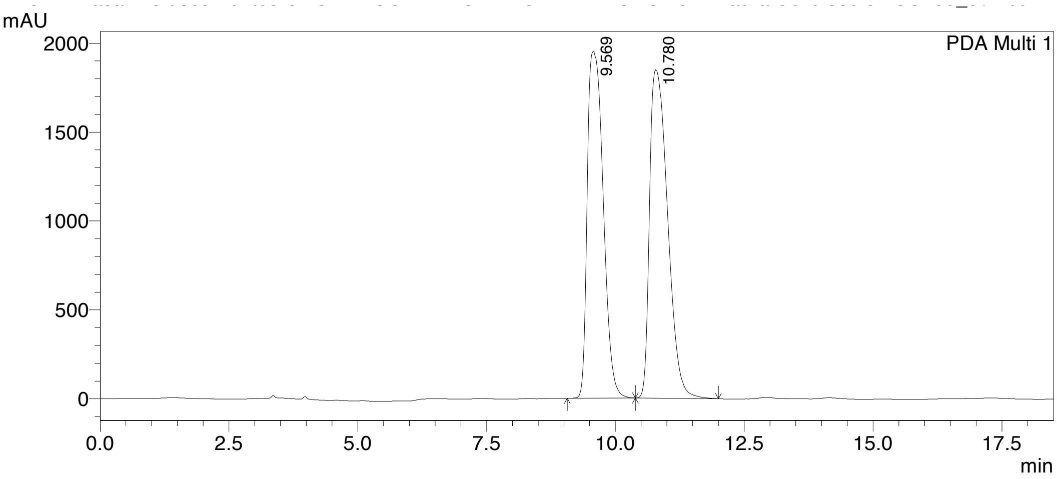

PeakTable

PDA Ch1 271nm 4nm

Peak#	Ret. Time	Area	Height	Area %	Height %
1	9.569	41808086	1950650	48.185	51.349
2	10.780	44957538	1848122	51.815	48.651
Total		86765624	3798772	100.000	100.000

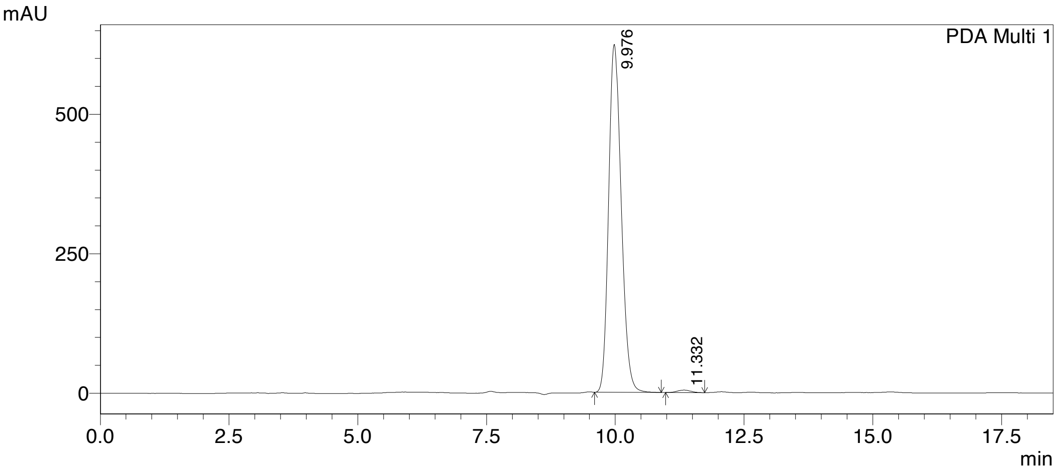

PeakTable

PDA Ch1 271nm 4nm

Peak#	Ret. Time	Area	Height	Area %	Height %
1	9.976	10588212	623627	99.239	99.293
2	11.332	81141	4440	0.761	0.707
Total		10669352	628067	100.000	100.000

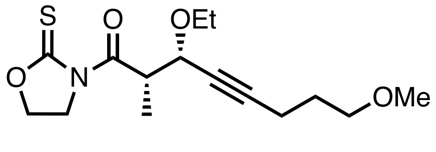

4l

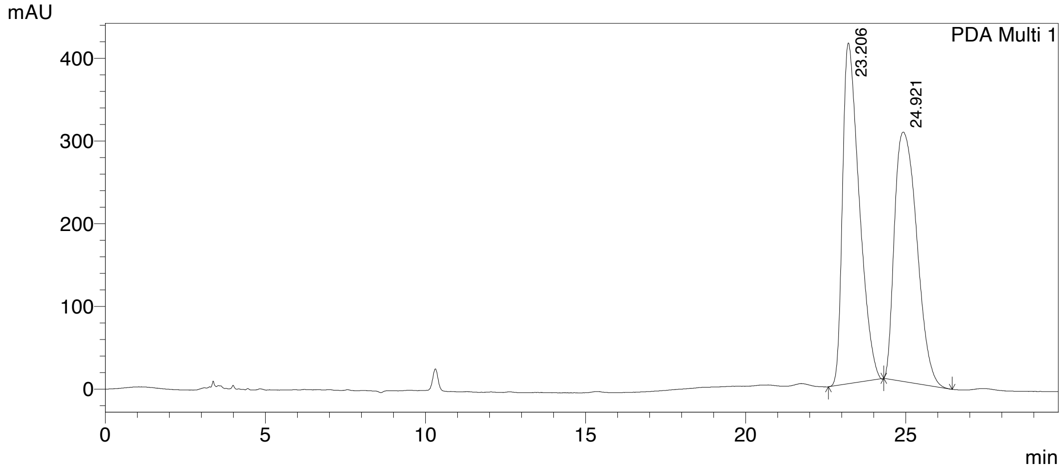

PeakTable

PDA Ch1 254nm 4nm

Peak#	Ret. Time	Area	Height	Area %	Height %
1	23.206	14913404	412279	50.388	57.741
2	24.921	14683572	301735	49.612	42.259
Total		29596976	714014	100.000	100.000

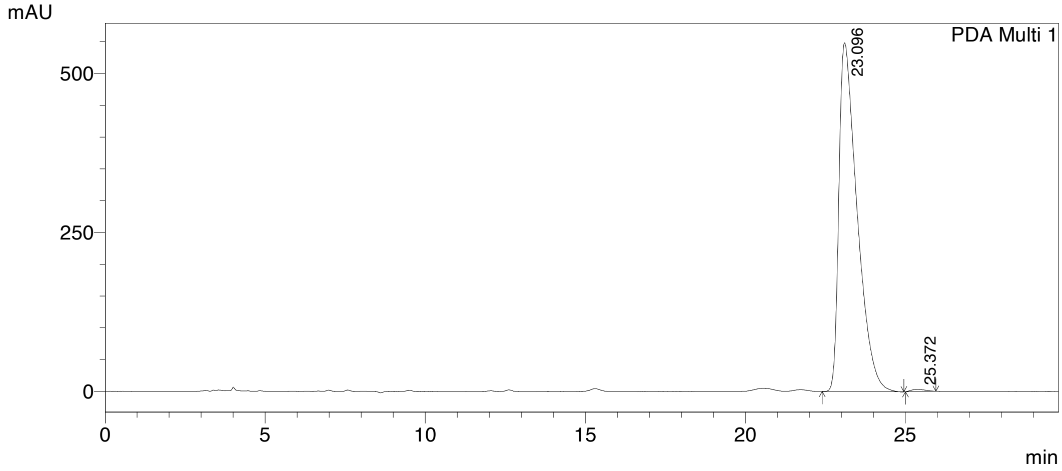

PeakTable

PDA Ch1 254nm 4nm

Peak#	Ret. Time	Area	Height	Area %	Height %
1	23.096	21694507	548681	99.549	99.398
2	25.372	98300	3323	0.451	0.602
Total		21792807	552004	100.000	100.000

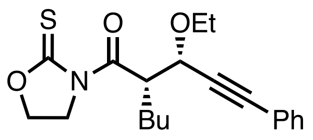

4m

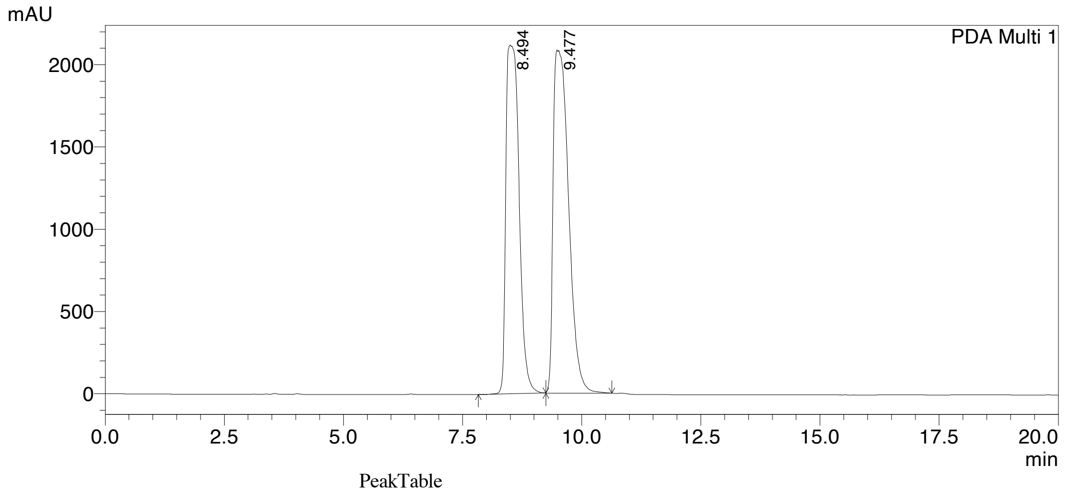

PeakTable

PDA Ch1 254nm 4nm

Peak#	Ret. Time	Area	Height	Area %	Height %
1	8.494	40922295	2120216	46.470	50.393
2	9.477	47139032	2087182	53.530	49.607
Total		88061327	4207398	100.000	100.000

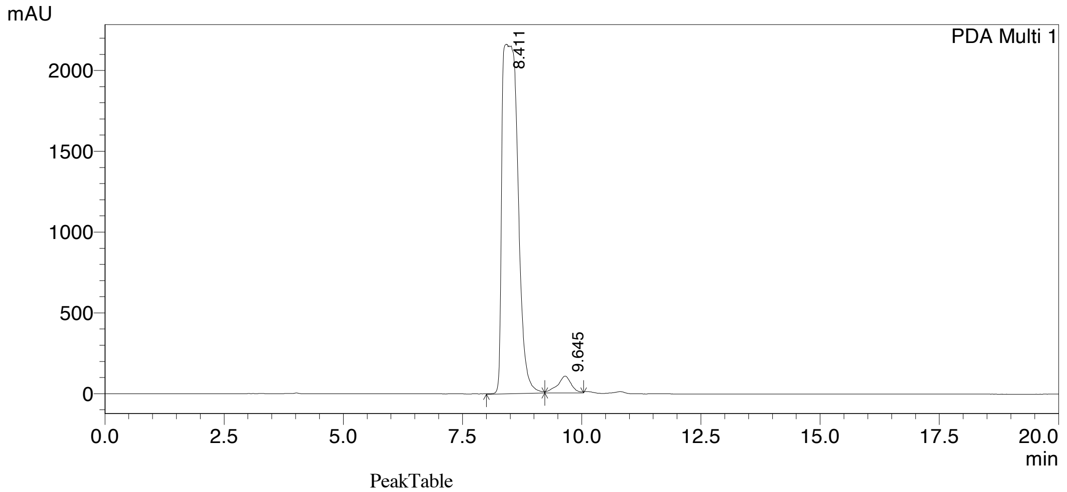

PeakTable

PDA Ch1 254nm 4nm

Peak#	Ret. Time	Area	Height	Area %	Height %
1	8.411	50801890	2163653	96.030	95.408
2	9.645	2100354	104145	3.970	4.592
Total		52902244	2267797	100.000	100.000

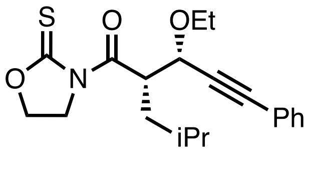

4n

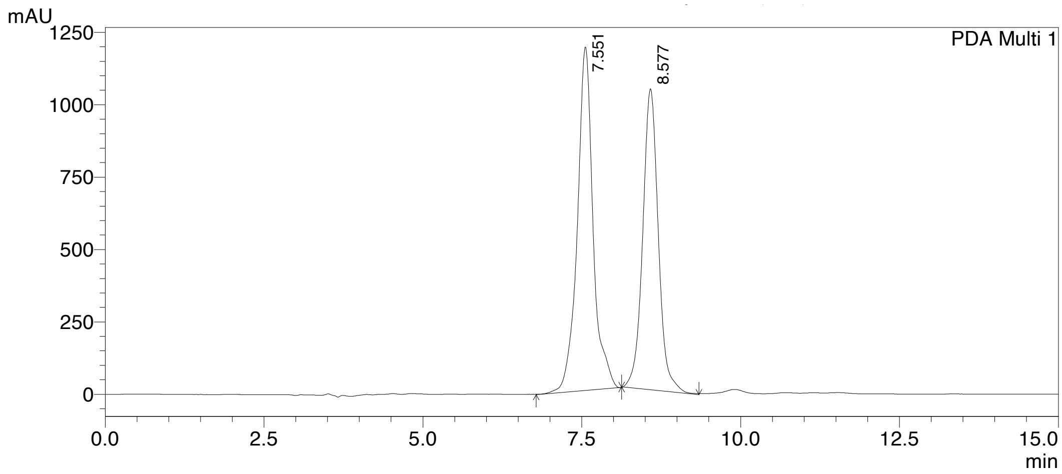

1 PDA Multi 1/254nm 4nm

PeakTable

PDA Ch1 254nm 4nm

Peak#	Ret. Time	Area	Height	Area %	Height %
1	7.551	20481798	1186000	53.475	53.316
2	8.577	17819798	1038475	46.525	46.684
Total		38301596	2224475	100.000	100.000

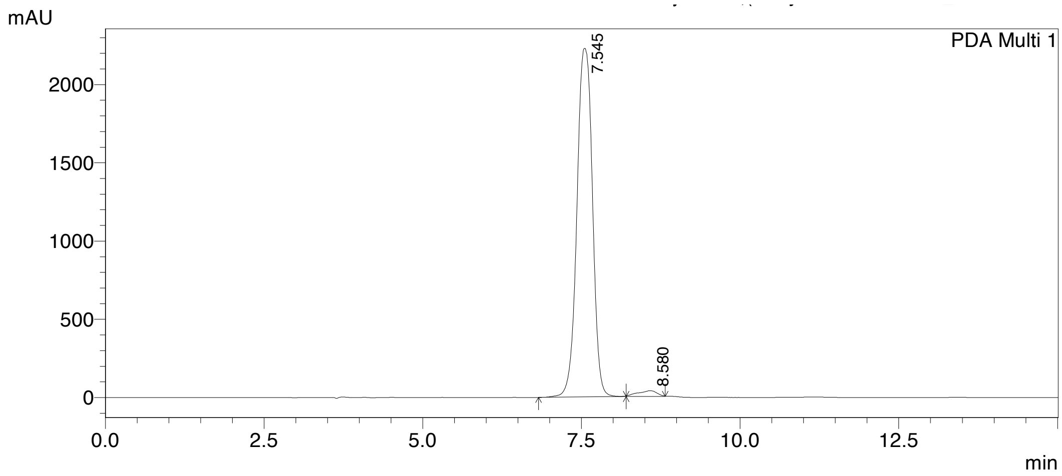

1 PDA Multi 1/254nm 4nm

PeakTable

PDA Ch1 254nm 4nm

Peak#	Ret. Time	Area	Height	Area %	Height %
1	7.545	37796613	2226795	98.035	98.394
2	8.580	757637	36347	1.965	1.606
Total		38554251	2263142	100.000	100.000

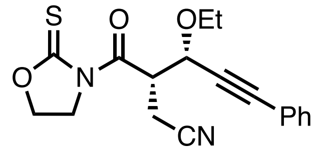

4o

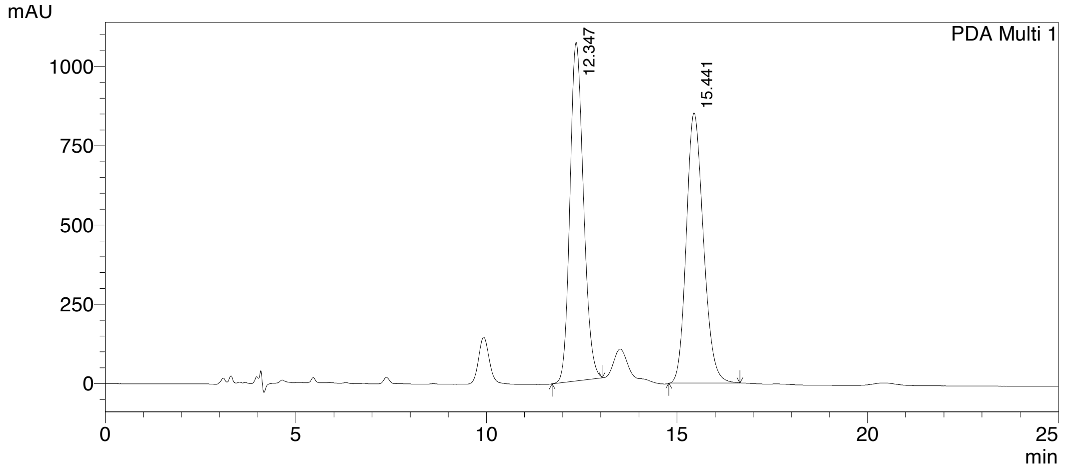

1 PDA Multi 1/231nm 4nm

PeakTable

PDA Ch1 231nm 4nm

Peak#	Ret. Time	Area	Height	Area %	Height %
1	12.347	26310279	1068354	49.770	55.639
2	15.441	26553789	851793	50.230	44.361
Total		52864068	1920147	100.000	100.000

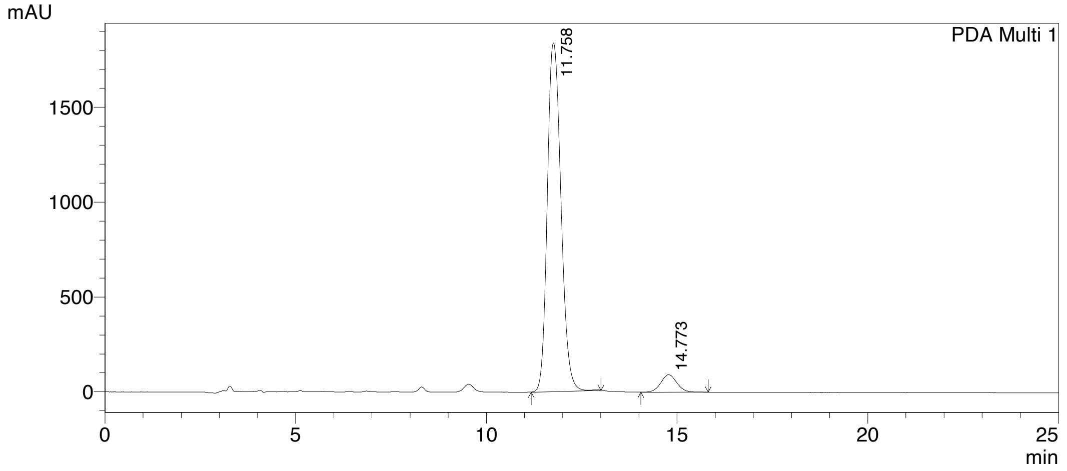

1 PDA Multi 1/231nm 4nm

PeakTable

PDA Ch1 231nm 4nm

Peak#	Ret. Time	Area	Height	Area %	Height %
1	11.758	45765210	1837001	94.586	95.253
2	14.773	2619771	91546	5.414	4.747
Total		48384981	1928547	100.000	100.000

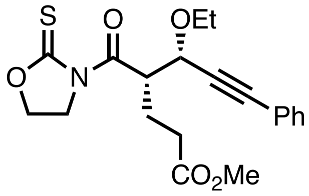

4p

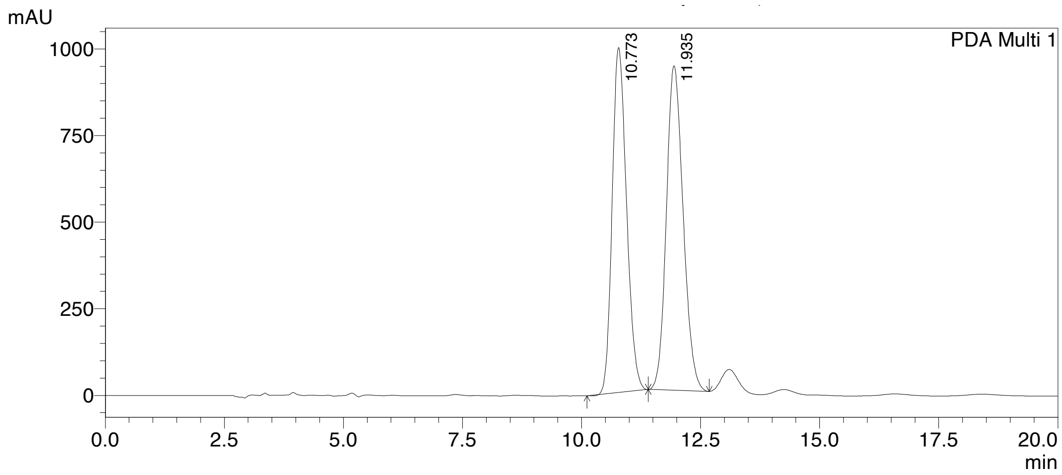

1 PDA Multi 1/254nm 4nm

PeakTable

PDA Ch1 254nm 4nm

Peak#	Ret. Time	Area	Height	Area %	Height %
1	10.773	20837191	995074	47.558	51.533
2	11.935	22976662	935886	52.442	48.467
Total		43813852	1930960	100.000	100.000

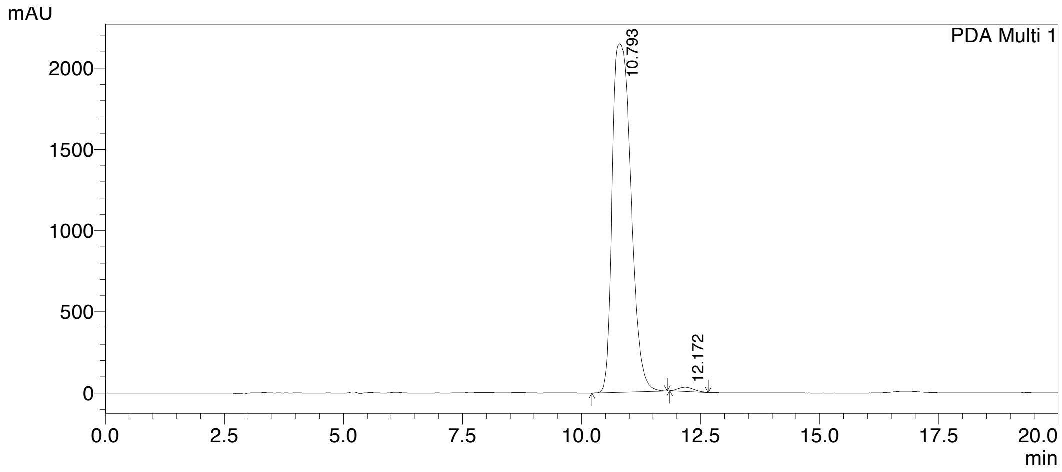

1 PDA Multi 1/254nm 4nm

PeakTable

PDA Ch1 254nm 4nm

Peak#	Ret. Time	Area	Height	Area %	Height %
1	10.793	58911884	2145904	99.000	98.813
2	12.172	595248	25778	1.000	1.187
Total		59507132	2171682	100.000	100.000

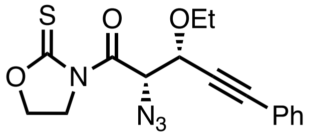

4q

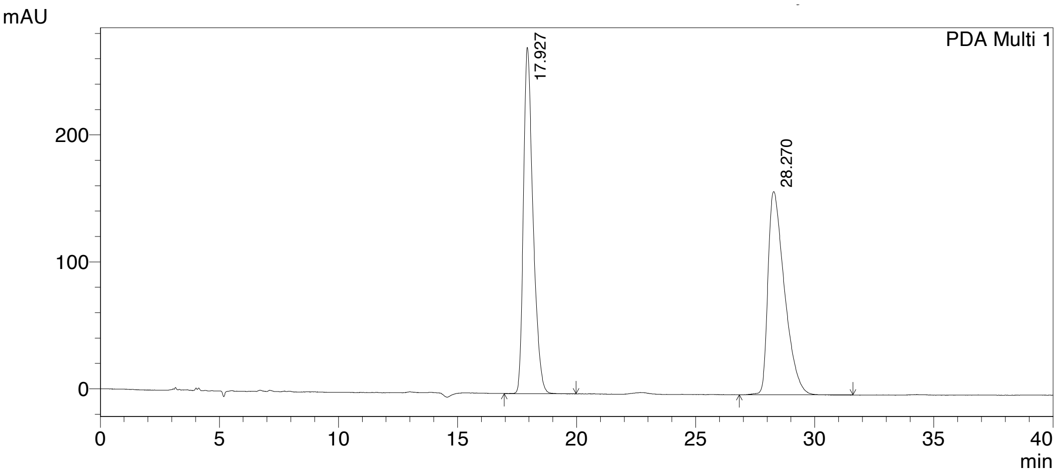

PeakTable

PDA Ch1 254nm 4nm

Peak#	Ret. Time	Area	Height	Area %	Height %
1	17.927	7623911	272731	49.751	63.020
2	28.270	7700223	160036	50.249	36.980
Total		15324134	432767	100.000	100.000

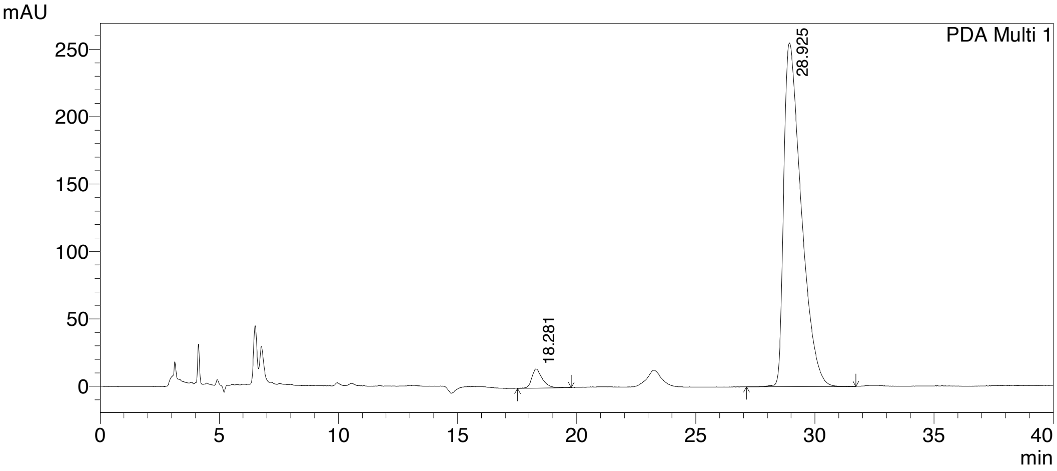

1 PDA Multi 1/254nm 4nm

PeakTable

PDA Ch1 254nm 4nm

Peak#	Ret. Time	Area	Height	Area %	Height %
1	18.281	434681	14191	3.233	5.271
2	28.925	13011870	255032	96.767	94.729
Total		13446551	269224	100.000	100.000

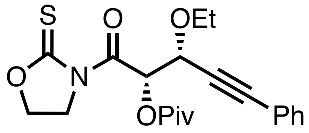

4r

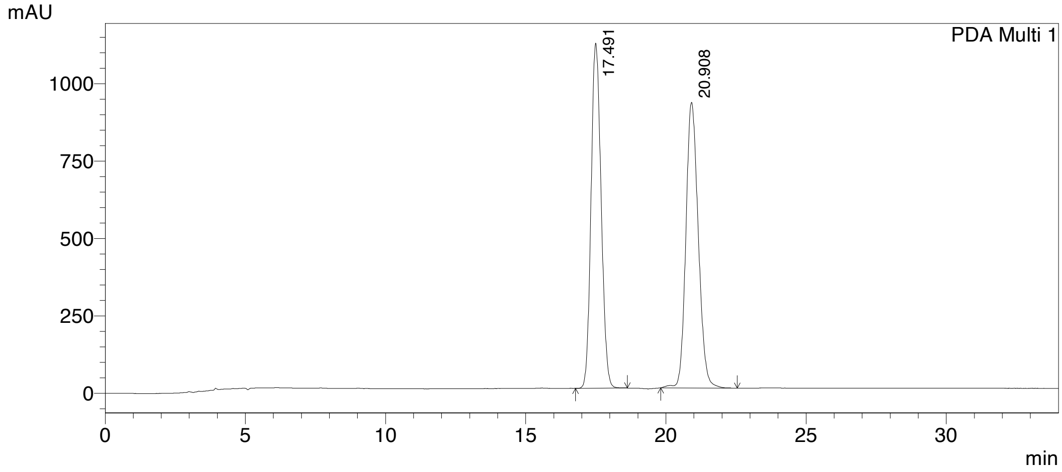

1 PDA Multi 1/254nm 4nm

PeakTable

PDA Ch1 254nm 4nm

Peak#	Ret. Time	Area	Height	Area %	Height %
1	17.491	27792999	1115238	49.435	54.726
2	20.908	28428528	922617	50.565	45.274
Total		56221527	2037855	100.000	100.000

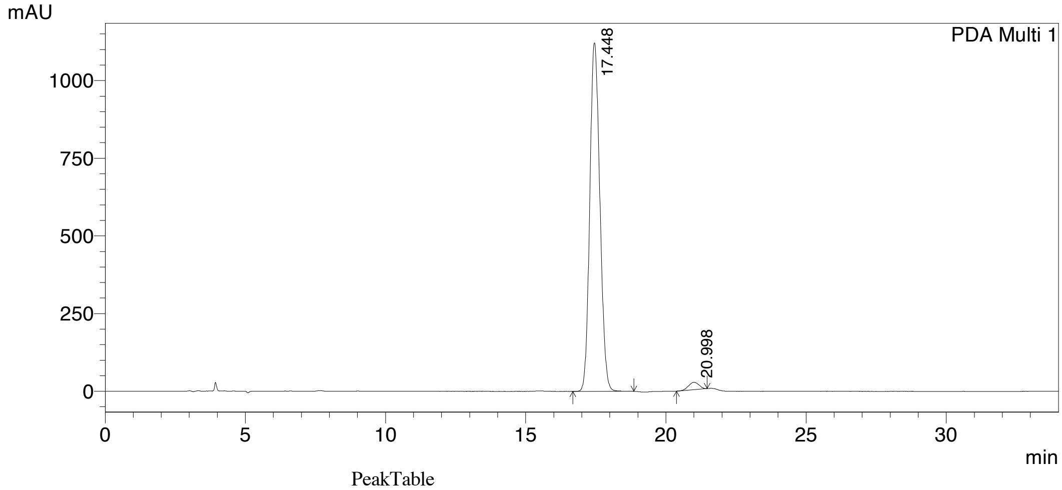

PeakTable

PDA Ch1 254nm 4nm

Peak#	Ret. Time	Area	Height	Area %	Height %
1	17.448	28099076	1122004	97.857	97.916
2	20.998	615479	23880	2.143	2.084
Total		28714556	1145883	100.000	100.000

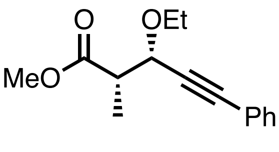

6a

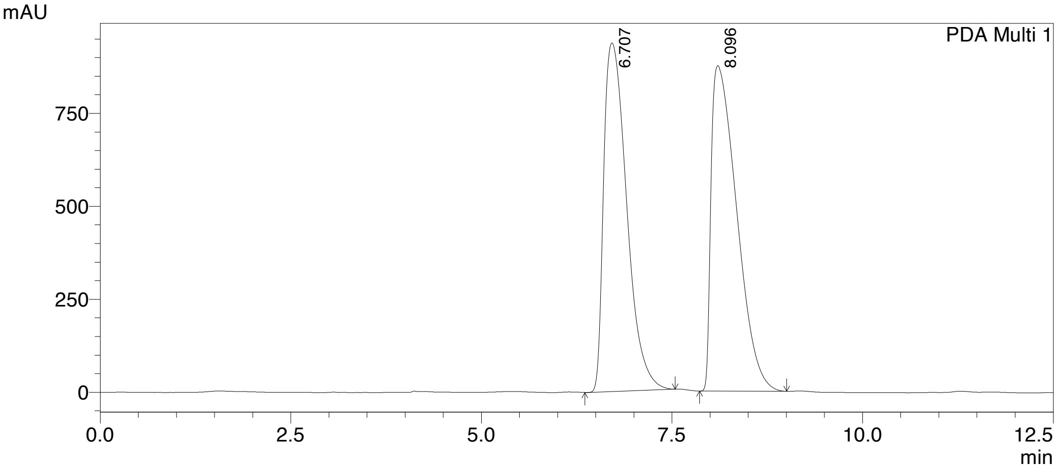

1 PDA Multi 1/254nm 4nm

PeakTable

PDA Ch1 254nm 4nm

Peak#	Ret. Time	Area	Height	Area %	Height %
1	6.707	19658054	937262	49.041	51.722
2	8.096	20427227	874849	50.959	48.278
Total		40085281	1812111	100.000	100.000

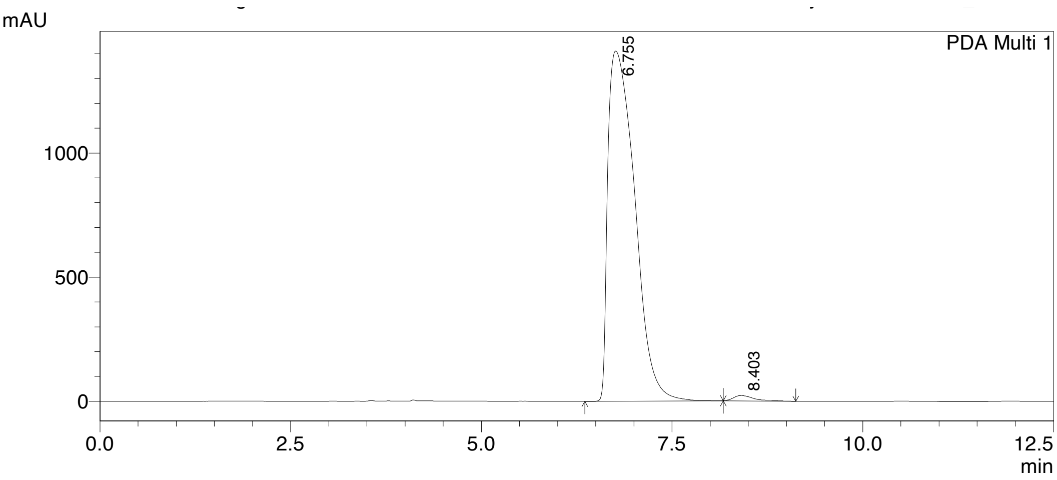

1 PDA Multi 1/254nm 4nm

PeakTable

PDA Ch1 254nm 4nm

Peak#	Ret. Time	Area	Height	Area %	Height %
1	6.755	34521279	1410480	98.824	98.472
2	8.403	410927	21884	1.176	1.528
Total		34932206	1432364	100.000	100.000

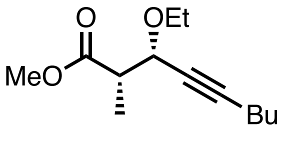

6e

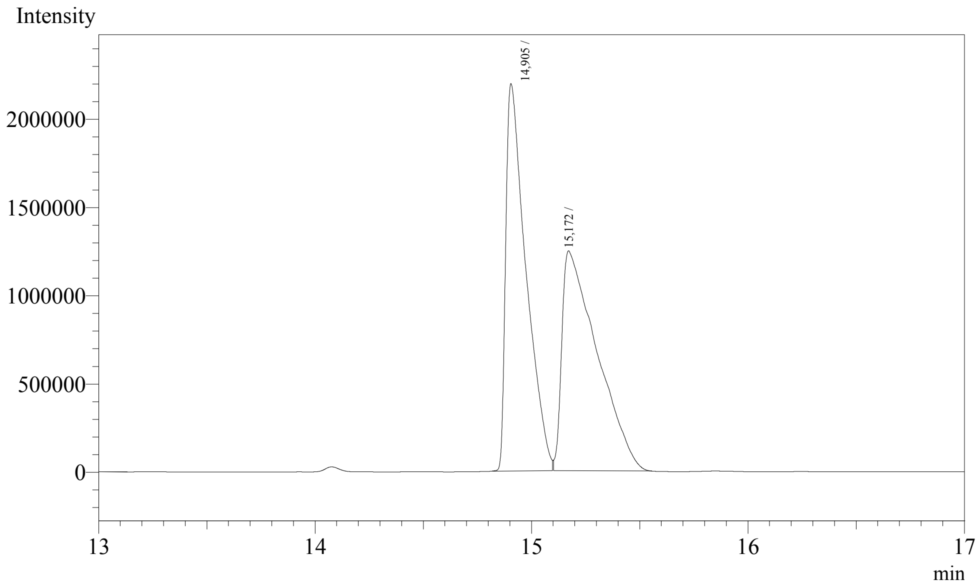

Peak#	Ret.Time	Area	Height	Conc.	Unit	Mark	ID#	Cmpd Name
1	14,905	14253897	2184957	50,296				
2	15,172	14086276	1242885	49,704				
Total		28340173	3427842					

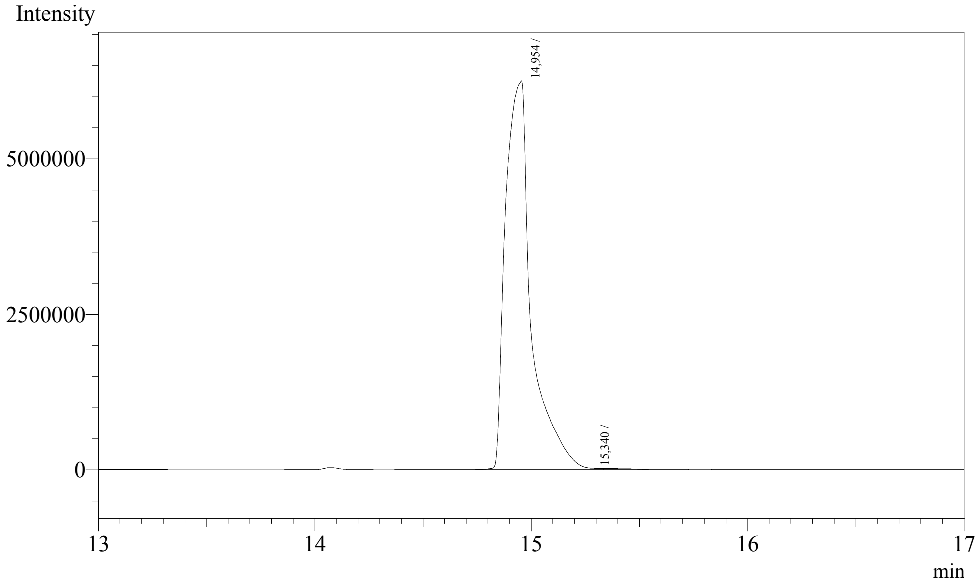

Peak#	Ret.Time	Area	Height	Conc.	Unit	Mark	ID#	Cmpd Name
1	14,954	51815610	6246311	99,733				
2	15,340	138575	17149	0,267				
Total		51954185	6263460					

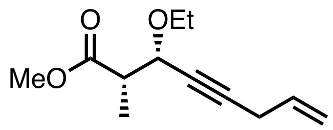

6f

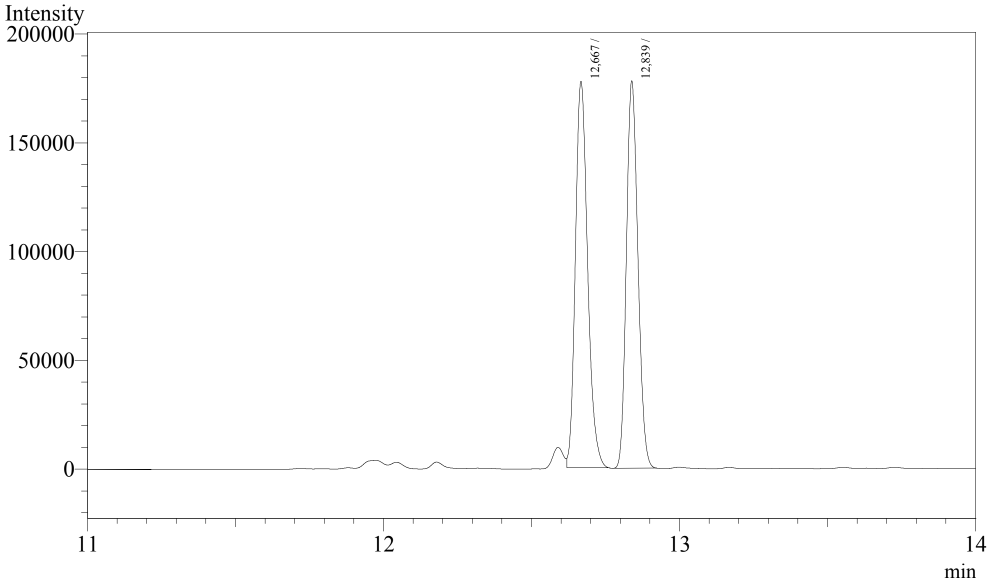

Peak#	Ret.Time	Area	Height	Conc.	Unit	Mark	ID#	Cmpd Name
1	12,667	509373	176675	51,404				
2	12,839	481540	175727	48,596				
Total		990913	352402					

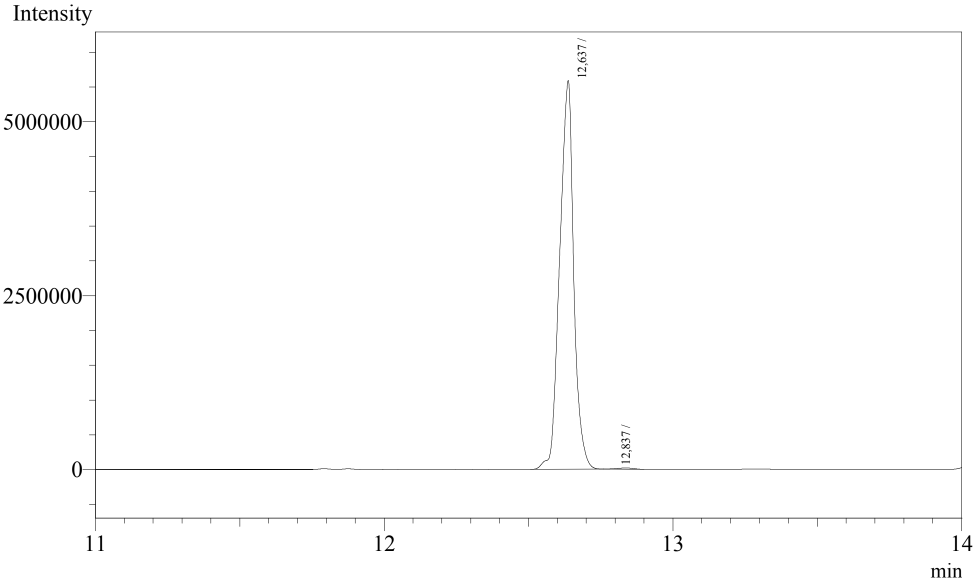

Peak#	Ret.Time	Area	Height	Conc.	Unit	Mark	ID#	Cmpd Name
1	12,637	18880826	5511338	99,633				
2	12,837	69501	18043	0,367				
Total		18950327	5529381					

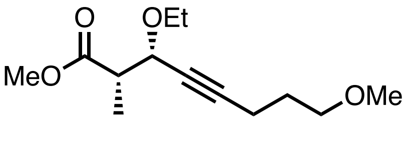

6l

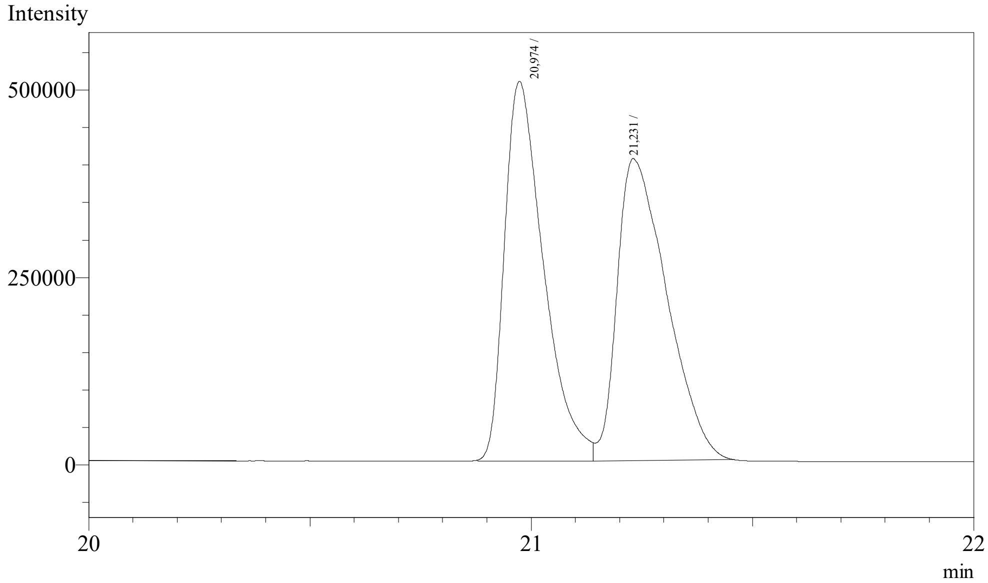

Peak#	Ret.Time	Area	Height	Conc.	Unit Mark	ID#	Cmpd Name
1	20,974	3121319	506422	49,772			
2	21,231	3149947	403053	50,228			
Total		6271266	909475				

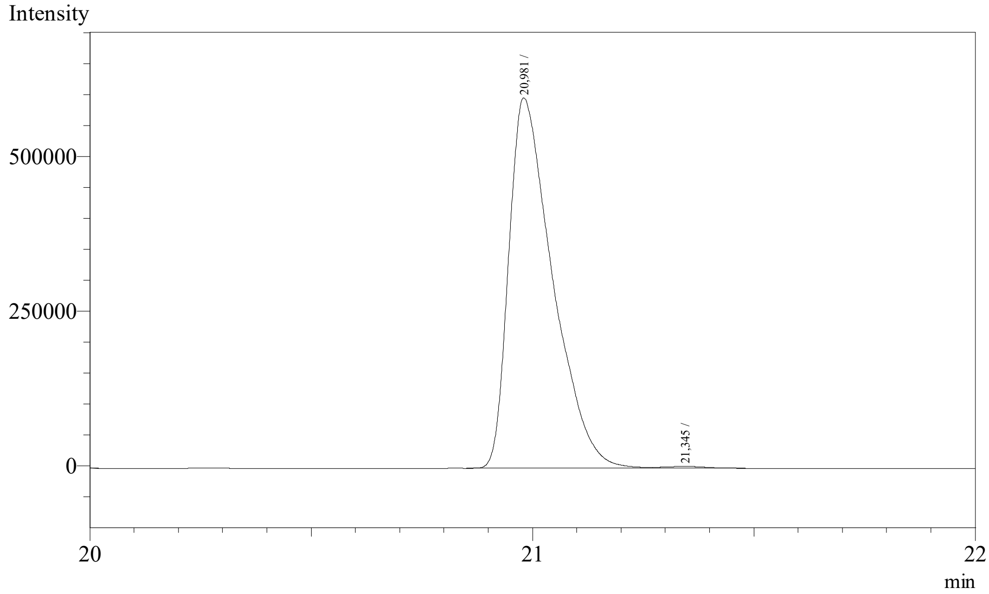

Peak#	Ret.Time	Area	Height	Conc.	Unit Mark	ID#	Cmpd Name
1	20,981	4111166	594723	99,594			
2	21,345	16751	2742	0,406			
Total		4127917	597465				

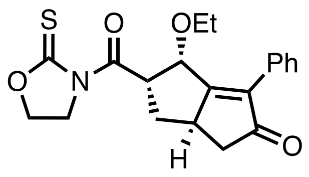

10a

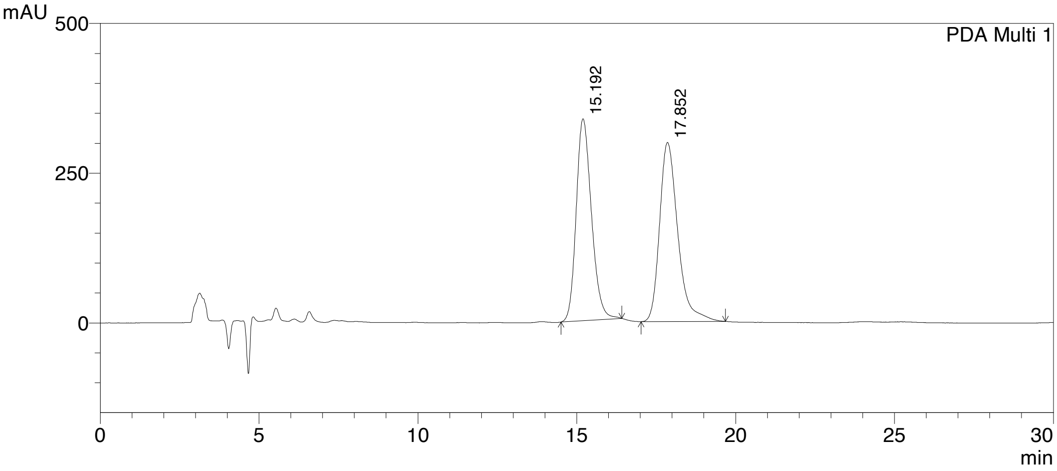

PeakTable

PDA Ch1 271nm 4nm

Peak#	Ret. Time	Area	Height	Area %	Height %
1	15.192	11241410	337282	48.537	52.991
2	17.852	11919086	299204	51.463	47.009
Total		23160496	636486	100.000	100.000

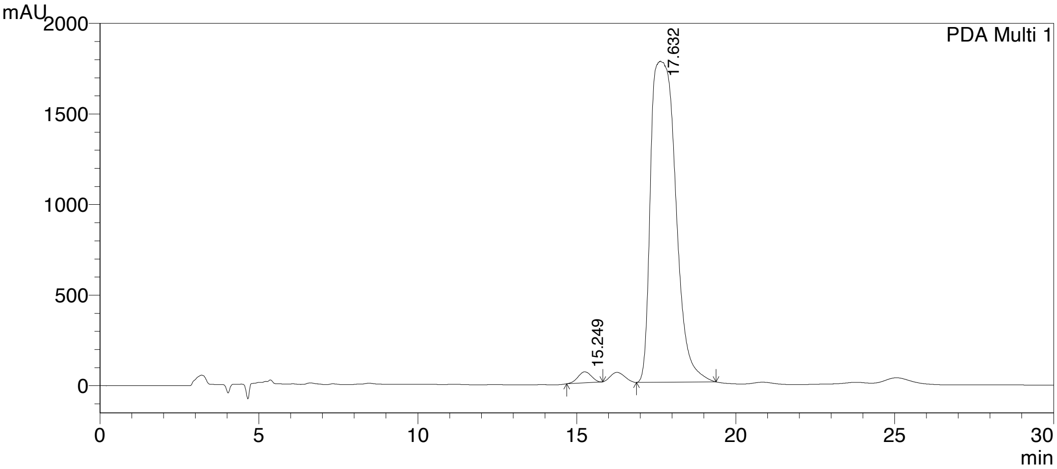

PeakTable

PDA Ch1 271nm 4nm

Peak#	Ret. Time	Area	Height	Area %	Height %
1	15.249	1750200	60427	1.765	3.297
2	17.632	97383862	1772545	98.235	96.703
Total		99134061	1832971	100.000	100.000

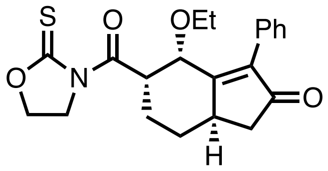

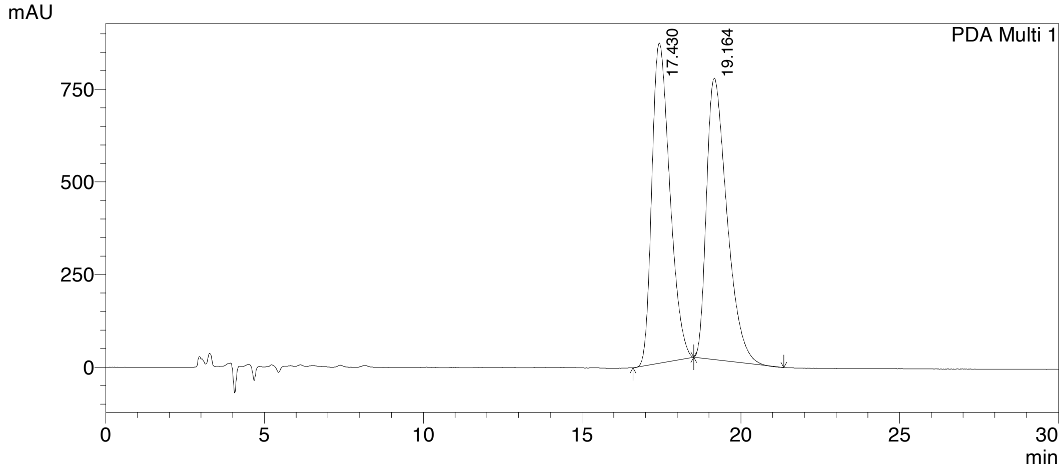

1 PDA Multi 1/254nm 4nm

PeakTable

PDA Ch1 254nm 4nm

Peak#	Ret. Time	Area	Height	Area %	Height %
1	17.430	34017308	863919	50.017	53.216
2	19.164	33994053	759508	49.983	46.784
Total		68011361	1623427	100.000	100.000

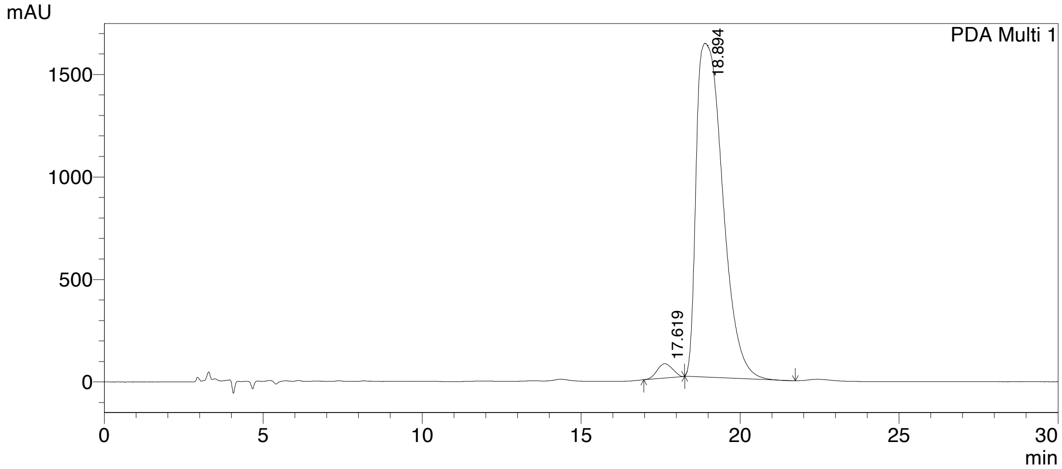

1 PDA Multi 1/254nm 4nm

PeakTable

PDA Ch1 254nm 4nm

Peak#	Ret. Time	Area	Height	Area %	Height %
1	17.619	2318895	69517	2.406	4.095
2	18.894	94063920	1628056	97.594	95.905
Total		96382815	1697573	100.000	100.000
